# Prediction of cassava protein interactome based on interolog method

**DOI:** 10.1038/s41598-017-17633-2

**Published:** 2017-12-08

**Authors:** Ratana Thanasomboon, Saowalak Kalapanulak, Supatcharee Netrphan, Treenut Saithong

**Affiliations:** 1 Biological Engineering Program, Faculty of Engineering, King Mongkut’s University of Technology Thonburi, Bangkok, 10140 Thailand; 2 Systems Biology and Bioinformatics Research Group, Pilot Plant Development and Training Institute, King Mongkut’s University of Technology Thonburi (Bang Khun Thian), Bangkok, 10150 Thailand; 3 Bioinformatics and Systems Biology Program, School of Bioresources and Technology, King Mongkut’s University of Technology Thonburi (Bang Khun Thian), Bangkok, 10150 Thailand; 4 National Center for Genetic Engineering and Biotechnology, Pathum Thani, 12120 Thailand

## Abstract

Cassava is a starchy root crop whose role in food security becomes more significant nowadays. Together with the industrial uses for versatile purposes, demand for cassava starch is continuously growing. However, in-depth study to uncover the mystery of cellular regulation, especially the interaction between proteins, is lacking. To reduce the knowledge gap in protein-protein interaction (PPI), genome-scale PPI network of cassava was constructed using interolog-based method (MePPI-In, available at http://bml.sbi.kmutt.ac.th/ppi). The network was constructed from the information of seven template plants. The MePPI-In included 90,173 interactions from 7,209 proteins. At least, 39 percent of the total predictions were found with supports from gene/protein expression data, while further co-expression analysis yielded 16 highly promising PPIs. In addition, domain-domain interaction information was employed to increase reliability of the network and guide the search for more groups of promising PPIs. Moreover, the topology and functional content of MePPI-In was similar to the networks of Arabidopsis and rice. The potential contribution of MePPI-In for various applications, such as protein-complex formation and prediction of protein function, was discussed and exemplified. The insights provided by our MePPI-In would hopefully enable us to pursue precise trait improvement in cassava.

## Introduction

Proteins are macromolecules that play crucial roles in a range of biological processes in cells. They do not only act as catalysts but are also involved in intracellular regulatory processes, *e.g*. signal transduction and transcriptional regulation^1^. Although specific function is assigned to each protein, too often, we see that the protein remains inactive in the cellular matrix. This is because the protein needs to go through some modification processes, such as protein dimerization and complex formation via protein binding. Cooperation between proteins, called protein-protein interaction (PPI), allows cells to dynamically modulate when proteins and their counterparts are turned on to play roles in particular cellular processes. Since these interactions are highly dependent on prevailing conditions of exposure, the PPI is considered a type of biological language utilized to synchronize cellular regulation, especially at post-translational level.

Due to the immense impact of PPIs on the regulation of cellular processes, great attempts have been devoted to capture the interactions between proteins as well as investigate their consequences. Earlier, availability of experimental techniques, such as affinity chromatography, immunoprecipitation and chemical crosslinking, only allowed for investigation of interaction of one, or maximally, a few protein pair at once^2^. Nowadays, large-scale detection of PPIs at genome-wide level has been made possible through yeast two-hybrid (Y2H), or affinity purification coupled with mass spectrometry (AP-MS)^3–5^. With these techniques, however, it still seems impossible to acquire knowledge in protein science at the rate that is fast enough to catch up with the big data currently available in post-genomic era.

Computational inference technique is an alternative method that can be used to identify the interactions between proteins. This technique is based on the hypothesis that the function of proteins, and also the interaction among them are conserved across their evolutionary lineage. By incorporating various types of data, such as amino acid sequences, functional domains, folding structure and co-evolution of interacting sites, successes in PPI conjecture in a wide range of organisms have been reported^6–9^. The information acquired from PPI prediction has helped expand the understanding of the regulation at protein level, such as PPI-based mechanism of signal transduction process, and molecular interaction underlying host-pathogen relationship^10–12^. Recently, in an effort to move beyond the available PPI data, sophisticated computational methods, such as machine learning^13,14^, Bayesian network^15,16^, physical docking^17^ and correlated mutation^18^, have been introduced. To approach genome-wide prediction, machine learning based methods are of wide interest. Series of effective algorithms have continuously been developed in an attempt to incorporate multiple genomic/proteomic features into a framework of PPI prediction^19–22^. These algorithmic methods predict PPI from amino acid sequences and their collective information, for instance evolutionary background. Some examples include support vector machine (SVM)^13,14,23^, rotation forest and decision tree^24,25^, Bayesian classification^15,16^, Naïve Bayes^26^, relevance vector machine (RVM)^27,28^ and weighted sparse representation (WSRC)^29,30^. These computational algorithms have contributed immensely to the study of PPI in a broad range of organisms, from bacteria^16^ to humans^31^. Nonetheless, the performance of the machine learning based-methods depends enormously on the numbers and quality of the employed data, especially the model-training information which are in general related to experimentally measured data. Application of such methods in non-model organisms, which always lack data, are quite challenging. To closely investigate the interaction of a protein set, computational methods that include information on protein structure into prediction regime, such as Struct2Net^32^ and physical docking^17^ are proposed.

In plants, earlier studies of PPI were limited to only a few species. The current PPI information of plants, especially cassava, has constrained choices of predictive methods. Not only the number of data is small, but only few experimental evidences are available. The computational prediction methods such as classification, machine learning and statistical inference were considered not suitable, and sometimes not applicable, for the status of data resource. A simple computational technique called interolog, which relies on existing data, is often adopted for PPI prediction under this restriction. The interolog method is inspired by the hypothesis that the function of protein is retained and passed through their orthologs in evolution-related organisms. The method, basically, infers PPI information from other well-studied species by orthology-based deduction. Most of PPI networks in plants were basically developed based on the interolog method, for example Arabidopsis^6^, rice^33^ and tomato^34^. The first plant PPI network constructed by interolog-based method was reported in 2007, describing 19,979 interactions of 3,617 Arabidopsis proteins^35^. Since the first publication was released, accuracy of the interolog-based PPI network has greatly been improved by integrating various sources of information, from both predictive studies and experimental measurements^36–38^. Presently, the PPI research of plant species has been expanded from model plants, *e.g*. Arabidopsis^6,35–39^ and rice^9,33,40^, to economic crops, *e.g* tomato^34^, maize^41^ and sweet orange^42^. The previous research works are the good evidences of the appropriateness of such method for PPI inference in plants.

Cassava (*Manihot esculenta* Crantz) is an important crop of the world, since its roots feed at least 800 million people^43^. Cassava yield improvement is thus, a major research topic ultimately aiming to guarantee food sufficiency for growing population. To date, the advent of high-throughput technology has improved our understanding of various aspects of cassava, especially root development^44,45^ and starch biosynthesis^46–48^. However, the information at protein level, which drives the physiology of cassava, is still a mystery. Several reports on protein expression exist under the conditions of interest^49–59^, but they do not provide further information on PPIs that might relate to post-translational or protein-level regulation. Here, by employing interolog approach, we propose the first genome-scale protein-protein interaction network of cassava (MePPI-In), using available PPI data and information from a variety of plant species. Our MePPI-In contains 90,173 interactions interconnecting 7,209 cassava proteins (approximately 21 percent of all proteins in the whole genome). These interactions were partially supported by protein/gene expression and domain-domain interaction data. The resulting PPI network provided the landscape of possible interactions that might help fill the knowledge-gap on post-translational regulation in cassava as exemplified in the last section.

## Methods

### Construction of cassava PPI network using interolog-based method

**Figure 1:**
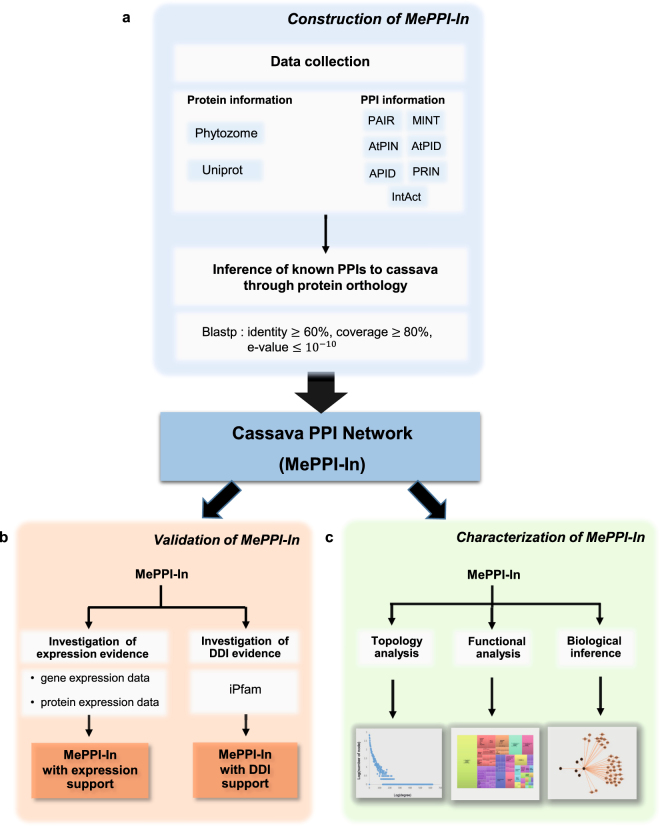
Overall methodology of PPI prediction in cassava consisting of three parts: (**a**) Construction of protein-protein interaction network of cassava using interolog-based approach (MePPI-In), (**b**) Validation of the PPIs proposed in MePPI-In using protein/gene expression or domain-domain interaction (DDI) evidence, and (**c**) Characterization of MePPI-In based on three aspects – network topology, functional contents and biological inference.

The interolog method is generally based on the inference of PPI information known to exist in other organisms. In this study, plant species, whose PPI information was employed for inference, were selected based on one of these criteria; (1) having a closed evolution with cassava (*i.e. Ricinus communis* (castor bean), *Populus trichocarpa* (poplar) and *Glycine max* (soybean)), (2) being recognized as a starch-storing plant (*i.e. Solanum tuberosum* (potato), *Zea mays* (maize) and *Oryza sativa* (rice)), or (3) having abundant PPI information (*i.e. Arabidopsis thaliana*). The protein information of these template plants was obtained from Phytozome V9^60^ and Uniprot^61^ databases, and the protein interaction information was collected from seven databases; IntAct^62^, MINT^63^, AtPIN^36^, AtPID^37^, PAIR^38^, APID^39^, and PRIN^9^ (Fig. 1a). To find protein orthologs in cassava, we performed BLASTp search against the cassava genome sequence. The cassava orthologous proteins were identified if the identity percentage ≥ 60, coverage percentage ≥ 80 and e-value ≤ 10^−10^. To be able to infer interaction that originally exists in one of the plant templates to cassava, orthologous proteins interconnected by such interaction must be identified in cassava. The cassava PPI network, MePPI-In, was then visualized using Cytoscape software^64^.

### Validation of MePPI-In based on expression data

**Figure 2:**
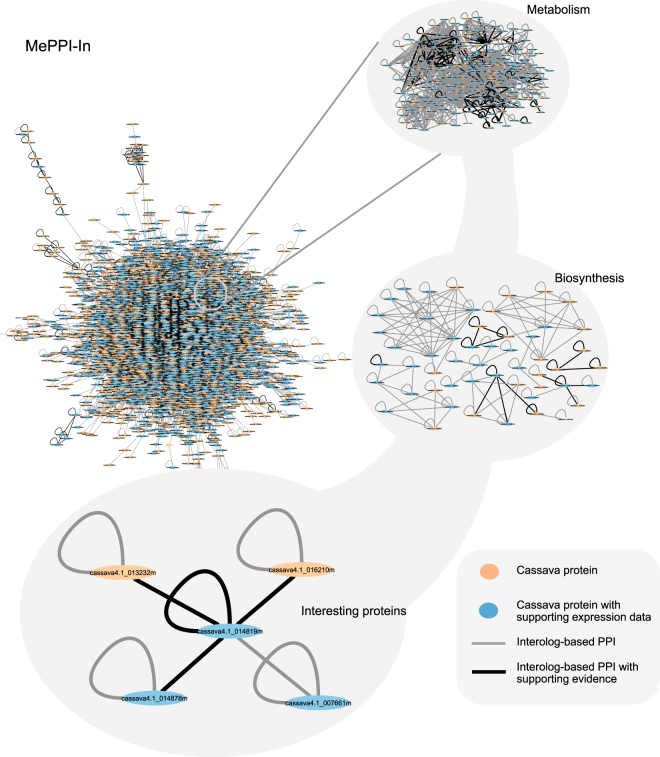
Cassava protein-protein interaction network (MePPI-In) derived by the interolog-based method. The network contained 90,173 interactions (edges) interconnecting 7,209 proteins (nodes). Different colors were given to both edges and nodes. The black edges represent PPIs with DDI or co-expression support, while the grey ones represent those with no supporting data. Blue color nodes represent proteins with supporting expression data^45,50–53,56,57,59,65–67^, while the orange ones have no expression support. (All information is publicly available at http://bml.sbi.kmutt.ac.th/ppi).

Protein and gene expression data were exploited in this prediction framework to directly indicate if the proteins, or products of genes, exist in real cassava system. Afterwards, the proteins were considered available for the interactions among them to occur. To perform this, we utilized seven sets of protein expression data (Li *et al*.^50^, Mitprasat *et al*.^51^, Naconsie *et al*.^52^, Otiwi *et al*.^53^, Sheffield *et al*.^56^, Vanderschuren *et al*.^57^ and Zhao *et al*.^59^), and four sets of gene expression data (Yang *et al*.^45^, Li *et al*.^65^, An *et al*.^66^ and Utsumi *et al*.^67^). For the gene expression data, it is important to note that only highly expressed genes that showed expression level above the 80^th^ percentile rank were used. In MePPI-In, the nodes (proteins) were highlighted as blue color, if their expression information was available, as shown in Fig. 2.

From eleven datasets mentioned above, only the time-series datasets of Naconsie *et al*.^52^, Yang *et al*.^45^, Li *et al*.^65^ and An *et al*.^66^ were employed to investigate the correlation of the expression profiles for interacting protein pair. This was based on the hypothesis that the genes/proteins with co-expression profile would have greater probability to interact than uncorrelated ones. Herein, the expression profiles of the highly expressed genes were determined based on Pearson’s correlation (Pearson correlation coefficient score (*PCC*))^68^, and co-expression of a protein pair in MePPI-In was suggested at *PCC* > 0.9 and *p*-value < 0.1.

### Validation of MePPI-In based on domain-domain interaction (DDI) data

Since proteins always interact via specific domains, the reliability of predicted PPIs could be determined using information on domain-domain interaction (DDI). In this work, the domain information of all proteins in the cassava PPI network was obtained from Pfam database^69^ and the interactions between protein domains were collected from iPfam database^70^ (Fig. 1b). From the original MePPI-In, different colors were given to the interactions (edges) with or without supporting DDI information as shown in Fig. 2.

### Scoring the confidence of the MePPI-In

The confidence of predicted PPIs in MePPI-In was determined based on the consistency of the results inferred by two methods: interolog and domain-domain interaction analyses. Each predicted interaction was given the level of confidence, in terms of confidence value (*CV*). The confidence value (*CV*) score was defined as a product of the confidence values from interolog (*CV*_*interolog*_) and DDI (*CV*_*DDI*_) (Equation 1). Since our interolog-based PPI prediction was derived from the evidence in plant templates, the *CV*_*interolog*_ was formulated to represent the confidence of the prediction, based upon the number of species from which the interaction was inferred. Additionally, this score was also weighted by the method employed to identify the interactions in the source organism, computational prediction or experimental measurements (Equation 2). The *CV*_*DDI*_ indicated the confidence of prediction based on domain-domain interactions, in which the number of interacting domains between a predicted protein pair was taken into account (Equation 3). The formulas were as follows:

1 $$CV=C{V}_{interolog}\times C{V}_{DDI}$$

2 $$C{V}_{interolog}=\frac{{\sum }_{i=1}^{N}{S}_{i}{M}_{i}}{N}$$

3 $$C{V}_{DDI}=dD$$

where *S* ∈ {0, 1} is the existence factor, indicating the presence of an orthologous protein pair in cassava genome. In this study, *S* is always equal to 1 because orthologous protein pairs need to be identified prior to being incorporated in the MePPI-In. *M* refers to the reliability of the method by which the interactions were identified: 0.5 for computational prediction, and 1 for experimental measurement. *N* ∈ {1, 2, …, 7} is the number of species from which the protein-protein interactions in cassava were inferred. *D* = [0, 1] refers to domain enrichment, which is the ratio of the reported DDI pairs to all interactions possibly happening among domains in a protein pair. For example, *D* calculated for three and four domain-containing proteins that interact via two DDIs is equal to 2/(3 × 4). To compensate the probability bias in calculation of *D* for the studied protein pairs that contain only one domain, the correction factor (*d*) = 0.5 was used. Otherwise, *d* was set to 1.

### Analysis of topology and functional content of MePPI-In

The MePPI-In network was characterized in terms of topology and biological function relevance (Fig. 1c). The topology of the network was analyzed using network analysis tools in Cytoscape^64^. The topological characteristics of MePPI-In were then compared with those of the cassava random network (simulated by Cytoscape containing the same number of nodes and average number of edges as MePPI-In), Arabidopsis^36^ and rice^9^ PPI networks. Biological function of the proteins in MePPI-In was examined through AgriGO^71^, from which GO enrichment analysis was determined using REVIGO^72^. The results were illustrated in scatterplot graph and compared with the GO enrichment of proteins in Arabidopsis^36^ and rice^9^ PPI networks.

## Results and Discussion

### Protein-protein interaction network of cassava

The interaction between proteins is a transient phenomenon that allows cells to be regulated at post-translational level. Since experimental investigation of PPIs is difficult and requires huge effort, prediction of protein interactions through computational techniques has, thus, widely been accepted^73,74^. In this study, interolog-based method was utilized to construct a genome-scale PPI network of cassava. Upon the homology-based principle of this method, seven plant species were selected as templates, based on one of the three criteria (the model plant, Arabidopsis, has abundant PPI information; potato, rice and maize are starch-storing crops; castor bean, poplar and soybean are closely related to cassava). According to PPI information from various databases (Fig. 1a), Arabidopsis has the most abundant PPI information (235,215 interactions of 17,962 proteins) followed by rice (76,829 interactions of 5,219 proteins), potato (42 interactions of 48 proteins), maize (25 interactions of 29 proteins), soybean (10 interactions of 12 proteins), castor bean (10 interactions of 10 proteins), and poplar (8 interactions of 10 proteins) (Table 1). To infer PPI information for cassava from each template plant, BLASTp search of the cassava genome sequence database was carried out. The cassava orthologous proteins that showed identity percentage ≥60, coverage percentage ≥80 and e-value ≤ 10^−10^ were identified. If these orthologous proteins matched the proteins of template plants that had previously been identified to have protein-protein interaction, such interactions were regarded as orthologous PPIs in cassava. Based on the results obtained, majority of the inferred PPIs were from Arabidopsis (90,069 interactions) followed by rice (212 interactions), potato (19 interactions), soybean (7 interactions), maize and poplar (5 interactions each) and castor bean (2 interactions) (Table 1).

**Table 1:** Protein-protein interactions in plant templates and MePPI-In.

Plants	Genome Information^60^		PPI Information			MePPI-In	
	Number of genes	Number of proteins	Number of PPIs	Number of proteins	Sources	Infered PPIs in cassava	Orthologs in casssava
Arabidopsis	27,416	35,386	235,215	17,962	ref.^36^ – ref.^39^	90,069	7,193
					ref.^62^ – ref.^63^		
Rice	55,986	154,310	76,829	5,219	ref.^9^, ref.^62^	212	84
Potato	35,119	59,699	42	48	ref.^62^	19	15
Maize	32,540	88,383	25	29	ref.^62^	5	8
Soybean	54,175	83,795	10	12	ref.^62^	7	7
Poplar	41,335	83,796	8	10	ref.^62^	5	7
Castorbean	25,878	31,576	10	10	ref.^62^	2	2
						90,173	7,209

The resulting interolog-based PPI network of cassava, or MePPI-In, is comprised of 90,173 interactions interconnecting 7,209 proteins, which accounted for *c.a*. 21 percent of proteins in the whole genome (Fig. S1). The overall predicted PPIs are available at http://bml.sbi.kmutt.ac.th/ppi. Figure 2 illustrates the overview of MePPI-In within which subnetwork demonstrated partial set of protein interaction, metabolism and sub-metabolism with specific group of proteins of interest. It is important to note here that different colors were given to both interactions (edges) and proteins (nodes) to indicate whether their existence could be supported by other evidences. Edge colors represented interactions from interolog-based method with or without co-expression or DDI support (black or grey), while node colors denoted the proteins with or without expression data (blue or orange) (see more details in the Supporting the interactions section below). Approximately 99 percent of the PPIs in MePPI-In were inferred from Arabidopsis and rice. None of the interactions included in our network was presented in all seven template plants. These results implied that availability of data was the main limitation in inference-based PPI network construction. To improve the confidence of the network derived originally from interolog-based prediction method, other available types of data, such as expression or domain-domain interaction, could be incorporated in the newly constructed MePPI-In.

### Supporting the interactions of proteins with expression data

Our MePPI-In was constructed using interolog-based method, which could only project the known PPIs in other plants to cassava. Accordingly, it might be helpful if collective information could be incorporated to support the occurrence of such predictions in cassava. In this study, expression of the proteins included in MePPI-In was examined using information from seven protein expression^50–53,56,57,59^ and four gene expression datasets^45,65–67^. Subsequently, co-expression of each interacting protein pair was also determined. This was based on the fact that interaction between two proteins occurs only if both proteins are presented at the same time.

**Figure 3:**
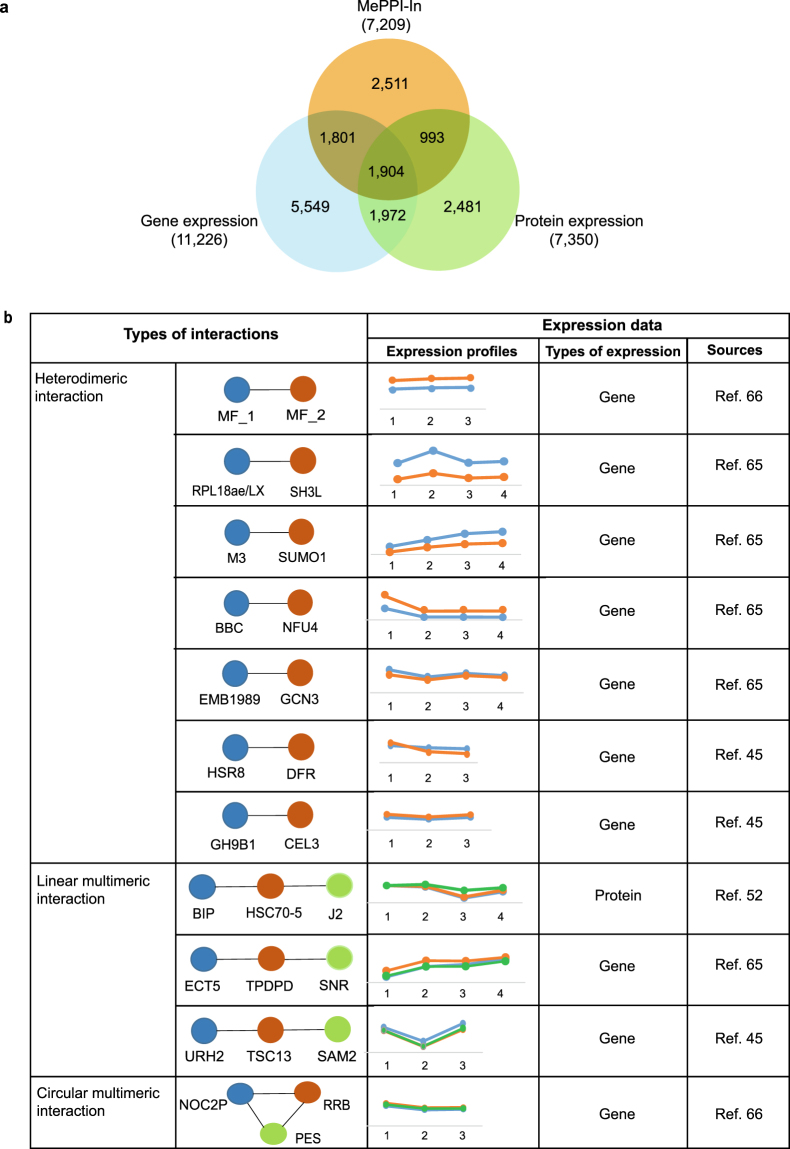
MePPI-In with supporting expression evidence. (**a**) Overview of the number of proteins in MePPI-In and expression information^45,50–53,56,57,59,65–67^. The numbers in parenthesis represented the total number of genes or proteins in each cohort. (**b**) The list of PPIs between proteins with co-expression profiles derived from time-series expression datasets of Yang *et al*.^45^ (cassava fibrous, intermediate and storage roots at 4 months), Li *et al*.^65^ (cassava leaves, stems and roots harvested at 2^nd^, 4^th^, 7^th^ and 10^th^ month), An *et al*.^66^ (cassava apical shoots subjected to cold at 7 °C for 0, 4 and 9 h) and Naconsie *et al*.^52^ (cassava storage roots harvested at 3^rd^, 6^th^, 9^th^ and 12^th^ months). The resulting interactions were classified into three groups based on the number of proteins and their topologies. Each protein was presented as a circle in the color that corresponds to the graph of its expression profile (see more information in Table S2).

From eleven expression datasets exploited here, 4,698 proteins expression were detected, from the total number of 7,209 proteins in MePPI-In, (Fig. 3a, Table S1). Accordingly, different colors were given to nodes (or proteins) shown in Fig. 2. The blue color nodes highlighted the proteins with supporting expression data, while the orange ones had no supporting expression data. Since expression of the proteins from eleven expression datasets indicated their presence in cassava, these proteins were then considered available for the interactions among them to occur. From the total of 90,173 interactions in MePPI-In, 35,146 interactions (or 39 percent) were observed to connect the proteins with supporting expression data.

Among the eleven expression datasets, the time-series datasets of Naconsie *et al*.^52^, Yang *et al*.^45^, Li *et al*.^65^ and An *et al*.^66^ were utilized to observe the correlation of expression between two proteins (Pearson correlation coefficient score (*PCC*) > 0.9 and *p*-value < 0.1). From the results obtained, there were 16 interactions that were identified to interconnect the proteins with co-expression pattern (Table S2). We further classified these PPIs into three groups based on the number of protein members and the types of interactions (Fig. 3b). First, the heterodimeric interactions represented interactions between two proteins, for example, an interaction between methionine adenosyltransferase 3 (M3) and small ubiquitin-like modifier 1 (SUMO1). The second group represented linear multimeric interactions, which probably exhibited the sequential functional relationship between proteins. The third was circular multimeric interactions. The interactions between nucleolar complex protein 2 (NOC2P), ribosomal RNA processing brix domain protein (RRB) and pescadillo-like protein (PES) potentially suggested functional relationship of these proteins in rRNA processing and cell proliferation control^75^.

### Supporting the interaction of proteins with DDI data

From our MePPI-In, reliability of each PPI was determined by incorporating the information on domain-domain interaction (DDI). This was based upon the observation that the proteins usually interact via specific domains. In this work, the domain information of proteins in MePPI-In were obtained from Pfam database^69^ and the interactions between protein domains were from iPfam database^70^ (Fig. 1b). From the total number of proteins proposed in MePPI-In, only 4,963 proteins (or 69 percent) were identified to have at least one domain. From these numbers, only 1,981 proteins (or 27 percent of the proteins proposed in MePPI-In or 40 percent of the proteins with domain information) were indicated, by iPfam, to interact via specific domains. Accordingly, only 6,826 from 90,173 interactions (~seven percent) could be confirmed through DDI information. Even with Pfam and iPfam, the largest universal repositories of protein domain information, only a small fraction of interactions initially proposed in MePPI-In were identified to have supporting DDI information. However, this did not mean that the DDI information could not provide any insight into the confidence level of PPIs obtained from interolog-based prediction method.

### Ranking the confidence of PPIs in MePPI-In based on DDI data

In this study, the confidence of the predicted PPIs in MePPI-In was classified into two groups. The PPIs with high confidence level (High (H), Table 2) represented those with supporting domain information (6,826 PPIs). The other class which exhibited basal confidence level (Basal (B), Table 2) included the remaining interactions in MePPI-In (83,347 PPIs). Emphases on the class of high confidence level, confidence value (*CV*) scores, for the 6,826 PPIs, were determined and classified into three sub-groups, based on the percentile rank of the *CV* scores (see Methods). The *CV* score basically ranges from 0 to 1, from the lowest to the highest level of confidence. However, the distribution of *CV* scores was observed to be positively skewed, meaning that majority of the PPIs had the *CV* score ≤ 0.5. This was because most of the PPIs in MePPI-In were obtained from computational prediction, not experimental measurements. Accordingly, the factor *M* for the calculation of *CV*_*interolog*_ was set at 0.5. Also, in most cases, the correction factor *d* for the calculation of *CV*_*DDI*_ was set at 0.5 to represent the DDIs between proteins with only one domain. The small values of both *CV*_*interolog*_ and *CV*_*DDI*_ only allowed a maximum final *CV* scores of 0.5. Accordingly, percentile calculation was employed to further classify these high confidence PPIs into three sub-classes: H1 (percentile of *CV* score > 80), H2 (percentile of *CV* score: 50–80) and H3 (percentile of *CV* score < 50) (Table 2).

**Table 2:** Classification of predicted PPIs based on the confidence level.

	Confidence level	Range of confidence value (*CV*)	Number	
			PPIs	Proteins
Total MePPI-In	—	—	90,173	7,209
Interolog, no DDI information	Basal (B)	—	83,347	5,228
Interolog, DDI - heterodimer	High (H)	[0.00, 1.00]	6,826	1,981
Percentile of *CV* score: >80	H1	[0.14, 1.00]	1,184	733
Percentile of *CV* score: 50–80	H2	[0.12, 0.14)	3,859	1,439
Percentile of *CV* score: <50	H3	[0.01, 0.12)	1,783	855

As mentioned above, the current knowledge of protein domain and DDI information can support only up to seven percent of the overall interactions in MePPI-In. However, the confidence level obtained, along with the PPIs, herein, should help contrast the reliability of each prediction for further investigation by experimental approaches.

### Characteristics of the MePPI-In Network

The MePPI-In proposed in this study is the first genome-wide protein-protein interaction network of cassava consisting of 90,173 interactions and 7,209 proteins. Here, characteristics of the MePPI-In were described in terms of the global network topology and the functional coverage. At last, we discussed how MePPI-In might be used to infer biological regulatory processes. Some of these applications include (1) identification of a hub protein in the interactome cascade, (2) functional identification of unknown protein, (3) inference of protein complex formation, and (4) study of protein connections in metabolic pathway as well as connection of various metabolic pathways via protein-protein interactions. These examples showed the contribution of MePPI-In in envisaging cellular communication via crosstalk at protein level.

### MePPI-In performs as a biological network

A protein-protein interaction network, such as MePPI-In, is generally constructed from available proteome and interactome data of reference species and the studied organism itself. The MePPI-In proposed here included knowledge of PPIs from both cassava and other plants. The constructed network contained a large number of possible interacting protein pairs; nonetheless, it was impossible to determine the exact coverage of the network constituents of the overall PPIs that exist in real living cells. The ill-defined network boundary did not allow assessment of its representativeness in the cellular PPI matrix. In this circumstance, network topology was employed, at least as an alternative, to suggest the plausibility of the proposed network as if it possesses the properties of common biological network system^76^. To investigate topology of MePPI-In, the key global network properties, including node degree distribution, average path length (*L*) and clustering coefficient (*C*_*i*_), were determined according to graphical analysis method^76^.

**Figure 4:**
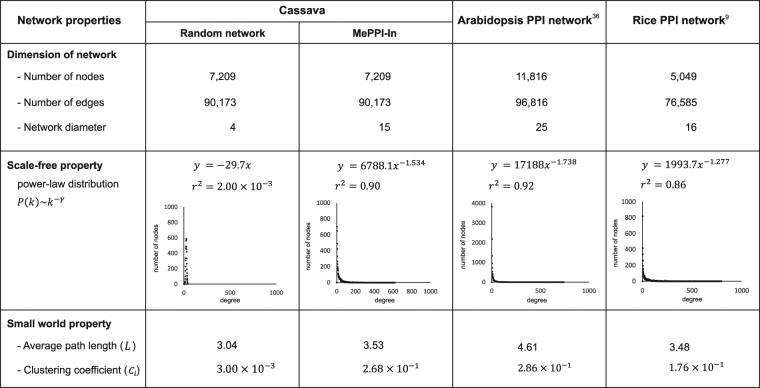
The global network properties of protein-protein interaction networks in cassava, Arabidopsis and rice.

The MePPI-In exhibited the biological network characteristics based upon the two supporting properties; scale-free and small world. First, the connectivity (*k*) of the proteins in MePPI-In followed a power-law distribution, *P*(*k*)~*k* ^−*γ*^. The MePPI-In exhibited scale-free property and showed an explicit deviation from a random network, in which most proteins had relatively the same numbers of interactions as shown in the relationship between degree and number of nodes (Fig. 4). In MePPI-In, most proteins have only a few interactions and only a few proteins, called hub proteins, have a large number of interactions. The scale-free property is not only observed in MePPI-In, but also occurs in other types of biological networks^76^, such as metabolic network, and gene regulatory network^77^. MePPI-In was analyzed and compared with the PPI networks of Arabidopsis^36^ and rice^9^. Figure 4 described the various features of these PPI networks, including network diameter, average path length and clustering coefficient. Although these PPI networks contained different numbers of interactions and proteins, all of them followed a power law distribution, a common behavior of biological networks in living organisms.

Second, the MePPI-In possessed a small-world structure. According to the definition given by Watz and Strogatz^78^, the average path length of a small-world network must be relatively greater than average path length (*L*) of random network, while the clustering coefficient (*C*_*i*_) of small world network is relatively much higher than *C*_*i*_ of random network (*L*_*small world*_ ≥ *L*_*random*_ but *C*_*i small world*_ ≫ *C*_*i random*_). The average path length (average of shortest paths between all possible pairs of proteins in the network) value of MePPI-In was 3.53, slightly greater than that of the random network with the same number of nodes and average number of edges per node (3.04). Moreover, the much higher clustering coeffiecient of MePPI-In, when comparing with random network shown in Fig. 4, also supported the small world property of our network. This property of MePPI-In is considered beneficial, since it can protect cassava from any perturbation caused by endogenous and exogenous stimuli.

In conclusion, based on the two network properties mentioned above, MePPI-In exhibits a well-presented biological network behavior. It is thus presumed that the MePPI-In contains sufficient information, hence, could be utilized as the model PPI network in cassava.

### Functional content of MePPI-In

**Figure 5:**
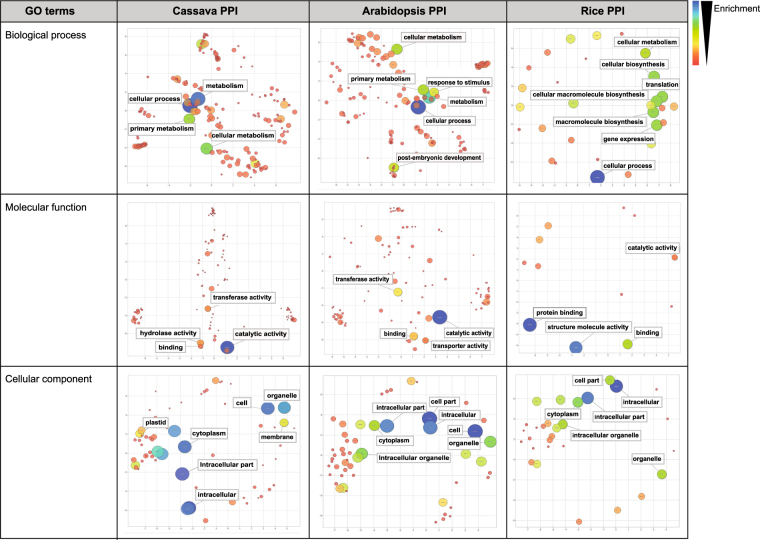
Comparison of the functional content of MePPI-In and the PPI networks of Arabidopsis and rice. The scatterplots were derived based on GO enrichment analysis. The node color showed degree of enrichment ranging from low (red) to high (blue), while the node size represented the frequency of the proteins in each functional group.

Besides the network topology, functional content of proteins in MePPI-In was determined to demonstrate some PPI-based regulation in cassava. The functional coverage of proteins in the network was examined by GO analysis. The results were presented based on three classes of gene ontology^79^, biological process, molecular function and cellular component. In addition to the basic GO terms, GO enrichment of proteins in MePPI-In was examined and the results were presented in scatterplot (Fig. 5). The node color showed degree of enrichment ranging from low (red) to high (blue), while the node size represented the frequency of proteins in each functional group. According to the scatterplot of MePPI-In, a large number of proteins, in ‘biological process’ class, were identified to be related to cellular and metabolic processes. These corresponded to the result in the ‘molecular function’ class, in which a large number of the proteins were observed to have catalytic activity or binding capacity, probably acting as enzymes in metabolic pathways and transcription factors in cellular regulatory processes. For the last class of gene ontology or the ‘cellular component’, our results provided no information on specific compartments of the cell where the PPIs tend to occur.

By comparing MePPI-In with the Arabidopsis and rice PPI networks, some similarities of the functional content of the proteins were observed (Fig. 5). These included the proteins that function in cellular processes and cellular metabolism, and possess catalytic or binding capacity. The functional content of MePPI-In was observed to be more closely related to Arabidopsis than to rice. These differences probably reflect the different nature of the plants as cassava and Arabidopsis are dicots, while rice is a monocot^80,81^.

### Inference of biological regulation from MePPI-In

The involvement of protein-protein interaction in mediating cellular regulation has been reported in several studies^82–84^. These studies demonstrated key roles of PPIs in post-translational regulation that governs biological processes in cells. In similar manner, we propose possible post-translational regulation in cassava using the information acquired from MePPI-In. Genome-scale network enabled us to access the extensive cooperation of PPIs underlying specific cellular regulatory process, beyond the explanation at an associative protein pair.

**Figure 6:**
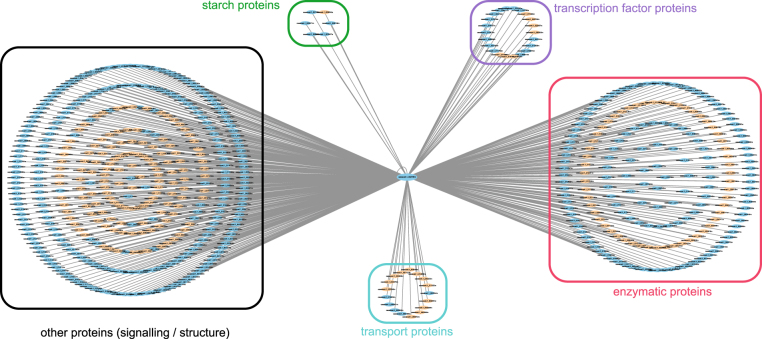
Interaction of the cassava heat shock protein 90.1 (HSP90.1; cassava4.1_002708m) and its partner proteins in MePPI-In. The nodes represent the proteins; blue for proteins with supporting expression data^45,50–53,56,57,59,65–67^ and orange for proteins with no supporting data. The edges represent interactions between HSP90.1 and its partners.

As the first global protein interaction network in cassava, our MePPI-In was able to illustrate the complexity of cellular regulation in cassava, from the highly elaborate topology of the network (Fig. 2). Moreover, our cassava PPI network (MePPI-In) has brought various insights. First, it helps in identifying the most important protein whose significance is reflected by its number of interactions with diverse partner proteins (denoted as high node degree in Fig. 2). Regarding the MePPI-In, heat shock protein 90.1 (HSP90.1; cassava4.1_002708m) showed the highest (620) number of connections (Fig. 6). HSP90.1 was recognized as a communication hub because it interacts with various types of proteins including transcription factors, signaling proteins, structural proteins and enzymatic proteins (Fig. 6). HSP90.1 was reported to play roles in various biological processes, including protein folding, intracellular transport, protein degradation and cell signaling^85,86^, which agrees with our finding. Similar to cassava, Arabidopsis and rice also use heat shock protein as the center of communication, but the hub protein in these two plants was heat shock protein 70 (HSP70)^33,36^. In MePPI-In, the HSP70 is one of the proteins with many connections (following only HPS90.1). However, partner proteins that interact with cassava HSP70 are different from those that interact with Arabidopsis and rice HSP70. These results suggested that cassava, Arabidopsis and rice may use these core proteins to respond to stress, but how these plants react are different since they use different mechanisms through different protein activities.

**Figure 7:**
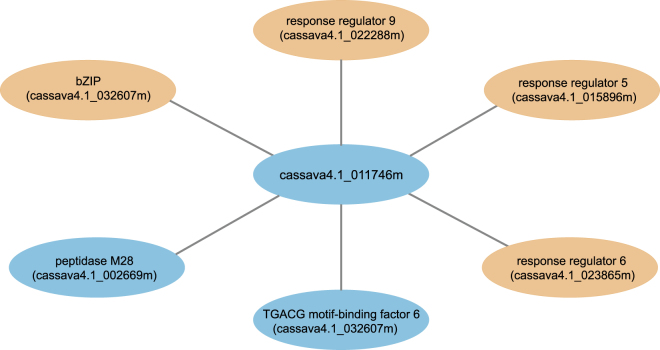
Interaction of an unknown protein (cassava4.1_011746m) with partner proteins with known function. The nodes represent the proteins; blue for proteins with supporting expression data^45,50–53,56,57,59,65–67^ and orange for proteins with no supporting data. The edges represent interactions between the unknown protein and its partners.

Second, the MePPI-In might be used to identify functions of unknown proteins in similar manner to Sharan *et al*.^87^. Proteins involved in same metabolic pathway usually interact to carry out a specific task required by cells. From MePPI-In, the unknown protein, cassava4.1_011746m, was observed to interact with five proteins; cassava4.1_032607m (basic leucine zipper transcription factor protein (bZIP)), cassava4.1_007074m (TGACG motif-binding factor 6), cassava4.1_015896m (response regulator 5), cassava4.1_023865m (response regulator 6), and cassava4.1_022288m (response regulator 9), all of which are transcription factors^88,89^ (Fig. 7). Based on its interaction with transcription factors, the unknown protein might act as another component in this transcriptional regulation cascade.

**Figure 8:**
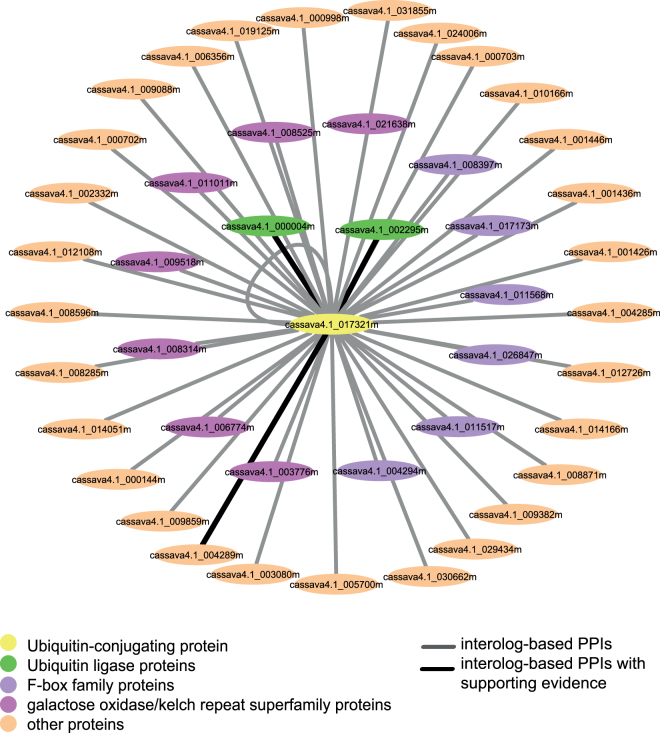
Protein complex of the cassava ubiquitin-conjugating protein (cassava4.1_017321m). The nodes represent the proteins; ubiquitin-conjugating protein (yellow); ubiquitin ligases (green); F-box family proteins (purple); galactose oxidase/kelch repeat superfamily proteins (pink); other proteins (orange). The edges represent PPIs: black – PPIs with DDI or co-expression support, and grey for – PPIs with no supporting data.

Third, the network could be used to investigate the possibility of proteins to form complexes*, as in vivo* proteins often work together by forming protein complex. From MePPI-In, interaction between ubiquitin-conjugating protein (E2; cassava4.1_017321m) and ubiquitin ligase proteins (E3; cassava4.1_000004m and cassava4.1_002295m) was observed with high interaction confidence (Fig. 8). This finding agreed well with the results from previous study which reported that during ubiquitination process, ubiquitin-conjugating proteins form complexes with ubiquitin ligase proteins prior to binding to target proteins^90^. In addition to the proteins that are known to form complexes, our network also indicated additional protein components that might form complex with the ubiquitin-conjugating protein (Fig. 8). These included the F-box family proteins, which were reported to mediate ubiquitination during protein degradation^91^, and galactose oxidase/kelch repeat superfamily proteins, which functions as substrate-specific adapter proteins in ubiquitin ligase binding^92^.

**Figure 9:**
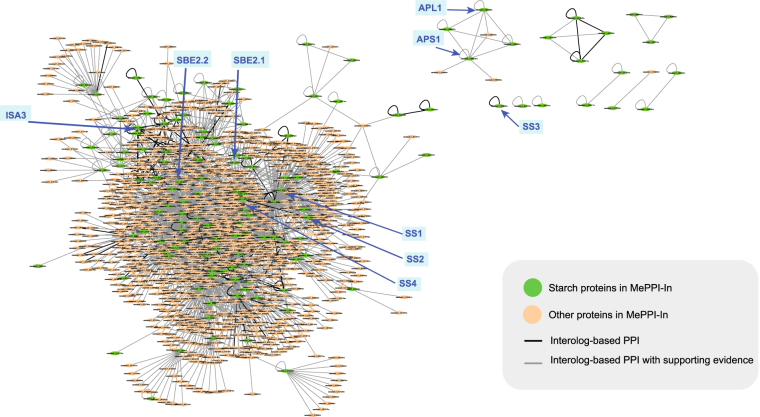
Cassava starch sub-network. The nodes represent starch proteins (green) and their first neighbors (orange). The edges represent interactions between proteins; black for PPIs with DDI or co-expression supporting data, and grey for PPIs with no supporting information. Within the starch sub-network, the arrows indicated where the starch synthases (SS1, SS2, SS3, SS4), starch branching enzymes (SBE2.1, SBE2.2), isoamylase (ISA3) and ADP glucose pyrophosphorylase in large and small subunit (APL1, APS1) resided.

Fourth, the network could be used to gain knowledge on a particular metabolic pathway. As the value of cassava mainly relies on its capacity to synthesize and store starch, our MePPI-In might provide some insight related to the biosynthesis of starch in cassava. Herein, all proteins involved in the CO_2_ fixation pathway (Calvin cycle), sucrose biosynthesis pathway and starch biosynthesis pathway (defined as starch proteins)^47^ as well as their partners, were presented in the form of starch sub-network (Fig. 9). According to Fig. 9, starch proteins interact not only with starch proteins, but with other proteins such as signaling proteins, regulatory proteins, and proteins in other metabolic pathways. These results suggested that starch metabolism was tightly regulated. Since starch proteins connected to proteins in other metabolic processes, its perturbations could eventually affect whole organism. This might be the reason why unexpected pleiotropic effects were often observed, even though the mutants in question had already been proven to lack only a single starch gene.

## Conclusions

The study of protein-protein interaction allows us to envisage potential post-translational regulation that mediates the cellular processes in cassava. Our MePPI-In is the first genome-scale protein-protein interaction network of cassava, consisting of 90,173 interactions and 7,209 proteins. The MePPI-In was constructed from extensive PPI data of seven plants (*i.e*. Arabidopsis, rice, potato, maize, castor bean, soybean, and poplar) using interolog-based method. The MePPI-In contained the largest number of PPIs in cassava, which are involved in many biological processes especially cellular process, and metabolism. Moreover, confidence value (*CV*) was calculated to rank the reliability of the prediction, which is beneficial for the discovery of promising PPI for further investigation. The biological insights gained from the MePPI-In network, hopefully, fill a part of the current gap of knowledge on cassava proteins and their function.

## Electronic supplementary material

Supplement Figure S1

Supplement Table S1

Supplement Table S2
