# Supplementary material for: Prediction of cassava protein interactome based on interolog method
Source: Sci Rep. 2017 Dec 8;7:17206. doi: 10.1038/s41598-017-17633-2 (PMC5722940; doi:10.1038/s41598-017-17633-2)
Supplement: Supplementary file 1 — Supplement Figure S1 [file 41598_2017_17633_MOESM1_ESM.pdf]

Prediction of cassava protein interactome based on interolog method

Ratana Thanasomboon,

Saowalak Kalapanulak,

Supatcharee Netrphand,

Treenut Saithong*

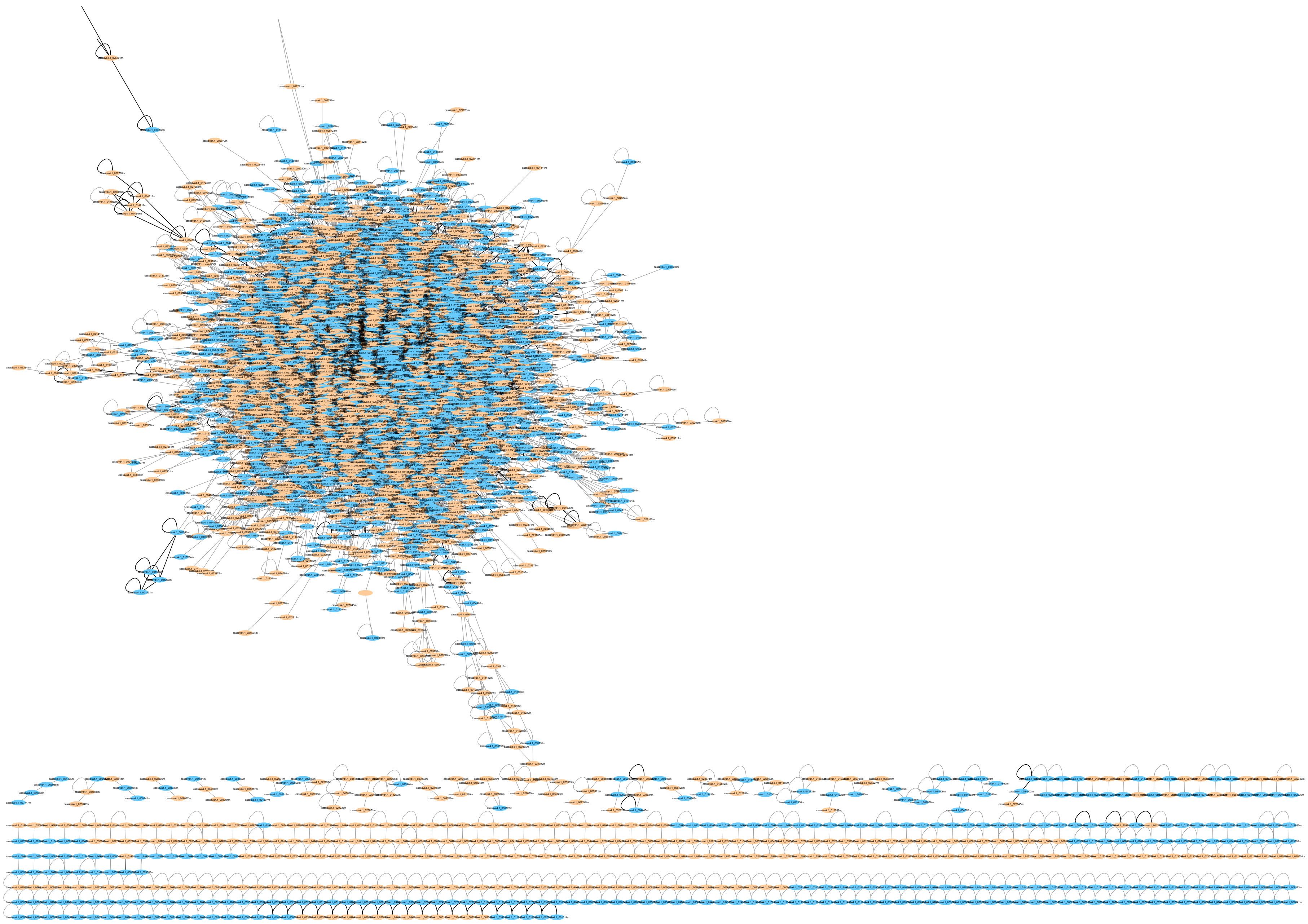

Figure S1 The constructed interolog-based protein-protein interaction network of cassava (MePPI-In). The network contained 90,173 interactions (edges) interconnecting 7,209 proteins (nodes). Different colors were given to both edges and nodes. The black edges represent PPIs with DDI or co-expression support while the grey ones represent those with no supportingdata. Blue color nodes represent proteins with supporting expression data[45,50-53,56-57,59,65-67], while the orange ones have no expression support.
