# Supplementary material for: Prediction of cassava protein interactome based on interolog method
Source: Sci Rep. 2017 Dec 8;7:17206. doi: 10.1038/s41598-017-17633-2 (PMC5722940; doi:10.1038/s41598-017-17633-2)
Supplement: Supplementary file 2 — Supplement Table S1 [file 41598_2017_17633_MOESM2_ESM.pdf]

Prediction of cassava protein interactome based on interolog method

Ratana Thanasomboon,

Saowalak Kalapanulak,

Supatcharee Netrphand,

Treenut Saithong*

Name (UniProt&Phytozome)[a]	Phytozome protein ID	Description	Expression[b]
cassava4.1_000004m|PACid:17967576	cassava4.1_000004m	ubiquitin-protein ligase 1	G
cassava4.1_000005m|PACid:17979331	cassava4.1_000005m	Phosphatidylinositol 3- and 4-kinase family protein with FAT domain	GP
cassava4.1_000008m|PACid:17981670	cassava4.1_000008m	Beige/BEACH domain ;WD domain, G-beta repeat protein	GP
cassava4.1_000009m|PACid:17961928	cassava4.1_000009m	P-loop containing nucleoside triphosphate hydrolases superfamily protein	GP
cassava4.1_000010m|PACid:17976258	cassava4.1_000010m	WD-40 repeat family protein / beige-related	No
cassava4.1_000019m|PACid:17965651	cassava4.1_000019m	ILITYHIA	P
cassava4.1_000025m|PACid:17985153	cassava4.1_000025m	transcription regulators	GP
cassava4.1_000030m|PACid:17983326	cassava4.1_000030m	Pre-mRNA-processing-splicing factor	P
cassava4.1_000033m|PACid:17971789	cassava4.1_000033m	acetyl-CoA carboxylase 1	GP
cassava4.1_000037m|PACid:17978488	cassava4.1_000037m	transcription regulatory protein SNF2, putative	G
cassava4.1_000039m|PACid:17989931	cassava4.1_000039m	NADH-dependent glutamate synthase 1	No
cassava4.1_000045m|PACid:17972406	cassava4.1_000045m	calpain-type cysteine protease family	G
cassava4.1_000047m|PACid:17989603	cassava4.1_000047m	ARM repeat superfamily protein	GP
cassava4.1_000048m|PACid:17988751	cassava4.1_000048m	binding	No
cassava4.1_000053m|PACid:17991397	cassava4.1_000053m	Armadillo/beta-catenin-like repeat ; C2 calcium/lipid-binding domain (CaLB) protein	G
cassava4.1_000063m|PACid:17962336	cassava4.1_000063m	ARM repeat superfamily protein	No
cassava4.1_000064m|PACid:17982815	cassava4.1_000064m	Phosphatidylinositol 3- and 4-kinase family protein	GP
cassava4.1_000068m|PACid:17975569	cassava4.1_000068m	dicer-like 1	G
cassava4.1_000069m|PACid:17966938	cassava4.1_000069m	Ataxia telangiectasia-mutated and RAD3-related	No
cassava4.1_000076m|PACid:17969257	cassava4.1_000076m	DNA binding;ATP binding;nucleic acid binding;binding;helicases;ATP binding	GP
cassava4.1_000079m|PACid:17965967	cassava4.1_000079m	recovery protein 3	No
cassava4.1_000080m|PACid:17977504	cassava4.1_000080m	HEAT repeat ;HECT-domain (ubiquitin-transferase)	GP
cassava4.1_000082m|PACid:17973406	cassava4.1_000082m	glucan synthase-like 4	No
cassava4.1_000089m|PACid:17980594	cassava4.1_000089m	guanyl-nucleotide exchange factors;GTPase binding;GTP binding	No
cassava4.1_000090m|PACid:17970186	cassava4.1_000090m	phosphatidylinositol-4-phosphate 5-kinase family protein	No
cassava4.1_000091m|PACid:17979196	cassava4.1_000091m	RNA polymerase II large subunit	GP
cassava4.1_000092m|PACid:17966117	cassava4.1_000092m	RNA polymerase II transcription mediators	GP
cassava4.1_000103m|PACid:17987979	cassava4.1_000103m	HOPM interactor 7	P
cassava4.1_000109m|PACid:17963290	cassava4.1_000109m	SEC7-like guanine nucleotide exchange family protein	GP
cassava4.1_000112m|PACid:17969988	cassava4.1_000112m	glucan synthase-like 1	GP
cassava4.1_000113m|PACid:17983579	cassava4.1_000113m	PHD finger family protein	No
cassava4.1_000117m|PACid:17981789	cassava4.1_000117m	chromatin remodeling 5	No
cassava4.1_000121m|PACid:17984592	cassava4.1_000121m	SEC7-like guanine nucleotide exchange family protein	GP
cassava4.1_000122m|PACid:17977763	cassava4.1_000122m	histone acetyltransferase of the CBP family 12	No
cassava4.1_000125m|PACid:17965129	cassava4.1_000125m	THO2	No
cassava4.1_000128m|PACid:17962067	cassava4.1_000128m	WD40/YVTN repeat-like-containing domain;Bromodomain	G
cassava4.1_000133m|PACid:17972973	cassava4.1_000133m	Clathrin, heavy chain	GP
cassava4.1_000135m|PACid:17975604	cassava4.1_000135m	Tetratricopeptide repeat (TPR)-like superfamily protein	P

cassava4.1_000143m|PACid:17990384	cassava4.1_000143m	binding	No
cassava4.1_000144m|PACid:17972049	cassava4.1_000144m	SNF2 domain-containing protein / helicase domain-containing protein	No
cassava4.1_000147m|PACid:17961992	cassava4.1_000147m	protein serine/threonine kinases;protein tyrosine kinases;ATP binding;protein kinases	No
cassava4.1_000150m|PACid:17983757	cassava4.1_000150m	UDP-glucose:glycoprotein glucosyltransferases	GP
cassava4.1_000160m|PACid:17992955	cassava4.1_000160m	Transducin/WD40 repeat-like superfamily protein	GP
cassava4.1_000163m|PACid:17973209	cassava4.1_000163m	protein kinases;ubiquitin-protein ligases	GP
cassava4.1_000166m|PACid:17985051	cassava4.1_000166m		No
cassava4.1_000170m|PACid:17969289	cassava4.1_000170m	global transcription factor group B1	No
cassava4.1_000182m|PACid:17968362	cassava4.1_000182m	multidrug resistance-associated protein 5	G
cassava4.1_000183m|PACid:17980565	cassava4.1_000183m	methyltransferase 1	No
cassava4.1_000187m|PACid:17985971	cassava4.1_000187m	protein kinase family protein / WD-40 repeat family protein	No
cassava4.1_000188m|PACid:17992942	cassava4.1_000188m	DNA-directed DNA polymerases	No
cassava4.1_000190m|PACid:17982605	cassava4.1_000190m	nuclear RNA polymerase A1	GP
cassava4.1_000191m|PACid:17982148	cassava4.1_000191m	Myosin family protein with Dil domain	P
cassava4.1_000195m|PACid:17973268	cassava4.1_000195m	P-loop containing nucleoside triphosphate hydrolases superfamily protein	GP
cassava4.1_000196m|PACid:17982094	cassava4.1_000196m	INO80 ortholog	No
cassava4.1_000199m|PACid:17969290	cassava4.1_000199m	global transcription factor group B1	G
cassava4.1_000202m|PACid:17971719	cassava4.1_000202m	myosin 2	GP
cassava4.1_000205m|PACid:17988975	cassava4.1_000205m	multidrug resistance-associated protein 4	No
cassava4.1_000208m|PACid:17984094	cassava4.1_000208m	ABC-2 and Plant PDR ABC-type transporter family protein	GP
cassava4.1_000209m|PACid:17988934	cassava4.1_000209m	multidrug resistance-associated protein 9	No
cassava4.1_000210m|PACid:17965900	cassava4.1_000210m	P-loop containing nucleoside triphosphate hydrolases superfamily protein	No
cassava4.1_000211m|PACid:17965135	cassava4.1_000211m	nucleoporin 155	P
cassava4.1_000214m|PACid:17974404	cassava4.1_000214m	glutamate synthase 1	GP
cassava4.1_000215m|PACid:17975111	cassava4.1_000215m	multidrug resistance-associated protein 3	G
cassava4.1_000219m|PACid:17960822	cassava4.1_000219m	multidrug resistance-associated protein 14	G
cassava4.1_000220m|PACid:17973312	cassava4.1_000220m	multidrug resistance-associated protein 6	G
cassava4.1_000224m|PACid:17959881	cassava4.1_000224m	chromatin remodeling factor CHD3 (PICKLE)	No
cassava4.1_000226m|PACid:17980007	cassava4.1_000226m	sec7 domain-containing protein	No
cassava4.1_000228m|PACid:17964285	cassava4.1_000228m	SCAR homolog 2	No
cassava4.1_000236m|PACid:17964597	cassava4.1_000236m	cleavage and polyadenylation specificity factor 160	No
cassava4.1_000238m|PACid:17972440	cassava4.1_000238m	pleiotropic drug resistance 11	GP
cassava4.1_000239m|PACid:17971690	cassava4.1_000239m	CLIP-associated protein	GP
cassava4.1_000246m|PACid:17988450	cassava4.1_000246m	SIN3-like 4	No
cassava4.1_000255m|PACid:17975316	cassava4.1_000255m	pleiotropic drug resistance 12	GP
cassava4.1_000256m|PACid:17981860	cassava4.1_000256m	ARM repeat superfamily protein	GP
cassava4.1_000258m|PACid:17986742	cassava4.1_000258m	tetratricopeptide repeat (TPR)-containing protein	GP
cassava4.1_000260m|PACid:17975364	cassava4.1_000260m	pleiotropic drug resistance 4	No
cassava4.1_000262m|PACid:17978828	cassava4.1_000262m	sec7 domain-containing protein	No

cassava4.1_000264m|PACid:17960675	cassava4.1_000264m	purine biosynthesis 4	GP
cassava4.1_000265m|PACid:17993596	cassava4.1_000265m	SIN3-like 4	No
cassava4.1_000277m|PACid:17969181	cassava4.1_000277m	magnesium ion binding;thiamin pyrophosphate binding;hydro-lyases	No
cassava4.1_000280m|PACid:17980200	cassava4.1_000280m	magnesium-chelatase subunit chlH, chloroplast, putative	GP
cassava4.1_000283m|PACid:17981711	cassava4.1_000283m	eukaryotic translation initiation factor 2 (eIF-2) family protein	GP
cassava4.1_000287m|PACid:17971287	cassava4.1_000287m	xanthine dehydrogenase 1	GP
cassava4.1_000300m|PACid:17979719	cassava4.1_000300m	P-loop containing nucleoside triphosphate hydrolases superfamily protein	G
cassava4.1_000301m|PACid:17969654	cassava4.1_000301m	nuclear RNA polymerase C1	No
cassava4.1_000302m|PACid:17993066	cassava4.1_000302m	HEAT repeat ;WD domain, G-beta repeat protein protein	GP
cassava4.1_000304m|PACid:17962169	cassava4.1_000304m	Protein kinase family protein with ARM repeat domain	No
cassava4.1_000306m|PACid:17968622	cassava4.1_000306m	ATP binding cassette subfamily B1	No
cassava4.1_000308m|PACid:17963456	cassava4.1_000308m	binding	No
cassava4.1_000310m|PACid:17979666	cassava4.1_000310m	binding	No
cassava4.1_000311m|PACid:17976622	cassava4.1_000311m	peroxisomal ABC transporter 1	GP
cassava4.1_000328m|PACid:17988926	cassava4.1_000328m	IKI3 family protein	GP
cassava4.1_000330m|PACid:17986510	cassava4.1_000330m	abscisic aldehyde oxidase 3	GP
cassava4.1_000332m|PACid:17961855	cassava4.1_000332m	REF4-related 1	No
cassava4.1_000342m|PACid:17969737	cassava4.1_000342m	callose synthase 1	GP
cassava4.1_000344m|PACid:17978037	cassava4.1_000344m	Protein kinase superfamily protein	GP
cassava4.1_000345m|PACid:17963748	cassava4.1_000345m	P-glycoprotein 11	P
cassava4.1_000352m|PACid:17981558	cassava4.1_000352m	AAA-type ATPase family protein	G
cassava4.1_000356m|PACid:17969267	cassava4.1_000356m	Protein kinase family protein with ARM repeat domain	No
cassava4.1_000358m|PACid:17981545	cassava4.1_000358m	translocon at the outer envelope membrane of chloroplasts 159	GP
cassava4.1_000361m|PACid:17994102	cassava4.1_000361m	Cleavage and polyadenylation specificity factor (CPSF) A subunit protein	No
cassava4.1_000363m|PACid:17991289	cassava4.1_000363m	sister chromatid cohesion 1 protein 4	No
cassava4.1_000364m|PACid:17977397	cassava4.1_000364m	DNA polymerase V family	GP
cassava4.1_000365m|PACid:17975422	cassava4.1_000365m	Tetratricopeptide repeat (TPR)-like superfamily protein	GP
cassava4.1_000371m|PACid:17988605	cassava4.1_000371m	related to KPC1	No
cassava4.1_000373m|PACid:17979749	cassava4.1_000373m	RNA helicase, putative	GP
cassava4.1_000375m|PACid:17982071	cassava4.1_000375m	oxoprolinase 1	No
cassava4.1_000376m|PACid:17960716	cassava4.1_000376m	target of rapamycin	GP
cassava4.1_000377m|PACid:17974173	cassava4.1_000377m	zinc ion binding	No
cassava4.1_000380m|PACid:17989128	cassava4.1_000380m	ARM repeat superfamily protein	GP
cassava4.1_000381m|PACid:17989793	cassava4.1_000381m	splicing factor, putative	P
cassava4.1_000382m|PACid:17981193	cassava4.1_000382m	phragmoplast-associated kinesin-related protein, putative	G
cassava4.1_000384m|PACid:17984309	cassava4.1_000384m	ABC transporter family protein	No
cassava4.1_000385m|PACid:17975374	cassava4.1_000385m	ATP binding cassette subfamily B19	No
cassava4.1_000388m|PACid:17979536	cassava4.1_000388m	histidine kinase 1	No
cassava4.1_000389m|PACid:17968165	cassava4.1_000389m	multimeric translocon complex in the outer envelope membrane 132	GP

cassava4.1_000395m|PACid:17987699	cassava4.1_000395m	histidine kinase 2	No
cassava4.1_000396m|PACid:17976571	cassava4.1_000396m	zinc finger protein-related	No
cassava4.1_000397m|PACid:17966345	cassava4.1_000397m	histidine kinase 1	G
cassava4.1_000398m|PACid:17960517	cassava4.1_000398m	P-glycoprotein 13	No
cassava4.1_000399m|PACid:17985450	cassava4.1_000399m	P-glycoprotein 2	GP
cassava4.1_000402m|PACid:17990097	cassava4.1_000402m	Patched family protein	No
cassava4.1_000408m|PACid:17979220	cassava4.1_000408m	kinesin-like calmodulin-binding protein (ZWICHEL)	G
cassava4.1_000424m|PACid:17970289	cassava4.1_000424m	P-glycoprotein 20	GP
cassava4.1_000425m|PACid:17973095	cassava4.1_000425m	ATPase E1-E2 type family protein / haloacid dehalogenase-like hydrolase family protein	G
cassava4.1_000430m|PACid:17966683	cassava4.1_000430m	BRI1 like	No
cassava4.1_000431m|PACid:17992205	cassava4.1_000431m	K+ efflux antiporter 2	GP
cassava4.1_000433m|PACid:17982277	cassava4.1_000433m	Structural maintenance of chromosomes (SMC) family protein	GP
cassava4.1_000435m|PACid:17984973	cassava4.1_000435m	Coatomer, alpha subunit	No
cassava4.1_000437m|PACid:17964883	cassava4.1_000437m	cullin-associated and neddylation dissociated	GP
cassava4.1_000445m|PACid:17968363	cassava4.1_000445m	chromatin remodeling 8	No
cassava4.1_000446m|PACid:17959974	cassava4.1_000446m	RNA binding;RNA binding	GP
cassava4.1_000448m|PACid:17987248	cassava4.1_000448m	myosin 2	GP
cassava4.1_000449m|PACid:17979372	cassava4.1_000449m	DEA(D/H)-box RNA helicase family protein	G
cassava4.1_000457m|PACid:17973769	cassava4.1_000457m	binding;RNA binding	GP
cassava4.1_000458m|PACid:17964687	cassava4.1_000458m	Carbohydrate-binding-like fold	P
cassava4.1_000462m|PACid:17985870	cassava4.1_000462m	RNA-dependent RNA polymerase 6	G
cassava4.1_000466m|PACid:17959806	cassava4.1_000466m	nuclear RNA polymerase D2A	No
cassava4.1_000470m|PACid:17970626	cassava4.1_000470m	Leucine-rich receptor-like protein kinase family protein	No
cassava4.1_000474m|PACid:17969060	cassava4.1_000474m	ATPase E1-E2 type family protein / haloacid dehalogenase-like hydrolase family protein	G
cassava4.1_000475m|PACid:17978726	cassava4.1_000475m	DNA-directed RNA polymerase family protein	GP
cassava4.1_000476m|PACid:17992530	cassava4.1_000476m		No
cassava4.1_000479m|PACid:17986623	cassava4.1_000479m	ATP-dependent RNA helicase, putative	GP
cassava4.1_000480m|PACid:17963119	cassava4.1_000480m	ATPase E1-E2 type family protein / haloacid dehalogenase-like hydrolase family protein	No
cassava4.1_000497m|PACid:17989907	cassava4.1_000497m	catalytics;carbohydrate kinases;phosphoglucan, water dikinases	P
cassava4.1_000500m|PACid:17960052	cassava4.1_000500m	gigantea protein (GI)	G
cassava4.1_000504m|PACid:17993636	cassava4.1_000504m	aminophospholipid ATPase 3	G
cassava4.1_000508m|PACid:17983401	cassava4.1_000508m	cell division cycle protein 48-related / CDC48-related	G
cassava4.1_000509m|PACid:17989623	cassava4.1_000509m	HEAT repeat-containing protein	GP
cassava4.1_000512m|PACid:17990360	cassava4.1_000512m	cellulose synthase-like D5	No
cassava4.1_000515m|PACid:17976935	cassava4.1_000515m	ARM repeat superfamily protein	GP
cassava4.1_000518m|PACid:17984248	cassava4.1_000518m	Protein kinase superfamily protein	GP
cassava4.1_000528m|PACid:17991488	cassava4.1_000528m	starch synthase 3	P
cassava4.1_000529m|PACid:17965535	cassava4.1_000529m	cellulose synthase-like D3	G
cassava4.1_000530m|PACid:17966059	cassava4.1_000530m		No

cassava4.1_000531m|PACid:17982636	cassava4.1_000531m	phytochrome B	GP
cassava4.1_000532m|PACid:17961337	cassava4.1_000532m	DEA(D/H)-box RNA helicase family protein	No
cassava4.1_000537m|PACid:17983542	cassava4.1_000537m	receptor-like protein kinase 2	No
cassava4.1_000538m|PACid:17993465	cassava4.1_000538m	MUTL protein homolog 1	GP
cassava4.1_000540m|PACid:17987294	cassava4.1_000540m	TOPLESS-related 1	No
cassava4.1_000548m|PACid:17984682	cassava4.1_000548m	BRI1-like 2	G
cassava4.1_000549m|PACid:17992041	cassava4.1_000549m	aminophospholipid ATPase 1	GP
cassava4.1_000551m|PACid:17992071	cassava4.1_000551m	peroxisome 1	No
cassava4.1_000556m|PACid:17961421	cassava4.1_000556m	RNA-dependent RNA polymerase 1	GP
cassava4.1_000558m|PACid:17970675	cassava4.1_000558m	WUS-interacting protein 2	P
cassava4.1_000560m|PACid:17973906	cassava4.1_000560m	TOPLESS-related 3	GP
cassava4.1_000563m|PACid:17967926	cassava4.1_000563m	transducin family protein / WD-40 repeat family protein	GP
cassava4.1_000564m|PACid:17969909	cassava4.1_000564m	5\'-3\' exoribonuclease 3	No
cassava4.1_000565m|PACid:17962523	cassava4.1_000565m	P-loop containing nucleoside triphosphate hydrolases superfamily protein	GP
cassava4.1_000566m|PACid:17982637	cassava4.1_000566m	zinc knuckle (CCHC-type) family protein	No
cassava4.1_000568m|PACid:17971917	cassava4.1_000568m	phytochrome E	No
cassava4.1_000570m|PACid:17973927	cassava4.1_000570m	phytochrome C	G
cassava4.1_000571m|PACid:17984065	cassava4.1_000571m	golgi alpha-mannosidase II	GP
cassava4.1_000574m|PACid:17978623	cassava4.1_000574m	Quinoprotein amine dehydrogenase, beta chain-like	G
cassava4.1_000575m|PACid:17993302	cassava4.1_000575m	phytochrome A	GP
cassava4.1_000580m|PACid:17980490	cassava4.1_000580m	RING/U-box superfamily protein	No
cassava4.1_000583m|PACid:17981252	cassava4.1_000583m	P-loop containing nucleoside triphosphate hydrolases superfamily protein	No
cassava4.1_000584m|PACid:17982058	cassava4.1_000584m	Leucine-rich repeat protein kinase family protein	No
cassava4.1_000596m|PACid:17981587	cassava4.1_000596m	sister-chromatid cohesion protein 3	GP
cassava4.1_000597m|PACid:17968881	cassava4.1_000597m	carbamoyl phosphate synthetase B	GP
cassava4.1_000599m|PACid:17994086	cassava4.1_000599m	ubiquitin-specific protease 12	GP
cassava4.1_000601m|PACid:17982951	cassava4.1_000601m	proton gradient regulation 3	G
cassava4.1_000603m|PACid:17981722	cassava4.1_000603m	Regulator of chromosome condensation (RCC1) family with FYVE zinc finger domain	G
cassava4.1_000606m|PACid:17987175	cassava4.1_000606m	hydrolases, acting on ester bonds	No
cassava4.1_000607m|PACid:17989398	cassava4.1_000607m	Sec23/Sec24 protein transport family protein	P
cassava4.1_000609m|PACid:17992784	cassava4.1_000609m	auxin response factor 19	G
cassava4.1_000611m|PACid:17987565	cassava4.1_000611m	DegP protease 7	No
cassava4.1_000617m|PACid:17968184	cassava4.1_000617m	trithorax-like protein 2	G
cassava4.1_000619m|PACid:17979824	cassava4.1_000619m	aminophospholipid ATPase 2	GP
cassava4.1_000622m|PACid:17981943	cassava4.1_000622m	Regulator of chromosome condensation (RCC1) family with FYVE zinc finger domain	G
cassava4.1_000624m|PACid:17969123	cassava4.1_000624m	Leucine-rich repeat protein kinase family protein	No
cassava4.1_000626m|PACid:17984245	cassava4.1_000626m	NF-X-like 1	G
cassava4.1_000631m|PACid:17961780	cassava4.1_000631m	Helicase protein with RING/U-box domain	No
cassava4.1_000632m|PACid:17972356	cassava4.1_000632m	Homeotic gene regulator	No

cassava4.1_000633m|PACid:17970958	cassava4.1_000633m	P-loop nucleoside triphosphate hydrolases superfamily protein with CH (Calponin Homology) domain	G
cassava4.1_000635m|PACid:17964909	cassava4.1_000635m	ubiquitin activating enzyme 2	G
cassava4.1_000640m|PACid:17961819	cassava4.1_000640m	TRAF-like superfamily protein	G
cassava4.1_000642m|PACid:17990728	cassava4.1_000642m	binding	P
cassava4.1_000643m|PACid:17967284	cassava4.1_000643m	Leucine-rich receptor-like protein kinase family protein	No
cassava4.1_000644m|PACid:17973180	cassava4.1_000644m	Leucine-rich receptor-like protein kinase family protein	No
cassava4.1_000646m|PACid:17979617	cassava4.1_000646m	tRNA synthetase class I (I, L, M and V) family protein	No
cassava4.1_000650m|PACid:17992499	cassava4.1_000650m		GP
cassava4.1_000651m|PACid:17985956	cassava4.1_000651m	protein serine/threonine phosphatases;protein kinases;cAMP-dependent protein kinase regulators	GP
cassava4.1_000654m|PACid:17962519	cassava4.1_000654m	ATP binding;leucine-tRNA ligases;aminoacyl-tRNA ligases;nucleotide binding;ATP binding;aminoacyl-tRNA ligases	GP
cassava4.1_000655m|PACid:17960792	cassava4.1_000655m	homolog of DNA mismatch repair protein MSH3	No
cassava4.1_000660m|PACid:17983646	cassava4.1_000660m	cellulose synthase 1	G
cassava4.1_000661m|PACid:17978021	cassava4.1_000661m	methionine S-methyltransferase	No
cassava4.1_000662m|PACid:17961633	cassava4.1_000662m	ARM repeat superfamily protein	GP
cassava4.1_000666m|PACid:17989343	cassava4.1_000666m	FtsH extracellular protease family	No
cassava4.1_000670m|PACid:17960022	cassava4.1_000670m	Leucine-rich receptor-like protein kinase family protein	No
cassava4.1_000671m|PACid:17993597	cassava4.1_000671m	SIN3-like 4	No
cassava4.1_000673m|PACid:17974315	cassava4.1_000673m	Cellulose synthase family protein	No
cassava4.1_000678m|PACid:17967919	cassava4.1_000678m	Vps51/Vps67 family (components of vesicular transport) protein	GP
cassava4.1_000681m|PACid:17973826	cassava4.1_000681m	global transcription factor C	G
cassava4.1_000688m|PACid:17985518	cassava4.1_000688m	tripeptidyl peptidase ii	GP
cassava4.1_000689m|PACid:17962130	cassava4.1_000689m	Leucine-rich repeat receptor-like protein kinase family protein	G
cassava4.1_000691m|PACid:17968101	cassava4.1_000691m	Structural maintenance of chromosomes (SMC) family protein	GP
cassava4.1_000696m|PACid:17988809	cassava4.1_000696m	Leucine-rich receptor-like protein kinase family protein	GP
cassava4.1_000699m|PACid:17975315	cassava4.1_000699m	SUPPRESSOR OF AUXIN RESISTANCE 3	GP
cassava4.1_000700m|PACid:17988865	cassava4.1_000700m	glycine-tRNA ligases	GP
cassava4.1_000702m|PACid:17961763	cassava4.1_000702m	chromatin-remodeling protein 11	No
cassava4.1_000703m|PACid:17971494	cassava4.1_000703m	chromatin-remodeling protein 11	No
cassava4.1_000707m|PACid:17970004	cassava4.1_000707m	Guanylate-binding family protein	GP
cassava4.1_000709m|PACid:17965809	cassava4.1_000709m	ankyrin repeat family protein / regulator of chromosome condensation (RCC1) family protein	G
cassava4.1_000716m|PACid:17980972	cassava4.1_000716m	endomembrane-type CA-ATPase 4	GP
cassava4.1_000719m|PACid:17972058	cassava4.1_000719m	starch synthase 4	GP
cassava4.1_000720m|PACid:17961167	cassava4.1_000720m	Kinesin motor family protein	No
cassava4.1_000722m|PACid:17983114	cassava4.1_000722m	glycine decarboxylase P-protein 1	GP
cassava4.1_000724m|PACid:17974694	cassava4.1_000724m	Di-glucose binding protein with Kinesin motor domain	No
cassava4.1_000725m|PACid:17969179	cassava4.1_000725m	phosphoenolpyruvate carboxylase 4	GP
cassava4.1_000727m|PACid:17966415	cassava4.1_000727m		No
cassava4.1_000729m|PACid:17967821	cassava4.1_000729m	pumilio 2	G
cassava4.1_000730m|PACid:17986234	cassava4.1_000730m	ATPase E1-E2 type family protein / haloacid dehalogenase-like hydrolase family protein	G

cassava4.1_000732m|PACid:17989703	cassava4.1_000732m	sucrose phosphate synthase 1F	GP
cassava4.1_000735m|PACid:17964765	cassava4.1_000735m	ubiquitin-specific protease 26	No
cassava4.1_000736m|PACid:17967927	cassava4.1_000736m	structural maintenance of chromosomes 5	G
cassava4.1_000738m|PACid:17973015	cassava4.1_000738m	global transcription factor C	G
cassava4.1_000739m|PACid:17970568	cassava4.1_000739m	lysine-ketoglutarate reductase/saccharopine dehydrogenase bifunctional enzyme	GP
cassava4.1_000741m|PACid:17973057	cassava4.1_000741m	ARM repeat superfamily protein	GP
cassava4.1_000744m|PACid:17985909	cassava4.1_000744m	sucrose phosphate synthase 2F	GP
cassava4.1_000746m|PACid:17978573	cassava4.1_000746m	ER-type Ca2+-ATPase 2	G
cassava4.1_000747m|PACid:17987123	cassava4.1_000747m	phytosylfokine-alpha receptor 2	No
cassava4.1_000751m|PACid:17991692	cassava4.1_000751m	P-loop containing nucleoside triphosphate hydrolases superfamily protein	No
cassava4.1_000752m|PACid:17984262	cassava4.1_000752m	P-loop containing nucleoside triphosphate hydrolases superfamily protein	G
cassava4.1_000753m|PACid:17978125	cassava4.1_000753m	ARM repeat superfamily protein	GP
cassava4.1_000756m|PACid:17973614	cassava4.1_000756m	phosphatidylinositol 4-OH kinase beta1	G
cassava4.1_000760m|PACid:17983850	cassava4.1_000760m	homolog of histone chaperone HIRA	GP
cassava4.1_000761m|PACid:17992295	cassava4.1_000761m	RNA helicase family protein	No
cassava4.1_000764m|PACid:17968678	cassava4.1_000764m	global transcription factor group A2	GP
cassava4.1_000765m|PACid:17979210	cassava4.1_000765m	Leucine-rich repeat transmembrane protein kinase	No
cassava4.1_000768m|PACid:17982396	cassava4.1_000768m	26S proteasome regulatory complex, non-ATPase subcomplex, Rpn2/Psmd1 subunit	P
cassava4.1_000770m|PACid:17968400	cassava4.1_000770m	cellulose synthase A4	No
cassava4.1_000771m|PACid:17985955	cassava4.1_000771m	SITE-1 protease	No
cassava4.1_000773m|PACid:17993223	cassava4.1_000773m	haloacid dehalogenase-like hydrolase family protein	GP
cassava4.1_000778m|PACid:17976399	cassava4.1_000778m	SU(VAR)3-9 homolog 6	No
cassava4.1_000779m|PACid:17963946	cassava4.1_000779m	DNA/RNA helicase protein	No
cassava4.1_000780m|PACid:17977897	cassava4.1_000780m	histidine kinase 3	G
cassava4.1_000785m|PACid:17967617	cassava4.1_000785m	Sec23/Sec24 protein transport family protein	GP
cassava4.1_000788m|PACid:17977968	cassava4.1_000788m	ARM repeat superfamily protein	GP
cassava4.1_000790m|PACid:17981680	cassava4.1_000790m	zinc finger (CCCH type) helicase family protein	G
cassava4.1_000791m|PACid:17976241	cassava4.1_000791m	phospholipase D beta 1	GP
cassava4.1_000792m|PACid:17974141	cassava4.1_000792m	C2 calcium/lipid-binding plant phosphoribosyltransferase family protein	No
cassava4.1_000793m|PACid:17991516	cassava4.1_000793m	Met-10+ like family protein / kelch repeat-containing protein	G
cassava4.1_000803m|PACid:17985641	cassava4.1_000803m	Leucine-rich repeat transmembrane protein kinase	GP
cassava4.1_000806m|PACid:17964340	cassava4.1_000806m	Ribosomal protein S5/Elongation factor G/III/V family protein	No
cassava4.1_000809m|PACid:17990880	cassava4.1_000809m	C2 calcium/lipid-binding and GRAM domain containing protein	G
cassava4.1_000811m|PACid:17985269	cassava4.1_000811m	Leucine-rich repeat protein kinase family protein	No
cassava4.1_000812m|PACid:17973446	cassava4.1_000812m	Translation initiation factor 2, small GTP-binding protein	No
cassava4.1_000814m|PACid:17971214	cassava4.1_000814m	Domain of unknown function (DUF1726) ;Putative ATPase (DUF699)	No
cassava4.1_000822m|PACid:17992054	cassava4.1_000822m	2-oxoglutarate dehydrogenase, E1 component	P
cassava4.1_000823m|PACid:17986955	cassava4.1_000823m	protein tyrosine kinase family protein	No
cassava4.1_000824m|PACid:17960905	cassava4.1_000824m	RNA helicase family protein	GP

cassava4.1_000825m|PACid:17969273	cassava4.1_000825m	exportin 1A	P
cassava4.1_000828m|PACid:17989079	cassava4.1_000828m	2-oxoglutarate dehydrogenase, E1 component	P
cassava4.1_000832m|PACid:17984452	cassava4.1_000832m	autoinhibited Ca2+-ATPase 1	GP
cassava4.1_000834m|PACid:17973526	cassava4.1_000834m	Adaptor protein complex AP-2, alpha subunit	P
cassava4.1_000835m|PACid:17994056	cassava4.1_000835m		No
cassava4.1_000839m|PACid:17965412	cassava4.1_000839m	Sucrose-phosphate synthase family protein	No
cassava4.1_000845m|PACid:17963191	cassava4.1_000845m	Argonaute family protein	No
cassava4.1_000848m|PACid:17990531	cassava4.1_000848m	copper-exporting ATPase / responsive-to-antagonist 1 / copper-transporting ATPase (RAN1)	No
cassava4.1_000851m|PACid:17966120	cassava4.1_000851m	cell division cycle 5	GP
cassava4.1_000852m|PACid:17989417	cassava4.1_000852m	Leucine-rich receptor-like protein kinase family protein	No
cassava4.1_000853m|PACid:17981694	cassava4.1_000853m	P-loop nucleoside triphosphate hydrolases superfamily protein with CH (Calponin Homology) domain	No
cassava4.1_000858m|PACid:17993744	cassava4.1_000858m	calcium ATPase 2	No
cassava4.1_000859m|PACid:17961472	cassava4.1_000859m	CHASE domain containing histidine kinase protein	No
cassava4.1_000864m|PACid:17988309	cassava4.1_000864m	Leucine-rich receptor-like protein kinase family protein	GP
cassava4.1_000865m|PACid:17993188	cassava4.1_000865m	eukaryotic translation initiation factor 3A	GP
cassava4.1_000869m|PACid:17965070	cassava4.1_000869m	Glycosyl hydrolase family 38 protein	GP
cassava4.1_000870m|PACid:17966779	cassava4.1_000870m	SET domain group 29	G
cassava4.1_000871m|PACid:17977039	cassava4.1_000871m	ARM repeat superfamily protein	P
cassava4.1_000874m|PACid:17975717	cassava4.1_000874m	C2 calcium/lipid-binding plant phosphoribosyltransferase family protein	GP
cassava4.1_000875m|PACid:17964700	cassava4.1_000875m	Albino or Glassy Yellow 1	GP
cassava4.1_000878m|PACid:17976886	cassava4.1_000878m	MUTS homolog 6	G
cassava4.1_000879m|PACid:17970345	cassava4.1_000879m	F-box family protein	No
cassava4.1_000882m|PACid:17966279	cassava4.1_000882m	Vacuolar sorting protein 39	GP
cassava4.1_000885m|PACid:17967550	cassava4.1_000885m	26S proteasome regulatory complex, non-ATPase subcomplex, Rpn2/Psmd1 subunit	P
cassava4.1_000887m|PACid:17993593	cassava4.1_000887m	Leucine-rich receptor-like protein kinase family protein	G
cassava4.1_000893m|PACid:17983569	cassava4.1_000893m	U-box domain-containing protein	GP
cassava4.1_000894m|PACid:17980957	cassava4.1_000894m	phototropin 1	GP
cassava4.1_000896m|PACid:17981516	cassava4.1_000896m	homolog of yeast autophagy 18 (ATG18) G	G
cassava4.1_000897m|PACid:17991003	cassava4.1_000897m	BRI1 suppressor 1 (BSU1)-like 2	GP
cassava4.1_000899m|PACid:17974353	cassava4.1_000899m	FTSH protease 12	GP
cassava4.1_000900m|PACid:17970964	cassava4.1_000900m	C2 calcium/lipid-binding plant phosphoribosyltransferase family protein	No
cassava4.1_000902m|PACid:17973101	cassava4.1_000902m	nuclear RNA polymerase A2	GP
cassava4.1_000907m|PACid:17964029	cassava4.1_000907m	Alanyl-tRNA synthetase, class IIc	GP
cassava4.1_000908m|PACid:17965103	cassava4.1_000908m	aconitase 3	GP
cassava4.1_000910m|PACid:17973253	cassava4.1_000910m	SNF2 domain-containing protein / helicase domain-containing protein / zinc finger protein-related	No
cassava4.1_000911m|PACid:17982099	cassava4.1_000911m	autoinhibited Ca2+-ATPase 11	No
cassava4.1_000913m|PACid:17980242	cassava4.1_000913m	NAD kinase 2	G
cassava4.1_000923m|PACid:17984191	cassava4.1_000923m	mitogen-activated protein kinase kinase kinase 7	GP
cassava4.1_000928m|PACid:17968938	cassava4.1_000928m	male gametophyte defective 3	G

cassava4.1_000929m|PACid:17987466	cassava4.1_000929m	DNA/RNA polymerases superfamily protein	No
cassava4.1_000930m|PACid:17977518	cassava4.1_000930m	HAESA-like 1	GP
cassava4.1_000932m|PACid:17961839	cassava4.1_000932m	Stabilizer of iron transporter SufD / Polynucleotidyl transferase	No
cassava4.1_000933m|PACid:17975985	cassava4.1_000933m	ARM repeat superfamily protein	G
cassava4.1_000938m|PACid:17987969	cassava4.1_000938m	arabinose kinase	No
cassava4.1_000940m|PACid:17963892	cassava4.1_000940m	Argonaute family protein	GP
cassava4.1_000941m|PACid:17964836	cassava4.1_000941m	RNA helicase, ATP-dependent, SK12/DOB1 protein	G
cassava4.1_000946m|PACid:17993452	cassava4.1_000946m	Stabilizer of iron transporter SufD / Polynucleotidyl transferase	G
cassava4.1_000948m|PACid:17972696	cassava4.1_000948m	Ribosomal protein S5/Elongation factor G/III/V family protein	P
cassava4.1_000949m|PACid:17967397	cassava4.1_000949m	TUDOR-SN protein 1	GP
cassava4.1_000950m|PACid:17970536	cassava4.1_000950m	Leucine-rich receptor-like protein kinase family protein	No
cassava4.1_000953m|PACid:17965041	cassava4.1_000953m	TUDOR-SN protein 1	GP
cassava4.1_000958m|PACid:17974497	cassava4.1_000958m	RNA helicase, ATP-dependent, SK12/DOB1 protein	No
cassava4.1_000960m|PACid:17971101	cassava4.1_000960m	zinc ion binding	GP
cassava4.1_000961m|PACid:17968041	cassava4.1_000961m	phototropin 2	No
cassava4.1_000962m|PACid:17974881	cassava4.1_000962m	HAESA-like 1	G
cassava4.1_000963m|PACid:17979042	cassava4.1_000963m	heavy metal atpase 5	No
cassava4.1_000966m|PACid:17982579	cassava4.1_000966m	catalytics	GP
cassava4.1_000969m|PACid:17973936	cassava4.1_000969m	polymerase gamma 2	No
cassava4.1_000970m|PACid:17987082	cassava4.1_000970m	Leucine-rich receptor-like protein kinase family protein	No
cassava4.1_000972m|PACid:17970431	cassava4.1_000972m	DEAD/DEAH box RNA helicase family protein	G
cassava4.1_000976m|PACid:17989854	cassava4.1_000976m	transferases;sulfuric ester hydrolases;catalytics;transferases	No
cassava4.1_000980m|PACid:17985821	cassava4.1_000980m	Insulinase (Peptidase family M16) protein	G
cassava4.1_000981m|PACid:17969244	cassava4.1_000981m	P-loop containing nucleoside triphosphate hydrolases superfamily protein	G
cassava4.1_000982m|PACid:17961705	cassava4.1_000982m	Protein kinase superfamily protein	No
cassava4.1_000983m|PACid:17960485	cassava4.1_000983m	Leucine-rich receptor-like protein kinase family protein	No
cassava4.1_000991m|PACid:17987755	cassava4.1_000991m	formin homology5	GP
cassava4.1_000992m|PACid:17975850	cassava4.1_000992m	cellulose synthase family protein	G
cassava4.1_000997m|PACid:17959869	cassava4.1_000997m	RNA recognition motif (RRM)-containing protein	No
cassava4.1_000998m|PACid:17971850	cassava4.1_000998m	transferases, transferring glycosyl groups	G
cassava4.1_001000m|PACid:17973828	cassava4.1_001000m	lon protease 1	P
cassava4.1_001005m|PACid:17959837	cassava4.1_001005m	casein lytic proteinase B4	GP
cassava4.1_001007m|PACid:17990325	cassava4.1_001007m	Prolyl oligopeptidase family protein	GP
cassava4.1_001010m|PACid:17962777	cassava4.1_001010m	transducin family protein / WD-40 repeat family protein	G
cassava4.1_001013m|PACid:17959997	cassava4.1_001013m	ubiquitin-specific protease 10	No
cassava4.1_001014m|PACid:17992865	cassava4.1_001014m	glycoside hydrolase family 2 protein	GP
cassava4.1_001015m|PACid:17982053	cassava4.1_001015m	Tetratricopeptide repeat (TPR)-like superfamily protein	No
cassava4.1_001017m|PACid:17968500	cassava4.1_001017m	Leucine-rich receptor-like protein kinase family protein	GP
cassava4.1_001019m|PACid:17974400	cassava4.1_001019m	valyl-tRNA synthetase / valine--tRNA ligase (VALRS)	P

cassava4.1_001025m|PACid:17972512	cassava4.1_001025m	replication factor C1	GP
cassava4.1_001026m|PACid:17978465	cassava4.1_001026m	cellular apoptosis susceptibility protein, putative / importin-alpha re-exporter, putative	P
cassava4.1_001029m|PACid:17961079	cassava4.1_001029m	KCBP-interacting protein kinase	No
cassava4.1_001034m|PACid:17970930	cassava4.1_001034m	Alkaline-phosphatase-like family protein	No
cassava4.1_001038m|PACid:17972736	cassava4.1_001038m	dicer-like 4	No
cassava4.1_001040m|PACid:17978860	cassava4.1_001040m	casein lytic proteinase B3	P
cassava4.1_001045m|PACid:17987109	cassava4.1_001045m	phosphoenolpyruvate carboxylase 3	P
cassava4.1_001046m|PACid:17983997	cassava4.1_001046m	villin 4	G
cassava4.1_001047m|PACid:17993086	cassava4.1_001047m	phosphoenolpyruvate carboxylase 1	GP
cassava4.1_001052m|PACid:17993589	cassava4.1_001052m	villin 2	GP
cassava4.1_001055m|PACid:17959859	cassava4.1_001055m	ABC1 family protein	No
cassava4.1_001056m|PACid:17979984	cassava4.1_001056m	ATP binding microtubule motor family protein	No
cassava4.1_001057m|PACid:17990837	cassava4.1_001057m	C-terminal domain phosphatase-like 1	No
cassava4.1_001059m|PACid:17986101	cassava4.1_001059m	Leucine-rich repeat protein kinase family protein	No
cassava4.1_001061m|PACid:17969270	cassava4.1_001061m	Leucine-rich repeat protein kinase family protein	No
cassava4.1_001063m|PACid:17963646	cassava4.1_001063m	vacuolar protein sorting 11	P
cassava4.1_001066m|PACid:17973977	cassava4.1_001066m	ARM repeat superfamily protein	No
cassava4.1_001076m|PACid:17991648	cassava4.1_001076m	Chaperone DnaJ-domain superfamily protein	No
cassava4.1_001079m|PACid:17964302	cassava4.1_001079m	D-alanine--D-alanine ligase family	GP
cassava4.1_001080m|PACid:17974182	cassava4.1_001080m	H(+)-ATPase 11	GP
cassava4.1_001082m|PACid:17968524	cassava4.1_001082m	Leucine-rich repeat transmembrane protein kinase family protein	No
cassava4.1_001085m|PACid:17965332	cassava4.1_001085m	presequence protease 1	GP
cassava4.1_001086m|PACid:17969334	cassava4.1_001086m	disproportionating enzyme 2	GP
cassava4.1_001092m|PACid:17967369	cassava4.1_001092m	Clp ATPase	GP
cassava4.1_001093m|PACid:17965095	cassava4.1_001093m	Alanyl-tRNA synthetase	P
cassava4.1_001094m|PACid:17970867	cassava4.1_001094m	Leucine-rich repeat protein kinase family protein	No
cassava4.1_001095m|PACid:17976351	cassava4.1_001095m	Restriction endonuclease, type II-like superfamily protein	G
cassava4.1_001096m|PACid:17985601	cassava4.1_001096m	Glycosyl transferase, family 35	P
cassava4.1_001099m|PACid:17987093	cassava4.1_001099m	transducin family protein / WD-40 repeat family protein	GP
cassava4.1_001103m|PACid:17967754	cassava4.1_001103m	splicing factor PWI domain-containing protein	GP
cassava4.1_001104m|PACid:17960917	cassava4.1_001104m	H(+)-ATPase 5	GP
cassava4.1_001105m|PACid:17990075	cassava4.1_001105m	Transcriptional factor B3 family protein / auxin-responsive factor AUX/IAA-related	No
cassava4.1_001106m|PACid:17981835	cassava4.1_001106m	Glycosyl hydrolase family 38 protein	P
cassava4.1_001107m|PACid:17961315	cassava4.1_001107m	Leucine-rich receptor-like protein kinase family protein	G
cassava4.1_001108m|PACid:17974816	cassava4.1_001108m	Beta-glucosidase, GBA2 type family protein	No
cassava4.1_001110m|PACid:17973139	cassava4.1_001110m	autoinhibited H(+)-ATPase isoform 10	GP
cassava4.1_001111m|PACid:17979454	cassava4.1_001111m	transmembrane kinase 1	G
cassava4.1_001112m|PACid:17993882	cassava4.1_001112m	RNAse E/G-like	G
cassava4.1_001113m|PACid:17973967	cassava4.1_001113m	ATP-binding cassette A2	GP

cassava4.1_001117m|PACid:17964580	cassava4.1_001117m	ERECTA-like 1	No
cassava4.1_001119m|PACid:17971431	cassava4.1_001119m	tetratricopeptide repeat (TPR)-containing protein	No
cassava4.1_001120m|PACid:17987232	cassava4.1_001120m	Transducin/WD40 repeat-like superfamily protein	G
cassava4.1_001129m|PACid:17984152	cassava4.1_001129m	golgin candidate 5	GP
cassava4.1_001133m|PACid:17965283	cassava4.1_001133m	ATP binding microtubule motor family protein	No
cassava4.1_001135m|PACid:17959808	cassava4.1_001135m	Coatomer, beta subunit	No
cassava4.1_001139m|PACid:17975196	cassava4.1_001139m	polyribonucleotide nucleotidyltransferase, putative	No
cassava4.1_001141m|PACid:17980456	cassava4.1_001141m	ARM repeat superfamily protein	P
cassava4.1_001142m|PACid:17992656	cassava4.1_001142m	Pyridoxal phosphate (PLP)-dependent transferases superfamily protein	No
cassava4.1_001145m|PACid:17973351	cassava4.1_001145m	HAESA-like 2	No
cassava4.1_001149m|PACid:17984105	cassava4.1_001149m	tRNAse Z4	G
cassava4.1_001157m|PACid:17971728	cassava4.1_001157m	minichromosome maintenance (MCM2/3/5) family protein	No
cassava4.1_001159m|PACid:17977554	cassava4.1_001159m	formin homology 1	G
cassava4.1_001164m|PACid:17965142	cassava4.1_001164m	transmembrane kinase 1	No
cassava4.1_001167m|PACid:17963954	cassava4.1_001167m	vacuolar protein sorting 41	No
cassava4.1_001172m|PACid:17990516	cassava4.1_001172m	ubiquitin-specific protease 23	G
cassava4.1_001174m|PACid:17988623	cassava4.1_001174m	peroxin 6	G
cassava4.1_001178m|PACid:17989361	cassava4.1_001178m	exoribonuclease 4	No
cassava4.1_001182m|PACid:17963415	cassava4.1_001182m	Leucine-rich repeat protein kinase family protein	G
cassava4.1_001186m|PACid:17978040	cassava4.1_001186m	ATP binding microtubule motor family protein	G
cassava4.1_001189m|PACid:17982618	cassava4.1_001189m	Tetratricopeptide repeat (TPR)-like superfamily protein	No
cassava4.1_001192m|PACid:17965026	cassava4.1_001192m	dual specificity protein phosphatase family protein	No
cassava4.1_001193m|PACid:17970955	cassava4.1_001193m	respiratory burst oxidase protein F	No
cassava4.1_001195m|PACid:17961257	cassava4.1_001195m	Glycosyl hydrolases family 31 protein	GP
cassava4.1_001203m|PACid:17994057	cassava4.1_001203m	eukaryotic translation initiation factor 3C	P
cassava4.1_001211m|PACid:17959950	cassava4.1_001211m	eukaryotic translation initiation factor 3C	No
cassava4.1_001215m|PACid:17973440	cassava4.1_001215m	trehalose-6-phosphate synthase	No
cassava4.1_001220m|PACid:17989148	cassava4.1_001220m	ARABIDILLO-1	No
cassava4.1_001222m|PACid:17992366	cassava4.1_001222m	Tetratricopeptide repeat (TPR)-like superfamily protein	G
cassava4.1_001223m|PACid:17990069	cassava4.1_001223m	trehalose-6-phosphate synthase	No
cassava4.1_001224m|PACid:17979812	cassava4.1_001224m	Leucine-rich repeat protein kinase family protein	No
cassava4.1_001231m|PACid:17965021	cassava4.1_001231m	CLPC homologue 1	P
cassava4.1_001235m|PACid:17984598	cassava4.1_001235m	glutamate receptor 2	G
cassava4.1_001236m|PACid:17990580	cassava4.1_001236m	Leucine-rich repeat protein kinase family protein	No
cassava4.1_001237m|PACid:17990499	cassava4.1_001237m	Protein kinase family protein with ARM repeat domain	GP
cassava4.1_001241m|PACid:17973318	cassava4.1_001241m	PLAT/LH2 domain-containing lipoxygenase family protein	GP
cassava4.1_001244m|PACid:17969107	cassava4.1_001244m	alpha-xylosidase 1	P
cassava4.1_001250m|PACid:17968495	cassava4.1_001250m	glycosyl hydrolase family 10 protein / carbohydrate-binding domain-containing protein	No
cassava4.1_001254m|PACid:17973678	cassava4.1_001254m	aspartate kinase-homoserine dehydrogenase ii	GP

cassava4.1_001255m|PACid:17971173	cassava4.1_001255m	golgin candidate 6	P
cassava4.1_001256m|PACid:17968726	cassava4.1_001256m	para-aminobenzoate (PABA) synthase family protein	No
cassava4.1_001258m|PACid:17990863	cassava4.1_001258m	Tetratricopeptide repeat (TPR)-like superfamily protein	GP
cassava4.1_001259m|PACid:17963396	cassava4.1_001259m	PLAT/LH2 domain-containing lipoxygenase family protein	GP
cassava4.1_001261m|PACid:17966694	cassava4.1_001261m	armadillo repeat kinesin 3	No
cassava4.1_001270m|PACid:17980065	cassava4.1_001270m	respiratory burst oxidase homologue D	GP
cassava4.1_001272m|PACid:17986580	cassava4.1_001272m	dynamin-like 3	P
cassava4.1_001275m|PACid:17976658	cassava4.1_001275m	Coatomer, beta\' subunit	No
cassava4.1_001280m|PACid:17967866	cassava4.1_001280m	Cellulose synthase family protein	No
cassava4.1_001283m|PACid:17991130	cassava4.1_001283m	sucrose synthase 6	P
cassava4.1_001287m|PACid:17969648	cassava4.1_001287m	Adaptin family protein	P
cassava4.1_001289m|PACid:17984526	cassava4.1_001289m	SEUSS transcriptional co-regulator	No
cassava4.1_001294m|PACid:17974129	cassava4.1_001294m	pantothenate kinase 2	No
cassava4.1_001300m|PACid:17962766	cassava4.1_001300m	heat shock protein 101	GP
cassava4.1_001302m|PACid:17965083	cassava4.1_001302m	NADPH/respiratory burst oxidase protein D	No
cassava4.1_001305m|PACid:17966586	cassava4.1_001305m	Argonaute family protein	GP
cassava4.1_001308m|PACid:17971192	cassava4.1_001308m	Protein kinase superfamily protein	G
cassava4.1_001309m|PACid:17986735	cassava4.1_001309m	heavy metal atpase 2	G
cassava4.1_001313m|PACid:17992631	cassava4.1_001313m	extra-large G-protein 1	G
cassava4.1_001315m|PACid:17971541	cassava4.1_001315m	SART-1 family	GP
cassava4.1_001317m|PACid:17964651	cassava4.1_001317m	Protein phosphatase 2C family protein	G
cassava4.1_001319m|PACid:17987272	cassava4.1_001319m	SPA1-related 3	G
cassava4.1_001326m|PACid:17981914	cassava4.1_001326m	Adaptin family protein	GP
cassava4.1_001337m|PACid:17976252	cassava4.1_001337m	armadillo repeat kinesin 2	No
cassava4.1_001338m|PACid:17972565	cassava4.1_001338m	Pre-mRNA-splicing factor 3	No
cassava4.1_001341m|PACid:17981088	cassava4.1_001341m	periodic tryptophan protein 2	GP
cassava4.1_001342m|PACid:17982998	cassava4.1_001342m	tetratricopeptide repeat (TPR)-containing protein	GP
cassava4.1_001343m|PACid:17991101	cassava4.1_001343m	DEA(D/H)-box RNA helicase family protein	No
cassava4.1_001344m|PACid:17974520	cassava4.1_001344m	transcription regulator NOT2/NOT3/NOT5 family protein	No
cassava4.1_001345m|PACid:17979866	cassava4.1_001345m	switch 2	No
cassava4.1_001348m|PACid:17966462	cassava4.1_001348m	aconitase 1	GP
cassava4.1_001357m|PACid:17970373	cassava4.1_001357m	auxin response factor 8	G
cassava4.1_001362m|PACid:17970800	cassava4.1_001362m	alpha-amylase-like 3	GP
cassava4.1_001365m|PACid:17977336	cassava4.1_001365m	Plant protein of unknown function (DUF827)	P
cassava4.1_001366m|PACid:17992946	cassava4.1_001366m	SET domain-containing protein	No
cassava4.1_001367m|PACid:17970125	cassava4.1_001367m	Transducin/WD40 repeat-like superfamily protein	No
cassava4.1_001370m|PACid:17965926	cassava4.1_001370m	Malectin/receptor-like protein kinase family protein	GP
cassava4.1_001373m|PACid:17981658	cassava4.1_001373m	ETO1-like 1	No
cassava4.1_001385m|PACid:17960448	cassava4.1_001385m	exocyst complex component sec3A	No

cassava4.1_001386m|PACid:17990602	cassava4.1_001386m	Leucine-rich repeat protein kinase family protein	G
cassava4.1_001387m|PACid:17972153	cassava4.1_001387m	lon protease 2	GP
cassava4.1_001391m|PACid:17969732	cassava4.1_001391m	coatomer gamma-2 subunit, putative / gamma-2 coat protein, putative / gamma-2 COP, putative	No
cassava4.1_001394m|PACid:17981663	cassava4.1_001394m	RELA/SPOT homolog 1	No
cassava4.1_001395m|PACid:17965718	cassava4.1_001395m	Transducin family protein / WD-40 repeat family protein	G
cassava4.1_001398m|PACid:17979930	cassava4.1_001398m	Leucine-rich repeat receptor-like protein kinase family protein	No
cassava4.1_001399m|PACid:17979676	cassava4.1_001399m	Tetratricopeptide repeat (TPR)-like superfamily protein	No
cassava4.1_001402m|PACid:17986337	cassava4.1_001402m	BRI1 suppressor 1 (BSU1)-like 1	GP
cassava4.1_001407m|PACid:17974373	cassava4.1_001407m	Leucine-rich repeat transmembrane protein kinase	No
cassava4.1_001409m|PACid:17966603	cassava4.1_001409m	respiratory burst oxidase homolog B	GP
cassava4.1_001410m|PACid:17970348	cassava4.1_001410m	P-type ATPase of Arabidopsis 2	G
cassava4.1_001420m|PACid:17961939	cassava4.1_001420m	binding	GP
cassava4.1_001421m|PACid:17960219	cassava4.1_001421m	MUTS homolog 2	No
cassava4.1_001423m|PACid:17993501	cassava4.1_001423m	P-loop containing nucleoside triphosphate hydrolases superfamily protein	G
cassava4.1_001426m|PACid:17980017	cassava4.1_001426m	anaphase-promoting complex/cyclosome 2	GP
cassava4.1_001428m|PACid:17984148	cassava4.1_001428m	AAA-type ATPase family protein	GP
cassava4.1_001429m|PACid:17974374	cassava4.1_001429m	Protein kinase superfamily protein with octicosapeptide/Phox/Bem1p domain	No
cassava4.1_001431m|PACid:17965681	cassava4.1_001431m	lipoxygenase 1	GP
cassava4.1_001434m|PACid:17982904	cassava4.1_001434m	Class II aaRS and biotin synthetases superfamily protein	GP
cassava4.1_001436m|PACid:17975119	cassava4.1_001436m	DNA topoisomerase 1 beta	G
cassava4.1_001446m|PACid:17967561	cassava4.1_001446m	DNA topoisomerase I alpha	No
cassava4.1_001447m|PACid:17971033	cassava4.1_001447m	Protein kinase superfamily protein	G
cassava4.1_001451m|PACid:17963562	cassava4.1_001451m	Adaptor protein complex AP-1, gamma subunit	P
cassava4.1_001453m|PACid:17964683	cassava4.1_001453m	Sec23/Sec24 protein transport family protein	No
cassava4.1_001460m|PACid:17981923	cassava4.1_001460m	Rho GTPase activation protein (RhoGAP) with PH domain	G
cassava4.1_001462m|PACid:17976882	cassava4.1_001462m	FAR1-related sequence 3	GP
cassava4.1_001468m|PACid:17980847	cassava4.1_001468m	DNA-binding protein with MIZ/SP-RING zinc finger, PHD-finger and SAP domain	G
cassava4.1_001470m|PACid:17964165	cassava4.1_001470m	26S proteasome regulatory subunit S2 1A	GP
cassava4.1_001472m|PACid:17976507	cassava4.1_001472m	Endosomal targeting BRO1-like domain-containing protein	GP
cassava4.1_001482m|PACid:17963423	cassava4.1_001482m	Zn-dependent exopeptidases superfamily protein	GP
cassava4.1_001484m|PACid:17962982	cassava4.1_001484m	Tetratricopeptide repeat (TPR)-like superfamily protein	No
cassava4.1_001488m|PACid:17988208	cassava4.1_001488m	ARM repeat superfamily protein	No
cassava4.1_001497m|PACid:17982480	cassava4.1_001497m	RNA recognition motif (RRM)-containing protein	No
cassava4.1_001498m|PACid:17970543	cassava4.1_001498m	aminopeptidase M1	P
cassava4.1_001500m|PACid:17985653	cassava4.1_001500m	retinoblastoma-related 1	No
cassava4.1_001502m|PACid:17961111	cassava4.1_001502m	Protein kinase superfamily protein	No
cassava4.1_001506m|PACid:17962015	cassava4.1_001506m	PLAT/LH2 domain-containing lipoxygenase family protein	GP
cassava4.1_001510m|PACid:17979708	cassava4.1_001510m	BTB/POZ domain-containing protein	No
cassava4.1_001512m|PACid:17970117	cassava4.1_001512m	RING/U-box superfamily protein	No

cassava4.1_001515m|PACid:17963017	cassava4.1_001515m	CRS1 / YhbY (CRM) domain-containing protein	G
cassava4.1_001517m|PACid:17976963	cassava4.1_001517m	trehalose-phosphatase/synthase 9	GP
cassava4.1_001534m|PACid:17987323	cassava4.1_001534m	Nucleoporin interacting component (Nup93/Nic96-like) family protein	No
cassava4.1_001535m|PACid:17972005	cassava4.1_001535m	MEI2-like protein 5	No
cassava4.1_001537m|PACid:17963535	cassava4.1_001537m	trehalose-phosphatase/synthase 9	GP
cassava4.1_001541m|PACid:17991854	cassava4.1_001541m	trehalose phosphate synthase	No
cassava4.1_001543m|PACid:17961998	cassava4.1_001543m	Tetratricopeptide repeat (TPR)-like superfamily protein	No
cassava4.1_001548m|PACid:17966551	cassava4.1_001548m	extra-large GTP-binding protein 3	GP
cassava4.1_001557m|PACid:17966319	cassava4.1_001557m	phospholipase D delta	No
cassava4.1_001558m|PACid:17966187	cassava4.1_001558m	trehalose phosphatase/synthase 5	No
cassava4.1_001559m|PACid:17981360	cassava4.1_001559m	spindle pole body component 98	No
cassava4.1_001560m|PACid:17967424	cassava4.1_001560m	RNA-metabolising metallo-beta-lactamase family protein	No
cassava4.1_001564m|PACid:17991264	cassava4.1_001564m	heat shock protein 70 (Hsp 70) family protein	GP
cassava4.1_001567m|PACid:17965008	cassava4.1_001567m	auxin response factor 2	G
cassava4.1_001568m|PACid:17977468	cassava4.1_001568m	pyruvate orthophosphate dikinase	GP
cassava4.1_001581m|PACid:17975540	cassava4.1_001581m	AMP deaminase, putative / myoadenylate deaminase, putative	No
cassava4.1_001583m|PACid:17965118	cassava4.1_001583m	UDP-Glycosyltransferase / trehalose-phosphatase family protein	GP
cassava4.1_001588m|PACid:17966846	cassava4.1_001588m	origin recognition complex 1	G
cassava4.1_001591m|PACid:17972085	cassava4.1_001591m	trehalose-phosphatase/synthase 7	GP
cassava4.1_001595m|PACid:17966954	cassava4.1_001595m	starch branching enzyme 2.1	GP
cassava4.1_001603m|PACid:17979503	cassava4.1_001603m	Golgi transport complex protein-related	GP
cassava4.1_001607m|PACid:17969515	cassava4.1_001607m	Heat shock protein 70 (Hsp 70) family protein	GP
cassava4.1_001610m|PACid:17980930	cassava4.1_001610m	SIT4 phosphatase-associated family protein	No
cassava4.1_001614m|PACid:17994154	cassava4.1_001614m	vacuoleless1 (VCL1)	GP
cassava4.1_001619m|PACid:17985458	cassava4.1_001619m	Homeobox-leucine zipper family protein / lipid-binding START domain-containing protein	No
cassava4.1_001620m|PACid:17966689	cassava4.1_001620m	phospholipase D beta 1	GP
cassava4.1_001621m|PACid:17960470	cassava4.1_001621m	beta-galactosidase 3	GP
cassava4.1_001626m|PACid:17966328	cassava4.1_001626m	alpha-glucan phosphorylase 2	GP
cassava4.1_001629m|PACid:17991140	cassava4.1_001629m	elongator protein 2	No
cassava4.1_001631m|PACid:17971134	cassava4.1_001631m	DEAD box RNA helicase family protein	GP
cassava4.1_001634m|PACid:17965272	cassava4.1_001634m	Phosphoinositide phosphatase family protein	G
cassava4.1_001637m|PACid:17966346	cassava4.1_001637m	beta-galactosidase 3	No
cassava4.1_001645m|PACid:17974596	cassava4.1_001645m	Ribosomal protein S5/Elongation factor G/III/V family protein	G
cassava4.1_001649m|PACid:17980963	cassava4.1_001649m	Homeobox-leucine zipper family protein / lipid-binding START domain-containing protein	No
cassava4.1_001650m|PACid:17960319	cassava4.1_001650m	adaptin family protein	GP
cassava4.1_001652m|PACid:17975963	cassava4.1_001652m	beta galactosidase 1	G
cassava4.1_001656m|PACid:17983769	cassava4.1_001656m	potassium transport 2/3	No
cassava4.1_001663m|PACid:17983946	cassava4.1_001663m	minichromosome maintenance (MCM2/3/5) family protein	No
cassava4.1_001668m|PACid:17968421	cassava4.1_001668m	Potassium transporter family protein	GP

cassava4.1_001669m|PACid:17993984	cassava4.1_001669m	RING/U-box superfamily protein with ARM repeat domain	GP
cassava4.1_001673m|PACid:17971534	cassava4.1_001673m	Homeobox-leucine zipper family protein / lipid-binding START domain-containing protein	No
cassava4.1_001674m|PACid:17983740	cassava4.1_001674m	Homeobox-leucine zipper family protein / lipid-binding START domain-containing protein	No
cassava4.1_001676m|PACid:17974654	cassava4.1_001676m	receptor kinase 3	No
cassava4.1_001678m|PACid:17993899	cassava4.1_001678m	tubulin-tyrosine ligases;tubulin-tyrosine ligases	GP
cassava4.1_001686m|PACid:17972441	cassava4.1_001686m	starch branching enzyme 2.2	GP
cassava4.1_001688m|PACid:17983157	cassava4.1_001688m	inflorescence meristem receptor-like kinase 2	P
cassava4.1_001690m|PACid:17988658	cassava4.1_001690m	S-adenosyl-L-methionine-dependent methyltransferases superfamily protein	GP
cassava4.1_001692m|PACid:17989960	cassava4.1_001692m	P-loop containing nucleoside triphosphate hydrolases superfamily protein	P
cassava4.1_001695m|PACid:17984734	cassava4.1_001695m	Subtilase family protein	No
cassava4.1_001697m|PACid:17960813	cassava4.1_001697m	P-loop containing nucleoside triphosphate hydrolases superfamily protein	GP
cassava4.1_001699m|PACid:17968307	cassava4.1_001699m	binding	No
cassava4.1_001703m|PACid:17964581	cassava4.1_001703m	conserved oligomeric Golgi complex component-related / COG complex component-related	No
cassava4.1_001704m|PACid:17980942	cassava4.1_001704m	hercules receptor kinase 1	No
cassava4.1_001706m|PACid:17978075	cassava4.1_001706m	polyribonucleotide nucleotidyltransferase, putative	No
cassava4.1_001709m|PACid:17991306	cassava4.1_001709m	FtsJ-like methyltransferase family protein	No
cassava4.1_001713m|PACid:17980672	cassava4.1_001713m	dynamin-related protein 3A	GP
cassava4.1_001721m|PACid:17980392	cassava4.1_001721m	phosphatidyl inositol monophosphate 5 kinase	No
cassava4.1_001724m|PACid:17979654	cassava4.1_001724m	acyl-CoA dehydrogenase-related	GP
cassava4.1_001726m|PACid:17985494	cassava4.1_001726m	DEAD/DEAH box helicase, putative	G
cassava4.1_001727m|PACid:17991670	cassava4.1_001727m	hercules receptor kinase 1	GP
cassava4.1_001732m|PACid:17972328	cassava4.1_001732m	ARF GTPase-activating protein	G
cassava4.1_001734m|PACid:17980552	cassava4.1_001734m	heavy metal atpase 1	No
cassava4.1_001736m|PACid:17990008	cassava4.1_001736m	Protein kinase superfamily protein	GP
cassava4.1_001746m|PACid:17962414	cassava4.1_001746m	Tetratricopeptide repeat (TPR)-like superfamily protein	No
cassava4.1_001758m|PACid:17960764	cassava4.1_001758m	FTSH protease 11	GP
cassava4.1_001760m|PACid:17976375	cassava4.1_001760m	DWNN domain, a CCHC-type zinc finger	G
cassava4.1_001764m|PACid:17976209	cassava4.1_001764m	Homeobox-leucine zipper family protein / lipid-binding START domain-containing protein	No
cassava4.1_001766m|PACid:17963691	cassava4.1_001766m	P-loop containing nucleoside triphosphate hydrolases superfamily protein	GP
cassava4.1_001767m|PACid:17966409	cassava4.1_001767m	FTSH protease 9	No
cassava4.1_001768m|PACid:17970385	cassava4.1_001768m	cullin4	No
cassava4.1_001769m|PACid:17972845	cassava4.1_001769m	auxin response factor 8	GP
cassava4.1_001770m|PACid:17966899	cassava4.1_001770m	mitochondrial substrate carrier family protein	GP
cassava4.1_001774m|PACid:17993073	cassava4.1_001774m	Mitogen activated protein kinase kinase kinase-related	No
cassava4.1_001780m|PACid:17969414	cassava4.1_001780m	S-adenosyl-L-methionine-dependent methyltransferases superfamily protein	GP
cassava4.1_001785m|PACid:17992196	cassava4.1_001785m	pre-mRNA splicing factor-related	GP
cassava4.1_001792m|PACid:17988193	cassava4.1_001792m	Transducin family protein / WD-40 repeat family protein	No
cassava4.1_001793m|PACid:17990036	cassava4.1_001793m	Membrane trafficking VPS53 family protein	GP
cassava4.1_001798m|PACid:17979388	cassava4.1_001798m	Mitogen activated protein kinase kinase kinase-related	G

cassava4.1_001801m|PACid:17992095	cassava4.1_001801m	PA-domain containing subtilase family protein	No
cassava4.1_001802m|PACid:17974277	cassava4.1_001802m	Cobalamin-independent synthase family protein	GP
cassava4.1_001804m|PACid:17993339	cassava4.1_001804m	PA-domain containing subtilase family protein	GP
cassava4.1_001805m|PACid:17971275	cassava4.1_001805m	molybdenum cofactor sulfurase (LOS5) (ABA3)	G
cassava4.1_001810m|PACid:17992004	cassava4.1_001810m	Transducin/WD40 repeat-like superfamily protein	No
cassava4.1_001811m|PACid:17961683	cassava4.1_001811m	homolog of yeast autophagy 18 (ATG18) H	G
cassava4.1_001812m|PACid:17972320	cassava4.1_001812m	LSD1-like 1	No
cassava4.1_001818m|PACid:17992831	cassava4.1_001818m	vacuolar protein sorting 34	G
cassava4.1_001822m|PACid:17970187	cassava4.1_001822m	Pentatricopeptide repeat (PPR) superfamily protein	G
cassava4.1_001823m|PACid:17967130	cassava4.1_001823m	ARM repeat superfamily protein	GP
cassava4.1_001824m|PACid:17973218	cassava4.1_001824m	Root hair defective 3 GTP-binding protein (RHD3)	GP
cassava4.1_001827m|PACid:17990133	cassava4.1_001827m	Chaperone protein htpG family protein	GP
cassava4.1_001828m|PACid:17980720	cassava4.1_001828m	DHFS-FPGS homolog B	GP
cassava4.1_001836m|PACid:17963379	cassava4.1_001836m	OSBP(oxysterol binding protein)-related protein 1D	G
cassava4.1_001837m|PACid:17967726	cassava4.1_001837m	FTSH protease 10	GP
cassava4.1_001838m|PACid:17979723	cassava4.1_001838m	P-loop containing nucleoside triphosphate hydrolases superfamily protein	G
cassava4.1_001840m|PACid:17981623	cassava4.1_001840m	sucrose synthase 3	P
cassava4.1_001841m|PACid:17971217	cassava4.1_001841m	ribonucleotide reductase 1	GP
cassava4.1_001842m|PACid:17974241	cassava4.1_001842m	RINT-1 / TIP-1 family	No
cassava4.1_001845m|PACid:17976295	cassava4.1_001845m	ATP-dependent RNA helicase, mitochondrial, putative	No
cassava4.1_001848m|PACid:17962935	cassava4.1_001848m	phospholipase D alpha 1	P
cassava4.1_001849m|PACid:17973224	cassava4.1_001849m	Phosphoinositide phosphatase family protein	G
cassava4.1_001851m|PACid:17976279	cassava4.1_001851m	early-responsive to dehydration stress protein (ERD4)	No
cassava4.1_001856m|PACid:17994046	cassava4.1_001856m	ABC-2 type transporter family protein	No
cassava4.1_001861m|PACid:17964906	cassava4.1_001861m	inflorescence meristem receptor-like kinase 2	P
cassava4.1_001862m|PACid:17986888	cassava4.1_001862m	K+ efflux antiporter 3	No
cassava4.1_001865m|PACid:17981645	cassava4.1_001865m	exocyst complex component sec15B	GP
cassava4.1_001866m|PACid:17979762	cassava4.1_001866m	ubiquitin-specific protease 14	GP
cassava4.1_001869m|PACid:17967478	cassava4.1_001869m	methionine--tRNA ligase, putative / methionyl-tRNA synthetase, putative / MetRS, putative	P
cassava4.1_001871m|PACid:17965267	cassava4.1_001871m	sucrose synthase 4	P
cassava4.1_001873m|PACid:17987023	cassava4.1_001873m	Cyclopropane-fatty-acyl-phospholipid synthase	No
cassava4.1_001874m|PACid:17972883	cassava4.1_001874m	sucrose synthase 6	GP
cassava4.1_001875m|PACid:17980409	cassava4.1_001875m	ATPase, AAA-type, CDC48 protein	P
cassava4.1_001883m|PACid:17979900	cassava4.1_001883m	nucleolar protein gar2-related	No
cassava4.1_001888m|PACid:17974575	cassava4.1_001888m	Transducin/WD40 repeat-like superfamily protein	G
cassava4.1_001892m|PACid:17975547	cassava4.1_001892m	MIF4G domain-containing protein / MA3 domain-containing protein	GP
cassava4.1_001895m|PACid:17960756	cassava4.1_001895m	histone mono-ubiquitination 2	G
cassava4.1_001897m|PACid:17964389	cassava4.1_001897m	Transducin family protein / WD-40 repeat family protein	G
cassava4.1_001902m|PACid:17973568	cassava4.1_001902m	Inorganic H pyrophosphatase family protein	GP

cassava4.1_001904m|PACid:17976772	cassava4.1_001904m	cation/H+ exchanger 18	No
cassava4.1_001907m|PACid:17979170	cassava4.1_001907m	Golgi-localized GRIP domain-containing protein	No
cassava4.1_001913m|PACid:17960455	cassava4.1_001913m	Chaperone DnaJ-domain superfamily protein	GP
cassava4.1_001915m|PACid:17974683	cassava4.1_001915m	MIF4G domain-containing protein / MA3 domain-containing protein	GP
cassava4.1_001921m|PACid:17962790	cassava4.1_001921m	Chaperone protein htpG family protein	GP
cassava4.1_001922m|PACid:17980722	cassava4.1_001922m	Transducin/WD40 repeat-like superfamily protein	G
cassava4.1_001924m|PACid:17987714	cassava4.1_001924m	HEAT SHOCK PROTEIN 89.1	GP
cassava4.1_001926m|PACid:17974620	cassava4.1_001926m	splicing factor-related	GP
cassava4.1_001927m|PACid:17990352	cassava4.1_001927m	P-loop containing nucleoside triphosphate hydrolases superfamily protein	No
cassava4.1_001928m|PACid:17988571	cassava4.1_001928m	homeobox-7	No
cassava4.1_001929m|PACid:17989261	cassava4.1_001929m	kinesin 1	No
cassava4.1_001931m|PACid:17981759	cassava4.1_001931m	Protein kinase family protein with ARM repeat domain	GP
cassava4.1_001933m|PACid:17987803	cassava4.1_001933m	kinesin 3	No
cassava4.1_001937m|PACid:17966338	cassava4.1_001937m	VPS35 homolog A	GP
cassava4.1_001943m|PACid:17991093	cassava4.1_001943m	glutamine-tRNA ligase, putative / glutaminyl-tRNA synthetase, putative / GlnRS, putative	GP
cassava4.1_001949m|PACid:17987251	cassava4.1_001949m	protein kinase family protein	G
cassava4.1_001953m|PACid:17990168	cassava4.1_001953m	VPS35 homolog B	No
cassava4.1_001954m|PACid:17990482	cassava4.1_001954m	hydrolases, acting on acid anhydrides, in phosphorus-containing anhydrides;ATP-dependent helicases;nucleic acid binding	G
cassava4.1_001959m|PACid:17975459	cassava4.1_001959m	UDP-Glycosyltransferase superfamily protein	G
cassava4.1_001960m|PACid:17977832	cassava4.1_001960m	Glycosyl hydrolase family protein	GP
cassava4.1_001961m|PACid:17972836	cassava4.1_001961m	exocyst complex component sec15A	G
cassava4.1_001975m|PACid:17960189	cassava4.1_001975m	potassium transporter 2	G
cassava4.1_001979m|PACid:17980934	cassava4.1_001979m	auxin response factor 4	G
cassava4.1_001980m|PACid:17993316	cassava4.1_001980m	DEAD box RNA helicase (RH3)	GP
cassava4.1_001982m|PACid:17977630	cassava4.1_001982m	Translation elongation factor EFG/EF2 protein	GP
cassava4.1_001983m|PACid:17969592	cassava4.1_001983m	Copper amine oxidase family protein	GP
cassava4.1_001985m|PACid:17982788	cassava4.1_001985m	chloride channel B	No
cassava4.1_001986m|PACid:17969299	cassava4.1_001986m	GRAS family transcription factor	No
cassava4.1_001992m|PACid:17962747	cassava4.1_001992m	EXS (ERD1/XPR1/SYG1) family protein	No
cassava4.1_001994m|PACid:17992868	cassava4.1_001994m	P-loop containing nucleoside triphosphate hydrolases superfamily protein	No
cassava4.1_002004m|PACid:17967713	cassava4.1_002004m	Raffinose synthase family protein	G
cassava4.1_002006m|PACid:17992769	cassava4.1_002006m	sec34-like family protein	No
cassava4.1_002010m|PACid:17982494	cassava4.1_002010m	LEUNIG_homolog	GP
cassava4.1_002011m|PACid:17993006	cassava4.1_002011m	phosphatidylinositol-4-phosphate 5-kinase 1	No
cassava4.1_002012m|PACid:17967574	cassava4.1_002012m	S-adenosyl-L-methionine-dependent methyltransferases superfamily protein	No
cassava4.1_002015m|PACid:17976298	cassava4.1_002015m	Protein kinase superfamily protein	No
cassava4.1_002018m|PACid:17963606	cassava4.1_002018m	CwfJ-like family protein	G
cassava4.1_002024m|PACid:17978890	cassava4.1_002024m	glycosyltransferase family protein 47	G
cassava4.1_002025m|PACid:17989859	cassava4.1_002025m	Noc2p family	GP

cassava4.1_002030m|PACid:17959919	cassava4.1_002030m	Protein kinase protein with adenine nucleotide alpha hydrolases-like domain	No
cassava4.1_002037m|PACid:17964046	cassava4.1_002037m	subtilase family protein	No
cassava4.1_002039m|PACid:17986582	cassava4.1_002039m	chloride channel C	G
cassava4.1_002041m|PACid:17982290	cassava4.1_002041m	K+ uptake permease 6	No
cassava4.1_002042m|PACid:17961434	cassava4.1_002042m	SNF2 domain-containing protein / helicase domain-containing protein	No
cassava4.1_002044m|PACid:17962807	cassava4.1_002044m	Zincin-like metalloproteases family protein	GP
cassava4.1_002045m|PACid:17989834	cassava4.1_002045m	Zincin-like metalloproteases family protein	GP
cassava4.1_002048m|PACid:17972999	cassava4.1_002048m	Subtilase family protein	GP
cassava4.1_002054m|PACid:17981203	cassava4.1_002054m	Sec23/Sec24 protein transport family protein	GP
cassava4.1_002058m|PACid:17993008	cassava4.1_002058m	OSBP(oxysterol binding protein)-related protein 1C	No
cassava4.1_002066m|PACid:17985466	cassava4.1_002066m	SIT4 phosphatase-associated family protein	GP
cassava4.1_002069m|PACid:17976884	cassava4.1_002069m	Vps51/Vps67 family (components of vesicular transport) protein	No
cassava4.1_002070m|PACid:17980199	cassava4.1_002070m	seed imbibition 2	No
cassava4.1_002074m|PACid:17970965	cassava4.1_002074m	acid-amino acid ligases;ligases;ATP binding;ATP binding;ligases	No
cassava4.1_002075m|PACid:17993430	cassava4.1_002075m	acylaminoacyl-peptidase-related	GP
cassava4.1_002076m|PACid:17964566	cassava4.1_002076m	subtilase 1.3	No
cassava4.1_002077m|PACid:17962011	cassava4.1_002077m	beta-xylosidase 1	GP
cassava4.1_002078m|PACid:17978232	cassava4.1_002078m	ARM repeat superfamily protein	No
cassava4.1_002093m|PACid:17966228	cassava4.1_002093m	Neutral/alkaline non-lysosomal ceramidase	No
cassava4.1_002095m|PACid:17967218	cassava4.1_002095m	CRINKLY4 related 1	No
cassava4.1_002098m|PACid:17991504	cassava4.1_002098m	ERD (early-responsive to dehydration stress) family protein	No
cassava4.1_002100m|PACid:17992527	cassava4.1_002100m	Xanthine/uracil permease family protein	No
cassava4.1_002101m|PACid:17972532	cassava4.1_002101m	Ypt/Rab-GAP domain of gyp1p superfamily protein	No
cassava4.1_002108m|PACid:17967124	cassava4.1_002108m	ERD (early-responsive to dehydration stress) family protein	No
cassava4.1_002110m|PACid:17991949	cassava4.1_002110m	Neutral/alkaline non-lysosomal ceramidase	G
cassava4.1_002113m|PACid:17993876	cassava4.1_002113m	Helicase protein with RING/U-box domain	No
cassava4.1_002114m|PACid:17990312	cassava4.1_002114m	chloride channel F	G
cassava4.1_002115m|PACid:17986958	cassava4.1_002115m	Zinc finger (C3HC4-type RING finger) family protein	No
cassava4.1_002117m|PACid:17991018	cassava4.1_002117m	Subtilisin-like serine endopeptidase family protein	No
cassava4.1_002118m|PACid:17985900	cassava4.1_002118m	S-domain-2 5	No
cassava4.1_002121m|PACid:17988514	cassava4.1_002121m	Glycosyl hydrolase family protein	No
cassava4.1_002122m|PACid:17961884	cassava4.1_002122m	PAS domain-containing protein tyrosine kinase family protein	No
cassava4.1_002129m|PACid:17986393	cassava4.1_002129m	acetyl Co-enzyme a carboxylase carboxyltransferase alpha subunit	GP
cassava4.1_002131m|PACid:17971038	cassava4.1_002131m	homolog of xeroderma pigmentosum complementation group B 1	GP
cassava4.1_002132m|PACid:17978993	cassava4.1_002132m	metalloendopeptidases;zinc ion binding	G
cassava4.1_002139m|PACid:17970589	cassava4.1_002139m	mechanosensitive channel of small conductance-like 10	G
cassava4.1_002143m|PACid:17975391	cassava4.1_002143m	Protein kinase superfamily protein	GP
cassava4.1_002144m|PACid:17990453	cassava4.1_002144m	centromere/kinetochore protein, putative (ZW10)	G
cassava4.1_002146m|PACid:17971986	cassava4.1_002146m	chloride channel E	G

cassava4.1_002148m|PACid:17992338	cassava4.1_002148m		G
cassava4.1_002149m|PACid:17990356	cassava4.1_002149m	beta-xylosidase 2	No
cassava4.1_002152m|PACid:17962165	cassava4.1_002152m	Signal transduction histidine kinase, hybrid-type, ethylene sensor	No
cassava4.1_002153m|PACid:17965388	cassava4.1_002153m	acetyl-CoA synthetase	P
cassava4.1_002156m|PACid:17975202	cassava4.1_002156m	Cobalamin-independent synthase family protein	GP
cassava4.1_002159m|PACid:17977198	cassava4.1_002159m	OSBP(oxysterol binding protein)-related protein 2A	No
cassava4.1_002160m|PACid:17975327	cassava4.1_002160m	Inorganic H pyrophosphatase family protein	GP
cassava4.1_002165m|PACid:17989397	cassava4.1_002165m	Signal transduction histidine kinase, hybrid-type, ethylene sensor	G
cassava4.1_002166m|PACid:17976687	cassava4.1_002166m	homeodomain GLABROUS 2	No
cassava4.1_002168m|PACid:17969452	cassava4.1_002168m	Leucine-rich repeat protein kinase family protein	No
cassava4.1_002176m|PACid:17973565	cassava4.1_002176m	camelliol C synthase 1	G
cassava4.1_002177m|PACid:17961711	cassava4.1_002177m	Transducin/WD40 repeat-like superfamily protein	No
cassava4.1_002179m|PACid:17981431	cassava4.1_002179m	oligopeptide transporter 4	G
cassava4.1_002184m|PACid:17974123	cassava4.1_002184m	Tetratricopeptide repeat (TPR)-like superfamily protein	GP
cassava4.1_002186m|PACid:17984718	cassava4.1_002186m	transducin family protein / WD-40 repeat family protein	P
cassava4.1_002190m|PACid:17979488	cassava4.1_002190m	Sec23/Sec24 protein transport family protein	P
cassava4.1_002192m|PACid:17975149	cassava4.1_002192m	heteroglycan glucosidase 1	GP
cassava4.1_002201m|PACid:17961155	cassava4.1_002201m	SNF2 domain-containing protein / helicase domain-containing protein	G
cassava4.1_002204m|PACid:17968932	cassava4.1_002204m	Pentatricopeptide repeat (PPR) superfamily protein	No
cassava4.1_002207m|PACid:17969928	cassava4.1_002207m	S-locus lectin protein kinase family protein	GP
cassava4.1_002211m|PACid:17979969	cassava4.1_002211m	poly(A) polymerase 1	G
cassava4.1_002215m|PACid:17966091	cassava4.1_002215m	transcription activators	GP
cassava4.1_002217m|PACid:17978284	cassava4.1_002217m	Protein kinase superfamily protein	GP
cassava4.1_002219m|PACid:17986321	cassava4.1_002219m	RAD3-like DNA-binding helicase protein	G
cassava4.1_002221m|PACid:17979840	cassava4.1_002221m	Translation elongation factor EFG/EF2 protein	No
cassava4.1_002223m|PACid:17973996	cassava4.1_002223m	plastid transcriptionally active 2	No
cassava4.1_002236m|PACid:17975912	cassava4.1_002236m		GP
cassava4.1_002239m|PACid:17987827	cassava4.1_002239m	heat shock protein 70 (Hsp 70) family protein	No
cassava4.1_002240m|PACid:17985852	cassava4.1_002240m	CDC27 family protein	G
cassava4.1_002243m|PACid:17960295	cassava4.1_002243m	inter-alpha-trypsin inhibitor heavy chain-related	GP
cassava4.1_002244m|PACid:17988463	cassava4.1_002244m	prolyl oligopeptidase family protein	GP
cassava4.1_002245m|PACid:17978499	cassava4.1_002245m	P-loop containing nucleoside triphosphate hydrolases superfamily protein	No
cassava4.1_002247m|PACid:17966805	cassava4.1_002247m	ALBINA 1	No
cassava4.1_002248m|PACid:17973767	cassava4.1_002248m	ABC-2 type transporter family protein	No
cassava4.1_002249m|PACid:17990297	cassava4.1_002249m	seed imbibition 1	No
cassava4.1_002250m|PACid:17970890	cassava4.1_002250m	mRNAadenosine methylase	GP
cassava4.1_002253m|PACid:17960102	cassava4.1_002253m	SEC6	P
cassava4.1_002259m|PACid:17979353	cassava4.1_002259m	chromatin remodeling 1	No
cassava4.1_002260m|PACid:17972314	cassava4.1_002260m	Minichromosome maintenance (MCM2/3/5) family protein	No

cassava4.1_002264m|PACid:17990201	cassava4.1_002264m	DEA(D/H)-box RNA helicase family protein	G
cassava4.1_002266m|PACid:17987277	cassava4.1_002266m		G
cassava4.1_002267m|PACid:17968762	cassava4.1_002267m	DNA ligase 1	No
cassava4.1_002272m|PACid:17989134	cassava4.1_002272m	GAMMA-TUBULIN COMPLEX PROTEIN 4	P
cassava4.1_002275m|PACid:17977539	cassava4.1_002275m	LETM1-like protein	GP
cassava4.1_002284m|PACid:17961799	cassava4.1_002284m	Sec23/Sec24 protein transport family protein	G
cassava4.1_002287m|PACid:17989735	cassava4.1_002287m	ABC-2 type transporter family protein	GP
cassava4.1_002291m|PACid:17963711	cassava4.1_002291m		GP
cassava4.1_002292m|PACid:17965870	cassava4.1_002292m	ARM repeat superfamily protein	G
cassava4.1_002295m|PACid:17982669	cassava4.1_002295m	ubiquitin protein ligase 6	GP
cassava4.1_002297m|PACid:17990173	cassava4.1_002297m	Subtilisin-like serine endopeptidase family protein	GP
cassava4.1_002307m|PACid:17988755	cassava4.1_002307m	Transketolase	GP
cassava4.1_002312m|PACid:17973134	cassava4.1_002312m	Protein kinase protein with adenine nucleotide alpha hydrolases-like domain	No
cassava4.1_002314m|PACid:17990262	cassava4.1_002314m	oligopeptide transporter	No
cassava4.1_002315m|PACid:17978428	cassava4.1_002315m		G
cassava4.1_002319m|PACid:17969631	cassava4.1_002319m	fructose-2,6-bisphosphatase	GP
cassava4.1_002322m|PACid:17981812	cassava4.1_002322m	transducin family protein / WD-40 repeat family protein	No
cassava4.1_002323m|PACid:17973445	cassava4.1_002323m	Transducin/WD40 repeat-like superfamily protein	No
cassava4.1_002326m|PACid:17961867	cassava4.1_002326m	cullin 1	GP
cassava4.1_002327m|PACid:17980390	cassava4.1_002327m	MuDR family transposase	No
cassava4.1_002328m|PACid:17973483	cassava4.1_002328m	sec23/sec24 transport family protein	No
cassava4.1_002332m|PACid:17971459	cassava4.1_002332m	cullin 1	GP
cassava4.1_002333m|PACid:17966181	cassava4.1_002333m	RNA helicase family protein	GP
cassava4.1_002343m|PACid:17966677	cassava4.1_002343m	peroxin 5	G
cassava4.1_002347m|PACid:17972165	cassava4.1_002347m	staurosporin and temperature sensitive 3-like b	GP
cassava4.1_002352m|PACid:17980487	cassava4.1_002352m	Tetratricopeptide repeat (TPR)-like superfamily protein	G
cassava4.1_002356m|PACid:17964462	cassava4.1_002356m	NADH-ubiquinone dehydrogenase, mitochondrial, putative	GP
cassava4.1_002359m|PACid:17984596	cassava4.1_002359m	tonoplast monosaccharide transporter2	GP
cassava4.1_002361m|PACid:17978724	cassava4.1_002361m	AAA-type ATPase family protein	GP
cassava4.1_002363m|PACid:17968874	cassava4.1_002363m	4-hydroxy-3-methylbut-2-enyl diphosphate synthase	P
cassava4.1_002370m|PACid:17992186	cassava4.1_002370m	two-pore channel 1	GP
cassava4.1_002372m|PACid:17989400	cassava4.1_002372m	endoribonucleases	GP
cassava4.1_002375m|PACid:17983480	cassava4.1_002375m	Signal transduction histidine kinase, hybrid-type, ethylene sensor	G
cassava4.1_002380m|PACid:17986242	cassava4.1_002380m	methylcrotonyl-CoA carboxylase alpha chain, mitochondrial / 3-methylcrotonyl-CoA carboxylase 1 (MCCA)	GP
cassava4.1_002381m|PACid:17988759	cassava4.1_002381m	delta 1-pyrroline-5-carboxylate synthase 2	GP
cassava4.1_002382m|PACid:17987502	cassava4.1_002382m	glycosyl hydrolase family 81 protein	GP
cassava4.1_002395m|PACid:17975999	cassava4.1_002395m	RELA/SPOT homolog 3	G
cassava4.1_002397m|PACid:17986511	cassava4.1_002397m	Tetratricopeptide repeat (TPR)-like superfamily protein	No
cassava4.1_002402m|PACid:17961609	cassava4.1_002402m	Calcineurin-like metallo-phosphoesterase superfamily protein	No

cassava4.1_002403m|PACid:17968536	cassava4.1_002403m	lipases;hydrolases, acting on ester bonds	G
cassava4.1_002407m|PACid:17961232	cassava4.1_002407m	DNA GYRASE B2	GP
cassava4.1_002411m|PACid:17988817	cassava4.1_002411m	structural constituent of nuclear pore	GP
cassava4.1_002415m|PACid:17963489	cassava4.1_002415m	Copper amine oxidase family protein	GP
cassava4.1_002416m|PACid:17987180	cassava4.1_002416m	DNA repair and meiosis protein (Mre11)	G
cassava4.1_002423m|PACid:17970889	cassava4.1_002423m	carbon-nitrogen hydrolase family protein	P
cassava4.1_002425m|PACid:17985459	cassava4.1_002425m	Outer arm dynein light chain 1 protein	No
cassava4.1_002439m|PACid:17990359	cassava4.1_002439m	Protein kinase superfamily protein	No
cassava4.1_002440m|PACid:17974908	cassava4.1_002440m	cullin 3	No
cassava4.1_002441m|PACid:17986004	cassava4.1_002441m	cyclic nucleotide gated channel 5	G
cassava4.1_002445m|PACid:17963706	cassava4.1_002445m	RNA helicase family protein	GP
cassava4.1_002448m|PACid:17973586	cassava4.1_002448m	Prolyl oligopeptidase family protein	No
cassava4.1_002450m|PACid:17992434	cassava4.1_002450m	Phosphatidylinositol-4-phosphate 5-kinase family protein	No
cassava4.1_002452m|PACid:17982946	cassava4.1_002452m	CCCH-type zinc finger protein with ARM repeat domain	G
cassava4.1_002459m|PACid:17990578	cassava4.1_002459m	Tetratricopeptide repeat (TPR)-like superfamily protein	GP
cassava4.1_002460m|PACid:17976816	cassava4.1_002460m	Tetratricopeptide repeat (TPR)-like superfamily protein	No
cassava4.1_002461m|PACid:17989332	cassava4.1_002461m	peptide-N-glycanase 1	GP
cassava4.1_002463m|PACid:17985346	cassava4.1_002463m	Zincin-like metalloproteases family protein	No
cassava4.1_002469m|PACid:17959752	cassava4.1_002469m	general control non-repressible 4	GP
cassava4.1_002470m|PACid:17966775	cassava4.1_002470m	outer envelope protein of 80 kDa	P
cassava4.1_002471m|PACid:17965126	cassava4.1_002471m	ARM repeat superfamily protein	GP
cassava4.1_002477m|PACid:17965682	cassava4.1_002477m	Tetratricopeptide repeat (TPR)-like superfamily protein	G
cassava4.1_002481m|PACid:17991473	cassava4.1_002481m	STRUBBELIG-receptor family 8	G
cassava4.1_002483m|PACid:17978367	cassava4.1_002483m	RNA helicase family protein	No
cassava4.1_002486m|PACid:17965979	cassava4.1_002486m	Trimeric LpxA-like enzyme	GP
cassava4.1_002487m|PACid:17993240	cassava4.1_002487m	Glutamyl/glutaminyl-tRNA synthetase, class Ic	P
cassava4.1_002488m|PACid:17980493	cassava4.1_002488m	Minichromosome maintenance (MCM2/3/5) family protein	GP
cassava4.1_002490m|PACid:17993460	cassava4.1_002490m		No
cassava4.1_002493m|PACid:17984139	cassava4.1_002493m	nuclear poly(a) polymerase	No
cassava4.1_002495m|PACid:17979715	cassava4.1_002495m	multifunctional protein 2	No
cassava4.1_002497m|PACid:17969163	cassava4.1_002497m	diacylglycerol kinase1	No
cassava4.1_002498m|PACid:17989891	cassava4.1_002498m		GP
cassava4.1_002500m|PACid:17973181	cassava4.1_002500m	Transducin/WD40 repeat-like superfamily protein	No
cassava4.1_002508m|PACid:17968769	cassava4.1_002508m	Pleckstrin homology (PH) and lipid-binding START domains-containing protein	No
cassava4.1_002509m|PACid:17972608	cassava4.1_002509m	Early-responsive to dehydration stress protein (ERD4)	GP
cassava4.1_002511m|PACid:17971702	cassava4.1_002511m	Protein kinase superfamily protein	G
cassava4.1_002513m|PACid:17966987	cassava4.1_002513m	Tetratricopeptide repeat (TPR)-like superfamily protein	GP
cassava4.1_002519m|PACid:17977256	cassava4.1_002519m	Copper amine oxidase family protein	No
cassava4.1_002521m|PACid:17977952	cassava4.1_002521m	long-chain base (LCB) kinase 1	G

cassava4.1_002526m|PACid:17976564	cassava4.1_002526m	ABC-2 type transporter family protein	No
cassava4.1_002531m|PACid:17965555	cassava4.1_002531m	with no lysine (K) kinase 1	No
cassava4.1_002533m|PACid:17985977	cassava4.1_002533m	translation initiation factor 3B1	GP
cassava4.1_002535m|PACid:17988670	cassava4.1_002535m	Protein kinase superfamily protein	No
cassava4.1_002536m|PACid:17962167	cassava4.1_002536m	Deoxyxylulose-5-phosphate synthase	GP
cassava4.1_002537m|PACid:17987719	cassava4.1_002537m	UDP-Glycosyltransferase superfamily protein	No
cassava4.1_002541m|PACid:17987203	cassava4.1_002541m	cyclic nucleotide-gated cation channel 4	G
cassava4.1_002543m|PACid:17965327	cassava4.1_002543m	transcription activators	G
cassava4.1_002544m|PACid:17964480	cassava4.1_002544m	C2 domain-containing protein	G
cassava4.1_002547m|PACid:17985527	cassava4.1_002547m	non-intrinsic ABC protein 8	No
cassava4.1_002548m|PACid:17963086	cassava4.1_002548m	GPI transamidase component family protein / Gaa1-like family protein	No
cassava4.1_002551m|PACid:17975597	cassava4.1_002551m	cyclic nucleotide gated channel 1	No
cassava4.1_002552m|PACid:17973003	cassava4.1_002552m	Prolyl oligopeptidase family protein	GP
cassava4.1_002554m|PACid:17989241	cassava4.1_002554m	AMP-dependent synthetase and ligase family protein	GP
cassava4.1_002558m|PACid:17961126	cassava4.1_002558m	arginine decarboxylase 2	G
cassava4.1_002561m|PACid:17990323	cassava4.1_002561m	Minichromosome maintenance (MCM2/3/5) family protein	No
cassava4.1_002565m|PACid:17978481	cassava4.1_002565m	cyclic nucleotide gated channel 1	G
cassava4.1_002569m|PACid:17978177	cassava4.1_002569m	plant U-box 17	No
cassava4.1_002574m|PACid:17963682	cassava4.1_002574m	HCO3- transporter family	No
cassava4.1_002575m|PACid:17991186	cassava4.1_002575m	Leucine-rich repeat protein kinase family protein	G
cassava4.1_002581m|PACid:17968787	cassava4.1_002581m	P-loop containing nucleoside triphosphate hydrolases superfamily protein	No
cassava4.1_002586m|PACid:17972118	cassava4.1_002586m	FTSH protease 4	GP
cassava4.1_002588m|PACid:17973133	cassava4.1_002588m	STRUBBELIG-receptor family 6	G
cassava4.1_002589m|PACid:17970896	cassava4.1_002589m	general control non-repressible 3	GP
cassava4.1_002593m|PACid:17990115	cassava4.1_002593m	FtsH extracellular protease family	No
cassava4.1_002594m|PACid:17974503	cassava4.1_002594m	pentatricopeptide (PPR) repeat-containing protein	No
cassava4.1_002596m|PACid:17969567	cassava4.1_002596m	ThiF family protein	G
cassava4.1_002600m|PACid:17969524	cassava4.1_002600m	S-adenosyl-L-methionine-dependent methyltransferases superfamily protein	No
cassava4.1_002604m|PACid:17978282	cassava4.1_002604m	Leucine-rich repeat protein kinase family protein	No
cassava4.1_002610m|PACid:17992732	cassava4.1_002610m	no pollen germination related 1	No
cassava4.1_002612m|PACid:17971495	cassava4.1_002612m	Galactose oxidase/kelch repeat superfamily protein	No
cassava4.1_002616m|PACid:17965684	cassava4.1_002616m	Metallopeptidase M24 family protein	No
cassava4.1_002621m|PACid:17968600	cassava4.1_002621m	Leucine-rich repeat protein kinase family protein	No
cassava4.1_002623m|PACid:17982650	cassava4.1_002623m	HCO3- transporter family	G
cassava4.1_002624m|PACid:17967703	cassava4.1_002624m	Threonyl-tRNA synthetase	GP
cassava4.1_002625m|PACid:17968431	cassava4.1_002625m	ribophorin II (RPN2) family protein	GP
cassava4.1_002626m|PACid:17989988	cassava4.1_002626m	cyclic nucleotide gated channel 1	G
cassava4.1_002631m|PACid:17976961	cassava4.1_002631m	poly(ADP-ribose) polymerase 2	No
cassava4.1_002634m|PACid:17974097	cassava4.1_002634m	Pentatricopeptide repeat (PPR) superfamily protein	GP

cassava4.1_002637m|PACid:17984301	cassava4.1_002637m	Concanavalin A-like lectin protein kinase family protein	No
cassava4.1_002640m|PACid:17982991	cassava4.1_002640m	ATP binding microtubule motor family protein	G
cassava4.1_002642m|PACid:17959926	cassava4.1_002642m	Phox (PX) domain-containing protein	G
cassava4.1_002644m|PACid:17971874	cassava4.1_002644m	Protein kinase superfamily protein	G
cassava4.1_002648m|PACid:17989132	cassava4.1_002648m	general control non-repressible 5	GP
cassava4.1_002650m|PACid:17979453	cassava4.1_002650m	Tetratricopeptide repeat (TPR)-like superfamily protein	G
cassava4.1_002661m|PACid:17988771	cassava4.1_002661m	sulfate transporter 4.1	No
cassava4.1_002664m|PACid:17960871	cassava4.1_002664m	Protein kinase family protein	GP
cassava4.1_002669m|PACid:17964239	cassava4.1_002669m	Peptidase M28 family protein	GP
cassava4.1_002673m|PACid:17962140	cassava4.1_002673m	ARM repeat protein interacting with ABF2	No
cassava4.1_002677m|PACid:17967867	cassava4.1_002677m	FAD/NAD(P)-binding oxidoreductase family protein	No
cassava4.1_002679m|PACid:17985781	cassava4.1_002679m	ubiquitin-specific protease 19	G
cassava4.1_002682m|PACid:17965069	cassava4.1_002682m	Pentatricopeptide repeat (PPR-like) superfamily protein	No
cassava4.1_002686m|PACid:17966966	cassava4.1_002686m	Pentatricopeptide repeat (PPR-like) superfamily protein	No
cassava4.1_002689m|PACid:17962950	cassava4.1_002689m	Pentatricopeptide repeat (PPR) superfamily protein	No
cassava4.1_002692m|PACid:17978245	cassava4.1_002692m	RNA helicase family protein	No
cassava4.1_002697m|PACid:17975205	cassava4.1_002697m	Protein kinase family protein	G
cassava4.1_002703m|PACid:17993113	cassava4.1_002703m	FtsH extracellular protease family	GP
cassava4.1_002706m|PACid:17974818	cassava4.1_002706m	chloroplast heat shock protein 70-2	P
cassava4.1_002707m|PACid:17968518	cassava4.1_002707m	Cellulose-synthase-like C12	G
cassava4.1_002708m|PACid:17989996	cassava4.1_002708m	heat shock protein 90.1	GP
cassava4.1_002712m|PACid:17974118	cassava4.1_002712m	MA3 domain-containing protein	GP
cassava4.1_002715m|PACid:17964724	cassava4.1_002715m	root hair specific 10	No
cassava4.1_002716m|PACid:17962449	cassava4.1_002716m	chloroplast heat shock protein 70-2	GP
cassava4.1_002719m|PACid:17983987	cassava4.1_002719m	galacturonosyltransferase 3	No
cassava4.1_002723m|PACid:17973191	cassava4.1_002723m	glycoside hydrolase family 2 protein	GP
cassava4.1_002725m|PACid:17979188	cassava4.1_002725m	tetratricopeptide repeat (TPR)-containing protein	No
cassava4.1_002729m|PACid:17966084	cassava4.1_002729m	Pseudouridine synthase family protein	No
cassava4.1_002733m|PACid:17963577	cassava4.1_002733m	Transducin/WD40 repeat-like superfamily protein	G
cassava4.1_002736m|PACid:17973536	cassava4.1_002736m	nucleobase-ascorbate transporter 12	No
cassava4.1_002740m|PACid:17961526	cassava4.1_002740m	glutamine-fructose-6-phosphate transaminase (isomerizing)s;sugar binding;transaminases	No
cassava4.1_002751m|PACid:17970820	cassava4.1_002751m	YELLOW STRIPE like 7	G
cassava4.1_002761m|PACid:17972364	cassava4.1_002761m	long-chain acyl-CoA synthetase 7	GP
cassava4.1_002766m|PACid:17977701	cassava4.1_002766m	ARM repeat superfamily protein	No
cassava4.1_002768m|PACid:17966470	cassava4.1_002768m	FG-GAP repeat-containing protein	GP
cassava4.1_002769m|PACid:17966707	cassava4.1_002769m	Major Facilitator Superfamily with SPX (SYG1/Pho81/XPR1) domain-containing protein	No
cassava4.1_002772m|PACid:17970762	cassava4.1_002772m	auxin response factor 9	No
cassava4.1_002776m|PACid:17969944	cassava4.1_002776m	Plasma-membrane choline transporter family protein	No
cassava4.1_002777m|PACid:17985857	cassava4.1_002777m	Cyclophilin-like peptidyl-prolyl cis-trans isomerase family protein	G

cassava4.1_002782m|PACid:17965569	cassava4.1_002782m	Leucine-rich repeat protein kinase family protein	G
cassava4.1_002785m|PACid:17991403	cassava4.1_002785m	long chain acyl-CoA synthetase 9	P
cassava4.1_002787m|PACid:17976361	cassava4.1_002787m	protein arginine methyltransferase 7	No
cassava4.1_002789m|PACid:17969355	cassava4.1_002789m	RING/FYVE/PHD zinc finger superfamily protein	No
cassava4.1_002801m|PACid:17983746	cassava4.1_002801m	cleavage and polyadenylation specificity factor 73-I	GP
cassava4.1_002804m|PACid:17979145	cassava4.1_002804m	white-brown complex homolog protein 11	G
cassava4.1_002805m|PACid:17969971	cassava4.1_002805m	RNA helicase family protein	No
cassava4.1_002807m|PACid:17990992	cassava4.1_002807m	Plant protein of unknown function (DUF639)	No
cassava4.1_002808m|PACid:17966839	cassava4.1_002808m	ABC-2 type transporter family protein	GP
cassava4.1_002811m|PACid:17992660	cassava4.1_002811m	Pentatricopeptide repeat (PPR) superfamily protein	G
cassava4.1_002812m|PACid:17971352	cassava4.1_002812m	ABC-2 type transporter family protein	No
cassava4.1_002813m|PACid:17970250	cassava4.1_002813m	Protein kinase superfamily protein	No
cassava4.1_002815m|PACid:17979452	cassava4.1_002815m	DEAD/DEAH box RNA helicase family protein	No
cassava4.1_002818m|PACid:17959796	cassava4.1_002818m	Cellulose-synthase-like C5	G
cassava4.1_002820m|PACid:17965618	cassava4.1_002820m	long-chain acyl-CoA synthetase 7	G
cassava4.1_002823m|PACid:17973444	cassava4.1_002823m	Vps52 / Sac2 family	GP
cassava4.1_002833m|PACid:17980648	cassava4.1_002833m	P450 reductase 1	No
cassava4.1_002834m|PACid:17988549	cassava4.1_002834m	S-adenosyl-L-methionine-dependent methyltransferases superfamily protein	G
cassava4.1_002837m|PACid:17970515	cassava4.1_002837m	translocon at the inner envelope membrane of chloroplasts 110	GP
cassava4.1_002839m|PACid:17977847	cassava4.1_002839m	glycyl-tRNA synthetase / glycine--tRNA ligase	P
cassava4.1_002840m|PACid:17989088	cassava4.1_002840m	catalytics	GP
cassava4.1_002844m|PACid:17981530	cassava4.1_002844m	basic helix-loop-helix (bHLH) DNA-binding superfamily protein	G
cassava4.1_002847m|PACid:17984120	cassava4.1_002847m	hydroxyproline-rich glycoprotein family protein	G
cassava4.1_002853m|PACid:17967631	cassava4.1_002853m	microtubule-associated proteins 65-1	GP
cassava4.1_002855m|PACid:17988948	cassava4.1_002855m	acyl-CoA oxidase 2	G
cassava4.1_002857m|PACid:17984978	cassava4.1_002857m	Minichromosome maintenance (MCM2/3/5) family protein	GP
cassava4.1_002859m|PACid:17968841	cassava4.1_002859m	Vacuolar import/degradation, Vid27-related protein	GP
cassava4.1_002861m|PACid:17972813	cassava4.1_002861m	DNA binding;nucleotide binding;nucleic acid binding;DNA-directed DNA polymerases	GP
cassava4.1_002867m|PACid:17960655	cassava4.1_002867m	Concanavalin A-like lectin protein kinase family protein	GP
cassava4.1_002868m|PACid:17969201	cassava4.1_002868m	Protein kinase superfamily protein	G
cassava4.1_002870m|PACid:17982872	cassava4.1_002870m	tetratricopetide-repeat thioredoxin-like 1	No
cassava4.1_002871m|PACid:17966812	cassava4.1_002871m	sulfite reductase	GP
cassava4.1_002874m|PACid:17974722	cassava4.1_002874m	P-loop containing nucleoside triphosphate hydrolases superfamily protein	GP
cassava4.1_002886m|PACid:17988729	cassava4.1_002886m	Protein kinase superfamily protein	G
cassava4.1_002901m|PACid:17976238	cassava4.1_002901m	galacturonosyltransferase 1	No
cassava4.1_002907m|PACid:17975995	cassava4.1_002907m	phospholipid:diacylglycerol acyltransferase	No
cassava4.1_002911m|PACid:17989949	cassava4.1_002911m	DnaJ / Sec63 Brl domains-containing protein	No
cassava4.1_002918m|PACid:17983950	cassava4.1_002918m	Basic helix-loop-helix (bHLH) DNA-binding family protein	G
cassava4.1_002919m|PACid:17964600	cassava4.1_002919m	DEAD box RNA helicase (PRH75)	GP

cassava4.1_002928m|PACid:17965910	cassava4.1_002928m	Protein of unknown function (DUF3550/UPF0682)	G
cassava4.1_002931m|PACid:17989768	cassava4.1_002931m	Protein kinase superfamily protein	No
cassava4.1_002932m|PACid:17968125	cassava4.1_002932m	HCP-like superfamily protein	GP
cassava4.1_002933m|PACid:17959834	cassava4.1_002933m	SWAP (Suppressor-of-White-APricot)/surp RNA-binding domain-containing protein	GP
cassava4.1_002937m|PACid:17970371	cassava4.1_002937m	Galactosyltransferase family protein	GP
cassava4.1_002941m|PACid:17962069	cassava4.1_002941m	chloride channel D	G
cassava4.1_002946m|PACid:17972380	cassava4.1_002946m	NPK1-related protein kinase 3	No
cassava4.1_002948m|PACid:17982792	cassava4.1_002948m	Protein kinase superfamily protein	No
cassava4.1_002954m|PACid:17969110	cassava4.1_002954m	ABC-2 type transporter family protein	No
cassava4.1_002955m|PACid:17977396	cassava4.1_002955m	mitochondrial HSO70 2	GP
cassava4.1_002958m|PACid:17971243	cassava4.1_002958m	Nucleolar GTP-binding protein	P
cassava4.1_002962m|PACid:17973282	cassava4.1_002962m	SU(VAR)3-9 homolog 3	G
cassava4.1_002963m|PACid:17964550	cassava4.1_002963m	Leucine-rich repeat protein kinase family protein	No
cassava4.1_002964m|PACid:17981435	cassava4.1_002964m	mitochondrial HSO70 2	GP
cassava4.1_002966m|PACid:17985614	cassava4.1_002966m	acyl-CoA oxidase 3	GP
cassava4.1_002967m|PACid:17986404	cassava4.1_002967m	mRNA capping enzyme family protein	No
cassava4.1_002968m|PACid:17977615	cassava4.1_002968m	transmembrane kinase-like 1	No
cassava4.1_002969m|PACid:17974504	cassava4.1_002969m	auxin response factor 1	G
cassava4.1_002971m|PACid:17993164	cassava4.1_002971m	Plant neutral invertase family protein	No
cassava4.1_002972m|PACid:17960221	cassava4.1_002972m	signal peptide peptidase	GP
cassava4.1_002979m|PACid:17985343	cassava4.1_002979m	Leucine-rich repeat protein kinase family protein	No
cassava4.1_002980m|PACid:17967195	cassava4.1_002980m	auxin response factor 16	No
cassava4.1_002983m|PACid:17971191	cassava4.1_002983m	Protein kinase protein with adenine nucleotide alpha hydrolases-like domain	G
cassava4.1_002984m|PACid:17960125	cassava4.1_002984m	Leucine-rich repeat protein kinase family protein	G
cassava4.1_002986m|PACid:17993849	cassava4.1_002986m	Transducin/WD40 repeat-like superfamily protein	G
cassava4.1_002989m|PACid:17988509	cassava4.1_002989m	cryptochrome 1	No
cassava4.1_002996m|PACid:17961085	cassava4.1_002996m		G
cassava4.1_002998m|PACid:17986462	cassava4.1_002998m	transporter associated with antigen processing protein 1	G
cassava4.1_003000m|PACid:17978757	cassava4.1_003000m	FTSH protease 6	GP
cassava4.1_003001m|PACid:17979101	cassava4.1_003001m	Leucine-rich repeat protein kinase family protein	GP
cassava4.1_003003m|PACid:17977122	cassava4.1_003003m	pol-like 5	G
cassava4.1_003007m|PACid:17972878	cassava4.1_003007m	glucose-inhibited division family A protein	No
cassava4.1_003012m|PACid:17978878	cassava4.1_003012m	Cyclic nucleotide-regulated ion channel family protein	G
cassava4.1_003015m|PACid:17966016	cassava4.1_003015m	acyl-CoA binding protein 4	No
cassava4.1_003018m|PACid:17970188	cassava4.1_003018m	SU(VAR)3-9 homolog 3	No
cassava4.1_003019m|PACid:17979342	cassava4.1_003019m	Protein of unknown function (DUF726)	No
cassava4.1_003020m|PACid:17989952	cassava4.1_003020m	OBP3-responsive gene 1	G
cassava4.1_003024m|PACid:17966823	cassava4.1_003024m	topoisomerase 6 subunit B	No
cassava4.1_003025m|PACid:17988867	cassava4.1_003025m	S-adenosyl-L-methionine-dependent methyltransferases superfamily protein	No

cassava4.1_003026m|PACid:17987031	cassava4.1_003026m	Cellulose-synthase-like C6	No
cassava4.1_003043m|PACid:17987189	cassava4.1_003043m	P-loop containing nucleoside triphosphate hydrolases superfamily protein	GP
cassava4.1_003050m|PACid:17964291	cassava4.1_003050m	RNI-like superfamily protein	G
cassava4.1_003053m|PACid:17983036	cassava4.1_003053m	Small GTP-binding protein	G
cassava4.1_003058m|PACid:17971079	cassava4.1_003058m	elongation factor family protein	No
cassava4.1_003059m|PACid:17976228	cassava4.1_003059m	Protein kinase superfamily protein	No
cassava4.1_003067m|PACid:17990058	cassava4.1_003067m	rhamnose biosynthesis 1	GP
cassava4.1_003069m|PACid:17964466	cassava4.1_003069m	Zn-dependent exopeptidases superfamily protein	G
cassava4.1_003071m|PACid:17992321	cassava4.1_003071m		GP
cassava4.1_003073m|PACid:17974045	cassava4.1_003073m	Leucine-rich repeat protein kinase family protein	No
cassava4.1_003074m|PACid:17984103	cassava4.1_003074m	ABA Overly-Sensitive 5	G
cassava4.1_003080m|PACid:17985896	cassava4.1_003080m	TBP-associated factor 5	GP
cassava4.1_003084m|PACid:17965965	cassava4.1_003084m	cationic amino acid transporter 4	G
cassava4.1_003088m|PACid:17986560	cassava4.1_003088m	MAK10 homologue	No
cassava4.1_003090m|PACid:17980165	cassava4.1_003090m	alpha-L-arabinofuranosidase 1	GP
cassava4.1_003091m|PACid:17987085	cassava4.1_003091m	Pentatricopeptide repeat (PPR) superfamily protein	G
cassava4.1_003096m|PACid:17985753	cassava4.1_003096m	Arginyl-tRNA synthetase, class Ic	P
cassava4.1_003100m|PACid:17982129	cassava4.1_003100m	replication protein A 1A	No
cassava4.1_003101m|PACid:17977677	cassava4.1_003101m	Galactosyltransferase family protein	G
cassava4.1_003102m|PACid:17983898	cassava4.1_003102m	receptor lectin kinase	G
cassava4.1_003110m|PACid:17983792	cassava4.1_003110m	GPI transamidase component Gpi16 subunit family protein	GP
cassava4.1_003114m|PACid:17991697	cassava4.1_003114m	Polymerase/histidinol phosphatase-like	G
cassava4.1_003115m|PACid:17985918	cassava4.1_003115m	proline extensin-like receptor kinase 1	GP
cassava4.1_003120m|PACid:17964875	cassava4.1_003120m	Phototropic-responsive NPH3 family protein	G
cassava4.1_003125m|PACid:17980309	cassava4.1_003125m	plant U-box 13	G
cassava4.1_003128m|PACid:17984127	cassava4.1_003128m	ATP-dependent peptidases;nucleotide binding;serine-type endopeptidases;DNA helicases;ATP binding	GP
cassava4.1_003132m|PACid:17992959	cassava4.1_003132m	zeaxanthin epoxidase (ZEP) (ABA1)	GP
cassava4.1_003135m|PACid:17962704	cassava4.1_003135m	histone deacetylase 5	GP
cassava4.1_003136m|PACid:17978864	cassava4.1_003136m	acyl-CoA oxidase 1	GP
cassava4.1_003139m|PACid:17991696	cassava4.1_003139m	NOT2 / NOT3 / NOT5 family	No
cassava4.1_003144m|PACid:17970871	cassava4.1_003144m	Heat shock protein 70 (Hsp 70) family protein	GP
cassava4.1_003145m|PACid:17976447	cassava4.1_003145m	acyl-CoA oxidase 1	GP
cassava4.1_003148m|PACid:17963662	cassava4.1_003148m	YELLOW STRIPE like 1	No
cassava4.1_003150m|PACid:17978281	cassava4.1_003150m	SRP72 RNA-binding domain	P
cassava4.1_003153m|PACid:17966529	cassava4.1_003153m	armadillo repeat only 2	No
cassava4.1_003161m|PACid:17983213	cassava4.1_003161m	Leucine-rich repeat protein kinase family protein	No
cassava4.1_003164m|PACid:17989554	cassava4.1_003164m	Cellulose-synthase-like C4	G
cassava4.1_003171m|PACid:17962491	cassava4.1_003171m	Pentatricopeptide repeat (PPR) superfamily protein	G
cassava4.1_003174m|PACid:17986687	cassava4.1_003174m	Phosphoribulokinase / Uridine kinase family	No

cassava4.1_003175m|PACid:17988425	cassava4.1_003175m	RNA-binding KH domain-containing protein	No
cassava4.1_003177m|PACid:17977258	cassava4.1_003177m	Leucine-rich repeat protein kinase family protein	G
cassava4.1_003179m|PACid:17961937	cassava4.1_003179m	GTP1/OBG family protein	G
cassava4.1_003182m|PACid:17984618	cassava4.1_003182m	long-chain acyl-CoA synthetase 2	No
cassava4.1_003184m|PACid:17990803	cassava4.1_003184m	plus-3 domain-containing protein	G
cassava4.1_003185m|PACid:17964650	cassava4.1_003185m	AMP-dependent synthetase and ligase family protein	GP
cassava4.1_003190m|PACid:17984282	cassava4.1_003190m	Leucine-rich repeat protein kinase family protein	No
cassava4.1_003193m|PACid:17978024	cassava4.1_003193m	receptor-like kinase 1	G
cassava4.1_003196m|PACid:17986691	cassava4.1_003196m	NagB/RpiA/CoA transferase-like superfamily protein	GP
cassava4.1_003200m|PACid:17962804	cassava4.1_003200m	Phosphoribulokinase / Uridine kinase family	G
cassava4.1_003201m|PACid:17963970	cassava4.1_003201m	Glycosyl hydrolase family protein	G
cassava4.1_003202m|PACid:17967519	cassava4.1_003202m	Concanavalin A-like lectin protein kinase family protein	No
cassava4.1_003204m|PACid:17989324	cassava4.1_003204m	Ankyrin repeat family protein	G
cassava4.1_003205m|PACid:17986974	cassava4.1_003205m	Actin binding Calponin homology (CH) domain-containing protein	G
cassava4.1_003207m|PACid:17969460	cassava4.1_003207m	Leucine-rich repeat protein kinase family protein	G
cassava4.1_003211m|PACid:17965037	cassava4.1_003211m	chlorsulfuron/imidazolinone resistant 1	GP
cassava4.1_003213m|PACid:17992490	cassava4.1_003213m	Mono-/di-acylglycerol lipase, N-terminal;Lipase, class 3	No
cassava4.1_003215m|PACid:17983524	cassava4.1_003215m	RabGAP/TBC domain-containing protein	GP
cassava4.1_003221m|PACid:17974621	cassava4.1_003221m	Endomembrane protein 70 protein family	GP
cassava4.1_003222m|PACid:17991021	cassava4.1_003222m	Endomembrane protein 70 protein family	GP
cassava4.1_003223m|PACid:17971827	cassava4.1_003223m	slufate transporter 2;1	No
cassava4.1_003226m|PACid:17965690	cassava4.1_003226m	NPK1-related protein kinase 1	No
cassava4.1_003227m|PACid:17982846	cassava4.1_003227m	P-loop containing nucleoside triphosphate hydrolases superfamily protein	No
cassava4.1_003228m|PACid:17989000	cassava4.1_003228m	slufate transporter 2;1	G
cassava4.1_003229m|PACid:17982986	cassava4.1_003229m	sulfate transporter 3;4	No
cassava4.1_003230m|PACid:17986211	cassava4.1_003230m	sulfate transporter 3;1	G
cassava4.1_003235m|PACid:17979659	cassava4.1_003235m	high chlorophyll fluorescent 107	No
cassava4.1_003238m|PACid:17978678	cassava4.1_003238m	galacturonosyltransferase 4	GP
cassava4.1_003239m|PACid:17965161	cassava4.1_003239m	response regulator 2	No
cassava4.1_003240m|PACid:17981189	cassava4.1_003240m	heat shock protein 70B	GP
cassava4.1_003242m|PACid:17986440	cassava4.1_003242m	chloroplast sensor kinase	No
cassava4.1_003248m|PACid:17991532	cassava4.1_003248m		G
cassava4.1_003249m|PACid:17988241	cassava4.1_003249m	YELLOW STRIPE like 3	No
cassava4.1_003258m|PACid:17969080	cassava4.1_003258m	phosphoinositide 4-kinase gamma 7	No
cassava4.1_003261m|PACid:17992501	cassava4.1_003261m	MSCS-like 2	No
cassava4.1_003265m|PACid:17984914	cassava4.1_003265m	Leucine-rich repeat protein kinase family protein	GP
cassava4.1_003267m|PACid:17992846	cassava4.1_003267m	cysteine-rich RLK (RECEPTOR-like protein kinase) 3	No
cassava4.1_003269m|PACid:17983678	cassava4.1_003269m	EIN3-binding F box protein 1	G
cassava4.1_003274m|PACid:17984435	cassava4.1_003274m	evolutionarily conserved C-terminal region 5	GP

cassava4.1_003276m|PACid:17981160	cassava4.1_003276m	MIRO-related GTP-ase 1	GP
cassava4.1_003278m|PACid:17990275	cassava4.1_003278m	leucine-rich repeat/extensin 2	P
cassava4.1_003282m|PACid:17985974	cassava4.1_003282m	epsin N-terminal homology (ENTH) domain-containing protein / clathrin assembly protein-related	G
cassava4.1_003283m|PACid:17979934	cassava4.1_003283m	Leucine-rich repeat protein kinase family protein	No
cassava4.1_003287m|PACid:17982463	cassava4.1_003287m	Ankyrin repeat family protein	G
cassava4.1_003290m|PACid:17978370	cassava4.1_003290m	Purple acid phosphatases superfamily protein	GP
cassava4.1_003293m|PACid:17970368	cassava4.1_003293m	sulfate transporter 91	No
cassava4.1_003298m|PACid:17971159	cassava4.1_003298m	Protein kinase superfamily protein	G
cassava4.1_003299m|PACid:17967481	cassava4.1_003299m	Beta-glucosidase, GBA2 type family protein	No
cassava4.1_003300m|PACid:17977054	cassava4.1_003300m		No
cassava4.1_003303m|PACid:17980842	cassava4.1_003303m	loricrin-related	G
cassava4.1_003309m|PACid:17966348	cassava4.1_003309m	Histidine kinase-, DNA gyrase B-, and HSP90-like ATPase family protein	No
cassava4.1_003310m|PACid:17960535	cassava4.1_003310m	flavodoxin family protein / radical SAM domain-containing protein	GP
cassava4.1_003312m|PACid:17974197	cassava4.1_003312m	tetratricopeptide repeat (TPR)-containing protein	G
cassava4.1_003314m|PACid:17968003	cassava4.1_003314m	DnaJ domain ;Myb-like DNA-binding domain	GP
cassava4.1_003315m|PACid:17978335	cassava4.1_003315m	Myotubularin-like phosphatases II superfamily	G
cassava4.1_003319m|PACid:17982599	cassava4.1_003319m	DNA-binding protein, putative	GP
cassava4.1_003321m|PACid:17975208	cassava4.1_003321m	cationic amino acid transporter 2	No
cassava4.1_003328m|PACid:17989501	cassava4.1_003328m	heat shock cognate protein 70-1	GP
cassava4.1_003331m|PACid:17988720	cassava4.1_003331m	heat shock cognate protein 70-1	GP
cassava4.1_003339m|PACid:17978298	cassava4.1_003339m	L-aspartate oxidase	G
cassava4.1_003343m|PACid:17989500	cassava4.1_003343m	heat shock protein 70	GP
cassava4.1_003350m|PACid:17991974	cassava4.1_003350m	thiaminC	No
cassava4.1_003354m|PACid:17985305	cassava4.1_003354m	VEFS-Box of polycomb protein	GP
cassava4.1_003358m|PACid:17968643	cassava4.1_003358m	plant glycogenin-like starch initiation protein 2	GP
cassava4.1_003361m|PACid:17963573	cassava4.1_003361m	SH3 domain-containing protein	No
cassava4.1_003365m|PACid:17991165	cassava4.1_003365m	leucine-rich repeat transmembrane protein kinase family protein	No
cassava4.1_003367m|PACid:17970425	cassava4.1_003367m	Auxin efflux carrier family protein	G
cassava4.1_003368m|PACid:17988044	cassava4.1_003368m	exocyst subunit exo70 family protein A1	No
cassava4.1_003369m|PACid:17961246	cassava4.1_003369m	Tetratricopeptide repeat (TPR)-like superfamily protein	No
cassava4.1_003372m|PACid:17967975	cassava4.1_003372m	sulfate transporter 1;3	No
cassava4.1_003373m|PACid:17969601	cassava4.1_003373m	Concanavalin A-like lectin protein kinase family protein	GP
cassava4.1_003376m|PACid:17985379	cassava4.1_003376m	poly(A) binding protein 4	GP
cassava4.1_003384m|PACid:17961758	cassava4.1_003384m	cryptochrome 2	G
cassava4.1_003385m|PACid:17974958	cassava4.1_003385m	Pentatricopeptide repeat (PPR) superfamily protein	No
cassava4.1_003387m|PACid:17987630	cassava4.1_003387m	ATP binding;valine-tRNA ligases;aminoacyl-tRNA ligases;nucleotide binding	No
cassava4.1_003388m|PACid:17972650	cassava4.1_003388m	ARM repeat superfamily protein	No
cassava4.1_003391m|PACid:17993226	cassava4.1_003391m	F-box family protein	No
cassava4.1_003395m|PACid:17984431	cassava4.1_003395m	pentatricopeptide (PPR) repeat-containing protein	GP

cassava4.1_003396m|PACid:17980047	cassava4.1_003396m	poly(A) binding protein 8	GP
cassava4.1_003400m|PACid:17985746	cassava4.1_003400m	Endomembrane protein 70 protein family	GP
cassava4.1_003404m|PACid:17962135	cassava4.1_003404m	Glycosyl hydrolase superfamily protein	GP
cassava4.1_003405m|PACid:17973171	cassava4.1_003405m	NADP-malic enzyme 4	P
cassava4.1_003407m|PACid:17974217	cassava4.1_003407m	Tetratricopeptide repeat (TPR)-like superfamily protein	G
cassava4.1_003410m|PACid:17983027	cassava4.1_003410m	Pentatricopeptide repeat (PPR-like) superfamily protein	No
cassava4.1_003414m|PACid:17979674	cassava4.1_003414m	COBRA-like protein-7 precursor	GP
cassava4.1_003417m|PACid:17975937	cassava4.1_003417m	exocyst subunit exo70 family protein F1	GP
cassava4.1_003420m|PACid:17987462	cassava4.1_003420m	cell division protein ftsH, putative	G
cassava4.1_003421m|PACid:17972321	cassava4.1_003421m	F-box/RNI-like superfamily protein	G
cassava4.1_003422m|PACid:17961281	cassava4.1_003422m	Leucine-rich repeat protein kinase family protein	No
cassava4.1_003425m|PACid:17977583	cassava4.1_003425m	2-isopropylmalate synthase 1	GP
cassava4.1_003428m|PACid:17968012	cassava4.1_003428m	high mobility group	No
cassava4.1_003429m|PACid:17968199	cassava4.1_003429m	Asparagine synthase family protein	No
cassava4.1_003433m|PACid:17984135	cassava4.1_003433m	protein kinase family protein / peptidoglycan-binding LysM domain-containing protein	G
cassava4.1_003434m|PACid:17977345	cassava4.1_003434m	Vacuolar import/degradation, Vid27-related protein	No
cassava4.1_003438m|PACid:17959740	cassava4.1_003438m	Ankyrin repeat family protein with DHHC zinc finger domain	No
cassava4.1_003442m|PACid:17973638	cassava4.1_003442m	ABC-2 type transporter family protein	No
cassava4.1_003443m|PACid:17986011	cassava4.1_003443m	Endomembrane protein 70 protein family	No
cassava4.1_003444m|PACid:17981455	cassava4.1_003444m	ethylene response sensor 1	G
cassava4.1_003447m|PACid:17993305	cassava4.1_003447m	Major facilitator superfamily protein	G
cassava4.1_003449m|PACid:17976032	cassava4.1_003449m	transmembrane nine 7	G
cassava4.1_003454m|PACid:17987498	cassava4.1_003454m	NagB/RpiA/CoA transferase-like superfamily protein	No
cassava4.1_003456m|PACid:17964392	cassava4.1_003456m	Transducin/WD40 repeat-like superfamily protein	G
cassava4.1_003457m|PACid:17992666	cassava4.1_003457m	with no lysine (K) kinase 6	No
cassava4.1_003467m|PACid:17984463	cassava4.1_003467m	Eukaryotic aspartyl protease family protein	G
cassava4.1_003471m|PACid:17985549	cassava4.1_003471m	phosphoglucomutase	P
cassava4.1_003473m|PACid:17963751	cassava4.1_003473m	Putative endonuclease or glycosyl hydrolase	No
cassava4.1_003476m|PACid:17973009	cassava4.1_003476m	transmembrane nine 7	GP
cassava4.1_003478m|PACid:17966397	cassava4.1_003478m	succinate dehydrogenase 1-1	GP
cassava4.1_003479m|PACid:17964143	cassava4.1_003479m	10-formyltetrahydrofolate synthetase	GP
cassava4.1_003480m|PACid:17976093	cassava4.1_003480m	Fatty acid hydroxylase superfamily	G
cassava4.1_003485m|PACid:17981389	cassava4.1_003485m	S-adenosyl-L-methionine-dependent methyltransferases superfamily protein	No
cassava4.1_003490m|PACid:17992565	cassava4.1_003490m	Dihydrolipoamide acetyltransferase, long form protein	GP
cassava4.1_003494m|PACid:17973215	cassava4.1_003494m	Amino acid dehydrogenase family protein	G
cassava4.1_003496m|PACid:17992090	cassava4.1_003496m	ATP-binding cassette 14	No
cassava4.1_003505m|PACid:17977129	cassava4.1_003505m	ARP protein (REF)	No
cassava4.1_003509m|PACid:17968413	cassava4.1_003509m	Tetratricopeptide repeat (TPR)-like superfamily protein	No
cassava4.1_003514m|PACid:17977680	cassava4.1_003514m	catalytics	GP

cassava4.1_003517m|PACid:17966733	cassava4.1_003517m	Leucine-rich repeat protein kinase family protein	GP
cassava4.1_003519m|PACid:17979361	cassava4.1_003519m	succinate dehydrogenase 1-1	P
cassava4.1_003521m|PACid:17966169	cassava4.1_003521m	alpha dioxygenase	G
cassava4.1_003527m|PACid:17973964	cassava4.1_003527m	P-loop containing nucleoside triphosphate hydrolases superfamily protein	No
cassava4.1_003530m|PACid:17978096	cassava4.1_003530m	Leucine-rich repeat protein kinase family protein	GP
cassava4.1_003531m|PACid:17976569	cassava4.1_003531m	Phototropic-responsive NPH3 family protein	GP
cassava4.1_003535m|PACid:17967120	cassava4.1_003535m	O-fucosyltransferase family protein	No
cassava4.1_003540m|PACid:17976062	cassava4.1_003540m	cation calcium exchanger 4	No
cassava4.1_003543m|PACid:17981834	cassava4.1_003543m	auxin F-box protein 5	No
cassava4.1_003546m|PACid:17970041	cassava4.1_003546m	receptor like protein 4	No
cassava4.1_003549m|PACid:17982623	cassava4.1_003549m	Tetratricopeptide repeat (TPR)-like superfamily protein	No
cassava4.1_003557m|PACid:17979751	cassava4.1_003557m	vaculolar sorting receptor 3	GP
cassava4.1_003558m|PACid:17978209	cassava4.1_003558m	Protein kinase superfamily protein	No
cassava4.1_003559m|PACid:17971284	cassava4.1_003559m	GTP-binding family protein	No
cassava4.1_003563m|PACid:17991713	cassava4.1_003563m	HAD-superfamily hydrolase, subfamily IG, 5\'-nucleotidase	GP
cassava4.1_003564m|PACid:17973871	cassava4.1_003564m	Pentatricopeptide repeat (PPR) superfamily protein	G
cassava4.1_003565m|PACid:17970503	cassava4.1_003565m	FAD-dependent oxidoreductase family protein	No
cassava4.1_003566m|PACid:17993219	cassava4.1_003566m	glucose-6-phosphate dehydrogenase 4	GP
cassava4.1_003570m|PACid:17985024	cassava4.1_003570m	Leucine-rich repeat protein kinase family protein	G
cassava4.1_003576m|PACid:17989073	cassava4.1_003576m	Phototropic-responsive NPH3 family protein	No
cassava4.1_003577m|PACid:17970494	cassava4.1_003577m	plant U-box 14	GP
cassava4.1_003579m|PACid:17982960	cassava4.1_003579m	CDPK-related kinase	No
cassava4.1_003580m|PACid:17970316	cassava4.1_003580m	potassium channel in Arabidopsis thaliana 3	No
cassava4.1_003582m|PACid:17969493	cassava4.1_003582m	Glycosyl hydrolase family protein	GP
cassava4.1_003584m|PACid:17979022	cassava4.1_003584m		GP
cassava4.1_003585m|PACid:17976033	cassava4.1_003585m	Transcription initiation factor TFIID subunit A	G
cassava4.1_003586m|PACid:17993903	cassava4.1_003586m	O-fucosyltransferase family protein	G
cassava4.1_003595m|PACid:17978500	cassava4.1_003595m	NAD(P)-binding Rossmann-fold superfamily protein	No
cassava4.1_003599m|PACid:17971981	cassava4.1_003599m	alpha-mannosidase 3	No
cassava4.1_003601m|PACid:17963421	cassava4.1_003601m	Phototropic-responsive NPH3 family protein	No
cassava4.1_003602m|PACid:17980183	cassava4.1_003602m	KU70 homolog	G
cassava4.1_003603m|PACid:17963194	cassava4.1_003603m	DHHC-type zinc finger family protein	No
cassava4.1_003609m|PACid:17963875	cassava4.1_003609m	Phototropic-responsive NPH3 family protein	No
cassava4.1_003610m|PACid:17963245	cassava4.1_003610m	S-adenosyl-L-methionine-dependent methyltransferases superfamily protein	No
cassava4.1_003615m|PACid:17988719	cassava4.1_003615m	NAD-dependent malic enzyme 1	GP
cassava4.1_003619m|PACid:17979244	cassava4.1_003619m	Methylthiotransferase	No
cassava4.1_003621m|PACid:17983604	cassava4.1_003621m	protein kinase family protein	G
cassava4.1_003622m|PACid:17968329	cassava4.1_003622m	lysyl-tRNA synthetase 1	No
cassava4.1_003625m|PACid:17977451	cassava4.1_003625m	clathrin adaptor complexes medium subunit family protein	G

cassava4.1_003629m|PACid:17961462	cassava4.1_003629m	flavin-binding, kelch repeat, f box 1	No
cassava4.1_003634m|PACid:17981720	cassava4.1_003634m	exocyst complex component sec5	No
cassava4.1_003637m|PACid:17979563	cassava4.1_003637m	Leucine-rich repeat protein kinase family protein	No
cassava4.1_003639m|PACid:17981642	cassava4.1_003639m	DEGP protease 2	No
cassava4.1_003643m|PACid:17992851	cassava4.1_003643m	NSP-interacting kinase 3	No
cassava4.1_003645m|PACid:17963026	cassava4.1_003645m	Ankyrin repeat family protein	G
cassava4.1_003649m|PACid:17965815	cassava4.1_003649m	vacuolar sorting receptor homolog 1	No
cassava4.1_003654m|PACid:17961393	cassava4.1_003654m	microtubule-associated proteins 70-2	G
cassava4.1_003658m|PACid:17967822	cassava4.1_003658m	translocon at the outer envelope membrane of chloroplasts 75-III	GP
cassava4.1_003659m|PACid:17991772	cassava4.1_003659m	alkaline/neutral invertase	G
cassava4.1_003660m|PACid:17988615	cassava4.1_003660m	somatic embryogenesis receptor-like kinase 1	GP
cassava4.1_003663m|PACid:17970701	cassava4.1_003663m	pleckstrin homology (PH) domain-containing protein	P
cassava4.1_003664m|PACid:17989579	cassava4.1_003664m	NSP-interacting kinase 1	No
cassava4.1_003668m|PACid:17991754	cassava4.1_003668m	cyclophilin71	No
cassava4.1_003671m|PACid:17971878	cassava4.1_003671m	ubiquitin-conjugating enzyme 25	G
cassava4.1_003672m|PACid:17962786	cassava4.1_003672m	RPA70-kDa subunit B	No
cassava4.1_003674m|PACid:17962266	cassava4.1_003674m	S-adenosyl-L-methionine-dependent methyltransferases superfamily protein	GP
cassava4.1_003676m|PACid:17974360	cassava4.1_003676m	vacuolar ATP synthase subunit A	P
cassava4.1_003677m|PACid:17968299	cassava4.1_003677m	vacuolar ATP synthase subunit A	GP
cassava4.1_003680m|PACid:17965113	cassava4.1_003680m	cleavage and polyadenylation specificity factor 73 kDa subunit-II	GP
cassava4.1_003682m|PACid:17991622	cassava4.1_003682m	P-loop containing nucleoside triphosphate hydrolases superfamily protein	G
cassava4.1_003684m|PACid:17994000	cassava4.1_003684m	Phototropic-responsive NPH3 family protein	GP
cassava4.1_003685m|PACid:17981508	cassava4.1_003685m	CTP synthase family protein	GP
cassava4.1_003687m|PACid:17992828	cassava4.1_003687m	cysteine-rich RLK (RECEPTOR-like protein kinase) 2	G
cassava4.1_003688m|PACid:17968945	cassava4.1_003688m	NSP-interacting kinase 2	No
cassava4.1_003695m|PACid:17975856	cassava4.1_003695m	O-fucosyltransferase family protein	No
cassava4.1_003698m|PACid:17977170	cassava4.1_003698m	glycosyl hydrolase 9C2	GP
cassava4.1_003699m|PACid:17986032	cassava4.1_003699m	Sec14p-like phosphatidylinositol transfer family protein	No
cassava4.1_003700m|PACid:17982169	cassava4.1_003700m	BAK1-interacting receptor-like kinase 1	GP
cassava4.1_003701m|PACid:17976954	cassava4.1_003701m	Ethylene insensitive 3 family protein	G
cassava4.1_003705m|PACid:17992813	cassava4.1_003705m	glycosyl hydrolase 9A1	GP
cassava4.1_003707m|PACid:17963319	cassava4.1_003707m	DNA polymerase alpha 2	No
cassava4.1_003712m|PACid:17983042	cassava4.1_003712m	Six-hairpin glycosidases superfamily protein	No
cassava4.1_003715m|PACid:17994052	cassava4.1_003715m	P-loop containing nucleoside triphosphate hydrolases superfamily protein	GP
cassava4.1_003717m|PACid:17986407	cassava4.1_003717m	O-fucosyltransferase family protein	No
cassava4.1_003719m|PACid:17992220	cassava4.1_003719m	Phototropic-responsive NPH3 family protein	No
cassava4.1_003724m|PACid:17971628	cassava4.1_003724m	Fatty acid hydroxylase superfamily	G
cassava4.1_003726m|PACid:17962707	cassava4.1_003726m	Di-glucose binding protein with Leucine-rich repeat domain	G
cassava4.1_003730m|PACid:17988132	cassava4.1_003730m	RH39	G

cassava4.1_003733m|PACid:17982336	cassava4.1_003733m	phosphoglucomutase, putative / glucose phosphomutase, putative	GP
cassava4.1_003736m|PACid:17978388	cassava4.1_003736m	QUASIMODO2 LIKE 2	No
cassava4.1_003737m|PACid:17978386	cassava4.1_003737m	Tetratricopeptide repeat (TPR)-like superfamily protein	G
cassava4.1_003741m|PACid:17987513	cassava4.1_003741m	RGA-like 1	G
cassava4.1_003744m|PACid:17973997	cassava4.1_003744m	Galactose oxidase/kelch repeat superfamily protein	No
cassava4.1_003746m|PACid:17961423	cassava4.1_003746m	DYNAMIN-like 1C	GP
cassava4.1_003747m|PACid:17993539	cassava4.1_003747m	Glycosyl hydrolase family 47 protein	G
cassava4.1_003748m|PACid:17987844	cassava4.1_003748m	signal recognition particle receptor alpha subunit family protein	GP
cassava4.1_003749m|PACid:17984572	cassava4.1_003749m	N-acetyl-l-glutamate synthase 2	G
cassava4.1_003754m|PACid:17971627	cassava4.1_003754m	DYNAMIN-like 1E	P
cassava4.1_003756m|PACid:17975881	cassava4.1_003756m	peptidase M1 family protein	P
cassava4.1_003757m|PACid:17962621	cassava4.1_003757m	Phosphofructokinase family protein	GP
cassava4.1_003764m|PACid:17960358	cassava4.1_003764m	Methylthiotransferase	G
cassava4.1_003768m|PACid:17965079	cassava4.1_003768m	purple acid phosphatase 27	GP
cassava4.1_003770m|PACid:17974917	cassava4.1_003770m	S-adenosyl-L-methionine-dependent methyltransferases superfamily protein	G
cassava4.1_003776m|PACid:17992022	cassava4.1_003776m	Galactose oxidase/kelch repeat superfamily protein	G
cassava4.1_003788m|PACid:17989529	cassava4.1_003788m	methionyl-tRNA synthetase / methionine--tRNA ligase / MetRS (cpMetRS)	GP
cassava4.1_003792m|PACid:17967198	cassava4.1_003792m	phosphoglucosamine mutase family protein	GP
cassava4.1_003793m|PACid:17961352	cassava4.1_003793m	homologue of NAP57	GP
cassava4.1_003794m|PACid:17986953	cassava4.1_003794m	Auxin efflux carrier family protein	No
cassava4.1_003795m|PACid:17986469	cassava4.1_003795m	armadillo repeat only 4	G
cassava4.1_003805m|PACid:17967567	cassava4.1_003805m	dehydratase family	GP
cassava4.1_003812m|PACid:17961003	cassava4.1_003812m	Calmodulin-binding protein	No
cassava4.1_003815m|PACid:17971742	cassava4.1_003815m	Auxin-responsive GH3 family protein	No
cassava4.1_003823m|PACid:17990207	cassava4.1_003823m	Ribophorin I	P
cassava4.1_003828m|PACid:17980637	cassava4.1_003828m	PTEN 2	GP
cassava4.1_003829m|PACid:17973331	cassava4.1_003829m	Glucose-methanol-choline (GMC) oxidoreductase family protein	No
cassava4.1_003830m|PACid:17984166	cassava4.1_003830m	Major facilitator superfamily protein	No
cassava4.1_003831m|PACid:17980943	cassava4.1_003831m	vacuolar proton ATPase A1	G
cassava4.1_003833m|PACid:17985115	cassava4.1_003833m		No
cassava4.1_003837m|PACid:17972478	cassava4.1_003837m	Zinc finger C-x8-C-x5-C-x3-H type family protein	G
cassava4.1_003838m|PACid:17973943	cassava4.1_003838m	Auxin-responsive GH3 family protein	G
cassava4.1_003839m|PACid:17971363	cassava4.1_003839m	P-loop containing nucleoside triphosphate hydrolases superfamily protein	P
cassava4.1_003840m|PACid:17968467	cassava4.1_003840m	cell division cycle 48B	G
cassava4.1_003842m|PACid:17976181	cassava4.1_003842m	UDP-Glycosyltransferase superfamily protein	G
cassava4.1_003847m|PACid:17992146	cassava4.1_003847m	STRUBBELIG-receptor family 3	No
cassava4.1_003848m|PACid:17978016	cassava4.1_003848m	Major facilitator superfamily protein	No
cassava4.1_003851m|PACid:17981314	cassava4.1_003851m	P-loop containing nucleoside triphosphate hydrolases superfamily protein	G
cassava4.1_003852m|PACid:17968197	cassava4.1_003852m	Purple acid phosphatases superfamily protein	GP

cassava4.1_003857m|PACid:17970705	cassava4.1_003857m	S-adenosyl-L-methionine-dependent methyltransferases superfamily protein	GP
cassava4.1_003858m|PACid:17970767	cassava4.1_003858m	dynamin-like protein	GP
cassava4.1_003861m|PACid:17964662	cassava4.1_003861m	ABC transporter 1	No
cassava4.1_003864m|PACid:17988958	cassava4.1_003864m	leucine-rich repeat transmembrane protein kinase family protein	GP
cassava4.1_003865m|PACid:17977167	cassava4.1_003865m	U2 snRNP auxilliary factor, large subunit, splicing factor	No
cassava4.1_003868m|PACid:17976525	cassava4.1_003868m	cation-chloride co-transporter 1	No
cassava4.1_003869m|PACid:17965163	cassava4.1_003869m		No
cassava4.1_003870m|PACid:17966684	cassava4.1_003870m	methylenetetrahydrofolate reductase 2	GP
cassava4.1_003872m|PACid:17968086	cassava4.1_003872m	transducin family protein / WD-40 repeat family protein	GP
cassava4.1_003876m|PACid:17979292	cassava4.1_003876m	Phosphoinositide phosphatase family protein	No
cassava4.1_003877m|PACid:17977611	cassava4.1_003877m	UDP-Glycosyltransferase superfamily protein	P
cassava4.1_003878m|PACid:17970419	cassava4.1_003878m	transducin family protein / WD-40 repeat family protein	No
cassava4.1_003882m|PACid:17993569	cassava4.1_003882m	P-loop containing nucleoside triphosphate hydrolases superfamily protein	No
cassava4.1_003883m|PACid:17972412	cassava4.1_003883m	TCP-1/cpn60 chaperonin family protein	GP
cassava4.1_003885m|PACid:17977400	cassava4.1_003885m	fatty acid amide hydrolase	GP
cassava4.1_003888m|PACid:17991950	cassava4.1_003888m	anthranilate synthase 2	GP
cassava4.1_003890m|PACid:17987501	cassava4.1_003890m	N2,N2-dimethylguanosine tRNA methyltransferase	G
cassava4.1_003891m|PACid:17989544	cassava4.1_003891m	Peroxidase superfamily protein	No
cassava4.1_003892m|PACid:17993708	cassava4.1_003892m	AICARFT/IMPCHase bienzyme family protein	P
cassava4.1_003895m|PACid:17990740	cassava4.1_003895m	Subtilase family protein	GP
cassava4.1_003896m|PACid:17980127	cassava4.1_003896m	N2,N2-dimethylguanosine tRNA methyltransferase	G
cassava4.1_003901m|PACid:17963092	cassava4.1_003901m	pescadillo-related	GP
cassava4.1_003906m|PACid:17986155	cassava4.1_003906m	Protein kinase superfamily protein	No
cassava4.1_003907m|PACid:17972056	cassava4.1_003907m	TCP-1/cpn60 chaperonin family protein	GP
cassava4.1_003908m|PACid:17982639	cassava4.1_003908m	ATP citrate lyase subunit B 2	GP
cassava4.1_003909m|PACid:17980874	cassava4.1_003909m	SAND family protein	GP
cassava4.1_003910m|PACid:17967833	cassava4.1_003910m	tRNA synthetase class II (D, K and N) family protein	GP
cassava4.1_003912m|PACid:17983668	cassava4.1_003912m	AFG1-like ATPase family protein	G
cassava4.1_003916m|PACid:17989200	cassava4.1_003916m	UDP-Glycosyltransferase superfamily protein	No
cassava4.1_003917m|PACid:17980524	cassava4.1_003917m	Ankyrin repeat family protein	G
cassava4.1_003919m|PACid:17985707	cassava4.1_003919m	golgin candidate 1	No
cassava4.1_003920m|PACid:17990487	cassava4.1_003920m	CwfJ-like family protein / zinc finger (CCCH-type) family protein	GP
cassava4.1_003922m|PACid:17965742	cassava4.1_003922m	ZIP metal ion transporter family	G
cassava4.1_003924m|PACid:17961084	cassava4.1_003924m	L-O-methylthreonine resistant 1	GP
cassava4.1_003926m|PACid:17980766	cassava4.1_003926m	MAC/Perforin domain-containing protein	No
cassava4.1_003927m|PACid:17983804	cassava4.1_003927m	GTP-binding family protein	G
cassava4.1_003930m|PACid:17981847	cassava4.1_003930m	pectin methylesterase PCR fragment F	GP
cassava4.1_003936m|PACid:17962368	cassava4.1_003936m	Leucine-rich repeat protein kinase family protein	No
cassava4.1_003937m|PACid:17966195	cassava4.1_003937m	GRAS family transcription factor	G

cassava4.1_003939m|PACid:17978350	cassava4.1_003939m	Protein kinase family protein	No
cassava4.1_003941m|PACid:17964876	cassava4.1_003941m	translocon at the outer membrane of chloroplasts 64-V	GP
cassava4.1_003943m|PACid:17977358	cassava4.1_003943m	RECQ helicase L2	No
cassava4.1_003946m|PACid:17990787	cassava4.1_003946m	Thiamine pyrophosphate dependent pyruvate decarboxylase family protein	GP
cassava4.1_003947m|PACid:17969276	cassava4.1_003947m	UDP-GLUCOSE PYROPHOSPHORYLASE 1	GP
cassava4.1_003948m|PACid:17961263	cassava4.1_003948m	CTP synthase family protein	No
cassava4.1_003949m|PACid:17993652	cassava4.1_003949m	Leucine-rich repeat protein kinase family protein	No
cassava4.1_003954m|PACid:17980997	cassava4.1_003954m	RNAse l inhibitor protein 2	G
cassava4.1_003955m|PACid:17994125	cassava4.1_003955m	with no lysine (K) kinase 4	No
cassava4.1_003958m|PACid:17977469	cassava4.1_003958m	F-box/RNI-like superfamily protein	No
cassava4.1_003959m|PACid:17972731	cassava4.1_003959m	adenylate kinase family protein	GP
cassava4.1_003960m|PACid:17983555	cassava4.1_003960m	Tetratricopeptide repeat (TPR)-like superfamily protein	No
cassava4.1_003963m|PACid:17988666	cassava4.1_003963m	Cupredoxin superfamily protein	GP
cassava4.1_003965m|PACid:17981379	cassava4.1_003965m	DNAse I-like superfamily protein	GP
cassava4.1_003968m|PACid:17975491	cassava4.1_003968m	arogenate dehydrogenase	No
cassava4.1_003969m|PACid:17971345	cassava4.1_003969m	Trypsin family protein	GP
cassava4.1_003970m|PACid:17985094	cassava4.1_003970m	Protein of unknown function (DUF3550/UPF0682)	No
cassava4.1_003971m|PACid:17963455	cassava4.1_003971m	L-galactono-1,4-lactone dehydrogenase	GP
cassava4.1_003976m|PACid:17994047	cassava4.1_003976m	DNAse I-like superfamily protein	G
cassava4.1_003977m|PACid:17981526	cassava4.1_003977m	Phototropic-responsive NPH3 family protein	No
cassava4.1_003978m|PACid:17963204	cassava4.1_003978m	NAD-dependent malic enzyme 2	No
cassava4.1_003982m|PACid:17968035	cassava4.1_003982m	hydroxy methylglutaryl CoA reductase 1	G
cassava4.1_003986m|PACid:17961475	cassava4.1_003986m	Microtubule associated protein (MAP65/ASE1) family protein	GP
cassava4.1_003987m|PACid:17986942	cassava4.1_003987m	MAP kinase 9	G
cassava4.1_003990m|PACid:17973817	cassava4.1_003990m	S-adenosyl-L-methionine-dependent methyltransferases superfamily protein	No
cassava4.1_003991m|PACid:17966923	cassava4.1_003991m	Splicing factor, CC1-like	GP
cassava4.1_003994m|PACid:17974469	cassava4.1_003994m	cationic amino acid transporter 8	G
cassava4.1_004004m|PACid:17977905	cassava4.1_004004m	glucose-6-phosphate dehydrogenase 2	GP
cassava4.1_004007m|PACid:17971956	cassava4.1_004007m	phosphoribosylaminoimidazole carboxylase, putative / AIR carboxylase, putative	No
cassava4.1_004017m|PACid:17974289	cassava4.1_004017m	O-fucosyltransferase family protein	G
cassava4.1_004022m|PACid:17967365	cassava4.1_004022m	signal recognition particle-related / SRP-related	P
cassava4.1_004025m|PACid:17979428	cassava4.1_004025m	MAP kinase 20	G
cassava4.1_004026m|PACid:17968963	cassava4.1_004026m	Major facilitator superfamily protein	G
cassava4.1_004027m|PACid:17977360	cassava4.1_004027m	S-adenosyl-L-methionine-dependent methyltransferases superfamily protein	G
cassava4.1_004031m|PACid:17968360	cassava4.1_004031m	Exostosin family protein	No
cassava4.1_004032m|PACid:17962844	cassava4.1_004032m	Major facilitator superfamily protein	G
cassava4.1_004033m|PACid:17993782	cassava4.1_004033m	haloacid dehalogenase-like hydrolase family protein	G
cassava4.1_004034m|PACid:17969550	cassava4.1_004034m	nitrate transporter 1.5	No
cassava4.1_004036m|PACid:17970337	cassava4.1_004036m	CTP synthase family protein	G

cassava4.1_004040m|PACid:17972081	cassava4.1_004040m	NAD(P)-binding Rossmann-fold superfamily protein	GP
cassava4.1_004043m|PACid:17989026	cassava4.1_004043m	RNI-like superfamily protein	GP
cassava4.1_004046m|PACid:17960153	cassava4.1_004046m	E2F target gene 1	No
cassava4.1_004047m|PACid:17961160	cassava4.1_004047m	Basic-leucine zipper (bZIP) transcription factor family protein	GP
cassava4.1_004052m|PACid:17966224	cassava4.1_004052m	roline-rich extensin-like receptor kinase 4	No
cassava4.1_004054m|PACid:17986054	cassava4.1_004054m	plant U-box 49	G
cassava4.1_004055m|PACid:17972855	cassava4.1_004055m	AMP-dependent synthetase and ligase family protein	No
cassava4.1_004063m|PACid:17962587	cassava4.1_004063m	transmembrane nine 1	GP
cassava4.1_004065m|PACid:17987122	cassava4.1_004065m	Sec1/munc18-like (SM) proteins superfamily	No
cassava4.1_004068m|PACid:17981524	cassava4.1_004068m	putative indole-3-acetic acid-amido synthetase GH3.9	No
cassava4.1_004070m|PACid:17976329	cassava4.1_004070m	Cupredoxin superfamily protein	P
cassava4.1_004072m|PACid:17962781	cassava4.1_004072m	decapping 5	GP
cassava4.1_004081m|PACid:17968002	cassava4.1_004081m	ABC transporter family protein	GP
cassava4.1_004083m|PACid:17975472	cassava4.1_004083m	D-3-phosphoglycerate dehydrogenase	GP
cassava4.1_004090m|PACid:17979376	cassava4.1_004090m	AMP-dependent synthetase and ligase family protein	GP
cassava4.1_004091m|PACid:17971595	cassava4.1_004091m	cell division cycle 45	No
cassava4.1_004093m|PACid:17988093	cassava4.1_004093m	Major facilitator superfamily protein	No
cassava4.1_004097m|PACid:17963647	cassava4.1_004097m	glucose-6-phosphate dehydrogenase 1	GP
cassava4.1_004103m|PACid:17960250	cassava4.1_004103m	histone deacetylase 15	G
cassava4.1_004106m|PACid:17963208	cassava4.1_004106m	S-adenosyl-L-methionine-dependent methyltransferases superfamily protein	GP
cassava4.1_004107m|PACid:17985117	cassava4.1_004107m	P-loop containing nucleoside triphosphate hydrolases superfamily protein	G
cassava4.1_004114m|PACid:17969566	cassava4.1_004114m	ketol-acid reductoisomerase	GP
cassava4.1_004118m|PACid:17985461	cassava4.1_004118m	tRNA synthetase beta subunit family protein	No
cassava4.1_004124m|PACid:17975273	cassava4.1_004124m	DEAD-box protein abstrakt, putative	G
cassava4.1_004130m|PACid:17980733	cassava4.1_004130m	ROP guanine nucleotide exchange factor 5	No
cassava4.1_004132m|PACid:17961805	cassava4.1_004132m	aldehyde dehydrogenase 22A1	No
cassava4.1_004134m|PACid:17961335	cassava4.1_004134m	Protein kinase superfamily protein	G
cassava4.1_004141m|PACid:17983146	cassava4.1_004141m	phospholipase C 2	GP
cassava4.1_004147m|PACid:17982797	cassava4.1_004147m	Major facilitator superfamily protein	No
cassava4.1_004154m|PACid:17982029	cassava4.1_004154m	Phosphatidylinositol 3- and 4-kinase ;Ubiquitin family protein	No
cassava4.1_004156m|PACid:17981886	cassava4.1_004156m	dgd1 suppressor 1	No
cassava4.1_004164m|PACid:17985770	cassava4.1_004164m	NADP-malic enzyme 3	GP
cassava4.1_004165m|PACid:17985339	cassava4.1_004165m	SKU5 similar 2	GP
cassava4.1_004169m|PACid:17984624	cassava4.1_004169m	RING/U-box superfamily protein	G
cassava4.1_004173m|PACid:17963813	cassava4.1_004173m	Sec14p-like phosphatidylinositol transfer family protein	GP
cassava4.1_004175m|PACid:17961457	cassava4.1_004175m	Endomembrane protein 70 protein family	No
cassava4.1_004177m|PACid:17960929	cassava4.1_004177m	O-fucosyltransferase family protein	No
cassava4.1_004178m|PACid:17977726	cassava4.1_004178m	CDPK-related kinase	G
cassava4.1_004180m|PACid:17970477	cassava4.1_004180m	Phototropic-responsive NPH3 family protein	No

cassava4.1_004186m|PACid:17977793	cassava4.1_004186m	RING/U-box superfamily protein	No
cassava4.1_004187m|PACid:17993233	cassava4.1_004187m	cytochrome P450, family 97, subfamily A, polypeptide 3	GP
cassava4.1_004192m|PACid:17981717	cassava4.1_004192m	Ca2+-activated RelA/spot homolog	G
cassava4.1_004196m|PACid:17992142	cassava4.1_004196m	Auxin-responsive GH3 family protein	No
cassava4.1_004198m|PACid:17989553	cassava4.1_004198m	DegP protease 9	GP
cassava4.1_004201m|PACid:17974864	cassava4.1_004201m	IBR domain-containing protein	GP
cassava4.1_004204m|PACid:17991174	cassava4.1_004204m	amino acid transporter 1	G
cassava4.1_004206m|PACid:17975372	cassava4.1_004206m	glutamine-dependent asparagine synthase 1	GP
cassava4.1_004209m|PACid:17962619	cassava4.1_004209m	hydroxy methylglutaryl CoA reductase 1	No
cassava4.1_004210m|PACid:17969050	cassava4.1_004210m	TCP-1/cpn60 chaperonin family protein	GP
cassava4.1_004212m|PACid:17992688	cassava4.1_004212m	FKBP-type peptidyl-prolyl cis-trans isomerase family protein	G
cassava4.1_004221m|PACid:17976614	cassava4.1_004221m	subtilisin-like serine protease 2	GP
cassava4.1_004222m|PACid:17969968	cassava4.1_004222m	Pentatricopeptide repeat (PPR) superfamily protein	No
cassava4.1_004223m|PACid:17993297	cassava4.1_004223m	MuDR family transposase	GP
cassava4.1_004225m|PACid:17965307	cassava4.1_004225m	O-fucosyltransferase family protein	No
cassava4.1_004226m|PACid:17970175	cassava4.1_004226m	pectin methylesterase 1	GP
cassava4.1_004228m|PACid:17986952	cassava4.1_004228m	translocon at the outer membrane of chloroplasts 64-III	GP
cassava4.1_004230m|PACid:17993504	cassava4.1_004230m	Pyruvate kinase family protein	GP
cassava4.1_004233m|PACid:17970826	cassava4.1_004233m	nitrite reductase 1	No
cassava4.1_004234m|PACid:17967320	cassava4.1_004234m	Major facilitator superfamily protein	G
cassava4.1_004236m|PACid:17969375	cassava4.1_004236m	mitochondrial editing factor 22	G
cassava4.1_004239m|PACid:17973750	cassava4.1_004239m	Ankyrin repeat family protein	G
cassava4.1_004240m|PACid:17977548	cassava4.1_004240m	Thioesterase/thiol ester dehydrase-isomerase superfamily protein	GP
cassava4.1_004241m|PACid:17964281	cassava4.1_004241m	Arginyl-tRNA synthetase, class Ic	P
cassava4.1_004242m|PACid:17963659	cassava4.1_004242m	GLN phosphoribosyl pyrophosphate amidotransferase 2	G
cassava4.1_004243m|PACid:17984314	cassava4.1_004243m	gamma-glutamyl transpeptidase 1	GP
cassava4.1_004244m|PACid:17983866	cassava4.1_004244m	Endomembrane protein 70 protein family	G
cassava4.1_004249m|PACid:17969116	cassava4.1_004249m	protein phosphatase 2A subunit A2	No
cassava4.1_004256m|PACid:17978564	cassava4.1_004256m	phosphoinositide 4-kinase gamma 4	G
cassava4.1_004260m|PACid:17967136	cassava4.1_004260m	Galactose oxidase/kelch repeat superfamily protein	G
cassava4.1_004262m|PACid:17960643	cassava4.1_004262m	Protein of unknown function (DUF1423)	G
cassava4.1_004263m|PACid:17968763	cassava4.1_004263m	chaperonin-60alpha	GP
cassava4.1_004273m|PACid:17960353	cassava4.1_004273m	Polynucleotide adenylyltransferase family protein	P
cassava4.1_004274m|PACid:17984699	cassava4.1_004274m	peptide transporter 2	No
cassava4.1_004277m|PACid:17987214	cassava4.1_004277m	sodium/calcium exchanger family protein / calcium-binding EF hand family protein	GP
cassava4.1_004280m|PACid:17986008	cassava4.1_004280m	Cytosol aminopeptidase family protein	GP
cassava4.1_004281m|PACid:17991497	cassava4.1_004281m	Ankyrin repeat family protein	No
cassava4.1_004285m|PACid:17963557	cassava4.1_004285m	RNI-like superfamily protein	G
cassava4.1_004287m|PACid:17970734	cassava4.1_004287m	phosphoinositide 4-kinase gamma 4	No

cassava4.1_004288m|PACid:17982262	cassava4.1_004288m	Transducin/WD40 repeat-like superfamily protein	GP
cassava4.1_004289m|PACid:17964495	cassava4.1_004289m	RPM1 interacting protein 2	No
cassava4.1_004290m|PACid:17991922	cassava4.1_004290m	phosphatidylinositol-speciwc phospholipase C4	No
cassava4.1_004294m|PACid:17981483	cassava4.1_004294m	F-box/RNI-like superfamily protein	No
cassava4.1_004298m|PACid:17988236	cassava4.1_004298m	Calcium-dependent protein kinase (CDPK) family protein	No
cassava4.1_004299m|PACid:17979365	cassava4.1_004299m	SEC14-like 12	No
cassava4.1_004300m|PACid:17993689	cassava4.1_004300m	SAC domain-containing protein 8	GP
cassava4.1_004303m|PACid:17966792	cassava4.1_004303m		GP
cassava4.1_004304m|PACid:17993832	cassava4.1_004304m		No
cassava4.1_004306m|PACid:17993111	cassava4.1_004306m	glutamate tRNA synthetase	No
cassava4.1_004309m|PACid:17969940	cassava4.1_004309m	Leucine-rich repeat (LRR) family protein	P
cassava4.1_004310m|PACid:17961395	cassava4.1_004310m	peptide transporter 2	G
cassava4.1_004325m|PACid:17961732	cassava4.1_004325m	beta-amylase 1	G
cassava4.1_004327m|PACid:17979190	cassava4.1_004327m	cleavage and polyadenylation specificity factor 100	GP
cassava4.1_004329m|PACid:17984993	cassava4.1_004329m	proline-rich spliceosome-associated (PSP) family protein	G
cassava4.1_004330m|PACid:17963188	cassava4.1_004330m	fucosyltransferase 1	No
cassava4.1_004332m|PACid:17969721	cassava4.1_004332m	Phosphoglucomutase/phosphomannomutase family protein	GP
cassava4.1_004335m|PACid:17962105	cassava4.1_004335m	CVP2 like 1	G
cassava4.1_004341m|PACid:17963336	cassava4.1_004341m	microtubule-associated protein 65-8	No
cassava4.1_004343m|PACid:17992136	cassava4.1_004343m	NAD(P)H dehydrogenase B1	GP
cassava4.1_004352m|PACid:17961696	cassava4.1_004352m	Protein kinase superfamily protein	No
cassava4.1_004356m|PACid:17960161	cassava4.1_004356m	Major facilitator superfamily protein	No
cassava4.1_004357m|PACid:17970167	cassava4.1_004357m	pectin methylesterase 3	GP
cassava4.1_004361m|PACid:17962470	cassava4.1_004361m	FAD/NAD(P)-binding oxidoreductase family protein	No
cassava4.1_004363m|PACid:17993844	cassava4.1_004363m	ARM repeat superfamily protein	No
cassava4.1_004364m|PACid:17984941	cassava4.1_004364m	Major facilitator superfamily protein	G
cassava4.1_004370m|PACid:17980936	cassava4.1_004370m	laccase 17	No
cassava4.1_004371m|PACid:17969314	cassava4.1_004371m	RNApolymerase sigma subunit 2	G
cassava4.1_004373m|PACid:17960907	cassava4.1_004373m	cryptochrome 3	GP
cassava4.1_004376m|PACid:17961687	cassava4.1_004376m	NOP56-like pre RNA processing ribonucleoprotein	No
cassava4.1_004377m|PACid:17984178	cassava4.1_004377m	chromatin remodeling factor18	No
cassava4.1_004379m|PACid:17963312	cassava4.1_004379m	phosphate transporter 2;1	No
cassava4.1_004380m|PACid:17962060	cassava4.1_004380m	kinase associated protein phosphatase	No
cassava4.1_004384m|PACid:17980581	cassava4.1_004384m	Plant L-ascorbate oxidase	GP
cassava4.1_004385m|PACid:17985145	cassava4.1_004385m		No
cassava4.1_004397m|PACid:17987276	cassava4.1_004397m	Leucine-rich repeat family protein	No
cassava4.1_004401m|PACid:17965072	cassava4.1_004401m	Protein kinase superfamily protein	No
cassava4.1_004402m|PACid:17983479	cassava4.1_004402m	Seven transmembrane MLO family protein	No
cassava4.1_004404m|PACid:17984193	cassava4.1_004404m	glycosyl hydrolase 9C1	G

cassava4.1_004405m|PACid:17989978	cassava4.1_004405m	plastidic pyruvate kinase beta subunit 1	GP
cassava4.1_004406m|PACid:17965775	cassava4.1_004406m	beta-hexosaminidase 2	No
cassava4.1_004407m|PACid:17967349	cassava4.1_004407m	DCD (Development and Cell Death) domain protein	GP
cassava4.1_004415m|PACid:17976388	cassava4.1_004415m	Class II aminoacyl-tRNA and biotin synthetases superfamily protein	No
cassava4.1_004416m|PACid:17964075	cassava4.1_004416m	O-acetyltransferase family protein	G
cassava4.1_004422m|PACid:17991847	cassava4.1_004422m	anaphase-promoting complex subunit 8	No
cassava4.1_004423m|PACid:17981824	cassava4.1_004423m	CLP protease regulatory subunit X	G
cassava4.1_004425m|PACid:17967702	cassava4.1_004425m	DEA(D/H)-box RNA helicase family protein	G
cassava4.1_004426m|PACid:17985267	cassava4.1_004426m	K+ efflux antiporter 5	No
cassava4.1_004428m|PACid:17966255	cassava4.1_004428m	ACT-like protein tyrosine kinase family protein	G
cassava4.1_004429m|PACid:17974083	cassava4.1_004429m	Glycosyl hydrolase family 47 protein	G
cassava4.1_004430m|PACid:17991699	cassava4.1_004430m	laccase 17	No
cassava4.1_004432m|PACid:17979373	cassava4.1_004432m	ACT-like protein tyrosine kinase family protein	No
cassava4.1_004434m|PACid:17965542	cassava4.1_004434m	NOP56-like pre RNA processing ribonucleoprotein	GP
cassava4.1_004435m|PACid:17988874	cassava4.1_004435m	anthranilate synthase alpha subunit 1	No
cassava4.1_004437m|PACid:17960366	cassava4.1_004437m	P-loop containing nucleoside triphosphate hydrolases superfamily protein	No
cassava4.1_004440m|PACid:17983077	cassava4.1_004440m	CDPK-related kinase 1	G
cassava4.1_004444m|PACid:17970392	cassava4.1_004444m	inositol transporter 2	G
cassava4.1_004445m|PACid:17977229	cassava4.1_004445m	Glucose-methanol-choline (GMC) oxidoreductase family protein	GP
cassava4.1_004446m|PACid:17978572	cassava4.1_004446m	Auxin-responsive GH3 family protein	GP
cassava4.1_004447m|PACid:17959895	cassava4.1_004447m	Cysteinyl-tRNA synthetase, class Ia family protein	No
cassava4.1_004448m|PACid:17991871	cassava4.1_004448m	SKU5 similar 15	G
cassava4.1_004452m|PACid:17978901	cassava4.1_004452m	Phototropic-responsive NPH3 family protein	GP
cassava4.1_004453m|PACid:17977228	cassava4.1_004453m	stress-inducible protein, putative	GP
cassava4.1_004454m|PACid:17990345	cassava4.1_004454m	Heat shock protein DnaJ with tetratricopeptide repeat	No
cassava4.1_004455m|PACid:17985268	cassava4.1_004455m	K+ efflux antiporter 5	G
cassava4.1_004458m|PACid:17962184	cassava4.1_004458m	heat shock protein 60	P
cassava4.1_004459m|PACid:17973612	cassava4.1_004459m	Plant invertase/pectin methylesterase inhibitor superfamily	No
cassava4.1_004460m|PACid:17961203	cassava4.1_004460m	DNA/RNA helicase protein	No
cassava4.1_004466m|PACid:17976152	cassava4.1_004466m	calcium dependent protein kinase 1	G
cassava4.1_004468m|PACid:17990830	cassava4.1_004468m	ARM repeat superfamily protein	No
cassava4.1_004470m|PACid:17966571	cassava4.1_004470m	high chlorophyll fluorescence phenotype 173	GP
cassava4.1_004476m|PACid:17962772	cassava4.1_004476m	STT7 homolog STN7	GP
cassava4.1_004480m|PACid:17971328	cassava4.1_004480m	dicarboxylate transporter 1	P
cassava4.1_004481m|PACid:17975156	cassava4.1_004481m	Leucine-rich repeat protein kinase family protein	G
cassava4.1_004484m|PACid:17970009	cassava4.1_004484m	Protein of unknown function (DUF616)	No
cassava4.1_004487m|PACid:17976794	cassava4.1_004487m	K+ uptake permease 11	No
cassava4.1_004490m|PACid:17978855	cassava4.1_004490m	Pyridoxal phosphate (PLP)-dependent transferases superfamily protein	GP
cassava4.1_004491m|PACid:17970874	cassava4.1_004491m	trehalase 1	No

cassava4.1_004493m|PACid:17976054	cassava4.1_004493m	Eukaryotic translation initiation factor 3 subunit 7 (eIF-3)	GP
cassava4.1_004496m|PACid:17973062	cassava4.1_004496m	transducin family protein / WD-40 repeat family protein	G
cassava4.1_004497m|PACid:17961300	cassava4.1_004497m		No
cassava4.1_004499m|PACid:17992766	cassava4.1_004499m		No
cassava4.1_004501m|PACid:17961116	cassava4.1_004501m	chloroplast signal recognition particle 54 kDa subunit	GP
cassava4.1_004502m|PACid:17960337	cassava4.1_004502m	Major facilitator superfamily protein	G
cassava4.1_004507m|PACid:17979383	cassava4.1_004507m	Seven transmembrane MLO family protein	G
cassava4.1_004508m|PACid:17971393	cassava4.1_004508m	ATP-binding casette family G25	No
cassava4.1_004510m|PACid:17993016	cassava4.1_004510m	mitogen-activated protein kinase kinase kinase 9	G
cassava4.1_004516m|PACid:17979404	cassava4.1_004516m	plant intracellular ras group-related LRR 4	GP
cassava4.1_004519m|PACid:17964023	cassava4.1_004519m	rho guanyl-nucleotide exchange factor 1	G
cassava4.1_004520m|PACid:17991341	cassava4.1_004520m	auxin signaling F-box 3	No
cassava4.1_004524m|PACid:17973994	cassava4.1_004524m	nitrate transporter 1:2	No
cassava4.1_004530m|PACid:17989017	cassava4.1_004530m	asparagine synthetase 3	GP
cassava4.1_004534m|PACid:17979818	cassava4.1_004534m	RING/U-box superfamily protein	No
cassava4.1_004535m|PACid:17979926	cassava4.1_004535m		GP
cassava4.1_004542m|PACid:17989802	cassava4.1_004542m	dicarboxylate transport 2.1	P
cassava4.1_004544m|PACid:17982885	cassava4.1_004544m	Alg9-like mannosyltransferase family	GP
cassava4.1_004554m|PACid:17980978	cassava4.1_004554m	Eukaryotic aspartyl protease family protein	No
cassava4.1_004562m|PACid:17989429	cassava4.1_004562m	Plant protein of unknown function (DUF946)	G
cassava4.1_004565m|PACid:17992817	cassava4.1_004565m	DC1 domain-containing protein	GP
cassava4.1_004569m|PACid:17986411	cassava4.1_004569m	peptide transporter 1	No
cassava4.1_004570m|PACid:17988734	cassava4.1_004570m	cationic amino acid transporter 9	G
cassava4.1_004571m|PACid:17987804	cassava4.1_004571m		No
cassava4.1_004572m|PACid:17984514	cassava4.1_004572m	vacuolar protein sorting 45	No
cassava4.1_004573m|PACid:17959964	cassava4.1_004573m	FAD-linked oxidases family protein	GP
cassava4.1_004577m|PACid:17966476	cassava4.1_004577m	calcium-dependent protein kinase 28	No
cassava4.1_004579m|PACid:17979619	cassava4.1_004579m	lipoamide dehydrogenase 1	P
cassava4.1_004580m|PACid:17964813	cassava4.1_004580m	GTP cyclohydrolase II	GP
cassava4.1_004581m|PACid:17978999	cassava4.1_004581m	Sugar isomerase (SIS) family protein	GP
cassava4.1_004588m|PACid:17986273	cassava4.1_004588m	histone acetyltransferase of the GNAT family 1	No
cassava4.1_004589m|PACid:17966836	cassava4.1_004589m	Pentatricopeptide repeat (PPR) superfamily protein	GP
cassava4.1_004596m|PACid:17976069	cassava4.1_004596m	RNA methyltransferase family protein	No
cassava4.1_004598m|PACid:17993507	cassava4.1_004598m	GLU-ADT subunit B	No
cassava4.1_004602m|PACid:17991512	cassava4.1_004602m	Phosphofructokinase family protein	GP
cassava4.1_004607m|PACid:17979057	cassava4.1_004607m	pectin methylesterase 61	GP
cassava4.1_004611m|PACid:17992211	cassava4.1_004611m		G
cassava4.1_004623m|PACid:17975278	cassava4.1_004623m	Major facilitator superfamily protein	No
cassava4.1_004624m|PACid:17982721	cassava4.1_004624m	VACUOLAR SORTING RECEPTOR 6	G

cassava4.1_004625m|PACid:17993873	cassava4.1_004625m		GP
cassava4.1_004630m|PACid:17991144	cassava4.1_004630m	ATP synthase alpha/beta family protein	GP
cassava4.1_004631m|PACid:17962087	cassava4.1_004631m	acyl-activating enzyme 7	GP
cassava4.1_004632m|PACid:17978852	cassava4.1_004632m	O-fucosyltransferase family protein	No
cassava4.1_004635m|PACid:17992778	cassava4.1_004635m	RED family protein	No
cassava4.1_004641m|PACid:17967036	cassava4.1_004641m	WD-40 repeat family protein	No
cassava4.1_004643m|PACid:17975918	cassava4.1_004643m	AMP-dependent synthetase and ligase family protein	No
cassava4.1_004644m|PACid:17972871	cassava4.1_004644m	Ubiquitin C-terminal hydrolases superfamily protein	GP
cassava4.1_004646m|PACid:17993707	cassava4.1_004646m	Tubulin binding cofactor C domain-containing protein	GP
cassava4.1_004654m|PACid:17960311	cassava4.1_004654m	fatty acid biosynthesis 1	P
cassava4.1_004658m|PACid:17965719	cassava4.1_004658m	4-coumarate:CoA ligase 3	GP
cassava4.1_004659m|PACid:17984653	cassava4.1_004659m	SWIB/MDM2 domain superfamily protein	No
cassava4.1_004661m|PACid:17965983	cassava4.1_004661m	D-aminoacid aminotransferase-like PLP-dependent enzymes superfamily protein	No
cassava4.1_004662m|PACid:17965003	cassava4.1_004662m	transducin family protein / WD-40 repeat family protein	No
cassava4.1_004665m|PACid:17971640	cassava4.1_004665m	Protein of unknown function, DUF647	G
cassava4.1_004666m|PACid:17973986	cassava4.1_004666m	radical SAM domain-containing protein / GCN5-related N-acetyltransferase (GNAT) family protein	G
cassava4.1_004670m|PACid:17969647	cassava4.1_004670m	cell elongation protein / DWARF1 / DIMINUTO (DIM)	GP
cassava4.1_004672m|PACid:17966236	cassava4.1_004672m	damaged DNA binding 2	G
cassava4.1_004677m|PACid:17965754	cassava4.1_004677m	glutathione synthetase 2	P
cassava4.1_004678m|PACid:17983772	cassava4.1_004678m	thiamin biosynthesis protein, putative	No
cassava4.1_004679m|PACid:17969716	cassava4.1_004679m	ENTH/ANTH/VHS superfamily protein	P
cassava4.1_004681m|PACid:17961744	cassava4.1_004681m	mitogen-activated protein kinase 16	G
cassava4.1_004682m|PACid:17974569	cassava4.1_004682m	subunit of exocyst complex 8	No
cassava4.1_004684m|PACid:17970157	cassava4.1_004684m	O-fucosyltransferase family protein	GP
cassava4.1_004686m|PACid:17967073	cassava4.1_004686m	TCP-1/cpn60 chaperonin family protein	GP
cassava4.1_004690m|PACid:17966414	cassava4.1_004690m	FAD/NAD(P)-binding oxidoreductase family protein	GP
cassava4.1_004699m|PACid:17972946	cassava4.1_004699m	appr-1-p processing enzyme family protein	GP
cassava4.1_004700m|PACid:17981278	cassava4.1_004700m	Major facilitator superfamily protein	No
cassava4.1_004701m|PACid:17960978	cassava4.1_004701m	Glutamyl-tRNA reductase family protein	GP
cassava4.1_004702m|PACid:17968004	cassava4.1_004702m	phosphoglucosamine mutase-related	GP
cassava4.1_004706m|PACid:17987010	cassava4.1_004706m	FKBP-type peptidyl-prolyl cis-trans isomerase family protein	GP
cassava4.1_004712m|PACid:17989634	cassava4.1_004712m	galacturonosyltransferase 9	No
cassava4.1_004718m|PACid:17983180	cassava4.1_004718m	Outer membrane OMP85 family protein	GP
cassava4.1_004723m|PACid:17991742	cassava4.1_004723m	AMP-dependent synthetase and ligase family protein	G
cassava4.1_004725m|PACid:17971590	cassava4.1_004725m	Nucleotide-diphospho-sugar transferases superfamily protein	GP
cassava4.1_004727m|PACid:17970597	cassava4.1_004727m	3beta-hydroxysteroid-dehydrogenase/decarboxylase isoform 2	No
cassava4.1_004729m|PACid:17976261	cassava4.1_004729m	ARM repeat superfamily protein	G
cassava4.1_004731m|PACid:17964051	cassava4.1_004731m	Magnesium transporter CorA-like family protein	G
cassava4.1_004736m|PACid:17965607	cassava4.1_004736m	Phosphoglycerate mutase, 2,3-bisphosphoglycerate-independent	No

cassava4.1_004739m|PACid:17979457	cassava4.1_004739m	GTP-binding family protein	G
cassava4.1_004740m|PACid:17982202	cassava4.1_004740m	nicotinate phosphoribosyltransferase 2	No
cassava4.1_004741m|PACid:17980034	cassava4.1_004741m	NAD(P)H dehydrogenase B2	No
cassava4.1_004749m|PACid:17986281	cassava4.1_004749m	glutathione reductase	No
cassava4.1_004751m|PACid:17964037	cassava4.1_004751m	MAC/Perforin domain-containing protein	GP
cassava4.1_004755m|PACid:17961953	cassava4.1_004755m	tetratricopeptide repeat (TPR)-containing protein	G
cassava4.1_004757m|PACid:17989945	cassava4.1_004757m	Folylpolyglutamate synthetase family protein	G
cassava4.1_004758m|PACid:17990243	cassava4.1_004758m	IBR domain-containing protein	G
cassava4.1_004759m|PACid:17965042	cassava4.1_004759m	Serine/threonine-protein kinase Rio1	G
cassava4.1_004761m|PACid:17966385	cassava4.1_004761m	Laccase/Diphenol oxidase family protein	No
cassava4.1_004766m|PACid:17963458	cassava4.1_004766m	Pentatricopeptide repeat (PPR) superfamily protein	G
cassava4.1_004767m|PACid:17980248	cassava4.1_004767m	Pentatricopeptide repeat (PPR) superfamily protein	No
cassava4.1_004768m|PACid:17971777	cassava4.1_004768m	serine hydroxymethyltransferase 7	GP
cassava4.1_004771m|PACid:17981254	cassava4.1_004771m	limit dextrinase	GP
cassava4.1_004773m|PACid:17971974	cassava4.1_004773m	Calcium-dependent protein kinase family protein	GP
cassava4.1_004774m|PACid:17981140	cassava4.1_004774m	calcium ion binding	No
cassava4.1_004778m|PACid:17960985	cassava4.1_004778m	xylulose kinase-2	No
cassava4.1_004781m|PACid:17977206	cassava4.1_004781m	P-loop containing nucleoside triphosphate hydrolases superfamily protein	G
cassava4.1_004784m|PACid:17970369	cassava4.1_004784m	Cupredoxin superfamily protein	P
cassava4.1_004785m|PACid:17986381	cassava4.1_004785m	Laccase/Diphenol oxidase family protein	No
cassava4.1_004787m|PACid:17971636	cassava4.1_004787m	MATE efflux family protein	No
cassava4.1_004789m|PACid:17985737	cassava4.1_004789m	phosphofructokinase 3	GP
cassava4.1_004790m|PACid:17975620	cassava4.1_004790m	laccase 6	No
cassava4.1_004793m|PACid:17969946	cassava4.1_004793m	Seven transmembrane MLO family protein	No
cassava4.1_004797m|PACid:17972900	cassava4.1_004797m	sorting nexin 2A	No
cassava4.1_004800m|PACid:17989601	cassava4.1_004800m	TCP-1/cpn60 chaperonin family protein	GP
cassava4.1_004804m|PACid:17980951	cassava4.1_004804m	RING/U-box superfamily protein	No
cassava4.1_004805m|PACid:17963660	cassava4.1_004805m	Ypt/Rab-GAP domain of gyp1p superfamily protein	G
cassava4.1_004814m|PACid:17980909	cassava4.1_004814m	PDI-like 1-4	GP
cassava4.1_004816m|PACid:17968554	cassava4.1_004816m	myb domain protein 3r-5	G
cassava4.1_004817m|PACid:17978631	cassava4.1_004817m	Aspartate kinase family protein	GP
cassava4.1_004818m|PACid:17967333	cassava4.1_004818m		G
cassava4.1_004819m|PACid:17961063	cassava4.1_004819m	ser/arg-rich protein kinase 4	No
cassava4.1_004820m|PACid:17963015	cassava4.1_004820m	stomatal cytokinesis defective / SCD1 protein (SCD1)	No
cassava4.1_004824m|PACid:17964231	cassava4.1_004824m	Class II aminoacyl-tRNA and biotin synthetases superfamily protein	GP
cassava4.1_004825m|PACid:17965423	cassava4.1_004825m	acyl activating enzyme 5	GP
cassava4.1_004827m|PACid:17981544	cassava4.1_004827m	Plant invertase/pectin methylesterase inhibitor superfamily	GP
cassava4.1_004831m|PACid:17969039	cassava4.1_004831m	aldehyde dehydrogenase 12A1	GP
cassava4.1_004836m|PACid:17963019	cassava4.1_004836m	CCAAT-displacement protein alternatively spliced product	No

cassava4.1_004840m|PACid:17973420	cassava4.1_004840m	Chaperone DnaJ-domain superfamily protein	No
cassava4.1_004841m|PACid:17984579	cassava4.1_004841m	squalene epoxidase 2	No
cassava4.1_004843m|PACid:17989588	cassava4.1_004843m	plastidic GLC translocator	No
cassava4.1_004848m|PACid:17967346	cassava4.1_004848m	homolog of nucleolar protein NOP56	GP
cassava4.1_004849m|PACid:17967098	cassava4.1_004849m	Transducin/WD40 repeat-like superfamily protein	No
cassava4.1_004852m|PACid:17991481	cassava4.1_004852m	ATP binding cassette protein 1	GP
cassava4.1_004858m|PACid:17988716	cassava4.1_004858m	GTP cyclohydrolase II	P
cassava4.1_004863m|PACid:17986632	cassava4.1_004863m	P-loop containing nucleoside triphosphate hydrolases superfamily protein	No
cassava4.1_004864m|PACid:17973137	cassava4.1_004864m	Dihydrolipoamide acetyltransferase, long form protein	GP
cassava4.1_004866m|PACid:17981242	cassava4.1_004866m	Transmembrane amino acid transporter family protein	G
cassava4.1_004870m|PACid:17989709	cassava4.1_004870m	ENTH/ANTH/VHS superfamily protein	GP
cassava4.1_004872m|PACid:17978581	cassava4.1_004872m	BTB/POZ/Kelch-associated protein	No
cassava4.1_004883m|PACid:17968505	cassava4.1_004883m	MATE efflux family protein	No
cassava4.1_004884m|PACid:17993658	cassava4.1_004884m	nitrate transporter 1:2	G
cassava4.1_004889m|PACid:17976861	cassava4.1_004889m	P-loop containing nucleoside triphosphate hydrolases superfamily protein	GP
cassava4.1_004897m|PACid:17969742	cassava4.1_004897m	Plant protein of unknown function (DUF946)	G
cassava4.1_004898m|PACid:17975565	cassava4.1_004898m	exocyst complex component sec10	GP
cassava4.1_004900m|PACid:17974867	cassava4.1_004900m	Spc97 / Spc98 family of spindle pole body (SBP) component	No
cassava4.1_004901m|PACid:17975722	cassava4.1_004901m	POZ/BTB containin G-protein 1	No
cassava4.1_004909m|PACid:17990159	cassava4.1_004909m	OPC-8:0 CoA ligase1	GP
cassava4.1_004921m|PACid:17966950	cassava4.1_004921m	acyl activating enzyme 1	GP
cassava4.1_004922m|PACid:17969086	cassava4.1_004922m	calcium-dependent protein kinase 1	GP
cassava4.1_004923m|PACid:17960464	cassava4.1_004923m	cytokinin oxidase 5	No
cassava4.1_004925m|PACid:17993391	cassava4.1_004925m	AGC (cAMP-dependent, cGMP-dependent and protein kinase C) kinase family protein	No
cassava4.1_004928m|PACid:17970019	cassava4.1_004928m	Apoptosis inhibitory protein 5 (API5)	GP
cassava4.1_004934m|PACid:17993470	cassava4.1_004934m	actin-related protein 9	No
cassava4.1_004939m|PACid:17979192	cassava4.1_004939m	ubiquitin family protein	No
cassava4.1_004943m|PACid:17978153	cassava4.1_004943m	purple acid phosphatase 15	No
cassava4.1_004947m|PACid:17964024	cassava4.1_004947m	SKU5 similar 5	GP
cassava4.1_004948m|PACid:17961356	cassava4.1_004948m	protein kinase family protein / C-type lectin domain-containing protein	No
cassava4.1_004949m|PACid:17979193	cassava4.1_004949m	ubiquitin family protein	No
cassava4.1_004951m|PACid:17988081	cassava4.1_004951m	GRAS family transcription factor	No
cassava4.1_004954m|PACid:17960750	cassava4.1_004954m	Class II aaRS and biotin synthetases superfamily protein	GP
cassava4.1_004956m|PACid:17967285	cassava4.1_004956m	Transducin/WD40 repeat-like superfamily protein	No
cassava4.1_004958m|PACid:17982167	cassava4.1_004958m	FRIGIDA-like protein	G
cassava4.1_004968m|PACid:17978859	cassava4.1_004968m	RNA-binding KH domain-containing protein	G
cassava4.1_004969m|PACid:17965827	cassava4.1_004969m	sodium hydrogen exchanger 2	G
cassava4.1_004975m|PACid:17988780	cassava4.1_004975m	T-complex protein 1 alpha subunit	GP
cassava4.1_004981m|PACid:17973582	cassava4.1_004981m	SKU5 similar 5	GP

cassava4.1_004982m|PACid:17979712	cassava4.1_004982m	protein arginine methyltransferase 4A	GP
cassava4.1_004987m|PACid:17964713	cassava4.1_004987m	Flavin containing amine oxidoreductase family	GP
cassava4.1_004991m|PACid:17985873	cassava4.1_004991m	translocon at the inner envelope membrane of chloroplasts 55-II	GP
cassava4.1_004999m|PACid:17988246	cassava4.1_004999m	Ypt/Rab-GAP domain of gyp1p superfamily protein	G
cassava4.1_005000m|PACid:17979142	cassava4.1_005000m	Amidase family protein	No
cassava4.1_005003m|PACid:17965856	cassava4.1_005003m	TCP-1/cpn60 chaperonin family protein	GP
cassava4.1_005005m|PACid:17986681	cassava4.1_005005m	Pentatricopeptide repeat (PPR-like) superfamily protein	No
cassava4.1_005006m|PACid:17965395	cassava4.1_005006m	4-coumarate:CoA ligase 2	GP
cassava4.1_005008m|PACid:17975549	cassava4.1_005008m	ARM repeat superfamily protein	No
cassava4.1_005010m|PACid:17977834	cassava4.1_005010m	homolog of yeast ADA2 2A	G
cassava4.1_005019m|PACid:17987381	cassava4.1_005019m	Cytochrome P450 superfamily protein	No
cassava4.1_005020m|PACid:17966828	cassava4.1_005020m	Integrase-type DNA-binding superfamily protein	No
cassava4.1_005021m|PACid:17987373	cassava4.1_005021m	Acyl-CoA N-acyltransferase with RING/FYVE/PHD-type zinc finger domain	No
cassava4.1_005023m|PACid:17967512	cassava4.1_005023m	EPS15 homology domain 1	No
cassava4.1_005025m|PACid:17981207	cassava4.1_005025m	DNA topoisomerase, type IA, core	No
cassava4.1_005038m|PACid:17983172	cassava4.1_005038m	ABC2 homolog 9	G
cassava4.1_005039m|PACid:17964951	cassava4.1_005039m	Protein phosphatase 2A regulatory B subunit family protein	GP
cassava4.1_005044m|PACid:17961592	cassava4.1_005044m	dihydrosphingosine phosphate lyase	GP
cassava4.1_005046m|PACid:17967709	cassava4.1_005046m	nucleobase-ascorbate transporter 7	No
cassava4.1_005049m|PACid:17988347	cassava4.1_005049m		G
cassava4.1_005050m|PACid:17967048	cassava4.1_005050m	cytochrome P450, family 86, subfamily B, polypeptide 1	No
cassava4.1_005051m|PACid:17971648	cassava4.1_005051m	transducin family protein / WD-40 repeat family protein	No
cassava4.1_005052m|PACid:17963904	cassava4.1_005052m	AMP-dependent synthetase and ligase family protein	GP
cassava4.1_005058m|PACid:17976451	cassava4.1_005058m	Pyridoxal phosphate (PLP)-dependent transferases superfamily protein	P
cassava4.1_005063m|PACid:17971382	cassava4.1_005063m	SIGNAL PEPTIDE PEPTIDASE-LIKE 3	No
cassava4.1_005065m|PACid:17974110	cassava4.1_005065m	proline-rich family protein	G
cassava4.1_005068m|PACid:17982159	cassava4.1_005068m	calmodulin-binding family protein	G
cassava4.1_005069m|PACid:17969664	cassava4.1_005069m	GTP-binding protein, HflX	G
cassava4.1_005070m|PACid:17987268	cassava4.1_005070m	Alg9-like mannosyltransferase family	No
cassava4.1_005072m|PACid:17968940	cassava4.1_005072m	glucuronidase 2	GP
cassava4.1_005074m|PACid:17968433	cassava4.1_005074m	Calcium-binding EF-hand family protein	G
cassava4.1_005077m|PACid:17975723	cassava4.1_005077m	cytochrome P450, family 86, subfamily A, polypeptide 8	No
cassava4.1_005089m|PACid:17991492	cassava4.1_005089m	SKU5 similar 4	P
cassava4.1_005091m|PACid:17965945	cassava4.1_005091m	Calcium-binding EF-hand family protein	No
cassava4.1_005092m|PACid:17968414	cassava4.1_005092m	aldehyde dehydrogenase 2B4	GP
cassava4.1_005096m|PACid:17961750	cassava4.1_005096m	purple acid phosphatase 23	No
cassava4.1_005101m|PACid:17978811	cassava4.1_005101m	DNAJ heat shock N-terminal domain-containing protein	No
cassava4.1_005104m|PACid:17981899	cassava4.1_005104m	pyridoxin (pyrodoxamine) 5\'-phosphate oxidase	No
cassava4.1_005105m|PACid:17977497	cassava4.1_005105m	synaptotagmin A	No

cassava4.1_005110m|PACid:17971620	cassava4.1_005110m	cytochrome P450, family 86, subfamily A, polypeptide 8	GP
cassava4.1_005111m|PACid:17961153	cassava4.1_005111m	GLN phosphoribosyl pyrophosphate amidotransferase 2	No
cassava4.1_005115m|PACid:17981591	cassava4.1_005115m	magnesium/proton exchanger	G
cassava4.1_005116m|PACid:17973827	cassava4.1_005116m	Peptide chain release factor 2	No
cassava4.1_005118m|PACid:17977037	cassava4.1_005118m	P-loop containing nucleoside triphosphate hydrolases superfamily protein	No
cassava4.1_005119m|PACid:17970984	cassava4.1_005119m	Peptidase M1 family protein	GP
cassava4.1_005121m|PACid:17969827	cassava4.1_005121m	Ankyrin repeat family protein	G
cassava4.1_005126m|PACid:17973532	cassava4.1_005126m	cellulose synthase-like A02	GP
cassava4.1_005130m|PACid:17979364	cassava4.1_005130m	polyamine oxidase 4	G
cassava4.1_005131m|PACid:17981307	cassava4.1_005131m	ACD1-like	No
cassava4.1_005134m|PACid:17989899	cassava4.1_005134m	carotenoid cleavage dioxygenase 8	No
cassava4.1_005136m|PACid:17965084	cassava4.1_005136m	calnexin 1	GP
cassava4.1_005137m|PACid:17972358	cassava4.1_005137m	RAN GTPase activating protein 2	G
cassava4.1_005144m|PACid:17962993	cassava4.1_005144m	DNA photolyase family protein	No
cassava4.1_005147m|PACid:17986645	cassava4.1_005147m	HAD-superfamily hydrolase, subfamily IG, 5\'-nucleotidase	GP
cassava4.1_005148m|PACid:17985914	cassava4.1_005148m	initiator tRNA phosphoribosyl transferase family protein	No
cassava4.1_005149m|PACid:17992418	cassava4.1_005149m	Protein of unknown function (DUF3411)	GP
cassava4.1_005155m|PACid:17986543	cassava4.1_005155m	aldehyde dehydrogenase 6B2	GP
cassava4.1_005156m|PACid:17963426	cassava4.1_005156m	MATE efflux family protein	G
cassava4.1_005163m|PACid:17975630	cassava4.1_005163m	SIGNAL PEPTIDE PEPTIDASE-LIKE 4	G
cassava4.1_005164m|PACid:17960526	cassava4.1_005164m	Calcium-dependent lipid-binding (CaLB domain) family protein	GP
cassava4.1_005171m|PACid:17972853	cassava4.1_005171m	ARM repeat superfamily protein	GP
cassava4.1_005176m|PACid:17971268	cassava4.1_005176m	calmodulin-domain protein kinase 7	G
cassava4.1_005181m|PACid:17992646	cassava4.1_005181m	SKU5 similar 17	GP
cassava4.1_005183m|PACid:17971404	cassava4.1_005183m	tubulin folding cofactor E / Pfifferling (PFI)	No
cassava4.1_005186m|PACid:17992773	cassava4.1_005186m	Plant invertase/pectin methylesterase inhibitor superfamily	No
cassava4.1_005189m|PACid:17968407	cassava4.1_005189m	Pre-mRNA splicing Prp18-interacting factor	No
cassava4.1_005198m|PACid:17975584	cassava4.1_005198m	origin recognition complex protein 5	No
cassava4.1_005199m|PACid:17991911	cassava4.1_005199m	Tetratricopeptide repeat (TPR)-like superfamily protein	No
cassava4.1_005200m|PACid:17970486	cassava4.1_005200m	DEA(D/H)-box RNA helicase family protein	G
cassava4.1_005207m|PACid:17980662	cassava4.1_005207m	L-Aspartase-like family protein	GP
cassava4.1_005209m|PACid:17988529	cassava4.1_005209m	Class-II DAHP synthetase family protein	GP
cassava4.1_005211m|PACid:17968743	cassava4.1_005211m	LIM domain-containing protein	No
cassava4.1_005212m|PACid:17962189	cassava4.1_005212m	phosphate transporter 1;3	GP
cassava4.1_005213m|PACid:17984464	cassava4.1_005213m	NRAMP metal ion transporter 2	G
cassava4.1_005215m|PACid:17972210	cassava4.1_005215m	Class II aminoacyl-tRNA and biotin synthetases superfamily protein	P
cassava4.1_005217m|PACid:17974485	cassava4.1_005217m	phosphofructokinase 5	No
cassava4.1_005218m|PACid:17984672	cassava4.1_005218m	polyol/monosaccharide transporter 5	G
cassava4.1_005219m|PACid:17975112	cassava4.1_005219m	TATA BOX ASSOCIATED FACTOR II 59	G

cassava4.1_005221m|PACid:17968412	cassava4.1_005221m	phosphate transporter 1;7	G
cassava4.1_005222m|PACid:17963072	cassava4.1_005222m	Pseudouridine synthase family protein	G
cassava4.1_005224m|PACid:17978879	cassava4.1_005224m	galacturonosyltransferase 13	No
cassava4.1_005227m|PACid:17982297	cassava4.1_005227m	phosphate transporter 1;7	G
cassava4.1_005231m|PACid:17968585	cassava4.1_005231m	Class II aminoacyl-tRNA and biotin synthetases superfamily protein	GP
cassava4.1_005232m|PACid:17967368	cassava4.1_005232m	calnexin 1	GP
cassava4.1_005236m|PACid:17976551	cassava4.1_005236m	damaged DNA binding protein 1A	GP
cassava4.1_005238m|PACid:17981965	cassava4.1_005238m	pyrimidine 2	GP
cassava4.1_005241m|PACid:17962290	cassava4.1_005241m	TCP-1/cpn60 chaperonin family protein	GP
cassava4.1_005244m|PACid:17976174	cassava4.1_005244m	fatty acid desaturase 6	GP
cassava4.1_005245m|PACid:17988901	cassava4.1_005245m	cytochrome P450, family 78, subfamily A, polypeptide 7	No
cassava4.1_005247m|PACid:17961150	cassava4.1_005247m	SUMO-activating enzyme 2	No
cassava4.1_005249m|PACid:17963325	cassava4.1_005249m	TCP-1/cpn60 chaperonin family protein	P
cassava4.1_005253m|PACid:17981800	cassava4.1_005253m	Major facilitator superfamily protein	GP
cassava4.1_005258m|PACid:17991443	cassava4.1_005258m	Pheophorbide a oxygenase family protein with Rieske [2Fe-2S] domain	No
cassava4.1_005260m|PACid:17966798	cassava4.1_005260m	calcium-dependent protein kinase 19	No
cassava4.1_005261m|PACid:17989590	cassava4.1_005261m	TCP-1/cpn60 chaperonin family protein	No
cassava4.1_005262m|PACid:17980221	cassava4.1_005262m	3-deoxy-D-arabino-heptulosonate 7-phosphate synthase 1	GP
cassava4.1_005264m|PACid:17993204	cassava4.1_005264m	importin alpha isoform 4	GP
cassava4.1_005265m|PACid:17970196	cassava4.1_005265m	alanine aminotransferase 2	GP
cassava4.1_005266m|PACid:17975050	cassava4.1_005266m	calcium-dependent protein kinase 33	P
cassava4.1_005268m|PACid:17988466	cassava4.1_005268m	DNA polymerase lambda (POLL)	G
cassava4.1_005270m|PACid:17978406	cassava4.1_005270m	glyoxal oxidase-related protein	No
cassava4.1_005271m|PACid:17964521	cassava4.1_005271m	1-amino-cyclopropane-1-carboxylate synthase 12	No
cassava4.1_005272m|PACid:17984647	cassava4.1_005272m	plastid division2	P
cassava4.1_005276m|PACid:17992734	cassava4.1_005276m	Xanthine/uracil permease family protein	No
cassava4.1_005279m|PACid:17991830	cassava4.1_005279m	Xanthine/uracil permease family protein	No
cassava4.1_005280m|PACid:17970581	cassava4.1_005280m	3-ketoacyl-CoA synthase 10	G
cassava4.1_005282m|PACid:17962598	cassava4.1_005282m	Glycinamide ribonucleotide (GAR) synthetase	GP
cassava4.1_005284m|PACid:17992236	cassava4.1_005284m		G
cassava4.1_005287m|PACid:17987822	cassava4.1_005287m	galacturonosyltransferase 12	G
cassava4.1_005292m|PACid:17987881	cassava4.1_005292m	transducin family protein / WD-40 repeat family protein	No
cassava4.1_005293m|PACid:17983106	cassava4.1_005293m	serine hydroxymethyltransferase 3	GP
cassava4.1_005294m|PACid:17962872	cassava4.1_005294m	FAD/NAD(P)-binding oxidoreductase family protein	G
cassava4.1_005295m|PACid:17961422	cassava4.1_005295m	Ankyrin repeat family protein	G
cassava4.1_005298m|PACid:17975871	cassava4.1_005298m	galacturonosyltransferase 10	No
cassava4.1_005299m|PACid:17961635	cassava4.1_005299m	DNA polymerase epsilon subunit B2	G
cassava4.1_005303m|PACid:17990349	cassava4.1_005303m	importin alpha isoform 1	P
cassava4.1_005305m|PACid:17983701	cassava4.1_005305m	PDI-like 1-5	GP

cassava4.1_005306m|PACid:17969675	cassava4.1_005306m	purple acid phosphatase 10	GP
cassava4.1_005309m|PACid:17992456	cassava4.1_005309m	TCP-1/cpn60 chaperonin family protein	P
cassava4.1_005319m|PACid:17961064	cassava4.1_005319m	Nucleotide-diphospho-sugar transferases superfamily protein	No
cassava4.1_005320m|PACid:17971020	cassava4.1_005320m	calcium-dependent protein kinase 6	GP
cassava4.1_005322m|PACid:17990563	cassava4.1_005322m	alpha/beta-Hydrolases superfamily protein	G
cassava4.1_005323m|PACid:17987801	cassava4.1_005323m	calmodulin-domain protein kinase 9	GP
cassava4.1_005326m|PACid:17976506	cassava4.1_005326m	Cysteinyl-tRNA synthetase, class Ia family protein	GP
cassava4.1_005327m|PACid:17990716	cassava4.1_005327m	membrane bound O-acyl transferase (MBOAT) family protein	No
cassava4.1_005328m|PACid:17971662	cassava4.1_005328m	Pentatricopeptide repeat (PPR) superfamily protein	G
cassava4.1_005329m|PACid:17966766	cassava4.1_005329m	NAD kinase 1	G
cassava4.1_005339m|PACid:17964155	cassava4.1_005339m	aldehyde dehydrogenase 5F1	No
cassava4.1_005343m|PACid:17988042	cassava4.1_005343m	associated molecule with the SH3 domain of STAM 1	G
cassava4.1_005345m|PACid:17980235	cassava4.1_005345m	calcium-dependent protein kinase 32	G
cassava4.1_005353m|PACid:17964418	cassava4.1_005353m	cytochrome P450, family 82, subfamily C, polypeptide 4	No
cassava4.1_005355m|PACid:17990686	cassava4.1_005355m	Pyridoxal-5\'-phosphate-dependent enzyme family protein	GP
cassava4.1_005358m|PACid:17988842	cassava4.1_005358m	ALG6, ALG8 glycosyltransferase family	G
cassava4.1_005360m|PACid:17982048	cassava4.1_005360m	plant glycogenin-like starch initiation protein 6	No
cassava4.1_005362m|PACid:17973852	cassava4.1_005362m	importin alpha isoform 1	No
cassava4.1_005364m|PACid:17985592	cassava4.1_005364m	transcription activators;DNA binding;RNA polymerase II transcription factors;catalytics;transcription initiation factors	GP
cassava4.1_005367m|PACid:17993272	cassava4.1_005367m	importin alpha isoform 1	No
cassava4.1_005369m|PACid:17979680	cassava4.1_005369m	importin alpha isoform 2	GP
cassava4.1_005372m|PACid:17978084	cassava4.1_005372m	Transducin/WD40 repeat-like superfamily protein	No
cassava4.1_005375m|PACid:17993400	cassava4.1_005375m	Peptidase S41 family protein	GP
cassava4.1_005383m|PACid:17969148	cassava4.1_005383m	cytochrome P450, family 78, subfamily A, polypeptide 10	G
cassava4.1_005384m|PACid:17982739	cassava4.1_005384m	Tetratricopeptide repeat (TPR)-like superfamily protein	GP
cassava4.1_005385m|PACid:17991799	cassava4.1_005385m	fucosyltransferase 12	G
cassava4.1_005386m|PACid:17961493	cassava4.1_005386m	Pentatricopeptide repeat (PPR) superfamily protein	No
cassava4.1_005392m|PACid:17975950	cassava4.1_005392m	heat shock protein 60-3A	GP
cassava4.1_005397m|PACid:17959794	cassava4.1_005397m	monogalactosyl diacylglycerol synthase 1	GP
cassava4.1_005401m|PACid:17994161	cassava4.1_005401m	Nucleotide/sugar transporter family protein	No
cassava4.1_005403m|PACid:17977111	cassava4.1_005403m	Ribonuclease III family protein	GP
cassava4.1_005404m|PACid:17990154	cassava4.1_005404m	phosphate transporter 1;9	No
cassava4.1_005410m|PACid:17987132	cassava4.1_005410m	haloacid dehalogenase-like hydrolase family protein	P
cassava4.1_005412m|PACid:17979403	cassava4.1_005412m	clathrin adaptor complexes medium subunit family protein	No
cassava4.1_005413m|PACid:17986025	cassava4.1_005413m	thymidylate synthase 1	GP
cassava4.1_005415m|PACid:17978632	cassava4.1_005415m	aspartate kinase 3	No
cassava4.1_005418m|PACid:17961045	cassava4.1_005418m	Pyruvate kinase family protein	GP
cassava4.1_005423m|PACid:17993552	cassava4.1_005423m	MOS4-associated complex 3A	GP
cassava4.1_005429m|PACid:17975591	cassava4.1_005429m	cytochrome P450, family 78, subfamily A, polypeptide 6	G

cassava4.1_005430m|PACid:17985060	cassava4.1_005430m	Regulator of chromosome condensation (RCC1) family protein	GP
cassava4.1_005434m|PACid:17983546	cassava4.1_005434m	Insulinase (Peptidase family M16) protein	GP
cassava4.1_005436m|PACid:17988945	cassava4.1_005436m	Eukaryotic aspartyl protease family protein	G
cassava4.1_005437m|PACid:17968443	cassava4.1_005437m	Glucose-1-phosphate adenylyltransferase family protein	No
cassava4.1_005438m|PACid:17985283	cassava4.1_005438m	phosphofructokinase 4	P
cassava4.1_005439m|PACid:17973579	cassava4.1_005439m	TCP-1/cpn60 chaperonin family protein	GP
cassava4.1_005442m|PACid:17964957	cassava4.1_005442m	Major facilitator superfamily protein	G
cassava4.1_005443m|PACid:17964963	cassava4.1_005443m	Xanthine/uracil permease family protein	No
cassava4.1_005445m|PACid:17960911	cassava4.1_005445m	cytochrome P450, family 714, subfamily A, polypeptide 1	G
cassava4.1_005448m|PACid:17976121	cassava4.1_005448m	Zn-dependent exopeptidases superfamily protein	G
cassava4.1_005450m|PACid:17964598	cassava4.1_005450m	Plant invertase/pectin methylesterase inhibitor superfamily	No
cassava4.1_005453m|PACid:17964849	cassava4.1_005453m	Protein of unknown function, DUF647	G
cassava4.1_005455m|PACid:17970236	cassava4.1_005455m	Protein kinase family protein	G
cassava4.1_005456m|PACid:17967604	cassava4.1_005456m	NOL1/NOP2/sun family protein / antitermination NusB domain-containing protein	GP
cassava4.1_005459m|PACid:17983822	cassava4.1_005459m	GPI transamidase component PIG-S-related	No
cassava4.1_005462m|PACid:17987663	cassava4.1_005462m	Pentatricopeptide repeat (PPR) superfamily protein	No
cassava4.1_005467m|PACid:17963437	cassava4.1_005467m	Protein phosphatase 2A regulatory B subunit family protein	G
cassava4.1_005468m|PACid:17987918	cassava4.1_005468m	alpha/beta-Hydrolases superfamily protein	GP
cassava4.1_005480m|PACid:17982103	cassava4.1_005480m	NADPH-dependent thioredoxin reductase C	GP
cassava4.1_005483m|PACid:17984967	cassava4.1_005483m	Tetratricopeptide repeat (TPR)-like superfamily protein	No
cassava4.1_005484m|PACid:17990192	cassava4.1_005484m	Pseudouridine synthase family protein	G
cassava4.1_005488m|PACid:17985340	cassava4.1_005488m	Mitochondrial import inner membrane translocase subunit Tim17/Tim22/Tim23 family protein	G
cassava4.1_005489m|PACid:17983928	cassava4.1_005489m	Xanthine/uracil permease family protein	No
cassava4.1_005492m|PACid:17990833	cassava4.1_005492m	sugar transporter 1	No
cassava4.1_005494m|PACid:17993003	cassava4.1_005494m	3\'-5\' exonuclease domain-containing protein	G
cassava4.1_005496m|PACid:17983611	cassava4.1_005496m	NAD(P)-binding Rossmann-fold superfamily protein	No
cassava4.1_005503m|PACid:17973142	cassava4.1_005503m	homology to ABI1	G
cassava4.1_005505m|PACid:17985139	cassava4.1_005505m	Protein phosphatase 2A regulatory B subunit family protein	No
cassava4.1_005506m|PACid:17978353	cassava4.1_005506m	Pentatricopeptide repeat (PPR) superfamily protein	No
cassava4.1_005508m|PACid:17968408	cassava4.1_005508m	Pre-mRNA splicing Prp18-interacting factor	No
cassava4.1_005510m|PACid:17970580	cassava4.1_005510m	cytochrome P450, family 711, subfamily A, polypeptide 1	No
cassava4.1_005513m|PACid:17971040	cassava4.1_005513m	glutamate-cysteine ligase	GP
cassava4.1_005514m|PACid:17960761	cassava4.1_005514m	AMP-dependent synthetase and ligase family protein	No
cassava4.1_005517m|PACid:17969902	cassava4.1_005517m	glycosyl hydrolase 9B7	GP
cassava4.1_005518m|PACid:17993865	cassava4.1_005518m	ADP glucose pyrophosphorylase 1	GP
cassava4.1_005521m|PACid:17961888	cassava4.1_005521m	3-ketoacyl-CoA synthase 2	No
cassava4.1_005528m|PACid:17968010	cassava4.1_005528m	Histone H3 K4-specific methyltransferase SET7/9 family protein	No
cassava4.1_005529m|PACid:17992193	cassava4.1_005529m	Major facilitator superfamily protein	G
cassava4.1_005532m|PACid:17967511	cassava4.1_005532m	beta-amylase 4	No

cassava4.1_005533m|PACid:17974745	cassava4.1_005533m	Y-family DNA polymerase H	No
cassava4.1_005535m|PACid:17991934	cassava4.1_005535m	Cell division control, Cdc6	No
cassava4.1_005536m|PACid:17985286	cassava4.1_005536m	phosphate starvation-induced gene 3	G
cassava4.1_005538m|PACid:17981938	cassava4.1_005538m	non-specific phospholipase C2	GP
cassava4.1_005539m|PACid:17988743	cassava4.1_005539m	RNA 3\'-terminal phosphate cyclase/enolpyruvate transferase, alpha/beta	GP
cassava4.1_005541m|PACid:17989644	cassava4.1_005541m	ALG6, ALG8 glycosyltransferase family	No
cassava4.1_005543m|PACid:17976035	cassava4.1_005543m	Pyridoxal-5\'-phosphate-dependent enzyme family protein	GP
cassava4.1_005545m|PACid:17987789	cassava4.1_005545m		GP
cassava4.1_005551m|PACid:17992023	cassava4.1_005551m	pheophytinase	GP
cassava4.1_005557m|PACid:17982818	cassava4.1_005557m	S-adenosyl-L-methionine-dependent methyltransferases superfamily protein	G
cassava4.1_005559m|PACid:17970600	cassava4.1_005559m	AGC (cAMP-dependent, cGMP-dependent and protein kinase C) kinase family protein	No
cassava4.1_005562m|PACid:17966555	cassava4.1_005562m	beta-amylase 5	No
cassava4.1_005564m|PACid:17982974	cassava4.1_005564m	P-loop containing nucleoside triphosphate hydrolases superfamily protein	GP
cassava4.1_005572m|PACid:17960705	cassava4.1_005572m	Domain of unknown function (DUF23)	G
cassava4.1_005578m|PACid:17966522	cassava4.1_005578m	plant-specific TFIIB-related protein	No
cassava4.1_005582m|PACid:17962585	cassava4.1_005582m	Lung seven transmembrane receptor family protein	G
cassava4.1_005586m|PACid:17985423	cassava4.1_005586m	RING/U-box superfamily protein	G
cassava4.1_005588m|PACid:17989408	cassava4.1_005588m	RNA-binding KH domain-containing protein	GP
cassava4.1_005595m|PACid:17983875	cassava4.1_005595m	Pentatricopeptide repeat (PPR) superfamily protein	No
cassava4.1_005597m|PACid:17984257	cassava4.1_005597m	Protein kinase superfamily protein	G
cassava4.1_005600m|PACid:17969562	cassava4.1_005600m	Transcription elongation factor (TFIIS) family protein	GP
cassava4.1_005619m|PACid:17992837	cassava4.1_005619m	Pectin lyase-like superfamily protein	No
cassava4.1_005627m|PACid:17970952	cassava4.1_005627m	Protein of unknown function (DUF604)	No
cassava4.1_005629m|PACid:17970498	cassava4.1_005629m	SERINE CARBOXYPEPTIDASE-LIKE 49	G
cassava4.1_005634m|PACid:17976937	cassava4.1_005634m	3-ketoacyl-CoA synthase 4	G
cassava4.1_005636m|PACid:17990570	cassava4.1_005636m	importin alpha isoform 9	GP
cassava4.1_005637m|PACid:17963542	cassava4.1_005637m	P-loop containing nucleoside triphosphate hydrolases superfamily protein	No
cassava4.1_005639m|PACid:17977151	cassava4.1_005639m	mitogen-activated protein kinase kinase 3	G
cassava4.1_005642m|PACid:17991187	cassava4.1_005642m	bidirectional amino acid transporter 1	G
cassava4.1_005643m|PACid:17963832	cassava4.1_005643m	sulfoquinovosyldiacylglycerol 2	No
cassava4.1_005644m|PACid:17970379	cassava4.1_005644m	RNA helicase 1	No
cassava4.1_005646m|PACid:17965442	cassava4.1_005646m	RING/FYVE/PHD-type zinc finger family protein	G
cassava4.1_005652m|PACid:17972360	cassava4.1_005652m	Leucine-rich repeat (LRR) family protein	No
cassava4.1_005653m|PACid:17987398	cassava4.1_005653m	cytochrome P450, family 735, subfamily A, polypeptide 1	No
cassava4.1_005656m|PACid:17970588	cassava4.1_005656m	pectin methylesterase 44	No
cassava4.1_005659m|PACid:17974452	cassava4.1_005659m	FRIGIDA-like protein	G
cassava4.1_005662m|PACid:17986053	cassava4.1_005662m	aluminum-activated, malate transporter 12	G
cassava4.1_005663m|PACid:17985259	cassava4.1_005663m	lipid transporters	G
cassava4.1_005665m|PACid:17975397	cassava4.1_005665m	P-loop containing nucleoside triphosphate hydrolases superfamily protein	No

cassava4.1_005674m|PACid:17969337	cassava4.1_005674m	cytochrome P450, family 87, subfamily A, polypeptide 6	No
cassava4.1_005676m|PACid:17976066	cassava4.1_005676m	tRNAHis guanylyltransferase	G
cassava4.1_005677m|PACid:17992938	cassava4.1_005677m	beta-glucosidase 45	G
cassava4.1_005678m|PACid:17972172	cassava4.1_005678m	apurinic endonuclease-redox protein	G
cassava4.1_005679m|PACid:17968214	cassava4.1_005679m	phosphoenolpyruvate carboxylase-related kinase 1	No
cassava4.1_005682m|PACid:17968526	cassava4.1_005682m	serine hydroxymethyltransferase 2	GP
cassava4.1_005683m|PACid:17983977	cassava4.1_005683m	Protein of unknown function (DUF604)	G
cassava4.1_005685m|PACid:17963567	cassava4.1_005685m	Guanylate-binding family protein	G
cassava4.1_005687m|PACid:17973636	cassava4.1_005687m	cyclin-dependent kinase C;1	No
cassava4.1_005690m|PACid:17965238	cassava4.1_005690m	glucose-6-phosphate dehydrogenase 6	GP
cassava4.1_005692m|PACid:17976781	cassava4.1_005692m	Na+/H+ antiporter 6	G
cassava4.1_005693m|PACid:17963695	cassava4.1_005693m	cytochrome P450, family 709, subfamily B, polypeptide 2	No
cassava4.1_005697m|PACid:17993975	cassava4.1_005697m	natural resistance-associated macrophage protein 3	No
cassava4.1_005700m|PACid:17978764	cassava4.1_005700m	SNF1 kinase homolog 10	No
cassava4.1_005702m|PACid:17962794	cassava4.1_005702m	Cytochrome P450 superfamily protein	P
cassava4.1_005703m|PACid:17977311	cassava4.1_005703m	Pyridoxal phosphate (PLP)-dependent transferases superfamily protein	GP
cassava4.1_005705m|PACid:17978158	cassava4.1_005705m	Pentatricopeptide repeat (PPR) superfamily protein	G
cassava4.1_005706m|PACid:17973323	cassava4.1_005706m	Protein phosphatase 2A, regulatory subunit PR55	GP
cassava4.1_005708m|PACid:17972709	cassava4.1_005708m	sugar transporter protein 7	G
cassava4.1_005710m|PACid:17989829	cassava4.1_005710m	serine transhydroxymethyltransferase 1	GP
cassava4.1_005712m|PACid:17980116	cassava4.1_005712m	SNF1 kinase homolog 10	G
cassava4.1_005724m|PACid:17965518	cassava4.1_005724m	Major facilitator superfamily protein	G
cassava4.1_005730m|PACid:17965481	cassava4.1_005730m	Translation elongation factor EF1A/initiation factor IF2gamma family protein	GP
cassava4.1_005740m|PACid:17965223	cassava4.1_005740m	Rubisco methyltransferase family protein	G
cassava4.1_005742m|PACid:17965052	cassava4.1_005742m	non-specific phospholipase C6	No
cassava4.1_005744m|PACid:17985811	cassava4.1_005744m	aldehyde dehydrogenase 2C4	GP
cassava4.1_005748m|PACid:17981798	cassava4.1_005748m	O-fucosyltransferase family protein	G
cassava4.1_005749m|PACid:17970372	cassava4.1_005749m	Protein of unknown function (DUF1712)	G
cassava4.1_005750m|PACid:17982415	cassava4.1_005750m	FAD/NAD(P)-binding oxidoreductase family protein	No
cassava4.1_005751m|PACid:17970549	cassava4.1_005751m	S-adenosyl-L-methionine-dependent methyltransferases superfamily protein	G
cassava4.1_005757m|PACid:17988169	cassava4.1_005757m	Protein of unknown function (DUF668)	G
cassava4.1_005758m|PACid:17966111	cassava4.1_005758m	Lung seven transmembrane receptor family protein	No
cassava4.1_005761m|PACid:17988198	cassava4.1_005761m	Mitochondrial transcription termination factor family protein	No
cassava4.1_005764m|PACid:17989611	cassava4.1_005764m	Protein kinase superfamily protein	No
cassava4.1_005767m|PACid:17960801	cassava4.1_005767m	XB3 ortholog 3 in Arabidopsis thaliana	No
cassava4.1_005770m|PACid:17978587	cassava4.1_005770m	AUTOPHAGY 6	G
cassava4.1_005774m|PACid:17976498	cassava4.1_005774m	quiescin-sulfhydryl oxidase 2	GP
cassava4.1_005782m|PACid:17960266	cassava4.1_005782m	transducin family protein / WD-40 repeat family protein	GP
cassava4.1_005789m|PACid:17984906	cassava4.1_005789m	FAD-binding Berberine family protein	GP

cassava4.1_005792m|PACid:17963244	cassava4.1_005792m	cytochrome P450, family 704, subfamily A, polypeptide 2	G
cassava4.1_005795m|PACid:17985897	cassava4.1_005795m	MATE efflux family protein	No
cassava4.1_005796m|PACid:17971961	cassava4.1_005796m	embryo sac development arrest 7	No
cassava4.1_005797m|PACid:17979700	cassava4.1_005797m	associated molecule with the SH3 domain of STAM 3	G
cassava4.1_005798m|PACid:17962721	cassava4.1_005798m	Pentatricopeptide repeat (PPR) superfamily protein	G
cassava4.1_005799m|PACid:17959923	cassava4.1_005799m	ARM repeat superfamily protein	No
cassava4.1_005802m|PACid:17971132	cassava4.1_005802m	monodehydroascorbate reductase 4	GP
cassava4.1_005803m|PACid:17983730	cassava4.1_005803m	RHO guanyl-nucleotide exchange factor 12	No
cassava4.1_005810m|PACid:17980955	cassava4.1_005810m	BTB/POZ domain-containing protein	No
cassava4.1_005818m|PACid:17991964	cassava4.1_005818m	cytochrome P450, family 86, subfamily A, polypeptide 1	No
cassava4.1_005819m|PACid:17976217	cassava4.1_005819m	Calcium-dependent lipid-binding (CaLB domain) family protein	GP
cassava4.1_005823m|PACid:17979495	cassava4.1_005823m	citrate synthase 2	No
cassava4.1_005826m|PACid:17975697	cassava4.1_005826m	Major facilitator superfamily protein	No
cassava4.1_005829m|PACid:17982056	cassava4.1_005829m	ammonium transporter 1;2	No
cassava4.1_005830m|PACid:17986346	cassava4.1_005830m	RAN GTPase activating protein 1	No
cassava4.1_005831m|PACid:17979602	cassava4.1_005831m	3-ketoacyl-CoA synthase 11	G
cassava4.1_005834m|PACid:17979683	cassava4.1_005834m	2-oxoacid dehydrogenases acyltransferase family protein	GP
cassava4.1_005836m|PACid:17986201	cassava4.1_005836m	alpha/beta-Hydrolases superfamily protein	No
cassava4.1_005838m|PACid:17960369	cassava4.1_005838m	myo-inositol-1-phosphate synthase 2	GP
cassava4.1_005840m|PACid:17979299	cassava4.1_005840m	nonsense-mediated mRNA decay NMD3 family protein	GP
cassava4.1_005841m|PACid:17964409	cassava4.1_005841m	GA requiring 3	No
cassava4.1_005843m|PACid:17979483	cassava4.1_005843m	U1 small nuclear ribonucleoprotein-70K	GP
cassava4.1_005844m|PACid:17965206	cassava4.1_005844m	Flavin containing amine oxidoreductase family	GP
cassava4.1_005850m|PACid:17973145	cassava4.1_005850m	Pyruvate kinase family protein	GP
cassava4.1_005851m|PACid:17972116	cassava4.1_005851m	calmodulin-binding family protein	G
cassava4.1_005856m|PACid:17982643	cassava4.1_005856m	mitochondrial lipoamide dehydrogenase 1	GP
cassava4.1_005857m|PACid:17988860	cassava4.1_005857m	myo-inositol-1-phosphate synthase 3	GP
cassava4.1_005859m|PACid:17993468	cassava4.1_005859m	isopropyl malate isomerase large subunit 1	GP
cassava4.1_005860m|PACid:17973132	cassava4.1_005860m	Lung seven transmembrane receptor family protein	No
cassava4.1_005861m|PACid:17978756	cassava4.1_005861m	Nucleotide-diphospho-sugar transferases superfamily protein	G
cassava4.1_005865m|PACid:17965753	cassava4.1_005865m	galacturonosyltransferase 7	No
cassava4.1_005873m|PACid:17960823	cassava4.1_005873m	cytochrome P450, family 715, subfamily A, polypeptide 1	No
cassava4.1_005880m|PACid:17978660	cassava4.1_005880m	phosphoadenosine phosphosulfate (PAPS) reductase family protein	G
cassava4.1_005887m|PACid:17970015	cassava4.1_005887m	DIE2/ALG10 family	G
cassava4.1_005889m|PACid:17990002	cassava4.1_005889m	splicing factor-related	No
cassava4.1_005890m|PACid:17966170	cassava4.1_005890m	B-S glucosidase 44	GP
cassava4.1_005891m|PACid:17970219	cassava4.1_005891m	pentatricopeptide (PPR) repeat-containing protein	G
cassava4.1_005893m|PACid:17966113	cassava4.1_005893m	Pyruvate kinase family protein	GP
cassava4.1_005894m|PACid:17965974	cassava4.1_005894m	sigma factor A	No

cassava4.1_005896m|PACid:17972990	cassava4.1_005896m	aldehyde dehydrogenase 7B4	G
cassava4.1_005903m|PACid:17993221	cassava4.1_005903m	Protein kinase superfamily protein	No
cassava4.1_005907m|PACid:17971270	cassava4.1_005907m	hexokinase-like 1	P
cassava4.1_005908m|PACid:17977210	cassava4.1_005908m	MATE efflux family protein	No
cassava4.1_005909m|PACid:17971474	cassava4.1_005909m	Protein kinase superfamily protein	G
cassava4.1_005910m|PACid:17972825	cassava4.1_005910m	cytochrome P450, family 98, subfamily A, polypeptide 3	GP
cassava4.1_005913m|PACid:17992503	cassava4.1_005913m	O-fucosyltransferase family protein	G
cassava4.1_005917m|PACid:17964549	cassava4.1_005917m	Major facilitator superfamily protein	G
cassava4.1_005923m|PACid:17967600	cassava4.1_005923m	Mitochondrial transcription termination factor family protein	G
cassava4.1_005927m|PACid:17977435	cassava4.1_005927m	Heavy metal transport/detoxification superfamily protein	No
cassava4.1_005932m|PACid:17992568	cassava4.1_005932m	MATE efflux family protein	G
cassava4.1_005933m|PACid:17977749	cassava4.1_005933m	MATE efflux family protein	No
cassava4.1_005939m|PACid:17993710	cassava4.1_005939m	cytochrome P450, family 712, subfamily A, polypeptide 1	No
cassava4.1_005942m|PACid:17963906	cassava4.1_005942m	serine carboxypeptidase-like 48	No
cassava4.1_005945m|PACid:17961106	cassava4.1_005945m	Rho GTPase activating protein with PAK-box/P21-Rho-binding domain	G
cassava4.1_005952m|PACid:17966808	cassava4.1_005952m	hexokinase-like 1	GP
cassava4.1_005953m|PACid:17981682	cassava4.1_005953m	glycosyl hydrolase 9B13	GP
cassava4.1_005958m|PACid:17991533	cassava4.1_005958m	Saposin-like aspartyl protease family protein	GP
cassava4.1_005959m|PACid:17982124	cassava4.1_005959m	homology to ABI1	No
cassava4.1_005964m|PACid:17969876	cassava4.1_005964m	methyltransferases;copper ion binding	G
cassava4.1_005968m|PACid:17964195	cassava4.1_005968m	O-Glycosyl hydrolases family 17 protein	No
cassava4.1_005970m|PACid:17987533	cassava4.1_005970m	O-Glycosyl hydrolases family 17 protein	No
cassava4.1_005973m|PACid:17990643	cassava4.1_005973m	Metallopeptidase M24 family protein	G
cassava4.1_005975m|PACid:17969126	cassava4.1_005975m	Pentatricopeptide repeat (PPR) superfamily protein	No
cassava4.1_005977m|PACid:17992730	cassava4.1_005977m	endoplasmic reticulum-type calcium-transporting ATPase 3	G
cassava4.1_005978m|PACid:17970708	cassava4.1_005978m	cinnamate-4-hydroxylase	GP
cassava4.1_005980m|PACid:17981871	cassava4.1_005980m	MATE efflux family protein	No
cassava4.1_005983m|PACid:17986386	cassava4.1_005983m	glycerol-3-phosphate acyltransferase 6	GP
cassava4.1_005985m|PACid:17988441	cassava4.1_005985m	glycine-rich protein	G
cassava4.1_005986m|PACid:17964204	cassava4.1_005986m	CDK-activating kinase 1AT	G
cassava4.1_005988m|PACid:17962259	cassava4.1_005988m	Cysteine proteinases superfamily protein	G
cassava4.1_005989m|PACid:17975982	cassava4.1_005989m	DEA(D/H)-box RNA helicase family protein	GP
cassava4.1_005995m|PACid:17963183	cassava4.1_005995m	O-fucosyltransferase family protein	No
cassava4.1_005998m|PACid:17989237	cassava4.1_005998m	Rubisco methyltransferase family protein	No
cassava4.1_006009m|PACid:17972992	cassava4.1_006009m	Insulinase (Peptidase family M16) protein	GP
cassava4.1_006011m|PACid:17977820	cassava4.1_006011m	Major facilitator superfamily protein	G
cassava4.1_006012m|PACid:17978888	cassava4.1_006012m	3-phosphoinositide-dependent protein kinase	No
cassava4.1_006013m|PACid:17976987	cassava4.1_006013m	Pyruvate kinase family protein	P
cassava4.1_006014m|PACid:17966029	cassava4.1_006014m	RING/U-box superfamily protein	G

cassava4.1_006015m|PACid:17982617	cassava4.1_006015m	serine carboxypeptidase-like 20	GP
cassava4.1_006018m|PACid:17979387	cassava4.1_006018m	ZPR1 zinc-finger domain protein	GP
cassava4.1_006024m|PACid:17974163	cassava4.1_006024m	aldehyde dehydrogenase 10A8	GP
cassava4.1_006026m|PACid:17967898	cassava4.1_006026m	calcium-dependent protein kinase 2	GP
cassava4.1_006032m|PACid:17988168	cassava4.1_006032m	MATE efflux family protein	No
cassava4.1_006033m|PACid:17971522	cassava4.1_006033m	diphthamide synthesis DPH2 family protein	G
cassava4.1_006035m|PACid:17964201	cassava4.1_006035m	Major facilitator superfamily protein	G
cassava4.1_006036m|PACid:17968341	cassava4.1_006036m	phytochelatin synthase 1 (PCS1)	No
cassava4.1_006038m|PACid:17975109	cassava4.1_006038m	Major facilitator superfamily protein	No
cassava4.1_006039m|PACid:17966024	cassava4.1_006039m	Protein kinase superfamily protein	GP
cassava4.1_006041m|PACid:17980717	cassava4.1_006041m	DA1-related protein 2	G
cassava4.1_006042m|PACid:17965050	cassava4.1_006042m	Mitochondrial substrate carrier family protein	GP
cassava4.1_006046m|PACid:17981057	cassava4.1_006046m	Amino acid permease family protein	G
cassava4.1_006050m|PACid:17969255	cassava4.1_006050m	Ribosomal protein S5 family protein	GP
cassava4.1_006052m|PACid:17976900	cassava4.1_006052m	cytochrome P450, family 94, subfamily B, polypeptide 2	No
cassava4.1_006055m|PACid:17962153	cassava4.1_006055m	CBS domain-containing protein with a domain of unknown function (DUF21)	GP
cassava4.1_006057m|PACid:17986405	cassava4.1_006057m	fructokinase-like 1	GP
cassava4.1_006061m|PACid:17979272	cassava4.1_006061m	2-oxoglutarate (2OG) and Fe(II)-dependent oxygenase superfamily protein	G
cassava4.1_006063m|PACid:17973476	cassava4.1_006063m	phosphoglycerate/bisphosphoglycerate mutase family protein	P
cassava4.1_006064m|PACid:17966465	cassava4.1_006064m	alternative NAD(P)H dehydrogenase 1	GP
cassava4.1_006067m|PACid:17980561	cassava4.1_006067m	Nucleotide-diphospho-sugar transferases superfamily protein	G
cassava4.1_006068m|PACid:17990845	cassava4.1_006068m	allantoinase	No
cassava4.1_006070m|PACid:17971260	cassava4.1_006070m	transferases;tRNA (5-methylaminomethyl-2-thiouridylate)-methyltransferases	G
cassava4.1_006073m|PACid:17990007	cassava4.1_006073m	sigma factor E	No
cassava4.1_006081m|PACid:17993102	cassava4.1_006081m	Major facilitator superfamily protein	G
cassava4.1_006083m|PACid:17971395	cassava4.1_006083m	calcium-dependent protein kinase 2	GP
cassava4.1_006084m|PACid:17984957	cassava4.1_006084m	Sec14p-like phosphatidylinositol transfer family protein	No
cassava4.1_006089m|PACid:17969239	cassava4.1_006089m	Major facilitator superfamily protein	G
cassava4.1_006090m|PACid:17987370	cassava4.1_006090m	P-loop containing nucleoside triphosphate hydrolases superfamily protein	GP
cassava4.1_006093m|PACid:17978475	cassava4.1_006093m	glycerol-3-phosphate acyltransferase 8	GP
cassava4.1_006095m|PACid:17977771	cassava4.1_006095m	Aldolase-type TIM barrel family protein	GP
cassava4.1_006097m|PACid:17968971	cassava4.1_006097m	Plasma-membrane choline transporter family protein	G
cassava4.1_006098m|PACid:17967014	cassava4.1_006098m	cytochrome P450, family 81, subfamily D, polypeptide 3	G
cassava4.1_006101m|PACid:17993838	cassava4.1_006101m	hydroperoxide lyase 1	GP
cassava4.1_006108m|PACid:17977765	cassava4.1_006108m	Major facilitator superfamily protein	No
cassava4.1_006109m|PACid:17991358	cassava4.1_006109m	PDI-like 1-1	GP
cassava4.1_006117m|PACid:17975858	cassava4.1_006117m	CBL-interacting protein kinase 12	G
cassava4.1_006122m|PACid:17982414	cassava4.1_006122m	O-fucosyltransferase family protein	GP
cassava4.1_006123m|PACid:17982657	cassava4.1_006123m	Mevalonate/galactokinase family protein	GP

cassava4.1_006124m|PACid:17988918	cassava4.1_006124m	Cation efflux family protein	G
cassava4.1_006126m|PACid:17983492	cassava4.1_006126m	glutamate decarboxylase 5	P
cassava4.1_006130m|PACid:17968039	cassava4.1_006130m	histone deacetylase 1	GP
cassava4.1_006133m|PACid:17979481	cassava4.1_006133m	Pleckstrin homology (PH) domain-containing protein	GP
cassava4.1_006134m|PACid:17963779	cassava4.1_006134m	Class II aaRS and biotin synthetases superfamily protein	GP
cassava4.1_006136m|PACid:17991171	cassava4.1_006136m	MATE efflux family protein	G
cassava4.1_006137m|PACid:17985260	cassava4.1_006137m	FK506 BINDING PROTEIN 53	No
cassava4.1_006138m|PACid:17985703	cassava4.1_006138m	hexokinase 1	GP
cassava4.1_006139m|PACid:17968274	cassava4.1_006139m	RNA helicase 36	No
cassava4.1_006140m|PACid:17972634	cassava4.1_006140m	MAP kinase 15	G
cassava4.1_006143m|PACid:17988283	cassava4.1_006143m	inositol transporter 1	G
cassava4.1_006145m|PACid:17988640	cassava4.1_006145m	BR-signaling kinase 2	No
cassava4.1_006146m|PACid:17967773	cassava4.1_006146m	O-Glycosyl hydrolases family 17 protein	No
cassava4.1_006148m|PACid:17970817	cassava4.1_006148m	BR-signaling kinase 1	GP
cassava4.1_006156m|PACid:17978704	cassava4.1_006156m	S-adenosyl-L-methionine-dependent methyltransferases superfamily protein	No
cassava4.1_006157m|PACid:17971244	cassava4.1_006157m	MATE efflux family protein	No
cassava4.1_006159m|PACid:17969879	cassava4.1_006159m	Protein phosphatase 2A regulatory B subunit family protein	No
cassava4.1_006165m|PACid:17985625	cassava4.1_006165m	Eukaryotic aspartyl protease family protein	GP
cassava4.1_006168m|PACid:17983198	cassava4.1_006168m	histidine acid phosphatase family protein	GP
cassava4.1_006175m|PACid:17969712	cassava4.1_006175m	glycosyl hydrolase 9B1	G
cassava4.1_006176m|PACid:17985946	cassava4.1_006176m	calcium-binding EF hand family protein	G
cassava4.1_006180m|PACid:17985050	cassava4.1_006180m	O-Glycosyl hydrolases family 17 protein	No
cassava4.1_006182m|PACid:17975310	cassava4.1_006182m	zinc finger (C3HC4-type RING finger) family protein	GP
cassava4.1_006183m|PACid:17969799	cassava4.1_006183m	aldehyde dehydrogenase 11A3	GP
cassava4.1_006185m|PACid:17975266	cassava4.1_006185m	Transmembrane amino acid transporter family protein	G
cassava4.1_006187m|PACid:17966563	cassava4.1_006187m	Transducin/WD40 repeat-like superfamily protein	No
cassava4.1_006188m|PACid:17967534	cassava4.1_006188m	glutathione-disulfide reductase	GP
cassava4.1_006189m|PACid:17962382	cassava4.1_006189m	O-Glycosyl hydrolases family 17 protein	P
cassava4.1_006196m|PACid:17961650	cassava4.1_006196m	glutamate decarboxylase	GP
cassava4.1_006200m|PACid:17989121	cassava4.1_006200m	alpha/beta-Hydrolases superfamily protein	G
cassava4.1_006204m|PACid:17967105	cassava4.1_006204m	ent-kaurenoic acid hydroxylase 2	No
cassava4.1_006207m|PACid:17973627	cassava4.1_006207m	Transducin/WD40 repeat-like superfamily protein	No
cassava4.1_006208m|PACid:17969208	cassava4.1_006208m	3-ketoacyl-CoA synthase 6	GP
cassava4.1_006212m|PACid:17976190	cassava4.1_006212m	aspartate/glutamate/uridylate kinase family protein	No
cassava4.1_006214m|PACid:17961615	cassava4.1_006214m	Protein phosphatase 2C family protein	No
cassava4.1_006215m|PACid:17975642	cassava4.1_006215m	Pectin lyase-like superfamily protein	GP
cassava4.1_006216m|PACid:17979158	cassava4.1_006216m	Homeodomain-like superfamily protein	No
cassava4.1_006217m|PACid:17980423	cassava4.1_006217m	Histidyl-tRNA synthetase 1	No
cassava4.1_006221m|PACid:17968750	cassava4.1_006221m	magnesium transporter 4	G

cassava4.1_006222m|PACid:17985868	cassava4.1_006222m	zinc finger (CCCH-type) family protein / D111/G-patch domain-containing protein	G
cassava4.1_006224m|PACid:17983335	cassava4.1_006224m	Signal recognition particle, SRP54 subunit protein	GP
cassava4.1_006230m|PACid:17976345	cassava4.1_006230m	beta vacuolar processing enzyme	No
cassava4.1_006233m|PACid:17962974	cassava4.1_006233m	Signal recognition particle, SRP54 subunit protein	GP
cassava4.1_006236m|PACid:17971643	cassava4.1_006236m	glycosyl hydrolase 9B18	No
cassava4.1_006240m|PACid:17975080	cassava4.1_006240m	Alpha amylase family protein	No
cassava4.1_006241m|PACid:17970939	cassava4.1_006241m	6-phosphogluconate dehydrogenase family protein	GP
cassava4.1_006242m|PACid:17973475	cassava4.1_006242m	Eukaryotic aspartyl protease family protein	No
cassava4.1_006245m|PACid:17986520	cassava4.1_006245m		G
cassava4.1_006247m|PACid:17980439	cassava4.1_006247m	Protein phosphatase 2A regulatory B subunit family protein	No
cassava4.1_006252m|PACid:17964773	cassava4.1_006252m	aldehyde dehydrogenase 3H1	GP
cassava4.1_006253m|PACid:17987531	cassava4.1_006253m	Cofactor-independent phosphoglycerate mutase	No
cassava4.1_006257m|PACid:17981793	cassava4.1_006257m	plectin-related	G
cassava4.1_006266m|PACid:17973050	cassava4.1_006266m	Pentatricopeptide repeat (PPR) superfamily protein	No
cassava4.1_006269m|PACid:17972117	cassava4.1_006269m	Cyclophilin-like peptidyl-prolyl cis-trans isomerase family protein	G
cassava4.1_006274m|PACid:17982976	cassava4.1_006274m	P-loop containing nucleoside triphosphate hydrolases superfamily protein	GP
cassava4.1_006279m|PACid:17960290	cassava4.1_006279m	Pectin lyase-like superfamily protein	No
cassava4.1_006280m|PACid:17978626	cassava4.1_006280m	BR-signaling kinase 3	No
cassava4.1_006282m|PACid:17965486	cassava4.1_006282m	N-acetylglucosamine-1-phosphate uridylyltransferase 1	GP
cassava4.1_006283m|PACid:17992622	cassava4.1_006283m	hydroxyproline-rich glycoprotein family protein	G
cassava4.1_006286m|PACid:17989309	cassava4.1_006286m	arginosuccinate synthase family	GP
cassava4.1_006288m|PACid:17970632	cassava4.1_006288m	Protein kinase superfamily protein	G
cassava4.1_006289m|PACid:17990776	cassava4.1_006289m	Transducin family protein / WD-40 repeat family protein	GP
cassava4.1_006294m|PACid:17991932	cassava4.1_006294m	CCCH-type zinc fingerfamily protein with RNA-binding domain	No
cassava4.1_006297m|PACid:17966485	cassava4.1_006297m	catalase 2	P
cassava4.1_006300m|PACid:17967917	cassava4.1_006300m	diacylglycerol kinase 5	No
cassava4.1_006303m|PACid:17984205	cassava4.1_006303m	catalase 2	P
cassava4.1_006304m|PACid:17983882	cassava4.1_006304m	bZIP transcription factor family protein	No
cassava4.1_006307m|PACid:17990854	cassava4.1_006307m	thioredoxin family protein	GP
cassava4.1_006318m|PACid:17982188	cassava4.1_006318m	phenylalanyl-tRNA synthetase, putative / phenylalanine--tRNA ligase, putative	GP
cassava4.1_006326m|PACid:17993105	cassava4.1_006326m	polyamine oxidase 2	G
cassava4.1_006328m|PACid:17981033	cassava4.1_006328m	Pentatricopeptide repeat (PPR-like) superfamily protein	No
cassava4.1_006333m|PACid:17986662	cassava4.1_006333m	CBS domain-containing protein with a domain of unknown function (DUF21)	GP
cassava4.1_006334m|PACid:17974949	cassava4.1_006334m	Insulinase (Peptidase family M16) family protein	No
cassava4.1_006335m|PACid:17969936	cassava4.1_006335m	Regulator of chromosome condensation (RCC1) family protein	No
cassava4.1_006336m|PACid:17991287	cassava4.1_006336m	beta glucosidase 42	GP
cassava4.1_006339m|PACid:17983232	cassava4.1_006339m	Protein of unknown function (DUF399 and DUF3411)	No
cassava4.1_006344m|PACid:17977291	cassava4.1_006344m	Flavin-binding monooxygenase family protein	G
cassava4.1_006349m|PACid:17977632	cassava4.1_006349m	AAA-ATPase 1	GP

cassava4.1_006353m|PACid:17988533	cassava4.1_006353m	UDP-glucosyl transferase 85A2	GP
cassava4.1_006356m|PACid:17985270	cassava4.1_006356m	RNI-like superfamily protein	No
cassava4.1_006360m|PACid:17968445	cassava4.1_006360m	Glucose-1-phosphate adenylyltransferase family protein	GP
cassava4.1_006367m|PACid:17979811	cassava4.1_006367m	monodehydroascorbate reductase 6	GP
cassava4.1_006368m|PACid:17966393	cassava4.1_006368m	Cytochrome P450 superfamily protein	No
cassava4.1_006370m|PACid:17986482	cassava4.1_006370m	Tetratricopeptide repeat (TPR)-like superfamily protein	GP
cassava4.1_006372m|PACid:17990804	cassava4.1_006372m	sphingosine kinase 1	No
cassava4.1_006373m|PACid:17973369	cassava4.1_006373m	Regulator of chromosome condensation (RCC1) family protein	No
cassava4.1_006377m|PACid:17968081	cassava4.1_006377m	S-adenosyl-L-methionine-dependent methyltransferases superfamily protein	No
cassava4.1_006387m|PACid:17980378	cassava4.1_006387m	Protein kinase superfamily protein	No
cassava4.1_006388m|PACid:17986276	cassava4.1_006388m	transducin family protein / WD-40 repeat family protein	G
cassava4.1_006389m|PACid:17967386	cassava4.1_006389m	long chain base2	No
cassava4.1_006391m|PACid:17983415	cassava4.1_006391m	histidinol dehydrogenase	GP
cassava4.1_006393m|PACid:17977370	cassava4.1_006393m	ENHANCED DISEASE RESISTANCE 2	G
cassava4.1_006409m|PACid:17968834	cassava4.1_006409m	ATPase, V1 complex, subunit B protein	P
cassava4.1_006412m|PACid:17976971	cassava4.1_006412m	NHL domain-containing protein	No
cassava4.1_006413m|PACid:17967117	cassava4.1_006413m	malonyl-CoA decarboxylase family protein	G
cassava4.1_006415m|PACid:17970966	cassava4.1_006415m	Protein kinase protein with tetratricopeptide repeat domain	GP
cassava4.1_006416m|PACid:17970566	cassava4.1_006416m	O-Glycosyl hydrolases family 17 protein	No
cassava4.1_006424m|PACid:17990197	cassava4.1_006424m	ATPase, V1 complex, subunit B protein	No
cassava4.1_006432m|PACid:17961511	cassava4.1_006432m	Pyridoxal-dependent decarboxylase family protein	GP
cassava4.1_006435m|PACid:17989643	cassava4.1_006435m	6-phosphogluconate dehydrogenase family protein	No
cassava4.1_006438m|PACid:17982604	cassava4.1_006438m	adenylosuccinate synthase	GP
cassava4.1_006439m|PACid:17979461	cassava4.1_006439m	Serine carboxypeptidase S28 family protein	P
cassava4.1_006440m|PACid:17991967	cassava4.1_006440m	Protein kinase protein with tetratricopeptide repeat domain	GP
cassava4.1_006443m|PACid:17986675	cassava4.1_006443m	Major facilitator superfamily protein	P
cassava4.1_006444m|PACid:17977643	cassava4.1_006444m	Amino acid permease family protein	G
cassava4.1_006445m|PACid:17977001	cassava4.1_006445m	PAM domain (PCI/PINT associated module) protein	GP
cassava4.1_006446m|PACid:17989061	cassava4.1_006446m	MATE efflux family protein	G
cassava4.1_006449m|PACid:17972212	cassava4.1_006449m	cellulase 3	GP
cassava4.1_006455m|PACid:17981761	cassava4.1_006455m	CYTOCHROME P450 51G1	GP
cassava4.1_006456m|PACid:17964491	cassava4.1_006456m	ferrochelatase 1	No
cassava4.1_006459m|PACid:17982076	cassava4.1_006459m	SKP1/ASK-interacting protein 16	No
cassava4.1_006462m|PACid:17965776	cassava4.1_006462m	MATE efflux family protein	G
cassava4.1_006463m|PACid:17963239	cassava4.1_006463m	RNAhelicase-like 8	No
cassava4.1_006464m|PACid:17961866	cassava4.1_006464m	Major facilitator superfamily protein	G
cassava4.1_006466m|PACid:17967944	cassava4.1_006466m	Protein kinase superfamily protein	No
cassava4.1_006469m|PACid:17993436	cassava4.1_006469m	XB3 ortholog 5 in Arabidopsis thaliana	G
cassava4.1_006470m|PACid:17976068	cassava4.1_006470m	cytochrome P450, family 90, subfamily D, polypeptide 1	G

cassava4.1_006473m|PACid:17978544	cassava4.1_006473m	Protein kinase superfamily protein	No
cassava4.1_006476m|PACid:17963337	cassava4.1_006476m	51 kDa subunit of complex I	GP
cassava4.1_006479m|PACid:17994168	cassava4.1_006479m	protein phosphatase 5.2	GP
cassava4.1_006481m|PACid:17969409	cassava4.1_006481m	Thiamin diphosphate-binding fold (THDP-binding) superfamily protein	GP
cassava4.1_006482m|PACid:17966691	cassava4.1_006482m	FIZZY-related 2	G
cassava4.1_006483m|PACid:17965560	cassava4.1_006483m	Major facilitator superfamily protein	GP
cassava4.1_006484m|PACid:17986774	cassava4.1_006484m	Tetratricopeptide repeat (TPR)-like superfamily protein	No
cassava4.1_006490m|PACid:17965649	cassava4.1_006490m	pfkB-like carbohydrate kinase family protein	G
cassava4.1_006491m|PACid:17968405	cassava4.1_006491m	pre-mRNA processing ribonucleoprotein binding region-containing protein	GP
cassava4.1_006492m|PACid:17980020	cassava4.1_006492m	Rubisco methyltransferase family protein	No
cassava4.1_006495m|PACid:17983407	cassava4.1_006495m	Pyridine nucleotide-disulphide oxidoreductase family protein	P
cassava4.1_006496m|PACid:17977025	cassava4.1_006496m	Tyrosyl-tRNA synthetase, class Ib, bacterial/mitochondrial	GP
cassava4.1_006498m|PACid:17982456	cassava4.1_006498m	S-adenosyl-L-homocysteine hydrolase	GP
cassava4.1_006499m|PACid:17989151	cassava4.1_006499m	FIZZY-related 3	No
cassava4.1_006500m|PACid:17960962	cassava4.1_006500m	amino acid permease 6	No
cassava4.1_006503m|PACid:17987006	cassava4.1_006503m	FAD-dependent oxidoreductase family protein	G
cassava4.1_006506m|PACid:17976264	cassava4.1_006506m	26S proteasome, regulatory subunit Rpn7;Proteasome component (PCI) domain	GP
cassava4.1_006508m|PACid:17967572	cassava4.1_006508m	S-adenosyl-L-homocysteine hydrolase	GP
cassava4.1_006513m|PACid:17961027	cassava4.1_006513m	RNA-binding (RRM/RBD/RNP motifs) family protein	No
cassava4.1_006515m|PACid:17974698	cassava4.1_006515m	O-Glycosyl hydrolases family 17 protein	No
cassava4.1_006518m|PACid:17993318	cassava4.1_006518m	Metallopeptidase M24 family protein	No
cassava4.1_006519m|PACid:17980379	cassava4.1_006519m	Tetratricopeptide repeat (TPR)-like superfamily protein	G
cassava4.1_006522m|PACid:17966194	cassava4.1_006522m	aldehyde dehydrogenase 3F1	GP
cassava4.1_006523m|PACid:17983514	cassava4.1_006523m	zinc finger (ubiquitin-hydrolase) domain-containing protein	G
cassava4.1_006524m|PACid:17979626	cassava4.1_006524m	pleiotropic regulatory locus 1	GP
cassava4.1_006533m|PACid:17975925	cassava4.1_006533m	Zn-dependent exopeptidases superfamily protein	GP
cassava4.1_006534m|PACid:17984315	cassava4.1_006534m	alanine:glyoxylate aminotransferase 2	GP
cassava4.1_006535m|PACid:17991348	cassava4.1_006535m	amino acid permease 3	G
cassava4.1_006537m|PACid:17968540	cassava4.1_006537m	amino acid permease 6	No
cassava4.1_006543m|PACid:17972114	cassava4.1_006543m	sulfoquinovosyldiacylglycerol 1	GP
cassava4.1_006544m|PACid:17974999	cassava4.1_006544m	Transducin/WD40 repeat-like superfamily protein	GP
cassava4.1_006547m|PACid:17969898	cassava4.1_006547m	Pseudouridine synthase/archaeosine transglycosylase-like family protein	P
cassava4.1_006548m|PACid:17978954	cassava4.1_006548m	DA1	G
cassava4.1_006550m|PACid:17992599	cassava4.1_006550m	long-chain base1	GP
cassava4.1_006552m|PACid:17978222	cassava4.1_006552m	Protein kinase superfamily protein	No
cassava4.1_006555m|PACid:17961318	cassava4.1_006555m	RING/U-box superfamily protein	No
cassava4.1_006559m|PACid:17962273	cassava4.1_006559m	diacylglycerol kinase 3	GP
cassava4.1_006562m|PACid:17982296	cassava4.1_006562m	translocase inner membrane subunit 44-2	GP
cassava4.1_006563m|PACid:17993031	cassava4.1_006563m	Vacuolar sorting protein 9 (VPS9) domain	No

cassava4.1_006566m|PACid:17982631	cassava4.1_006566m	Pectin lyase-like superfamily protein	No
cassava4.1_006569m|PACid:17960331	cassava4.1_006569m	alpha/beta-Hydrolases superfamily protein	GP
cassava4.1_006570m|PACid:17985020	cassava4.1_006570m	Transmembrane amino acid transporter family protein	G
cassava4.1_006574m|PACid:17960638	cassava4.1_006574m	non-intrinsic ABC protein 6	G
cassava4.1_006578m|PACid:17988599	cassava4.1_006578m	Tetratricopeptide repeat (TPR)-like superfamily protein	G
cassava4.1_006580m|PACid:17987230	cassava4.1_006580m	NHL domain-containing protein	G
cassava4.1_006584m|PACid:17962513	cassava4.1_006584m	Protein of unknown function (DUF1682)	P
cassava4.1_006585m|PACid:17975976	cassava4.1_006585m	ubiquitin-specific protease 6	GP
cassava4.1_006586m|PACid:17981205	cassava4.1_006586m	selenium-binding protein 2	GP
cassava4.1_006591m|PACid:17978563	cassava4.1_006591m	Eukaryotic aspartyl protease family protein	GP
cassava4.1_006593m|PACid:17960378	cassava4.1_006593m	MATE efflux family protein	No
cassava4.1_006595m|PACid:17986292	cassava4.1_006595m	Major facilitator superfamily protein	G
cassava4.1_006600m|PACid:17978089	cassava4.1_006600m	Protein kinase superfamily protein	GP
cassava4.1_006601m|PACid:17989018	cassava4.1_006601m	aspartate aminotransferase	GP
cassava4.1_006605m|PACid:17968275	cassava4.1_006605m	phosphoglycerate kinase 1	GP
cassava4.1_006606m|PACid:17975833	cassava4.1_006606m	Protein kinase superfamily protein	No
cassava4.1_006608m|PACid:17961918	cassava4.1_006608m	Zinc finger C-x8-C-x5-C-x3-H type family protein	No
cassava4.1_006611m|PACid:17965289	cassava4.1_006611m	RING/FYVE/PHD-type zinc finger family protein	No
cassava4.1_006614m|PACid:17985236	cassava4.1_006614m	Transducin/WD40 repeat-like superfamily protein	No
cassava4.1_006616m|PACid:17963589	cassava4.1_006616m	cytochrome P450, family 716, subfamily A, polypeptide 1	G
cassava4.1_006617m|PACid:17963347	cassava4.1_006617m	alanine-2-oxoglutarate aminotransferase 2	G
cassava4.1_006621m|PACid:17969144	cassava4.1_006621m	zinc finger (C3HC4-type RING finger) family protein	G
cassava4.1_006625m|PACid:17989898	cassava4.1_006625m	phosphofructokinase 3	No
cassava4.1_006628m|PACid:17965368	cassava4.1_006628m	O-Glycosyl hydrolases family 17 protein	P
cassava4.1_006630m|PACid:17985742	cassava4.1_006630m	AAA-type ATPase family protein / ankyrin repeat family protein	G
cassava4.1_006633m|PACid:17980185	cassava4.1_006633m	Protein kinase superfamily protein	G
cassava4.1_006634m|PACid:17973371	cassava4.1_006634m	UDP-glucose 6-dehydrogenase family protein	GP
cassava4.1_006635m|PACid:17982799	cassava4.1_006635m	uridine kinase/uracil phosphoribosyltransferase 1	GP
cassava4.1_006643m|PACid:17961394	cassava4.1_006643m	ZIP metal ion transporter family	No
cassava4.1_006652m|PACid:17964617	cassava4.1_006652m		No
cassava4.1_006656m|PACid:17976617	cassava4.1_006656m	xylose isomerase family protein	GP
cassava4.1_006657m|PACid:17969185	cassava4.1_006657m	UDP-glucose 6-dehydrogenase family protein	No
cassava4.1_006659m|PACid:17985393	cassava4.1_006659m	Pectin lyase-like superfamily protein	No
cassava4.1_006660m|PACid:17980486	cassava4.1_006660m	serine carboxypeptidase-like 29	GP
cassava4.1_006663m|PACid:17983559	cassava4.1_006663m	UDP-glucose 6-dehydrogenase family protein	GP
cassava4.1_006665m|PACid:17961419	cassava4.1_006665m	1-aminocyclopropane-1-carboxylic acid (acc) synthase 6	G
cassava4.1_006667m|PACid:17970448	cassava4.1_006667m	Transducin/WD40 repeat-like superfamily protein	G
cassava4.1_006669m|PACid:17966920	cassava4.1_006669m	Transducin/WD40 repeat-like superfamily protein	No
cassava4.1_006671m|PACid:17969969	cassava4.1_006671m	vascular plant one zinc finger protein	G

cassava4.1_006672m|PACid:17986558	cassava4.1_006672m	purple acid phosphatase 26	GP
cassava4.1_006676m|PACid:17986568	cassava4.1_006676m	Transducin family protein / WD-40 repeat family protein	No
cassava4.1_006678m|PACid:17988760	cassava4.1_006678m	Regulator of chromosome condensation (RCC1) family protein	G
cassava4.1_006679m|PACid:17975672	cassava4.1_006679m	RNA-binding (RRM/RBD/RNP motifs) family protein	GP
cassava4.1_006682m|PACid:17972762	cassava4.1_006682m	O-Glycosyl hydrolases family 17 protein	No
cassava4.1_006686m|PACid:17970901	cassava4.1_006686m	UBX domain-containing protein	P
cassava4.1_006689m|PACid:17965544	cassava4.1_006689m	O-Glycosyl hydrolases family 17 protein	GP
cassava4.1_006696m|PACid:17969017	cassava4.1_006696m	2-oxoacid dehydrogenases acyltransferase family protein	GP
cassava4.1_006697m|PACid:17990421	cassava4.1_006697m	Eukaryotic aspartyl protease family protein	No
cassava4.1_006705m|PACid:17965610	cassava4.1_006705m	serine/threonine protein kinase 2	G
cassava4.1_006707m|PACid:17966570	cassava4.1_006707m	Protein of unknown function (DUF604)	No
cassava4.1_006708m|PACid:17974240	cassava4.1_006708m	Radical SAM superfamily protein	No
cassava4.1_006709m|PACid:17986024	cassava4.1_006709m	O-Glycosyl hydrolases family 17 protein	No
cassava4.1_006710m|PACid:17967239	cassava4.1_006710m	homolog of mamallian P58IPK	G
cassava4.1_006719m|PACid:17987128	cassava4.1_006719m	ARF-GAP domain 5	No
cassava4.1_006720m|PACid:17992270	cassava4.1_006720m	cyclin-dependent kinase E;1	G
cassava4.1_006725m|PACid:17993412	cassava4.1_006725m	ureidoglycolate amidohydrolase	GP
cassava4.1_006727m|PACid:17969674	cassava4.1_006727m	UDP-Glycosyltransferase superfamily protein	G
cassava4.1_006730m|PACid:17965000	cassava4.1_006730m	glutamate-1-semialdehyde 2,1-aminomutase 2	GP
cassava4.1_006731m|PACid:17986995	cassava4.1_006731m	GTP cyclohydrolase I	G
cassava4.1_006734m|PACid:17968639	cassava4.1_006734m	Polynucleotidyl transferase, ribonuclease H-like superfamily protein	G
cassava4.1_006735m|PACid:17970653	cassava4.1_006735m	Ankyrin repeat family protein / BTB/POZ domain-containing protein	G
cassava4.1_006743m|PACid:17966676	cassava4.1_006743m	methionine aminopeptidase 2B	No
cassava4.1_006749m|PACid:17971455	cassava4.1_006749m	tonneau 2 (TON2)	No
cassava4.1_006760m|PACid:17965930	cassava4.1_006760m	homolog of yeast sucrose nonfermenting 4	GP
cassava4.1_006764m|PACid:17984247	cassava4.1_006764m	alpha/beta-Hydrolases superfamily protein	G
cassava4.1_006765m|PACid:17982217	cassava4.1_006765m	ferredoxin hydrogenases	No
cassava4.1_006768m|PACid:17964315	cassava4.1_006768m	SOS3-interacting protein 1	No
cassava4.1_006769m|PACid:17970909	cassava4.1_006769m	Cam interacting protein 111	No
cassava4.1_006770m|PACid:17990843	cassava4.1_006770m	serine carboxypeptidase-like 31	G
cassava4.1_006774m|PACid:17993051	cassava4.1_006774m	Galactose oxidase/kelch repeat superfamily protein	G
cassava4.1_006779m|PACid:17963373	cassava4.1_006779m	Protein of Unknown Function (DUF239)	G
cassava4.1_006782m|PACid:17979255	cassava4.1_006782m	Tetratricopeptide repeat (TPR)-like superfamily protein	No
cassava4.1_006786m|PACid:17985123	cassava4.1_006786m	alpha/beta-Hydrolases superfamily protein	No
cassava4.1_006793m|PACid:17987166	cassava4.1_006793m	SecY protein transport family protein	GP
cassava4.1_006794m|PACid:17990664	cassava4.1_006794m	casein kinase 1	No
cassava4.1_006803m|PACid:17960670	cassava4.1_006803m	rubisco activase	P
cassava4.1_006804m|PACid:17978359	cassava4.1_006804m	biotin F	G
cassava4.1_006812m|PACid:17975493	cassava4.1_006812m	monogalactosyldiacylglycerol synthase 2	G

cassava4.1_006818m|PACid:17991960	cassava4.1_006818m	cytosolic enolase	GP
cassava4.1_006819m|PACid:17980519	cassava4.1_006819m	tryptophan synthase beta-subunit 2	GP
cassava4.1_006821m|PACid:17973606	cassava4.1_006821m	Thiamin diphosphate-binding fold (THDP-binding) superfamily protein	GP
cassava4.1_006826m|PACid:17960214	cassava4.1_006826m	Transcription factor TFIIE, alpha subunit	G
cassava4.1_006827m|PACid:17992178	cassava4.1_006827m	Protein phosphatase 2C family protein	No
cassava4.1_006829m|PACid:17963274	cassava4.1_006829m	shaggy-like protein kinase 32	G
cassava4.1_006834m|PACid:17988986	cassava4.1_006834m	Zinc finger C-x8-C-x5-C-x3-H type family protein	No
cassava4.1_006836m|PACid:17990810	cassava4.1_006836m	Tetratricopeptide repeat (TPR)-like superfamily protein	G
cassava4.1_006837m|PACid:17968714	cassava4.1_006837m	patatin-like protein 6	G
cassava4.1_006839m|PACid:17987050	cassava4.1_006839m	Integrin-linked protein kinase family	G
cassava4.1_006841m|PACid:17970948	cassava4.1_006841m	BTB/POZ domain with WD40/YVTN repeat-like protein	GP
cassava4.1_006842m|PACid:17988674	cassava4.1_006842m	L-Aspartase-like family protein	No
cassava4.1_006843m|PACid:17984382	cassava4.1_006843m	uridine 5\'-monophosphate synthase / UMP synthase (PYRE-F) (UMPS)	GP
cassava4.1_006845m|PACid:17980632	cassava4.1_006845m	Granulin repeat cysteine protease family protein	P
cassava4.1_006849m|PACid:17981981	cassava4.1_006849m	phosphoenolpyruvate carboxylase-related kinase 2	No
cassava4.1_006853m|PACid:17969747	cassava4.1_006853m	Citrate synthase family protein	GP
cassava4.1_006854m|PACid:17988113	cassava4.1_006854m	Nucleic acid-binding proteins superfamily	GP
cassava4.1_006859m|PACid:17970404	cassava4.1_006859m	ornithine-delta-aminotransferase	GP
cassava4.1_006860m|PACid:17981970	cassava4.1_006860m	Tryptophan/tyrosine permease	GP
cassava4.1_006863m|PACid:17967645	cassava4.1_006863m	O-Glycosyl hydrolases family 17 protein	GP
cassava4.1_006864m|PACid:17988647	cassava4.1_006864m	Cytochrome P450 superfamily protein	No
cassava4.1_006869m|PACid:17961484	cassava4.1_006869m	Rubisco methyltransferase family protein	GP
cassava4.1_006870m|PACid:17978497	cassava4.1_006870m	gamma-tubulin	No
cassava4.1_006872m|PACid:17973236	cassava4.1_006872m	casein kinase 1-like protein 2	G
cassava4.1_006876m|PACid:17971847	cassava4.1_006876m	Vacuolar sorting protein 9 (VPS9) domain	No
cassava4.1_006879m|PACid:17961413	cassava4.1_006879m	plant UBX domain-containing protein 2	G
cassava4.1_006881m|PACid:17978124	cassava4.1_006881m	RING/U-box superfamily protein	G
cassava4.1_006896m|PACid:17977955	cassava4.1_006896m	Eukaryotic aspartyl protease family protein	No
cassava4.1_006898m|PACid:17981281	cassava4.1_006898m	protein serine/threonine kinases;ATP binding;catalytics	G
cassava4.1_006899m|PACid:17974044	cassava4.1_006899m	1-deoxy-D-xylulose 5-phosphate reductoisomerase	GP
cassava4.1_006905m|PACid:17976667	cassava4.1_006905m	HD domain-containing metal-dependent phosphohydrolase family protein	No
cassava4.1_006906m|PACid:17988179	cassava4.1_006906m	2-oxoacid dehydrogenases acyltransferase family protein	GP
cassava4.1_006916m|PACid:17985504	cassava4.1_006916m	ARF-GAP domain 6	GP
cassava4.1_006917m|PACid:17961244	cassava4.1_006917m	RNA-binding (RRM/RBD/RNP motifs) family protein	GP
cassava4.1_006919m|PACid:17987758	cassava4.1_006919m	Dihydrolipoamide succinyltransferase	No
cassava4.1_006923m|PACid:17993925	cassava4.1_006923m	WD-40 repeat family protein / notchless protein, putative	G
cassava4.1_006924m|PACid:17967525	cassava4.1_006924m	serine hydroxymethyltransferase 4	GP
cassava4.1_006931m|PACid:17969136	cassava4.1_006931m	Pyridine nucleotide-disulphide oxidoreductase family protein	P
cassava4.1_006934m|PACid:17964718	cassava4.1_006934m	apyrase 2	G

cassava4.1_006939m|PACid:17981772	cassava4.1_006939m	AFG1-like ATPase family protein	No
cassava4.1_006960m|PACid:17982060	cassava4.1_006960m	mitochondrial 28S ribosomal protein S29-related	G
cassava4.1_006961m|PACid:17989826	cassava4.1_006961m	G-protein coupled receptors;GTPase activators	G
cassava4.1_006965m|PACid:17961642	cassava4.1_006965m	UDP-glucose pyrophosphorylase 2	GP
cassava4.1_006966m|PACid:17982185	cassava4.1_006966m	cyclase associated protein 1	P
cassava4.1_006968m|PACid:17981903	cassava4.1_006968m	scarecrow-like 3	No
cassava4.1_006970m|PACid:17976618	cassava4.1_006970m	CBL-interacting protein kinase 21	No
cassava4.1_006971m|PACid:17959725	cassava4.1_006971m	laccase 7	No
cassava4.1_006974m|PACid:17980897	cassava4.1_006974m	Transducin/WD40 repeat-like superfamily protein	G
cassava4.1_006976m|PACid:17969606	cassava4.1_006976m	histone deacetylase 6	G
cassava4.1_006981m|PACid:17964949	cassava4.1_006981m	O-Glycosyl hydrolases family 17 protein	G
cassava4.1_006985m|PACid:17991368	cassava4.1_006985m	IAA-leucine resistant (ILR)-like gene 6	No
cassava4.1_006990m|PACid:17960217	cassava4.1_006990m	Transmembrane Fragile-X-F-associated protein	No
cassava4.1_006991m|PACid:17971989	cassava4.1_006991m	3-ketoacyl-acyl carrier protein synthase I	GP
cassava4.1_006993m|PACid:17979349	cassava4.1_006993m	Tetratricopeptide repeat (TPR)-like superfamily protein	G
cassava4.1_007000m|PACid:17967817	cassava4.1_007000m	cytochrome P450, family 707, subfamily A, polypeptide 2	G
cassava4.1_007002m|PACid:17976397	cassava4.1_007002m	polypyrimidine tract-binding protein 1	G
cassava4.1_007003m|PACid:17961411	cassava4.1_007003m	Pectin lyase-like superfamily protein	GP
cassava4.1_007006m|PACid:17967988	cassava4.1_007006m	cytochrome P450, family 707, subfamily A, polypeptide 1	G
cassava4.1_007007m|PACid:17961583	cassava4.1_007007m	SCP1-like small phosphatase 5	G
cassava4.1_007008m|PACid:17971056	cassava4.1_007008m	Pectin lyase-like superfamily protein	GP
cassava4.1_007009m|PACid:17962288	cassava4.1_007009m	Regulator of chromosome condensation (RCC1) family protein	No
cassava4.1_007010m|PACid:17967843	cassava4.1_007010m	6-phosphogluconate dehydrogenase family protein	GP
cassava4.1_007012m|PACid:17964097	cassava4.1_007012m	Peptidase S41 family protein	No
cassava4.1_007017m|PACid:17961259	cassava4.1_007017m	Eukaryotic aspartyl protease family protein	G
cassava4.1_007019m|PACid:17979137	cassava4.1_007019m	cystathionine beta-lyase	P
cassava4.1_007021m|PACid:17977090	cassava4.1_007021m	P-loop containing nucleoside triphosphate hydrolases superfamily protein	No
cassava4.1_007022m|PACid:17971664	cassava4.1_007022m	peroxisomal 3-ketoacyl-CoA thiolase 3	GP
cassava4.1_007023m|PACid:17981135	cassava4.1_007023m	Ribophorin I	GP
cassava4.1_007024m|PACid:17986229	cassava4.1_007024m	Rho GTPase activating protein with PAK-box/P21-Rho-binding domain	No
cassava4.1_007025m|PACid:17987281	cassava4.1_007025m	RNI-like superfamily protein	No
cassava4.1_007026m|PACid:17975085	cassava4.1_007026m	co-factor for nitrate, reductase and xanthine dehydrogenase 5	GP
cassava4.1_007032m|PACid:17981818	cassava4.1_007032m	Protein kinase superfamily protein	G
cassava4.1_007033m|PACid:17964698	cassava4.1_007033m	Calcium-binding endonuclease/exonuclease/phosphatase family	G
cassava4.1_007039m|PACid:17960325	cassava4.1_007039m	RNA polymerase I-associated factor PAF67	GP
cassava4.1_007049m|PACid:17966627	cassava4.1_007049m	Phospholipid/glycerol acyltransferase family protein	No
cassava4.1_007050m|PACid:17986459	cassava4.1_007050m	alpha/beta-Hydrolases superfamily protein	No
cassava4.1_007055m|PACid:17989723	cassava4.1_007055m	Major facilitator superfamily protein	G
cassava4.1_007056m|PACid:17969305	cassava4.1_007056m	Transducin/WD40 repeat-like superfamily protein	No

cassava4.1_007062m|PACid:17994048	cassava4.1_007062m	Peptidase M28 family protein	No
cassava4.1_007063m|PACid:17961469	cassava4.1_007063m	PIF / Ping-Pong family of plant transposases	No
cassava4.1_007066m|PACid:17986055	cassava4.1_007066m	Arabinanase/levansucrase/invertase	G
cassava4.1_007069m|PACid:17963978	cassava4.1_007069m	alpha/beta-Hydrolases superfamily protein	No
cassava4.1_007070m|PACid:17971572	cassava4.1_007070m	O-Glycosyl hydrolases family 17 protein	P
cassava4.1_007074m|PACid:17973059	cassava4.1_007074m	TGACG motif-binding factor 6	G
cassava4.1_007075m|PACid:17968223	cassava4.1_007075m	MBOAT (membrane bound O-acyl transferase) family protein	No
cassava4.1_007076m|PACid:17993442	cassava4.1_007076m	Plant protein of unknown function (DUF828) with plant pleckstrin homology-like region	No
cassava4.1_007081m|PACid:17966714	cassava4.1_007081m	hydroxymethylglutaryl-CoA synthase / HMG-CoA synthase	GP
cassava4.1_007082m|PACid:17980178	cassava4.1_007082m	permease, cytosine/purines, uracil, thiamine, allantoin family protein	No
cassava4.1_007090m|PACid:17982285	cassava4.1_007090m	UB-like protease 1D	No
cassava4.1_007094m|PACid:17985984	cassava4.1_007094m	aspartate aminotransferase 5	GP
cassava4.1_007101m|PACid:17991632	cassava4.1_007101m	HhH-GPD base excision DNA repair family protein	G
cassava4.1_007114m|PACid:17993615	cassava4.1_007114m	Transmembrane amino acid transporter family protein	G
cassava4.1_007118m|PACid:17976256	cassava4.1_007118m	digalactosyl diacylglycerol deficient 2	G
cassava4.1_007125m|PACid:17980140	cassava4.1_007125m	brassinosteroid-6-oxidase 2	No
cassava4.1_007128m|PACid:17982455	cassava4.1_007128m	sirtuin 1	G
cassava4.1_007130m|PACid:17979530	cassava4.1_007130m	eukaryotic translation initiation factor 2 gamma subunit	GP
cassava4.1_007131m|PACid:17966453	cassava4.1_007131m	Leucine-rich repeat (LRR) family protein	GP
cassava4.1_007132m|PACid:17991498	cassava4.1_007132m	calcium-dependent protein kinase 21	GP
cassava4.1_007135m|PACid:17982406	cassava4.1_007135m	Ypt/Rab-GAP domain of gyp1p superfamily protein	G
cassava4.1_007136m|PACid:17972643	cassava4.1_007136m	CBL-interacting protein kinase 23	G
cassava4.1_007144m|PACid:17962108	cassava4.1_007144m	casein kinase I	G
cassava4.1_007150m|PACid:17986056	cassava4.1_007150m	Arabinanase/levansucrase/invertase	No
cassava4.1_007155m|PACid:17963439	cassava4.1_007155m	RING domain ligase2	No
cassava4.1_007160m|PACid:17962212	cassava4.1_007160m	ATP sulfurylase 1	GP
cassava4.1_007163m|PACid:17984600	cassava4.1_007163m	Peptidase M20/M25/M40 family protein	GP
cassava4.1_007166m|PACid:17978629	cassava4.1_007166m	putative mitochondrial RNA helicase 2	GP
cassava4.1_007169m|PACid:17961546	cassava4.1_007169m	Protein kinase superfamily protein	GP
cassava4.1_007173m|PACid:17978740	cassava4.1_007173m	maternal effect embryo arrest 18	No
cassava4.1_007177m|PACid:17977906	cassava4.1_007177m	Protein kinase superfamily protein	G
cassava4.1_007181m|PACid:17982503	cassava4.1_007181m	peroxisomal 3-ketoacyl-CoA thiolase 3	GP
cassava4.1_007182m|PACid:17963273	cassava4.1_007182m	glycoprotease 1	No
cassava4.1_007186m|PACid:17990544	cassava4.1_007186m	6-phosphogluconate dehydrogenase family protein	No
cassava4.1_007189m|PACid:17973709	cassava4.1_007189m	ankyrin repeat family protein	G
cassava4.1_007190m|PACid:17983583	cassava4.1_007190m	serine carboxypeptidase-like 25	No
cassava4.1_007191m|PACid:17977194	cassava4.1_007191m	Sec1/munc18-like (SM) proteins superfamily	No
cassava4.1_007193m|PACid:17966418	cassava4.1_007193m	purple acid phosphatase 25	No
cassava4.1_007195m|PACid:17992355	cassava4.1_007195m	FASCICLIN-like arabinogalactan protein 17 precursor	GP

cassava4.1_007196m|PACid:17990288	cassava4.1_007196m		GP
cassava4.1_007197m|PACid:17977777	cassava4.1_007197m	4-hydroxy-3-methylbut-2-enyl diphosphate reductase	No
cassava4.1_007211m|PACid:17961543	cassava4.1_007211m	high chlorophyll fluorescent 109	No
cassava4.1_007212m|PACid:17967242	cassava4.1_007212m	transferases;nucleotidyltransferases	GP
cassava4.1_007220m|PACid:17987581	cassava4.1_007220m	Transmembrane amino acid transporter family protein	No
cassava4.1_007221m|PACid:17990129	cassava4.1_007221m	hexokinase 3	GP
cassava4.1_007222m|PACid:17981484	cassava4.1_007222m	photolyase/blue-light receptor 2	No
cassava4.1_007226m|PACid:17969797	cassava4.1_007226m	LJRHL1-like 1	G
cassava4.1_007227m|PACid:17989576	cassava4.1_007227m	Transmembrane amino acid transporter family protein	No
cassava4.1_007228m|PACid:17991518	cassava4.1_007228m	APS reductase 3	GP
cassava4.1_007229m|PACid:17982684	cassava4.1_007229m	3\'-5\'-exoribonuclease family protein	No
cassava4.1_007234m|PACid:17989823	cassava4.1_007234m	Pyridoxal phosphate (PLP)-dependent transferases superfamily protein	GP
cassava4.1_007237m|PACid:17990017	cassava4.1_007237m	Major facilitator superfamily protein	G
cassava4.1_007238m|PACid:17992378	cassava4.1_007238m	methionine gamma-lyase	GP
cassava4.1_007243m|PACid:17986443	cassava4.1_007243m	Protein of Unknown Function (DUF239)	No
cassava4.1_007244m|PACid:17990948	cassava4.1_007244m	Transducin/WD40 repeat-like superfamily protein	No
cassava4.1_007245m|PACid:17985553	cassava4.1_007245m	Pectinacetylesterase family protein	No
cassava4.1_007248m|PACid:17970397	cassava4.1_007248m	endoplasmic reticulum oxidoreductins 1	No
cassava4.1_007254m|PACid:17961981	cassava4.1_007254m	UDP-xylosyltransferase 2	G
cassava4.1_007255m|PACid:17961575	cassava4.1_007255m	Galactose oxidase/kelch repeat superfamily protein	No
cassava4.1_007258m|PACid:17980911	cassava4.1_007258m	dsRNA-binding protein 2	No
cassava4.1_007262m|PACid:17993103	cassava4.1_007262m	Regulator of chromosome condensation (RCC1) family protein	No
cassava4.1_007264m|PACid:17971131	cassava4.1_007264m	RING domain ligase2	No
cassava4.1_007265m|PACid:17977158	cassava4.1_007265m	Pentatricopeptide repeat (PPR) superfamily protein	No
cassava4.1_007266m|PACid:17986911	cassava4.1_007266m	SOS3-interacting protein 1	G
cassava4.1_007284m|PACid:17972890	cassava4.1_007284m	galacturonosyltransferase 15	G
cassava4.1_007298m|PACid:17963080	cassava4.1_007298m	RING domain ligase2	No
cassava4.1_007301m|PACid:17961548	cassava4.1_007301m	DNA primase, large subunit family	GP
cassava4.1_007302m|PACid:17982190	cassava4.1_007302m	O-Glycosyl hydrolases family 17 protein	GP
cassava4.1_007312m|PACid:17984791	cassava4.1_007312m	Transmembrane amino acid transporter family protein	G
cassava4.1_007313m|PACid:17988302	cassava4.1_007313m	nitrogen fixation S (NIFS)-like 1	GP
cassava4.1_007314m|PACid:17973867	cassava4.1_007314m	Pyridoxal phosphate (PLP)-dependent transferases superfamily protein	G
cassava4.1_007317m|PACid:17994043	cassava4.1_007317m	FASCICLIN-like arabinogalactan protein 17 precursor	GP
cassava4.1_007320m|PACid:17982555	cassava4.1_007320m	plant U-box 9	G
cassava4.1_007322m|PACid:17974202	cassava4.1_007322m	P-loop containing nucleoside triphosphate hydrolases superfamily protein	No
cassava4.1_007337m|PACid:17967255	cassava4.1_007337m	uridine kinase-like 3	GP
cassava4.1_007351m|PACid:17969216	cassava4.1_007351m	bZIP transcription factor family protein	No
cassava4.1_007352m|PACid:17975622	cassava4.1_007352m	UDP-Glycosyltransferase superfamily protein	No
cassava4.1_007354m|PACid:17963106	cassava4.1_007354m	high affinity nitrate transporter 2.7	G

cassava4.1_007359m|PACid:17967756	cassava4.1_007359m	OSBP(oxysterol binding protein)-related protein 3C	P
cassava4.1_007366m|PACid:17975271	cassava4.1_007366m	plastid transcriptionally active 17	GP
cassava4.1_007367m|PACid:17992440	cassava4.1_007367m	3-ketoacyl-CoA synthase 7	No
cassava4.1_007369m|PACid:17983378	cassava4.1_007369m	serine carboxypeptidase-like 45	No
cassava4.1_007370m|PACid:17980331	cassava4.1_007370m	cation exchanger 3	No
cassava4.1_007371m|PACid:17971919	cassava4.1_007371m	pyrimidine d	GP
cassava4.1_007372m|PACid:17963578	cassava4.1_007372m	Protein kinase superfamily protein	G
cassava4.1_007379m|PACid:17983541	cassava4.1_007379m	Saccharopine dehydrogenase	GP
cassava4.1_007380m|PACid:17965562	cassava4.1_007380m	Transducin family protein / WD-40 repeat family protein	G
cassava4.1_007387m|PACid:17982397	cassava4.1_007387m	UDP-D-glucuronate 4-epimerase 6	G
cassava4.1_007390m|PACid:17966125	cassava4.1_007390m	Presenilin-1	No
cassava4.1_007392m|PACid:17969309	cassava4.1_007392m	Transducin family protein / WD-40 repeat family protein	G
cassava4.1_007394m|PACid:17964072	cassava4.1_007394m	Integrin-linked protein kinase family	GP
cassava4.1_007396m|PACid:17973775	cassava4.1_007396m	Chalcone-flavanone isomerase family protein	GP
cassava4.1_007405m|PACid:17963068	cassava4.1_007405m	Isocitrate/isopropylmalate dehydrogenase family protein	GP
cassava4.1_007409m|PACid:17984673	cassava4.1_007409m	UBX domain-containing protein	G
cassava4.1_007414m|PACid:17972038	cassava4.1_007414m	Enhancer of polycomb-like transcription factor protein	G
cassava4.1_007421m|PACid:17979616	cassava4.1_007421m	Pectin lyase-like superfamily protein	No
cassava4.1_007427m|PACid:17962728	cassava4.1_007427m	COBRA-like extracellular glycosyl-phosphatidyl inositol-anchored protein family	G
cassava4.1_007429m|PACid:17993709	cassava4.1_007429m	DNA polymerase delta small subunit	No
cassava4.1_007435m|PACid:17976102	cassava4.1_007435m	63 kDa inner membrane family protein	No
cassava4.1_007436m|PACid:17961915	cassava4.1_007436m	TRICHOME BIREFRINGENCE-LIKE 11	No
cassava4.1_007438m|PACid:17988847	cassava4.1_007438m	homolog of asparagine-linked glycosylation 12	No
cassava4.1_007440m|PACid:17976663	cassava4.1_007440m	cyclophilin 38	GP
cassava4.1_007450m|PACid:17975059	cassava4.1_007450m	mannose-1-phosphate guanylyltransferase (GDP)	No
cassava4.1_007451m|PACid:17972961	cassava4.1_007451m	fatty acid desaturase 8	G
cassava4.1_007452m|PACid:17991636	cassava4.1_007452m	Regulator of chromosome condensation (RCC1) family protein	GP
cassava4.1_007455m|PACid:17965067	cassava4.1_007455m	DHHC-type zinc finger family protein	G
cassava4.1_007458m|PACid:17993908	cassava4.1_007458m	Protein kinase superfamily protein	GP
cassava4.1_007460m|PACid:17988191	cassava4.1_007460m	sucrose-proton symporter 2	G
cassava4.1_007465m|PACid:17970521	cassava4.1_007465m	Protein kinase superfamily protein	G
cassava4.1_007468m|PACid:17967140	cassava4.1_007468m	glyceraldehyde-3-phosphate dehydrogenase B subunit	GP
cassava4.1_007469m|PACid:17965189	cassava4.1_007469m	Mitochondrial transcription termination factor family protein	GP
cassava4.1_007474m|PACid:17990292	cassava4.1_007474m	amino acid permease 7	G
cassava4.1_007482m|PACid:17981916	cassava4.1_007482m	Clathrin adaptor complexes medium subunit family protein	GP
cassava4.1_007486m|PACid:17987211	cassava4.1_007486m	isopentenyltransferase 9	GP
cassava4.1_007489m|PACid:17980332	cassava4.1_007489m	cation exchanger 3	No
cassava4.1_007492m|PACid:17984982	cassava4.1_007492m	transcription factor-related	GP
cassava4.1_007494m|PACid:17978073	cassava4.1_007494m	DegP protease 1	No

cassava4.1_007497m|PACid:17979160	cassava4.1_007497m	Homeodomain-like superfamily protein	No
cassava4.1_007499m|PACid:17981543	cassava4.1_007499m	GTP binding Elongation factor Tu family protein	No
cassava4.1_007500m|PACid:17967640	cassava4.1_007500m	mannose-1-phosphate guanylyltransferase (GDP)	No
cassava4.1_007508m|PACid:17984480	cassava4.1_007508m	Transducin/WD40 repeat-like superfamily protein	No
cassava4.1_007511m|PACid:17988560	cassava4.1_007511m	Transducin/WD40 repeat-like superfamily protein	No
cassava4.1_007514m|PACid:17968664	cassava4.1_007514m	ACT domain repeat 3	G
cassava4.1_007516m|PACid:17961978	cassava4.1_007516m	Pectin lyase-like superfamily protein	GP
cassava4.1_007521m|PACid:17978256	cassava4.1_007521m	Protein of unknown function (DUF3537)	No
cassava4.1_007522m|PACid:17991620	cassava4.1_007522m	tubulin alpha-2 chain	G
cassava4.1_007524m|PACid:17987324	cassava4.1_007524m	HOPW1-1-interacting 1	GP
cassava4.1_007540m|PACid:17993625	cassava4.1_007540m	pyruvate dehydrogenase complex E1 alpha subunit	P
cassava4.1_007543m|PACid:17961071	cassava4.1_007543m	purple acid phosphatase 22	No
cassava4.1_007544m|PACid:17970785	cassava4.1_007544m	Pectin lyase-like superfamily protein	No
cassava4.1_007549m|PACid:17960392	cassava4.1_007549m	tubulin beta-1 chain	GP
cassava4.1_007552m|PACid:17959886	cassava4.1_007552m	RNA-binding KH domain-containing protein	GP
cassava4.1_007553m|PACid:17987797	cassava4.1_007553m	Ypt/Rab-GAP domain of gyp1p superfamily protein	No
cassava4.1_007558m|PACid:17972378	cassava4.1_007558m	E1 C-terminal related 1	G
cassava4.1_007560m|PACid:17972004	cassava4.1_007560m	tubulin alpha-5	GP
cassava4.1_007564m|PACid:17981807	cassava4.1_007564m	myosin heavy chain-related	No
cassava4.1_007565m|PACid:17982418	cassava4.1_007565m	GTP binding Elongation factor Tu family protein	GP
cassava4.1_007566m|PACid:17992739	cassava4.1_007566m	interferon-related developmental regulator family protein / IFRD protein family	G
cassava4.1_007568m|PACid:17982405	cassava4.1_007568m	Pectin lyase-like superfamily protein	G
cassava4.1_007571m|PACid:17985143	cassava4.1_007571m	lysine histidine transporter 1	G
cassava4.1_007577m|PACid:17969568	cassava4.1_007577m	Hydrolase-like protein family	G
cassava4.1_007581m|PACid:17977727	cassava4.1_007581m	Transducin/WD40 repeat-like superfamily protein	No
cassava4.1_007583m|PACid:17984880	cassava4.1_007583m	Protein kinase superfamily protein	No
cassava4.1_007590m|PACid:17969207	cassava4.1_007590m	Transducin/WD40 repeat-like superfamily protein	No
cassava4.1_007596m|PACid:17971831	cassava4.1_007596m	Translation initiation factor IF2/IF5	GP
cassava4.1_007600m|PACid:17979754	cassava4.1_007600m	UDP-glucuronic acid decarboxylase 1	GP
cassava4.1_007612m|PACid:17993101	cassava4.1_007612m	phytoene desaturation 1	GP
cassava4.1_007615m|PACid:17972899	cassava4.1_007615m	Protein kinase superfamily protein	No
cassava4.1_007618m|PACid:17986063	cassava4.1_007618m		No
cassava4.1_007620m|PACid:17991131	cassava4.1_007620m	Auxin efflux carrier family protein	G
cassava4.1_007622m|PACid:17972831	cassava4.1_007622m	vacuolar ATP synthase subunit H family protein	GP
cassava4.1_007625m|PACid:17959908	cassava4.1_007625m	Protein kinase superfamily protein	No
cassava4.1_007629m|PACid:17983984	cassava4.1_007629m	ACT domain repeat 4	No
cassava4.1_007632m|PACid:17985561	cassava4.1_007632m	tubulin beta 8	P
cassava4.1_007635m|PACid:17972327	cassava4.1_007635m	Protein kinase superfamily protein	G
cassava4.1_007637m|PACid:17976173	cassava4.1_007637m	BTB/POZ domain with WD40/YVTN repeat-like protein	G

cassava4.1_007638m|PACid:17986820	cassava4.1_007638m	gametophytic factor 2	G
cassava4.1_007641m|PACid:17978675	cassava4.1_007641m	Transducin/WD40 repeat-like superfamily protein	G
cassava4.1_007643m|PACid:17960380	cassava4.1_007643m	3-ketoacyl-CoA synthase 4	G
cassava4.1_007646m|PACid:17986798	cassava4.1_007646m	SWAP (Suppressor-of-White-APricot)/surp domain-containing protein / D111/G-patch domain-containing protein	G
cassava4.1_007648m|PACid:17964537	cassava4.1_007648m	Peptidase family M48 family protein	GP
cassava4.1_007649m|PACid:17983053	cassava4.1_007649m	Protein kinase superfamily protein	No
cassava4.1_007650m|PACid:17967223	cassava4.1_007650m	beta-6 tubulin	No
cassava4.1_007655m|PACid:17980288	cassava4.1_007655m	XB3 ortholog 1 in Arabidopsis thaliana	G
cassava4.1_007659m|PACid:17966460	cassava4.1_007659m	serine/threonine phosphatase 7	G
cassava4.1_007660m|PACid:17968519	cassava4.1_007660m	CBL-interacting protein kinase 8	G
cassava4.1_007661m|PACid:17960947	cassava4.1_007661m	Pyridoxal phosphate (PLP)-dependent transferases superfamily protein	GP
cassava4.1_007663m|PACid:17983322	cassava4.1_007663m	Leucine-rich repeat (LRR) family protein	No
cassava4.1_007666m|PACid:17986500	cassava4.1_007666m	D6 protein kinase like 2	G
cassava4.1_007669m|PACid:17963122	cassava4.1_007669m	Pentatricopeptide repeat (PPR) superfamily protein	No
cassava4.1_007673m|PACid:17979089	cassava4.1_007673m	Enolase	GP
cassava4.1_007675m|PACid:17977069	cassava4.1_007675m	ARM repeat superfamily protein	No
cassava4.1_007681m|PACid:17960536	cassava4.1_007681m	xylem bark cysteine peptidase 3	GP
cassava4.1_007682m|PACid:17966439	cassava4.1_007682m	3-dehydroquinate synthase, putative	GP
cassava4.1_007683m|PACid:17970819	cassava4.1_007683m	guanosine nucleotide diphosphate dissociation inhibitor 1	GP
cassava4.1_007684m|PACid:17982517	cassava4.1_007684m	Ypt/Rab-GAP domain of gyp1p superfamily protein	G
cassava4.1_007687m|PACid:17971708	cassava4.1_007687m	regulatory particle AAA-ATPase 2A	GP
cassava4.1_007695m|PACid:17991352	cassava4.1_007695m	Histone H3 K4-specific methyltransferase SET7/9 family protein	G
cassava4.1_007696m|PACid:17969933	cassava4.1_007696m	root FNR 1	GP
cassava4.1_007698m|PACid:17991759	cassava4.1_007698m	Protein kinase superfamily protein	G
cassava4.1_007703m|PACid:17973484	cassava4.1_007703m	magnesium (Mg) transporter 10	No
cassava4.1_007705m|PACid:17971800	cassava4.1_007705m	Mitochondrial substrate carrier family protein	G
cassava4.1_007708m|PACid:17984867	cassava4.1_007708m	nudix hydrolase homolog 19	GP
cassava4.1_007709m|PACid:17982300	cassava4.1_007709m	aspartate aminotransferase 3	GP
cassava4.1_007710m|PACid:17989047	cassava4.1_007710m	NAD(P)-binding Rossmann-fold superfamily protein	GP
cassava4.1_007713m|PACid:17984884	cassava4.1_007713m	tubulin beta 8	P
cassava4.1_007714m|PACid:17993014	cassava4.1_007714m	Translation initiation factor IF2/IF5	No
cassava4.1_007717m|PACid:17966719	cassava4.1_007717m	ACT domain repeat 8	G
cassava4.1_007720m|PACid:17964645	cassava4.1_007720m	methyltransferases	G
cassava4.1_007722m|PACid:17990408	cassava4.1_007722m	magnesium transporter 2	No
cassava4.1_007723m|PACid:17966405	cassava4.1_007723m	Protein kinase superfamily protein	G
cassava4.1_007726m|PACid:17972173	cassava4.1_007726m	WD-40 repeat family protein / small nuclear ribonucleoprotein Prp4p-related	GP
cassava4.1_007730m|PACid:17961664	cassava4.1_007730m	PRP38 family protein	No
cassava4.1_007734m|PACid:17964710	cassava4.1_007734m	myb-like transcription factor family protein	No
cassava4.1_007736m|PACid:17991354	cassava4.1_007736m	Peptidase M20/M25/M40 family protein	GP

cassava4.1_007738m|PACid:17985604	cassava4.1_007738m	RNA-binding protein 47B	G
cassava4.1_007739m|PACid:17983211	cassava4.1_007739m	Arabidopsis thaliana protein of unknown function (DUF794)	No
cassava4.1_007741m|PACid:17960218	cassava4.1_007741m	Transmembrane Fragile-X-F-associated protein	No
cassava4.1_007743m|PACid:17987392	cassava4.1_007743m	alpha/beta-Hydrolases superfamily protein	G
cassava4.1_007750m|PACid:17971896	cassava4.1_007750m	26S proteasome regulatory subunit, putative (RPN5)	GP
cassava4.1_007756m|PACid:17993326	cassava4.1_007756m	Aldolase-type TIM barrel family protein	P
cassava4.1_007767m|PACid:17982867	cassava4.1_007767m	cobalt ion binding	No
cassava4.1_007773m|PACid:17981275	cassava4.1_007773m	phosphate transporter 4;6	No
cassava4.1_007774m|PACid:17970095	cassava4.1_007774m	ACT domain repeat 6	G
cassava4.1_007775m|PACid:17964243	cassava4.1_007775m	peptidase M20/M25/M40 family protein	P
cassava4.1_007776m|PACid:17972772	cassava4.1_007776m	Protein of unknown function (DUF1350)	G
cassava4.1_007777m|PACid:17993516	cassava4.1_007777m	Polyketide cyclase/dehydrase and lipid transport superfamily protein	No
cassava4.1_007779m|PACid:17990785	cassava4.1_007779m	DNAJ heat shock N-terminal domain-containing protein	G
cassava4.1_007780m|PACid:17982770	cassava4.1_007780m	GRAS family transcription factor	No
cassava4.1_007782m|PACid:17989090	cassava4.1_007782m	proline transporter 2	No
cassava4.1_007783m|PACid:17975199	cassava4.1_007783m	Polynucleotidyl transferase, ribonuclease H fold protein with HRDC domain	No
cassava4.1_007788m|PACid:17981700	cassava4.1_007788m	tetratricopeptide repeat (TPR)-containing protein	GP
cassava4.1_007790m|PACid:17992172	cassava4.1_007790m	Core-2/I-branching beta-1,6-N-acetylglucosaminyltransferase family protein	No
cassava4.1_007792m|PACid:17960167	cassava4.1_007792m	Melibiase family protein	GP
cassava4.1_007794m|PACid:17976702	cassava4.1_007794m	NAD-dependent glycerol-3-phosphate dehydrogenase family protein	No
cassava4.1_007795m|PACid:17963040	cassava4.1_007795m	actin-related protein 4	GP
cassava4.1_007798m|PACid:17991590	cassava4.1_007798m	proline transporter 2	No
cassava4.1_007803m|PACid:17971892	cassava4.1_007803m	NAD(P)-binding Rossmann-fold superfamily protein	GP
cassava4.1_007806m|PACid:17960748	cassava4.1_007806m	polypyrimidine tract-binding protein 2	G
cassava4.1_007807m|PACid:17980815	cassava4.1_007807m	dsRNA-binding protein 5	No
cassava4.1_007810m|PACid:17985627	cassava4.1_007810m	Lung seven transmembrane receptor family protein	G
cassava4.1_007811m|PACid:17966790	cassava4.1_007811m	TRAUCO	G
cassava4.1_007819m|PACid:17978396	cassava4.1_007819m	Protein of unknown function, DUF647	GP
cassava4.1_007824m|PACid:17967816	cassava4.1_007824m	CBL-interacting protein kinase 3	GP
cassava4.1_007825m|PACid:17993687	cassava4.1_007825m	acyl-CoA oxidase 4	GP
cassava4.1_007826m|PACid:17967814	cassava4.1_007826m	proteasome family protein	No
cassava4.1_007828m|PACid:17975964	cassava4.1_007828m	peptidase M20/M25/M40 family protein	P
cassava4.1_007830m|PACid:17967358	cassava4.1_007830m	Glycosyltransferase family 29 (sialyltransferase) family protein	G
cassava4.1_007834m|PACid:17975401	cassava4.1_007834m	CLP-similar protein 3	GP
cassava4.1_007836m|PACid:17980238	cassava4.1_007836m	Tetratricopeptide repeat (TPR)-like superfamily protein	G
cassava4.1_007849m|PACid:17969803	cassava4.1_007849m	SOS3-interacting protein 3	G
cassava4.1_007850m|PACid:17989444	cassava4.1_007850m	DEA(D/H)-box RNA helicase family protein	P
cassava4.1_007854m|PACid:17987900	cassava4.1_007854m	Transducin/WD40 repeat-like superfamily protein	GP
cassava4.1_007862m|PACid:17977751	cassava4.1_007862m	RNA-binding (RRM/RBD/RNP motifs) family protein	G

cassava4.1_007874m|PACid:17978716	cassava4.1_007874m	ferrochelatase 2	GP
cassava4.1_007876m|PACid:17966191	cassava4.1_007876m	FUS3-complementing gene 1	G
cassava4.1_007887m|PACid:17993275	cassava4.1_007887m	myb domain protein 61	No
cassava4.1_007888m|PACid:17991551	cassava4.1_007888m	Ubiquitin-specific protease family C19-related protein	G
cassava4.1_007889m|PACid:17991081	cassava4.1_007889m	lactate/malate dehydrogenase family protein	GP
cassava4.1_007894m|PACid:17981603	cassava4.1_007894m	Transducin/WD40 repeat-like superfamily protein	GP
cassava4.1_007895m|PACid:17962962	cassava4.1_007895m	Mov34/MPN/PAD-1 family protein	GP
cassava4.1_007900m|PACid:17981909	cassava4.1_007900m	Eukaryotic aspartyl protease family protein	GP
cassava4.1_007902m|PACid:17977010	cassava4.1_007902m	eukaryotic release factor 1-3	GP
cassava4.1_007906m|PACid:17963350	cassava4.1_007906m	Transducin/WD40 repeat-like superfamily protein	No
cassava4.1_007907m|PACid:17972592	cassava4.1_007907m	CBL-interacting protein kinase 9	G
cassava4.1_007909m|PACid:17981108	cassava4.1_007909m	dolichyl-diphosphooligosaccharide-protein glycosyltransferase 48kDa subunit family protein	GP
cassava4.1_007911m|PACid:17979111	cassava4.1_007911m	HXXXD-type acyl-transferase family protein	GP
cassava4.1_007912m|PACid:17960116	cassava4.1_007912m	polypyrimidine tract-binding protein 3	No
cassava4.1_007913m|PACid:17981998	cassava4.1_007913m	highly ABA-induced PP2C gene 3	G
cassava4.1_007915m|PACid:17990654	cassava4.1_007915m	myristoyl-CoA:protein N-myristoyltransferase	GP
cassava4.1_007916m|PACid:17986224	cassava4.1_007916m	RNA-binding protein-defense related 1	No
cassava4.1_007922m|PACid:17990949	cassava4.1_007922m	Copine (Calcium-dependent phospholipid-binding protein) family	GP
cassava4.1_007923m|PACid:17989111	cassava4.1_007923m	cytochrome c oxidase 10	G
cassava4.1_007931m|PACid:17987246	cassava4.1_007931m	Class I glutamine amidotransferase-like superfamily protein	GP
cassava4.1_007932m|PACid:17983483	cassava4.1_007932m	chorismate synthase, putative / 5-enolpyruvylshikimate-3-phosphate phospholyase, putative	GP
cassava4.1_007934m|PACid:17964305	cassava4.1_007934m	alanine:glyoxylate aminotransferase 3	No
cassava4.1_007935m|PACid:17975573	cassava4.1_007935m	Cytidine/deoxycytidylate deaminase family protein	GP
cassava4.1_007936m|PACid:17976836	cassava4.1_007936m	E2F transcription factor 1	No
cassava4.1_007939m|PACid:17972954	cassava4.1_007939m	HXXXD-type acyl-transferase family protein	No
cassava4.1_007942m|PACid:17963291	cassava4.1_007942m	UDP-D-glucuronate 4-epimerase 3	No
cassava4.1_007946m|PACid:17969287	cassava4.1_007946m	Nucleotide/sugar transporter family protein	GP
cassava4.1_007953m|PACid:17987460	cassava4.1_007953m	Mannose-6-phosphate isomerase, type I	GP
cassava4.1_007954m|PACid:17960007	cassava4.1_007954m	Lung seven transmembrane receptor family protein	GP
cassava4.1_007973m|PACid:17975763	cassava4.1_007973m	pyruvate dehydrogenase E1 alpha	GP
cassava4.1_007975m|PACid:17966088	cassava4.1_007975m	Protein of Unknown Function (DUF239)	No
cassava4.1_007977m|PACid:17988383	cassava4.1_007977m	Tetratricopeptide repeat (TPR)-like superfamily protein	No
cassava4.1_007980m|PACid:17961031	cassava4.1_007980m	monodehydroascorbate reductase 1	GP
cassava4.1_007981m|PACid:17968295	cassava4.1_007981m	Ergosterol biosynthesis ERG4/ERG24 family	GP
cassava4.1_007986m|PACid:17993852	cassava4.1_007986m	PDI-like 2-2	GP
cassava4.1_007988m|PACid:17967825	cassava4.1_007988m	Aldolase superfamily protein	No
cassava4.1_007990m|PACid:17986989	cassava4.1_007990m	Protein kinase superfamily protein	G
cassava4.1_007991m|PACid:17963442	cassava4.1_007991m	Exostosin family protein	G
cassava4.1_007994m|PACid:17966375	cassava4.1_007994m	Cyclin B2;3	No

cassava4.1_007998m|PACid:17991896	cassava4.1_007998m	highly ABA-induced PP2C gene 3	No
cassava4.1_007999m|PACid:17979374	cassava4.1_007999m	Glycosyl hydrolase superfamily protein	No
cassava4.1_008000m|PACid:17967906	cassava4.1_008000m		G
cassava4.1_008003m|PACid:17970670	cassava4.1_008003m	actin-related protein 6	No
cassava4.1_008007m|PACid:17973963	cassava4.1_008007m	FUS3-complementing gene 2	G
cassava4.1_008011m|PACid:17969263	cassava4.1_008011m	Protein kinase superfamily protein	G
cassava4.1_008013m|PACid:17977458	cassava4.1_008013m	glutamine synthetase 2	G
cassava4.1_008014m|PACid:17991989	cassava4.1_008014m	SOS3-interacting protein 4	G
cassava4.1_008025m|PACid:17966626	cassava4.1_008025m	cell cycle checkpoint control protein family	No
cassava4.1_008032m|PACid:17991366	cassava4.1_008032m	mraW methylase family protein	G
cassava4.1_008033m|PACid:17974838	cassava4.1_008033m	Ubiquitin carboxyl-terminal hydrolase family protein	G
cassava4.1_008034m|PACid:17962121	cassava4.1_008034m	Core-2/I-branching beta-1,6-N-acetylglucosaminyltransferase family protein	No
cassava4.1_008035m|PACid:17988702	cassava4.1_008035m	asparagine-linked glycosylation 3	G
cassava4.1_008036m|PACid:17991380	cassava4.1_008036m	Aldolase superfamily protein	GP
cassava4.1_008037m|PACid:17965883	cassava4.1_008037m	microfibrillar-associated protein-related	No
cassava4.1_008042m|PACid:17986271	cassava4.1_008042m	Transducin/WD40 repeat-like superfamily protein	No
cassava4.1_008043m|PACid:17987920	cassava4.1_008043m	RNA-binding protein 47C	G
cassava4.1_008044m|PACid:17976744	cassava4.1_008044m	SMAD/FHA domain-containing protein	G
cassava4.1_008045m|PACid:17981186	cassava4.1_008045m	hydroxycinnamoyl-CoA shikimate/quinate hydroxycinnamoyl transferase	GP
cassava4.1_008046m|PACid:17987768	cassava4.1_008046m	Protein kinase superfamily protein	G
cassava4.1_008050m|PACid:17980389	cassava4.1_008050m	CBL-interacting protein kinase 1	No
cassava4.1_008051m|PACid:17988775	cassava4.1_008051m	Mitochondrial substrate carrier family protein	No
cassava4.1_008053m|PACid:17987391	cassava4.1_008053m	Transducin/WD40 repeat-like superfamily protein	GP
cassava4.1_008058m|PACid:17973558	cassava4.1_008058m	AAA-type ATPase family protein	GP
cassava4.1_008061m|PACid:17973239	cassava4.1_008061m	Eukaryotic aspartyl protease family protein	GP
cassava4.1_008064m|PACid:17990147	cassava4.1_008064m	CYCLIN B2;4	No
cassava4.1_008065m|PACid:17993797	cassava4.1_008065m	UDP-D-glucuronate 4-epimerase 1	G
cassava4.1_008068m|PACid:17992173	cassava4.1_008068m	Glycosyl hydrolase superfamily protein	G
cassava4.1_008072m|PACid:17980542	cassava4.1_008072m	HXXXD-type acyl-transferase family protein	No
cassava4.1_008075m|PACid:17979360	cassava4.1_008075m	Leucine-rich repeat (LRR) family protein	No
cassava4.1_008076m|PACid:17988145	cassava4.1_008076m	PDI-like 5-2	GP
cassava4.1_008077m|PACid:17959954	cassava4.1_008077m	Ubiquitin carboxyl-terminal hydrolase family protein	No
cassava4.1_008080m|PACid:17991769	cassava4.1_008080m	histone deacetylase 9	G
cassava4.1_008084m|PACid:17978332	cassava4.1_008084m	Galactose oxidase/kelch repeat superfamily protein	No
cassava4.1_008092m|PACid:17973913	cassava4.1_008092m	MBOAT (membrane bound O-acyl transferase) family protein	No
cassava4.1_008093m|PACid:17960009	cassava4.1_008093m	NAC (No Apical Meristem) domain transcriptional regulator superfamily protein	No
cassava4.1_008107m|PACid:17967304	cassava4.1_008107m	P-loop containing nucleoside triphosphate hydrolases superfamily protein	GP
cassava4.1_008109m|PACid:17960533	cassava4.1_008109m	Nucleotide-diphospho-sugar transferase family protein	GP
cassava4.1_008116m|PACid:17964312	cassava4.1_008116m	serine/threonine protein kinase 1	G

cassava4.1_008118m|PACid:17965089	cassava4.1_008118m	NAD(P)-binding Rossmann-fold superfamily protein	No
cassava4.1_008119m|PACid:17964434	cassava4.1_008119m	Protein phosphatase 2C family protein	No
cassava4.1_008128m|PACid:17982260	cassava4.1_008128m	Protein of unknown function, DUF647	No
cassava4.1_008131m|PACid:17969281	cassava4.1_008131m	Protein of unknown function (DUF1005)	No
cassava4.1_008132m|PACid:17984406	cassava4.1_008132m	Eukaryotic aspartyl protease family protein	P
cassava4.1_008135m|PACid:17963353	cassava4.1_008135m	Clathrin adaptor complexes medium subunit family protein	GP
cassava4.1_008136m|PACid:17973838	cassava4.1_008136m	Protein phosphatase 2C family protein	No
cassava4.1_008141m|PACid:17967494	cassava4.1_008141m	Phosphatidate cytidylyltransferase family protein	No
cassava4.1_008143m|PACid:17983609	cassava4.1_008143m	DEAD/DEAH box RNA helicase family protein	GP
cassava4.1_008147m|PACid:17991109	cassava4.1_008147m	Protein phosphatase 2C family protein	No
cassava4.1_008162m|PACid:17960657	cassava4.1_008162m	protein phosphatase 2CA	GP
cassava4.1_008168m|PACid:17968060	cassava4.1_008168m	Core-2/I-branching beta-1,6-N-acetylglucosaminyltransferase family protein	G
cassava4.1_008175m|PACid:17984238	cassava4.1_008175m	pyrimidine 1	GP
cassava4.1_008177m|PACid:17992276	cassava4.1_008177m	Sucrose-6F-phosphate phosphohydrolase family protein	GP
cassava4.1_008178m|PACid:17991043	cassava4.1_008178m	Pseudouridine synthase family protein	No
cassava4.1_008182m|PACid:17973423	cassava4.1_008182m	RING/U-box superfamily protein	G
cassava4.1_008184m|PACid:17987602	cassava4.1_008184m	Galactose oxidase/kelch repeat superfamily protein	G
cassava4.1_008185m|PACid:17992447	cassava4.1_008185m	protein-protein interaction regulator family protein	GP
cassava4.1_008186m|PACid:17985833	cassava4.1_008186m	DEAD/DEAH box RNA helicase family protein	GP
cassava4.1_008188m|PACid:17975931	cassava4.1_008188m	SNF1-related protein kinase regulatory subunit gamma 1	GP
cassava4.1_008189m|PACid:17973989	cassava4.1_008189m	Protein of Unknown Function (DUF239)	G
cassava4.1_008197m|PACid:17962012	cassava4.1_008197m	SNARE associated Golgi protein family	G
cassava4.1_008201m|PACid:17966284	cassava4.1_008201m	Transmembrane amino acid transporter family protein	G
cassava4.1_008203m|PACid:17961367	cassava4.1_008203m	Spo11/DNA topoisomerase VI, subunit A protein	G
cassava4.1_008204m|PACid:17982279	cassava4.1_008204m	equilibrative nucleotide transporter 1	G
cassava4.1_008205m|PACid:17967417	cassava4.1_008205m		G
cassava4.1_008208m|PACid:17985786	cassava4.1_008208m	casein kinase I-like 3	No
cassava4.1_008209m|PACid:17962297	cassava4.1_008209m	Actin-like ATPase superfamily protein	No
cassava4.1_008212m|PACid:17970164	cassava4.1_008212m	regulatory particle triple-A 1A	GP
cassava4.1_008218m|PACid:17985745	cassava4.1_008218m	Flavin-binding monooxygenase family protein	No
cassava4.1_008219m|PACid:17970242	cassava4.1_008219m	Tyrosine transaminase family protein	G
cassava4.1_008220m|PACid:17974364	cassava4.1_008220m	glyceraldehyde-3-phosphate dehydrogenase of plastid 2	GP
cassava4.1_008228m|PACid:17987403	cassava4.1_008228m	Class I glutamine amidotransferase-like superfamily protein	No
cassava4.1_008230m|PACid:17989824	cassava4.1_008230m	CBS domain-containing protein with a domain of unknown function (DUF21)	G
cassava4.1_008231m|PACid:17971936	cassava4.1_008231m	Ca2+ activated outward rectifying K+ channel 6	No
cassava4.1_008232m|PACid:17971316	cassava4.1_008232m	GTP-binding protein-related	GP
cassava4.1_008233m|PACid:17977116	cassava4.1_008233m	Protein kinase superfamily protein	GP
cassava4.1_008239m|PACid:17994071	cassava4.1_008239m	aromatic and neutral transporter 1	G
cassava4.1_008240m|PACid:17985330	cassava4.1_008240m	Transducin/WD40 repeat-like superfamily protein	No

cassava4.1_008251m|PACid:17976353	cassava4.1_008251m	Transmembrane amino acid transporter family protein	No
cassava4.1_008255m|PACid:17975213	cassava4.1_008255m	BTB-POZ and MATH domain 4	G
cassava4.1_008260m|PACid:17983989	cassava4.1_008260m	Protein kinase superfamily protein	No
cassava4.1_008262m|PACid:17965225	cassava4.1_008262m	Proteasome component (PCI) domain protein	No
cassava4.1_008263m|PACid:17965851	cassava4.1_008263m	non-photochemical quenching 1	No
cassava4.1_008265m|PACid:17978291	cassava4.1_008265m	formate dehydrogenase	P
cassava4.1_008267m|PACid:17959776	cassava4.1_008267m	Phototropic-responsive NPH3 family protein	No
cassava4.1_008270m|PACid:17988124	cassava4.1_008270m	histidinol phosphate aminotransferase 1	P
cassava4.1_008272m|PACid:17975643	cassava4.1_008272m	alpha/beta-Hydrolases superfamily protein	No
cassava4.1_008274m|PACid:17983092	cassava4.1_008274m	iron regulated transporter 3	G
cassava4.1_008276m|PACid:17990448	cassava4.1_008276m	phenylalanyl-tRNA synthetase class IIc family protein	GP
cassava4.1_008280m|PACid:17975570	cassava4.1_008280m	Peptidase family M48 family protein	P
cassava4.1_008282m|PACid:17976313	cassava4.1_008282m	Trypsin family protein with PDZ domain	GP
cassava4.1_008284m|PACid:17985090	cassava4.1_008284m	GCR2-like 1	G
cassava4.1_008285m|PACid:17960025	cassava4.1_008285m	tubby like protein 1	G
cassava4.1_008286m|PACid:17979970	cassava4.1_008286m	cytochrome P450, family 77, subfamily A, polypeptide 5 pseudogene	GP
cassava4.1_008291m|PACid:17987000	cassava4.1_008291m	AGC (cAMP-dependent, cGMP-dependent and protein kinase C) kinase family protein	No
cassava4.1_008299m|PACid:17973516	cassava4.1_008299m	Transducin/WD40 repeat-like superfamily protein	No
cassava4.1_008300m|PACid:17991732	cassava4.1_008300m	Protein of unknown function (DUF760)	G
cassava4.1_008306m|PACid:17960087	cassava4.1_008306m	DHHC-type zinc finger family protein	GP
cassava4.1_008307m|PACid:17993202	cassava4.1_008307m	ATP-citrate lyase A-3	GP
cassava4.1_008310m|PACid:17966609	cassava4.1_008310m	regulatory particle triple-A ATPase 5A	GP
cassava4.1_008312m|PACid:17975703	cassava4.1_008312m	Acyl-CoA thioesterase family protein	G
cassava4.1_008314m|PACid:17988433	cassava4.1_008314m	Galactose oxidase/kelch repeat superfamily protein	G
cassava4.1_008316m|PACid:17967488	cassava4.1_008316m	Pyridoxal-5\'-phosphate-dependent enzyme family protein	G
cassava4.1_008317m|PACid:17973187	cassava4.1_008317m	RNA-binding (RRM/RBD/RNP motifs) family protein	GP
cassava4.1_008319m|PACid:17993121	cassava4.1_008319m	carbamoyl phosphate synthetase A	GP
cassava4.1_008320m|PACid:17984328	cassava4.1_008320m	Flavin-binding monooxygenase family protein	No
cassava4.1_008321m|PACid:17966381	cassava4.1_008321m	RNA binding;GTP binding	GP
cassava4.1_008324m|PACid:17976943	cassava4.1_008324m	CDP-diacylglycerol synthase 1	GP
cassava4.1_008333m|PACid:17983355	cassava4.1_008333m	Protein of unknown function (DUF300)	G
cassava4.1_008338m|PACid:17974795	cassava4.1_008338m	Translation elongation factor EF1B, gamma chain	No
cassava4.1_008344m|PACid:17964748	cassava4.1_008344m	ATP-citrate lyase A-1	P
cassava4.1_008351m|PACid:17977338	cassava4.1_008351m	alpha-amylase-like	No
cassava4.1_008352m|PACid:17993215	cassava4.1_008352m	Cystathionine beta-synthase (CBS) family protein	No
cassava4.1_008363m|PACid:17974970	cassava4.1_008363m	non-ATPase subunit 9	GP
cassava4.1_008367m|PACid:17988025	cassava4.1_008367m	EAP30/Vps36 family protein	G
cassava4.1_008369m|PACid:17974806	cassava4.1_008369m	shaggy-like protein kinase 41	No
cassava4.1_008376m|PACid:17977864	cassava4.1_008376m	calreticulin 3	GP

cassava4.1_008379m|PACid:17989489	cassava4.1_008379m	ACT domain-containing protein	G
cassava4.1_008382m|PACid:17976989	cassava4.1_008382m	magnesium chelatase i2	G
cassava4.1_008384m|PACid:17964108	cassava4.1_008384m	TRICHOME BIREFRINGENCE-LIKE 33	No
cassava4.1_008387m|PACid:17968420	cassava4.1_008387m	ATP citrate lyase (ACL) family protein	GP
cassava4.1_008388m|PACid:17975093	cassava4.1_008388m	Calcium-dependent lipid-binding (CaLB domain) family protein	No
cassava4.1_008395m|PACid:17960407	cassava4.1_008395m	cytochrome P450, family 722, subfamily A, polypeptide 1	G
cassava4.1_008396m|PACid:17965508	cassava4.1_008396m	fumarylacetoacetase, putative	GP
cassava4.1_008397m|PACid:17972263	cassava4.1_008397m	F-box family protein	No
cassava4.1_008398m|PACid:17990796	cassava4.1_008398m		No
cassava4.1_008399m|PACid:17990228	cassava4.1_008399m	FAD-dependent oxidoreductase family protein	GP
cassava4.1_008402m|PACid:17964926	cassava4.1_008402m	SEC14 cytosolic factor family protein / phosphoglyceride transfer family protein	No
cassava4.1_008404m|PACid:17962703	cassava4.1_008404m	F-box/RNI-like superfamily protein	No
cassava4.1_008405m|PACid:17990946	cassava4.1_008405m	O-fucosyltransferase family protein	No
cassava4.1_008407m|PACid:17982014	cassava4.1_008407m	Galactose oxidase/kelch repeat superfamily protein	GP
cassava4.1_008417m|PACid:17973462	cassava4.1_008417m	solanesyl diphosphate synthase 2	No
cassava4.1_008418m|PACid:17976969	cassava4.1_008418m	Galactose oxidase/kelch repeat superfamily protein	No
cassava4.1_008420m|PACid:17978384	cassava4.1_008420m	magnesium transporter 3	No
cassava4.1_008421m|PACid:17969343	cassava4.1_008421m	regulatory particle triple-A ATPase 3	GP
cassava4.1_008424m|PACid:17978644	cassava4.1_008424m	Phosphoglycerate mutase family protein	G
cassava4.1_008427m|PACid:17987484	cassava4.1_008427m	Protein kinase superfamily protein	No
cassava4.1_008437m|PACid:17984913	cassava4.1_008437m	regulatory particle triple-A ATPase 6A	GP
cassava4.1_008442m|PACid:17979560	cassava4.1_008442m	Auxin efflux carrier family protein	G
cassava4.1_008448m|PACid:17964275	cassava4.1_008448m	Protein of unknown function (DUF1005)	G
cassava4.1_008453m|PACid:17973912	cassava4.1_008453m	calreticulin 1b	GP
cassava4.1_008454m|PACid:17979606	cassava4.1_008454m	regulatory particle triple-A ATPase 6A	P
cassava4.1_008456m|PACid:17980451	cassava4.1_008456m	GMP synthase (glutamine-hydrolyzing), putative / glutamine amidotransferase, putative	No
cassava4.1_008460m|PACid:17961406	cassava4.1_008460m	C2H2-like zinc finger protein	G
cassava4.1_008463m|PACid:17972063	cassava4.1_008463m	Protein of Unknown Function (DUF239)	G
cassava4.1_008465m|PACid:17973987	cassava4.1_008465m	tryptophan biosynthesis 1	GP
cassava4.1_008467m|PACid:17984555	cassava4.1_008467m	branched-chain aminotransferase 3	P
cassava4.1_008470m|PACid:17979527	cassava4.1_008470m	aspartate aminotransferase 1	GP
cassava4.1_008476m|PACid:17970172	cassava4.1_008476m	Ubiquitin-conjugating enzyme/RWD-like protein	G
cassava4.1_008479m|PACid:17986945	cassava4.1_008479m	Arabidopsis thaliana protein of unknown function (DUF794)	G
cassava4.1_008482m|PACid:17967899	cassava4.1_008482m	Major facilitator superfamily protein	No
cassava4.1_008484m|PACid:17990842	cassava4.1_008484m	Major facilitator superfamily protein	G
cassava4.1_008498m|PACid:17962187	cassava4.1_008498m	NAD(P)-binding Rossmann-fold superfamily protein	No
cassava4.1_008499m|PACid:17971895	cassava4.1_008499m	Transducin/WD40 repeat-like superfamily protein	P
cassava4.1_008502m|PACid:17991679	cassava4.1_008502m	Protein kinase superfamily protein	No
cassava4.1_008506m|PACid:17994003	cassava4.1_008506m	casein kinase II, alpha chain 2	GP

cassava4.1_008507m|PACid:17963686	cassava4.1_008507m	Pectinacetylesterase family protein	No
cassava4.1_008508m|PACid:17965772	cassava4.1_008508m	phosphorylethanolamine cytidylyltransferase 1	P
cassava4.1_008510m|PACid:17972602	cassava4.1_008510m	DNAJ homologue 2	GP
cassava4.1_008511m|PACid:17993284	cassava4.1_008511m	Pectinacetylesterase family protein	No
cassava4.1_008513m|PACid:17980958	cassava4.1_008513m	geminivirus rep interacting kinase 2	G
cassava4.1_008515m|PACid:17973248	cassava4.1_008515m	NagB/RpiA/CoA transferase-like superfamily protein	GP
cassava4.1_008518m|PACid:17975349	cassava4.1_008518m	Sodium Bile acid symporter family	GP
cassava4.1_008519m|PACid:17982880	cassava4.1_008519m	Uroporphyrinogen decarboxylase	GP
cassava4.1_008521m|PACid:17984870	cassava4.1_008521m	NAD(P)-binding Rossmann-fold superfamily protein	GP
cassava4.1_008523m|PACid:17984556	cassava4.1_008523m	branched-chain amino acid aminotransferase 5 / branched-chain amino acid transaminase 5 (BCAT5)	No
cassava4.1_008525m|PACid:17969031	cassava4.1_008525m	Galactose oxidase/kelch repeat superfamily protein	G
cassava4.1_008533m|PACid:17989510	cassava4.1_008533m	Radical SAM superfamily protein	G
cassava4.1_008539m|PACid:17977109	cassava4.1_008539m	Acyl-CoA N-acyltransferases (NAT) superfamily protein	No
cassava4.1_008540m|PACid:17970804	cassava4.1_008540m	protein kinase 2B	G
cassava4.1_008541m|PACid:17966789	cassava4.1_008541m	RNA-binding (RRM/RBD/RNP motifs) family protein	GP
cassava4.1_008542m|PACid:17992150	cassava4.1_008542m	GCR2-like 2	GP
cassava4.1_008546m|PACid:17964387	cassava4.1_008546m	Ubiquitin carboxyl-terminal hydrolase family protein	G
cassava4.1_008551m|PACid:17968478	cassava4.1_008551m	Glycosyl hydrolase superfamily protein	No
cassava4.1_008552m|PACid:17989108	cassava4.1_008552m	disproportionating enzyme	GP
cassava4.1_008555m|PACid:17969246	cassava4.1_008555m	scramblase-related	P
cassava4.1_008556m|PACid:17983863	cassava4.1_008556m	alpha/beta-Hydrolases superfamily protein	G
cassava4.1_008559m|PACid:17981064	cassava4.1_008559m	TBP-associated factor 2	No
cassava4.1_008563m|PACid:17960893	cassava4.1_008563m	FAD-dependent oxidoreductase family protein	No
cassava4.1_008565m|PACid:17967091	cassava4.1_008565m	ENTH/VHS/GAT family protein	G
cassava4.1_008566m|PACid:17959994	cassava4.1_008566m	Cation efflux family protein	G
cassava4.1_008567m|PACid:17970476	cassava4.1_008567m	GHMP kinase family protein	GP
cassava4.1_008571m|PACid:17979276	cassava4.1_008571m	Protein kinase superfamily protein	No
cassava4.1_008575m|PACid:17972164	cassava4.1_008575m	Endosomal targeting BRO1-like domain-containing protein	G
cassava4.1_008579m|PACid:17974146	cassava4.1_008579m	ADP-glucose pyrophosphorylase family protein	GP
cassava4.1_008588m|PACid:17971861	cassava4.1_008588m	Pectinacetylesterase family protein	G
cassava4.1_008594m|PACid:17960989	cassava4.1_008594m	Nucleotide-diphospho-sugar transferases superfamily protein	No
cassava4.1_008595m|PACid:17982914	cassava4.1_008595m	metacaspase 4	GP
cassava4.1_008596m|PACid:17966304	cassava4.1_008596m	tubby like protein 2	G
cassava4.1_008600m|PACid:17975440	cassava4.1_008600m	plant U-box 23	No
cassava4.1_008604m|PACid:17979400	cassava4.1_008604m	cytosolic NADP+-dependent isocitrate dehydrogenase	GP
cassava4.1_008605m|PACid:17969223	cassava4.1_008605m	FAD-dependent oxidoreductase family protein	G
cassava4.1_008610m|PACid:17962081	cassava4.1_008610m	Serinc-domain containing serine and sphingolipid biosynthesis protein	No
cassava4.1_008615m|PACid:17985176	cassava4.1_008615m	rRNA processing protein-related	No
cassava4.1_008619m|PACid:17964925	cassava4.1_008619m	TRICHOME BIREFRINGENCE-LIKE 34	No

cassava4.1_008620m|PACid:17975078	cassava4.1_008620m	trigger factor type chaperone family protein	GP
cassava4.1_008622m|PACid:17971637	cassava4.1_008622m	squalene synthase 1	GP
cassava4.1_008627m|PACid:17980675	cassava4.1_008627m	beta-ureidopropionase	GP
cassava4.1_008628m|PACid:17990704	cassava4.1_008628m	proteasome family protein	No
cassava4.1_008631m|PACid:17990653	cassava4.1_008631m	eukaryotic translation initiation factor 4A1	GP
cassava4.1_008637m|PACid:17981485	cassava4.1_008637m	splicing factor Prp18 family protein	G
cassava4.1_008639m|PACid:17973178	cassava4.1_008639m	eukaryotic translation initiation factor 4A1	GP
cassava4.1_008642m|PACid:17993148	cassava4.1_008642m	Ribosomal protein S24/S35, mitochondrial	GP
cassava4.1_008647m|PACid:17979232	cassava4.1_008647m	Protein kinase superfamily protein	G
cassava4.1_008653m|PACid:17971716	cassava4.1_008653m	pfkB-like carbohydrate kinase family protein	GP
cassava4.1_008657m|PACid:17969121	cassava4.1_008657m	malate dehydrogenase	GP
cassava4.1_008659m|PACid:17961571	cassava4.1_008659m	LUC7 related protein	P
cassava4.1_008660m|PACid:17977031	cassava4.1_008660m	Serinc-domain containing serine and sphingolipid biosynthesis protein	G
cassava4.1_008662m|PACid:17990003	cassava4.1_008662m	manganese tracking factor for mitochondrial SOD2	G
cassava4.1_008665m|PACid:17982449	cassava4.1_008665m	ubiquitin-associated (UBA)/TS-N domain-containing protein	GP
cassava4.1_008668m|PACid:17959729	cassava4.1_008668m	high cyclic electron flow 1	No
cassava4.1_008670m|PACid:17968613	cassava4.1_008670m	ACT-like superfamily protein	G
cassava4.1_008671m|PACid:17988271	cassava4.1_008671m	Basic-leucine zipper (bZIP) transcription factor family protein	G
cassava4.1_008672m|PACid:17972554	cassava4.1_008672m	LisH/CRA/RING-U-box domains-containing protein	No
cassava4.1_008674m|PACid:17983529	cassava4.1_008674m	Proteasome component (PCI) domain protein	P
cassava4.1_008677m|PACid:17961835	cassava4.1_008677m	ribosomal protein S1	GP
cassava4.1_008678m|PACid:17969935	cassava4.1_008678m	G protein coupled receptor	No
cassava4.1_008681m|PACid:17982926	cassava4.1_008681m	serine carboxypeptidase-like 51	No
cassava4.1_008685m|PACid:17961541	cassava4.1_008685m	ARM repeat superfamily protein	P
cassava4.1_008687m|PACid:17989025	cassava4.1_008687m	ATP-dependent caseinolytic (Clp) protease/crotonase family protein	No
cassava4.1_008690m|PACid:17975185	cassava4.1_008690m	glutamate dehydrogenase 1	GP
cassava4.1_008693m|PACid:17985306	cassava4.1_008693m	glutamate dehydrogenase 2	P
cassava4.1_008700m|PACid:17992487	cassava4.1_008700m	oligouridylate binding protein 1B	No
cassava4.1_008709m|PACid:17982888	cassava4.1_008709m	Plant protein of unknown function (DUF828)	No
cassava4.1_008713m|PACid:17965917	cassava4.1_008713m	glutamate dehydrogenase 1	P
cassava4.1_008714m|PACid:17968887	cassava4.1_008714m	general transcription factor II H2	G
cassava4.1_008718m|PACid:17983942	cassava4.1_008718m	purine permease 4	G
cassava4.1_008719m|PACid:17989767	cassava4.1_008719m	basic region/leucine zipper transcription factor 16	No
cassava4.1_008721m|PACid:17982085	cassava4.1_008721m	Basic-leucine zipper (bZIP) transcription factor family protein	G
cassava4.1_008730m|PACid:17973044	cassava4.1_008730m	protein arginine methyltransferase 6	G
cassava4.1_008736m|PACid:17992488	cassava4.1_008736m	oligouridylate binding protein 1B	G
cassava4.1_008738m|PACid:17971555	cassava4.1_008738m	GroES-like zinc-binding alcohol dehydrogenase family protein	P
cassava4.1_008740m|PACid:17986182	cassava4.1_008740m	Ubiquitin carboxyl-terminal hydrolase family protein	G
cassava4.1_008741m|PACid:17977496	cassava4.1_008741m	Transducin family protein / WD-40 repeat family protein	No

cassava4.1_008742m|PACid:17961913	cassava4.1_008742m	PLANT HOMOLOGOUS TO PARAFIBROMIN	GP
cassava4.1_008744m|PACid:17986274	cassava4.1_008744m	BTB-POZ and MATH domain 2	G
cassava4.1_008745m|PACid:17968386	cassava4.1_008745m	Glutathione S-transferase family protein	GP
cassava4.1_008747m|PACid:17989315	cassava4.1_008747m	eukaryotic initiation factor 4A-III	P
cassava4.1_008753m|PACid:17986965	cassava4.1_008753m	DNAJ heat shock family protein	GP
cassava4.1_008754m|PACid:17973575	cassava4.1_008754m	alpha-amylase-like 2	No
cassava4.1_008759m|PACid:17988706	cassava4.1_008759m	protein kinase 1B	No
cassava4.1_008766m|PACid:17988321	cassava4.1_008766m	ARG1-like 1	G
cassava4.1_008773m|PACid:17980283	cassava4.1_008773m	DNA primases;DNA primases	No
cassava4.1_008774m|PACid:17992929	cassava4.1_008774m	PPDK regulatory protein	G
cassava4.1_008785m|PACid:17977755	cassava4.1_008785m	UDP-Glycosyltransferase superfamily protein	GP
cassava4.1_008786m|PACid:17986977	cassava4.1_008786m		No
cassava4.1_008791m|PACid:17973856	cassava4.1_008791m	Protein kinase superfamily protein	No
cassava4.1_008797m|PACid:17960678	cassava4.1_008797m	BTB/POZ/MATH-domains containing protein	G
cassava4.1_008799m|PACid:17987909	cassava4.1_008799m	phospholipase A 2A	GP
cassava4.1_008804m|PACid:17970021	cassava4.1_008804m	Glucose-6-phosphate/phosphate translocator-related	No
cassava4.1_008812m|PACid:17990583	cassava4.1_008812m	Ribosomal protein L4/L1 family	GP
cassava4.1_008814m|PACid:17973304	cassava4.1_008814m	nodulin MtN21 /EamA-like transporter family protein	No
cassava4.1_008817m|PACid:17970395	cassava4.1_008817m	Transketolase family protein	G
cassava4.1_008818m|PACid:17959836	cassava4.1_008818m	Zinc finger protein 622	GP
cassava4.1_008821m|PACid:17976991	cassava4.1_008821m	Peptidase C13 family	GP
cassava4.1_008822m|PACid:17968848	cassava4.1_008822m	Glycine cleavage T-protein family	GP
cassava4.1_008826m|PACid:17994054	cassava4.1_008826m	Phosphatidic acid phosphatase (PAP2) family protein	G
cassava4.1_008827m|PACid:17994113	cassava4.1_008827m	Cation efflux family protein	No
cassava4.1_008828m|PACid:17973023	cassava4.1_008828m	RNI-like superfamily protein	G
cassava4.1_008832m|PACid:17991334	cassava4.1_008832m	MALE GAMETOPHYTE DEFECTIVE 2	No
cassava4.1_008836m|PACid:17970389	cassava4.1_008836m	P-loop containing nucleoside triphosphate hydrolases superfamily protein	G
cassava4.1_008837m|PACid:17967792	cassava4.1_008837m	Transcription factor IIA, alpha/beta subunit	G
cassava4.1_008840m|PACid:17977149	cassava4.1_008840m	Protein kinase superfamily protein	P
cassava4.1_008841m|PACid:17971269	cassava4.1_008841m	NAD(P)-binding Rossmann-fold superfamily protein	GP
cassava4.1_008844m|PACid:17983647	cassava4.1_008844m	aspartate aminotransferase 2	GP
cassava4.1_008851m|PACid:17976558	cassava4.1_008851m	Chaperone DnaJ-domain superfamily protein	GP
cassava4.1_008852m|PACid:17985114	cassava4.1_008852m	Pectin lyase-like superfamily protein	No
cassava4.1_008855m|PACid:17963375	cassava4.1_008855m	Pectate lyase family protein	No
cassava4.1_008856m|PACid:17978393	cassava4.1_008856m	ureide permease 2	G
cassava4.1_008859m|PACid:17962582	cassava4.1_008859m	ATP phosphoribosyl transferase 2	No
cassava4.1_008865m|PACid:17984578	cassava4.1_008865m	heptahelical protein 4	G
cassava4.1_008871m|PACid:17986260	cassava4.1_008871m	tubby like protein 3	No
cassava4.1_008878m|PACid:17967641	cassava4.1_008878m	FASCICLIN-like arabinogalactan 1	GP

cassava4.1_008880m|PACid:17975599	cassava4.1_008880m	Galactose oxidase/kelch repeat superfamily protein	G
cassava4.1_008885m|PACid:17990881	cassava4.1_008885m	P-loop containing nucleoside triphosphate hydrolases superfamily protein	No
cassava4.1_008887m|PACid:17972799	cassava4.1_008887m	Calcineurin-like metallo-phosphoesterase superfamily protein	G
cassava4.1_008888m|PACid:17970374	cassava4.1_008888m	Galactose oxidase/kelch repeat superfamily protein	G
cassava4.1_008889m|PACid:17975088	cassava4.1_008889m	Galactosyltransferase family protein	No
cassava4.1_008894m|PACid:17973923	cassava4.1_008894m	phosphoribulokinase	GP
cassava4.1_008899m|PACid:17993995	cassava4.1_008899m	Acetamidase/Formamidase family protein	P
cassava4.1_008904m|PACid:17969649	cassava4.1_008904m	nodulin MtN21 /EamA-like transporter family protein	G
cassava4.1_008905m|PACid:17981851	cassava4.1_008905m	protein kinase 1B	G
cassava4.1_008906m|PACid:17971852	cassava4.1_008906m	Ubiquitin carboxyl-terminal hydrolase family protein	No
cassava4.1_008908m|PACid:17985373	cassava4.1_008908m	purple acid phosphatase 28	No
cassava4.1_008913m|PACid:17992528	cassava4.1_008913m		No
cassava4.1_008916m|PACid:17989232	cassava4.1_008916m	acetoacetyl-CoA thiolase 2	GP
cassava4.1_008920m|PACid:17978275	cassava4.1_008920m	Minichromosome maintenance (MCM2/3/5) family protein	GP
cassava4.1_008921m|PACid:17970137	cassava4.1_008921m	Protein of unknown function (DUF707)	G
cassava4.1_008923m|PACid:17981155	cassava4.1_008923m	Pectinacetylesterase family protein	P
cassava4.1_008924m|PACid:17991491	cassava4.1_008924m	arogenate dehydratase 1	G
cassava4.1_008925m|PACid:17986488	cassava4.1_008925m	Protein of unknown function (DUF707)	No
cassava4.1_008926m|PACid:17977222	cassava4.1_008926m	alpha/beta-Hydrolases superfamily protein	G
cassava4.1_008930m|PACid:17985157	cassava4.1_008930m	methionine aminopeptidase 1A	No
cassava4.1_008933m|PACid:17979976	cassava4.1_008933m	MAP kinase 6	G
cassava4.1_008934m|PACid:17971734	cassava4.1_008934m	ARM repeat superfamily protein	GP
cassava4.1_008935m|PACid:17970489	cassava4.1_008935m	ARF-GAP domain 8	GP
cassava4.1_008940m|PACid:17982030	cassava4.1_008940m	regulatory particle non-ATPase 10	P
cassava4.1_008946m|PACid:17981231	cassava4.1_008946m	2A phosphatase associated protein of 46 kD	G
cassava4.1_008950m|PACid:17963845	cassava4.1_008950m	S-adenosyl-L-methionine-dependent methyltransferases superfamily protein	No
cassava4.1_008951m|PACid:17967668	cassava4.1_008951m	guanylate kinase	No
cassava4.1_008953m|PACid:17972547	cassava4.1_008953m	Protein of unknown function (DUF155)	No
cassava4.1_008956m|PACid:17967606	cassava4.1_008956m	Protein of unknown function (DUF3537)	No
cassava4.1_008960m|PACid:17963056	cassava4.1_008960m	glyceraldehyde 3-phosphate dehydrogenase A subunit 2	GP
cassava4.1_008963m|PACid:17986318	cassava4.1_008963m	chloroplast stem-loop binding protein of 41 kDa	GP
cassava4.1_008969m|PACid:17973958	cassava4.1_008969m	dicarboxylate diiron protein, putative (Crd1)	G
cassava4.1_008972m|PACid:17993474	cassava4.1_008972m	NAD(P)-linked oxidoreductase superfamily protein	No
cassava4.1_008974m|PACid:17973458	cassava4.1_008974m	Protein kinase superfamily protein	G
cassava4.1_008977m|PACid:17979467	cassava4.1_008977m	nodulin MtN21 /EamA-like transporter family protein	G
cassava4.1_008978m|PACid:17990898	cassava4.1_008978m	Inositol monophosphatase family protein	GP
cassava4.1_008981m|PACid:17959995	cassava4.1_008981m	Galactosyltransferase family protein	G
cassava4.1_008988m|PACid:17962047	cassava4.1_008988m	magnesium transporter 9	G
cassava4.1_008993m|PACid:17970305	cassava4.1_008993m	Protein kinase superfamily protein	G

cassava4.1_008996m|PACid:17977704	cassava4.1_008996m	oxidoreductases, acting on the aldehyde or oxo group of donors, NAD or NADP as acceptor;copper ion binding	P
cassava4.1_009000m|PACid:17991547	cassava4.1_009000m	Coproporphyrinogen III oxidase	GP
cassava4.1_009004m|PACid:17981948	cassava4.1_009004m	alanine:glyoxylate aminotransferase	No
cassava4.1_009005m|PACid:17990714	cassava4.1_009005m	arginine methyltransferase 11	G
cassava4.1_009007m|PACid:17978231	cassava4.1_009007m		No
cassava4.1_009013m|PACid:17977243	cassava4.1_009013m	3-ketoacyl-acyl carrier protein synthase III	GP
cassava4.1_009020m|PACid:17968301	cassava4.1_009020m	phosphoglycerate kinase	GP
cassava4.1_009023m|PACid:17969919	cassava4.1_009023m	C2H2-like zinc finger protein	No
cassava4.1_009024m|PACid:17990434	cassava4.1_009024m	tubby like protein 8	No
cassava4.1_009025m|PACid:17988650	cassava4.1_009025m	DNA/RNA-binding protein Kin17, conserved region	No
cassava4.1_009027m|PACid:17973673	cassava4.1_009027m	Inositol monophosphatase family protein	GP
cassava4.1_009029m|PACid:17976124	cassava4.1_009029m	RNA-binding (RRM/RBD/RNP motifs) family protein	G
cassava4.1_009031m|PACid:17987063	cassava4.1_009031m	Core-2/I-branching beta-1,6-N-acetylglucosaminyltransferase family protein	No
cassava4.1_009035m|PACid:17987606	cassava4.1_009035m	Rad23 UV excision repair protein family	P
cassava4.1_009036m|PACid:17968647	cassava4.1_009036m	Mitochondrial substrate carrier family protein	No
cassava4.1_009040m|PACid:17984774	cassava4.1_009040m	peroxin 10	No
cassava4.1_009042m|PACid:17959988	cassava4.1_009042m	esterase/lipase/thioesterase family protein	No
cassava4.1_009044m|PACid:17975137	cassava4.1_009044m	ATP binding	No
cassava4.1_009048m|PACid:17969948	cassava4.1_009048m	protochlorophyllide oxidoreductase A	GP
cassava4.1_009051m|PACid:17979184	cassava4.1_009051m	ATP-dependent caseinolytic (Clp) protease/crotonase family protein	P
cassava4.1_009054m|PACid:17966495	cassava4.1_009054m		No
cassava4.1_009055m|PACid:17959751	cassava4.1_009055m	glucose-6-phosphate/phosphate translocator 2	GP
cassava4.1_009059m|PACid:17964295	cassava4.1_009059m	WRKY DNA-binding protein 33	G
cassava4.1_009060m|PACid:17981251	cassava4.1_009060m	Protein kinase superfamily protein	No
cassava4.1_009061m|PACid:17969679	cassava4.1_009061m	AAA-type ATPase family protein	GP
cassava4.1_009063m|PACid:17987150	cassava4.1_009063m	protochlorophyllide oxidoreductase A	P
cassava4.1_009064m|PACid:17982556	cassava4.1_009064m	Auxin-responsive family protein	G
cassava4.1_009065m|PACid:17969844	cassava4.1_009065m	DNAJ heat shock N-terminal domain-containing protein	G
cassava4.1_009066m|PACid:17973250	cassava4.1_009066m	P-loop containing nucleoside triphosphate hydrolases superfamily protein	GP
cassava4.1_009068m|PACid:17968046	cassava4.1_009068m	Auxin-responsive family protein	G
cassava4.1_009073m|PACid:17989127	cassava4.1_009073m	Phosphoribosyltransferase family protein	GP
cassava4.1_009076m|PACid:17979064	cassava4.1_009076m	arginine biosynthesis protein ArgJ family	No
cassava4.1_009080m|PACid:17979476	cassava4.1_009080m	Protein kinase superfamily protein	G
cassava4.1_009082m|PACid:17968105	cassava4.1_009082m	cyclin-dependent kinase D1;3	No
cassava4.1_009088m|PACid:17961529	cassava4.1_009088m	HCP-like superfamily protein with MYND-type zinc finger	G
cassava4.1_009089m|PACid:17962759	cassava4.1_009089m	PLC-like phosphodiesterases superfamily protein	GP
cassava4.1_009102m|PACid:17962829	cassava4.1_009102m	Transmembrane amino acid transporter family protein	G
cassava4.1_009104m|PACid:17966559	cassava4.1_009104m	Nucleotidylyl transferase superfamily protein	No
cassava4.1_009112m|PACid:17974690	cassava4.1_009112m	Protein phosphatase 2C family protein	G

cassava4.1_009119m|PACid:17970453	cassava4.1_009119m	oxophytodienoate-reductase 3	P
cassava4.1_009120m|PACid:17969706	cassava4.1_009120m	Protein kinase superfamily protein	G
cassava4.1_009122m|PACid:17993047	cassava4.1_009122m	Galactosyltransferase family protein	G
cassava4.1_009125m|PACid:17976348	cassava4.1_009125m	Pentatricopeptide repeat (PPR) superfamily protein	No
cassava4.1_009127m|PACid:17982950	cassava4.1_009127m	guanylate kinase	G
cassava4.1_009128m|PACid:17971858	cassava4.1_009128m	NAD(P)-binding Rossmann-fold superfamily protein	No
cassava4.1_009129m|PACid:17991686	cassava4.1_009129m	DNA repair (Rad51) family protein	No
cassava4.1_009134m|PACid:17989594	cassava4.1_009134m	AAR2 protein family	G
cassava4.1_009135m|PACid:17960417	cassava4.1_009135m	Proteasome component (PCI) domain protein	GP
cassava4.1_009138m|PACid:17976427	cassava4.1_009138m	myb domain protein 16	No
cassava4.1_009140m|PACid:17966251	cassava4.1_009140m	fructose-bisphosphate aldolase 2	GP
cassava4.1_009141m|PACid:17970205	cassava4.1_009141m	RNA-binding (RRM/RBD/RNP motifs) family protein	No
cassava4.1_009148m|PACid:17970518	cassava4.1_009148m	S-adenosyl-L-methionine-dependent methyltransferases superfamily protein	G
cassava4.1_009154m|PACid:17988182	cassava4.1_009154m	choline kinase 1	G
cassava4.1_009157m|PACid:17981505	cassava4.1_009157m	phosphoribosylformylglycinamidine cyclo-ligase, chloroplast / phosphoribosyl-aminoimidazole synthetase	GP
cassava4.1_009165m|PACid:17967236	cassava4.1_009165m	pseudouridine synthase family protein	G
cassava4.1_009168m|PACid:17972031	cassava4.1_009168m	TBP-associated factor 15	G
cassava4.1_009169m|PACid:17971259	cassava4.1_009169m	Protein of unknown function (DUF3411)	GP
cassava4.1_009171m|PACid:17981959	cassava4.1_009171m	RHOMBOID-like 1	No
cassava4.1_009172m|PACid:17971693	cassava4.1_009172m	NAD(P)-binding Rossmann-fold superfamily protein	P
cassava4.1_009173m|PACid:17977117	cassava4.1_009173m	Plant stearoyl-acyl-carrier-protein desaturase family protein	GP
cassava4.1_009176m|PACid:17972630	cassava4.1_009176m	Core-2/I-branching beta-1,6-N-acetylglucosaminyltransferase family protein	No
cassava4.1_009179m|PACid:17960193	cassava4.1_009179m	Protein kinase superfamily protein	G
cassava4.1_009181m|PACid:17982163	cassava4.1_009181m	alpha/beta-Hydrolases superfamily protein	GP
cassava4.1_009184m|PACid:17972098	cassava4.1_009184m	histone deacetylase 2	No
cassava4.1_009188m|PACid:17969002	cassava4.1_009188m	alpha/beta-Hydrolases superfamily protein	GP
cassava4.1_009189m|PACid:17987086	cassava4.1_009189m	cytochrome P450, family 97, subfamily B, polypeptide 3	No
cassava4.1_009192m|PACid:17983900	cassava4.1_009192m	Fes1A	G
cassava4.1_009197m|PACid:17983099	cassava4.1_009197m	gamma vacuolar processing enzyme	No
cassava4.1_009200m|PACid:17960133	cassava4.1_009200m	Glycine cleavage T-protein family	No
cassava4.1_009202m|PACid:17963481	cassava4.1_009202m	Plant stearoyl-acyl-carrier-protein desaturase family protein	G
cassava4.1_009203m|PACid:17965578	cassava4.1_009203m	Target of Myb protein 1	G
cassava4.1_009206m|PACid:17991302	cassava4.1_009206m	Chalcone and stilbene synthase family protein	No
cassava4.1_009207m|PACid:17988140	cassava4.1_009207m	transducin family protein / WD-40 repeat family protein	G
cassava4.1_009208m|PACid:17970283	cassava4.1_009208m	Cation efflux family protein	G
cassava4.1_009210m|PACid:17984512	cassava4.1_009210m	Plant stearoyl-acyl-carrier-protein desaturase family protein	G
cassava4.1_009212m|PACid:17966212	cassava4.1_009212m	Protein phosphatase 2C family protein	G
cassava4.1_009213m|PACid:17964900	cassava4.1_009213m	UbiA prenyltransferase family protein	No
cassava4.1_009217m|PACid:17976496	cassava4.1_009217m	Aldolase superfamily protein	GP

cassava4.1_009220m|PACid:17973453	cassava4.1_009220m	NAD(P)-binding Rossmann-fold superfamily protein	GP
cassava4.1_009223m|PACid:17993048	cassava4.1_009223m	Galactosyltransferase family protein	G
cassava4.1_009224m|PACid:17967085	cassava4.1_009224m	photosystem II stability/assembly factor, chloroplast (HCF136)	No
cassava4.1_009227m|PACid:17975542	cassava4.1_009227m	metallopeptidase M24 family protein	G
cassava4.1_009228m|PACid:17982835	cassava4.1_009228m	sulfite oxidase	No
cassava4.1_009229m|PACid:17963963	cassava4.1_009229m	P-loop containing nucleoside triphosphate hydrolases superfamily protein	No
cassava4.1_009230m|PACid:17979274	cassava4.1_009230m	GTP binding	GP
cassava4.1_009231m|PACid:17963564	cassava4.1_009231m	Class I glutamine amidotransferase-like superfamily protein	GP
cassava4.1_009232m|PACid:17970155	cassava4.1_009232m	Eukaryotic translation initiation factor 2B (eIF-2B) family protein	GP
cassava4.1_009235m|PACid:17983010	cassava4.1_009235m	Pyridoxal phosphate (PLP)-dependent transferases superfamily protein	No
cassava4.1_009241m|PACid:17970685	cassava4.1_009241m	Nucleoside transporter family protein	No
cassava4.1_009245m|PACid:17963775	cassava4.1_009245m	S-adenosylmethionine synthetase 2	GP
cassava4.1_009248m|PACid:17968542	cassava4.1_009248m	CW7	No
cassava4.1_009250m|PACid:17980924	cassava4.1_009250m	DHBP synthase RibB-like alpha/beta domain;GTP cyclohydrolase II	No
cassava4.1_009251m|PACid:17981482	cassava4.1_009251m	galacturonosyltransferase-like 7	G
cassava4.1_009253m|PACid:17990586	cassava4.1_009253m	RHOMBOID-like 1	No
cassava4.1_009259m|PACid:17987589	cassava4.1_009259m	Regulator of chromosome condensation (RCC1) family protein	GP
cassava4.1_009262m|PACid:17973335	cassava4.1_009262m	nuclear matrix protein-related	No
cassava4.1_009263m|PACid:17963679	cassava4.1_009263m	Protein phosphatase 2C family protein	No
cassava4.1_009269m|PACid:17977657	cassava4.1_009269m	proline iminopeptidase	GP
cassava4.1_009270m|PACid:17959877	cassava4.1_009270m	EamA-like transporter family	No
cassava4.1_009272m|PACid:17986478	cassava4.1_009272m	beta-1,4-N-acetylglucosaminyltransferase family protein	G
cassava4.1_009273m|PACid:17970376	cassava4.1_009273m	Protein of unknown function, DUF642	No
cassava4.1_009276m|PACid:17986579	cassava4.1_009276m	Core-2/I-branching beta-1,6-N-acetylglucosaminyltransferase family protein	No
cassava4.1_009280m|PACid:17991702	cassava4.1_009280m	arogenate dehydratase 2	G
cassava4.1_009281m|PACid:17964674	cassava4.1_009281m	S-adenosylmethionine synthetase 2	No
cassava4.1_009286m|PACid:17980521	cassava4.1_009286m	glucose 6-phosphate/phosphate translocator 1	No
cassava4.1_009289m|PACid:17987988	cassava4.1_009289m	G protein alpha subunit 1	No
cassava4.1_009293m|PACid:17964454	cassava4.1_009293m	Serine protease inhibitor (SERPIN) family protein	GP
cassava4.1_009294m|PACid:17979422	cassava4.1_009294m	Class I glutamine amidotransferase-like superfamily protein	P
cassava4.1_009299m|PACid:17961709	cassava4.1_009299m	DHHC-type zinc finger family protein	G
cassava4.1_009304m|PACid:17971454	cassava4.1_009304m	tubby like protein 3	No
cassava4.1_009305m|PACid:17989321	cassava4.1_009305m	strictosidine synthase-like 3	GP
cassava4.1_009306m|PACid:17978891	cassava4.1_009306m	phosphate transporter traffic facilitator1	G
cassava4.1_009307m|PACid:17990680	cassava4.1_009307m		G
cassava4.1_009309m|PACid:17967735	cassava4.1_009309m	4-(cytidine 5\'-phospho)-2-C-methyl-D-erithritol kinase	GP
cassava4.1_009313m|PACid:17993633	cassava4.1_009313m	zinc ion binding;nucleic acid binding;hydrolases, acting on acid anhydrides	No
cassava4.1_009317m|PACid:17992098	cassava4.1_009317m	Core-2/I-branching beta-1,6-N-acetylglucosaminyltransferase family protein	G
cassava4.1_009325m|PACid:17988915	cassava4.1_009325m	zinc ion binding	No

cassava4.1_009328m|PACid:17980814	cassava4.1_009328m	DHHC-type zinc finger family protein	No
cassava4.1_009336m|PACid:17978235	cassava4.1_009336m	Homeodomain-like superfamily protein	G
cassava4.1_009342m|PACid:17987951	cassava4.1_009342m	Oxidoreductase, zinc-binding dehydrogenase family protein	GP
cassava4.1_009343m|PACid:17981298	cassava4.1_009343m	S-adenosyl-L-methionine-dependent methyltransferases superfamily protein	G
cassava4.1_009345m|PACid:17960109	cassava4.1_009345m	Protein of unknown function (DUF793)	G
cassava4.1_009346m|PACid:17979446	cassava4.1_009346m	S-adenosyl-L-methionine-dependent methyltransferases superfamily protein	G
cassava4.1_009347m|PACid:17991355	cassava4.1_009347m	nodulin MtN21 /EamA-like transporter family protein	G
cassava4.1_009348m|PACid:17989913	cassava4.1_009348m	Nucleic acid-binding proteins superfamily	GP
cassava4.1_009354m|PACid:17986816	cassava4.1_009354m	ADP/ATP carrier 2	GP
cassava4.1_009356m|PACid:17980429	cassava4.1_009356m	methionine adenosyltransferase 3	GP
cassava4.1_009357m|PACid:17993728	cassava4.1_009357m	Protein phosphatase 2C family protein	No
cassava4.1_009358m|PACid:17963891	cassava4.1_009358m	UDP-D-apiose/UDP-D-xylose synthase 2	GP
cassava4.1_009368m|PACid:17971248	cassava4.1_009368m	arginine/serine-rich 45	No
cassava4.1_009369m|PACid:17977257	cassava4.1_009369m	Zinc finger, C3HC4 type (RING finger) family protein	G
cassava4.1_009371m|PACid:17960414	cassava4.1_009371m	DNA glycosylase superfamily protein	No
cassava4.1_009374m|PACid:17980271	cassava4.1_009374m	R-protein L3 B	No
cassava4.1_009376m|PACid:17988724	cassava4.1_009376m	sedoheptulose-bisphosphatase	GP
cassava4.1_009378m|PACid:17983251	cassava4.1_009378m	Uroporphyrinogen decarboxylase	GP
cassava4.1_009382m|PACid:17991649	cassava4.1_009382m	RNI-like superfamily protein	No
cassava4.1_009389m|PACid:17980741	cassava4.1_009389m	ovate family protein 2	G
cassava4.1_009390m|PACid:17990034	cassava4.1_009390m	P-loop containing nucleoside triphosphate hydrolases superfamily protein	No
cassava4.1_009391m|PACid:17969153	cassava4.1_009391m	aminoalcoholphosphotransferase 1	No
cassava4.1_009393m|PACid:17972046	cassava4.1_009393m	alpha/beta-Hydrolases superfamily protein	G
cassava4.1_009394m|PACid:17965633	cassava4.1_009394m	nucleotide repair protein, putative	G
cassava4.1_009407m|PACid:17988431	cassava4.1_009407m	Protein kinase superfamily protein	No
cassava4.1_009408m|PACid:17988204	cassava4.1_009408m	P-loop containing nucleoside triphosphate hydrolases superfamily protein	GP
cassava4.1_009417m|PACid:17976212	cassava4.1_009417m	RNA-binding CRS1 / YhbY (CRM) domain-containing protein	G
cassava4.1_009419m|PACid:17991420	cassava4.1_009419m	8-oxoguanine-DNA glycosylase 1	G
cassava4.1_009422m|PACid:17959978	cassava4.1_009422m	GroES-like zinc-binding dehydrogenase family protein	No
cassava4.1_009425m|PACid:17968535	cassava4.1_009425m	branched-chain amino acid transaminase 2	No
cassava4.1_009431m|PACid:17987871	cassava4.1_009431m	Protein of unknown function (DUF793)	G
cassava4.1_009433m|PACid:17962076	cassava4.1_009433m	tubby like protein 3	No
cassava4.1_009435m|PACid:17963372	cassava4.1_009435m	alpha-fucosidase 1	P
cassava4.1_009436m|PACid:17963728	cassava4.1_009436m	purple acid phosphatase 29	GP
cassava4.1_009437m|PACid:17970103	cassava4.1_009437m	actin related protein 2	G
cassava4.1_009443m|PACid:17971770	cassava4.1_009443m	phosphotyrosyl phosphatase activator (PTPA) family protein	G
cassava4.1_009445m|PACid:17976838	cassava4.1_009445m	nitrilase 4	GP
cassava4.1_009448m|PACid:17971809	cassava4.1_009448m	Endoplasmic reticulum vesicle transporter protein	No
cassava4.1_009449m|PACid:17986284	cassava4.1_009449m	PATATIN-like protein 9	G

cassava4.1_009452m|PACid:17992586	cassava4.1_009452m	protein kinase C-like zinc finger protein	G
cassava4.1_009456m|PACid:17967649	cassava4.1_009456m	PYRIMIDINE B	GP
cassava4.1_009459m|PACid:17971336	cassava4.1_009459m	2-oxoglutarate (2OG) and Fe(II)-dependent oxygenase superfamily protein	No
cassava4.1_009461m|PACid:17971135	cassava4.1_009461m	GDSL-like Lipase/Acylhydrolase superfamily protein	GP
cassava4.1_009463m|PACid:17963148	cassava4.1_009463m	beta-1,4-N-acetylglucosaminyltransferase family protein	No
cassava4.1_009466m|PACid:17975453	cassava4.1_009466m	TLD-domain containing nucleolar protein	No
cassava4.1_009470m|PACid:17985019	cassava4.1_009470m	thylakoid ATP/ADP carrier	GP
cassava4.1_009472m|PACid:17976177	cassava4.1_009472m	CTC-interacting domain 11	G
cassava4.1_009477m|PACid:17977503	cassava4.1_009477m	hydroxypyruvate reductase	GP
cassava4.1_009478m|PACid:17966554	cassava4.1_009478m	Proteasome component (PCI) domain protein	GP
cassava4.1_009479m|PACid:17976084	cassava4.1_009479m	diphthamide synthesis DPH2 family protein	G
cassava4.1_009481m|PACid:17976747	cassava4.1_009481m	Protein kinase superfamily protein	No
cassava4.1_009483m|PACid:17975820	cassava4.1_009483m	Protein phosphatase 2C family protein	No
cassava4.1_009487m|PACid:17979965	cassava4.1_009487m	Tyrosyl-tRNA synthetase, class Ib, bacterial/mitochondrial	No
cassava4.1_009493m|PACid:17970061	cassava4.1_009493m	26S proteasome, regulatory subunit Rpn7;Proteasome component (PCI) domain	GP
cassava4.1_009494m|PACid:17982543	cassava4.1_009494m	NAD-dependent glycerol-3-phosphate dehydrogenase family protein	No
cassava4.1_009500m|PACid:17976055	cassava4.1_009500m	RNA-binding (RRM/RBD/RNP motifs) family protein	No
cassava4.1_009503m|PACid:17983331	cassava4.1_009503m	Cystathionine beta-synthase (CBS) protein	No
cassava4.1_009509m|PACid:17969371	cassava4.1_009509m	CLP protease proteolytic subunit 1	G
cassava4.1_009510m|PACid:17973789	cassava4.1_009510m	Protein phosphatase 2C family protein	No
cassava4.1_009511m|PACid:17976540	cassava4.1_009511m	Nucleotidylyl transferase superfamily protein	GP
cassava4.1_009513m|PACid:17967167	cassava4.1_009513m	12-oxophytodienoate reductase 2	No
cassava4.1_009514m|PACid:17977830	cassava4.1_009514m	LisH/CRA/RING-U-box domains-containing protein	No
cassava4.1_009518m|PACid:17978239	cassava4.1_009518m	Galactose oxidase/kelch repeat superfamily protein	G
cassava4.1_009522m|PACid:17960452	cassava4.1_009522m	Walls Are Thin 1	No
cassava4.1_009525m|PACid:17975229	cassava4.1_009525m	myb domain protein 105	G
cassava4.1_009530m|PACid:17963734	cassava4.1_009530m	Ca2+ activated outward rectifying K+ channel 5	G
cassava4.1_009531m|PACid:17965363	cassava4.1_009531m	Protein phosphatase 2C family protein	G
cassava4.1_009533m|PACid:17973717	cassava4.1_009533m	Pseudouridine synthase family protein	G
cassava4.1_009537m|PACid:17978036	cassava4.1_009537m	Protein phosphatase 2C family protein	G
cassava4.1_009541m|PACid:17987195	cassava4.1_009541m	gamma-glutamyl hydrolase 2	G
cassava4.1_009542m|PACid:17966006	cassava4.1_009542m	mevalonate kinase	No
cassava4.1_009543m|PACid:17991869	cassava4.1_009543m	shikimate kinase like 2	GP
cassava4.1_009544m|PACid:17983490	cassava4.1_009544m	D-cysteine desulfhydrase	GP
cassava4.1_009552m|PACid:17974139	cassava4.1_009552m	GDSL-like Lipase/Acylhydrolase superfamily protein	No
cassava4.1_009562m|PACid:17961363	cassava4.1_009562m	Protein kinase superfamily protein	No
cassava4.1_009566m|PACid:17973549	cassava4.1_009566m	NAD(P)-linked oxidoreductase superfamily protein	GP
cassava4.1_009567m|PACid:17965001	cassava4.1_009567m	Pseudouridine synthase family protein	G
cassava4.1_009571m|PACid:17965499	cassava4.1_009571m	cysteine synthase 26	GP

cassava4.1_009573m|PACid:17964591	cassava4.1_009573m	small G protein family protein / RhoGAP family protein	GP
cassava4.1_009578m|PACid:17967122	cassava4.1_009578m	Core-2/I-branching beta-1,6-N-acetylglucosaminyltransferase family protein	G
cassava4.1_009581m|PACid:17977840	cassava4.1_009581m	Protein of unknown function (DUF620)	No
cassava4.1_009584m|PACid:17960395	cassava4.1_009584m	alpha/beta-Hydrolases superfamily protein	G
cassava4.1_009587m|PACid:17971288	cassava4.1_009587m	RNA-binding KH domain-containing protein	G
cassava4.1_009589m|PACid:17989580	cassava4.1_009589m	pfkB-like carbohydrate kinase family protein	GP
cassava4.1_009590m|PACid:17990824	cassava4.1_009590m	riboflavin kinase/FMN hydrolase	G
cassava4.1_009595m|PACid:17970809	cassava4.1_009595m	Radical SAM superfamily protein	G
cassava4.1_009598m|PACid:17980877	cassava4.1_009598m	Polyketide synthase, enoylreductase family protein	GP
cassava4.1_009600m|PACid:17970686	cassava4.1_009600m	Glycosyl hydrolase superfamily protein	G
cassava4.1_009603m|PACid:17993715	cassava4.1_009603m	fatty acid desaturase 2	No
cassava4.1_009604m|PACid:17981466	cassava4.1_009604m	Transducin/WD40 repeat-like superfamily protein	No
cassava4.1_009606m|PACid:17987401	cassava4.1_009606m	ARM repeat superfamily protein	G
cassava4.1_009613m|PACid:17993565	cassava4.1_009613m	Serine carboxypeptidase S28 family protein	GP
cassava4.1_009614m|PACid:17985752	cassava4.1_009614m	Mitochondrial substrate carrier family protein	GP
cassava4.1_009623m|PACid:17989637	cassava4.1_009623m	Rad23 UV excision repair protein family	GP
cassava4.1_009626m|PACid:17977764	cassava4.1_009626m	Protein of unknown function, DUF547	G
cassava4.1_009634m|PACid:17990667	cassava4.1_009634m	NagB/RpiA/CoA transferase-like superfamily protein	GP
cassava4.1_009639m|PACid:17979300	cassava4.1_009639m	Ribosomal protein L1p/L10e family	No
cassava4.1_009642m|PACid:17979787	cassava4.1_009642m	HAL2-like	No
cassava4.1_009650m|PACid:17962576	cassava4.1_009650m	polyubiquitin 10	No
cassava4.1_009661m|PACid:17983919	cassava4.1_009661m	Protein kinase superfamily protein	G
cassava4.1_009664m|PACid:17973307	cassava4.1_009664m	Core-2/I-branching beta-1,6-N-acetylglucosaminyltransferase family protein	G
cassava4.1_009669m|PACid:17961523	cassava4.1_009669m	PLC-like phosphodiesterases superfamily protein	No
cassava4.1_009671m|PACid:17973481	cassava4.1_009671m	Glycosyl hydrolase superfamily protein	G
cassava4.1_009674m|PACid:17971527	cassava4.1_009674m	alpha/beta-Hydrolases superfamily protein	G
cassava4.1_009678m|PACid:17965520	cassava4.1_009678m	hydroxymethylbilane synthase	GP
cassava4.1_009680m|PACid:17992608	cassava4.1_009680m	PQ-loop repeat family protein / transmembrane family protein	No
cassava4.1_009689m|PACid:17991337	cassava4.1_009689m	Protein kinase superfamily protein	No
cassava4.1_009692m|PACid:17977960	cassava4.1_009692m	Calcium-binding EF hand family protein	GP
cassava4.1_009693m|PACid:17968154	cassava4.1_009693m	tetraticopeptide domain-containing thioredoxin	GP
cassava4.1_009694m|PACid:17994158	cassava4.1_009694m	SHAGGY-related protein kinase dZeta	G
cassava4.1_009697m|PACid:17993198	cassava4.1_009697m	chloroplast RNA binding	GP
cassava4.1_009701m|PACid:17964323	cassava4.1_009701m	Protein kinase superfamily protein	G
cassava4.1_009702m|PACid:17985726	cassava4.1_009702m	Pectin lyase-like superfamily protein	No
cassava4.1_009703m|PACid:17985316	cassava4.1_009703m	GroES-like zinc-binding alcohol dehydrogenase family protein	GP
cassava4.1_009705m|PACid:17989626	cassava4.1_009705m	alpha/beta-Hydrolases superfamily protein	G
cassava4.1_009707m|PACid:17980388	cassava4.1_009707m	Nucleotide-sugar transporter family protein	G
cassava4.1_009709m|PACid:17964594	cassava4.1_009709m	2-oxoglutarate (2OG) and Fe(II)-dependent oxygenase superfamily protein	G

cassava4.1_009713m|PACid:17960490	cassava4.1_009713m		GP
cassava4.1_009715m|PACid:17979831	cassava4.1_009715m	ADP/ATP carrier 3	GP
cassava4.1_009718m|PACid:17991805	cassava4.1_009718m	Protein kinase superfamily protein	No
cassava4.1_009719m|PACid:17973685	cassava4.1_009719m	ARABIDOPSIS TRITHORAX-RELATED PROTEIN 5	G
cassava4.1_009721m|PACid:17979358	cassava4.1_009721m	histone deacetylase 8	No
cassava4.1_009722m|PACid:17973090	cassava4.1_009722m	Radical SAM superfamily protein	G
cassava4.1_009723m|PACid:17987239	cassava4.1_009723m	tubby like protein 7	G
cassava4.1_009726m|PACid:17993377	cassava4.1_009726m	Protein kinase superfamily protein	G
cassava4.1_009729m|PACid:17964394	cassava4.1_009729m	GroES-like zinc-binding dehydrogenase family protein	P
cassava4.1_009730m|PACid:17979474	cassava4.1_009730m	actin-related protein C1A	No
cassava4.1_009731m|PACid:17977791	cassava4.1_009731m	lysophosphatidyl acyltransferase 5	G
cassava4.1_009735m|PACid:17978895	cassava4.1_009735m	dual specificity protein phosphatase (DsPTP1) family protein	GP
cassava4.1_009737m|PACid:17969406	cassava4.1_009737m	F-box/RNI-like superfamily protein	G
cassava4.1_009739m|PACid:17979036	cassava4.1_009739m	Haloacid dehalogenase-like hydrolase (HAD) superfamily protein	G
cassava4.1_009741m|PACid:17986722	cassava4.1_009741m	nodulin MtN21 /EamA-like transporter family protein	G
cassava4.1_009742m|PACid:17990736	cassava4.1_009742m	BREVIS RADIX-like 4	No
cassava4.1_009743m|PACid:17970341	cassava4.1_009743m	Mitochondrial substrate carrier family protein	G
cassava4.1_009744m|PACid:17976859	cassava4.1_009744m	Protein kinase superfamily protein	P
cassava4.1_009748m|PACid:17977067	cassava4.1_009748m	BTB and TAZ domain protein 4	G
cassava4.1_009755m|PACid:17977815	cassava4.1_009755m	indole-3-acetic acid inducible 9	No
cassava4.1_009756m|PACid:17972420	cassava4.1_009756m	PRP38 family protein	G
cassava4.1_009767m|PACid:17992931	cassava4.1_009767m	adenosine/AMP deaminase family protein	No
cassava4.1_009780m|PACid:17993116	cassava4.1_009780m	actin 7	No
cassava4.1_009782m|PACid:17988600	cassava4.1_009782m	actin 3	No
cassava4.1_009783m|PACid:17979927	cassava4.1_009783m	actin 1	No
cassava4.1_009786m|PACid:17969727	cassava4.1_009786m	GTP binding protein beta 1	GP
cassava4.1_009791m|PACid:17993480	cassava4.1_009791m	ATP-dependent RNA helicase, mitochondrial (SUV3)	G
cassava4.1_009793m|PACid:17984889	cassava4.1_009793m	protein arginine methyltransferase 10	G
cassava4.1_009795m|PACid:17970534	cassava4.1_009795m	Nitrilase/cyanide hydratase and apolipoprotein N-acyltransferase family protein	GP
cassava4.1_009796m|PACid:17980446	cassava4.1_009796m	isocitrate dehydrogenase V	GP
cassava4.1_009797m|PACid:17982521	cassava4.1_009797m	RNA cyclase family protein	G
cassava4.1_009799m|PACid:17979703	cassava4.1_009799m	Glycosyl hydrolase superfamily protein	GP
cassava4.1_009803m|PACid:17961431	cassava4.1_009803m	semialdehyde dehydrogenase family protein	P
cassava4.1_009804m|PACid:17971921	cassava4.1_009804m	Protein kinase superfamily protein	No
cassava4.1_009811m|PACid:17980368	cassava4.1_009811m	Glyceraldehyde-3-phosphate dehydrogenase-like family protein	GP
cassava4.1_009814m|PACid:17978376	cassava4.1_009814m	Cobalamin biosynthesis CobW-like protein	G
cassava4.1_009815m|PACid:17965664	cassava4.1_009815m	GDP-D-mannose 3\',5\'-epimerase	G
cassava4.1_009816m|PACid:17983655	cassava4.1_009816m	DNA-binding protein phosphatase 1	G
cassava4.1_009822m|PACid:17967484	cassava4.1_009822m	RNA polymerase I subunit 43	No

cassava4.1_009825m|PACid:17983501	cassava4.1_009825m	protein kinase family protein	No
cassava4.1_009827m|PACid:17980947	cassava4.1_009827m	Galactose oxidase/kelch repeat superfamily protein	No
cassava4.1_009829m|PACid:17989764	cassava4.1_009829m	alpha/beta-Hydrolases superfamily protein	G
cassava4.1_009832m|PACid:17989518	cassava4.1_009832m	Prenyltransferase family protein	No
cassava4.1_009835m|PACid:17974539	cassava4.1_009835m	Afadin/alpha-actinin-binding protein	GP
cassava4.1_009836m|PACid:17991543	cassava4.1_009836m	ATPase, F1 complex, gamma subunit protein	GP
cassava4.1_009840m|PACid:17991296	cassava4.1_009840m	vacuolar ATP synthase subunit C (VATC) / V-ATPase C subunit / vacuolar proton pump C subunit (DET3)	P
cassava4.1_009844m|PACid:17963024	cassava4.1_009844m	histone-lysine N-methyltransferase ASHH3	GP
cassava4.1_009846m|PACid:17989631	cassava4.1_009846m	formyltransferase, putative	GP
cassava4.1_009848m|PACid:17993923	cassava4.1_009848m	FMN-linked oxidoreductases superfamily protein	No
cassava4.1_009852m|PACid:17974367	cassava4.1_009852m	mercaptopyruvate sulfurtransferase 1	No
cassava4.1_009853m|PACid:17976664	cassava4.1_009853m	Protein kinase superfamily protein	No
cassava4.1_009858m|PACid:17970880	cassava4.1_009858m	Metal-dependent protein hydrolase	GP
cassava4.1_009859m|PACid:17974809	cassava4.1_009859m	zinc finger (C2H2 type) family protein	G
cassava4.1_009862m|PACid:17976478	cassava4.1_009862m	glycerol-3-phosphate acyltransferase 9	G
cassava4.1_009863m|PACid:17982104	cassava4.1_009863m	P-loop containing nucleoside triphosphate hydrolases superfamily protein	No
cassava4.1_009864m|PACid:17968740	cassava4.1_009864m	Bifunctional inhibitor/lipid-transfer protein/seed storage 2S albumin superfamily protein	G
cassava4.1_009867m|PACid:17964779	cassava4.1_009867m	stromal ascorbate peroxidase	GP
cassava4.1_009868m|PACid:17982137	cassava4.1_009868m	alpha/beta-Hydrolases superfamily protein	P
cassava4.1_009873m|PACid:17973121	cassava4.1_009873m	Calcium-dependent ARF-type GTPase activating protein family	G
cassava4.1_009883m|PACid:17982035	cassava4.1_009883m	RNA-binding (RRM/RBD/RNP motifs) family protein	GP
cassava4.1_009885m|PACid:17982651	cassava4.1_009885m	Protein phosphatase 2C family protein	No
cassava4.1_009887m|PACid:17981953	cassava4.1_009887m	chloroplastic NIFS-like cysteine desulfurase	P
cassava4.1_009888m|PACid:17962906	cassava4.1_009888m	Protein of unknown function, DUF642	No
cassava4.1_009890m|PACid:17962466	cassava4.1_009890m	Pseudouridine synthase family protein	No
cassava4.1_009892m|PACid:17980778	cassava4.1_009892m	Radical SAM superfamily protein	G
cassava4.1_009894m|PACid:17992055	cassava4.1_009894m	nodulin MtN21 /EamA-like transporter family protein	No
cassava4.1_009899m|PACid:17979766	cassava4.1_009899m	Peroxisomal membrane 22 kDa (Mpv17/PMP22) family protein	G
cassava4.1_009904m|PACid:17991177	cassava4.1_009904m	2-oxoglutarate (2OG) and Fe(II)-dependent oxygenase superfamily protein	No
cassava4.1_009906m|PACid:17989701	cassava4.1_009906m	heptahelical transmembrane protein1	G
cassava4.1_009909m|PACid:17959755	cassava4.1_009909m	gibberellin 3-oxidase 1	No
cassava4.1_009911m|PACid:17962271	cassava4.1_009911m	catalytics;transferases;[acyl-carrier-protein] S-malonyltransferases;binding	GP
cassava4.1_009915m|PACid:17970216	cassava4.1_009915m	Eukaryotic protein of unknown function (DUF914)	G
cassava4.1_009923m|PACid:17962913	cassava4.1_009923m	alpha/beta-Hydrolases superfamily protein	GP
cassava4.1_009925m|PACid:17992866	cassava4.1_009925m	chloroplast signal recognition particle component (CAO)	GP
cassava4.1_009927m|PACid:17989989	cassava4.1_009927m	phosphatase-related	GP
cassava4.1_009931m|PACid:17988668	cassava4.1_009931m	Haloacid dehalogenase-like hydrolase (HAD) superfamily protein	No
cassava4.1_009933m|PACid:17967169	cassava4.1_009933m	GDSL-like Lipase/Acylhydrolase superfamily protein	No
cassava4.1_009936m|PACid:17986787	cassava4.1_009936m	FKBP-type peptidyl-prolyl cis-trans isomerase family protein	No

cassava4.1_009941m|PACid:17969422	cassava4.1_009941m	mitogen-activated protein kinase 1	No
cassava4.1_009942m|PACid:17961123	cassava4.1_009942m	Papain family cysteine protease	GP
cassava4.1_009945m|PACid:17964603	cassava4.1_009945m		GP
cassava4.1_009949m|PACid:17985532	cassava4.1_009949m	arginine/serine-rich splicing factor 35	GP
cassava4.1_009952m|PACid:17966390	cassava4.1_009952m	isocitrate dehydrogenase III	GP
cassava4.1_009954m|PACid:17960525	cassava4.1_009954m	mitogen-activated protein kinase 1	No
cassava4.1_009955m|PACid:17994174	cassava4.1_009955m	Calcineurin-like metallo-phosphoesterase superfamily protein	G
cassava4.1_009970m|PACid:17974242	cassava4.1_009970m	alpha/beta-Hydrolases superfamily protein	No
cassava4.1_009971m|PACid:17979327	cassava4.1_009971m	NAD(P)-linked oxidoreductase superfamily protein	GP
cassava4.1_009973m|PACid:17963135	cassava4.1_009973m	Core-2/I-branching beta-1,6-N-acetylglucosaminyltransferase family protein	G
cassava4.1_009974m|PACid:17961135	cassava4.1_009974m	SH3 domain-containing protein	No
cassava4.1_009975m|PACid:17988614	cassava4.1_009975m	TLD-domain containing nucleolar protein	G
cassava4.1_009980m|PACid:17988969	cassava4.1_009980m	Tetratricopeptide repeat (TPR)-like superfamily protein	GP
cassava4.1_009985m|PACid:17986655	cassava4.1_009985m	ornithine carbamoyltransferase	P
cassava4.1_009986m|PACid:17985871	cassava4.1_009986m	ATP-dependent caseinolytic (Clp) protease/crotonase family protein	G
cassava4.1_009988m|PACid:17979114	cassava4.1_009988m	SIGNAL PEPTIDE PEPTIDASE-LIKE 1	G
cassava4.1_009991m|PACid:17976403	cassava4.1_009991m	Oxidoreductase family protein	GP
cassava4.1_009992m|PACid:17978487	cassava4.1_009992m	GDSL-like Lipase/Acylhydrolase superfamily protein	No
cassava4.1_009993m|PACid:17964985	cassava4.1_009993m	phosphate transporter 3;1	No
cassava4.1_009996m|PACid:17976021	cassava4.1_009996m	myb domain protein 26	No
cassava4.1_009997m|PACid:17980161	cassava4.1_009997m	diaminopimelate epimerase family protein	GP
cassava4.1_009999m|PACid:17980964	cassava4.1_009999m	Protein kinase superfamily protein	G
cassava4.1_010000m|PACid:17991103	cassava4.1_010000m	NAD+ transporter 2	G
cassava4.1_010002m|PACid:17966525	cassava4.1_010002m	alpha/beta-Hydrolases superfamily protein	G
cassava4.1_010005m|PACid:17975589	cassava4.1_010005m	MAP kinase 4	G
cassava4.1_010012m|PACid:17985257	cassava4.1_010012m	high-affinity nickel-transport family protein	G
cassava4.1_010013m|PACid:17983306	cassava4.1_010013m	Purple acid phosphatases superfamily protein	No
cassava4.1_010014m|PACid:17960722	cassava4.1_010014m	Pectin lyase-like superfamily protein	GP
cassava4.1_010018m|PACid:17968578	cassava4.1_010018m	FAD/NAD(P)-binding oxidoreductase family protein	GP
cassava4.1_010020m|PACid:17975868	cassava4.1_010020m	geranylgeranyl pyrophosphate synthase 1	GP
cassava4.1_010025m|PACid:17963541	cassava4.1_010025m	pfkB-like carbohydrate kinase family protein	No
cassava4.1_010027m|PACid:17983653	cassava4.1_010027m	Protein of unknown function, DUF642	No
cassava4.1_010034m|PACid:17966577	cassava4.1_010034m	galacturonosyltransferase-like 10	G
cassava4.1_010035m|PACid:17987815	cassava4.1_010035m	Transducin/WD40 repeat-like superfamily protein	G
cassava4.1_010036m|PACid:17988638	cassava4.1_010036m	ubiquitin-specific protease 3	No
cassava4.1_010037m|PACid:17993583	cassava4.1_010037m	Protein of unknown function, DUF642	GP
cassava4.1_010038m|PACid:17990993	cassava4.1_010038m	ubiquitin-specific protease 3	G
cassava4.1_010039m|PACid:17985100	cassava4.1_010039m	FAD/NAD(P)-binding oxidoreductase family protein	G
cassava4.1_010043m|PACid:17971785	cassava4.1_010043m	Haloacid dehalogenase-like hydrolase (HAD) superfamily protein	No

cassava4.1_010044m|PACid:17968956	cassava4.1_010044m	methionine aminopeptidase 1B	GP
cassava4.1_010045m|PACid:17993258	cassava4.1_010045m	Protein phosphatase 2C family protein	G
cassava4.1_010050m|PACid:17960745	cassava4.1_010050m	spermidine synthase 3	No
cassava4.1_010051m|PACid:17993042	cassava4.1_010051m	holocarboxylase synthase 1	G
cassava4.1_010056m|PACid:17975387	cassava4.1_010056m	reversibly glycosylated polypeptide 3	GP
cassava4.1_010059m|PACid:17979346	cassava4.1_010059m	NADPH-dependent thioredoxin reductase A	No
cassava4.1_010063m|PACid:17964356	cassava4.1_010063m	deoxyhypusine synthase	GP
cassava4.1_010067m|PACid:17980371	cassava4.1_010067m	pyruvate dehydrogenase kinase	No
cassava4.1_010072m|PACid:17984207	cassava4.1_010072m	Protein kinase superfamily protein	G
cassava4.1_010076m|PACid:17984879	cassava4.1_010076m	Actin-like ATPase superfamily protein	No
cassava4.1_010077m|PACid:17985700	cassava4.1_010077m	Protein of unknown function, DUF642	No
cassava4.1_010081m|PACid:17991295	cassava4.1_010081m	senescence-related gene 3	No
cassava4.1_010082m|PACid:17982673	cassava4.1_010082m	GDSL-like Lipase/Acylhydrolase superfamily protein	No
cassava4.1_010084m|PACid:17960990	cassava4.1_010084m	KNOTTED-like from Arabidopsis thaliana	G
cassava4.1_010087m|PACid:17961777	cassava4.1_010087m	Protein phosphatase 2C family protein	No
cassava4.1_010088m|PACid:17974673	cassava4.1_010088m	mRNA capping enzyme family protein	G
cassava4.1_010090m|PACid:17986920	cassava4.1_010090m	D111/G-patch domain-containing protein	No
cassava4.1_010092m|PACid:17977150	cassava4.1_010092m	Protein kinase superfamily protein	No
cassava4.1_010113m|PACid:17968026	cassava4.1_010113m	Glycosyl hydrolase family protein with chitinase insertion domain	P
cassava4.1_010114m|PACid:17972014	cassava4.1_010114m	Uncharacterised conserved protein (UCP030210)	GP
cassava4.1_010115m|PACid:17968296	cassava4.1_010115m	Mitochondrial substrate carrier family protein	No
cassava4.1_010116m|PACid:17993241	cassava4.1_010116m	Transketolase family protein	GP
cassava4.1_010118m|PACid:17966061	cassava4.1_010118m	amidase 1	No
cassava4.1_010124m|PACid:17982135	cassava4.1_010124m	DWD (DDB1-binding WD40 protein) hypersensitive to ABA 1	No
cassava4.1_010126m|PACid:17970384	cassava4.1_010126m	Aldolase-type TIM barrel family protein	G
cassava4.1_010130m|PACid:17986669	cassava4.1_010130m	pfkB-like carbohydrate kinase family protein	No
cassava4.1_010135m|PACid:17984485	cassava4.1_010135m	CYCLIN A3;4	No
cassava4.1_010140m|PACid:17963994	cassava4.1_010140m	cinnamyl alcohol dehydrogenase 6	P
cassava4.1_010141m|PACid:17972057	cassava4.1_010141m	GDSL-like Lipase/Acylhydrolase superfamily protein	G
cassava4.1_010145m|PACid:17972283	cassava4.1_010145m	Aldolase-type TIM barrel family protein	No
cassava4.1_010146m|PACid:17970121	cassava4.1_010146m	Ribosomal RNA processing Brix domain protein	No
cassava4.1_010158m|PACid:17990690	cassava4.1_010158m	nucleosome assembly protein 1;2	No
cassava4.1_010159m|PACid:17990025	cassava4.1_010159m	NAD(P)-linked oxidoreductase superfamily protein	No
cassava4.1_010164m|PACid:17960545	cassava4.1_010164m	Papain family cysteine protease	GP
cassava4.1_010165m|PACid:17978804	cassava4.1_010165m	Hyaluronan / mRNA binding family	GP
cassava4.1_010166m|PACid:17966227	cassava4.1_010166m	RNA recognition motif (RRM)-containing protein	G
cassava4.1_010172m|PACid:17973886	cassava4.1_010172m	nodulin MtN21 /EamA-like transporter family protein	No
cassava4.1_010173m|PACid:17963025	cassava4.1_010173m	NAD(P)-binding Rossmann-fold superfamily protein	G
cassava4.1_010176m|PACid:17978871	cassava4.1_010176m	nudix hydrolase homolog 8	G

cassava4.1_010180m|PACid:17979980	cassava4.1_010180m	homoserine kinase	GP
cassava4.1_010182m|PACid:17979487	cassava4.1_010182m	dihydrodipicolinate synthase	GP
cassava4.1_010183m|PACid:17987477	cassava4.1_010183m	reversibly glycosylated polypeptide 1	No
cassava4.1_010184m|PACid:17978171	cassava4.1_010184m	NAD(P)H dehydrogenase C1	GP
cassava4.1_010185m|PACid:17982691	cassava4.1_010185m	NAD(P)-binding Rossmann-fold superfamily protein	GP
cassava4.1_010187m|PACid:17974734	cassava4.1_010187m	O-methyltransferase 1	P
cassava4.1_010192m|PACid:17976432	cassava4.1_010192m	alpha/beta-Hydrolases superfamily protein	G
cassava4.1_010198m|PACid:17977048	cassava4.1_010198m	Rad23 UV excision repair protein family	GP
cassava4.1_010199m|PACid:17963150	cassava4.1_010199m	RNA-binding (RRM/RBD/RNP motifs) family protein	G
cassava4.1_010200m|PACid:17972357	cassava4.1_010200m		GP
cassava4.1_010205m|PACid:17971962	cassava4.1_010205m	branched-chain alpha-keto acid decarboxylase E1 beta subunit	GP
cassava4.1_010206m|PACid:17990691	cassava4.1_010206m	nucleosome assembly protein 1;2	No
cassava4.1_010209m|PACid:17982808	cassava4.1_010209m	NAD(P)-binding Rossmann-fold superfamily protein	G
cassava4.1_010212m|PACid:17966443	cassava4.1_010212m	flavanone 3-hydroxylase	GP
cassava4.1_010218m|PACid:17993936	cassava4.1_010218m	Protein of unknown function, DUF642	No
cassava4.1_010219m|PACid:17991685	cassava4.1_010219m	mitogen-activated protein kinase 3	GP
cassava4.1_010222m|PACid:17961896	cassava4.1_010222m	2-oxoglutarate (2OG) and Fe(II)-dependent oxygenase superfamily protein	No
cassava4.1_010226m|PACid:17986791	cassava4.1_010226m	urophorphyrin methylase 1	G
cassava4.1_010228m|PACid:17968264	cassava4.1_010228m	2-oxoglutarate (2OG) and Fe(II)-dependent oxygenase superfamily protein	No
cassava4.1_010230m|PACid:17973027	cassava4.1_010230m	Amino acid dehydrogenase family protein	GP
cassava4.1_010231m|PACid:17961951	cassava4.1_010231m	2-oxoglutarate (2OG) and Fe(II)-dependent oxygenase superfamily protein	G
cassava4.1_010235m|PACid:17975639	cassava4.1_010235m	Plant protein 1589 of unknown function	No
cassava4.1_010236m|PACid:17987666	cassava4.1_010236m	acyl-CoA binding protein 2	GP
cassava4.1_010240m|PACid:17978747	cassava4.1_010240m	Pectin lyase-like superfamily protein	No
cassava4.1_010244m|PACid:17985947	cassava4.1_010244m		GP
cassava4.1_010257m|PACid:17961192	cassava4.1_010257m	Leucine-rich repeat (LRR) family protein	P
cassava4.1_010258m|PACid:17972977	cassava4.1_010258m	UDP-Glycosyltransferase superfamily protein	GP
cassava4.1_010261m|PACid:17969145	cassava4.1_010261m	Mitochondrial substrate carrier family protein	No
cassava4.1_010262m|PACid:17970440	cassava4.1_010262m	Glycosyl hydrolase superfamily protein	No
cassava4.1_010265m|PACid:17985585	cassava4.1_010265m	GroES-like zinc-binding alcohol dehydrogenase family protein	GP
cassava4.1_010266m|PACid:17959922	cassava4.1_010266m	COP9-signalosome 5B	GP
cassava4.1_010269m|PACid:17967000	cassava4.1_010269m	alpha/beta-Hydrolases superfamily protein	G
cassava4.1_010270m|PACid:17983165	cassava4.1_010270m	2-oxoglutarate (2OG) and Fe(II)-dependent oxygenase superfamily protein	No
cassava4.1_010275m|PACid:17992457	cassava4.1_010275m	Aldolase-type TIM barrel family protein	GP
cassava4.1_010278m|PACid:17962377	cassava4.1_010278m	BTB and TAZ domain protein 3	No
cassava4.1_010282m|PACid:17993255	cassava4.1_010282m	GDSL-like Lipase/Acylhydrolase superfamily protein	No
cassava4.1_010284m|PACid:17990350	cassava4.1_010284m	galacturonosyltransferase-like 7	No
cassava4.1_010285m|PACid:17966901	cassava4.1_010285m	origin recognition complex second largest subunit 2	G
cassava4.1_010290m|PACid:17983184	cassava4.1_010290m	Paxneb protein-related	G

cassava4.1_010291m|PACid:17989789	cassava4.1_010291m	2-oxoglutarate (2OG) and Fe(II)-dependent oxygenase superfamily protein	No
cassava4.1_010293m|PACid:17960169	cassava4.1_010293m	Haloacid dehalogenase-like hydrolase (HAD) superfamily protein	No
cassava4.1_010297m|PACid:17964640	cassava4.1_010297m	Protein kinase superfamily protein	GP
cassava4.1_010301m|PACid:17974767	cassava4.1_010301m	TRICHOME BIREFRINGENCE-LIKE 38	G
cassava4.1_010307m|PACid:17986793	cassava4.1_010307m	Leucine-rich repeat (LRR) family protein	GP
cassava4.1_010312m|PACid:17988846	cassava4.1_010312m	purine permease 11	G
cassava4.1_010313m|PACid:17967170	cassava4.1_010313m	GDSL-like Lipase/Acylhydrolase superfamily protein	No
cassava4.1_010314m|PACid:17986115	cassava4.1_010314m	Basic-leucine zipper (bZIP) transcription factor family protein	No
cassava4.1_010315m|PACid:17981999	cassava4.1_010315m	cell division control 2	G
cassava4.1_010316m|PACid:17990498	cassava4.1_010316m	cinnamyl alcohol dehydrogenase 9	No
cassava4.1_010320m|PACid:17990502	cassava4.1_010320m	elicitor-activated gene 3-2	No
cassava4.1_010324m|PACid:17966380	cassava4.1_010324m	ferredoxin-NADP(+)-oxidoreductase 1	P
cassava4.1_010325m|PACid:17976196	cassava4.1_010325m	AAA-type ATPase family protein	No
cassava4.1_010329m|PACid:17984137	cassava4.1_010329m	GDSL-like Lipase/Acylhydrolase superfamily protein	GP
cassava4.1_010336m|PACid:17983109	cassava4.1_010336m	Galactosyltransferase family protein	No
cassava4.1_010337m|PACid:17979237	cassava4.1_010337m	autophagy protein Apg5 family	G
cassava4.1_010338m|PACid:17989295	cassava4.1_010338m	DTW domain-containing protein	No
cassava4.1_010342m|PACid:17990489	cassava4.1_010342m	elicitor-activated gene 3-1	P
cassava4.1_010343m|PACid:17987770	cassava4.1_010343m	senescence-related gene 1	GP
cassava4.1_010347m|PACid:17963773	cassava4.1_010347m	Protein kinase superfamily protein	No
cassava4.1_010349m|PACid:17991635	cassava4.1_010349m	translation elongation factor Ts (EF-Ts), putative	GP
cassava4.1_010351m|PACid:17976221	cassava4.1_010351m	signal recognition particle receptor protein, chloroplast (FTSY)	GP
cassava4.1_010353m|PACid:17970066	cassava4.1_010353m	sucrose nonfermenting 1(SNF1)-related protein kinase 2.3	GP
cassava4.1_010356m|PACid:17984932	cassava4.1_010356m	Heat shock protein DnaJ, N-terminal with domain of unknown function (DUF1977)	No
cassava4.1_010357m|PACid:17992548	cassava4.1_010357m	2-oxoglutarate (2OG) and Fe(II)-dependent oxygenase superfamily protein	G
cassava4.1_010358m|PACid:17977729	cassava4.1_010358m	Protein kinase superfamily protein	GP
cassava4.1_010360m|PACid:17964641	cassava4.1_010360m	Protein kinase superfamily protein	No
cassava4.1_010361m|PACid:17964163	cassava4.1_010361m	DNA glycosylase superfamily protein	G
cassava4.1_010370m|PACid:17977705	cassava4.1_010370m	peptidyl-prolyl cis-trans isomerase / cyclophilin-40 (CYP40) / rotamase	GP
cassava4.1_010372m|PACid:17960039	cassava4.1_010372m	NagB/RpiA/CoA transferase-like superfamily protein	No
cassava4.1_010375m|PACid:17974341	cassava4.1_010375m	zinc finger (CCCH-type/C3HC4-type RING finger) family protein	G
cassava4.1_010383m|PACid:17985855	cassava4.1_010383m	Protein phosphatase 2C family protein	No
cassava4.1_010384m|PACid:17987428	cassava4.1_010384m	S-adenosylmethionine decarboxylase	G
cassava4.1_010388m|PACid:17988979	cassava4.1_010388m	Glucose-1-phosphate adenylyltransferase family protein	GP
cassava4.1_010389m|PACid:17960661	cassava4.1_010389m	Glucose-1-phosphate adenylyltransferase family protein	G
cassava4.1_010393m|PACid:17982023	cassava4.1_010393m		G
cassava4.1_010399m|PACid:17993929	cassava4.1_010399m	actin-related protein 7	P
cassava4.1_010405m|PACid:17978276	cassava4.1_010405m	Minichromosome maintenance (MCM2/3/5) family protein	No
cassava4.1_010412m|PACid:17960515	cassava4.1_010412m	sterol methyltransferase 2	G

cassava4.1_010413m|PACid:17966802	cassava4.1_010413m	NAD(P)-binding Rossmann-fold superfamily protein	No
cassava4.1_010416m|PACid:17974853	cassava4.1_010416m	2-phosphoglycolate phosphatase 1	G
cassava4.1_010419m|PACid:17985712	cassava4.1_010419m	Glyoxalase/Bleomycin resistance protein/Dioxygenase superfamily protein	GP
cassava4.1_010421m|PACid:17988498	cassava4.1_010421m	tonoplast dicarboxylate transporter	G
cassava4.1_010422m|PACid:17988121	cassava4.1_010422m	Pseudouridine synthase family protein	No
cassava4.1_010424m|PACid:17963268	cassava4.1_010424m	P-loop containing nucleoside triphosphate hydrolases superfamily protein	GP
cassava4.1_010426m|PACid:17985202	cassava4.1_010426m	metacaspase 1	G
cassava4.1_010431m|PACid:17990109	cassava4.1_010431m	sterol methyltransferase 2	No
cassava4.1_010437m|PACid:17988131	cassava4.1_010437m	Oxidoreductase family protein	GP
cassava4.1_010444m|PACid:17991550	cassava4.1_010444m	KNOX/ELK homeobox transcription factor	No
cassava4.1_010455m|PACid:17987664	cassava4.1_010455m	polyprenyltransferase 1	No
cassava4.1_010458m|PACid:17982661	cassava4.1_010458m	F-box family protein	No
cassava4.1_010463m|PACid:17990331	cassava4.1_010463m	thioredoxin family protein	GP
cassava4.1_010468m|PACid:17965130	cassava4.1_010468m	Zinc finger, C3HC4 type (RING finger) family protein	No
cassava4.1_010470m|PACid:17973710	cassava4.1_010470m	Ypt/Rab-GAP domain of gyp1p superfamily protein	No
cassava4.1_010472m|PACid:17965689	cassava4.1_010472m	Amino acid dehydrogenase family protein	GP
cassava4.1_010486m|PACid:17994014	cassava4.1_010486m	Clathrin adaptor complexes medium subunit family protein	G
cassava4.1_010488m|PACid:17987596	cassava4.1_010488m	plant adhesion molecule 1	No
cassava4.1_010490m|PACid:17984517	cassava4.1_010490m	serine carboxypeptidase-like 42	P
cassava4.1_010493m|PACid:17982615	cassava4.1_010493m	RNA-binding CRS1 / YhbY (CRM) domain protein	G
cassava4.1_010496m|PACid:17960816	cassava4.1_010496m	SKP1-like 21	G
cassava4.1_010502m|PACid:17961042	cassava4.1_010502m	Aldolase superfamily protein	P
cassava4.1_010503m|PACid:17965790	cassava4.1_010503m	sequence-specific DNA binding transcription factors;DNA binding;DNA binding	G
cassava4.1_010509m|PACid:17974511	cassava4.1_010509m	Aldolase superfamily protein	GP
cassava4.1_010513m|PACid:17980965	cassava4.1_010513m	RmlC-like cupins superfamily protein	GP
cassava4.1_010514m|PACid:17986002	cassava4.1_010514m	myo-inositol monophosphatase like 1	P
cassava4.1_010515m|PACid:17973450	cassava4.1_010515m	recA DNA recombination family protein	G
cassava4.1_010517m|PACid:17985776	cassava4.1_010517m	DNA methyltransferase-2	G
cassava4.1_010519m|PACid:17989326	cassava4.1_010519m	threonine aldolase 1	No
cassava4.1_010525m|PACid:17978717	cassava4.1_010525m	SNF1-related protein kinase 2.10	No
cassava4.1_010528m|PACid:17974117	cassava4.1_010528m	NAC domain containing protein 100	No
cassava4.1_010531m|PACid:17983495	cassava4.1_010531m	glutamate decarboxylase	G
cassava4.1_010538m|PACid:17971453	cassava4.1_010538m	alpha/beta-Hydrolases superfamily protein	No
cassava4.1_010540m|PACid:17964694	cassava4.1_010540m	Cysteine proteinases superfamily protein	G
cassava4.1_010542m|PACid:17973602	cassava4.1_010542m	cinnamyl alcohol dehydrogenase 9	No
cassava4.1_010551m|PACid:17971296	cassava4.1_010551m	translocation protein-related	GP
cassava4.1_010554m|PACid:17970837	cassava4.1_010554m	RNA-binding (RRM/RBD/RNP motifs) family protein	No
cassava4.1_010558m|PACid:17982751	cassava4.1_010558m	RING/U-box superfamily protein	No
cassava4.1_010561m|PACid:17967257	cassava4.1_010561m	Aldolase superfamily protein	GP

cassava4.1_010563m|PACid:17982047	cassava4.1_010563m	repressor of lrx1	G
cassava4.1_010565m|PACid:17967275	cassava4.1_010565m	RING/U-box superfamily protein	No
cassava4.1_010568m|PACid:17972791	cassava4.1_010568m	nodulin MtN21 /EamA-like transporter family protein	No
cassava4.1_010572m|PACid:17972435	cassava4.1_010572m	GDSL-like Lipase/Acylhydrolase superfamily protein	No
cassava4.1_010574m|PACid:17992009	cassava4.1_010574m	esterase/lipase/thioesterase family protein	GP
cassava4.1_010578m|PACid:17990635	cassava4.1_010578m	MAP kinase kinase 2	GP
cassava4.1_010579m|PACid:17987444	cassava4.1_010579m		G
cassava4.1_010580m|PACid:17974031	cassava4.1_010580m	BTB and TAZ domain protein 1	No
cassava4.1_010581m|PACid:17979542	cassava4.1_010581m	glutamine synthase clone R1	GP
cassava4.1_010582m|PACid:17965098	cassava4.1_010582m	CCAAT-binding factor	G
cassava4.1_010585m|PACid:17977662	cassava4.1_010585m	peroxisomal NAD-malate dehydrogenase 1	G
cassava4.1_010588m|PACid:17964530	cassava4.1_010588m	N-terminal nucleophile aminohydrolases (Ntn hydrolases) superfamily protein	G
cassava4.1_010591m|PACid:17980296	cassava4.1_010591m		No
cassava4.1_010592m|PACid:17985656	cassava4.1_010592m	MAP kinase kinase 5	No
cassava4.1_010597m|PACid:17964465	cassava4.1_010597m	glutamine synthase clone R1	P
cassava4.1_010608m|PACid:17975776	cassava4.1_010608m	Phosphoenolpyruvate carboxylase family protein	No
cassava4.1_010609m|PACid:17986141	cassava4.1_010609m	peroxisomal NAD-malate dehydrogenase 1	P
cassava4.1_010610m|PACid:17977566	cassava4.1_010610m	myb-like HTH transcriptional regulator family protein	G
cassava4.1_010614m|PACid:17969411	cassava4.1_010614m	sequence-specific DNA binding transcription factors	G
cassava4.1_010615m|PACid:17990770	cassava4.1_010615m	Cysteine proteinases superfamily protein	GP
cassava4.1_010617m|PACid:17990063	cassava4.1_010617m	senescence-related gene 1	No
cassava4.1_010620m|PACid:17987783	cassava4.1_010620m	thiazole biosynthetic enzyme, chloroplast (ARA6) (THI1) (THI4)	GP
cassava4.1_010626m|PACid:17990456	cassava4.1_010626m	Calcium-binding EF-hand family protein	G
cassava4.1_010633m|PACid:17975611	cassava4.1_010633m	Nucleotide-diphospho-sugar transferase family protein	G
cassava4.1_010643m|PACid:17985034	cassava4.1_010643m	N-acetyl-l-glutamate kinase	GP
cassava4.1_010644m|PACid:17969099	cassava4.1_010644m	galacturonosyltransferase-like 3	No
cassava4.1_010650m|PACid:17970570	cassava4.1_010650m	S-adenosyl-L-methionine-dependent methyltransferases superfamily protein	G
cassava4.1_010651m|PACid:17966943	cassava4.1_010651m	F-box protein 2	G
cassava4.1_010652m|PACid:17986529	cassava4.1_010652m	cinnamyl-alcohol dehydrogenase	No
cassava4.1_010653m|PACid:17987376	cassava4.1_010653m	O-methyltransferase family protein	GP
cassava4.1_010657m|PACid:17975174	cassava4.1_010657m	ATPase family associated with various cellular activities (AAA)	GP
cassava4.1_010661m|PACid:17961364	cassava4.1_010661m	Zinc finger C-x8-C-x5-C-x3-H type family protein	GP
cassava4.1_010662m|PACid:17978844	cassava4.1_010662m	pyrimidin 4	No
cassava4.1_010664m|PACid:17989784	cassava4.1_010664m	Mitochondrial substrate carrier family protein	G
cassava4.1_010669m|PACid:17987936	cassava4.1_010669m	XB3 ortholog 2 in Arabidopsis thaliana	No
cassava4.1_010677m|PACid:17960489	cassava4.1_010677m	Nucleotide-diphospho-sugar transferases superfamily protein	G
cassava4.1_010682m|PACid:17978568	cassava4.1_010682m	adenine nucleotide transporter 1	No
cassava4.1_010683m|PACid:17963492	cassava4.1_010683m	HSP70-interacting protein 1	No
cassava4.1_010684m|PACid:17983760	cassava4.1_010684m	Protein kinase superfamily protein	GP

cassava4.1_010685m|PACid:17960424	cassava4.1_010685m	dihydroflavonol 4-reductase	G
cassava4.1_010697m|PACid:17987726	cassava4.1_010697m	electron transfer flavoprotein alpha	P
cassava4.1_010701m|PACid:17961022	cassava4.1_010701m	GDSL-like Lipase/Acylhydrolase family protein	No
cassava4.1_010724m|PACid:17967934	cassava4.1_010724m	Ribosomal protein L1p/L10e family	No
cassava4.1_010725m|PACid:17975244	cassava4.1_010725m	Uncharacterized protein family (UPF0016)	GP
cassava4.1_010728m|PACid:17972066	cassava4.1_010728m	SH3 domain-containing protein	G
cassava4.1_010734m|PACid:17973524	cassava4.1_010734m	Nucleotide-diphospho-sugar transferases superfamily protein	No
cassava4.1_010741m|PACid:17969668	cassava4.1_010741m	NAD(P)-binding Rossmann-fold superfamily protein	G
cassava4.1_010742m|PACid:17988621	cassava4.1_010742m	C-CAP/cofactor C-like domain-containing protein	No
cassava4.1_010746m|PACid:17984374	cassava4.1_010746m	Protein kinase superfamily protein	GP
cassava4.1_010751m|PACid:17965404	cassava4.1_010751m	RING/U-box superfamily protein	G
cassava4.1_010752m|PACid:17961825	cassava4.1_010752m	Surfeit locus protein 6	G
cassava4.1_010753m|PACid:17975769	cassava4.1_010753m		G
cassava4.1_010756m|PACid:17969320	cassava4.1_010756m	GPCR-type G protein 2	No
cassava4.1_010757m|PACid:17966252	cassava4.1_010757m	fructose-bisphosphate aldolase 2	No
cassava4.1_010769m|PACid:17968618	cassava4.1_010769m	Transcription factor DP	No
cassava4.1_010774m|PACid:17965025	cassava4.1_010774m	HCP-like superfamily protein with MYND-type zinc finger	No
cassava4.1_010775m|PACid:17984468	cassava4.1_010775m	ARM repeat superfamily protein	G
cassava4.1_010777m|PACid:17977809	cassava4.1_010777m	nodulin MtN21 /EamA-like transporter family protein	G
cassava4.1_010781m|PACid:17965287	cassava4.1_010781m	3\'-5\' exonuclease domain-containing protein / KH domain-containing protein	No
cassava4.1_010784m|PACid:17973521	cassava4.1_010784m	GDSL-like Lipase/Acylhydrolase superfamily protein	G
cassava4.1_010785m|PACid:17979893	cassava4.1_010785m	C2H2-type zinc finger family protein	G
cassava4.1_010790m|PACid:17987358	cassava4.1_010790m	Peroxisomal membrane 22 kDa (Mpv17/PMP22) family protein	No
cassava4.1_010791m|PACid:17959969	cassava4.1_010791m	RNI-like superfamily protein	GP
cassava4.1_010795m|PACid:17971167	cassava4.1_010795m	Protein kinase superfamily protein	G
cassava4.1_010797m|PACid:17985554	cassava4.1_010797m	Pentatricopeptide repeat (PPR) superfamily protein	G
cassava4.1_010798m|PACid:17961847	cassava4.1_010798m	Protein kinase superfamily protein	No
cassava4.1_010800m|PACid:17964194	cassava4.1_010800m	6-phosphogluconate dehydrogenase family protein	P
cassava4.1_010801m|PACid:17966321	cassava4.1_010801m	serine acetyltransferase 3;2	G
cassava4.1_010805m|PACid:17979742	cassava4.1_010805m	ATPase, V0/A0 complex, subunit C/D	P
cassava4.1_010809m|PACid:17966695	cassava4.1_010809m	leucoanthocyanidin dioxygenase	GP
cassava4.1_010813m|PACid:17972019	cassava4.1_010813m	ankyrin repeat-containing 2B	No
cassava4.1_010822m|PACid:17991048	cassava4.1_010822m	alternative oxidase 2	GP
cassava4.1_010823m|PACid:17973879	cassava4.1_010823m		G
cassava4.1_010828m|PACid:17965284	cassava4.1_010828m	RAS associated with diabetes protein 51	No
cassava4.1_010831m|PACid:17966322	cassava4.1_010831m	serine acetyltransferase 3;2	No
cassava4.1_010837m|PACid:17961225	cassava4.1_010837m	Endoplasmic reticulum vesicle transporter protein	No
cassava4.1_010847m|PACid:17962991	cassava4.1_010847m	Transducin/WD40 repeat-like superfamily protein	GP
cassava4.1_010849m|PACid:17985771	cassava4.1_010849m	Phosphorylase superfamily protein	GP

cassava4.1_010850m|PACid:17962895	cassava4.1_010850m	fatty acid desaturase 5	No
cassava4.1_010852m|PACid:17982369	cassava4.1_010852m	RNA-binding (RRM/RBD/RNP motifs) family protein	GP
cassava4.1_010854m|PACid:17987668	cassava4.1_010854m	thylakoid-associated phosphatase 38	GP
cassava4.1_010857m|PACid:17972654	cassava4.1_010857m	LEM3 (ligand-effect modulator 3) family protein / CDC50 family protein	G
cassava4.1_010863m|PACid:17977552	cassava4.1_010863m	Zinc-binding dehydrogenase family protein	GP
cassava4.1_010864m|PACid:17975287	cassava4.1_010864m	Transducin/WD40 repeat-like superfamily protein	GP
cassava4.1_010865m|PACid:17979003	cassava4.1_010865m	Transducin/WD40 repeat-like superfamily protein	No
cassava4.1_010867m|PACid:17970765	cassava4.1_010867m	methionine aminopeptidase 1D	GP
cassava4.1_010874m|PACid:17983846	cassava4.1_010874m	NAD(P)-binding Rossmann-fold superfamily protein	No
cassava4.1_010879m|PACid:17983666	cassava4.1_010879m	Nucleotide-sugar transporter family protein	G
cassava4.1_010884m|PACid:17970365	cassava4.1_010884m	LEM3 (ligand-effect modulator 3) family protein / CDC50 family protein	GP
cassava4.1_010888m|PACid:17961076	cassava4.1_010888m	Transducin/WD40 repeat-like superfamily protein	No
cassava4.1_010889m|PACid:17961180	cassava4.1_010889m	NAD(P)-binding Rossmann-fold superfamily protein	GP
cassava4.1_010894m|PACid:17960805	cassava4.1_010894m	Protein phosphatase 2C family protein	G
cassava4.1_010896m|PACid:17975363	cassava4.1_010896m	syntaxin of plants 32	No
cassava4.1_010906m|PACid:17983848	cassava4.1_010906m	nitrilase 4	GP
cassava4.1_010907m|PACid:17993161	cassava4.1_010907m	Clathrin adaptor complexes medium subunit family protein	No
cassava4.1_010908m|PACid:17968236	cassava4.1_010908m	UDP-D-glucose/UDP-D-galactose 4-epimerase 1	P
cassava4.1_010913m|PACid:17986715	cassava4.1_010913m	serine/threonine protein kinase 3	GP
cassava4.1_010916m|PACid:17965196	cassava4.1_010916m	nucleotide binding protein 35	GP
cassava4.1_010924m|PACid:17981384	cassava4.1_010924m	Aha1 domain-containing protein	GP
cassava4.1_010925m|PACid:17971808	cassava4.1_010925m	Sec14p-like phosphatidylinositol transfer family protein	GP
cassava4.1_010927m|PACid:17966150	cassava4.1_010927m	GDSL-like Lipase/Acylhydrolase superfamily protein	No
cassava4.1_010934m|PACid:17989604	cassava4.1_010934m	alpha/beta-Hydrolases superfamily protein	G
cassava4.1_010940m|PACid:17987572	cassava4.1_010940m		GP
cassava4.1_010943m|PACid:17983089	cassava4.1_010943m	N-MYC downregulated-like 2	G
cassava4.1_010944m|PACid:17982713	cassava4.1_010944m	Protein of unknown function (DUF803)	G
cassava4.1_010946m|PACid:17980976	cassava4.1_010946m	Zinc-binding dehydrogenase family protein	P
cassava4.1_010947m|PACid:17967110	cassava4.1_010947m	quinolinate phoshoribosyltransferase	No
cassava4.1_010955m|PACid:17966115	cassava4.1_010955m	UTP:galactose-1-phosphate uridylyltransferases;ribose-5-phosphate adenylyltransferases	G
cassava4.1_010956m|PACid:17974128	cassava4.1_010956m	Transducin/WD40 repeat-like superfamily protein	No
cassava4.1_010959m|PACid:17965517	cassava4.1_010959m	Succinyl-CoA ligase, alpha subunit	GP
cassava4.1_010963m|PACid:17976888	cassava4.1_010963m	Ribosomal RNA adenine dimethylase family protein	G
cassava4.1_010964m|PACid:17986328	cassava4.1_010964m	Nuclear pore localisation protein NPL4	GP
cassava4.1_010966m|PACid:17973105	cassava4.1_010966m	phytochrome-associated protein 1	No
cassava4.1_010967m|PACid:17988507	cassava4.1_010967m	Phosphoglycerate mutase family protein	G
cassava4.1_010968m|PACid:17979261	cassava4.1_010968m	vascular related NAC-domain protein 1	No
cassava4.1_010970m|PACid:17985513	cassava4.1_010970m	pfkB-like carbohydrate kinase family protein	GP
cassava4.1_010972m|PACid:17965139	cassava4.1_010972m	UDP-XYL synthase 5	No

cassava4.1_010973m|PACid:17981659	cassava4.1_010973m	PETER PAN-like protein	G
cassava4.1_010980m|PACid:17981463	cassava4.1_010980m	DNAJ heat shock family protein	GP
cassava4.1_010982m|PACid:17983391	cassava4.1_010982m	non-intrinsic ABC protein 11	GP
cassava4.1_010985m|PACid:17970520	cassava4.1_010985m	Mitochondrial substrate carrier family protein	No
cassava4.1_010986m|PACid:17985382	cassava4.1_010986m		GP
cassava4.1_010989m|PACid:17981744	cassava4.1_010989m	GroES-like zinc-binding alcohol dehydrogenase family protein	GP
cassava4.1_010990m|PACid:17962085	cassava4.1_010990m	alpha/beta-Hydrolases superfamily protein	No
cassava4.1_010991m|PACid:17979784	cassava4.1_010991m	NAC (No Apical Meristem) domain transcriptional regulator superfamily protein	G
cassava4.1_010994m|PACid:17976059	cassava4.1_010994m	Mitochondrial substrate carrier family protein	G
cassava4.1_010995m|PACid:17993945	cassava4.1_010995m	sterol methyltransferase 1	No
cassava4.1_010997m|PACid:17989284	cassava4.1_010997m	NAD(P)-binding Rossmann-fold superfamily protein	GP
cassava4.1_011007m|PACid:17977714	cassava4.1_011007m	2-oxoglutarate (2OG) and Fe(II)-dependent oxygenase superfamily protein	G
cassava4.1_011008m|PACid:17968021	cassava4.1_011008m	EYES ABSENT homolog	G
cassava4.1_011009m|PACid:17982458	cassava4.1_011009m	zinc transporter 11 precursor	G
cassava4.1_011010m|PACid:17979968	cassava4.1_011010m	Transducin/WD40 repeat-like superfamily protein	GP
cassava4.1_011011m|PACid:17981578	cassava4.1_011011m	Galactose oxidase/kelch repeat superfamily protein	No
cassava4.1_011018m|PACid:17972810	cassava4.1_011018m	alpha/beta-Hydrolases superfamily protein	G
cassava4.1_011023m|PACid:17987235	cassava4.1_011023m	Galactosyltransferase family protein	G
cassava4.1_011025m|PACid:17963022	cassava4.1_011025m	chloroplast outer envelope protein 37	GP
cassava4.1_011037m|PACid:17969842	cassava4.1_011037m	Nucleotide-sugar transporter family protein	G
cassava4.1_011040m|PACid:17961701	cassava4.1_011040m	alpha/beta-Hydrolases superfamily protein	G
cassava4.1_011041m|PACid:17986231	cassava4.1_011041m	alpha/beta-Hydrolases superfamily protein	No
cassava4.1_011043m|PACid:17961734	cassava4.1_011043m	Mo25 family protein	No
cassava4.1_011050m|PACid:17990051	cassava4.1_011050m	Eukaryotic translation initiation factor 2 subunit 1	GP
cassava4.1_011051m|PACid:17983858	cassava4.1_011051m	P-loop containing nucleoside triphosphate hydrolases superfamily protein	No
cassava4.1_011052m|PACid:17959887	cassava4.1_011052m	Galactosyltransferase family protein	No
cassava4.1_011053m|PACid:17976153	cassava4.1_011053m	Core-2/I-branching beta-1,6-N-acetylglucosaminyltransferase family protein	G
cassava4.1_011055m|PACid:17977752	cassava4.1_011055m	cardiolipin synthase	G
cassava4.1_011058m|PACid:17986625	cassava4.1_011058m	Zinc-binding dehydrogenase family protein	GP
cassava4.1_011061m|PACid:17970474	cassava4.1_011061m	alpha/beta-Hydrolases superfamily protein	No
cassava4.1_011064m|PACid:17986177	cassava4.1_011064m	E3 Ubiquitin ligase family protein	G
cassava4.1_011078m|PACid:17987829	cassava4.1_011078m	chloroplastic lipocalin	GP
cassava4.1_011080m|PACid:17967518	cassava4.1_011080m	RING/U-box superfamily protein	No
cassava4.1_011085m|PACid:17969945	cassava4.1_011085m		No
cassava4.1_011086m|PACid:17974155	cassava4.1_011086m	DNAJ heat shock family protein	No
cassava4.1_011091m|PACid:17978257	cassava4.1_011091m	N-terminal nucleophile aminohydrolases (Ntn hydrolases) superfamily protein	GP
cassava4.1_011093m|PACid:17972270	cassava4.1_011093m	NAD(P)-binding Rossmann-fold superfamily protein	GP
cassava4.1_011094m|PACid:17989385	cassava4.1_011094m	type one serine/threonine protein phosphatase 4	No
cassava4.1_011100m|PACid:17977431	cassava4.1_011100m	ribonucleotide reductase 2A	G

cassava4.1_011102m|PACid:17967155	cassava4.1_011102m	S-adenosyl-L-methionine-dependent methyltransferases superfamily protein	No
cassava4.1_011118m|PACid:17989666	cassava4.1_011118m	alpha/beta-Hydrolases superfamily protein	G
cassava4.1_011120m|PACid:17990883	cassava4.1_011120m	farnesyl diphosphate synthase 2	No
cassava4.1_011121m|PACid:17988967	cassava4.1_011121m	Leucine carboxyl methyltransferase	G
cassava4.1_011122m|PACid:17991442	cassava4.1_011122m	golgi nucleotide sugar transporter 5	No
cassava4.1_011123m|PACid:17978346	cassava4.1_011123m	Transducin/WD40 repeat-like superfamily protein	GP
cassava4.1_011125m|PACid:17984513	cassava4.1_011125m		No
cassava4.1_011129m|PACid:17970528	cassava4.1_011129m	Mitochondrial substrate carrier family protein	G
cassava4.1_011132m|PACid:17964238	cassava4.1_011132m	2-oxoglutarate (2OG) and Fe(II)-dependent oxygenase superfamily protein	No
cassava4.1_011133m|PACid:17987642	cassava4.1_011133m	Lactate/malate dehydrogenase family protein	GP
cassava4.1_011135m|PACid:17960892	cassava4.1_011135m	WRKY DNA-binding protein 11	G
cassava4.1_011138m|PACid:17962178	cassava4.1_011138m	homocysteine S-methyltransferase 3	GP
cassava4.1_011140m|PACid:17966406	cassava4.1_011140m	Protein kinase superfamily protein	No
cassava4.1_011147m|PACid:17986682	cassava4.1_011147m	SWAP (Suppressor-of-White-APricot)/surp domain-containing protein	No
cassava4.1_011149m|PACid:17969327	cassava4.1_011149m		G
cassava4.1_011154m|PACid:17963860	cassava4.1_011154m	Transducin/WD40 repeat-like superfamily protein	GP
cassava4.1_011156m|PACid:17985648	cassava4.1_011156m	TRICHOME BIREFRINGENCE-LIKE 41	No
cassava4.1_011159m|PACid:17991391	cassava4.1_011159m	ATPase family associated with various cellular activities (AAA)	GP
cassava4.1_011163m|PACid:17960712	cassava4.1_011163m	Inositol 1,3,4-trisphosphate 5/6-kinase family protein	No
cassava4.1_011165m|PACid:17984899	cassava4.1_011165m	ribosome biogenesis regulatory protein (RRS1) family protein	G
cassava4.1_011169m|PACid:17979863	cassava4.1_011169m	DNAJ heat shock family protein	G
cassava4.1_011172m|PACid:17981801	cassava4.1_011172m	signal peptide peptidase	P
cassava4.1_011173m|PACid:17989205	cassava4.1_011173m	DNAJ heat shock family protein	GP
cassava4.1_011174m|PACid:17964342	cassava4.1_011174m	Tetratricopeptide repeat (TPR)-like superfamily protein	No
cassava4.1_011177m|PACid:17972447	cassava4.1_011177m	adenosine kinase 2	GP
cassava4.1_011178m|PACid:17976002	cassava4.1_011178m	glyoxylate reductase 2	No
cassava4.1_011180m|PACid:17988497	cassava4.1_011180m	Mo25 family protein	GP
cassava4.1_011189m|PACid:17990661	cassava4.1_011189m	recA DNA recombination family protein	No
cassava4.1_011190m|PACid:17982289	cassava4.1_011190m	spermidine synthase 1	GP
cassava4.1_011192m|PACid:17985932	cassava4.1_011192m	CONSTANS-like 4	G
cassava4.1_011193m|PACid:17981668	cassava4.1_011193m	alpha/beta-Hydrolases superfamily protein	G
cassava4.1_011197m|PACid:17981706	cassava4.1_011197m	Inositol monophosphatase family protein	GP
cassava4.1_011199m|PACid:17986332	cassava4.1_011199m	homocysteine methyltransferase 2	No
cassava4.1_011202m|PACid:17971987	cassava4.1_011202m	indigoidine synthase A family protein	G
cassava4.1_011203m|PACid:17972620	cassava4.1_011203m	Transducin/WD40 repeat-like superfamily protein	G
cassava4.1_011207m|PACid:17971504	cassava4.1_011207m	Protein of unknown function (DUF607)	GP
cassava4.1_011211m|PACid:17968808	cassava4.1_011211m	Pathogenesis-related thaumatin superfamily protein	G
cassava4.1_011215m|PACid:17980191	cassava4.1_011215m	geranylgeranyl reductase	GP
cassava4.1_011218m|PACid:17988691	cassava4.1_011218m	Mitochondrial substrate carrier family protein	No

cassava4.1_011222m|PACid:17979510	cassava4.1_011222m	translation initiation factor 3 subunit H1	No
cassava4.1_011224m|PACid:17983839	cassava4.1_011224m	alpha/beta-Hydrolases superfamily protein	GP
cassava4.1_011225m|PACid:17990693	cassava4.1_011225m	nucleosome assembly protein 1;2	No
cassava4.1_011231m|PACid:17962137	cassava4.1_011231m	Protein of unknown function (DUF2361)	No
cassava4.1_011234m|PACid:17962073	cassava4.1_011234m	Adenosylmethionine decarboxylase family protein	No
cassava4.1_011240m|PACid:17988205	cassava4.1_011240m	dsRNA-binding domain-like superfamily protein	No
cassava4.1_011244m|PACid:17988059	cassava4.1_011244m	plastidic type i signal peptidase 1	GP
cassava4.1_011247m|PACid:17992879	cassava4.1_011247m	ZIP metal ion transporter family	No
cassava4.1_011249m|PACid:17981363	cassava4.1_011249m	seven in absentia of Arabidopsis 2	G
cassava4.1_011258m|PACid:17973258	cassava4.1_011258m	alpha-galactosidase 1	GP
cassava4.1_011266m|PACid:17979305	cassava4.1_011266m	FAD-linked oxidases family protein	GP
cassava4.1_011272m|PACid:17964746	cassava4.1_011272m	SBP (S-ribonuclease binding protein) family protein	No
cassava4.1_011275m|PACid:17986944	cassava4.1_011275m	Galactose mutarotase-like superfamily protein	GP
cassava4.1_011276m|PACid:17960253	cassava4.1_011276m	enolase 1	GP
cassava4.1_011279m|PACid:17968772	cassava4.1_011279m	Nucleotide/sugar transporter family protein	No
cassava4.1_011280m|PACid:17960072	cassava4.1_011280m	protein tyrosine phosphatase 1	No
cassava4.1_011281m|PACid:17964730	cassava4.1_011281m	galactinol synthase 4	No
cassava4.1_011283m|PACid:17964943	cassava4.1_011283m	cinnamoyl coa reductase 1	GP
cassava4.1_011284m|PACid:17971226	cassava4.1_011284m	Ras-related small GTP-binding family protein	G
cassava4.1_011285m|PACid:17978207	cassava4.1_011285m	Nucleotide/sugar transporter family protein	No
cassava4.1_011287m|PACid:17960766	cassava4.1_011287m	NAD(P)-binding Rossmann-fold superfamily protein	GP
cassava4.1_011292m|PACid:17991724	cassava4.1_011292m	purple acid phosphatase 17	G
cassava4.1_011293m|PACid:17971385	cassava4.1_011293m	Protein of unknown function (DUF803)	No
cassava4.1_011297m|PACid:17983728	cassava4.1_011297m	Pex2/Pex12 N-terminal domain-containing protein / zinc finger (C3HC4-type RING finger) family protein	G
cassava4.1_011298m|PACid:17970407	cassava4.1_011298m	gibberellin 2-oxidase	No
cassava4.1_011300m|PACid:17985185	cassava4.1_011300m	arginase	P
cassava4.1_011302m|PACid:17963126	cassava4.1_011302m	Transducin/WD40 repeat-like superfamily protein	G
cassava4.1_011307m|PACid:17976807	cassava4.1_011307m	PapD-like superfamily protein	G
cassava4.1_011311m|PACid:17989021	cassava4.1_011311m	Lactate/malate dehydrogenase family protein	No
cassava4.1_011312m|PACid:17982215	cassava4.1_011312m	P-loop containing nucleoside triphosphate hydrolases superfamily protein	No
cassava4.1_011318m|PACid:17992088	cassava4.1_011318m	Protein kinase superfamily protein	No
cassava4.1_011328m|PACid:17988888	cassava4.1_011328m	Nucleotide-diphospho-sugar transferases superfamily protein	No
cassava4.1_011330m|PACid:17976590	cassava4.1_011330m	sequence-specific DNA binding transcription factors;sequence-specific DNA binding	G
cassava4.1_011333m|PACid:17984980	cassava4.1_011333m	fatty acid desaturase family protein	No
cassava4.1_011337m|PACid:17989145	cassava4.1_011337m	syntaxin of plants 31	No
cassava4.1_011338m|PACid:17982387	cassava4.1_011338m	Sec14p-like phosphatidylinositol transfer family protein	No
cassava4.1_011341m|PACid:17984033	cassava4.1_011341m	temperature sensing protein-related	G
cassava4.1_011342m|PACid:17971010	cassava4.1_011342m	Nucleotide-sugar transporter family protein	No
cassava4.1_011347m|PACid:17975907	cassava4.1_011347m	glyceraldehyde-3-phosphate dehydrogenase C subunit 1	GP

cassava4.1_011353m|PACid:17972928	cassava4.1_011353m	FKBP12 interacting protein 37	G
cassava4.1_011356m|PACid:17960248	cassava4.1_011356m	acyl-activating enzyme 17	GP
cassava4.1_011390m|PACid:17989777	cassava4.1_011390m	myb-like HTH transcriptional regulator family protein	G
cassava4.1_011398m|PACid:17990648	cassava4.1_011398m	Mitochondrial substrate carrier family protein	No
cassava4.1_011400m|PACid:17961496	cassava4.1_011400m	purple acid phosphatase 3	G
cassava4.1_011405m|PACid:17969805	cassava4.1_011405m	Sugar isomerase (SIS) family protein	No
cassava4.1_011413m|PACid:17988812	cassava4.1_011413m	alpha/beta-Hydrolases superfamily protein	No
cassava4.1_011417m|PACid:17976206	cassava4.1_011417m	D-isomer specific 2-hydroxyacid dehydrogenase family protein	G
cassava4.1_011419m|PACid:17989926	cassava4.1_011419m	basic helix-loop-helix (bHLH) DNA-binding superfamily protein	No
cassava4.1_011421m|PACid:17964701	cassava4.1_011421m	galactinol synthase 1	G
cassava4.1_011427m|PACid:17964488	cassava4.1_011427m	N-MYC downregulated-like 1	No
cassava4.1_011428m|PACid:17975896	cassava4.1_011428m	Family of unknown function (DUF572)	No
cassava4.1_011429m|PACid:17976453	cassava4.1_011429m	myb-like transcription factor family protein	G
cassava4.1_011432m|PACid:17978063	cassava4.1_011432m	carbonic anhydrase 1	GP
cassava4.1_011439m|PACid:17974967	cassava4.1_011439m	uvrB/uvrC motif-containing protein	GP
cassava4.1_011445m|PACid:17980725	cassava4.1_011445m	Mitochondrial substrate carrier family protein	G
cassava4.1_011446m|PACid:17974555	cassava4.1_011446m	P-loop containing nucleoside triphosphate hydrolases superfamily protein	G
cassava4.1_011453m|PACid:17965186	cassava4.1_011453m	Chlorophyll A-B binding family protein	G
cassava4.1_011455m|PACid:17977444	cassava4.1_011455m		No
cassava4.1_011457m|PACid:17981785	cassava4.1_011457m	F-box family protein	G
cassava4.1_011465m|PACid:17962435	cassava4.1_011465m	ubiquitin family protein	GP
cassava4.1_011466m|PACid:17970546	cassava4.1_011466m	S-adenosyl-L-methionine-dependent methyltransferases superfamily protein	No
cassava4.1_011471m|PACid:17965929	cassava4.1_011471m	S-adenosyl-L-methionine-dependent methyltransferases superfamily protein	No
cassava4.1_011474m|PACid:17991097	cassava4.1_011474m	lipid phosphate phosphatase 3	No
cassava4.1_011479m|PACid:17982790	cassava4.1_011479m	Peptidase family M48 family protein	GP
cassava4.1_011483m|PACid:17974126	cassava4.1_011483m	31-kDa RNA binding protein	GP
cassava4.1_011486m|PACid:17961851	cassava4.1_011486m	Mitochondrial substrate carrier family protein	G
cassava4.1_011487m|PACid:17977097	cassava4.1_011487m	casein kinase alpha 1	G
cassava4.1_011490m|PACid:17978873	cassava4.1_011490m	Phosphotyrosine protein phosphatases superfamily protein	G
cassava4.1_011491m|PACid:17964164	cassava4.1_011491m	farnesyltransferase A	No
cassava4.1_011496m|PACid:17962361	cassava4.1_011496m	RNA-binding (RRM/RBD/RNP motifs) family protein	GP
cassava4.1_011501m|PACid:17966204	cassava4.1_011501m	Transducin/WD40 repeat-like superfamily protein	No
cassava4.1_011502m|PACid:17964753	cassava4.1_011502m	enoyl-CoA hydratase/isomerase D	G
cassava4.1_011505m|PACid:17977192	cassava4.1_011505m	serine racemase	GP
cassava4.1_011507m|PACid:17967748	cassava4.1_011507m	NAD(P)-binding Rossmann-fold superfamily protein	GP
cassava4.1_011514m|PACid:17974098	cassava4.1_011514m	NAD(P)-binding Rossmann-fold superfamily protein	No
cassava4.1_011517m|PACid:17986484	cassava4.1_011517m	F-box family protein	No
cassava4.1_011519m|PACid:17988481	cassava4.1_011519m	sirtuin 2	G
cassava4.1_011531m|PACid:17964879	cassava4.1_011531m	Aldolase-type TIM barrel family protein	GP

cassava4.1_011532m|PACid:17988318	cassava4.1_011532m	UDP-galactose transporter 3	No
cassava4.1_011533m|PACid:17987407	cassava4.1_011533m	CLP protease proteolytic subunit 3	GP
cassava4.1_011539m|PACid:17987338	cassava4.1_011539m	PS II oxygen-evolving complex 1	GP
cassava4.1_011543m|PACid:17983732	cassava4.1_011543m	mitotic phosphoprotein N\' end (MPPN) family protein	No
cassava4.1_011544m|PACid:17960049	cassava4.1_011544m	Tetratricopeptide repeat (TPR)-like superfamily protein	No
cassava4.1_011545m|PACid:17964514	cassava4.1_011545m	SPFH/Band 7/PHB domain-containing membrane-associated protein family	GP
cassava4.1_011548m|PACid:17965202	cassava4.1_011548m	ATP12 protein-related	GP
cassava4.1_011549m|PACid:17988399	cassava4.1_011549m	Acyl-CoA N-acyltransferases (NAT) superfamily protein	No
cassava4.1_011550m|PACid:17993466	cassava4.1_011550m	Lactate/malate dehydrogenase family protein	GP
cassava4.1_011551m|PACid:17971533	cassava4.1_011551m	NAD(P)-binding Rossmann-fold superfamily protein	GP
cassava4.1_011552m|PACid:17963225	cassava4.1_011552m	Protein kinase superfamily protein	GP
cassava4.1_011556m|PACid:17985888	cassava4.1_011556m	Peroxidase superfamily protein	No
cassava4.1_011563m|PACid:17965167	cassava4.1_011563m	xyloglucan endotransglucosylase/hydrolase 28	G
cassava4.1_011568m|PACid:17988816	cassava4.1_011568m	F-box family protein	G
cassava4.1_011569m|PACid:17976554	cassava4.1_011569m	lipid phosphate phosphatase 2	G
cassava4.1_011570m|PACid:17988416	cassava4.1_011570m	CYCLIN D1;1	No
cassava4.1_011571m|PACid:17972642	cassava4.1_011571m	Metallo-hydrolase/oxidoreductase superfamily protein	No
cassava4.1_011573m|PACid:17973600	cassava4.1_011573m	Amino acid kinase family protein	G
cassava4.1_011574m|PACid:17966412	cassava4.1_011574m	Peroxidase superfamily protein	P
cassava4.1_011578m|PACid:17975802	cassava4.1_011578m	pfkB-like carbohydrate kinase family protein	GP
cassava4.1_011581m|PACid:17986020	cassava4.1_011581m		No
cassava4.1_011584m|PACid:17982732	cassava4.1_011584m	pfkB-like carbohydrate kinase family protein	GP
cassava4.1_011585m|PACid:17969357	cassava4.1_011585m	xyloglucan endotransglucosylase/hydrolase 9	GP
cassava4.1_011592m|PACid:17973534	cassava4.1_011592m	Zinc finger C-x8-C-x5-C-x3-H type family protein	G
cassava4.1_011593m|PACid:17971486	cassava4.1_011593m	Mitochondrial substrate carrier family protein	No
cassava4.1_011604m|PACid:17986857	cassava4.1_011604m	Peroxidase superfamily protein	GP
cassava4.1_011608m|PACid:17968631	cassava4.1_011608m	Peptidase C12, ubiquitin carboxyl-terminal hydrolase 1	GP
cassava4.1_011616m|PACid:17978317	cassava4.1_011616m	ARM repeat superfamily protein	G
cassava4.1_011622m|PACid:17981096	cassava4.1_011622m	aspartate-glutamate racemase family	G
cassava4.1_011625m|PACid:17974209	cassava4.1_011625m	KNOTTED1-like homeobox gene 6	No
cassava4.1_011626m|PACid:17991606	cassava4.1_011626m	Nucleotide-diphospho-sugar transferases superfamily protein	G
cassava4.1_011627m|PACid:17992288	cassava4.1_011627m	syntaxin of plants 121	P
cassava4.1_011631m|PACid:17987654	cassava4.1_011631m	Polynucleotidyl transferase, ribonuclease H-like superfamily protein	G
cassava4.1_011632m|PACid:17986963	cassava4.1_011632m	Transducin/WD40 repeat-like superfamily protein	GP
cassava4.1_011639m|PACid:17985578	cassava4.1_011639m	RHOMBOID-like protein 3	G
cassava4.1_011640m|PACid:17962094	cassava4.1_011640m	Tetratricopeptide repeat (TPR)-like superfamily protein	No
cassava4.1_011644m|PACid:17968142	cassava4.1_011644m	receptor for activated C kinase 1B	GP
cassava4.1_011646m|PACid:17976911	cassava4.1_011646m	Ferritin/ribonucleotide reductase-like family protein	No
cassava4.1_011647m|PACid:17987352	cassava4.1_011647m	plastid transcriptionally active 14	G

cassava4.1_011651m|PACid:17978050	cassava4.1_011651m	SNARE associated Golgi protein family	G
cassava4.1_011652m|PACid:17987033	cassava4.1_011652m	Oxidoreductase, zinc-binding dehydrogenase family protein	P
cassava4.1_011656m|PACid:17985391	cassava4.1_011656m	2-oxoglutarate (2OG) and Fe(II)-dependent oxygenase superfamily protein	GP
cassava4.1_011660m|PACid:17972132	cassava4.1_011660m	Mitochondrial substrate carrier family protein	GP
cassava4.1_011665m|PACid:17981159	cassava4.1_011665m	Pectinacetylesterase family protein	No
cassava4.1_011666m|PACid:17973732	cassava4.1_011666m	phosphatidylglycerolphosphate synthase 1	No
cassava4.1_011668m|PACid:17984854	cassava4.1_011668m	methyltransferases	G
cassava4.1_011670m|PACid:17993667	cassava4.1_011670m	SPFH/Band 7/PHB domain-containing membrane-associated protein family	GP
cassava4.1_011672m|PACid:17963443	cassava4.1_011672m	NAD(P)-binding Rossmann-fold superfamily protein	GP
cassava4.1_011684m|PACid:17984831	cassava4.1_011684m	BTB/POZ domain-containing protein	No
cassava4.1_011687m|PACid:17960430	cassava4.1_011687m	bifunctional nuclease in basal defense response 1	No
cassava4.1_011690m|PACid:17972484	cassava4.1_011690m	hydroxyproline-rich glycoprotein family protein	GP
cassava4.1_011697m|PACid:17969412	cassava4.1_011697m	Phosphoenolpyruvate carboxylase family protein	No
cassava4.1_011698m|PACid:17971228	cassava4.1_011698m	S-adenosylmethionine carrier 1	GP
cassava4.1_011699m|PACid:17984621	cassava4.1_011699m	purple acid phosphatase 3	No
cassava4.1_011700m|PACid:17982388	cassava4.1_011700m	potassium channel beta subunit 1	No
cassava4.1_011701m|PACid:17968017	cassava4.1_011701m	FKBP-type peptidyl-prolyl cis-trans isomerase family protein	No
cassava4.1_011703m|PACid:17971520	cassava4.1_011703m	Eukaryotic translation initiation factor eIF2A family protein	No
cassava4.1_011704m|PACid:17980222	cassava4.1_011704m	Peroxidase superfamily protein	GP
cassava4.1_011707m|PACid:17991540	cassava4.1_011707m	fibrillin	GP
cassava4.1_011711m|PACid:17982078	cassava4.1_011711m	glyoxalase 2-4	No
cassava4.1_011713m|PACid:17967104	cassava4.1_011713m	glyoxalase 2-4	G
cassava4.1_011715m|PACid:17960376	cassava4.1_011715m	Leucine-rich repeat (LRR) family protein	No
cassava4.1_011716m|PACid:17988368	cassava4.1_011716m	RNA-binding (RRM/RBD/RNP motifs) family protein	G
cassava4.1_011729m|PACid:17989246	cassava4.1_011729m	Galactose oxidase/kelch repeat superfamily protein	GP
cassava4.1_011730m|PACid:17969162	cassava4.1_011730m	Homocysteine S-methyltransferase family protein	G
cassava4.1_011736m|PACid:17988960	cassava4.1_011736m	SPX (SYG1/Pho81/XPR1) domain-containing protein	G
cassava4.1_011738m|PACid:17979243	cassava4.1_011738m	Peroxidase superfamily protein	GP
cassava4.1_011739m|PACid:17984263	cassava4.1_011739m	N-terminal nucleophile aminohydrolases (Ntn hydrolases) superfamily protein	GP
cassava4.1_011740m|PACid:17979928	cassava4.1_011740m	polygalacturonase inhibiting protein 1	P
cassava4.1_011746m|PACid:17964973	cassava4.1_011746m		G
cassava4.1_011747m|PACid:17981641	cassava4.1_011747m	Cysteine proteinases superfamily protein	G
cassava4.1_011750m|PACid:17991365	cassava4.1_011750m	BTB/POZ domain-containing protein	G
cassava4.1_011751m|PACid:17984095	cassava4.1_011751m	2-oxoglutarate (2OG) and Fe(II)-dependent oxygenase superfamily protein	GP
cassava4.1_011752m|PACid:17992474	cassava4.1_011752m	Tetratricopeptide repeat (TPR)-like superfamily protein	GP
cassava4.1_011756m|PACid:17980554	cassava4.1_011756m	end binding protein 1C	No
cassava4.1_011757m|PACid:17989142	cassava4.1_011757m	NAD(P)-binding Rossmann-fold superfamily protein	GP
cassava4.1_011759m|PACid:17984954	cassava4.1_011759m	Calcium-dependent ARF-type GTPase activating protein family	G
cassava4.1_011761m|PACid:17966617	cassava4.1_011761m	ATP-dependent caseinolytic (Clp) protease/crotonase family protein	GP

cassava4.1_011763m|PACid:17993641	cassava4.1_011763m	DNAJ heat shock family protein	G
cassava4.1_011768m|PACid:17976682	cassava4.1_011768m	Peroxidase superfamily protein	P
cassava4.1_011773m|PACid:17961404	cassava4.1_011773m	Transducin/WD40 repeat-like superfamily protein	GP
cassava4.1_011779m|PACid:17960166	cassava4.1_011779m	Peroxidase superfamily protein	GP
cassava4.1_011781m|PACid:17989589	cassava4.1_011781m	Flavodoxin family protein	No
cassava4.1_011782m|PACid:17982804	cassava4.1_011782m	Alpha-helical ferredoxin	GP
cassava4.1_011783m|PACid:17972862	cassava4.1_011783m	Phosphoribosyltransferase family protein	P
cassava4.1_011785m|PACid:17989415	cassava4.1_011785m	O-acetylserine (thiol) lyase (OAS-TL) isoform A1	GP
cassava4.1_011790m|PACid:17976433	cassava4.1_011790m	formyltetrahydrofolate deformylase, putative	GP
cassava4.1_011791m|PACid:17973738	cassava4.1_011791m	Nitrilase/cyanide hydratase and apolipoprotein N-acyltransferase family protein	GP
cassava4.1_011792m|PACid:17974399	cassava4.1_011792m	Plant protein 1589 of unknown function	No
cassava4.1_011793m|PACid:17984045	cassava4.1_011793m	dicarboxylate carrier 2	No
cassava4.1_011794m|PACid:17993253	cassava4.1_011794m	syntaxin of plants 43	G
cassava4.1_011798m|PACid:17976413	cassava4.1_011798m	ATP-dependent caseinolytic (Clp) protease/crotonase family protein	GP
cassava4.1_011803m|PACid:17968403	cassava4.1_011803m	ENTH/VHS/GAT family protein	No
cassava4.1_011808m|PACid:17989475	cassava4.1_011808m	Homeodomain-like superfamily protein	No
cassava4.1_011809m|PACid:17961923	cassava4.1_011809m	O-acetylserine (thiol) lyase (OAS-TL) isoform A1	P
cassava4.1_011811m|PACid:17964649	cassava4.1_011811m	Tetratricopeptide repeat (TPR)-like superfamily protein	No
cassava4.1_011816m|PACid:17990832	cassava4.1_011816m	oxidoreductase, zinc-binding dehydrogenase family protein	GP
cassava4.1_011825m|PACid:17990927	cassava4.1_011825m	Mitochondrial substrate carrier family protein	No
cassava4.1_011830m|PACid:17965914	cassava4.1_011830m	FMN binding	GP
cassava4.1_011832m|PACid:17980056	cassava4.1_011832m	S-adenosyl-L-methionine-dependent methyltransferases superfamily protein	GP
cassava4.1_011836m|PACid:17964677	cassava4.1_011836m	Protein of unknown function (DUF1295)	No
cassava4.1_011838m|PACid:17972376	cassava4.1_011838m	pfkB-like carbohydrate kinase family protein	P
cassava4.1_011842m|PACid:17966712	cassava4.1_011842m	PGR5-LIKE A	GP
cassava4.1_011845m|PACid:17965908	cassava4.1_011845m	Methyltransferase family protein	G
cassava4.1_011846m|PACid:17972300	cassava4.1_011846m	DEGP protease 5	GP
cassava4.1_011852m|PACid:17984869	cassava4.1_011852m	FAD/NAD(P)-binding oxidoreductase	GP
cassava4.1_011854m|PACid:17985369	cassava4.1_011854m	solute:sodium symporters;urea transmembrane transporters	No
cassava4.1_011856m|PACid:17992980	cassava4.1_011856m	dihydroflavonol 4-reductase-like1	No
cassava4.1_011858m|PACid:17986490	cassava4.1_011858m	NAD(P)-linked oxidoreductase superfamily protein	No
cassava4.1_011864m|PACid:17964783	cassava4.1_011864m	replication factor C subunit 3	No
cassava4.1_011868m|PACid:17990035	cassava4.1_011868m	NagB/RpiA/CoA transferase-like superfamily protein	GP
cassava4.1_011869m|PACid:17965954	cassava4.1_011869m	DNA-damage-repair/toleration protein (DRT102)	GP
cassava4.1_011873m|PACid:17960334	cassava4.1_011873m	Protease-associated (PA) RING/U-box zinc finger family protein	G
cassava4.1_011879m|PACid:17962677	cassava4.1_011879m	N-terminal nucleophile aminohydrolases (Ntn hydrolases) superfamily protein	GP
cassava4.1_011880m|PACid:17985376	cassava4.1_011880m	RNA-binding (RRM/RBD/RNP motifs) family protein	No
cassava4.1_011881m|PACid:17978596	cassava4.1_011881m	Protein with RING/U-box and TRAF-like domains	No
cassava4.1_011882m|PACid:17984216	cassava4.1_011882m	NAD(P)-linked oxidoreductase superfamily protein	GP

cassava4.1_011883m|PACid:17968964	cassava4.1_011883m	gamma subunit of Mt ATP synthase	GP
cassava4.1_011884m|PACid:17965031	cassava4.1_011884m	SUMO-activating enzyme 1A	No
cassava4.1_011897m|PACid:17974351	cassava4.1_011897m	myb domain protein 63	No
cassava4.1_011908m|PACid:17991422	cassava4.1_011908m	F-box protein 7	G
cassava4.1_011910m|PACid:17985503	cassava4.1_011910m	NAD(P)-binding Rossmann-fold superfamily protein	No
cassava4.1_011913m|PACid:17973832	cassava4.1_011913m	nicotianamine synthase 3	No
cassava4.1_011922m|PACid:17977429	cassava4.1_011922m	isopentenyltransferase 5	No
cassava4.1_011927m|PACid:17965735	cassava4.1_011927m	Calcineurin-like metallo-phosphoesterase superfamily protein	GP
cassava4.1_011929m|PACid:17977899	cassava4.1_011929m	oxidoreductase, 2OG-Fe(II) oxygenase family protein	G
cassava4.1_011932m|PACid:17990764	cassava4.1_011932m	Transducin/WD40 repeat-like superfamily protein	GP
cassava4.1_011939m|PACid:17981561	cassava4.1_011939m	syntaxin of plants 42	No
cassava4.1_011944m|PACid:17982524	cassava4.1_011944m		G
cassava4.1_011945m|PACid:17963522	cassava4.1_011945m	Peroxidase superfamily protein	GP
cassava4.1_011946m|PACid:17991613	cassava4.1_011946m	Ribosomal protein L10 family protein	No
cassava4.1_011947m|PACid:17962152	cassava4.1_011947m	alpha/beta-Hydrolases superfamily protein	P
cassava4.1_011950m|PACid:17963178	cassava4.1_011950m	Peroxidase superfamily protein	No
cassava4.1_011951m|PACid:17970149	cassava4.1_011951m	alpha/beta-Hydrolases superfamily protein	No
cassava4.1_011955m|PACid:17991382	cassava4.1_011955m	YbaK/aminoacyl-tRNA synthetase-associated domain	GP
cassava4.1_011958m|PACid:17984834	cassava4.1_011958m	uridine-ribohydrolase 2	GP
cassava4.1_011960m|PACid:17960400	cassava4.1_011960m	Pathogenesis-related thaumatin superfamily protein	G
cassava4.1_011962m|PACid:17962648	cassava4.1_011962m	ribosomal RNA processing 4	G
cassava4.1_011965m|PACid:17984246	cassava4.1_011965m	MAP kinase kinase 9	G
cassava4.1_011967m|PACid:17988289	cassava4.1_011967m	Transducin/WD40 repeat-like superfamily protein	No
cassava4.1_011968m|PACid:17962856	cassava4.1_011968m	NAD(P)-binding Rossmann-fold superfamily protein	No
cassava4.1_011970m|PACid:17979513	cassava4.1_011970m	Pathogenesis-related thaumatin superfamily protein	No
cassava4.1_011971m|PACid:17987423	cassava4.1_011971m	Mitochondrial substrate carrier family protein	GP
cassava4.1_011979m|PACid:17993052	cassava4.1_011979m	4-phospho-panto-thenoylcysteine synthetase	No
cassava4.1_011980m|PACid:17977294	cassava4.1_011980m	RHOMBOID-like 2	G
cassava4.1_011981m|PACid:17983317	cassava4.1_011981m		G
cassava4.1_011988m|PACid:17994037	cassava4.1_011988m	SET domain protein 38	No
cassava4.1_011990m|PACid:17970986	cassava4.1_011990m	Calcineurin-like metallo-phosphoesterase superfamily protein	GP
cassava4.1_011991m|PACid:17974188	cassava4.1_011991m	Haloacid dehalogenase-like hydrolase (HAD) superfamily protein	GP
cassava4.1_011992m|PACid:17961947	cassava4.1_011992m	peroxisomal adenine nucleotide carrier 1	No
cassava4.1_011995m|PACid:17964132	cassava4.1_011995m	Calcineurin-like metallo-phosphoesterase superfamily protein	G
cassava4.1_011996m|PACid:17974161	cassava4.1_011996m	Calcineurin-like metallo-phosphoesterase superfamily protein	G
cassava4.1_012000m|PACid:17971721	cassava4.1_012000m	homolog of bacterial PANC	GP
cassava4.1_012002m|PACid:17962285	cassava4.1_012002m	beta-ketoacyl reductase 1	P
cassava4.1_012004m|PACid:17963387	cassava4.1_012004m	beta-ketoacyl reductase 2	No
cassava4.1_012006m|PACid:17987854	cassava4.1_012006m	SKP1 interacting partner 1	No

cassava4.1_012014m|PACid:17984819	cassava4.1_012014m	Dihydrodipicolinate reductase, bacterial/plant	GP
cassava4.1_012016m|PACid:17981009	cassava4.1_012016m	triosephosphate isomerase	GP
cassava4.1_012020m|PACid:17989861	cassava4.1_012020m	Peroxidase superfamily protein	P
cassava4.1_012030m|PACid:17965833	cassava4.1_012030m	peroxisomal adenine nucleotide carrier 1	GP
cassava4.1_012033m|PACid:17965890	cassava4.1_012033m	RNA binding (RRM/RBD/RNP motifs) family protein	G
cassava4.1_012034m|PACid:17987730	cassava4.1_012034m	Cell differentiation, Rcd1-like protein	No
cassava4.1_012044m|PACid:17990187	cassava4.1_012044m	NAD(P)-binding Rossmann-fold superfamily protein	GP
cassava4.1_012045m|PACid:17980071	cassava4.1_012045m	ethylene-forming enzyme	GP
cassava4.1_012046m|PACid:17972404	cassava4.1_012046m	Lysyl-tRNA synthetase, class II	No
cassava4.1_012047m|PACid:17961033	cassava4.1_012047m	Aldolase-type TIM barrel family protein	GP
cassava4.1_012049m|PACid:17976965	cassava4.1_012049m	ARM repeat superfamily protein	G
cassava4.1_012054m|PACid:17980562	cassava4.1_012054m	MRG family protein	No
cassava4.1_012058m|PACid:17966167	cassava4.1_012058m	P-loop containing nucleoside triphosphate hydrolases superfamily protein	No
cassava4.1_012061m|PACid:17984383	cassava4.1_012061m	annexin 3	No
cassava4.1_012064m|PACid:17962279	cassava4.1_012064m	Peroxidase superfamily protein	No
cassava4.1_012065m|PACid:17984417	cassava4.1_012065m	peroxin 7	GP
cassava4.1_012070m|PACid:17963277	cassava4.1_012070m	NAD(P)-binding Rossmann-fold superfamily protein	GP
cassava4.1_012072m|PACid:17991712	cassava4.1_012072m	G-protein-coupled receptor 1	G
cassava4.1_012074m|PACid:17990628	cassava4.1_012074m	PLC-like phosphodiesterases superfamily protein	GP
cassava4.1_012091m|PACid:17994135	cassava4.1_012091m	alpha/beta-Hydrolases superfamily protein	G
cassava4.1_012093m|PACid:17965573	cassava4.1_012093m	alpha/beta-Hydrolases superfamily protein	No
cassava4.1_012094m|PACid:17964551	cassava4.1_012094m	alpha/beta-Hydrolases superfamily protein	No
cassava4.1_012097m|PACid:17975563	cassava4.1_012097m	Galactose mutarotase-like superfamily protein	No
cassava4.1_012104m|PACid:17966359	cassava4.1_012104m	alpha/beta-Hydrolases superfamily protein	GP
cassava4.1_012107m|PACid:17980838	cassava4.1_012107m	Galactose mutarotase-like superfamily protein	GP
cassava4.1_012108m|PACid:17985563	cassava4.1_012108m	RNI-like superfamily protein	No
cassava4.1_012110m|PACid:17992261	cassava4.1_012110m	chaperone protein dnaJ-related	G
cassava4.1_012111m|PACid:17981696	cassava4.1_012111m	alpha/beta-Hydrolases superfamily protein	GP
cassava4.1_012120m|PACid:17969326	cassava4.1_012120m	cyclin-dependent kinase B1;2	No
cassava4.1_012121m|PACid:17981725	cassava4.1_012121m	ribosomal protein L3 plastid	GP
cassava4.1_012123m|PACid:17988778	cassava4.1_012123m	annexin 2	GP
cassava4.1_012125m|PACid:17961089	cassava4.1_012125m	Calcineurin-like metallo-phosphoesterase superfamily protein	GP
cassava4.1_012126m|PACid:17980735	cassava4.1_012126m	type one serine/threonine protein phosphatase 4	No
cassava4.1_012129m|PACid:17973018	cassava4.1_012129m	translocase of the outer mitochondrial membrane 40	GP
cassava4.1_012132m|PACid:17987554	cassava4.1_012132m	lipid phosphate phosphatase 3	No
cassava4.1_012133m|PACid:17988902	cassava4.1_012133m	type one serine/threonine protein phosphatase 4	G
cassava4.1_012134m|PACid:17986408	cassava4.1_012134m	S-adenosyl-L-methionine-dependent methyltransferases superfamily protein	G
cassava4.1_012136m|PACid:17974992	cassava4.1_012136m	basic chitinase	GP
cassava4.1_012139m|PACid:17970793	cassava4.1_012139m	Arabidopsis Inositol phosphorylceramide synthase 1	No

cassava4.1_012143m|PACid:17988242	cassava4.1_012143m	Transducin/WD40 repeat-like superfamily protein	GP
cassava4.1_012149m|PACid:17961107	cassava4.1_012149m	Ribosomal RNA processing Brix domain protein	GP
cassava4.1_012159m|PACid:17980267	cassava4.1_012159m	Mitochondrial substrate carrier family protein	No
cassava4.1_012160m|PACid:17982428	cassava4.1_012160m	Homeodomain-like superfamily protein	No
cassava4.1_012161m|PACid:17993179	cassava4.1_012161m	Pseudouridine synthase family protein	No
cassava4.1_012162m|PACid:17982273	cassava4.1_012162m	xyloglucan:xyloglucosyl transferase 33	G
cassava4.1_012169m|PACid:17991523	cassava4.1_012169m	Ribosomal protein L4/L1 family	G
cassava4.1_012173m|PACid:17991801	cassava4.1_012173m	alpha/beta-Hydrolases superfamily protein	GP
cassava4.1_012178m|PACid:17981563	cassava4.1_012178m	Transducin/WD40 repeat-like superfamily protein	No
cassava4.1_012190m|PACid:17963953	cassava4.1_012190m	S-adenosyl-L-methionine-dependent methyltransferases superfamily protein	No
cassava4.1_012192m|PACid:17984371	cassava4.1_012192m	annexin 8	P
cassava4.1_012194m|PACid:17976483	cassava4.1_012194m	basic pentacysteine 4	No
cassava4.1_012195m|PACid:17968993	cassava4.1_012195m	light-harvesting chlorophyll-protein complex I subunit A4	G
cassava4.1_012202m|PACid:17981570	cassava4.1_012202m	NAD+ transporter 1	G
cassava4.1_012203m|PACid:17981283	cassava4.1_012203m	RAB geranylgeranyl transferase beta subunit 2	No
cassava4.1_012206m|PACid:17964601	cassava4.1_012206m	autophagy 3 (APG3)	No
cassava4.1_012207m|PACid:17984144	cassava4.1_012207m	D-isomer specific 2-hydroxyacid dehydrogenase family protein	No
cassava4.1_012212m|PACid:17976183	cassava4.1_012212m	vitamin E pathway gene 5	G
cassava4.1_012219m|PACid:17963600	cassava4.1_012219m	ABC transporter of the mitochondrion 3	P
cassava4.1_012220m|PACid:17964979	cassava4.1_012220m	NAD(P)-binding Rossmann-fold superfamily protein	G
cassava4.1_012223m|PACid:17984395	cassava4.1_012223m	ARM repeat superfamily protein	G
cassava4.1_012226m|PACid:17984255	cassava4.1_012226m	basic helix-loop-helix (bHLH) DNA-binding superfamily protein	No
cassava4.1_012227m|PACid:17981284	cassava4.1_012227m	RAB geranylgeranyl transferase beta subunit 1	G
cassava4.1_012228m|PACid:17965715	cassava4.1_012228m	cyclin H;1	No
cassava4.1_012229m|PACid:17964716	cassava4.1_012229m	syntaxin of plants 111	No
cassava4.1_012231m|PACid:17987146	cassava4.1_012231m	S-adenosyl-L-methionine-dependent methyltransferases superfamily protein	No
cassava4.1_012234m|PACid:17969283	cassava4.1_012234m	TatD related DNase	No
cassava4.1_012245m|PACid:17988409	cassava4.1_012245m	ABL interactor-like protein 2	No
cassava4.1_012246m|PACid:17960737	cassava4.1_012246m	NAD(P)-binding Rossmann-fold superfamily protein	GP
cassava4.1_012247m|PACid:17974727	cassava4.1_012247m	Cyclin-like family protein	GP
cassava4.1_012250m|PACid:17991924	cassava4.1_012250m	type one serine/threonine protein phosphatase 2	G
cassava4.1_012253m|PACid:17970517	cassava4.1_012253m	protein phosphatase 2A-4	No
cassava4.1_012254m|PACid:17978425	cassava4.1_012254m	Duplicated homeodomain-like superfamily protein	No
cassava4.1_012256m|PACid:17977553	cassava4.1_012256m	Zinc-binding dehydrogenase family protein	No
cassava4.1_012259m|PACid:17968550	cassava4.1_012259m	Transducin/WD40 repeat-like superfamily protein	GP
cassava4.1_012261m|PACid:17962743	cassava4.1_012261m	endoplasmic reticulum-adenine nucleotide transporter 1	G
cassava4.1_012264m|PACid:17979771	cassava4.1_012264m	S-adenosyl-L-methionine-dependent methyltransferases superfamily protein	No
cassava4.1_012266m|PACid:17973437	cassava4.1_012266m	CTC-interacting domain 9	G
cassava4.1_012271m|PACid:17962626	cassava4.1_012271m	cyclin-dependent kinase B2;2	No

cassava4.1_012274m|PACid:17987387	cassava4.1_012274m	transporter associated with antigen processing protein 2	G
cassava4.1_012279m|PACid:17981538	cassava4.1_012279m	NAD(P)-binding Rossmann-fold superfamily protein	No
cassava4.1_012280m|PACid:17971399	cassava4.1_012280m	40s ribosomal protein SA	P
cassava4.1_012284m|PACid:17987927	cassava4.1_012284m	Mov34/MPN/PAD-1 family protein	No
cassava4.1_012285m|PACid:17967359	cassava4.1_012285m	inositol polyphosphate kinase 2 beta	No
cassava4.1_012297m|PACid:17960472	cassava4.1_012297m	Brassinosteroid signalling positive regulator (BZR1) family protein	G
cassava4.1_012304m|PACid:17985654	cassava4.1_012304m	uracil dna glycosylase	G
cassava4.1_012309m|PACid:17982662	cassava4.1_012309m	Translation elongation factor EF1B/ribosomal protein S6 family protein	G
cassava4.1_012313m|PACid:17986677	cassava4.1_012313m	Erythronate-4-phosphate dehydrogenase family protein	No
cassava4.1_012330m|PACid:17976678	cassava4.1_012330m	Ribosomal RNA processing Brix domain protein	No
cassava4.1_012341m|PACid:17960866	cassava4.1_012341m	Homeodomain-like superfamily protein	No
cassava4.1_012343m|PACid:17989716	cassava4.1_012343m	lipase 1	G
cassava4.1_012345m|PACid:17966340	cassava4.1_012345m	Galactose mutarotase-like superfamily protein	No
cassava4.1_012350m|PACid:17976921	cassava4.1_012350m	Cytochrome C1 family	GP
cassava4.1_012353m|PACid:17980093	cassava4.1_012353m	acetyl Co-enzyme a carboxylase biotin carboxylase subunit	P
cassava4.1_012354m|PACid:17965221	cassava4.1_012354m	Cytochrome C1 family	No
cassava4.1_012363m|PACid:17963101	cassava4.1_012363m	S-adenosyl-L-methionine-dependent methyltransferases superfamily protein	G
cassava4.1_012370m|PACid:17985908	cassava4.1_012370m	myo-inositol oxygenase 4	G
cassava4.1_012372m|PACid:17979912	cassava4.1_012372m	Cation efflux family protein	No
cassava4.1_012374m|PACid:17961994	cassava4.1_012374m	Survival protein SurE-like phosphatase/nucleotidase	G
cassava4.1_012375m|PACid:17988882	cassava4.1_012375m	3-oxo-5-alpha-steroid 4-dehydrogenase family protein	GP
cassava4.1_012380m|PACid:17991600	cassava4.1_012380m	syntaxin of plants 81	G
cassava4.1_012389m|PACid:17979624	cassava4.1_012389m	ubiquitin-conjugating enzyme 32	G
cassava4.1_012391m|PACid:17970894	cassava4.1_012391m		GP
cassava4.1_012396m|PACid:17969519	cassava4.1_012396m	Mitochondrial substrate carrier family protein	No
cassava4.1_012399m|PACid:17973771	cassava4.1_012399m	rhomboid protein-related	G
cassava4.1_012401m|PACid:17976125	cassava4.1_012401m	uricase / urate oxidase / nodulin 35, putative	GP
cassava4.1_012402m|PACid:17992541	cassava4.1_012402m	Aldolase-type TIM barrel family protein	GP
cassava4.1_012404m|PACid:17971991	cassava4.1_012404m	phosphate transporter 3;3	G
cassava4.1_012405m|PACid:17971529	cassava4.1_012405m		G
cassava4.1_012407m|PACid:17961122	cassava4.1_012407m	NAD(P)-linked oxidoreductase superfamily protein	GP
cassava4.1_012416m|PACid:17990157	cassava4.1_012416m	enoyl-CoA hydratase 2	GP
cassava4.1_012426m|PACid:17975715	cassava4.1_012426m	DHHC-type zinc finger family protein	No
cassava4.1_012428m|PACid:17992433	cassava4.1_012428m	6-phosphogluconate dehydrogenase family protein	GP
cassava4.1_012429m|PACid:17974132	cassava4.1_012429m	protein-l-isoaspartate methyltransferase 1	GP
cassava4.1_012442m|PACid:17966711	cassava4.1_012442m	nudix hydrolase homolog 14	GP
cassava4.1_012443m|PACid:17971797	cassava4.1_012443m	histone acetyltransferase of the MYST family 1	G
cassava4.1_012444m|PACid:17976465	cassava4.1_012444m	CLP protease R subunit 4	G
cassava4.1_012445m|PACid:17968455	cassava4.1_012445m	NmrA-like negative transcriptional regulator family protein	G

cassava4.1_012448m|PACid:17968446	cassava4.1_012448m	NmrA-like negative transcriptional regulator family protein	GP
cassava4.1_012454m|PACid:17971994	cassava4.1_012454m	purin-rich alpha 1	GP
cassava4.1_012457m|PACid:17966047	cassava4.1_012457m	Protein of unknown function, transmembrane-40	G
cassava4.1_012458m|PACid:17963681	cassava4.1_012458m	nuclear encoded CLP protease 5	GP
cassava4.1_012472m|PACid:17972236	cassava4.1_012472m	coenzyme Q 3	No
cassava4.1_012475m|PACid:17981478	cassava4.1_012475m	pale cress protein (PAC)	G
cassava4.1_012484m|PACid:17966362	cassava4.1_012484m	zinc finger (C2H2 type) family protein	No
cassava4.1_012490m|PACid:17963228	cassava4.1_012490m	DHHC-type zinc finger family protein	G
cassava4.1_012492m|PACid:17978001	cassava4.1_012492m	GCIP-interacting family protein	G
cassava4.1_012496m|PACid:17988379	cassava4.1_012496m	protein phosphatase 2A-2	GP
cassava4.1_012499m|PACid:17991548	cassava4.1_012499m	Coproporphyrinogen III oxidase	No
cassava4.1_012501m|PACid:17971161	cassava4.1_012501m	aldose 1-epimerase family protein	GP
cassava4.1_012502m|PACid:17966039	cassava4.1_012502m	Subunits of heterodimeric actin filament capping protein Capz superfamily	G
cassava4.1_012506m|PACid:17962696	cassava4.1_012506m	LAG1 longevity assurance homolog 3	No
cassava4.1_012509m|PACid:17970146	cassava4.1_012509m	secretory carrier 3	No
cassava4.1_012516m|PACid:17970630	cassava4.1_012516m	Ribosomal protein S5 family protein	GP
cassava4.1_012518m|PACid:17993911	cassava4.1_012518m	Haloacid dehalogenase-like hydrolase (HAD) superfamily protein	GP
cassava4.1_012521m|PACid:17993662	cassava4.1_012521m	protein phosphatase 2A-2	No
cassava4.1_012531m|PACid:17987349	cassava4.1_012531m	Molybdenum cofactor sulfurase family protein	G
cassava4.1_012540m|PACid:17975096	cassava4.1_012540m	protein phosphatase X 2	G
cassava4.1_012545m|PACid:17983071	cassava4.1_012545m	ubiquitin 4	G
cassava4.1_012552m|PACid:17968988	cassava4.1_012552m	soluble N-ethylmaleimide-sensitive factor adaptor protein 33	No
cassava4.1_012556m|PACid:17963270	cassava4.1_012556m	myb domain protein 17	G
cassava4.1_012558m|PACid:17974464	cassava4.1_012558m	plant uncoupling mitochondrial protein 1	GP
cassava4.1_012564m|PACid:17976199	cassava4.1_012564m	Ribosomal RNA adenine dimethylase family protein	No
cassava4.1_012569m|PACid:17993688	cassava4.1_012569m	CAX-interacting protein 2	GP
cassava4.1_012570m|PACid:17963529	cassava4.1_012570m	basic helix-loop-helix (bHLH) DNA-binding superfamily protein	G
cassava4.1_012571m|PACid:17987641	cassava4.1_012571m	homogentisate 1,2-dioxygenase	GP
cassava4.1_012577m|PACid:17991505	cassava4.1_012577m	plant UBX domain containing protein 4	GP
cassava4.1_012578m|PACid:17967288	cassava4.1_012578m	30S ribosomal protein, putative	GP
cassava4.1_012579m|PACid:17972331	cassava4.1_012579m		No
cassava4.1_012582m|PACid:17980078	cassava4.1_012582m	regulatory particle non-ATPase 13	GP
cassava4.1_012584m|PACid:17963806	cassava4.1_012584m	adenosine monophosphate kinase	No
cassava4.1_012590m|PACid:17985201	cassava4.1_012590m	homeobox-leucine zipper protein 3	No
cassava4.1_012598m|PACid:17966257	cassava4.1_012598m	uncoupling protein 2	No
cassava4.1_012600m|PACid:17990218	cassava4.1_012600m	Pathogenesis-related thaumatin superfamily protein	No
cassava4.1_012602m|PACid:17968883	cassava4.1_012602m	20S proteasome beta subunit G1	GP
cassava4.1_012605m|PACid:17983433	cassava4.1_012605m	2-phosphoglycolate phosphatase 2	G
cassava4.1_012606m|PACid:17965803	cassava4.1_012606m	syntaxin of plants 131	GP

cassava4.1_012607m|PACid:17986896	cassava4.1_012607m	phosphoinositide binding	GP
cassava4.1_012609m|PACid:17966703	cassava4.1_012609m	sterol-4alpha-methyl oxidase 1-1	No
cassava4.1_012610m|PACid:17985006	cassava4.1_012610m	Galactose mutarotase-like superfamily protein	No
cassava4.1_012614m|PACid:17982841	cassava4.1_012614m	DCD (Development and Cell Death) domain protein	G
cassava4.1_012617m|PACid:17977202	cassava4.1_012617m	nucleotide-rhamnose synthase/epimerase-reductase	No
cassava4.1_012618m|PACid:17991164	cassava4.1_012618m	FAD/NAD(P)-binding oxidoreductase	GP
cassava4.1_012619m|PACid:17966189	cassava4.1_012619m	NAD(P)-binding Rossmann-fold superfamily protein	GP
cassava4.1_012623m|PACid:17977939	cassava4.1_012623m	isopentenyl pyrophosphate:dimethylallyl pyrophosphate isomerase 2	GP
cassava4.1_012624m|PACid:17963519	cassava4.1_012624m	geranyl diphosphate synthase 1	G
cassava4.1_012627m|PACid:17966704	cassava4.1_012627m	SNARE associated Golgi protein family	No
cassava4.1_012631m|PACid:17977093	cassava4.1_012631m	Homeodomain-like/winged-helix DNA-binding family protein	No
cassava4.1_012632m|PACid:17980208	cassava4.1_012632m	3\'-5\'-exoribonuclease family protein	No
cassava4.1_012639m|PACid:17976056	cassava4.1_012639m	myo-inositol oxygenase 1	G
cassava4.1_012640m|PACid:17973222	cassava4.1_012640m	Calcineurin-like metallo-phosphoesterase superfamily protein	GP
cassava4.1_012645m|PACid:17993768	cassava4.1_012645m	RING-H2 finger C1A	G
cassava4.1_012650m|PACid:17966266	cassava4.1_012650m	damaged DNA binding;exodeoxyribonuclease IIIs	G
cassava4.1_012656m|PACid:17969864	cassava4.1_012656m	RNA-binding (RRM/RBD/RNP motifs) family protein with retrovirus zinc finger-like domain	G
cassava4.1_012657m|PACid:17987320	cassava4.1_012657m	golgi snare 11	No
cassava4.1_012671m|PACid:17992712	cassava4.1_012671m	P-loop containing nucleoside triphosphate hydrolases superfamily protein	GP
cassava4.1_012681m|PACid:17991740	cassava4.1_012681m		G
cassava4.1_012687m|PACid:17979919	cassava4.1_012687m	Plastid-lipid associated protein PAP / fibrillin family protein	P
cassava4.1_012689m|PACid:17964313	cassava4.1_012689m	NAD(P)-binding Rossmann-fold superfamily protein	G
cassava4.1_012692m|PACid:17986236	cassava4.1_012692m	P-loop containing nucleoside triphosphate hydrolases superfamily protein	G
cassava4.1_012693m|PACid:17961558	cassava4.1_012693m	isopentenyl pyrophosphate:dimethylallyl pyrophosphate isomerase 2	GP
cassava4.1_012700m|PACid:17989328	cassava4.1_012700m	Protein prenylyltransferase superfamily protein	No
cassava4.1_012705m|PACid:17986166	cassava4.1_012705m	transducin family protein / WD-40 repeat family protein	P
cassava4.1_012717m|PACid:17980772	cassava4.1_012717m	telomere repeat binding factor 1	No
cassava4.1_012724m|PACid:17990263	cassava4.1_012724m	5\'-AMP-activated protein kinase beta-2 subunit protein	No
cassava4.1_012725m|PACid:17981473	cassava4.1_012725m	transducin family protein / WD-40 repeat family protein	P
cassava4.1_012726m|PACid:17970757	cassava4.1_012726m	Nuclear transport factor 2 (NTF2) family protein	G
cassava4.1_012728m|PACid:17965811	cassava4.1_012728m	potassium channel tetramerisation domain-containing protein / pentapeptide repeat-containing protein	G
cassava4.1_012731m|PACid:17979369	cassava4.1_012731m	2-oxoglutarate (2OG) and Fe(II)-dependent oxygenase superfamily protein	GP
cassava4.1_012733m|PACid:17961405	cassava4.1_012733m	UDP-3-O-acyl N-acetylglycosamine deacetylase family protein	No
cassava4.1_012734m|PACid:17984938	cassava4.1_012734m	6-phosphogluconate dehydrogenase family protein	No
cassava4.1_012735m|PACid:17973495	cassava4.1_012735m	Plant protein of unknown function (DUF868)	No
cassava4.1_012736m|PACid:17993810	cassava4.1_012736m		G
cassava4.1_012737m|PACid:17979805	cassava4.1_012737m	Protein kinase superfamily protein	No
cassava4.1_012739m|PACid:17967290	cassava4.1_012739m	Putative methyltransferase family protein	No
cassava4.1_012745m|PACid:17984222	cassava4.1_012745m	pyrophosphorylase 6	GP

cassava4.1_012749m|PACid:17988437	cassava4.1_012749m	Protein of unknown function (DUF569)	G
cassava4.1_012752m|PACid:17992918	cassava4.1_012752m	Chaperone DnaJ-domain superfamily protein	No
cassava4.1_012764m|PACid:17985579	cassava4.1_012764m	Dihydrodipicolinate reductase, bacterial/plant	GP
cassava4.1_012766m|PACid:17969813	cassava4.1_012766m	P-loop containing nucleoside triphosphate hydrolases superfamily protein	No
cassava4.1_012773m|PACid:17962223	cassava4.1_012773m	2-oxoglutarate (2OG) and Fe(II)-dependent oxygenase superfamily protein	GP
cassava4.1_012774m|PACid:17984074	cassava4.1_012774m	Protein kinase superfamily protein	No
cassava4.1_012779m|PACid:17967004	cassava4.1_012779m	Protein kinase superfamily protein	No
cassava4.1_012783m|PACid:17981554	cassava4.1_012783m	Amino acid dehydrogenase family protein	P
cassava4.1_012792m|PACid:17965997	cassava4.1_012792m	Plant protein of unknown function (DUF868)	No
cassava4.1_012793m|PACid:17971993	cassava4.1_012793m	Mitochondrial substrate carrier family protein	P
cassava4.1_012795m|PACid:17974013	cassava4.1_012795m	Phosphoglycerate mutase family protein	No
cassava4.1_012796m|PACid:17981653	cassava4.1_012796m	Phenazine biosynthesis PhzC/PhzF protein	GP
cassava4.1_012801m|PACid:17960518	cassava4.1_012801m	chloroplastic drought-induced stress protein of 32 kD	GP
cassava4.1_012806m|PACid:17984607	cassava4.1_012806m	NAD(P)-binding Rossmann-fold superfamily protein	G
cassava4.1_012809m|PACid:17970915	cassava4.1_012809m	Transducin/WD40 repeat-like superfamily protein	No
cassava4.1_012811m|PACid:17976318	cassava4.1_012811m	ureidoglycine aminohydrolase	No
cassava4.1_012812m|PACid:17970142	cassava4.1_012812m	endonuclease 4	No
cassava4.1_012814m|PACid:17974394	cassava4.1_012814m		No
cassava4.1_012828m|PACid:17976175	cassava4.1_012828m	Nucleotide/sugar transporter family protein	G
cassava4.1_012829m|PACid:17978020	cassava4.1_012829m	nucleic acid binding;RNA binding	G
cassava4.1_012834m|PACid:17969024	cassava4.1_012834m	RING/U-box superfamily protein	G
cassava4.1_012844m|PACid:17992923	cassava4.1_012844m	xyloglucan endotransglucosylase/hydrolase 8	G
cassava4.1_012845m|PACid:17963783	cassava4.1_012845m	translocon at the outer envelope membrane of chloroplasts 33	No
cassava4.1_012846m|PACid:17979982	cassava4.1_012846m	ribosomal protein L5 B	No
cassava4.1_012851m|PACid:17969920	cassava4.1_012851m	Abscisic acid-responsive (TB2/DP1, HVA22) family protein	G
cassava4.1_012857m|PACid:17988619	cassava4.1_012857m	RNA-binding (RRM/RBD/RNP motifs) family protein	No
cassava4.1_012862m|PACid:17968283	cassava4.1_012862m	short-chain dehydrogenase-reductase B	GP
cassava4.1_012863m|PACid:17963073	cassava4.1_012863m		GP
cassava4.1_012868m|PACid:17982857	cassava4.1_012868m	Mitochondrial substrate carrier family protein	GP
cassava4.1_012871m|PACid:17985903	cassava4.1_012871m	HOPW1-1-interacting 2	No
cassava4.1_012879m|PACid:17979363	cassava4.1_012879m	Protein kinase superfamily protein	G
cassava4.1_012883m|PACid:17961038	cassava4.1_012883m	phloem protein 2-A15	No
cassava4.1_012887m|PACid:17982891	cassava4.1_012887m	Sec20 family protein	No
cassava4.1_012892m|PACid:17985904	cassava4.1_012892m	HOPW1-1-interacting 2	No
cassava4.1_012893m|PACid:17973858	cassava4.1_012893m	D111/G-patch domain-containing protein	G
cassava4.1_012894m|PACid:17976164	cassava4.1_012894m	beta-hydroxyisobutyryl-CoA hydrolase 1	No
cassava4.1_012896m|PACid:17969883	cassava4.1_012896m	Mog1/PsbP/DUF1795-like photosystem II reaction center PsbP family protein	GP
cassava4.1_012905m|PACid:17974533	cassava4.1_012905m	atypical CYS HIS rich thioredoxin 4	G
cassava4.1_012906m|PACid:17980671	cassava4.1_012906m	ZIM-LIKE 2	No

cassava4.1_012907m|PACid:17988240	cassava4.1_012907m	ABL interactor-like protein 2	G
cassava4.1_012908m|PACid:17967910	cassava4.1_012908m	photosystem II reaction center PSB29 protein	GP
cassava4.1_012911m|PACid:17970239	cassava4.1_012911m	3-hydroxyacyl-CoA dehydrogenase family protein	GP
cassava4.1_012919m|PACid:17969047	cassava4.1_012919m	3-phosphoserine phosphatase	GP
cassava4.1_012920m|PACid:17990305	cassava4.1_012920m	RNA-binding KH domain-containing protein	G
cassava4.1_012921m|PACid:17967856	cassava4.1_012921m	Duplicated homeodomain-like superfamily protein	G
cassava4.1_012923m|PACid:17971205	cassava4.1_012923m	serine acetyltransferase 1;1	G
cassava4.1_012924m|PACid:17968218	cassava4.1_012924m	phloem protein 2-A12	G
cassava4.1_012926m|PACid:17988057	cassava4.1_012926m	nucleolar essential protein-related	G
cassava4.1_012933m|PACid:17990999	cassava4.1_012933m	phosphate transporter 4;5	G
cassava4.1_012936m|PACid:17989890	cassava4.1_012936m	YebC-related	G
cassava4.1_012938m|PACid:17973982	cassava4.1_012938m	Rhodanese/Cell cycle control phosphatase superfamily protein	GP
cassava4.1_012943m|PACid:17960015	cassava4.1_012943m	NAC (No Apical Meristem) domain transcriptional regulator superfamily protein	G
cassava4.1_012947m|PACid:17990052	cassava4.1_012947m	Eukaryotic translation initiation factor 2 subunit 1	No
cassava4.1_012951m|PACid:17972701	cassava4.1_012951m	alpha/beta-Hydrolases superfamily protein	No
cassava4.1_012952m|PACid:17974832	cassava4.1_012952m	RNA-binding KH domain-containing protein	G
cassava4.1_012963m|PACid:17971883	cassava4.1_012963m	Nucleotide-diphospho-sugar transferases superfamily protein	GP
cassava4.1_012969m|PACid:17978577	cassava4.1_012969m	ABI-1-like 1	No
cassava4.1_012973m|PACid:17989798	cassava4.1_012973m	EXORDIUM like 2	No
cassava4.1_012974m|PACid:17988748	cassava4.1_012974m	eukaryotic translation initiation factor 3G1	No
cassava4.1_012982m|PACid:17961183	cassava4.1_012982m	RmlC-like cupins superfamily protein	G
cassava4.1_012986m|PACid:17974424	cassava4.1_012986m	Haloacid dehalogenase-like hydrolase (HAD) superfamily protein	No
cassava4.1_013001m|PACid:17963526	cassava4.1_013001m	IND1(iron-sulfur protein required for NADH dehydrogenase)-like	No
cassava4.1_013002m|PACid:17972730	cassava4.1_013002m	Cyclophilin-like peptidyl-prolyl cis-trans isomerase family protein	GP
cassava4.1_013005m|PACid:17984381	cassava4.1_013005m	Ribosomal RNA processing Brix domain protein	G
cassava4.1_013007m|PACid:17991059	cassava4.1_013007m	zinc finger (C2H2 type, AN1-like) family protein	G
cassava4.1_013011m|PACid:17967272	cassava4.1_013011m	xyloglucan endotransglucosylase/hydrolase 16	GP
cassava4.1_013012m|PACid:17989886	cassava4.1_013012m	ataurora1	G
cassava4.1_013014m|PACid:17977026	cassava4.1_013014m	xyloglucan endotransglucosylase/hydrolase 5	GP
cassava4.1_013015m|PACid:17985023	cassava4.1_013015m	xyloglucan endotransglycosylase 6	No
cassava4.1_013027m|PACid:17991905	cassava4.1_013027m	ribosomal protein L4	GP
cassava4.1_013028m|PACid:17967471	cassava4.1_013028m	Tetratricopeptide repeat (TPR)-like superfamily protein	G
cassava4.1_013030m|PACid:17988542	cassava4.1_013030m		No
cassava4.1_013033m|PACid:17974214	cassava4.1_013033m	RING/FYVE/PHD zinc finger superfamily protein	G
cassava4.1_013035m|PACid:17991234	cassava4.1_013035m	SPX domain gene 2	No
cassava4.1_013037m|PACid:17993697	cassava4.1_013037m	nudix hydrolase homolog 23	No
cassava4.1_013038m|PACid:17986954	cassava4.1_013038m	Protein of unknown function (DUF1295)	No
cassava4.1_013044m|PACid:17973356	cassava4.1_013044m	xyloglucan endotransglucosylase/hydrolase 6	No
cassava4.1_013047m|PACid:17984873	cassava4.1_013047m	small nuclear ribonucleoprotein associated protein B	GP

cassava4.1_013054m|PACid:17984936	cassava4.1_013054m	SPX domain gene 2	G
cassava4.1_013061m|PACid:17977568	cassava4.1_013061m	Haloacid dehalogenase-like hydrolase (HAD) superfamily protein	G
cassava4.1_013076m|PACid:17974814	cassava4.1_013076m	Protein of unknown function (DUF579)	G
cassava4.1_013077m|PACid:17985983	cassava4.1_013077m		GP
cassava4.1_013084m|PACid:17987974	cassava4.1_013084m	Ankyrin repeat family protein	G
cassava4.1_013087m|PACid:17993084	cassava4.1_013087m	glyoxalase II 3	No
cassava4.1_013094m|PACid:17967955	cassava4.1_013094m	S-adenosyl-L-methionine-dependent methyltransferases superfamily protein	No
cassava4.1_013096m|PACid:17968754	cassava4.1_013096m	xyloglucan endotransglucosylase/hydrolase 32	No
cassava4.1_013099m|PACid:17987586	cassava4.1_013099m	5\'-AMP-activated protein kinase-related	No
cassava4.1_013101m|PACid:17969626	cassava4.1_013101m	Pathogenesis-related thaumatin superfamily protein	G
cassava4.1_013103m|PACid:17977057	cassava4.1_013103m	APS kinase	No
cassava4.1_013110m|PACid:17961373	cassava4.1_013110m	Aldolase-type TIM barrel family protein	G
cassava4.1_013114m|PACid:17962616	cassava4.1_013114m	Predicted AT-hook DNA-binding family protein	G
cassava4.1_013118m|PACid:17972949	cassava4.1_013118m	xyloglucan endotransglycosylase 6	No
cassava4.1_013119m|PACid:17977651	cassava4.1_013119m	CLP protease P4	GP
cassava4.1_013120m|PACid:17987362	cassava4.1_013120m	prohibitin 1	P
cassava4.1_013121m|PACid:17981474	cassava4.1_013121m	thiamin pyrophosphokinase1	No
cassava4.1_013125m|PACid:17969757	cassava4.1_013125m	cofactor of nitrate reductase and xanthine dehydrogenase 2	No
cassava4.1_013127m|PACid:17984851	cassava4.1_013127m	Plant haem oxygenase (decyclizing) family protein	GP
cassava4.1_013136m|PACid:17966860	cassava4.1_013136m	S-adenosyl-L-methionine-dependent methyltransferases superfamily protein	GP
cassava4.1_013142m|PACid:17960563	cassava4.1_013142m	2-oxoglutarate (2OG) and Fe(II)-dependent oxygenase superfamily protein	G
cassava4.1_013144m|PACid:17993901	cassava4.1_013144m	Acyl-CoA N-acyltransferases (NAT) superfamily protein	G
cassava4.1_013146m|PACid:17991640	cassava4.1_013146m	glyoxylate reductase 1	GP
cassava4.1_013147m|PACid:17984960	cassava4.1_013147m	Coatomer epsilon subunit	GP
cassava4.1_013150m|PACid:17994004	cassava4.1_013150m	2-oxoglutarate (2OG) and Fe(II)-dependent oxygenase superfamily protein	No
cassava4.1_013151m|PACid:17984789	cassava4.1_013151m	alpha-soluble NSF attachment protein 2	P
cassava4.1_013157m|PACid:17980169	cassava4.1_013157m	alpha/beta-Hydrolases superfamily protein	GP
cassava4.1_013158m|PACid:17968853	cassava4.1_013158m	glyoxalase I homolog	GP
cassava4.1_013163m|PACid:17966002	cassava4.1_013163m	Ribosomal protein L3 family protein	GP
cassava4.1_013165m|PACid:17985022	cassava4.1_013165m	xyloglucan endotransglycosylase 6	No
cassava4.1_013176m|PACid:17986658	cassava4.1_013176m	Sec14p-like phosphatidylinositol transfer family protein	No
cassava4.1_013177m|PACid:17978863	cassava4.1_013177m	homeobox protein 2	G
cassava4.1_013178m|PACid:17985489	cassava4.1_013178m	histidine biosynthesis bifunctional protein (HISIE)	GP
cassava4.1_013180m|PACid:17967168	cassava4.1_013180m	Rieske (2Fe-2S) domain-containing protein	No
cassava4.1_013189m|PACid:17981404	cassava4.1_013189m	ascorbate peroxidase 3	No
cassava4.1_013191m|PACid:17962437	cassava4.1_013191m	senescence associated gene 18	G
cassava4.1_013192m|PACid:17971433	cassava4.1_013192m	plasma membrane intrinsic protein 2	P
cassava4.1_013198m|PACid:17977441	cassava4.1_013198m	P-loop containing nucleoside triphosphate hydrolases superfamily protein	GP
cassava4.1_013199m|PACid:17992917	cassava4.1_013199m	tobamovirus multiplication 1	No

cassava4.1_013209m|PACid:17969303	cassava4.1_013209m	Protein of unknown function (DUF1218)	G
cassava4.1_013213m|PACid:17982528	cassava4.1_013213m	S-adenosyl-L-methionine-dependent methyltransferases superfamily protein	GP
cassava4.1_013216m|PACid:17985689	cassava4.1_013216m	NAD(P)-binding Rossmann-fold superfamily protein	P
cassava4.1_013217m|PACid:17966655	cassava4.1_013217m		G
cassava4.1_013221m|PACid:17974194	cassava4.1_013221m	SPFH/Band 7/PHB domain-containing membrane-associated protein family	No
cassava4.1_013224m|PACid:17988932	cassava4.1_013224m	eukaryotic translation initiation factor 2	No
cassava4.1_013229m|PACid:17961789	cassava4.1_013229m	Plastid-lipid associated protein PAP / fibrillin family protein	GP
cassava4.1_013232m|PACid:17972564	cassava4.1_013232m	adenosine kinase	No
cassava4.1_013235m|PACid:17962306	cassava4.1_013235m	NAD(P)-binding Rossmann-fold superfamily protein	GP
cassava4.1_013239m|PACid:17971730	cassava4.1_013239m	U2 small nuclear ribonucleoprotein A	GP
cassava4.1_013241m|PACid:17977992	cassava4.1_013241m	Uncharacterized conserved protein (DUF2358)	G
cassava4.1_013243m|PACid:17983975	cassava4.1_013243m	plasma membrane intrinsic protein 1;4	P
cassava4.1_013244m|PACid:17982776	cassava4.1_013244m	uracil phosphoribosyltransferase	GP
cassava4.1_013249m|PACid:17982889	cassava4.1_013249m	3\'-5\'-exoribonuclease family protein	G
cassava4.1_013250m|PACid:17975053	cassava4.1_013250m	Putative lysine decarboxylase family protein	No
cassava4.1_013254m|PACid:17976142	cassava4.1_013254m	ACT domain-containing protein	GP
cassava4.1_013263m|PACid:17968451	cassava4.1_013263m	non-intrinsic ABC protein 14	GP
cassava4.1_013274m|PACid:17988998	cassava4.1_013274m	casein kinase II beta chain 1	G
cassava4.1_013275m|PACid:17979937	cassava4.1_013275m	Chaperone DnaJ-domain superfamily protein	No
cassava4.1_013281m|PACid:17989399	cassava4.1_013281m	Ribose 5-phosphate isomerase, type A protein	GP
cassava4.1_013284m|PACid:17980861	cassava4.1_013284m	plasma membrane intrinsic protein 2;4	GP
cassava4.1_013285m|PACid:17988328	cassava4.1_013285m	Homeobox-leucine zipper protein family	G
cassava4.1_013286m|PACid:17993141	cassava4.1_013286m	Xyloglucan endotransglucosylase/hydrolase family protein	GP
cassava4.1_013291m|PACid:17972091	cassava4.1_013291m	xyloglucan endotransglycosylase 6	P
cassava4.1_013294m|PACid:17980352	cassava4.1_013294m	light harvesting complex photosystem II	GP
cassava4.1_013299m|PACid:17960228	cassava4.1_013299m	Translin family protein	G
cassava4.1_013300m|PACid:17963500	cassava4.1_013300m	RNA-binding (RRM/RBD/RNP motifs) family protein	G
cassava4.1_013301m|PACid:17962617	cassava4.1_013301m	U2 snRNP auxiliary factor small subunit, putative	G
cassava4.1_013313m|PACid:17964054	cassava4.1_013313m		No
cassava4.1_013314m|PACid:17983214	cassava4.1_013314m	dual specificity protein phosphatase (DsPTP1) family protein	GP
cassava4.1_013316m|PACid:17988375	cassava4.1_013316m	SPFH/Band 7/PHB domain-containing membrane-associated protein family	No
cassava4.1_013325m|PACid:17977249	cassava4.1_013325m	Transcription initiation factor TFIIE, beta subunit	No
cassava4.1_013328m|PACid:17968717	cassava4.1_013328m	plasma membrane intrinsic protein 2;5	P
cassava4.1_013331m|PACid:17966333	cassava4.1_013331m	DNA glycosylase superfamily protein	G
cassava4.1_013332m|PACid:17988443	cassava4.1_013332m	Phenazine biosynthesis PhzC/PhzF protein	P
cassava4.1_013335m|PACid:17983341	cassava4.1_013335m	post-illumination chlorophyll fluorescence increase	GP
cassava4.1_013341m|PACid:17974047	cassava4.1_013341m	5-formyltetrahydrofolate cycloligase	No
cassava4.1_013342m|PACid:17970215	cassava4.1_013342m	xyloglucan endotransglycosylase 6	No
cassava4.1_013344m|PACid:17961195	cassava4.1_013344m	2-oxoglutarate (2OG) and Fe(II)-dependent oxygenase superfamily protein	G

cassava4.1_013346m|PACid:17975885	cassava4.1_013346m	DHHC-type zinc finger family protein	No
cassava4.1_013351m|PACid:17990548	cassava4.1_013351m	S-formylglutathione hydrolase	GP
cassava4.1_013355m|PACid:17965842	cassava4.1_013355m		G
cassava4.1_013356m|PACid:17982090	cassava4.1_013356m	Calcium-binding EF-hand family protein	GP
cassava4.1_013358m|PACid:17980407	cassava4.1_013358m	xyloglucan endotransglucosylase/hydrolase 32	No
cassava4.1_013362m|PACid:17985142	cassava4.1_013362m	succinate dehydrogenase 2-2	GP
cassava4.1_013368m|PACid:17977911	cassava4.1_013368m	chloroplastic acetylcoenzyme A carboxylase 1	GP
cassava4.1_013370m|PACid:17977393	cassava4.1_013370m	Phosphoglycerate mutase family protein	No
cassava4.1_013373m|PACid:17971794	cassava4.1_013373m	Protein phosphatase 2C family protein	P
cassava4.1_013378m|PACid:17969696	cassava4.1_013378m	phytanoyl-CoA dioxygenase (PhyH) family protein	GP
cassava4.1_013383m|PACid:17988546	cassava4.1_013383m	Protein phosphatase 2C family protein	GP
cassava4.1_013384m|PACid:17986980	cassava4.1_013384m	carboxyl terminus of HSC70-interacting protein	G
cassava4.1_013391m|PACid:17974472	cassava4.1_013391m	expansin A13	GP
cassava4.1_013393m|PACid:17973744	cassava4.1_013393m	tetraspanin6	No
cassava4.1_013394m|PACid:17971658	cassava4.1_013394m	PPPDE putative thiol peptidase family protein	No
cassava4.1_013395m|PACid:17978972	cassava4.1_013395m	Biotin/lipoate A/B protein ligase family	No
cassava4.1_013399m|PACid:17979855	cassava4.1_013399m	Protein phosphatase 2C family protein	GP
cassava4.1_013400m|PACid:17988462	cassava4.1_013400m	origin recognition complex protein 6	No
cassava4.1_013405m|PACid:17960415	cassava4.1_013405m	ATP-dependent protease La (LON) domain protein	GP
cassava4.1_013407m|PACid:17988424	cassava4.1_013407m	ssDNA-binding transcriptional regulator	GP
cassava4.1_013411m|PACid:17966350	cassava4.1_013411m	YEATS family protein	No
cassava4.1_013419m|PACid:17987521	cassava4.1_013419m	NAD(P)-binding Rossmann-fold superfamily protein	GP
cassava4.1_013422m|PACid:17981056	cassava4.1_013422m	Class I glutamine amidotransferase-like superfamily protein	No
cassava4.1_013425m|PACid:17991927	cassava4.1_013425m	NAD(P)-binding Rossmann-fold superfamily protein	No
cassava4.1_013428m|PACid:17984644	cassava4.1_013428m	clast3-related	GP
cassava4.1_013435m|PACid:17990851	cassava4.1_013435m	bifunctional nuclease i	G
cassava4.1_013439m|PACid:17966057	cassava4.1_013439m	RNA-binding KH domain-containing protein	No
cassava4.1_013441m|PACid:17962362	cassava4.1_013441m	uridylyltransferase-related	GP
cassava4.1_013442m|PACid:17970748	cassava4.1_013442m	Nucleoside diphosphate kinase family protein	GP
cassava4.1_013450m|PACid:17970523	cassava4.1_013450m	conserved oligomeric Golgi complex component-related / COG complex component-related	No
cassava4.1_013454m|PACid:17962230	cassava4.1_013454m	hydroxyethylthiazole kinase family protein	No
cassava4.1_013462m|PACid:17972228	cassava4.1_013462m	eukaryotic translation initiation factor SUI1 family protein	G
cassava4.1_013463m|PACid:17980419	cassava4.1_013463m	chloroplast RNA-binding protein 29	G
cassava4.1_013467m|PACid:17963196	cassava4.1_013467m	NAC-like, activated by AP3/PI	No
cassava4.1_013469m|PACid:17974028	cassava4.1_013469m	D-ribulose-5-phosphate-3-epimerase	GP
cassava4.1_013472m|PACid:17968338	cassava4.1_013472m	non-intrinsic ABC protein 4	No
cassava4.1_013475m|PACid:17965961	cassava4.1_013475m	NADH:cytochrome B5 reductase 1	GP
cassava4.1_013476m|PACid:17976819	cassava4.1_013476m	Polynucleotidyl transferase, ribonuclease H-like superfamily protein	G
cassava4.1_013482m|PACid:17990816	cassava4.1_013482m		G

cassava4.1_013484m|PACid:17977310	cassava4.1_013484m		No
cassava4.1_013485m|PACid:17970029	cassava4.1_013485m	nudix hydrolase homolog 15	G
cassava4.1_013486m|PACid:17973597	cassava4.1_013486m	Ribosomal protein L18e/L15 superfamily protein	No
cassava4.1_013489m|PACid:17972090	cassava4.1_013489m	xyloglucan endotransglycosylase 6	No
cassava4.1_013497m|PACid:17987077	cassava4.1_013497m	S-adenosyl-L-methionine-dependent methyltransferases superfamily protein	No
cassava4.1_013502m|PACid:17990613	cassava4.1_013502m	Arp2/3 complex, 34 kD subunit p34-Arc	No
cassava4.1_013503m|PACid:17991592	cassava4.1_013503m	Duplicated homeodomain-like superfamily protein	G
cassava4.1_013507m|PACid:17980276	cassava4.1_013507m	C2 domain-containing protein / GRAM domain-containing protein	No
cassava4.1_013511m|PACid:17992152	cassava4.1_013511m	lumazine-binding family protein	GP
cassava4.1_013514m|PACid:17963882	cassava4.1_013514m	CHY-type/CTCHY-type/RING-type Zinc finger protein	No
cassava4.1_013518m|PACid:17960210	cassava4.1_013518m	casein kinase II beta chain 2	G
cassava4.1_013524m|PACid:17985118	cassava4.1_013524m	prohibitin 3	GP
cassava4.1_013526m|PACid:17976454	cassava4.1_013526m	Pentapeptide repeat-containing protein	GP
cassava4.1_013531m|PACid:17979203	cassava4.1_013531m	Protein of unknown function (DUF3353)	No
cassava4.1_013536m|PACid:17971398	cassava4.1_013536m	SNARE associated Golgi protein family	G
cassava4.1_013537m|PACid:17966700	cassava4.1_013537m	CLP protease proteolytic subunit 2	GP
cassava4.1_013540m|PACid:17969556	cassava4.1_013540m	tobamovirus multiplication 2A	No
cassava4.1_013550m|PACid:17986045	cassava4.1_013550m	Fe superoxide dismutase 3	GP
cassava4.1_013553m|PACid:17970491	cassava4.1_013553m	RNA-binding (RRM/RBD/RNP motifs) family protein	No
cassava4.1_013559m|PACid:17989174	cassava4.1_013559m	Integral membrane Yip1 family protein	G
cassava4.1_013563m|PACid:17989418	cassava4.1_013563m	Acyl-CoA N-acyltransferases (NAT) superfamily protein	G
cassava4.1_013570m|PACid:17961432	cassava4.1_013570m	anthranilate synthase beta subunit 1	GP
cassava4.1_013573m|PACid:17973244	cassava4.1_013573m	Metallo-hydrolase/oxidoreductase superfamily protein	No
cassava4.1_013587m|PACid:17994060	cassava4.1_013587m	Ribosomal protein L22p/L17e family protein	No
cassava4.1_013590m|PACid:17966436	cassava4.1_013590m	transducin family protein / WD-40 repeat family protein	No
cassava4.1_013592m|PACid:17979405	cassava4.1_013592m	Phospholipid/glycerol acyltransferase family protein	G
cassava4.1_013602m|PACid:17978003	cassava4.1_013602m	mitotic checkpoint family protein	No
cassava4.1_013608m|PACid:17973077	cassava4.1_013608m	NAD(P)-binding Rossmann-fold superfamily protein	GP
cassava4.1_013609m|PACid:17975077	cassava4.1_013609m	RmlC-like cupins superfamily protein	G
cassava4.1_013610m|PACid:17992003	cassava4.1_013610m	Integral membrane Yip1 family protein	G
cassava4.1_013612m|PACid:17971157	cassava4.1_013612m	S-adenosyl-L-methionine-dependent methyltransferases superfamily protein	G
cassava4.1_013616m|PACid:17969403	cassava4.1_013616m	Protein phosphatase 2C family protein	G
cassava4.1_013617m|PACid:17993769	cassava4.1_013617m	Peroxisomal membrane 22 kDa (Mpv17/PMP22) family protein	GP
cassava4.1_013619m|PACid:17988464	cassava4.1_013619m	voltage dependent anion channel 1	GP
cassava4.1_013623m|PACid:17971302	cassava4.1_013623m	ZIP metal ion transporter family	No
cassava4.1_013625m|PACid:17990538	cassava4.1_013625m	Nucleotide-sugar transporter family protein	No
cassava4.1_013630m|PACid:17975897	cassava4.1_013630m	Family of unknown function (DUF572)	No
cassava4.1_013633m|PACid:17964644	cassava4.1_013633m	Chitinase family protein	No
cassava4.1_013634m|PACid:17960178	cassava4.1_013634m	appr-1-p processing enzyme family protein	No

cassava4.1_013640m|PACid:17968292	cassava4.1_013640m	Per1-like family protein	No
cassava4.1_013641m|PACid:17986163	cassava4.1_013641m	ribosome recycling factor, chloroplast precursor	GP
cassava4.1_013646m|PACid:17972597	cassava4.1_013646m	TRAM, LAG1 and CLN8 (TLC) lipid-sensing domain containing protein	G
cassava4.1_013649m|PACid:17989348	cassava4.1_013649m	phosphoenolpyruvate carboxylase kinase 2	G
cassava4.1_013651m|PACid:17992821	cassava4.1_013651m	associated molecule with the SH3 domain of STAM 2	No
cassava4.1_013665m|PACid:17974406	cassava4.1_013665m	pyrroline-5- carboxylate (P5C) reductase	GP
cassava4.1_013669m|PACid:17961600	cassava4.1_013669m	delta(3,5),delta(2,4)-dienoyl-CoA isomerase 1	GP
cassava4.1_013671m|PACid:17986923	cassava4.1_013671m	Glycolipid transfer protein (GLTP) family protein	GP
cassava4.1_013684m|PACid:17991633	cassava4.1_013684m	photosystem I light harvesting complex gene 3	GP
cassava4.1_013686m|PACid:17983149	cassava4.1_013686m	phosphoribosylanthranilate isomerase 1	G
cassava4.1_013687m|PACid:17974445	cassava4.1_013687m	HISTIDINE BIOSYNTHESIS 5B	No
cassava4.1_013689m|PACid:17970912	cassava4.1_013689m	Syntaxin/t-SNARE family protein	No
cassava4.1_013693m|PACid:17960134	cassava4.1_013693m	Cytokine-induced anti-apoptosis inhibitor 1, Fe-S biogenesis	GP
cassava4.1_013694m|PACid:17985760	cassava4.1_013694m	Polynucleotidyl transferase, ribonuclease H-like superfamily protein	G
cassava4.1_013697m|PACid:17977846	cassava4.1_013697m	Ubiquinol-cytochrome C reductase iron-sulfur subunit	GP
cassava4.1_013711m|PACid:17969271	cassava4.1_013711m	rubredoxin family protein	No
cassava4.1_013713m|PACid:17977326	cassava4.1_013713m	Tic22-like family protein	No
cassava4.1_013714m|PACid:17989165	cassava4.1_013714m	light harvesting complex photosystem II	GP
cassava4.1_013716m|PACid:17963083	cassava4.1_013716m	peptide deformylase 1B	GP
cassava4.1_013717m|PACid:17975830	cassava4.1_013717m	voltage dependent anion channel 1	GP
cassava4.1_013725m|PACid:17965236	cassava4.1_013725m	PQ-loop repeat family protein / transmembrane family protein	G
cassava4.1_013726m|PACid:17970171	cassava4.1_013726m		No
cassava4.1_013728m|PACid:17988456	cassava4.1_013728m	RING/FYVE/PHD zinc finger superfamily protein	No
cassava4.1_013729m|PACid:17975313	cassava4.1_013729m	Polynucleotidyl transferase, ribonuclease H-like superfamily protein	G
cassava4.1_013734m|PACid:17989591	cassava4.1_013734m	sterol 1	G
cassava4.1_013735m|PACid:17960921	cassava4.1_013735m	Chlorophyll A-B binding family protein	GP
cassava4.1_013737m|PACid:17987042	cassava4.1_013737m	Cyclase family protein	GP
cassava4.1_013738m|PACid:17983503	cassava4.1_013738m	homolog of carrot EP3-3 chitinase	G
cassava4.1_013740m|PACid:17989371	cassava4.1_013740m	peroxin-12	G
cassava4.1_013747m|PACid:17982538	cassava4.1_013747m	N-terminal nucleophile aminohydrolases (Ntn hydrolases) superfamily protein	No
cassava4.1_013751m|PACid:17969124	cassava4.1_013751m	CLP protease proteolytic subunit 6	GP
cassava4.1_013752m|PACid:17982043	cassava4.1_013752m	Thymidine kinase	No
cassava4.1_013756m|PACid:17963530	cassava4.1_013756m	Aldolase-type TIM barrel family protein	P
cassava4.1_013757m|PACid:17966565	cassava4.1_013757m	peroxin 22	GP
cassava4.1_013758m|PACid:17965469	cassava4.1_013758m	nudix hydrolase homolog 3	GP
cassava4.1_013761m|PACid:17986653	cassava4.1_013761m	proteasome alpha subunit F1	GP
cassava4.1_013769m|PACid:17977501	cassava4.1_013769m	Basic-leucine zipper (bZIP) transcription factor family protein	G
cassava4.1_013770m|PACid:17986076	cassava4.1_013770m	ER lumen protein retaining receptor family protein	No
cassava4.1_013772m|PACid:17988880	cassava4.1_013772m	NAD(P)-binding Rossmann-fold superfamily protein	P

cassava4.1_013773m|PACid:17979430	cassava4.1_013773m	dehydroascorbate reductase 1	GP
cassava4.1_013774m|PACid:17989956	cassava4.1_013774m	PPPDE putative thiol peptidase family protein	G
cassava4.1_013778m|PACid:17966182	cassava4.1_013778m	EXS (ERD1/XPR1/SYG1) family protein	No
cassava4.1_013787m|PACid:17981006	cassava4.1_013787m	ER lumen protein retaining receptor family protein	No
cassava4.1_013789m|PACid:17972247	cassava4.1_013789m	enzyme binding;tetrapyrrole binding	GP
cassava4.1_013790m|PACid:17959832	cassava4.1_013790m	UDP-N-acetylglucosamine (UAA) transporter family	No
cassava4.1_013791m|PACid:17970268	cassava4.1_013791m	tetraspanin8	GP
cassava4.1_013792m|PACid:17975631	cassava4.1_013792m		GP
cassava4.1_013793m|PACid:17969071	cassava4.1_013793m	microtubule end binding protein EB1A	G
cassava4.1_013800m|PACid:17990171	cassava4.1_013800m	ER lumen protein retaining receptor family protein	No
cassava4.1_013803m|PACid:17976354	cassava4.1_013803m	tRNA/rRNA methyltransferase (SpoU) family protein	G
cassava4.1_013810m|PACid:17990454	cassava4.1_013810m	indole-3-butyric acid response 5	No
cassava4.1_013814m|PACid:17982853	cassava4.1_013814m	non-intrinsic ABC protein 3	No
cassava4.1_013816m|PACid:17984574	cassava4.1_013816m	receptor like protein 44	No
cassava4.1_013820m|PACid:17982034	cassava4.1_013820m	ribose-5-phosphate isomerase 2	G
cassava4.1_013821m|PACid:17991878	cassava4.1_013821m	sterol 4-alpha-methyl-oxidase 2-1	No
cassava4.1_013823m|PACid:17967217	cassava4.1_013823m	gamma carbonic anhydrase 1	GP
cassava4.1_013826m|PACid:17960683	cassava4.1_013826m	Integral membrane Yip1 family protein	G
cassava4.1_013829m|PACid:17976146	cassava4.1_013829m	UDP-Glycosyltransferase superfamily protein	G
cassava4.1_013832m|PACid:17969167	cassava4.1_013832m	ribosomal protein L15	GP
cassava4.1_013836m|PACid:17992406	cassava4.1_013836m	GNS1/SUR4 membrane protein family	G
cassava4.1_013839m|PACid:17986672	cassava4.1_013839m	gamma carbonic anhydrase 1	P
cassava4.1_013842m|PACid:17989526	cassava4.1_013842m	SC35-like splicing factor 30	GP
cassava4.1_013844m|PACid:17965033	cassava4.1_013844m	Fe superoxide dismutase 2	No
cassava4.1_013847m|PACid:17974514	cassava4.1_013847m	NAD(P)-binding Rossmann-fold superfamily protein	No
cassava4.1_013852m|PACid:17967021	cassava4.1_013852m	alpha/beta-Hydrolases superfamily protein	G
cassava4.1_013855m|PACid:17963607	cassava4.1_013855m	Cystathionine beta-synthase (CBS) family protein	GP
cassava4.1_013856m|PACid:17980420	cassava4.1_013856m	chloroplast RNA-binding protein 29	No
cassava4.1_013865m|PACid:17972469	cassava4.1_013865m	SMAD/FHA domain-containing protein	No
cassava4.1_013868m|PACid:17964068	cassava4.1_013868m	eukaryotic translation initiation factor 4E	No
cassava4.1_013871m|PACid:17963854	cassava4.1_013871m	eukaryotic translation initiation factor 2 beta subunit	No
cassava4.1_013872m|PACid:17984511	cassava4.1_013872m	Mog1/PsbP/DUF1795-like photosystem II reaction center PsbP family protein	GP
cassava4.1_013875m|PACid:17968047	cassava4.1_013875m	Inositol monophosphatase family protein	GP
cassava4.1_013879m|PACid:17977810	cassava4.1_013879m	NAD-dependent epimerase/dehydratase family protein	No
cassava4.1_013883m|PACid:17973890	cassava4.1_013883m	Photosystem II reaction center PsbP family protein	No
cassava4.1_013884m|PACid:17965645	cassava4.1_013884m	RNA-binding (RRM/RBD/RNP motifs) family protein	GP
cassava4.1_013886m|PACid:17974671	cassava4.1_013886m	Integrase-type DNA-binding superfamily protein	G
cassava4.1_013890m|PACid:17991452	cassava4.1_013890m	RHOMBOID-like protein 13	No
cassava4.1_013893m|PACid:17965582	cassava4.1_013893m	Syntaxin/t-SNARE family protein	G

cassava4.1_013895m|PACid:17961699	cassava4.1_013895m	Phosphoglycerate mutase family protein	No
cassava4.1_013903m|PACid:17983431	cassava4.1_013903m	Integral membrane HRF1 family protein	G
cassava4.1_013904m|PACid:17990779	cassava4.1_013904m	alpha/beta-Hydrolases superfamily protein	GP
cassava4.1_013907m|PACid:17971448	cassava4.1_013907m	CHY-type/CTCHY-type/RING-type Zinc finger protein	No
cassava4.1_013908m|PACid:17980382	cassava4.1_013908m	novel plant snare 13	GP
cassava4.1_013912m|PACid:17981697	cassava4.1_013912m	zinc finger (C3HC4-type RING finger) family protein	G
cassava4.1_013914m|PACid:17980166	cassava4.1_013914m	ubiquitin-conjugating enzyme 22	G
cassava4.1_013917m|PACid:17986369	cassava4.1_013917m	Josephin family protein	G
cassava4.1_013921m|PACid:17992320	cassava4.1_013921m	2-cysteine peroxiredoxin B	GP
cassava4.1_013926m|PACid:17971278	cassava4.1_013926m	Ribosomal protein S5 family protein	No
cassava4.1_013930m|PACid:17983105	cassava4.1_013930m		G
cassava4.1_013954m|PACid:17967654	cassava4.1_013954m	Homeobox-leucine zipper protein family	No
cassava4.1_013955m|PACid:17964590	cassava4.1_013955m	HAD superfamily, subfamily IIIB acid phosphatase	G
cassava4.1_013956m|PACid:17966034	cassava4.1_013956m	NAD(P)-binding Rossmann-fold superfamily protein	GP
cassava4.1_013957m|PACid:17977913	cassava4.1_013957m	photosystem I light harvesting complex gene 2	G
cassava4.1_013959m|PACid:17990760	cassava4.1_013959m	Stress responsive alpha-beta barrel domain protein	GP
cassava4.1_013965m|PACid:17985009	cassava4.1_013965m	BTB/POZ domain-containing protein	G
cassava4.1_013978m|PACid:17985672	cassava4.1_013978m	ferritin 4	GP
cassava4.1_013980m|PACid:17981260	cassava4.1_013980m	photosystem I light harvesting complex gene 5	G
cassava4.1_013984m|PACid:17980530	cassava4.1_013984m	regulatory particle non-ATPase 12A	GP
cassava4.1_013986m|PACid:17973349	cassava4.1_013986m	Homeobox-leucine zipper protein family	G
cassava4.1_013990m|PACid:17971470	cassava4.1_013990m	alanine-tRNA ligases;nucleic acid binding;ligases, forming aminoacyl-tRNA and related compounds	G
cassava4.1_013992m|PACid:17991377	cassava4.1_013992m	Glutathione S-transferase family protein	G
cassava4.1_013997m|PACid:17966578	cassava4.1_013997m	Predicted pyridoxal phosphate-dependent enzyme, YBL036C type	GP
cassava4.1_013998m|PACid:17991890	cassava4.1_013998m	proliferating cell nuclear antigen 2	P
cassava4.1_013999m|PACid:17992708	cassava4.1_013999m	alpha/beta-Hydrolases superfamily protein	G
cassava4.1_014000m|PACid:17985774	cassava4.1_014000m	Protein of unknown function (DUF502)	G
cassava4.1_014002m|PACid:17975942	cassava4.1_014002m	Phospholipid/glycerol acyltransferase family protein	No
cassava4.1_014004m|PACid:17975393	cassava4.1_014004m	6-phosphogluconolactonase 1	No
cassava4.1_014009m|PACid:17977557	cassava4.1_014009m	FASCICLIN-like arabinoogalactan 7	GP
cassava4.1_014012m|PACid:17988787	cassava4.1_014012m	ELMO/CED-12 family protein	No
cassava4.1_014018m|PACid:17981101	cassava4.1_014018m	peptide deformylase 1A	GP
cassava4.1_014019m|PACid:17989773	cassava4.1_014019m	cytochrome c oxidase assembly protein CtaG / Cox11 family	G
cassava4.1_014024m|PACid:17968271	cassava4.1_014024m	nuclear factor Y, subunit C2	G
cassava4.1_014026m|PACid:17971589	cassava4.1_014026m	Tetratricopeptide repeat (TPR)-like superfamily protein	No
cassava4.1_014032m|PACid:17976662	cassava4.1_014032m	syntaxin of plants 71	P
cassava4.1_014036m|PACid:17960198	cassava4.1_014036m	enoyl-CoA hydratase/isomerase A	GP
cassava4.1_014040m|PACid:17964052	cassava4.1_014040m	light-harvesting chlorophyll-protein complex II subunit B1	GP
cassava4.1_014041m|PACid:17974534	cassava4.1_014041m	PHD finger protein-related	No

cassava4.1_014045m|PACid:17986998	cassava4.1_014045m	RAB GTPase homolog A5E	P
cassava4.1_014047m|PACid:17993718	cassava4.1_014047m	D111/G-patch domain-containing protein	G
cassava4.1_014049m|PACid:17967920	cassava4.1_014049m	catalytic LigB subunit of aromatic ring-opening dioxygenase family	G
cassava4.1_014050m|PACid:17983338	cassava4.1_014050m	phosphatidic acid phosphatase-related / PAP2-related	No
cassava4.1_014051m|PACid:17968924	cassava4.1_014051m	NAP1-related protein 2	GP
cassava4.1_014055m|PACid:17967728	cassava4.1_014055m	photosystem II light harvesting complex gene 2.1	No
cassava4.1_014058m|PACid:17973125	cassava4.1_014058m	Glutaredoxin family protein	G
cassava4.1_014066m|PACid:17992153	cassava4.1_014066m	expansin B3	G
cassava4.1_014069m|PACid:17979309	cassava4.1_014069m	RNA-binding (RRM/RBD/RNP motifs) family protein	G
cassava4.1_014072m|PACid:17961833	cassava4.1_014072m	Inosine triphosphate pyrophosphatase family protein	GP
cassava4.1_014074m|PACid:17970527	cassava4.1_014074m	Duplicated homeodomain-like superfamily protein	No
cassava4.1_014075m|PACid:17973372	cassava4.1_014075m	general regulatory factor 12	No
cassava4.1_014082m|PACid:17966508	cassava4.1_014082m	2-oxoglutarate (2OG) and Fe(II)-dependent oxygenase superfamily protein	G
cassava4.1_014088m|PACid:17977183	cassava4.1_014088m	tetraspanin10	G
cassava4.1_014094m|PACid:17978486	cassava4.1_014094m	Ribosomal L5P family protein	GP
cassava4.1_014107m|PACid:17972530	cassava4.1_014107m	tonneau 1b (TON1b)	No
cassava4.1_014114m|PACid:17960296	cassava4.1_014114m	photosystem I light harvesting complex gene 6	G
cassava4.1_014116m|PACid:17978968	cassava4.1_014116m	peroxin 11A	G
cassava4.1_014117m|PACid:17979760	cassava4.1_014117m	light-harvesting chlorophyll B-binding protein 3	GP
cassava4.1_014119m|PACid:17962736	cassava4.1_014119m	Metal-dependent phosphohydrolase	GP
cassava4.1_014129m|PACid:17986773	cassava4.1_014129m	AGAMOUS-like 26	No
cassava4.1_014132m|PACid:17988421	cassava4.1_014132m	sphingoid base hydroxylase 2	No
cassava4.1_014138m|PACid:17989357	cassava4.1_014138m	Ribosomal protein S3Ae	P
cassava4.1_014140m|PACid:17972034	cassava4.1_014140m	Ribosomal protein S4 (RPS4A) family protein	No
cassava4.1_014141m|PACid:17983785	cassava4.1_014141m	acyl-activating enzyme 18	No
cassava4.1_014142m|PACid:17963460	cassava4.1_014142m	S-adenosyl-L-methionine-dependent methyltransferases superfamily protein	G
cassava4.1_014149m|PACid:17986622	cassava4.1_014149m	RNA-binding (RRM/RBD/RNP motifs) family protein	No
cassava4.1_014151m|PACid:17963296	cassava4.1_014151m	SNARE associated Golgi protein family	G
cassava4.1_014157m|PACid:17969676	cassava4.1_014157m	Transcription initiation factor IIF, beta subunit	G
cassava4.1_014159m|PACid:17984558	cassava4.1_014159m	P-loop containing nucleoside triphosphate hydrolases superfamily protein	No
cassava4.1_014161m|PACid:17981034	cassava4.1_014161m	methylthioadenosine nucleosidase 1	GP
cassava4.1_014162m|PACid:17976638	cassava4.1_014162m	SPla/RYanodine receptor (SPRY) domain-containing protein	GP
cassava4.1_014164m|PACid:17961859	cassava4.1_014164m	Haloacid dehalogenase-like hydrolase (HAD) superfamily protein	No
cassava4.1_014166m|PACid:17992875	cassava4.1_014166m	peptidoglycan-binding LysM domain-containing protein	G
cassava4.1_014167m|PACid:17964557	cassava4.1_014167m	peptide met sulfoxide reductase 4	GP
cassava4.1_014168m|PACid:17983220	cassava4.1_014168m	ferritin 4	No
cassava4.1_014169m|PACid:17976633	cassava4.1_014169m	basic helix-loop-helix (bHLH) DNA-binding superfamily protein	No
cassava4.1_014170m|PACid:17961209	cassava4.1_014170m	Mog1/PsbP/DUF1795-like photosystem II reaction center PsbP family protein	GP
cassava4.1_014171m|PACid:17967328	cassava4.1_014171m	Amidase family protein	GP

cassava4.1_014174m|PACid:17983250	cassava4.1_014174m	biotin/lipoyl attachment domain-containing protein	No
cassava4.1_014175m|PACid:17992996	cassava4.1_014175m	NAD(P)-binding Rossmann-fold superfamily protein	No
cassava4.1_014178m|PACid:17962249	cassava4.1_014178m	homolog of human DNA ligase iv-binding protein XRCC4	No
cassava4.1_014185m|PACid:17964106	cassava4.1_014185m	ferritin 2	GP
cassava4.1_014189m|PACid:17985589	cassava4.1_014189m	vacuolar ATP synthase subunit D (VATD) / V-ATPase D subunit / vacuolar proton pump D subunit (VATPD)	GP
cassava4.1_014201m|PACid:17985273	cassava4.1_014201m	peptide met sulfoxide reductase 4	GP
cassava4.1_014206m|PACid:17985602	cassava4.1_014206m	Ribosomal protein L2 family	GP
cassava4.1_014211m|PACid:17974226	cassava4.1_014211m	Leucine-rich repeat (LRR) family protein	GP
cassava4.1_014212m|PACid:17972927	cassava4.1_014212m	Sec14p-like phosphatidylinositol transfer family protein	No
cassava4.1_014213m|PACid:17991797	cassava4.1_014213m	Ribosomal protein L2 family	No
cassava4.1_014216m|PACid:17993099	cassava4.1_014216m	photosystem II subunit P-1	GP
cassava4.1_014220m|PACid:17967240	cassava4.1_014220m	Mitochondrial import inner membrane translocase subunit Tim17/Tim22/Tim23 family protein	GP
cassava4.1_014223m|PACid:17971842	cassava4.1_014223m	general regulatory factor 2	GP
cassava4.1_014226m|PACid:17983289	cassava4.1_014226m	Ribosomal protein S8e family protein	G
cassava4.1_014227m|PACid:17977598	cassava4.1_014227m	Thioredoxin superfamily protein	G
cassava4.1_014230m|PACid:17979765	cassava4.1_014230m	membrane-associated mannitol-induced	No
cassava4.1_014235m|PACid:17987252	cassava4.1_014235m	general regulatory factor 9	GP
cassava4.1_014237m|PACid:17992262	cassava4.1_014237m	sorting nexin 1	GP
cassava4.1_014247m|PACid:17974901	cassava4.1_014247m	Haloacid dehalogenase-like hydrolase (HAD) superfamily protein	GP
cassava4.1_014250m|PACid:17988620	cassava4.1_014250m	RNA-binding (RRM/RBD/RNP motifs) family protein	No
cassava4.1_014255m|PACid:17986698	cassava4.1_014255m	Protein of unknown function (DUF3353)	G
cassava4.1_014259m|PACid:17960618	cassava4.1_014259m	C-terminal domain phosphatase-like 4	No
cassava4.1_014260m|PACid:17978132	cassava4.1_014260m	pre-mRNA-processing protein 40C	G
cassava4.1_014261m|PACid:17985181	cassava4.1_014261m	Proteasome component (PCI) domain protein	P
cassava4.1_014262m|PACid:17964017	cassava4.1_014262m	expansin-like A1	GP
cassava4.1_014268m|PACid:17981876	cassava4.1_014268m	expansin A4	GP
cassava4.1_014269m|PACid:17961515	cassava4.1_014269m	cyclophilin 20-2	GP
cassava4.1_014276m|PACid:17964744	cassava4.1_014276m	signal recognition particle binding	G
cassava4.1_014277m|PACid:17988580	cassava4.1_014277m	SPX domain gene 3	G
cassava4.1_014278m|PACid:17961316	cassava4.1_014278m	HAD superfamily, subfamily IIIB acid phosphatase	No
cassava4.1_014288m|PACid:17992593	cassava4.1_014288m	Transmembrane CLPTM1 family protein	G
cassava4.1_014289m|PACid:17966045	cassava4.1_014289m	nuclear factor Y, subunit C9	G
cassava4.1_014291m|PACid:17973671	cassava4.1_014291m	S-adenosyl-L-methionine-dependent methyltransferases superfamily protein	GP
cassava4.1_014294m|PACid:17981983	cassava4.1_014294m		G
cassava4.1_014298m|PACid:17975435	cassava4.1_014298m	NC domain-containing protein-related	G
cassava4.1_014305m|PACid:17985848	cassava4.1_014305m	chorismate mutase 2	No
cassava4.1_014307m|PACid:17963043	cassava4.1_014307m	HARMLESS TO OZONE LAYER 1	G
cassava4.1_014312m|PACid:17966379	cassava4.1_014312m	Pathogenesis-related thaumatin superfamily protein	G
cassava4.1_014316m|PACid:17989250	cassava4.1_014316m	Adenine nucleotide alpha hydrolases-like superfamily protein	G

cassava4.1_014318m|PACid:17986612	cassava4.1_014318m	alpha/beta-Hydrolases superfamily protein	No
cassava4.1_014319m|PACid:17993090	cassava4.1_014319m	general regulatory factor 9	No
cassava4.1_014321m|PACid:17992853	cassava4.1_014321m	S-adenosyl-L-methionine-dependent methyltransferases superfamily protein	No
cassava4.1_014322m|PACid:17962933	cassava4.1_014322m	alpha/beta-Hydrolases superfamily protein	G
cassava4.1_014325m|PACid:17989307	cassava4.1_014325m	Ribosomal protein L7Ae/L30e/S12e/Gadd45 family protein	GP
cassava4.1_014329m|PACid:17984858	cassava4.1_014329m	NAD(P)-binding Rossmann-fold superfamily protein	GP
cassava4.1_014334m|PACid:17976582	cassava4.1_014334m	seryl-tRNA synthetase / serine--tRNA ligase	No
cassava4.1_014335m|PACid:17970634	cassava4.1_014335m	Ribosomal protein L7Ae/L30e/S12e/Gadd45 family protein	P
cassava4.1_014337m|PACid:17972936	cassava4.1_014337m	ELMO/CED-12 family protein	G
cassava4.1_014338m|PACid:17963109	cassava4.1_014338m	carbonic anhydrase 1	No
cassava4.1_014347m|PACid:17966479	cassava4.1_014347m	WUSCHEL related homeobox 13	G
cassava4.1_014348m|PACid:17961370	cassava4.1_014348m	Thioredoxin superfamily protein	GP
cassava4.1_014349m|PACid:17965541	cassava4.1_014349m	AIG2-like (avirulence induced gene) family protein	G
cassava4.1_014351m|PACid:17979653	cassava4.1_014351m	zinc knuckle (CCHC-type) family protein	G
cassava4.1_014352m|PACid:17969792	cassava4.1_014352m	ortholog of human splicing factor SC35	GP
cassava4.1_014354m|PACid:17993622	cassava4.1_014354m	Polynucleotidyl transferase, ribonuclease H-like superfamily protein	G
cassava4.1_014356m|PACid:17966795	cassava4.1_014356m	RNA-binding (RRM/RBD/RNP motifs) family protein	No
cassava4.1_014361m|PACid:17982723	cassava4.1_014361m	Haloacid dehalogenase-like hydrolase (HAD) superfamily protein	GP
cassava4.1_014372m|PACid:17974338	cassava4.1_014372m	RNA-binding (RRM/RBD/RNP motifs) family protein	GP
cassava4.1_014374m|PACid:17992847	cassava4.1_014374m	beta carbonic anhydrase 3	P
cassava4.1_014378m|PACid:17981031	cassava4.1_014378m	Translocon-associated protein (TRAP), alpha subunit	GP
cassava4.1_014381m|PACid:17988161	cassava4.1_014381m	Subunits of heterodimeric actin filament capping protein Capz superfamily	No
cassava4.1_014385m|PACid:17988586	cassava4.1_014385m	SGNH hydrolase-type esterase superfamily protein	No
cassava4.1_014387m|PACid:17963915	cassava4.1_014387m	3\'-5\'-exoribonuclease family protein	No
cassava4.1_014391m|PACid:17967971	cassava4.1_014391m	light harvesting complex photosystem II subunit 6	No
cassava4.1_014398m|PACid:17988472	cassava4.1_014398m	expansin A4	No
cassava4.1_014405m|PACid:17991502	cassava4.1_014405m	indole-3-butyric acid response 1	No
cassava4.1_014408m|PACid:17969587	cassava4.1_014408m	Ribosomal protein L19 family protein	G
cassava4.1_014410m|PACid:17965277	cassava4.1_014410m	chaperonin 20	GP
cassava4.1_014427m|PACid:17977563	cassava4.1_014427m	NAD(P)-binding Rossmann-fold superfamily protein	GP
cassava4.1_014428m|PACid:17987171	cassava4.1_014428m	Aluminium induced protein with YGL and LRDR motifs	GP
cassava4.1_014435m|PACid:17976610	cassava4.1_014435m	myb domain protein 4	G
cassava4.1_014438m|PACid:17983973	cassava4.1_014438m	PLAC8 family protein	GP
cassava4.1_014440m|PACid:17989528	cassava4.1_014440m	expansin A4	No
cassava4.1_014448m|PACid:17986961	cassava4.1_014448m	modifier of snc1,4	GP
cassava4.1_014454m|PACid:17988950	cassava4.1_014454m	triosephosphate isomerase	GP
cassava4.1_014459m|PACid:17966816	cassava4.1_014459m	NADH-ubiquinone oxidoreductase 24 kDa subunit, putative	No
cassava4.1_014465m|PACid:17964691	cassava4.1_014465m	rotamase CYP 4	GP
cassava4.1_014467m|PACid:17967502	cassava4.1_014467m	Alkaline phytoceramidase (aPHC)	No

cassava4.1_014468m|PACid:17981767	cassava4.1_014468m	FKBP-like peptidyl-prolyl cis-trans isomerase family protein	G
cassava4.1_014470m|PACid:17989126	cassava4.1_014470m	thiamin pyrophosphokinase1	No
cassava4.1_014471m|PACid:17963808	cassava4.1_014471m	D-isomer specific 2-hydroxyacid dehydrogenase family protein	No
cassava4.1_014474m|PACid:17981607	cassava4.1_014474m	spliceosomal protein U1A	G
cassava4.1_014476m|PACid:17979834	cassava4.1_014476m	CAP-binding protein 20	G
cassava4.1_014477m|PACid:17985348	cassava4.1_014477m	LUC7 N_terminus domain-containing protein	No
cassava4.1_014479m|PACid:17988989	cassava4.1_014479m	plant VAP homolog 12	GP
cassava4.1_014480m|PACid:17980316	cassava4.1_014480m	Pathogenesis-related thaumatin superfamily protein	GP
cassava4.1_014487m|PACid:17986262	cassava4.1_014487m	Mitochondrial substrate carrier family protein	No
cassava4.1_014492m|PACid:17983354	cassava4.1_014492m	NAC domain containing protein 83	G
cassava4.1_014493m|PACid:17966720	cassava4.1_014493m	Sodium Bile acid symporter family	G
cassava4.1_014497m|PACid:17988457	cassava4.1_014497m	RING/FYVE/PHD zinc finger superfamily protein	No
cassava4.1_014500m|PACid:17961942	cassava4.1_014500m	plastid developmental protein DAG, putative	GP
cassava4.1_014513m|PACid:17960426	cassava4.1_014513m	expansin 11	No
cassava4.1_014515m|PACid:17975665	cassava4.1_014515m	Reticulon family protein	P
cassava4.1_014517m|PACid:17991489	cassava4.1_014517m	carboxylesterases	P
cassava4.1_014521m|PACid:17984782	cassava4.1_014521m	alfin-like 5	G
cassava4.1_014525m|PACid:17981401	cassava4.1_014525m	myb domain protein 4	No
cassava4.1_014527m|PACid:17971530	cassava4.1_014527m	alfin-like 6	GP
cassava4.1_014529m|PACid:17988064	cassava4.1_014529m	basic transcription factor 3	GP
cassava4.1_014530m|PACid:17980657	cassava4.1_014530m	general regulatory factor 8	P
cassava4.1_014531m|PACid:17976020	cassava4.1_014531m	alfin-like 3	No
cassava4.1_014538m|PACid:17977334	cassava4.1_014538m	Polyketide cyclase / dehydrase and lipid transport protein	No
cassava4.1_014542m|PACid:17962440	cassava4.1_014542m	S-adenosyl-L-methionine-dependent methyltransferases superfamily protein	G
cassava4.1_014543m|PACid:17982825	cassava4.1_014543m	zinc ion binding;nucleic acid binding;zinc ion binding	G
cassava4.1_014546m|PACid:17971552	cassava4.1_014546m	UNC-50 family protein	G
cassava4.1_014547m|PACid:17991559	cassava4.1_014547m	zinc knuckle (CCHC-type) family protein	G
cassava4.1_014548m|PACid:17979018	cassava4.1_014548m	Integral membrane Yip1 family protein	G
cassava4.1_014549m|PACid:17960179	cassava4.1_014549m	expansin A8	No
cassava4.1_014552m|PACid:17964869	cassava4.1_014552m	NAD(P)-binding Rossmann-fold superfamily protein	GP
cassava4.1_014555m|PACid:17985193	cassava4.1_014555m	FKBP-like peptidyl-prolyl cis-trans isomerase family protein	GP
cassava4.1_014557m|PACid:17965669	cassava4.1_014557m	Ribosomal protein L30/L7 family protein	GP
cassava4.1_014560m|PACid:17986401	cassava4.1_014560m	Nuclear transport factor 2 (NTF2) family protein	No
cassava4.1_014561m|PACid:17980397	cassava4.1_014561m	gamma tonoplast intrinsic protein	GP
cassava4.1_014574m|PACid:17974382	cassava4.1_014574m	Immunoglobulin E-set superfamily protein	No
cassava4.1_014577m|PACid:17971975	cassava4.1_014577m	expansin A20	No
cassava4.1_014581m|PACid:17966137	cassava4.1_014581m	Acyl-CoA N-acyltransferases (NAT) superfamily protein	G
cassava4.1_014585m|PACid:17967879	cassava4.1_014585m	expansin A1	No
cassava4.1_014588m|PACid:17990101	cassava4.1_014588m	Alba DNA/RNA-binding protein	GP

cassava4.1_014591m|PACid:17968912	cassava4.1_014591m	pyridoxine biosynthesis 2	GP
cassava4.1_014592m|PACid:17980211	cassava4.1_014592m	tRNA (guanine-N-7) methyltransferase	G
cassava4.1_014594m|PACid:17968000	cassava4.1_014594m	Haloacid dehalogenase-like hydrolase (HAD) superfamily protein	G
cassava4.1_014597m|PACid:17981820	cassava4.1_014597m		G
cassava4.1_014599m|PACid:17993445	cassava4.1_014599m	electron transfer flavoprotein beta	GP
cassava4.1_014616m|PACid:17986938	cassava4.1_014616m	FKBP-like peptidyl-prolyl cis-trans isomerase family protein	GP
cassava4.1_014623m|PACid:17976289	cassava4.1_014623m	tonoplast intrinsic protein 2;3	G
cassava4.1_014626m|PACid:17979034	cassava4.1_014626m	glutathione S-transferase THETA 2	GP
cassava4.1_014632m|PACid:17977803	cassava4.1_014632m	related to AP2 6l	G
cassava4.1_014635m|PACid:17987781	cassava4.1_014635m	EAP30/Vps36 family protein	G
cassava4.1_014636m|PACid:17965011	cassava4.1_014636m	20S proteasome alpha subunit C1	No
cassava4.1_014637m|PACid:17976899	cassava4.1_014637m	Thioredoxin superfamily protein	GP
cassava4.1_014638m|PACid:17984520	cassava4.1_014638m	Nodulin MtN3 family protein	G
cassava4.1_014642m|PACid:17980870	cassava4.1_014642m	ascorbate peroxidase 1	P
cassava4.1_014647m|PACid:17982953	cassava4.1_014647m	lysophosphatidyl acyltransferase 2	No
cassava4.1_014672m|PACid:17966532	cassava4.1_014672m	Peptide methionine sulfoxide reductase family protein	No
cassava4.1_014674m|PACid:17976440	cassava4.1_014674m	Aconitase/3-isopropylmalate dehydratase protein	GP
cassava4.1_014675m|PACid:17976325	cassava4.1_014675m	peroxin 19-1	G
cassava4.1_014679m|PACid:17976615	cassava4.1_014679m	proteasome alpha subunit D2	P
cassava4.1_014683m|PACid:17968129	cassava4.1_014683m	Pathogenesis-related thaumatin superfamily protein	G
cassava4.1_014684m|PACid:17981900	cassava4.1_014684m	20S proteasome alpha subunit G1	GP
cassava4.1_014687m|PACid:17969016	cassava4.1_014687m	Glycolipid transfer protein (GLTP) family protein	No
cassava4.1_014690m|PACid:17987647	cassava4.1_014690m	Polynucleotidyl transferase, ribonuclease H-like superfamily protein	No
cassava4.1_014691m|PACid:17987416	cassava4.1_014691m	Peptidyl-tRNA hydrolase family protein	No
cassava4.1_014697m|PACid:17993541	cassava4.1_014697m	Aluminium induced protein with YGL and LRDR motifs	GP
cassava4.1_014699m|PACid:17988512	cassava4.1_014699m	Ribosomal protein S6e	GP
cassava4.1_014701m|PACid:17983127	cassava4.1_014701m	calcineurin B-like protein 10	No
cassava4.1_014710m|PACid:17984125	cassava4.1_014710m	delta tonoplast integral protein	GP
cassava4.1_014718m|PACid:17978831	cassava4.1_014718m	BAX inhibitor 1	No
cassava4.1_014719m|PACid:17980023	cassava4.1_014719m	expansin A1	No
cassava4.1_014729m|PACid:17963232	cassava4.1_014729m	phosphomannomutase	P
cassava4.1_014739m|PACid:17974273	cassava4.1_014739m	osmotin 34	GP
cassava4.1_014740m|PACid:17967060	cassava4.1_014740m	RAS associated with diabetes protein 51C	G
cassava4.1_014748m|PACid:17963433	cassava4.1_014748m	ELMO/CED-12 family protein	No
cassava4.1_014750m|PACid:17987309	cassava4.1_014750m	Ribosomal protein L30/L7 family protein	G
cassava4.1_014751m|PACid:17975474	cassava4.1_014751m	Domain of unknown function (DUF303)	GP
cassava4.1_014759m|PACid:17961962	cassava4.1_014759m	Bax inhibitor-1 family protein	No
cassava4.1_014760m|PACid:17993098	cassava4.1_014760m	Reticulon family protein	No
cassava4.1_014763m|PACid:17985392	cassava4.1_014763m	2-oxoglutarate (2OG) and Fe(II)-dependent oxygenase superfamily protein	No

cassava4.1_014765m|PACid:17982866	cassava4.1_014765m	Ribosomal protein L13 family protein	GP
cassava4.1_014767m|PACid:17970070	cassava4.1_014767m	tonoplast intrinsic protein 4;1	GP
cassava4.1_014769m|PACid:17969861	cassava4.1_014769m	Phosphoglycerate mutase family protein	G
cassava4.1_014770m|PACid:17977107	cassava4.1_014770m	LOB domain-containing protein 37	No
cassava4.1_014772m|PACid:17993985	cassava4.1_014772m	LOB domain-containing protein 37	G
cassava4.1_014778m|PACid:17965029	cassava4.1_014778m	gamma carbonic anhydrase like 1	GP
cassava4.1_014781m|PACid:17967679	cassava4.1_014781m	RNI-like superfamily protein	No
cassava4.1_014782m|PACid:17959976	cassava4.1_014782m	syntaxin of plants 61	G
cassava4.1_014785m|PACid:17960288	cassava4.1_014785m	20S proteasome subunit PAA2	GP
cassava4.1_014788m|PACid:17993303	cassava4.1_014788m	Sec14p-like phosphatidylinositol transfer family protein	G
cassava4.1_014790m|PACid:17960564	cassava4.1_014790m	PPPDE putative thiol peptidase family protein	No
cassava4.1_014793m|PACid:17969986	cassava4.1_014793m		G
cassava4.1_014796m|PACid:17960952	cassava4.1_014796m	SET domain group 37	G
cassava4.1_014799m|PACid:17972994	cassava4.1_014799m	20S proteasome subunit PAA2	GP
cassava4.1_014804m|PACid:17976606	cassava4.1_014804m	Adenine nucleotide alpha hydrolases-like superfamily protein	GP
cassava4.1_014806m|PACid:17961426	cassava4.1_014806m	vacuolar iron transporter 1	No
cassava4.1_014813m|PACid:17970020	cassava4.1_014813m		GP
cassava4.1_014819m|PACid:17975903	cassava4.1_014819m	adenylate kinase 1	P
cassava4.1_014829m|PACid:17978602	cassava4.1_014829m	RNA-binding (RRM/RBD/RNP motifs) family protein	GP
cassava4.1_014831m|PACid:17966073	cassava4.1_014831m	RNA-binding (RRM/RBD/RNP motifs) family protein	G
cassava4.1_014835m|PACid:17981321	cassava4.1_014835m	SC35-like splicing factor 30A	No
cassava4.1_014836m|PACid:17979214	cassava4.1_014836m	Translation initiation factor IF6	GP
cassava4.1_014844m|PACid:17975135	cassava4.1_014844m	nuclear-encoded CLP protease P7	No
cassava4.1_014848m|PACid:17983924	cassava4.1_014848m	Protein of unknown function (DUF1637)	G
cassava4.1_014849m|PACid:17989380	cassava4.1_014849m	indoleacetic acid-induced protein 16	G
cassava4.1_014851m|PACid:17991484	cassava4.1_014851m	endonuclease/exonuclease/phosphatase family protein	G
cassava4.1_014854m|PACid:17961298	cassava4.1_014854m	nuclear shuttle interacting	GP
cassava4.1_014856m|PACid:17985134	cassava4.1_014856m		No
cassava4.1_014857m|PACid:17960406	cassava4.1_014857m	thaumatin-like protein 3	G
cassava4.1_014859m|PACid:17962662	cassava4.1_014859m	Cytochrome b561/ferric reductase transmembrane protein family	No
cassava4.1_014860m|PACid:17992367	cassava4.1_014860m		P
cassava4.1_014862m|PACid:17973313	cassava4.1_014862m	cystatin B	GP
cassava4.1_014873m|PACid:17976527	cassava4.1_014873m	Protein of unknown function (DUF1218)	G
cassava4.1_014876m|PACid:17994112	cassava4.1_014876m	DNA-3-methyladenine glycosylase (MAG)	G
cassava4.1_014878m|PACid:17985319	cassava4.1_014878m	P-loop containing nucleoside triphosphate hydrolases superfamily protein	GP
cassava4.1_014881m|PACid:17982856	cassava4.1_014881m	Peptidase M20/M25/M40 family protein	P
cassava4.1_014882m|PACid:17985463	cassava4.1_014882m	copper ion binding	GP
cassava4.1_014883m|PACid:17981961	cassava4.1_014883m	RING/U-box superfamily protein	G
cassava4.1_014891m|PACid:17977740	cassava4.1_014891m	DERLIN-2.2	No

cassava4.1_014898m|PACid:17988583	cassava4.1_014898m	differentiation and greening-like 1	GP
cassava4.1_014902m|PACid:17974450	cassava4.1_014902m		No
cassava4.1_014906m|PACid:17983484	cassava4.1_014906m	photosystem I light harvesting complex gene 1	GP
cassava4.1_014907m|PACid:17966231	cassava4.1_014907m	RING/U-box superfamily protein	G
cassava4.1_014909m|PACid:17967980	cassava4.1_014909m		GP
cassava4.1_014911m|PACid:17980900	cassava4.1_014911m	myb domain protein 48	G
cassava4.1_014913m|PACid:17980929	cassava4.1_014913m	splicing endonuclease 2	G
cassava4.1_014918m|PACid:17982507	cassava4.1_014918m	nicotinate/nicotinamide mononucleotide adenyltransferase	GP
cassava4.1_014923m|PACid:17967764	cassava4.1_014923m	urease accessory protein F	G
cassava4.1_014928m|PACid:17962096	cassava4.1_014928m	indole-3-acetic acid inducible 14	No
cassava4.1_014931m|PACid:17961450	cassava4.1_014931m	Sec14p-like phosphatidylinositol transfer family protein	G
cassava4.1_014936m|PACid:17964271	cassava4.1_014936m	DERLIN-1	G
cassava4.1_014937m|PACid:17961898	cassava4.1_014937m	Haloacid dehalogenase-like hydrolase (HAD) superfamily protein	G
cassava4.1_014943m|PACid:17987725	cassava4.1_014943m	Cystathionine beta-synthase (CBS) family protein	P
cassava4.1_014946m|PACid:17987046	cassava4.1_014946m	nicotinamidase 1	No
cassava4.1_014947m|PACid:17989648	cassava4.1_014947m	Ribosomal protein S5 domain 2-like superfamily protein	No
cassava4.1_014954m|PACid:17972950	cassava4.1_014954m	Ankyrin repeat family protein	No
cassava4.1_014955m|PACid:17972519	cassava4.1_014955m	Nucleotide-diphospho-sugar transferases superfamily protein	GP
cassava4.1_014956m|PACid:17970152	cassava4.1_014956m	LisH and RanBPM domains containing protein	GP
cassava4.1_014957m|PACid:17976980	cassava4.1_014957m	SGNH hydrolase-type esterase superfamily protein	No
cassava4.1_014966m|PACid:17992860	cassava4.1_014966m	Pentatricopeptide repeat (PPR) superfamily protein	G
cassava4.1_014967m|PACid:17974405	cassava4.1_014967m	tubulin folding cofactor B	GP
cassava4.1_014969m|PACid:17974199	cassava4.1_014969m	SGNH hydrolase-type esterase superfamily protein	GP
cassava4.1_014971m|PACid:17986193	cassava4.1_014971m	Bax inhibitor-1 family protein	G
cassava4.1_014972m|PACid:17961282	cassava4.1_014972m	elongation factor P (EF-P) family protein	No
cassava4.1_014978m|PACid:17982748	cassava4.1_014978m	Translin family protein	GP
cassava4.1_014989m|PACid:17978763	cassava4.1_014989m	expansin A5	No
cassava4.1_014992m|PACid:17980335	cassava4.1_014992m	RING/U-box superfamily protein	No
cassava4.1_014994m|PACid:17992511	cassava4.1_014994m	Ribosomal protein S5 domain 2-like superfamily protein	G
cassava4.1_014995m|PACid:17978560	cassava4.1_014995m	3\'-5\'-exoribonuclease family protein	G
cassava4.1_015001m|PACid:17991837	cassava4.1_015001m	PNAS-3 related	GP
cassava4.1_015004m|PACid:17989751	cassava4.1_015004m	Glutathione S-transferase family protein	P
cassava4.1_015007m|PACid:17974597	cassava4.1_015007m	Lojap-related protein	G
cassava4.1_015011m|PACid:17971224	cassava4.1_015011m	copper ion binding;cobalt ion binding;zinc ion binding	GP
cassava4.1_015014m|PACid:17993154	cassava4.1_015014m	transcription regulatory protein SNF5, putative (BSH)	No
cassava4.1_015018m|PACid:17974385	cassava4.1_015018m	proton gradient regulation 7	No
cassava4.1_015026m|PACid:17987350	cassava4.1_015026m	Molybdenum cofactor sulfurase family protein	No
cassava4.1_015030m|PACid:17988633	cassava4.1_015030m	Tetratricopeptide repeat (TPR)-like superfamily protein	GP
cassava4.1_015032m|PACid:17962633	cassava4.1_015032m	Tho complex subunit 7/Mft1p	G

cassava4.1_015044m|PACid:17982240	cassava4.1_015044m	ORMDL family protein	G
cassava4.1_015050m|PACid:17988891	cassava4.1_015050m	alfin-like 1	G
cassava4.1_015054m|PACid:17974176	cassava4.1_015054m	nucleoside diphosphate kinase 2	GP
cassava4.1_015063m|PACid:17985166	cassava4.1_015063m	golgi snare 12	GP
cassava4.1_015069m|PACid:17977321	cassava4.1_015069m	Membrane fusion protein Use1	No
cassava4.1_015072m|PACid:17966876	cassava4.1_015072m	Protein of unknown function (DUF760)	No
cassava4.1_015073m|PACid:17962877	cassava4.1_015073m	Chaperone DnaJ-domain superfamily protein	No
cassava4.1_015074m|PACid:17992312	cassava4.1_015074m	nudix hydrolase homolog 27	G
cassava4.1_015076m|PACid:17961908	cassava4.1_015076m	alpha/beta-Hydrolases superfamily protein	GP
cassava4.1_015077m|PACid:17979398	cassava4.1_015077m	Far-red impaired responsive (FAR1) family protein	No
cassava4.1_015080m|PACid:17990031	cassava4.1_015080m	Rhodanese/Cell cycle control phosphatase superfamily protein	GP
cassava4.1_015083m|PACid:17974320	cassava4.1_015083m	Mog1/PsbP/DUF1795-like photosystem II reaction center PsbP family protein	GP
cassava4.1_015085m|PACid:17970949	cassava4.1_015085m	glutathione peroxidase 6	P
cassava4.1_015090m|PACid:17985930	cassava4.1_015090m	glutathione peroxidase 1	No
cassava4.1_015091m|PACid:17975241	cassava4.1_015091m	RING/FYVE/PHD zinc finger superfamily protein	No
cassava4.1_015094m|PACid:17962034	cassava4.1_015094m	indole-3-butyric acid response 10	GP
cassava4.1_015097m|PACid:17966021	cassava4.1_015097m	HSP20-like chaperones superfamily protein	No
cassava4.1_015099m|PACid:17975792	cassava4.1_015099m	salt tolerance homologue	No
cassava4.1_015100m|PACid:17961368	cassava4.1_015100m	Glutathione S-transferase family protein	GP
cassava4.1_015102m|PACid:17985186	cassava4.1_015102m	Plant VAMP (vesicle-associated membrane protein) family protein	No
cassava4.1_015109m|PACid:17993004	cassava4.1_015109m	Tetratricopeptide repeat (TPR)-like superfamily protein	GP
cassava4.1_015116m|PACid:17987913	cassava4.1_015116m	succinate dehydrogenase 5	GP
cassava4.1_015117m|PACid:17983411	cassava4.1_015117m	fatty acid hydroxylase 1	No
cassava4.1_015120m|PACid:17971210	cassava4.1_015120m	SOUL heme-binding family protein	GP
cassava4.1_015125m|PACid:17967658	cassava4.1_015125m	basic helix-loop-helix (bHLH) DNA-binding superfamily protein	No
cassava4.1_015127m|PACid:17970169	cassava4.1_015127m	20S proteasome alpha subunit E2	P
cassava4.1_015129m|PACid:17976154	cassava4.1_015129m	SNF7 family protein	G
cassava4.1_015152m|PACid:17970288	cassava4.1_015152m	PsbP-like protein 1	No
cassava4.1_015155m|PACid:17976385	cassava4.1_015155m	ubiquitin C-terminal hydrolase 3	G
cassava4.1_015159m|PACid:17962217	cassava4.1_015159m	Aluminium induced protein with YGL and LRDR motifs	No
cassava4.1_015165m|PACid:17992972	cassava4.1_015165m	Phosphoglycerate mutase family protein	GP
cassava4.1_015166m|PACid:17966454	cassava4.1_015166m	cyclophilin 5	GP
cassava4.1_015167m|PACid:17990298	cassava4.1_015167m	Calcium-binding EF-hand family protein	G
cassava4.1_015179m|PACid:17987811	cassava4.1_015179m	basic helix-loop-helix (bHLH) DNA-binding superfamily protein	G
cassava4.1_015181m|PACid:17993529	cassava4.1_015181m	seed gene 1	G
cassava4.1_015185m|PACid:17968263	cassava4.1_015185m	proteasome subunit PAB1	P
cassava4.1_015190m|PACid:17982487	cassava4.1_015190m	Haloacid dehalogenase-like hydrolase (HAD) superfamily protein	G
cassava4.1_015194m|PACid:17985315	cassava4.1_015194m	PLAC8 family protein	G
cassava4.1_015196m|PACid:17978109	cassava4.1_015196m	C2H2-like zinc finger protein	G

cassava4.1_015203m|PACid:17963284	cassava4.1_015203m	peroxin 11c	No
cassava4.1_015208m|PACid:17984250	cassava4.1_015208m	Mannose-P-dolichol utilization defect 1 protein	No
cassava4.1_015209m|PACid:17961403	cassava4.1_015209m	caffeoyl-CoA 3-O-methyltransferase	No
cassava4.1_015214m|PACid:17959907	cassava4.1_015214m	Protein of unknown function, DUF599	G
cassava4.1_015218m|PACid:17976262	cassava4.1_015218m	peroxin 11c	GP
cassava4.1_015225m|PACid:17966368	cassava4.1_015225m	RING/U-box superfamily protein	No
cassava4.1_015231m|PACid:17973047	cassava4.1_015231m	ubiquitin-conjugating enzyme 34	No
cassava4.1_015236m|PACid:17974968	cassava4.1_015236m	Protein of unknown function (DUF155)	No
cassava4.1_015242m|PACid:17965258	cassava4.1_015242m	GroES-like zinc-binding alcohol dehydrogenase family protein	GP
cassava4.1_015246m|PACid:17984780	cassava4.1_015246m	Ribosomal protein S3 family protein	No
cassava4.1_015256m|PACid:17976712	cassava4.1_015256m	heat shock protein 21	GP
cassava4.1_015259m|PACid:17965285	cassava4.1_015259m	eukaryotic translation initiation factor 3K	GP
cassava4.1_015262m|PACid:17992606	cassava4.1_015262m	RING/U-box superfamily protein	G
cassava4.1_015272m|PACid:17960181	cassava4.1_015272m	manganese superoxide dismutase 1	P
cassava4.1_015277m|PACid:17992049	cassava4.1_015277m	SNARE-like superfamily protein	GP
cassava4.1_015278m|PACid:17992158	cassava4.1_015278m	Developmental regulator, ULTRAPETALA	G
cassava4.1_015282m|PACid:17967501	cassava4.1_015282m	syntaxin of plants 52	No
cassava4.1_015284m|PACid:17986119	cassava4.1_015284m	plastid ribosomal protein l11	GP
cassava4.1_015294m|PACid:17969950	cassava4.1_015294m	Acyl-CoA N-acyltransferases (NAT) superfamily protein	GP
cassava4.1_015297m|PACid:17963585	cassava4.1_015297m	NIFU-like protein 2	GP
cassava4.1_015298m|PACid:17981540	cassava4.1_015298m	endoplasmic reticulum auxin binding protein 1	No
cassava4.1_015299m|PACid:17990683	cassava4.1_015299m	atypical CYS HIS rich thioredoxin 2	GP
cassava4.1_015302m|PACid:17989659	cassava4.1_015302m	cytochrome B561-1	G
cassava4.1_015304m|PACid:17982357	cassava4.1_015304m	FKBP-like peptidyl-prolyl cis-trans isomerase family protein	No
cassava4.1_015313m|PACid:17960129	cassava4.1_015313m	Class I peptide chain release factor	No
cassava4.1_015314m|PACid:17981228	cassava4.1_015314m	Acyl-CoA N-acyltransferases (NAT) superfamily protein	P
cassava4.1_015319m|PACid:17975913	cassava4.1_015319m	Translation elongation factor EF1B/ribosomal protein S6 family protein	GP
cassava4.1_015323m|PACid:17987686	cassava4.1_015323m	NYC1-like	No
cassava4.1_015324m|PACid:17982559	cassava4.1_015324m		G
cassava4.1_015326m|PACid:17979657	cassava4.1_015326m	Cyclophilin-like peptidyl-prolyl cis-trans isomerase family protein	No
cassava4.1_015334m|PACid:17990938	cassava4.1_015334m	FKBP-like peptidyl-prolyl cis-trans isomerase family protein	GP
cassava4.1_015336m|PACid:17980935	cassava4.1_015336m	DHBP synthase RibB-like alpha/beta domain	No
cassava4.1_015341m|PACid:17964605	cassava4.1_015341m	nucleotide-sensitive chloride conductance regulator (ICln) family protein	GP
cassava4.1_015349m|PACid:17959817	cassava4.1_015349m	N-terminal nucleophile aminohydrolases (Ntn hydrolases) superfamily protein	GP
cassava4.1_015350m|PACid:17985543	cassava4.1_015350m	Cytochrome b561/ferric reductase transmembrane protein family	GP
cassava4.1_015360m|PACid:17986760	cassava4.1_015360m	Ribosomal protein L6 family protein	GP
cassava4.1_015370m|PACid:17975008	cassava4.1_015370m	ribonuclease 2	GP
cassava4.1_015376m|PACid:17987283	cassava4.1_015376m	Domain of unknown function (DUF298)	G
cassava4.1_015379m|PACid:17966507	cassava4.1_015379m	vacuolar ATP synthase subunit E1	GP

cassava4.1_015380m|PACid:17979414	cassava4.1_015380m	vacuolar ATP synthase subunit E1	GP
cassava4.1_015381m|PACid:17987962	cassava4.1_015381m	Ergosterol biosynthesis ERG4/ERG24 family	No
cassava4.1_015382m|PACid:17963879	cassava4.1_015382m	lactoylglutathione lyase family protein / glyoxalase I family protein	GP
cassava4.1_015385m|PACid:17982478	cassava4.1_015385m	Cwf15 / Cwc15 cell cycle control family protein	G
cassava4.1_015392m|PACid:17990961	cassava4.1_015392m	DEA(D/H)-box RNA helicase family protein	No
cassava4.1_015400m|PACid:17965398	cassava4.1_015400m	Calcium-dependent lipid-binding (CaLB domain) family protein	G
cassava4.1_015404m|PACid:17979485	cassava4.1_015404m	phosducin-like protein 3 homolog	No
cassava4.1_015406m|PACid:17988411	cassava4.1_015406m	Glutathione S-transferase family protein	GP
cassava4.1_015411m|PACid:17979306	cassava4.1_015411m	Thioesterase superfamily protein	GP
cassava4.1_015419m|PACid:17975704	cassava4.1_015419m	decoy	No
cassava4.1_015420m|PACid:17980502	cassava4.1_015420m	Got1/Sft2-like vescicle transport protein family	G
cassava4.1_015422m|PACid:17965082	cassava4.1_015422m	nuclear factor Y, subunit C1	G
cassava4.1_015426m|PACid:17972189	cassava4.1_015426m	non-intrinsic ABC protein 10	G
cassava4.1_015428m|PACid:17992409	cassava4.1_015428m	Ribosomal protein L10 family protein	No
cassava4.1_015432m|PACid:17969196	cassava4.1_015432m	Uncharacterized protein family (UPF0016)	No
cassava4.1_015433m|PACid:17960538	cassava4.1_015433m	TRAM, LAG1 and CLN8 (TLC) lipid-sensing domain containing protein	G
cassava4.1_015434m|PACid:17985684	cassava4.1_015434m	S-adenosyl-L-methionine-dependent methyltransferases superfamily protein	P
cassava4.1_015438m|PACid:17979123	cassava4.1_015438m	Thioredoxin superfamily protein	GP
cassava4.1_015439m|PACid:17974652	cassava4.1_015439m	plastid developmental protein DAG, putative	No
cassava4.1_015447m|PACid:17983307	cassava4.1_015447m	polyubiquitin 3	G
cassava4.1_015449m|PACid:17992562	cassava4.1_015449m	FH interacting protein 1	P
cassava4.1_015455m|PACid:17991886	cassava4.1_015455m	SELT-like protein precursor	G
cassava4.1_015460m|PACid:17963934	cassava4.1_015460m	SNF7 family protein	No
cassava4.1_015462m|PACid:17983340	cassava4.1_015462m	embryo sac development arrest 14	No
cassava4.1_015465m|PACid:17977248	cassava4.1_015465m	copper chaperone for SOD1	GP
cassava4.1_015468m|PACid:17969899	cassava4.1_015468m	DNA-directed RNA polymerases	No
cassava4.1_015470m|PACid:17978851	cassava4.1_015470m	PPPDE putative thiol peptidase family protein	No
cassava4.1_015475m|PACid:17959858	cassava4.1_015475m	PPPDE putative thiol peptidase family protein	G
cassava4.1_015478m|PACid:17976333	cassava4.1_015478m	PPPDE putative thiol peptidase family protein	G
cassava4.1_015482m|PACid:17960514	cassava4.1_015482m	RING/U-box superfamily protein	G
cassava4.1_015483m|PACid:17973363	cassava4.1_015483m	photosystem I subunit F	G
cassava4.1_015496m|PACid:17964441	cassava4.1_015496m		G
cassava4.1_015498m|PACid:17965539	cassava4.1_015498m	membrin 11	G
cassava4.1_015500m|PACid:17976817	cassava4.1_015500m	Nuclear transport factor 2 (NTF2) family protein	GP
cassava4.1_015501m|PACid:17975203	cassava4.1_015501m	novel cap-binding protein	No
cassava4.1_015502m|PACid:17974600	cassava4.1_015502m	photosynthetic electron transfer C	No
cassava4.1_015512m|PACid:17968815	cassava4.1_015512m	ROP-interactive CRIB motif-containing protein 6	No
cassava4.1_015514m|PACid:17968181	cassava4.1_015514m	phosphatidylinositol synthase 1	G
cassava4.1_015530m|PACid:17964987	cassava4.1_015530m	Cytidine/deoxycytidylate deaminase family protein	No

cassava4.1_015536m|PACid:17987905	cassava4.1_015536m	Phosphoglycerate mutase family protein	GP
cassava4.1_015537m|PACid:17975564	cassava4.1_015537m	RAB GTPase homolog A3	No
cassava4.1_015542m|PACid:17991313	cassava4.1_015542m	nudix hydrolase homolog 12	No
cassava4.1_015543m|PACid:17992518	cassava4.1_015543m	K-box region and MADS-box transcription factor family protein	G
cassava4.1_015558m|PACid:17981713	cassava4.1_015558m	Vacuolar iron transporter (VIT) family protein	No
cassava4.1_015564m|PACid:17968208	cassava4.1_015564m	coenzyme Q biosynthesis Coq4 family protein / ubiquinone biosynthesis Coq4 family protein	No
cassava4.1_015568m|PACid:17988921	cassava4.1_015568m	RAB GTPase homolog A4A	P
cassava4.1_015570m|PACid:17962332	cassava4.1_015570m	NAD(P)-binding Rossmann-fold superfamily protein	G
cassava4.1_015573m|PACid:17965234	cassava4.1_015573m	Aldolase-type TIM barrel family protein	GP
cassava4.1_015575m|PACid:17980651	cassava4.1_015575m	K-box region and MADS-box transcription factor family protein	G
cassava4.1_015576m|PACid:17979717	cassava4.1_015576m	Fumarylacetoacetate (FAA) hydrolase family	P
cassava4.1_015578m|PACid:17963938	cassava4.1_015578m	copper/zinc superoxide dismutase 2	No
cassava4.1_015583m|PACid:17981521	cassava4.1_015583m	Protein kinase superfamily protein	No
cassava4.1_015589m|PACid:17976674	cassava4.1_015589m	Ribosomal protein L6 family	No
cassava4.1_015590m|PACid:17980143	cassava4.1_015590m	RECA homolog 3	No
cassava4.1_015603m|PACid:17990014	cassava4.1_015603m	vacuolar protein sorting 26A	GP
cassava4.1_015614m|PACid:17986780	cassava4.1_015614m	RAB GTPase homolog A4C	No
cassava4.1_015617m|PACid:17976561	cassava4.1_015617m	transcription regulators	No
cassava4.1_015618m|PACid:17967797	cassava4.1_015618m	Glutathione S-transferase, C-terminal-like;Translation elongation factor EF1B/ribosomal protein S6	GP
cassava4.1_015622m|PACid:17961127	cassava4.1_015622m	Protein of unknown function (DUF1640)	GP
cassava4.1_015623m|PACid:17970860	cassava4.1_015623m	glutathione S-transferase zeta 1	GP
cassava4.1_015625m|PACid:17989124	cassava4.1_015625m	cyclin p3;1	G
cassava4.1_015626m|PACid:17973146	cassava4.1_015626m	SNF7 family protein	G
cassava4.1_015629m|PACid:17966174	cassava4.1_015629m	cyclin p2;1	G
cassava4.1_015632m|PACid:17989620	cassava4.1_015632m	S-adenosyl-L-methionine-dependent methyltransferases superfamily protein	GP
cassava4.1_015640m|PACid:17982460	cassava4.1_015640m	RNA-binding KH domain-containing protein	G
cassava4.1_015644m|PACid:17970853	cassava4.1_015644m	Translation initiation factor eIF3 subunit	GP
cassava4.1_015646m|PACid:17975834	cassava4.1_015646m	Metal-dependent phosphohydrolase	GP
cassava4.1_015648m|PACid:17961672	cassava4.1_015648m	Cyclophilin-like peptidyl-prolyl cis-trans isomerase family protein	G
cassava4.1_015655m|PACid:17963177	cassava4.1_015655m	Vesicle transport v-SNARE family protein	No
cassava4.1_015656m|PACid:17967118	cassava4.1_015656m	PHD finger family protein / bromo-adjacent homology (BAH) domain-containing protein	G
cassava4.1_015657m|PACid:17967153	cassava4.1_015657m	FKBP-like peptidyl-prolyl cis-trans isomerase family protein	No
cassava4.1_015658m|PACid:17990084	cassava4.1_015658m		GP
cassava4.1_015667m|PACid:17963200	cassava4.1_015667m	N-terminal nucleophile aminohydrolases (Ntn hydrolases) superfamily protein	P
cassava4.1_015669m|PACid:17973011	cassava4.1_015669m	Cyclophilin-like peptidyl-prolyl cis-trans isomerase family protein	GP
cassava4.1_015674m|PACid:17987448	cassava4.1_015674m		G
cassava4.1_015675m|PACid:17983871	cassava4.1_015675m	nitrilase-like protein 1	P
cassava4.1_015683m|PACid:17991427	cassava4.1_015683m	emp24/gp25L/p24 family/GOLD family protein	P
cassava4.1_015684m|PACid:17986467	cassava4.1_015684m	MAK16 protein-related	No

cassava4.1_015701m|PACid:17992126	cassava4.1_015701m	RAB GTPase homolog A5A	P
cassava4.1_015707m|PACid:17963638	cassava4.1_015707m	pyrophosphorylase 4	GP
cassava4.1_015708m|PACid:17964541	cassava4.1_015708m	polyadenylate-binding protein 1	G
cassava4.1_015716m|PACid:17990291	cassava4.1_015716m	basic helix-loop-helix (bHLH) DNA-binding superfamily protein	No
cassava4.1_015721m|PACid:17962643	cassava4.1_015721m	PLATZ transcription factor family protein	G
cassava4.1_015726m|PACid:17993847	cassava4.1_015726m	B-cell receptor-associated 31-like	GP
cassava4.1_015729m|PACid:17993796	cassava4.1_015729m	Lateral organ boundaries (LOB) domain family protein	No
cassava4.1_015737m|PACid:17974737	cassava4.1_015737m	nascent polypeptide-associated complex subunit alpha-like protein 2	GP
cassava4.1_015747m|PACid:17978792	cassava4.1_015747m	Plant VAMP (vesicle-associated membrane protein) family protein	No
cassava4.1_015753m|PACid:17982664	cassava4.1_015753m	DNA replication protein-related	No
cassava4.1_015756m|PACid:17985424	cassava4.1_015756m	NFU domain protein 3	G
cassava4.1_015757m|PACid:17974929	cassava4.1_015757m	Cyclophilin-like peptidyl-prolyl cis-trans isomerase family protein	GP
cassava4.1_015758m|PACid:17971464	cassava4.1_015758m	vesicle-associated membrane protein 724	No
cassava4.1_015762m|PACid:17966378	cassava4.1_015762m	6,7-dimethyl-8-ribityllumazine synthase / DMRL synthase / lumazine synthase / riboflavin synthase	GP
cassava4.1_015763m|PACid:17968116	cassava4.1_015763m	SNF7 family protein	GP
cassava4.1_015768m|PACid:17978900	cassava4.1_015768m	Ribosomal protein L16p/L10e family protein	GP
cassava4.1_015775m|PACid:17988259	cassava4.1_015775m	Ribosomal protein L16p/L10e family protein	No
cassava4.1_015778m|PACid:17968232	cassava4.1_015778m		G
cassava4.1_015781m|PACid:17966037	cassava4.1_015781m	Co-chaperone GrpE family protein	GP
cassava4.1_015793m|PACid:17959868	cassava4.1_015793m	vesicle-associated membrane protein 713	P
cassava4.1_015794m|PACid:17975878	cassava4.1_015794m	NAD(P)-binding Rossmann-fold superfamily protein	GP
cassava4.1_015811m|PACid:17976839	cassava4.1_015811m	vesicle-associated membrane protein 714	GP
cassava4.1_015812m|PACid:17988742	cassava4.1_015812m	C-8,7 sterol isomerase	GP
cassava4.1_015813m|PACid:17989983	cassava4.1_015813m	myosin heavy chain-related	G
cassava4.1_015814m|PACid:17980131	cassava4.1_015814m	Vesicle transport v-SNARE family protein	No
cassava4.1_015817m|PACid:17981263	cassava4.1_015817m	Thioredoxin superfamily protein	GP
cassava4.1_015822m|PACid:17962333	cassava4.1_015822m	gamma-soluble NSF attachment protein	GP
cassava4.1_015824m|PACid:17964910	cassava4.1_015824m	SMAD/FHA domain-containing protein	No
cassava4.1_015826m|PACid:17993920	cassava4.1_015826m		G
cassava4.1_015833m|PACid:17987968	cassava4.1_015833m	ChaC-like family protein	No
cassava4.1_015834m|PACid:17967611	cassava4.1_015834m	vesicle-associated membrane protein 726	No
cassava4.1_015835m|PACid:17987690	cassava4.1_015835m	Leucine-rich repeat (LRR) family protein	G
cassava4.1_015837m|PACid:17989351	cassava4.1_015837m	SSXT family protein	No
cassava4.1_015840m|PACid:17961184	cassava4.1_015840m	RAB GTPase homolog A5D	GP
cassava4.1_015842m|PACid:17993321	cassava4.1_015842m	Peptidase C15, pyroglutamyl peptidase I-like	No
cassava4.1_015843m|PACid:17960806	cassava4.1_015843m	C-repeat-binding factor 4	G
cassava4.1_015844m|PACid:17967554	cassava4.1_015844m	Ribosomal protein S8e family protein	GP
cassava4.1_015848m|PACid:17981106	cassava4.1_015848m	Quinone reductase family protein	No
cassava4.1_015852m|PACid:17979490	cassava4.1_015852m	histone H1-3	G

cassava4.1_015854m|PACid:17970064	cassava4.1_015854m	glutathione S-transferase TAU 19	GP
cassava4.1_015858m|PACid:17964669	cassava4.1_015858m	germin-like protein 10	P
cassava4.1_015862m|PACid:17985940	cassava4.1_015862m	SNF7 family protein	GP
cassava4.1_015872m|PACid:17973424	cassava4.1_015872m	glutathione S-transferase TAU 19	GP
cassava4.1_015878m|PACid:17981879	cassava4.1_015878m	Calcium-binding EF-hand family protein	G
cassava4.1_015883m|PACid:17962128	cassava4.1_015883m	basic helix-loop-helix (bHLH) DNA-binding superfamily protein	No
cassava4.1_015893m|PACid:17988739	cassava4.1_015893m	RNA-binding (RRM/RBD/RNP motifs) family protein	No
cassava4.1_015895m|PACid:17973168	cassava4.1_015895m	Synaptobrevin family protein	No
cassava4.1_015896m|PACid:17974172	cassava4.1_015896m	response regulator 5	No
cassava4.1_015898m|PACid:17968770	cassava4.1_015898m	Putative lysine decarboxylase family protein	No
cassava4.1_015899m|PACid:17978920	cassava4.1_015899m	B-cell receptor-associated protein 31-like	G
cassava4.1_015901m|PACid:17982670	cassava4.1_015901m	sequence-specific DNA binding transcription factors	G
cassava4.1_015908m|PACid:17983271	cassava4.1_015908m	Galactose oxidase/kelch repeat superfamily protein	GP
cassava4.1_015909m|PACid:17984212	cassava4.1_015909m	methionine sulfoxide reductase B 2	GP
cassava4.1_015911m|PACid:17972316	cassava4.1_015911m	SGS domain-containing protein	GP
cassava4.1_015912m|PACid:17992141	cassava4.1_015912m	Tetraspanin family protein	No
cassava4.1_015917m|PACid:17985920	cassava4.1_015917m	stromal cell-derived factor 2-like protein precursor	G
cassava4.1_015918m|PACid:17980306	cassava4.1_015918m	RNA-binding (RRM/RBD/RNP motifs) family protein	No
cassava4.1_015925m|PACid:17972102	cassava4.1_015925m	Putative lysine decarboxylase family protein	No
cassava4.1_015934m|PACid:17970040	cassava4.1_015934m	RAB GTPase homolog A1F	GP
cassava4.1_015936m|PACid:17961131	cassava4.1_015936m	PHD finger family protein / bromo-adjacent homology (BAH) domain-containing protein	No
cassava4.1_015938m|PACid:17979066	cassava4.1_015938m	Ras-related small GTP-binding family protein	GP
cassava4.1_015960m|PACid:17965485	cassava4.1_015960m	Ribosomal protein L7/L12, oligomerisation;Ribosomal protein L7/L12, C-terminal/adaptor protein ClpS-like	No
cassava4.1_015964m|PACid:17964577	cassava4.1_015964m	protein binding;zinc ion binding	No
cassava4.1_015966m|PACid:17983377	cassava4.1_015966m	germin-like protein 10	P
cassava4.1_015968m|PACid:17981842	cassava4.1_015968m	RAB GTPase homolog A2B	No
cassava4.1_015969m|PACid:17971089	cassava4.1_015969m	RAB GTPase homolog A1F	GP
cassava4.1_015972m|PACid:17973537	cassava4.1_015972m	Ribosomal protein L1p/L10e family	GP
cassava4.1_015987m|PACid:17960542	cassava4.1_015987m	LisH and RanBPM domains containing protein	No
cassava4.1_015988m|PACid:17969187	cassava4.1_015988m	Integral membrane HPP family protein	No
cassava4.1_015990m|PACid:17972480	cassava4.1_015990m	RAB GTPase homolog C2A	No
cassava4.1_015993m|PACid:17969256	cassava4.1_015993m	Ribosomal protein L17 family protein	GP
cassava4.1_015998m|PACid:17974803	cassava4.1_015998m	RAB GTPase 11C	GP
cassava4.1_016001m|PACid:17960326	cassava4.1_016001m		G
cassava4.1_016005m|PACid:17978916	cassava4.1_016005m	endoplasmic reticulum retention defective 2B	G
cassava4.1_016006m|PACid:17977474	cassava4.1_016006m	tRNA/rRNA methyltransferase (SpoU) family protein	G
cassava4.1_016020m|PACid:17978189	cassava4.1_016020m	ER lumen protein retaining receptor family protein	No
cassava4.1_016021m|PACid:17990682	cassava4.1_016021m	Thioredoxin superfamily protein	GP
cassava4.1_016025m|PACid:17977371	cassava4.1_016025m	Mob1/phocein family protein	G

cassava4.1_016028m|PACid:17964487	cassava4.1_016028m	NADH-ubiquinone oxidoreductase 20 kDa subunit, mitochondrial	GP
cassava4.1_016034m|PACid:17968195	cassava4.1_016034m	plasmodesmata callose-binding protein 3	No
cassava4.1_016035m|PACid:17977565	cassava4.1_016035m		G
cassava4.1_016043m|PACid:17980013	cassava4.1_016043m	emp24/gp25L/p24 family/GOLD family protein	GP
cassava4.1_016053m|PACid:17985673	cassava4.1_016053m	Polyketide cyclase/dehydrase and lipid transport superfamily protein	GP
cassava4.1_016054m|PACid:17964907	cassava4.1_016054m	PYR1-like 4	G
cassava4.1_016056m|PACid:17966201	cassava4.1_016056m	pyrophosphorylase 2	P
cassava4.1_016064m|PACid:17978681	cassava4.1_016064m	snRNA activating complex family protein	G
cassava4.1_016068m|PACid:17988757	cassava4.1_016068m	glutathione S-transferase phi 8	GP
cassava4.1_016073m|PACid:17990257	cassava4.1_016073m	Thioredoxin superfamily protein	GP
cassava4.1_016074m|PACid:17988477	cassava4.1_016074m	non-intrinsic ABC protein 9	G
cassava4.1_016078m|PACid:17983202	cassava4.1_016078m	prenylated RAB acceptor 1.B4	GP
cassava4.1_016079m|PACid:17978992	cassava4.1_016079m		GP
cassava4.1_016083m|PACid:17991272	cassava4.1_016083m	calcineurin B-like protein 1	No
cassava4.1_016084m|PACid:17967258	cassava4.1_016084m	RNA-binding (RRM/RBD/RNP motifs) family protein	No
cassava4.1_016087m|PACid:17964267	cassava4.1_016087m	S-adenosyl-L-methionine-dependent methyltransferases superfamily protein	No
cassava4.1_016088m|PACid:17971625	cassava4.1_016088m	RING/U-box superfamily protein	No
cassava4.1_016102m|PACid:17970278	cassava4.1_016102m	dehydroascorbate reductase 2	GP
cassava4.1_016105m|PACid:17989104	cassava4.1_016105m	Plant-specific transcription factor YABBY family protein	No
cassava4.1_016114m|PACid:17967064	cassava4.1_016114m	RNA-binding (RRM/RBD/RNP motifs) family protein with retrovirus zinc finger-like domain	No
cassava4.1_016116m|PACid:17960774	cassava4.1_016116m	membrane-associated progesterone binding protein 3	GP
cassava4.1_016121m|PACid:17991115	cassava4.1_016121m	HAL3-like protein A	G
cassava4.1_016123m|PACid:17959945	cassava4.1_016123m	SC35-like splicing factor 30A	GP
cassava4.1_016125m|PACid:17982142	cassava4.1_016125m	homolog of CFIM-25	G
cassava4.1_016126m|PACid:17968812	cassava4.1_016126m	vacuolar protein sorting-associated protein 2.3	No
cassava4.1_016130m|PACid:17963251	cassava4.1_016130m	AGAMOUS-like 20	G
cassava4.1_016132m|PACid:17965158	cassava4.1_016132m	Ribosomal L28 family	No
cassava4.1_016137m|PACid:17969609	cassava4.1_016137m	Octicosapeptide/Phox/Bem1p family protein	G
cassava4.1_016157m|PACid:17963088	cassava4.1_016157m	GTP-binding 2	GP
cassava4.1_016159m|PACid:17978682	cassava4.1_016159m	RAB GTPase homolog B1C	GP
cassava4.1_016162m|PACid:17990614	cassava4.1_016162m	TBP-associated factor 11	No
cassava4.1_016169m|PACid:17974081	cassava4.1_016169m	RHO-related protein from plants 10	No
cassava4.1_016170m|PACid:17961143	cassava4.1_016170m	B-box type zinc finger family protein	No
cassava4.1_016173m|PACid:17964349	cassava4.1_016173m	mitochondrially targeted single-stranded DNA binding protein	GP
cassava4.1_016191m|PACid:17982045	cassava4.1_016191m	Nucleic acid-binding, OB-fold-like protein	G
cassava4.1_016192m|PACid:17990321	cassava4.1_016192m	Ribosomal protein L19e family protein	No
cassava4.1_016193m|PACid:17993079	cassava4.1_016193m	glutathione S-transferase PHI 9	P
cassava4.1_016194m|PACid:17971141	cassava4.1_016194m	ADP-ribosylation factor A1B	No
cassava4.1_016199m|PACid:17985474	cassava4.1_016199m	D-Tyr-tRNA(Tyr) deacylase family protein	No

cassava4.1_016205m|PACid:17982469	cassava4.1_016205m	Ribosomal protein S10p/S20e family protein	No
cassava4.1_016210m|PACid:17989119	cassava4.1_016210m	P-loop containing nucleoside triphosphate hydrolases superfamily protein	No
cassava4.1_016211m|PACid:17960168	cassava4.1_016211m	Chalcone-flavanone isomerase family protein	No
cassava4.1_016220m|PACid:17961578	cassava4.1_016220m	Uncharacterised conserved protein ycf60	GP
cassava4.1_016222m|PACid:17973655	cassava4.1_016222m	RAB GTPASE HOMOLOG B18	GP
cassava4.1_016224m|PACid:17975421	cassava4.1_016224m	NDR1/HIN1-like 1	GP
cassava4.1_016231m|PACid:17970151	cassava4.1_016231m	Vacuolar protein sorting-associated protein VPS28 family protein	G
cassava4.1_016232m|PACid:17959968	cassava4.1_016232m	ribosomal protein 5B	G
cassava4.1_016237m|PACid:17964221	cassava4.1_016237m	RHO-related protein from plants 9	No
cassava4.1_016243m|PACid:17990684	cassava4.1_016243m	germin 3	G
cassava4.1_016252m|PACid:17988578	cassava4.1_016252m	prenylated RAB acceptor 1.A3	G
cassava4.1_016255m|PACid:17993519	cassava4.1_016255m	emp24/gp25L/p24 family/GOLD family protein	GP
cassava4.1_016260m|PACid:17962823	cassava4.1_016260m	prenylated RAB acceptor 1.A1	G
cassava4.1_016261m|PACid:17989372	cassava4.1_016261m	pathogenesis-related 4	No
cassava4.1_016263m|PACid:17961144	cassava4.1_016263m	B-box type zinc finger family protein	No
cassava4.1_016265m|PACid:17987778	cassava4.1_016265m	OTU-like cysteine protease family protein	No
cassava4.1_016282m|PACid:17967419	cassava4.1_016282m	Transcriptional coactivator/pterin dehydratase	GP
cassava4.1_016290m|PACid:17977841	cassava4.1_016290m		G
cassava4.1_016291m|PACid:17965583	cassava4.1_016291m	adenosine-5\'-phosphosulfate (APS) kinase 3	No
cassava4.1_016295m|PACid:17975655	cassava4.1_016295m	GRF1-interacting factor 3	No
cassava4.1_016299m|PACid:17963410	cassava4.1_016299m	DNA-binding HORMA family protein	No
cassava4.1_016300m|PACid:17983738	cassava4.1_016300m	RAB GTPase homolog G3D	GP
cassava4.1_016309m|PACid:17988636	cassava4.1_016309m	PSF2	G
cassava4.1_016310m|PACid:17978635	cassava4.1_016310m	Acyl-CoA N-acyltransferases (NAT) superfamily protein	G
cassava4.1_016312m|PACid:17982044	cassava4.1_016312m	photosystem I subunit D-2	GP
cassava4.1_016313m|PACid:17968722	cassava4.1_016313m	decapping 2	No
cassava4.1_016318m|PACid:17960875	cassava4.1_016318m	GTP-binding protein 1	G
cassava4.1_016319m|PACid:17989041	cassava4.1_016319m	Ras-related small GTP-binding family protein	GP
cassava4.1_016323m|PACid:17983107	cassava4.1_016323m	Protein of unknown function (DUF1000)	GP
cassava4.1_016326m|PACid:17982811	cassava4.1_016326m	RAB GTPase homolog G3F	GP
cassava4.1_016331m|PACid:17967605	cassava4.1_016331m	Ribosomal protein L13 family protein	GP
cassava4.1_016339m|PACid:17973036	cassava4.1_016339m	Nascent polypeptide-associated complex (NAC), alpha subunit family protein	GP
cassava4.1_016341m|PACid:17973461	cassava4.1_016341m	Uncharacterised protein family (UPF0497)	G
cassava4.1_016342m|PACid:17978935	cassava4.1_016342m	ribosomal protein S9	GP
cassava4.1_016348m|PACid:17969236	cassava4.1_016348m	breast basic conserved 1	GP
cassava4.1_016350m|PACid:17991154	cassava4.1_016350m	Thioesterase superfamily protein	G
cassava4.1_016357m|PACid:17992208	cassava4.1_016357m	GATA type zinc finger transcription factor family protein	G
cassava4.1_016358m|PACid:17988777	cassava4.1_016358m	Sugar isomerase (SIS) family protein	G
cassava4.1_016362m|PACid:17964533	cassava4.1_016362m	SOH1 family protein	G

cassava4.1_016379m|PACid:17960069	cassava4.1_016379m	RAB GTPase homolog G3A	P
cassava4.1_016390m|PACid:17962926	cassava4.1_016390m	Protein of unknown function (DUF567)	No
cassava4.1_016396m|PACid:17964019	cassava4.1_016396m	Ribosomal protein L2 family	G
cassava4.1_016399m|PACid:17973941	cassava4.1_016399m	nicotinamidase 2	G
cassava4.1_016446m|PACid:17985337	cassava4.1_016446m	Ribosomal protein L23/L15e family protein	GP
cassava4.1_016447m|PACid:17976897	cassava4.1_016447m	Ribosomal protein L13 family protein	GP
cassava4.1_016449m|PACid:17969461	cassava4.1_016449m	Rer1 family protein	No
cassava4.1_016457m|PACid:17961228	cassava4.1_016457m	zinc induced facilitator-like 1	No
cassava4.1_016464m|PACid:17968627	cassava4.1_016464m	ribosomal protein L12-A	GP
cassava4.1_016465m|PACid:17964780	cassava4.1_016465m	proteasome beta subunit C1	GP
cassava4.1_016470m|PACid:17978114	cassava4.1_016470m	glutathione peroxidase 2	GP
cassava4.1_016479m|PACid:17978846	cassava4.1_016479m	RAS 5	GP
cassava4.1_016480m|PACid:17964586	cassava4.1_016480m	RAS 5	GP
cassava4.1_016489m|PACid:17980441	cassava4.1_016489m	Ankyrin repeat family protein	No
cassava4.1_016496m|PACid:17972795	cassava4.1_016496m	Pyridoxamine 5\'-phosphate oxidase family protein	No
cassava4.1_016498m|PACid:17970049	cassava4.1_016498m	flavodoxin-like quinone reductase 1	P
cassava4.1_016499m|PACid:17988134	cassava4.1_016499m	Uncharacterised conserved protein UCP009193	No
cassava4.1_016500m|PACid:17991929	cassava4.1_016500m	Quinone reductase family protein	GP
cassava4.1_016501m|PACid:17985328	cassava4.1_016501m	RAS 5	GP
cassava4.1_016503m|PACid:17976038	cassava4.1_016503m	vacuolar protein sorting 46.1	GP
cassava4.1_016505m|PACid:17975554	cassava4.1_016505m	electron transport SCO1/SenC family protein	GP
cassava4.1_016519m|PACid:17988671	cassava4.1_016519m	cyclin p4;1	G
cassava4.1_016521m|PACid:17972160	cassava4.1_016521m	ATPase, F1 complex, delta/epsilon subunit	GP
cassava4.1_016523m|PACid:17981245	cassava4.1_016523m	WCRKC thioredoxin 2	No
cassava4.1_016526m|PACid:17992227	cassava4.1_016526m	cold-regulated 413-plasma membrane 2	No
cassava4.1_016530m|PACid:17980087	cassava4.1_016530m	S-adenosyl-L-methionine-dependent methyltransferases superfamily protein	G
cassava4.1_016536m|PACid:17967514	cassava4.1_016536m	Uncharacterised protein family (UPF0172)	GP
cassava4.1_016539m|PACid:17966205	cassava4.1_016539m	SEC14 cytosolic factor family protein / phosphoglyceride transfer family protein	G
cassava4.1_016540m|PACid:17987336	cassava4.1_016540m	cold-regulated 413-plasma membrane 2	No
cassava4.1_016542m|PACid:17988319	cassava4.1_016542m	Mog1/PsbP/DUF1795-like photosystem II reaction center PsbP family protein	G
cassava4.1_016543m|PACid:17962216	cassava4.1_016543m	20S proteasome beta subunit D1	GP
cassava4.1_016551m|PACid:17993180	cassava4.1_016551m	C2H2 and C2HC zinc fingers superfamily protein	G
cassava4.1_016554m|PACid:17978876	cassava4.1_016554m	Calcium-binding EF-hand family protein	G
cassava4.1_016558m|PACid:17970255	cassava4.1_016558m	squamosa promoter binding protein-like 5	G
cassava4.1_016569m|PACid:17981086	cassava4.1_016569m	partner of SLD five 1	G
cassava4.1_016572m|PACid:17962390	cassava4.1_016572m	Protein of unknown function (DUF1218)	G
cassava4.1_016573m|PACid:17965567	cassava4.1_016573m	TATA binding protein 2	No
cassava4.1_016578m|PACid:17962495	cassava4.1_016578m	Ras-related small GTP-binding family protein	P
cassava4.1_016579m|PACid:17963524	cassava4.1_016579m	RNA polymerase Rpb7 N-terminal domain-containing protein	G

cassava4.1_016583m|PACid:17965720	cassava4.1_016583m	FKBP-like peptidyl-prolyl cis-trans isomerase family protein	No
cassava4.1_016584m|PACid:17972240	cassava4.1_016584m	nuclear factor Y, subunit B3	G
cassava4.1_016592m|PACid:17981387	cassava4.1_016592m	TMPIT-like protein	GP
cassava4.1_016593m|PACid:17988762	cassava4.1_016593m	Arabidopsis RAC-like 9	No
cassava4.1_016595m|PACid:17981226	cassava4.1_016595m	Ras-related small GTP-binding family protein	P
cassava4.1_016599m|PACid:17967931	cassava4.1_016599m	Acyl-CoA N-acyltransferases (NAT) superfamily protein	No
cassava4.1_016604m|PACid:17960808	cassava4.1_016604m	Cleavage/polyadenylation specificity factor, 25kDa subunit	GP
cassava4.1_016605m|PACid:17993486	cassava4.1_016605m	Protein of unknown function (DUF640)	G
cassava4.1_016606m|PACid:17993420	cassava4.1_016606m	RmlC-like cupins superfamily protein	G
cassava4.1_016616m|PACid:17968946	cassava4.1_016616m	Protein of unknown function (DUF962)	GP
cassava4.1_016620m|PACid:17962162	cassava4.1_016620m	Eukaryotic initiation factor 4E protein	GP
cassava4.1_016626m|PACid:17970007	cassava4.1_016626m	Mitochondrial substrate carrier family protein	No
cassava4.1_016630m|PACid:17990022	cassava4.1_016630m	TBP-associated factor 7	G
cassava4.1_016652m|PACid:17992485	cassava4.1_016652m	NADH:ubiquinone oxidoreductase intermediate-associated protein 30	G
cassava4.1_016658m|PACid:17980506	cassava4.1_016658m	nuclear factor Y, subunit B3	No
cassava4.1_016661m|PACid:17990658	cassava4.1_016661m	Lactoylglutathione lyase / glyoxalase I family protein	G
cassava4.1_016672m|PACid:17984393	cassava4.1_016672m	NADPH:quinone oxidoreductase	No
cassava4.1_016673m|PACid:17962416	cassava4.1_016673m	glycerol-3-phosphatase 1	GP
cassava4.1_016680m|PACid:17968452	cassava4.1_016680m	PIN domain-like family protein	G
cassava4.1_016681m|PACid:17981406	cassava4.1_016681m	RAC-like 3	G
cassava4.1_016682m|PACid:17992549	cassava4.1_016682m	purin 7	GP
cassava4.1_016688m|PACid:17960156	cassava4.1_016688m	RING/U-box superfamily protein	G
cassava4.1_016698m|PACid:17993762	cassava4.1_016698m	Ribosomal protein S4	GP
cassava4.1_016700m|PACid:17994045	cassava4.1_016700m	Isoprenylcysteine carboxyl methyltransferase (ICMT) family	No
cassava4.1_016711m|PACid:17959810	cassava4.1_016711m	Uncharacterised BCR, YbaB family COG0718	GP
cassava4.1_016713m|PACid:17982922	cassava4.1_016713m	ribosomal protein L9	GP
cassava4.1_016717m|PACid:17977354	cassava4.1_016717m	Homeodomain-like superfamily protein	G
cassava4.1_016719m|PACid:17966243	cassava4.1_016719m	Arabidopsis RAC-like 1	No
cassava4.1_016720m|PACid:17992411	cassava4.1_016720m	COP9 signalosome, subunit CSN8	GP
cassava4.1_016729m|PACid:17975664	cassava4.1_016729m	Pyridoxamine 5\'-phosphate oxidase family protein	G
cassava4.1_016734m|PACid:17969963	cassava4.1_016734m	RAC-like 2	No
cassava4.1_016739m|PACid:17966807	cassava4.1_016739m	RNA-binding (RRM/RBD/RNP motifs) family protein	GP
cassava4.1_016745m|PACid:17976071	cassava4.1_016745m	Fcf2 pre-rRNA processing protein	No
cassava4.1_016749m|PACid:17982836	cassava4.1_016749m	ribosomal protein large subunit 27	G
cassava4.1_016750m|PACid:17992705	cassava4.1_016750m	GATA type zinc finger transcription factor family protein	G
cassava4.1_016754m|PACid:17972207	cassava4.1_016754m	frataxin homolog	No
cassava4.1_016755m|PACid:17982784	cassava4.1_016755m	methionine sulfoxide reductase B 1	GP
cassava4.1_016758m|PACid:17993856	cassava4.1_016758m	Phosphotyrosine protein phosphatases superfamily protein	G
cassava4.1_016772m|PACid:17969444	cassava4.1_016772m	Translation initiation factor SUI1 family protein	No

cassava4.1_016773m|PACid:17967727	cassava4.1_016773m	molybdopterin biosynthesis MoaE family protein	GP
cassava4.1_016776m|PACid:17984088	cassava4.1_016776m	NDH-dependent cyclic electron flow 1	GP
cassava4.1_016778m|PACid:17985303	cassava4.1_016778m	Plant invertase/pectin methylesterase inhibitor superfamily protein	GP
cassava4.1_016781m|PACid:17963802	cassava4.1_016781m	GLNB1 homolog	P
cassava4.1_016784m|PACid:17985758	cassava4.1_016784m	adenine phosphoribosyltransferase 5	GP
cassava4.1_016785m|PACid:17964063	cassava4.1_016785m	RNA ligase/cyclic nucleotide phosphodiesterase family protein	GP
cassava4.1_016788m|PACid:17983718	cassava4.1_016788m	Isochorismatase family protein	GP
cassava4.1_016789m|PACid:17963616	cassava4.1_016789m	Acyl-CoA N-acyltransferases (NAT) superfamily protein	G
cassava4.1_016794m|PACid:17976109	cassava4.1_016794m	translocon-associated protein beta (TRAPB) family protein	No
cassava4.1_016800m|PACid:17970694	cassava4.1_016800m	Thioesterase superfamily protein	G
cassava4.1_016802m|PACid:17986668	cassava4.1_016802m	endoplasmatic reticulum retrieval protein 1B	G
cassava4.1_016803m|PACid:17993627	cassava4.1_016803m	homeobox protein 54	G
cassava4.1_016807m|PACid:17987689	cassava4.1_016807m	Ribosomal protein L6 family	GP
cassava4.1_016808m|PACid:17971942	cassava4.1_016808m	Ribosomal protein L6 family	GP
cassava4.1_016816m|PACid:17965009	cassava4.1_016816m	ubiquitin-conjugating enzyme 27	G
cassava4.1_016819m|PACid:17963223	cassava4.1_016819m	Remorin family protein	GP
cassava4.1_016825m|PACid:17977924	cassava4.1_016825m	thioredoxin F2	GP
cassava4.1_016835m|PACid:17968436	cassava4.1_016835m	endoplasmatic reticulum retrieval protein 1B	No
cassava4.1_016837m|PACid:17972190	cassava4.1_016837m	Transport protein particle (TRAPP) component	No
cassava4.1_016842m|PACid:17962255	cassava4.1_016842m	AUX/IAA transcriptional regulator family protein	G
cassava4.1_016846m|PACid:17969286	cassava4.1_016846m	ADP-ribosylation factor B1B	P
cassava4.1_016848m|PACid:17960782	cassava4.1_016848m	regulatory components of ABA receptor 3	G
cassava4.1_016850m|PACid:17991824	cassava4.1_016850m	Ribosomal protein L12/ ATP-dependent Clp protease adaptor protein ClpS family protein	No
cassava4.1_016855m|PACid:17979710	cassava4.1_016855m	secretion-associated RAS super family 2	GP
cassava4.1_016856m|PACid:17962256	cassava4.1_016856m	secretion-associated RAS super family 2	GP
cassava4.1_016859m|PACid:17979874	cassava4.1_016859m	PLANT CADMIUM RESISTANCE 2	G
cassava4.1_016860m|PACid:17961354	cassava4.1_016860m		G
cassava4.1_016871m|PACid:17989672	cassava4.1_016871m	anti- silencing function 1b	G
cassava4.1_016873m|PACid:17993367	cassava4.1_016873m	AUX/IAA transcriptional regulator family protein	No
cassava4.1_016879m|PACid:17970908	cassava4.1_016879m	Protein of unknown function DUF106, transmembrane	GP
cassava4.1_016882m|PACid:17966388	cassava4.1_016882m	anaphase promoting complex 10	G
cassava4.1_016885m|PACid:17962422	cassava4.1_016885m	histidine triad nucleotide-binding 3	G
cassava4.1_016888m|PACid:17959854	cassava4.1_016888m	serine/arginine-rich 22	GP
cassava4.1_016892m|PACid:17961463	cassava4.1_016892m	EF hand calcium-binding protein family	No
cassava4.1_016905m|PACid:17962335	cassava4.1_016905m	SNF7 family protein	No
cassava4.1_016921m|PACid:17976821	cassava4.1_016921m	Protein of unknown function (DUF1279)	No
cassava4.1_016923m|PACid:17970927	cassava4.1_016923m		G
cassava4.1_016926m|PACid:17968922	cassava4.1_016926m	Calcineurin-like metallo-phosphoesterase superfamily protein	G
cassava4.1_016933m|PACid:17978157	cassava4.1_016933m	Ribosomal protein S7e family protein	No

cassava4.1_016938m|PACid:17968422	cassava4.1_016938m	Protein of unknown function (DUF640)	G
cassava4.1_016969m|PACid:17962846	cassava4.1_016969m	Protein of unknown function (DUF1218)	G
cassava4.1_016972m|PACid:17966897	cassava4.1_016972m		G
cassava4.1_016975m|PACid:17988963	cassava4.1_016975m		G
cassava4.1_016984m|PACid:17992192	cassava4.1_016984m	thioredoxin M-type 1	G
cassava4.1_016985m|PACid:17962568	cassava4.1_016985m		G
cassava4.1_016986m|PACid:17961549	cassava4.1_016986m	glycosylphosphatidylinositol-anchored lipid protein transfer 1	No
cassava4.1_016988m|PACid:17965942	cassava4.1_016988m	calmodulin like 42	G
cassava4.1_016994m|PACid:17983343	cassava4.1_016994m	adenine phosphoribosyl transferase 2	No
cassava4.1_016999m|PACid:17991608	cassava4.1_016999m	GATA type zinc finger transcription factor family protein	No
cassava4.1_017001m|PACid:17978517	cassava4.1_017001m	regulatory component of ABA receptor 1	GP
cassava4.1_017003m|PACid:17988911	cassava4.1_017003m	GATA type zinc finger transcription factor family protein	No
cassava4.1_017005m|PACid:17987787	cassava4.1_017005m	endoribonuclease L-PSP family protein	GP
cassava4.1_017013m|PACid:17960941	cassava4.1_017013m	ChaC-like family protein	GP
cassava4.1_017018m|PACid:17989401	cassava4.1_017018m	Protein of unknown function (DUF640)	G
cassava4.1_017040m|PACid:17973291	cassava4.1_017040m	translocase of inner mitochondrial membrane 23	G
cassava4.1_017041m|PACid:17963531	cassava4.1_017041m	Ribosomal protein S19 family protein	GP
cassava4.1_017050m|PACid:17973264	cassava4.1_017050m	Thioredoxin z	P
cassava4.1_017051m|PACid:17980475	cassava4.1_017051m	Heavy metal transport/detoxification superfamily protein	No
cassava4.1_017054m|PACid:17972120	cassava4.1_017054m	PYR1-like 2	No
cassava4.1_017060m|PACid:17979183	cassava4.1_017060m	Ribosomal protein L12 family protein	G
cassava4.1_017064m|PACid:17981988	cassava4.1_017064m	cytochrome C oxidase 6B	G
cassava4.1_017067m|PACid:17977040	cassava4.1_017067m	Protein of unknown function (DUF1068)	No
cassava4.1_017068m|PACid:17982655	cassava4.1_017068m	prefoldin 3	GP
cassava4.1_017070m|PACid:17986395	cassava4.1_017070m	Mediator complex, subunit Med10	G
cassava4.1_017075m|PACid:17991285	cassava4.1_017075m		No
cassava4.1_017084m|PACid:17993531	cassava4.1_017084m	RmlC-like cupins superfamily protein	G
cassava4.1_017087m|PACid:17983522	cassava4.1_017087m	Bifunctional inhibitor/lipid-transfer protein/seed storage 2S albumin superfamily protein	No
cassava4.1_017088m|PACid:17964336	cassava4.1_017088m	ribosomal protein L12-A	P
cassava4.1_017091m|PACid:17982532	cassava4.1_017091m	Ku70-binding family protein	No
cassava4.1_017093m|PACid:17964123	cassava4.1_017093m	ribosomal protein L18	GP
cassava4.1_017095m|PACid:17960056	cassava4.1_017095m	Hypoxanthine-guanine phosphoribosyltransferase	No
cassava4.1_017097m|PACid:17971466	cassava4.1_017097m	RNA binding	No
cassava4.1_017120m|PACid:17989052	cassava4.1_017120m	FKBP-like peptidyl-prolyl cis-trans isomerase family protein	GP
cassava4.1_017126m|PACid:17965987	cassava4.1_017126m	Cytidine/deoxycytidylate deaminase family protein	G
cassava4.1_017144m|PACid:17979585	cassava4.1_017144m	Peroxisomal membrane 22 kDa (Mpv17/PMP22) family protein	G
cassava4.1_017157m|PACid:17972690	cassava4.1_017157m	temperature-induced lipocalin	GP
cassava4.1_017166m|PACid:17962023	cassava4.1_017166m	Peroxisomal membrane 22 kDa (Mpv17/PMP22) family protein	G
cassava4.1_017168m|PACid:17987217	cassava4.1_017168m	Protein of unknown function (DUF1218)	G

cassava4.1_017171m|PACid:17974750	cassava4.1_017171m	zinc finger (C2H2 type) family protein	No
cassava4.1_017172m|PACid:17987427	cassava4.1_017172m	ADP-ribosylation factor-like A1A	GP
cassava4.1_017173m|PACid:17984484	cassava4.1_017173m	F-box family protein	G
cassava4.1_017179m|PACid:17975017	cassava4.1_017179m	ADP-ribosylation factor-like A1C	No
cassava4.1_017180m|PACid:17959938	cassava4.1_017180m	Protein of unknown function (DUF1218)	No
cassava4.1_017185m|PACid:17988993	cassava4.1_017185m	plant VAP homolog 12	No
cassava4.1_017190m|PACid:17977889	cassava4.1_017190m	Acyl-CoA N-acyltransferases (NAT) superfamily protein	No
cassava4.1_017195m|PACid:17968748	cassava4.1_017195m	pfkB-like carbohydrate kinase family protein	GP
cassava4.1_017198m|PACid:17979533	cassava4.1_017198m	Rhodanese/Cell cycle control phosphatase superfamily protein	GP
cassava4.1_017199m|PACid:17961742	cassava4.1_017199m	ADP-ribosylation factor C1	No
cassava4.1_017202m|PACid:17970970	cassava4.1_017202m	ubiquitin-conjugating enzyme 5	G
cassava4.1_017203m|PACid:17984916	cassava4.1_017203m	Dynein light chain type 1 family protein	No
cassava4.1_017205m|PACid:17981127	cassava4.1_017205m	RUB1 conjugating enzyme 1	GP
cassava4.1_017208m|PACid:17976907	cassava4.1_017208m	WRKY DNA-binding protein 75	No
cassava4.1_017209m|PACid:17989543	cassava4.1_017209m	RNA ligase/cyclic nucleotide phosphodiesterase family protein	G
cassava4.1_017237m|PACid:17968085	cassava4.1_017237m	Calcium-dependent lipid-binding (CaLB domain) family protein	G
cassava4.1_017238m|PACid:17985928	cassava4.1_017238m	glutamine dumper 3	No
cassava4.1_017252m|PACid:17976357	cassava4.1_017252m	adenine phosphoribosyl transferase 4	No
cassava4.1_017256m|PACid:17959870	cassava4.1_017256m	ADP-ribosylation factor 3	G
cassava4.1_017257m|PACid:17983571	cassava4.1_017257m	Alpha-L RNA-binding motif/Ribosomal protein S4 family protein	G
cassava4.1_017263m|PACid:17983935	cassava4.1_017263m	Ribosomal L5P family protein	GP
cassava4.1_017266m|PACid:17983657	cassava4.1_017266m	ATPase, F0/V0 complex, subunit C protein	G
cassava4.1_017268m|PACid:17981306	cassava4.1_017268m	Ubiquitin-conjugating enzyme family protein	No
cassava4.1_017272m|PACid:17968920	cassava4.1_017272m		G
cassava4.1_017274m|PACid:17964138	cassava4.1_017274m	pseudouridine synthase and archaeosine transglycosylase (PUA) domain-containing protein	GP
cassava4.1_017275m|PACid:17984472	cassava4.1_017275m	adenine phosphoribosyl transferase 1	GP
cassava4.1_017280m|PACid:17971144	cassava4.1_017280m	ADP-ribosylation factor A1F	No
cassava4.1_017282m|PACid:17968102	cassava4.1_017282m	Bifunctional inhibitor/lipid-transfer protein/seed storage 2S albumin superfamily protein	GP
cassava4.1_017289m|PACid:17966670	cassava4.1_017289m	COP9 signalosome subunit 6A	No
cassava4.1_017290m|PACid:17993867	cassava4.1_017290m	Protein of unknown function (DUF1068)	No
cassava4.1_017302m|PACid:17966417	cassava4.1_017302m		G
cassava4.1_017304m|PACid:17987378	cassava4.1_017304m	Modifier of rudimentary (Mod(r)) protein	G
cassava4.1_017305m|PACid:17965993	cassava4.1_017305m	Peptidyl-tRNA hydrolase II (PTH2) family protein	G
cassava4.1_017310m|PACid:17964615	cassava4.1_017310m	ADP-ribosylation factor A1F	No
cassava4.1_017321m|PACid:17966809	cassava4.1_017321m	ubiquitin-conjugating enzyme 20	G
cassava4.1_017335m|PACid:17962903	cassava4.1_017335m	Peptidase S24/S26A/S26B/S26C family protein	No
cassava4.1_017337m|PACid:17986164	cassava4.1_017337m	methyl-CPG-binding domain 4	G
cassava4.1_017338m|PACid:17989937	cassava4.1_017338m	RNA binding Plectin/S10 domain-containing protein	No
cassava4.1_017340m|PACid:17985824	cassava4.1_017340m		No

cassava4.1_017342m|PACid:17968454	cassava4.1_017342m	Adenine nucleotide alpha hydrolases-like superfamily protein	GP
cassava4.1_017347m|PACid:17982874	cassava4.1_017347m	Protein of unknown function (DUF640)	No
cassava4.1_017356m|PACid:17988398	cassava4.1_017356m	E2F/DP family winged-helix DNA-binding domain	G
cassava4.1_017359m|PACid:17974735	cassava4.1_017359m	FK506-binding protein 16-2	GP
cassava4.1_017366m|PACid:17976699	cassava4.1_017366m	Metallo-hydrolase/oxidoreductase superfamily protein	No
cassava4.1_017379m|PACid:17981992	cassava4.1_017379m	ARM repeat superfamily protein	No
cassava4.1_017386m|PACid:17977646	cassava4.1_017386m		No
cassava4.1_017390m|PACid:17974979	cassava4.1_017390m	Protein of unknown function, DUF538	G
cassava4.1_017398m|PACid:17983286	cassava4.1_017398m	ATPase E1	GP
cassava4.1_017402m|PACid:17974483	cassava4.1_017402m	Protein-tyrosine phosphatase-like, PTPLA	G
cassava4.1_017409m|PACid:17972025	cassava4.1_017409m	Ribosomal protein L18ae/LX family protein	GP
cassava4.1_017415m|PACid:17964090	cassava4.1_017415m	SNARE-like superfamily protein	G
cassava4.1_017418m|PACid:17964035	cassava4.1_017418m	nuclear factor Y, subunit B10	G
cassava4.1_017420m|PACid:17988495	cassava4.1_017420m	glycosyltransferase family protein 28	G
cassava4.1_017424m|PACid:17977046	cassava4.1_017424m	CAX interacting protein 1	G
cassava4.1_017425m|PACid:17963365	cassava4.1_017425m	Ribosomal protein L22p/L17e family protein	GP
cassava4.1_017431m|PACid:17982807	cassava4.1_017431m		No
cassava4.1_017432m|PACid:17968916	cassava4.1_017432m	P-loop containing nucleoside triphosphate hydrolases superfamily protein	G
cassava4.1_017438m|PACid:17967897	cassava4.1_017438m	Got1/Sft2-like vescicle transport protein family	G
cassava4.1_017445m|PACid:17982267	cassava4.1_017445m	serine/arginine-rich 22	G
cassava4.1_017450m|PACid:17971928	cassava4.1_017450m	SNARE-like superfamily protein	No
cassava4.1_017455m|PACid:17973013	cassava4.1_017455m	TATA binding protein associated factor 21kDa subunit	No
cassava4.1_017464m|PACid:17982230	cassava4.1_017464m		No
cassava4.1_017474m|PACid:17965934	cassava4.1_017474m	Protein of unknown function (DUF1000)	GP
cassava4.1_017475m|PACid:17990479	cassava4.1_017475m	Ankyrin repeat family protein	G
cassava4.1_017479m|PACid:17993910	cassava4.1_017479m	cyanase	GP
cassava4.1_017484m|PACid:17987342	cassava4.1_017484m		No
cassava4.1_017486m|PACid:17964855	cassava4.1_017486m	RNA polymerase Rpb7-like, N-terminal domain	No
cassava4.1_017491m|PACid:17981962	cassava4.1_017491m	DNA-directed RNA polymerase II	GP
cassava4.1_017495m|PACid:17974897	cassava4.1_017495m	HVA22 homologue C	G
cassava4.1_017501m|PACid:17975285	cassava4.1_017501m	Calcium-binding EF-hand family protein	GP
cassava4.1_017506m|PACid:17978957	cassava4.1_017506m	DUTP-PYROPHOSPHATASE-LIKE 1	No
cassava4.1_017507m|PACid:17961647	cassava4.1_017507m	Adenine nucleotide alpha hydrolases-like superfamily protein	GP
cassava4.1_017511m|PACid:17978078	cassava4.1_017511m	Adenine nucleotide alpha hydrolases-like superfamily protein	GP
cassava4.1_017513m|PACid:17965640	cassava4.1_017513m	Transport protein particle (TRAPP) component	GP
cassava4.1_017533m|PACid:17962340	cassava4.1_017533m	LSD1 zinc finger family protein	G
cassava4.1_017534m|PACid:17981651	cassava4.1_017534m	Acyl-CoA N-acyltransferases (NAT) superfamily protein	GP
cassava4.1_017537m|PACid:17965791	cassava4.1_017537m	rotamase cyclophilin 2	GP
cassava4.1_017539m|PACid:17977467	cassava4.1_017539m	dual specificity protein phosphatase 1	G

cassava4.1_017540m|PACid:17968533	cassava4.1_017540m		GP
cassava4.1_017542m|PACid:17983862	cassava4.1_017542m	centroradialis	No
cassava4.1_017551m|PACid:17990432	cassava4.1_017551m	SNARE-like superfamily protein	G
cassava4.1_017552m|PACid:17972570	cassava4.1_017552m	nuclear factor Y, subunit B8	G
cassava4.1_017561m|PACid:17967121	cassava4.1_017561m	Chaperonin-like RbcX protein	No
cassava4.1_017565m|PACid:17963515	cassava4.1_017565m	Ribosomal protein L18ae family	G
cassava4.1_017575m|PACid:17974679	cassava4.1_017575m	Protein of unknown function (DUF788)	No
cassava4.1_017587m|PACid:17993078	cassava4.1_017587m	Protein of unknown function (DUF640)	G
cassava4.1_017589m|PACid:17980639	cassava4.1_017589m	Adenine nucleotide alpha hydrolases-like superfamily protein	G
cassava4.1_017598m|PACid:17971738	cassava4.1_017598m	photosystem II family protein	GP
cassava4.1_017605m|PACid:17977757	cassava4.1_017605m	Mitochondrial import inner membrane translocase subunit Tim17/Tim22/Tim23 family protein	G
cassava4.1_017606m|PACid:17974947	cassava4.1_017606m	HIT-type Zinc finger family protein	No
cassava4.1_017607m|PACid:17984342	cassava4.1_017607m	Glutaredoxin family protein	GP
cassava4.1_017616m|PACid:17973681	cassava4.1_017616m	photosystem I reaction center subunit PSI-N, chloroplast, putative / PSI-N, putative (PSAN)	G
cassava4.1_017617m|PACid:17986957	cassava4.1_017617m	Calcium-binding EF-hand family protein	GP
cassava4.1_017629m|PACid:17981073	cassava4.1_017629m	Lactoylglutathione lyase / glyoxalase I family protein	G
cassava4.1_017632m|PACid:17983059	cassava4.1_017632m	Ankyrin repeat family protein	G
cassava4.1_017635m|PACid:17986724	cassava4.1_017635m	F-box family protein	G
cassava4.1_017643m|PACid:17988709	cassava4.1_017643m	Protein of unknown function (DUF498/DUF598)	No
cassava4.1_017654m|PACid:17992109	cassava4.1_017654m	RNA-binding (RRM/RBD/RNP motifs) family protein	G
cassava4.1_017662m|PACid:17968055	cassava4.1_017662m	rotamase CYP 1	GP
cassava4.1_017692m|PACid:17964917	cassava4.1_017692m	Protein of unknown function, DUF538	G
cassava4.1_017694m|PACid:17962354	cassava4.1_017694m	Protein of unknown function, DUF538	G
cassava4.1_017695m|PACid:17975677	cassava4.1_017695m	brassinosteroid-responsive RING-H2	G
cassava4.1_017700m|PACid:17971821	cassava4.1_017700m	Histone superfamily protein	No
cassava4.1_017704m|PACid:17980039	cassava4.1_017704m	centrin2	No
cassava4.1_017705m|PACid:17973574	cassava4.1_017705m	plastocyanin 1	GP
cassava4.1_017708m|PACid:17963771	cassava4.1_017708m	photosystem I P subunit	No
cassava4.1_017711m|PACid:17965064	cassava4.1_017711m	Rubredoxin-like superfamily protein	G
cassava4.1_017712m|PACid:17960757	cassava4.1_017712m		G
cassava4.1_017719m|PACid:17960873	cassava4.1_017719m	SNARE-like superfamily protein	No
cassava4.1_017720m|PACid:17985822	cassava4.1_017720m	Basic-leucine zipper (bZIP) transcription factor family protein	G
cassava4.1_017727m|PACid:17980343	cassava4.1_017727m	Peptidase S24/S26A/S26B/S26C family protein	No
cassava4.1_017728m|PACid:17982370	cassava4.1_017728m	protein binding	G
cassava4.1_017729m|PACid:17960111	cassava4.1_017729m	thioredoxin Y2	GP
cassava4.1_017731m|PACid:17981075	cassava4.1_017731m	glutaredoxin 4	GP
cassava4.1_017733m|PACid:17994066	cassava4.1_017733m	Mediator complex, subunit Med7	No
cassava4.1_017738m|PACid:17966625	cassava4.1_017738m	translationally controlled tumor protein	GP
cassava4.1_017749m|PACid:17984445	cassava4.1_017749m	RNA polymerase II, Rpb4, core protein	No

cassava4.1_017750m|PACid:17979881	cassava4.1_017750m	GCN5L1 family protein	G
cassava4.1_017755m|PACid:17979043	cassava4.1_017755m	glutathione peroxidase 6	GP
cassava4.1_017758m|PACid:17976436	cassava4.1_017758m	Bifunctional inhibitor/lipid-transfer protein/seed storage 2S albumin superfamily protein	No
cassava4.1_017759m|PACid:17977156	cassava4.1_017759m	Ribosomal protein L24e family protein	G
cassava4.1_017768m|PACid:17973768	cassava4.1_017768m	ATP synthase D chain, mitochondrial	GP
cassava4.1_017773m|PACid:17978534	cassava4.1_017773m		GP
cassava4.1_017775m|PACid:17961039	cassava4.1_017775m	Family of unknown function (DUF662)	GP
cassava4.1_017779m|PACid:17964012	cassava4.1_017779m	hemoglobin 3	G
cassava4.1_017788m|PACid:17986506	cassava4.1_017788m	Calcium-dependent lipid-binding (CaLB domain) family protein	No
cassava4.1_017790m|PACid:17989171	cassava4.1_017790m	Signal peptidase subunit	GP
cassava4.1_017796m|PACid:17960830	cassava4.1_017796m	NADH-ubiquinone oxidoreductase-related	P
cassava4.1_017799m|PACid:17974421	cassava4.1_017799m	Tetratricopeptide repeat (TPR)-like superfamily protein	G
cassava4.1_017801m|PACid:17989497	cassava4.1_017801m	ubiquitin-conjugating enzyme 14	G
cassava4.1_017802m|PACid:17967260	cassava4.1_017802m	Ribosomal protein L11 family protein	GP
cassava4.1_017814m|PACid:17962958	cassava4.1_017814m	Rubredoxin-like superfamily protein	No
cassava4.1_017815m|PACid:17979531	cassava4.1_017815m	Clathrin adaptor complex small chain family protein	G
cassava4.1_017819m|PACid:17965266	cassava4.1_017819m		GP
cassava4.1_017820m|PACid:17973430	cassava4.1_017820m	Peptidase S24/S26A/S26B/S26C family protein	G
cassava4.1_017821m|PACid:17985611	cassava4.1_017821m	Ribonuclease E inhibitor RraA/Dimethylmenaquinone methyltransferase	G
cassava4.1_017830m|PACid:17971140	cassava4.1_017830m	glucuronokinase G	G
cassava4.1_017831m|PACid:17980307	cassava4.1_017831m	ubiquitin carrier protein 7	G
cassava4.1_017832m|PACid:17961591	cassava4.1_017832m	Ribonuclease E inhibitor RraA/Dimethylmenaquinone methyltransferase	GP
cassava4.1_017833m|PACid:17972272	cassava4.1_017833m	Ribosomal protein L14p/L23e family protein	No
cassava4.1_017838m|PACid:17974112	cassava4.1_017838m	SufE/NifU family protein	G
cassava4.1_017839m|PACid:17992808	cassava4.1_017839m	Oligosaccharyltransferase complex/magnesium transporter family protein	No
cassava4.1_017845m|PACid:17991361	cassava4.1_017845m	vacuolar H+-pumping ATPase 16 kDa proteolipid subunit 4	G
cassava4.1_017856m|PACid:17979049	cassava4.1_017856m	RNA-binding (RRM/RBD/RNP motifs) family protein	No
cassava4.1_017866m|PACid:17975538	cassava4.1_017866m	PEBP (phosphatidylethanolamine-binding protein) family protein	G
cassava4.1_017868m|PACid:17985059	cassava4.1_017868m	ATPase, F0/V0 complex, subunit C protein	G
cassava4.1_017871m|PACid:17980723	cassava4.1_017871m	HSP20-like chaperones superfamily protein	GP
cassava4.1_017889m|PACid:17981366	cassava4.1_017889m	Translation protein SH3-like family protein	No
cassava4.1_017893m|PACid:17980346	cassava4.1_017893m	high mobility group B1	No
cassava4.1_017904m|PACid:17961752	cassava4.1_017904m	Adenine nucleotide alpha hydrolases-like superfamily protein	G
cassava4.1_017905m|PACid:17973873	cassava4.1_017905m	Protein of unknown function, DUF538	GP
cassava4.1_017908m|PACid:17977180	cassava4.1_017908m	ribosomal protein L24	GP
cassava4.1_017910m|PACid:17994082	cassava4.1_017910m	Translation protein SH3-like family protein	GP
cassava4.1_017913m|PACid:17963302	cassava4.1_017913m	glycine decarboxylase complex H	GP
cassava4.1_017914m|PACid:17981089	cassava4.1_017914m	Rubredoxin-like superfamily protein	GP
cassava4.1_017915m|PACid:17976624	cassava4.1_017915m	CAP (Cysteine-rich secretory proteins, Antigen 5, and Pathogenesis-related 1 protein) superfamily protein	G

cassava4.1_017922m|PACid:17983981	cassava4.1_017922m	brassinosteroid-responsive RING-H2	No
cassava4.1_017925m|PACid:17992284	cassava4.1_017925m	Cyclophilin-like peptidyl-prolyl cis-trans isomerase family protein	GP
cassava4.1_017928m|PACid:17975446	cassava4.1_017928m	embryo defective 2735	G
cassava4.1_017929m|PACid:17982251	cassava4.1_017929m	basic pathogenesis-related protein 1	No
cassava4.1_017935m|PACid:17965774	cassava4.1_017935m	EF hand calcium-binding protein family	No
cassava4.1_017937m|PACid:17983635	cassava4.1_017937m	Acyl-CoA N-acyltransferases (NAT) superfamily protein	G
cassava4.1_017938m|PACid:17992301	cassava4.1_017938m	alpha/beta-Hydrolases superfamily protein	No
cassava4.1_017944m|PACid:17979303	cassava4.1_017944m	4-phosphopantetheine adenylyltransferase	G
cassava4.1_017946m|PACid:17985004	cassava4.1_017946m	Ribosomal L18p/L5e family protein	GP
cassava4.1_017950m|PACid:17982243	cassava4.1_017950m	basic pathogenesis-related protein 1	No
cassava4.1_017954m|PACid:17988219	cassava4.1_017954m	plastid transcriptionally active7	G
cassava4.1_017973m|PACid:17985926	cassava4.1_017973m	thioredoxin-dependent peroxidase 1	GP
cassava4.1_017984m|PACid:17982614	cassava4.1_017984m	2Fe-2S ferredoxin-like superfamily protein	P
cassava4.1_017996m|PACid:17973530	cassava4.1_017996m	nuclear factor Y, subunit B11	No
cassava4.1_017997m|PACid:17969678	cassava4.1_017997m	ubiquitin-conjugating enzyme 18	No
cassava4.1_018000m|PACid:17981702	cassava4.1_018000m	Adenine nucleotide alpha hydrolases-like superfamily protein	No
cassava4.1_018005m|PACid:17972042	cassava4.1_018005m	Clathrin adaptor complex small chain family protein	No
cassava4.1_018010m|PACid:17970081	cassava4.1_018010m	Adenine nucleotide alpha hydrolases-like superfamily protein	GP
cassava4.1_018023m|PACid:17976207	cassava4.1_018023m	Cyclophilin-like peptidyl-prolyl cis-trans isomerase family protein	GP
cassava4.1_018031m|PACid:17973861	cassava4.1_018031m	Chaperone DnaJ-domain superfamily protein	G
cassava4.1_018036m|PACid:17993356	cassava4.1_018036m		G
cassava4.1_018037m|PACid:17987722	cassava4.1_018037m	sumo conjugation enzyme 1	G
cassava4.1_018045m|PACid:17961205	cassava4.1_018045m	basic transcription factor 3	G
cassava4.1_018047m|PACid:17972732	cassava4.1_018047m	ribosomal protein S11-beta	GP
cassava4.1_018049m|PACid:17975211	cassava4.1_018049m	copper/zinc superoxide dismutase 3	GP
cassava4.1_018055m|PACid:17993722	cassava4.1_018055m	Mitochondrial import inner membrane translocase subunit Tim17/Tim22/Tim23 family protein	No
cassava4.1_018056m|PACid:17983717	cassava4.1_018056m	ribosomal protein S17	GP
cassava4.1_018059m|PACid:17963195	cassava4.1_018059m	eukaryotic elongation factor 5A-1	GP
cassava4.1_018061m|PACid:17969335	cassava4.1_018061m	NADH:ubiquinone oxidoreductase, 17.2kDa subunit	GP
cassava4.1_018069m|PACid:17965111	cassava4.1_018069m	MMS ZWEI homologue 1	GP
cassava4.1_018078m|PACid:17978453	cassava4.1_018078m	thioredoxin 2	GP
cassava4.1_018081m|PACid:17973598	cassava4.1_018081m	Ribosomal protein L17 family protein	G
cassava4.1_018086m|PACid:17973593	cassava4.1_018086m	Surfeit locus protein 5 subunit 22 of Mediator complex	G
cassava4.1_018102m|PACid:17980498	cassava4.1_018102m	RING/U-box superfamily protein	G
cassava4.1_018116m|PACid:17986686	cassava4.1_018116m	Ribosomal protein L7Ae/L30e/S12e/Gadd45 family protein	No
cassava4.1_018125m|PACid:17993380	cassava4.1_018125m	HR-like lesion-inducing protein-related	GP
cassava4.1_018129m|PACid:17960396	cassava4.1_018129m	E3 ubiquitin ligase SCF complex subunit SKP1/ASK1 family protein	GP
cassava4.1_018132m|PACid:17975733	cassava4.1_018132m	ORMDL family protein	G
cassava4.1_018134m|PACid:17966503	cassava4.1_018134m	HSP20-like chaperones superfamily protein	GP

cassava4.1_018137m|PACid:17961255	cassava4.1_018137m	Regulator of chromosome condensation (RCC1) family protein	G
cassava4.1_018143m|PACid:17962261	cassava4.1_018143m	HR-like lesion-inducing protein-related	No
cassava4.1_018144m|PACid:17993792	cassava4.1_018144m	peroxin4	G
cassava4.1_018158m|PACid:17970560	cassava4.1_018158m	17.6 kDa class II heat shock protein	GP
cassava4.1_018162m|PACid:17984067	cassava4.1_018162m	Acyl-CoA N-acyltransferases (NAT) superfamily protein	No
cassava4.1_018165m|PACid:17964686	cassava4.1_018165m	ubiquitin 6	No
cassava4.1_018172m|PACid:17961067	cassava4.1_018172m	Iron-sulphur cluster biosynthesis family protein	No
cassava4.1_018174m|PACid:17972886	cassava4.1_018174m	HVA22-like protein F	G
cassava4.1_018182m|PACid:17979517	cassava4.1_018182m	S phase kinase-associated protein 1	G
cassava4.1_018187m|PACid:17977055	cassava4.1_018187m	NADH-ubiquinone oxidoreductase-related	GP
cassava4.1_018189m|PACid:17966247	cassava4.1_018189m	mitochondrial ribosomal protein L11	G
cassava4.1_018195m|PACid:17986695	cassava4.1_018195m	nuclear factor Y, subunit B13	G
cassava4.1_018198m|PACid:17982312	cassava4.1_018198m		No
cassava4.1_018202m|PACid:17992472	cassava4.1_018202m	HSP20-like chaperones superfamily protein	No
cassava4.1_018207m|PACid:17970470	cassava4.1_018207m	Single hybrid motif superfamily protein	GP
cassava4.1_018211m|PACid:17966463	cassava4.1_018211m	Protein of unknown function (DUF59)	G
cassava4.1_018220m|PACid:17977480	cassava4.1_018220m	farnesylated protein 6	GP
cassava4.1_018221m|PACid:17965428	cassava4.1_018221m	ribonuclease P family protein / Rpp14 family protein	No
cassava4.1_018230m|PACid:17962078	cassava4.1_018230m	histidine-containing phosphotransmitter 1	No
cassava4.1_018235m|PACid:17988895	cassava4.1_018235m	ribosomal protein L23AB	No
cassava4.1_018243m|PACid:17960682	cassava4.1_018243m	Uncharacterised protein family (UPF0497)	No
cassava4.1_018244m|PACid:17977476	cassava4.1_018244m	histidine-containing phosphotransmitter 1	No
cassava4.1_018245m|PACid:17967895	cassava4.1_018245m	Thioesterase superfamily protein	G
cassava4.1_018248m|PACid:17960030	cassava4.1_018248m	related to ubiquitin 1	GP
cassava4.1_018250m|PACid:17962186	cassava4.1_018250m	Ribosomal L28 family	GP
cassava4.1_018260m|PACid:17985228	cassava4.1_018260m	double-stranded RNA binding protein-related / DsRBD protein-related	No
cassava4.1_018265m|PACid:17982700	cassava4.1_018265m	ankyrin repeat family protein	G
cassava4.1_018266m|PACid:17987264	cassava4.1_018266m	ubiquitin-conjugating enzyme 35	P
cassava4.1_018277m|PACid:17984176	cassava4.1_018277m	Thioesterase superfamily protein	No
cassava4.1_018278m|PACid:17971604	cassava4.1_018278m	FK506- and rapamycin-binding protein 15 kD-2	GP
cassava4.1_018285m|PACid:17986208	cassava4.1_018285m	histidine-containing phosphotransfer factor 5	GP
cassava4.1_018286m|PACid:17982738	cassava4.1_018286m	Calcium-binding EF-hand family protein	No
cassava4.1_018287m|PACid:17986970	cassava4.1_018287m	ARF-GAP domain 11	G
cassava4.1_018288m|PACid:17983018	cassava4.1_018288m	structural molecules	G
cassava4.1_018291m|PACid:17975183	cassava4.1_018291m	ubiquiting-conjugating enzyme 2	No
cassava4.1_018296m|PACid:17988166	cassava4.1_018296m	S18 ribosomal protein	GP
cassava4.1_018300m|PACid:17970719	cassava4.1_018300m	transcriptional coactivator p15 (PC4) family protein (KELP)	G
cassava4.1_018307m|PACid:17968641	cassava4.1_018307m	Thioredoxin superfamily protein	No
cassava4.1_018316m|PACid:17985711	cassava4.1_018316m	ascorbate peroxidase 6	GP

cassava4.1_018317m|PACid:17988467	cassava4.1_018317m	ubiquiting-conjugating enzyme 2	G
cassava4.1_018320m|PACid:17989051	cassava4.1_018320m	mago nashi family protein	GP
cassava4.1_018324m|PACid:17985325	cassava4.1_018324m	Ribosomal protein S13/S15	GP
cassava4.1_018330m|PACid:17968551	cassava4.1_018330m	P4H isoform 1	G
cassava4.1_018334m|PACid:17980808	cassava4.1_018334m	photosystem I subunit G	GP
cassava4.1_018335m|PACid:17983842	cassava4.1_018335m	photosystem I subunit O	G
cassava4.1_018337m|PACid:17979442	cassava4.1_018337m	ADP-ribosylation factor family protein	No
cassava4.1_018342m|PACid:17964478	cassava4.1_018342m	Ribosomal protein L35	G
cassava4.1_018348m|PACid:17979172	cassava4.1_018348m	Heavy metal transport/detoxification superfamily protein	No
cassava4.1_018350m|PACid:17962298	cassava4.1_018350m	mRNA splicing factor, thioredoxin-like U5 snRNP	G
cassava4.1_018356m|PACid:17978473	cassava4.1_018356m	Late embryogenesis abundant protein	G
cassava4.1_018357m|PACid:17970377	cassava4.1_018357m	Heavy metal transport/detoxification superfamily protein	No
cassava4.1_018359m|PACid:17993491	cassava4.1_018359m	calmodulin-like 11	G
cassava4.1_018362m|PACid:17983868	cassava4.1_018362m	Ribosomal protein S11 family protein	No
cassava4.1_018369m|PACid:17971853	cassava4.1_018369m	Heavy metal transport/detoxification superfamily protein	G
cassava4.1_018377m|PACid:17980948	cassava4.1_018377m	PDI-like 5-1	GP
cassava4.1_018383m|PACid:17993618	cassava4.1_018383m	calmodulin 5	G
cassava4.1_018386m|PACid:17986122	cassava4.1_018386m	histone H2A 12	G
cassava4.1_018387m|PACid:17993208	cassava4.1_018387m	Polynucleotidyl transferase, ribonuclease H-like superfamily protein	G
cassava4.1_018392m|PACid:17981424	cassava4.1_018392m	Cox19-like CHCH family protein	G
cassava4.1_018393m|PACid:17962693	cassava4.1_018393m	nudix hydrolase 1	No
cassava4.1_018398m|PACid:17960435	cassava4.1_018398m	Ribosomal protein L27 family protein	G
cassava4.1_018400m|PACid:17990743	cassava4.1_018400m	Ribosomal protein L18ae family	G
cassava4.1_018407m|PACid:17983188	cassava4.1_018407m	Yippee family putative zinc-binding protein	No
cassava4.1_018412m|PACid:17977300	cassava4.1_018412m	Calcium-dependent lipid-binding (CaLB domain) family protein	No
cassava4.1_018415m|PACid:17972798	cassava4.1_018415m	Eukaryotic protein of unknown function (DUF842)	No
cassava4.1_018423m|PACid:17989929	cassava4.1_018423m	ubiquitin-conjugating enzyme 10	No
cassava4.1_018427m|PACid:17989928	cassava4.1_018427m	ubiquitin-conjugating enzyme 10	No
cassava4.1_018430m|PACid:17970120	cassava4.1_018430m	haemoglobin 2	No
cassava4.1_018436m|PACid:17988653	cassava4.1_018436m	Nucleoside diphosphate kinase family protein	GP
cassava4.1_018439m|PACid:17965971	cassava4.1_018439m	ubiquitin-conjugating enzyme 28	G
cassava4.1_018440m|PACid:17990301	cassava4.1_018440m	ubiquitin-conjugating enzyme 28	G
cassava4.1_018445m|PACid:17981497	cassava4.1_018445m	Small nuclear ribonucleoprotein family protein	No
cassava4.1_018461m|PACid:17971386	cassava4.1_018461m	ubiquitin-conjugating enzyme 30	G
cassava4.1_018463m|PACid:17961295	cassava4.1_018463m	ubiquitin-conjugating enzyme 28	No
cassava4.1_018471m|PACid:17992083	cassava4.1_018471m	Ribosomal protein S5 domain 2-like superfamily protein	GP
cassava4.1_018485m|PACid:17992894	cassava4.1_018485m	NTF2-like	GP
cassava4.1_018486m|PACid:17962904	cassava4.1_018486m	SNARE-like superfamily protein	G
cassava4.1_018487m|PACid:17976223	cassava4.1_018487m	SIN3 associated polypeptide P18	G

cassava4.1_018491m|PACid:17983182	cassava4.1_018491m	Pleckstrin homology (PH) domain superfamily protein	G
cassava4.1_018493m|PACid:17977279	cassava4.1_018493m	Calcium-binding EF-hand family protein	GP
cassava4.1_018495m|PACid:17984689	cassava4.1_018495m		No
cassava4.1_018497m|PACid:17962461	cassava4.1_018497m	2Fe-2S ferredoxin-like superfamily protein	G
cassava4.1_018503m|PACid:17970946	cassava4.1_018503m	RNA polymerase Rpb8	No
cassava4.1_018504m|PACid:17964952	cassava4.1_018504m	RmlC-like cupins superfamily protein	G
cassava4.1_018506m|PACid:17968355	cassava4.1_018506m	actin depolymerizing factor 6	No
cassava4.1_018520m|PACid:17973046	cassava4.1_018520m	Ubiquitin-conjugating enzyme family protein	G
cassava4.1_018524m|PACid:17974122	cassava4.1_018524m	Ribosomal protein L18e/L15 superfamily protein	GP
cassava4.1_018528m|PACid:17969656	cassava4.1_018528m		G
cassava4.1_018529m|PACid:17964341	cassava4.1_018529m		GP
cassava4.1_018530m|PACid:17992347	cassava4.1_018530m	ubiquitin E2 variant 1D-4	P
cassava4.1_018531m|PACid:17993745	cassava4.1_018531m	Translation protein SH3-like family protein	G
cassava4.1_018539m|PACid:17990186	cassava4.1_018539m	high mobility group B2	No
cassava4.1_018541m|PACid:17975973	cassava4.1_018541m	Ribosomal L28e protein family	No
cassava4.1_018546m|PACid:17976766	cassava4.1_018546m	HIS triad family protein 3	GP
cassava4.1_018549m|PACid:17973514	cassava4.1_018549m	G10 family protein	G
cassava4.1_018561m|PACid:17982933	cassava4.1_018561m	Histone superfamily protein	GP
cassava4.1_018563m|PACid:17966146	cassava4.1_018563m	Nucleic acid-binding, OB-fold-like protein	GP
cassava4.1_018569m|PACid:17966600	cassava4.1_018569m	histone H2A 7	No
cassava4.1_018572m|PACid:17969210	cassava4.1_018572m	FKBP-like peptidyl-prolyl cis-trans isomerase family protein	GP
cassava4.1_018580m|PACid:17968304	cassava4.1_018580m	SAUR-like auxin-responsive protein family	G
cassava4.1_018584m|PACid:17983716	cassava4.1_018584m	photosystem I subunit H-1	No
cassava4.1_018586m|PACid:17974842	cassava4.1_018586m	Nucleic acid-binding, OB-fold-like protein	G
cassava4.1_018587m|PACid:17962260	cassava4.1_018587m	2Fe-2S ferredoxin-like superfamily protein	G
cassava4.1_018593m|PACid:17983487	cassava4.1_018593m	Nucleic acid-binding, OB-fold-like protein	G
cassava4.1_018605m|PACid:17980089	cassava4.1_018605m		G
cassava4.1_018608m|PACid:17969618	cassava4.1_018608m	actin depolymerizing factor 5	No
cassava4.1_018609m|PACid:17983530	cassava4.1_018609m	Ribosomal protein S19e family protein	GP
cassava4.1_018614m|PACid:17989690	cassava4.1_018614m	Ribosomal protein S19e family protein	GP
cassava4.1_018616m|PACid:17969765	cassava4.1_018616m	Calcium-binding EF-hand family protein	GP
cassava4.1_018619m|PACid:17965059	cassava4.1_018619m	HVA22 homologue E	G
cassava4.1_018628m|PACid:17961876	cassava4.1_018628m	GRIM-19 protein	GP
cassava4.1_018630m|PACid:17990353	cassava4.1_018630m	basic region/leucine zipper motif 53	G
cassava4.1_018631m|PACid:17966911	cassava4.1_018631m	SNARE-like superfamily protein	G
cassava4.1_018633m|PACid:17985516	cassava4.1_018633m	RNA polymerase Rpb6	G
cassava4.1_018636m|PACid:17965058	cassava4.1_018636m	HVA22 homologue E	No
cassava4.1_018640m|PACid:17991938	cassava4.1_018640m	pleckstrin homologue 1	GP
cassava4.1_018642m|PACid:17971985	cassava4.1_018642m	HMG-box (high mobility group) DNA-binding family protein	No

cassava4.1_018643m|PACid:17985730	cassava4.1_018643m	SNARE-like superfamily protein	GP
cassava4.1_018651m|PACid:17982225	cassava4.1_018651m		GP
cassava4.1_018653m|PACid:17982122	cassava4.1_018653m	Ribosomal protein L18ae family	No
cassava4.1_018665m|PACid:17990958	cassava4.1_018665m	mRNA splicing factor, thioredoxin-like U5 snRNP	G
cassava4.1_018667m|PACid:17990049	cassava4.1_018667m	Protein of unknown function (DUF59)	G
cassava4.1_018673m|PACid:17990568	cassava4.1_018673m	Ribosomal protein S12/S23 family protein	G
cassava4.1_018674m|PACid:17982879	cassava4.1_018674m	multiprotein bridging factor 1B	GP
cassava4.1_018675m|PACid:17972802	cassava4.1_018675m	dual specificity protein phosphatase-related	G
cassava4.1_018679m|PACid:17990205	cassava4.1_018679m	SNARE-like superfamily protein	No
cassava4.1_018692m|PACid:17979061	cassava4.1_018692m	RNA-binding (RRM/RBD/RNP motifs) family protein	G
cassava4.1_018698m|PACid:17989696	cassava4.1_018698m	acclimation of photosynthesis to environment	G
cassava4.1_018706m|PACid:17960192	cassava4.1_018706m	SNARE-like superfamily protein	No
cassava4.1_018725m|PACid:17961689	cassava4.1_018725m	gamma histone variant H2AX	No
cassava4.1_018732m|PACid:17964942	cassava4.1_018732m	Ribosomal protein L7Ae/L30e/S12e/Gadd45 family protein	G
cassava4.1_018739m|PACid:17979864	cassava4.1_018739m	Nucleic acid-binding, OB-fold-like protein	No
cassava4.1_018741m|PACid:17993348	cassava4.1_018741m	Ribosomal protein L14p/L23e family protein	G
cassava4.1_018744m|PACid:17977147	cassava4.1_018744m	Got1/Sft2-like vescicle transport protein family	No
cassava4.1_018753m|PACid:17961679	cassava4.1_018753m	actin depolymerizing factor 1	No
cassava4.1_018755m|PACid:17961730	cassava4.1_018755m	SWIB/MDM2 domain superfamily protein	GP
cassava4.1_018756m|PACid:17973443	cassava4.1_018756m	photosystem II subunit R	G
cassava4.1_018765m|PACid:17980312	cassava4.1_018765m	basic helix-loop-helix (bHLH) DNA-binding superfamily protein	G
cassava4.1_018767m|PACid:17977722	cassava4.1_018767m	Ribosomal protein L7Ae/L30e/S12e/Gadd45 family protein	G
cassava4.1_018776m|PACid:17981618	cassava4.1_018776m	Small nuclear ribonucleoprotein family protein	No
cassava4.1_018779m|PACid:17959913	cassava4.1_018779m	TBP-associated factor II 15	G
cassava4.1_018780m|PACid:17980003	cassava4.1_018780m	Ribosomal L32p protein family	G
cassava4.1_018781m|PACid:17972867	cassava4.1_018781m	phy rapidly regulated 1	No
cassava4.1_018783m|PACid:17986120	cassava4.1_018783m	actin depolymerizing factor 4	GP
cassava4.1_018784m|PACid:17979001	cassava4.1_018784m	Iron-sulphur cluster biosynthesis family protein	G
cassava4.1_018786m|PACid:17966036	cassava4.1_018786m	RNA polymerase II, Rpb4, core protein	G
cassava4.1_018787m|PACid:17965698	cassava4.1_018787m	thioredoxin H-type 9	GP
cassava4.1_018792m|PACid:17967504	cassava4.1_018792m	glycine-rich RNA-binding protein 2	GP
cassava4.1_018797m|PACid:17974460	cassava4.1_018797m	Glutaredoxin family protein	GP
cassava4.1_018806m|PACid:17982429	cassava4.1_018806m	glycine-rich RNA-binding protein 2	GP
cassava4.1_018811m|PACid:17979959	cassava4.1_018811m	glycine-rich protein	No
cassava4.1_018813m|PACid:17966702	cassava4.1_018813m	Cornichon family protein	G
cassava4.1_018826m|PACid:17987949	cassava4.1_018826m	Nucleic acid-binding, OB-fold-like protein	No
cassava4.1_018829m|PACid:17992842	cassava4.1_018829m	Nucleic acid-binding, OB-fold-like protein	GP
cassava4.1_018839m|PACid:17965866	cassava4.1_018839m	Ribosomal protein S24e family protein	No
cassava4.1_018849m|PACid:17982343	cassava4.1_018849m		No

cassava4.1_018852m|PACid:17988675	cassava4.1_018852m	Histone superfamily protein	GP
cassava4.1_018856m|PACid:17977421	cassava4.1_018856m	signal recognition particle 19 kDa protein, putative / SRP19, putative	GP
cassava4.1_018859m|PACid:17981973	cassava4.1_018859m	Chaperone DnaJ-domain superfamily protein	No
cassava4.1_018865m|PACid:17963527	cassava4.1_018865m	Ribosomal protein S16 family protein	G
cassava4.1_018866m|PACid:17963848	cassava4.1_018866m	histone H2A 8	No
cassava4.1_018867m|PACid:17986293	cassava4.1_018867m	histone H2A 11	G
cassava4.1_018872m|PACid:17971346	cassava4.1_018872m	Histone superfamily protein	P
cassava4.1_018873m|PACid:17964258	cassava4.1_018873m		G
cassava4.1_018885m|PACid:17984410	cassava4.1_018885m	sigma factor binding protein 1	G
cassava4.1_018894m|PACid:17981476	cassava4.1_018894m	HSP20-like chaperones superfamily protein	G
cassava4.1_018897m|PACid:17992371	cassava4.1_018897m	Ribosomal L27e protein family	P
cassava4.1_018900m|PACid:17974468	cassava4.1_018900m	Ribosomal L27e protein family	GP
cassava4.1_018904m|PACid:17988878	cassava4.1_018904m	GroES-like family protein	GP
cassava4.1_018905m|PACid:17985177	cassava4.1_018905m	ARP2/3 complex 16 kDa subunit (p16-Arc)	G
cassava4.1_018909m|PACid:17965771	cassava4.1_018909m	SWIB/MDM2 domain superfamily protein	GP
cassava4.1_018911m|PACid:17989831	cassava4.1_018911m	hydroxyproline-rich glycoprotein family protein	G
cassava4.1_018915m|PACid:17973232	cassava4.1_018915m	D111/G-patch domain-containing protein	No
cassava4.1_018918m|PACid:17972106	cassava4.1_018918m	Thioredoxin superfamily protein	G
cassava4.1_018920m|PACid:17984860	cassava4.1_018920m	histone H2A protein 9	GP
cassava4.1_018922m|PACid:17983673	cassava4.1_018922m	histone H2A protein 9	No
cassava4.1_018932m|PACid:17988254	cassava4.1_018932m	cytochrome B5 isoform E	No
cassava4.1_018935m|PACid:17994036	cassava4.1_018935m	Dihydroneopterin aldolase	No
cassava4.1_018936m|PACid:17987741	cassava4.1_018936m	histone H2A 10	GP
cassava4.1_018941m|PACid:17980387	cassava4.1_018941m	mediator 21	No
cassava4.1_018955m|PACid:17982435	cassava4.1_018955m	cytochrome B5 isoform B	GP
cassava4.1_018957m|PACid:17983368	cassava4.1_018957m		No
cassava4.1_018959m|PACid:17982525	cassava4.1_018959m	Rhodanese/Cell cycle control phosphatase superfamily protein	G
cassava4.1_018965m|PACid:17961781	cassava4.1_018965m	Ribosomal protein L14	P
cassava4.1_018973m|PACid:17990980	cassava4.1_018973m		GP
cassava4.1_018974m|PACid:17986990	cassava4.1_018974m	Ribosomal protein L32e	GP
cassava4.1_018992m|PACid:17979265	cassava4.1_018992m	Glutaredoxin family protein	G
cassava4.1_019004m|PACid:17964260	cassava4.1_019004m	profilin 5	No
cassava4.1_019007m|PACid:17960173	cassava4.1_019007m	Ribosomal protein S26e family protein	GP
cassava4.1_019018m|PACid:17974302	cassava4.1_019018m	Thioredoxin superfamily protein	GP
cassava4.1_019020m|PACid:17984362	cassava4.1_019020m	LYR family of Fe/S cluster biogenesis protein	GP
cassava4.1_019034m|PACid:17969776	cassava4.1_019034m		G
cassava4.1_019037m|PACid:17964229	cassava4.1_019037m	profilin 3	GP
cassava4.1_019041m|PACid:17990178	cassava4.1_019041m	Small nuclear ribonucleoprotein family protein	GP
cassava4.1_019048m|PACid:17970116	cassava4.1_019048m	serine protease inhibitor, Kazal-type family protein	No

cassava4.1_019049m|PACid:17982558	cassava4.1_019049m		No
cassava4.1_019054m|PACid:17985766	cassava4.1_019054m	profilin 1	G
cassava4.1_019059m|PACid:17985047	cassava4.1_019059m		GP
cassava4.1_019066m|PACid:17977870	cassava4.1_019066m	mitochondrial acyl carrier protein 2	GP
cassava4.1_019073m|PACid:17961796	cassava4.1_019073m	Target SNARE coiled-coil domain protein	No
cassava4.1_019080m|PACid:17985794	cassava4.1_019080m	Alba DNA/RNA-binding protein	GP
cassava4.1_019083m|PACid:17989481	cassava4.1_019083m	ribosomal protein S15A	G
cassava4.1_019092m|PACid:17982242	cassava4.1_019092m	vacuolar ATPase subunit F family protein	GP
cassava4.1_019094m|PACid:17983332	cassava4.1_019094m	prefoldin 6	GP
cassava4.1_019104m|PACid:17985975	cassava4.1_019104m	ribosomal protein S15A E	G
cassava4.1_019106m|PACid:17968494	cassava4.1_019106m	homolog of yeast ergosterol28	No
cassava4.1_019110m|PACid:17963807	cassava4.1_019110m	PREFOLDIN 1	GP
cassava4.1_019113m|PACid:17982039	cassava4.1_019113m	Rab5-interacting family protein	No
cassava4.1_019117m|PACid:17970251	cassava4.1_019117m	Yippee family putative zinc-binding protein	G
cassava4.1_019119m|PACid:17966845	cassava4.1_019119m		No
cassava4.1_019125m|PACid:17993087	cassava4.1_019125m	TBP-associated factor 13	G
cassava4.1_019134m|PACid:17984878	cassava4.1_019134m	Ribosomal protein L7Ae/L30e/S12e/Gadd45 family protein	G
cassava4.1_019136m|PACid:17961451	cassava4.1_019136m	Dynein light chain type 1 family protein	G
cassava4.1_019137m|PACid:17973297	cassava4.1_019137m	Small nuclear ribonucleoprotein family protein	GP
cassava4.1_019139m|PACid:17992264	cassava4.1_019139m	Ubiquitin supergroup;Ribosomal protein L40e	G
cassava4.1_019141m|PACid:17985173	cassava4.1_019141m	Ubiquitin-like superfamily protein	No
cassava4.1_019153m|PACid:17972073	cassava4.1_019153m	double-stranded DNA-binding family protein	GP
cassava4.1_019158m|PACid:17964612	cassava4.1_019158m	Ubiquitin-like superfamily protein	G
cassava4.1_019165m|PACid:17979644	cassava4.1_019165m	Ribosomal L22e protein family	No
cassava4.1_019183m|PACid:17965699	cassava4.1_019183m	ABI3-interacting protein 3	No
cassava4.1_019184m|PACid:17990354	cassava4.1_019184m	CP12 domain-containing protein 2	G
cassava4.1_019188m|PACid:17966548	cassava4.1_019188m	sulfiredoxin	G
cassava4.1_019197m|PACid:17971389	cassava4.1_019197m	Proteinase inhibitor, propeptide	GP
cassava4.1_019200m|PACid:17977207	cassava4.1_019200m	replication factor C 2	GP
cassava4.1_019207m|PACid:17967413	cassava4.1_019207m	SAP domain-containing protein	G
cassava4.1_019211m|PACid:17987737	cassava4.1_019211m	Ubiquitin-like superfamily protein	G
cassava4.1_019216m|PACid:17976312	cassava4.1_019216m		GP
cassava4.1_019220m|PACid:17987705	cassava4.1_019220m	Sterile alpha motif (SAM) domain-containing protein	No
cassava4.1_019228m|PACid:17985548	cassava4.1_019228m		G
cassava4.1_019231m|PACid:17968032	cassava4.1_019231m	Ribosomal protein L20	G
cassava4.1_019232m|PACid:17989031	cassava4.1_019232m	mitochondrial acyl carrier protein 1	GP
cassava4.1_019234m|PACid:17986925	cassava4.1_019234m		G
cassava4.1_019241m|PACid:17972908	cassava4.1_019241m	Thioredoxin superfamily protein	No
cassava4.1_019244m|PACid:17989280	cassava4.1_019244m	C-terminal cysteine residue is changed to a serine 1	GP

cassava4.1_019259m|PACid:17968838	cassava4.1_019259m		G
cassava4.1_019268m|PACid:17967779	cassava4.1_019268m	RNA-binding (RRM/RBD/RNP motifs) family protein	G
cassava4.1_019281m|PACid:17985292	cassava4.1_019281m	Ribosomal protein S10p/S20e family protein	GP
cassava4.1_019289m|PACid:17973792	cassava4.1_019289m	Ribosomal L29 family protein	GP
cassava4.1_019294m|PACid:17982184	cassava4.1_019294m	cold, circadian rhythm, and RNA binding 1	GP
cassava4.1_019307m|PACid:17989559	cassava4.1_019307m		G
cassava4.1_019309m|PACid:17961535	cassava4.1_019309m	nuclear transport factor 2B	GP
cassava4.1_019315m|PACid:17976966	cassava4.1_019315m	BET1P/SFT1P-like protein 14A	GP
cassava4.1_019318m|PACid:17983630	cassava4.1_019318m	Cytochrome bd ubiquinol oxidase, 14kDa subunit	GP
cassava4.1_019321m|PACid:17993403	cassava4.1_019321m	proton gradient regulation 5	No
cassava4.1_019325m|PACid:17981268	cassava4.1_019325m	sterol carrier protein 2	GP
cassava4.1_019327m|PACid:17985681	cassava4.1_019327m	cytochrome B5-like protein	No
cassava4.1_019332m|PACid:17977227	cassava4.1_019332m	Ubiquitin-like superfamily protein	G
cassava4.1_019335m|PACid:17975533	cassava4.1_019335m	Calcium-binding EF-hand family protein	G
cassava4.1_019336m|PACid:17991727	cassava4.1_019336m	Ssu72-like family protein	G
cassava4.1_019337m|PACid:17980268	cassava4.1_019337m	Trm112p-like protein	G
cassava4.1_019353m|PACid:17993675	cassava4.1_019353m	Tautomerase/MIF superfamily protein	P
cassava4.1_019357m|PACid:17980254	cassava4.1_019357m		No
cassava4.1_019368m|PACid:17972818	cassava4.1_019368m	ribosomal protein S28	GP
cassava4.1_019377m|PACid:17974293	cassava4.1_019377m	Ribosomal protein L31e family protein	G
cassava4.1_019383m|PACid:17974330	cassava4.1_019383m	Ribosomal protein L31e family protein	No
cassava4.1_019392m|PACid:17964256	cassava4.1_019392m	Ribosomal protein L31e family protein	G
cassava4.1_019396m|PACid:17981853	cassava4.1_019396m	stress enhanced protein 1	G
cassava4.1_019399m|PACid:17978805	cassava4.1_019399m	Ribosomal protein L34e superfamily protein	GP
cassava4.1_019402m|PACid:17976148	cassava4.1_019402m	NFU domain protein 4	GP
cassava4.1_019405m|PACid:17983873	cassava4.1_019405m	copper ion binding	GP
cassava4.1_019409m|PACid:17988428	cassava4.1_019409m	Ribosomal protein L34e superfamily protein	G
cassava4.1_019410m|PACid:17989923	cassava4.1_019410m		G
cassava4.1_019412m|PACid:17980300	cassava4.1_019412m	Signal recognition particle, SRP9/SRP14 subunit	G
cassava4.1_019414m|PACid:17961482	cassava4.1_019414m	Bifunctional inhibitor/lipid-transfer protein/seed storage 2S albumin superfamily protein	GP
cassava4.1_019416m|PACid:17987828	cassava4.1_019416m	Ubiquitin-like superfamily protein	No
cassava4.1_019421m|PACid:17968610	cassava4.1_019421m	zinc finger (C2H2 type) family protein	G
cassava4.1_019424m|PACid:17979673	cassava4.1_019424m	Dynein light chain type 1 family protein	GP
cassava4.1_019432m|PACid:17960597	cassava4.1_019432m	Transmembrane proteins 14C	G
cassava4.1_019439m|PACid:17967493	cassava4.1_019439m		G
cassava4.1_019440m|PACid:17992103	cassava4.1_019440m	mitochondrial ribosomal protein L51/S25/CI-B8 family protein	GP
cassava4.1_019441m|PACid:17991629	cassava4.1_019441m	P-loop containing nucleoside triphosphate hydrolases superfamily protein	No
cassava4.1_019444m|PACid:17966464	cassava4.1_019444m	peptidylprolyl cis/trans isomerase, NIMA-interacting 1	G
cassava4.1_019446m|PACid:17988634	cassava4.1_019446m	Bifunctional inhibitor/lipid-transfer protein/seed storage 2S albumin superfamily protein	G

cassava4.1_019449m|PACid:17987317	cassava4.1_019449m	Rab5-interacting family protein	No
cassava4.1_019456m|PACid:17960491	cassava4.1_019456m	thioredoxin H-type 1	GP
cassava4.1_019459m|PACid:17980548	cassava4.1_019459m	membrane-anchored ubiquitin-fold protein 4 precursor	G
cassava4.1_019461m|PACid:17967402	cassava4.1_019461m	Ubiquitin family protein	No
cassava4.1_019468m|PACid:17983825	cassava4.1_019468m	small ubiquitin-like modifier 2	GP
cassava4.1_019471m|PACid:17979951	cassava4.1_019471m		G
cassava4.1_019472m|PACid:17992394	cassava4.1_019472m	plastid-specific ribosomal protein 4	GP
cassava4.1_019480m|PACid:17971775	cassava4.1_019480m	membrane-anchored ubiquitin-fold protein 6 precursor	G
cassava4.1_019483m|PACid:17984593	cassava4.1_019483m		No
cassava4.1_019485m|PACid:17973928	cassava4.1_019485m		GP
cassava4.1_019500m|PACid:17971009	cassava4.1_019500m	Lactoylglutathione lyase / glyoxalase I family protein	No
cassava4.1_019509m|PACid:17963890	cassava4.1_019509m	5\'-AMP-activated protein kinase beta-2 subunit protein	No
cassava4.1_019515m|PACid:17983532	cassava4.1_019515m	membrane-anchored ubiquitin-fold protein 1 precursor	No
cassava4.1_019519m|PACid:17986769	cassava4.1_019519m	DNA-directed RNA polymerase, RBP11-like	GP
cassava4.1_019528m|PACid:17961130	cassava4.1_019528m	LYR family of Fe/S cluster biogenesis protein	GP
cassava4.1_019536m|PACid:17975962	cassava4.1_019536m	RING-box 1	No
cassava4.1_019538m|PACid:17977157	cassava4.1_019538m	Protein Transporter, Pam16	G
cassava4.1_019539m|PACid:17991524	cassava4.1_019539m	Expressed protein	G
cassava4.1_019550m|PACid:17990393	cassava4.1_019550m	Defender against death (DAD family) protein	No
cassava4.1_019565m|PACid:17960609	cassava4.1_019565m	Tautomerase/MIF superfamily protein	G
cassava4.1_019569m|PACid:17962175	cassava4.1_019569m	RNA polymerases M/15 Kd subunit	G
cassava4.1_019570m|PACid:17975978	cassava4.1_019570m	ELF4-like 4	No
cassava4.1_019573m|PACid:17991753	cassava4.1_019573m	60S acidic ribosomal protein family	No
cassava4.1_019580m|PACid:17990698	cassava4.1_019580m	ELF4-like 4	G
cassava4.1_019583m|PACid:17987260	cassava4.1_019583m	Ubiquitin domain-containing protein	GP
cassava4.1_019588m|PACid:17973486	cassava4.1_019588m	60S acidic ribosomal protein family	GP
cassava4.1_019600m|PACid:17964924	cassava4.1_019600m	Tautomerase/MIF superfamily protein	G
cassava4.1_019601m|PACid:17966641	cassava4.1_019601m	Translation initiation factor SUI1 family protein	GP
cassava4.1_019611m|PACid:17979801	cassava4.1_019611m	Dynein light chain type 1 family protein	No
cassava4.1_019615m|PACid:17992274	cassava4.1_019615m	Vacuolar protein sorting 55 (VPS55) family protein	G
cassava4.1_019616m|PACid:17969232	cassava4.1_019616m	tubulin folding cofactor A (KIESEL)	GP
cassava4.1_019619m|PACid:17963510	cassava4.1_019619m	Ribosomal protein L7Ae/L30e/S12e/Gadd45 family protein	G
cassava4.1_019622m|PACid:17966884	cassava4.1_019622m	Chaperone DnaJ-domain superfamily protein	G
cassava4.1_019624m|PACid:17962486	cassava4.1_019624m	cytochrome c-2	GP
cassava4.1_019626m|PACid:17987763	cassava4.1_019626m	Translation initiation factor SUI1 family protein	GP
cassava4.1_019640m|PACid:17983631	cassava4.1_019640m	Cytochrome bd ubiquinol oxidase, 14kDa subunit	No
cassava4.1_019641m|PACid:17964841	cassava4.1_019641m	FK506-binding protein 12	GP
cassava4.1_019646m|PACid:17989022	cassava4.1_019646m	Ribosomal protein L35Ae family protein	GP
cassava4.1_019647m|PACid:17961748	cassava4.1_019647m	60S acidic ribosomal protein family	No

cassava4.1_019653m|PACid:17972585	cassava4.1_019653m	SAUR-like auxin-responsive protein family	No
cassava4.1_019659m|PACid:17979263	cassava4.1_019659m	HSP20-like chaperones superfamily protein	GP
cassava4.1_019675m|PACid:17962146	cassava4.1_019675m	DNA-binding enhancer protein-related	GP
cassava4.1_019683m|PACid:17984971	cassava4.1_019683m		GP
cassava4.1_019688m|PACid:17975920	cassava4.1_019688m	Uncharacterised protein family (UPF0041)	GP
cassava4.1_019690m|PACid:17990596	cassava4.1_019690m	PHF5-like protein	No
cassava4.1_019698m|PACid:17978605	cassava4.1_019698m	Ribosomal protein L36e family protein	No
cassava4.1_019700m|PACid:17975450	cassava4.1_019700m		No
cassava4.1_019701m|PACid:17968721	cassava4.1_019701m	vacuolar membrane ATPase 10	No
cassava4.1_019703m|PACid:17961749	cassava4.1_019703m	60S acidic ribosomal protein family	No
cassava4.1_019704m|PACid:17975517	cassava4.1_019704m	Ribosomal protein S16 family protein	G
cassava4.1_019705m|PACid:17975700	cassava4.1_019705m	Ribosomal protein L36e family protein	GP
cassava4.1_019714m|PACid:17962737	cassava4.1_019714m		G
cassava4.1_019718m|PACid:17992072	cassava4.1_019718m	heat stable protein 1	P
cassava4.1_019741m|PACid:17991493	cassava4.1_019741m	Uncharacterised protein family (UPF0041)	GP
cassava4.1_019745m|PACid:17971930	cassava4.1_019745m	Transcriptional coactivator/pterin dehydratase	G
cassava4.1_019747m|PACid:17989008	cassava4.1_019747m	Small nuclear ribonucleoprotein family protein	GP
cassava4.1_019754m|PACid:17968453	cassava4.1_019754m	Ribosomal protein S25 family protein	GP
cassava4.1_019766m|PACid:17980892	cassava4.1_019766m	Preprotein translocase Sec, Sec61-beta subunit protein	GP
cassava4.1_019769m|PACid:17992275	cassava4.1_019769m	Nucleic acid-binding, OB-fold-like protein	No
cassava4.1_019772m|PACid:17976393	cassava4.1_019772m	GAST1 protein homolog 4	No
cassava4.1_019781m|PACid:17960799	cassava4.1_019781m	Ribosomal protein L36e family protein	GP
cassava4.1_019792m|PACid:17991957	cassava4.1_019792m	RNApolymerase 14 kDa subunit	G
cassava4.1_019797m|PACid:17993535	cassava4.1_019797m	G-protein gamma subunit 2	G
cassava4.1_019801m|PACid:17990383	cassava4.1_019801m	transcription initiation factor IIA gamma chain / TFIIA-gamma (TFIIA-S)	G
cassava4.1_019804m|PACid:17971439	cassava4.1_019804m	Cox19-like CHCH family protein	GP
cassava4.1_019812m|PACid:17970870	cassava4.1_019812m	Yippee family putative zinc-binding protein	No
cassava4.1_019816m|PACid:17962998	cassava4.1_019816m	Complex I subunit NDUFS6	P
cassava4.1_019824m|PACid:17973540	cassava4.1_019824m	Stress responsive A/B Barrel Domain	GP
cassava4.1_019827m|PACid:17982218	cassava4.1_019827m		GP
cassava4.1_019829m|PACid:17968076	cassava4.1_019829m	Zinc-binding ribosomal protein family protein	G
cassava4.1_019834m|PACid:17969904	cassava4.1_019834m	SAUR-like auxin-responsive protein family	G
cassava4.1_019835m|PACid:17994178	cassava4.1_019835m	PLAC8 family protein	No
cassava4.1_019838m|PACid:17967710	cassava4.1_019838m	Gibberellin-regulated family protein	G
cassava4.1_019856m|PACid:17985087	cassava4.1_019856m	SAUR-like auxin-responsive protein family	G
cassava4.1_019863m|PACid:17988488	cassava4.1_019863m	ssDNA-binding transcriptional regulator	G
cassava4.1_019874m|PACid:17973499	cassava4.1_019874m	Expressed protein	G
cassava4.1_019880m|PACid:17976169	cassava4.1_019880m	Sodium Bile acid symporter family	G
cassava4.1_019885m|PACid:17967861	cassava4.1_019885m	Ubiquitin-like superfamily protein	GP

cassava4.1_019894m|PACid:17966100	cassava4.1_019894m		GP
cassava4.1_019905m|PACid:17990396	cassava4.1_019905m		G
cassava4.1_019906m|PACid:17989100	cassava4.1_019906m		G
cassava4.1_019908m|PACid:17990048	cassava4.1_019908m	co-factor for nitrate, reductase and xanthine dehydrogenase 7	No
cassava4.1_019913m|PACid:17969956	cassava4.1_019913m		No
cassava4.1_019920m|PACid:17965739	cassava4.1_019920m	NADH-ubiquinone oxidoreductase-related	G
cassava4.1_019922m|PACid:17959889	cassava4.1_019922m	enhancer of rudimentary protein, putative	G
cassava4.1_019926m|PACid:17984642	cassava4.1_019926m	NADH-ubiquinone oxidoreductase B18 subunit, putative	GP
cassava4.1_019952m|PACid:17981018	cassava4.1_019952m	SAUR-like auxin-responsive protein family	No
cassava4.1_019956m|PACid:17981597	cassava4.1_019956m	Thioredoxin superfamily protein	G
cassava4.1_019964m|PACid:17975046	cassava4.1_019964m	SWIB/MDM2 domain superfamily protein	No
cassava4.1_019974m|PACid:17985793	cassava4.1_019974m	membrane-associated progesterone binding protein 2	GP
cassava4.1_019983m|PACid:17963247	cassava4.1_019983m	Ubiquitin related modifier 1	G
cassava4.1_019995m|PACid:17967594	cassava4.1_019995m	small ubiquitin-like modifier 1	GP
cassava4.1_019998m|PACid:17974306	cassava4.1_019998m	Small nuclear ribonucleoprotein family protein	No
cassava4.1_020000m|PACid:17974113	cassava4.1_020000m		P
cassava4.1_020012m|PACid:17991888	cassava4.1_020012m	BTB/POZ domain-containing protein	No
cassava4.1_020027m|PACid:17971725	cassava4.1_020027m	NADH-ubiquinone oxidoreductase B8 subunit, putative	GP
cassava4.1_020044m|PACid:17960048	cassava4.1_020044m	Small nuclear ribonucleoprotein family protein	G
cassava4.1_020049m|PACid:17981859	cassava4.1_020049m	chaperonin 10	GP
cassava4.1_020051m|PACid:17983698	cassava4.1_020051m	RHO guanyl-nucleotide exchange factor 11	G
cassava4.1_020084m|PACid:17972142	cassava4.1_020084m	postsynaptic protein-related	No
cassava4.1_020085m|PACid:17960318	cassava4.1_020085m	Zinc-binding ribosomal protein family protein	G
cassava4.1_020099m|PACid:17967777	cassava4.1_020099m	Tim10/DDP family zinc finger protein	GP
cassava4.1_020116m|PACid:17983003	cassava4.1_020116m	Zinc-binding ribosomal protein family protein	No
cassava4.1_020125m|PACid:17970595	cassava4.1_020125m		G
cassava4.1_020126m|PACid:17966621	cassava4.1_020126m	Hypoxia-responsive family protein	GP
cassava4.1_020132m|PACid:17963768	cassava4.1_020132m	60S acidic ribosomal protein family	GP
cassava4.1_020136m|PACid:17960203	cassava4.1_020136m	Plant protein 1589 of unknown function	No
cassava4.1_020140m|PACid:17970276	cassava4.1_020140m	Plant protein 1589 of unknown function	G
cassava4.1_020147m|PACid:17960955	cassava4.1_020147m		No
cassava4.1_020154m|PACid:17989574	cassava4.1_020154m	Cytochrome c oxidase biogenesis protein Cmc1-like	No
cassava4.1_020156m|PACid:17980037	cassava4.1_020156m	homolog of anti-oxidant 1	GP
cassava4.1_020169m|PACid:17973660	cassava4.1_020169m	BolA-like family protein	G
cassava4.1_020187m|PACid:17991267	cassava4.1_020187m	Zinc-binding ribosomal protein family protein	G
cassava4.1_020189m|PACid:17981893	cassava4.1_020189m	Mitochondrial ribosomal protein L27	No
cassava4.1_020191m|PACid:17990116	cassava4.1_020191m		G
cassava4.1_020199m|PACid:17963369	cassava4.1_020199m	Microsomal signal peptidase 12 kDa subunit (SPC12)	No
cassava4.1_020202m|PACid:17977932	cassava4.1_020202m		GP

cassava4.1_020207m|PACid:17962374	cassava4.1_020207m	Small nuclear ribonucleoprotein family protein	G
cassava4.1_020210m|PACid:17966666	cassava4.1_020210m	acyl-CoA-binding protein 6	P
cassava4.1_020247m|PACid:17963266	cassava4.1_020247m		No
cassava4.1_020253m|PACid:17965725	cassava4.1_020253m	Methyltransferase-related protein	G
cassava4.1_020258m|PACid:17992100	cassava4.1_020258m		GP
cassava4.1_020260m|PACid:17965741	cassava4.1_020260m		G
cassava4.1_020269m|PACid:17965460	cassava4.1_020269m		G
cassava4.1_020272m|PACid:17959983	cassava4.1_020272m	translocase of the inner mitochondrial membrane 13	GP
cassava4.1_020279m|PACid:17965527	cassava4.1_020279m	small nuclear ribonucleoprotein F	G
cassava4.1_020282m|PACid:17981446	cassava4.1_020282m	heat shock factor binding protein	G
cassava4.1_020283m|PACid:17988244	cassava4.1_020283m	Small nuclear ribonucleoprotein family protein	GP
cassava4.1_020308m|PACid:17983813	cassava4.1_020308m	Small nuclear ribonucleoprotein family protein	G
cassava4.1_020317m|PACid:17975766	cassava4.1_020317m	Homeodomain-like superfamily protein	G
cassava4.1_020320m|PACid:17980070	cassava4.1_020320m	ribosomal protein S27	GP
cassava4.1_020321m|PACid:17980287	cassava4.1_020321m	selenoprotein family protein	GP
cassava4.1_020327m|PACid:17963593	cassava4.1_020327m	Domain of unknown function (DUF543)	GP
cassava4.1_020336m|PACid:17984723	cassava4.1_020336m	CSL zinc finger domain-containing protein	G
cassava4.1_020346m|PACid:17992812	cassava4.1_020346m	ribosomal protein S27	GP
cassava4.1_020359m|PACid:17989839	cassava4.1_020359m	BRICK1, putative	G
cassava4.1_020373m|PACid:17991917	cassava4.1_020373m	Tim10/DDP family zinc finger protein	No
cassava4.1_020375m|PACid:17982178	cassava4.1_020375m	RAD-like 1	No
cassava4.1_020380m|PACid:17988966	cassava4.1_020380m	NADH-ubiquinone oxidoreductase-related	No
cassava4.1_020381m|PACid:17972644	cassava4.1_020381m		G
cassava4.1_020427m|PACid:17966606	cassava4.1_020427m	Ribosomal protein S21e	No
cassava4.1_020446m|PACid:17982936	cassava4.1_020446m	CDK-subunit 2	No
cassava4.1_020459m|PACid:17960707	cassava4.1_020459m		GP
cassava4.1_020466m|PACid:17991070	cassava4.1_020466m	Protein of unknown function (DUF1138)	GP
cassava4.1_020468m|PACid:17968730	cassava4.1_020468m	dolichol phosphate-mannose biosynthesis regulatory protein-related	G
cassava4.1_020488m|PACid:17971871	cassava4.1_020488m	cytochrome c oxidase 17	No
cassava4.1_020506m|PACid:17980196	cassava4.1_020506m	Protein of unknown function (DUF1138)	No
cassava4.1_020520m|PACid:17975685	cassava4.1_020520m	arabinogalactan protein 20	No
cassava4.1_020540m|PACid:17964995	cassava4.1_020540m	translocase inner membrane subunit 8	G
cassava4.1_020556m|PACid:17960586	cassava4.1_020556m	low-molecular-weight cysteine-rich 69	G
cassava4.1_020566m|PACid:17959724	cassava4.1_020566m	Yos1-like protein	G
cassava4.1_020575m|PACid:17986604	cassava4.1_020575m		G
cassava4.1_020620m|PACid:17963983	cassava4.1_020620m	Low temperature and salt responsive protein family	No
cassava4.1_020661m|PACid:17970967	cassava4.1_020661m	isoprenoid F	G
cassava4.1_020673m|PACid:17981589	cassava4.1_020673m	outer envelope membrane protein 7	G
cassava4.1_020685m|PACid:17986932	cassava4.1_020685m	Cytochrome c	No

cassava4.1_020695m|PACid:17960572	cassava4.1_020695m	AtGCP3 interacting protein 1	No
cassava4.1_020696m|PACid:17961025	cassava4.1_020696m	ubiquinol-cytochrome C reductase UQCRX/QCR9-like family protein	GP
cassava4.1_020702m|PACid:17972451	cassava4.1_020702m	RNA polymerases N / 8 kDa subunit	G
cassava4.1_020707m|PACid:17971575	cassava4.1_020707m	RNA polymerases N / 8 kDa subunit	G
cassava4.1_020718m|PACid:17968448	cassava4.1_020718m		No
cassava4.1_020733m|PACid:17993648	cassava4.1_020733m	ATP synthase epsilon chain, mitochondrial	G
cassava4.1_020750m|PACid:17975916	cassava4.1_020750m		G
cassava4.1_020752m|PACid:17967598	cassava4.1_020752m	ATPase, V0 complex, subunit E	No
cassava4.1_020756m|PACid:17987597	cassava4.1_020756m	Ribosomal protein L39 family protein	G
cassava4.1_020758m|PACid:17982822	cassava4.1_020758m	secE/sec61-gamma protein transport protein	G
cassava4.1_020761m|PACid:17961599	cassava4.1_020761m	Protein of unknown function (DUF 3339)	G
cassava4.1_020763m|PACid:17982075	cassava4.1_020763m	Ribosomal L38e protein family	GP
cassava4.1_020834m|PACid:17991785	cassava4.1_020834m	cytochrome-c oxidases;electron carriers	GP
cassava4.1_020859m|PACid:17966952	cassava4.1_020859m		GP
cassava4.1_020872m|PACid:17963452	cassava4.1_020872m	Eukaryotic rpb5 RNA polymerase subunit family protein	No
cassava4.1_020876m|PACid:17994069	cassava4.1_020876m	TatD related DNase	No
cassava4.1_020894m|PACid:17980058	cassava4.1_020894m	organic cation/carnitine transporter1	No
cassava4.1_020908m|PACid:17961632	cassava4.1_020908m	myb domain protein 5	No
cassava4.1_020951m|PACid:17963230	cassava4.1_020951m	SET domain-containing protein	No
cassava4.1_020957m|PACid:17990122	cassava4.1_020957m	RING/FYVE/PHD zinc finger superfamily protein	No
cassava4.1_020979m|PACid:17964192	cassava4.1_020979m	chromatin protein family	No
cassava4.1_020983m|PACid:17973816	cassava4.1_020983m	Patatin-like phospholipase family protein	No
cassava4.1_020990m|PACid:17971732	cassava4.1_020990m	eukaryotic release factor 1-3	GP
cassava4.1_020994m|PACid:17993132	cassava4.1_020994m	SAUR-like auxin-responsive protein family	No
cassava4.1_021038m|PACid:17990296	cassava4.1_021038m	P-loop containing nucleoside triphosphate hydrolases superfamily protein	No
cassava4.1_021053m|PACid:17971176	cassava4.1_021053m	myb domain protein 24	No
cassava4.1_021056m|PACid:17971351	cassava4.1_021056m	ARM repeat superfamily protein	P
cassava4.1_021057m|PACid:17985187	cassava4.1_021057m	Nucleotide-diphospho-sugar transferase family protein	No
cassava4.1_021067m|PACid:17976448	cassava4.1_021067m	Sec14p-like phosphatidylinositol transfer family protein	No
cassava4.1_021077m|PACid:17994032	cassava4.1_021077m	RHOMBOID-like protein 15	G
cassava4.1_021085m|PACid:17992240	cassava4.1_021085m		No
cassava4.1_021101m|PACid:17985429	cassava4.1_021101m	Tetratricopeptide repeat (TPR)-like superfamily protein	No
cassava4.1_021112m|PACid:17989569	cassava4.1_021112m	cytochrome P450, family 735, subfamily A, polypeptide 1	No
cassava4.1_021115m|PACid:17978062	cassava4.1_021115m	cysteine-rich RLK (RECEPTOR-like protein kinase) 42	No
cassava4.1_021119m|PACid:17990709	cassava4.1_021119m	HXXXD-type acyl-transferase family protein	No
cassava4.1_021122m|PACid:17991844	cassava4.1_021122m	RNA-dependent RNA polymerase 2	No
cassava4.1_021137m|PACid:17977544	cassava4.1_021137m	ACT domain-containing small subunit of acetolactate synthase protein	P
cassava4.1_021141m|PACid:17978837	cassava4.1_021141m	SPX domain gene 4	G
cassava4.1_021166m|PACid:17967165	cassava4.1_021166m	12-oxophytodienoate reductase 2	No

cassava4.1_021180m|PACid:17969342	cassava4.1_021180m	P-loop containing nucleoside triphosphate hydrolases superfamily protein	No
cassava4.1_021189m|PACid:17975139	cassava4.1_021189m	Serinc-domain containing serine and sphingolipid biosynthesis protein	No
cassava4.1_021196m|PACid:17968159	cassava4.1_021196m	calcium-dependent protein kinase 24	No
cassava4.1_021204m|PACid:17967338	cassava4.1_021204m	cytochrome P450, family 94, subfamily B, polypeptide 3	No
cassava4.1_021244m|PACid:17964543	cassava4.1_021244m	peroxin 11B	No
cassava4.1_021248m|PACid:17979662	cassava4.1_021248m	TRICHOME BIREFRINGENCE-LIKE 4	No
cassava4.1_021269m|PACid:17991276	cassava4.1_021269m	alpha/beta-Hydrolases superfamily protein	G
cassava4.1_021278m|PACid:17961935	cassava4.1_021278m	basic helix-loop-helix (bHLH) DNA-binding superfamily protein	No
cassava4.1_021280m|PACid:17985107	cassava4.1_021280m	zinc finger protein 7	No
cassava4.1_021283m|PACid:17992718	cassava4.1_021283m	ankyrin repeat family protein	G
cassava4.1_021296m|PACid:17987529	cassava4.1_021296m	basic leucine-zipper 42	No
cassava4.1_021299m|PACid:17993432	cassava4.1_021299m	Uncharacterised protein family (UPF0497)	No
cassava4.1_021305m|PACid:17961080	cassava4.1_021305m	Pentatricopeptide repeat (PPR) superfamily protein	No
cassava4.1_021312m|PACid:17983398	cassava4.1_021312m	exocyst complex component 84B	No
cassava4.1_021327m|PACid:17980770	cassava4.1_021327m	DNA/RNA polymerases superfamily protein	No
cassava4.1_021329m|PACid:17963692	cassava4.1_021329m	Haloacid dehalogenase-like hydrolase (HAD) superfamily protein	No
cassava4.1_021334m|PACid:17967102	cassava4.1_021334m	3-beta hydroxysteroid dehydrogenase/isomerase family protein	No
cassava4.1_021348m|PACid:17971608	cassava4.1_021348m	Concanavalin A-like lectin protein kinase family protein	No
cassava4.1_021365m|PACid:17971182	cassava4.1_021365m	beta galactosidase 1	No
cassava4.1_021385m|PACid:17979078	cassava4.1_021385m	nudix hydrolase homolog 20	GP
cassava4.1_021390m|PACid:17971979	cassava4.1_021390m	cellulose synthase like D4	No
cassava4.1_021394m|PACid:17961601	cassava4.1_021394m	DNA-binding HORMA family protein	No
cassava4.1_021417m|PACid:17990209	cassava4.1_021417m	SAUR-like auxin-responsive protein family	No
cassava4.1_021426m|PACid:17973426	cassava4.1_021426m	NAD(H) kinase 3	No
cassava4.1_021427m|PACid:17990554	cassava4.1_021427m	metaxin-related	G
cassava4.1_021430m|PACid:17973894	cassava4.1_021430m	Tetratricopeptide repeat (TPR)-like superfamily protein	No
cassava4.1_021443m|PACid:17969611	cassava4.1_021443m	Oxoglutarate/iron-dependent oxygenase	No
cassava4.1_021447m|PACid:17978869	cassava4.1_021447m	Tetratricopeptide repeat (TPR)-like superfamily protein	No
cassava4.1_021460m|PACid:17966355	cassava4.1_021460m	Leucine-rich receptor-like protein kinase family protein	No
cassava4.1_021465m|PACid:17968858	cassava4.1_021465m	O-Glycosyl hydrolases family 17 protein	No
cassava4.1_021498m|PACid:17966009	cassava4.1_021498m	Dynamin related protein 4C	No
cassava4.1_021499m|PACid:17962765	cassava4.1_021499m	Adenosylmethionine decarboxylase family protein	G
cassava4.1_021504m|PACid:17963095	cassava4.1_021504m	ATP binding;ATP-dependent helicases;DNA helicases	No
cassava4.1_021520m|PACid:17982747	cassava4.1_021520m	lysine decarboxylase family protein	No
cassava4.1_021525m|PACid:17990579	cassava4.1_021525m	Phosphatidic acid phosphatase (PAP2) family protein	GP
cassava4.1_021530m|PACid:17990807	cassava4.1_021530m	syntaxin of plants 124	No
cassava4.1_021532m|PACid:17967116	cassava4.1_021532m	tRNA synthetase class I (I, L, M and V) family protein	No
cassava4.1_021533m|PACid:17976539	cassava4.1_021533m	multidrug resistance-associated protein 1	No
cassava4.1_021536m|PACid:17988365	cassava4.1_021536m	Tetratricopeptide repeat (TPR)-like superfamily protein	No

cassava4.1_021540m|PACid:17985878	cassava4.1_021540m	staurosporin and temperature sensitive 3-like A	No
cassava4.1_021543m|PACid:17975451	cassava4.1_021543m	exocyst subunit exo70 family protein A2	No
cassava4.1_021544m|PACid:17985066	cassava4.1_021544m	ATP binding microtubule motor family protein	No
cassava4.1_021545m|PACid:17965628	cassava4.1_021545m	Malectin/receptor-like protein kinase family protein	No
cassava4.1_021565m|PACid:17981679	cassava4.1_021565m	Glutathione S-transferase family protein	No
cassava4.1_021570m|PACid:17962066	cassava4.1_021570m	Major facilitator superfamily protein	No
cassava4.1_021575m|PACid:17964745	cassava4.1_021575m	nitrate transporter2.5	No
cassava4.1_021583m|PACid:17982338	cassava4.1_021583m	CDK inhibitor P21 binding protein	No
cassava4.1_021587m|PACid:17964595	cassava4.1_021587m	Tetratricopeptide repeat (TPR)-like superfamily protein	No
cassava4.1_021589m|PACid:17959736	cassava4.1_021589m	paramyosin-related	No
cassava4.1_021619m|PACid:17961906	cassava4.1_021619m	pleiotropic drug resistance 9	No
cassava4.1_021638m|PACid:17985659	cassava4.1_021638m	Kelch repeat-containing F-box family protein	No
cassava4.1_021642m|PACid:17980122	cassava4.1_021642m	Regulator of chromosome condensation (RCC1) family protein	No
cassava4.1_021649m|PACid:17977141	cassava4.1_021649m		G
cassava4.1_021657m|PACid:17986307	cassava4.1_021657m	receptor-like kinase in in flowers 3	G
cassava4.1_021677m|PACid:17963803	cassava4.1_021677m	NAD(P)-binding Rossmann-fold superfamily protein	No
cassava4.1_021680m|PACid:17982237	cassava4.1_021680m	ABC transporter family protein	G
cassava4.1_021705m|PACid:17974288	cassava4.1_021705m	MATE efflux family protein	No
cassava4.1_021729m|PACid:17987226	cassava4.1_021729m	P-loop containing nucleoside triphosphate hydrolases superfamily protein	No
cassava4.1_021733m|PACid:17978302	cassava4.1_021733m	Exonuclease family protein	No
cassava4.1_021801m|PACid:17974487	cassava4.1_021801m	Alkaline-phosphatase-like family protein	No
cassava4.1_021804m|PACid:17991007	cassava4.1_021804m	proteasome activating protein 200	GP
cassava4.1_021806m|PACid:17977073	cassava4.1_021806m	root hair specific 19	No
cassava4.1_021807m|PACid:17987538	cassava4.1_021807m	Protein kinase superfamily protein	No
cassava4.1_021813m|PACid:17976975	cassava4.1_021813m	HXXXD-type acyl-transferase family protein	No
cassava4.1_021817m|PACid:17988928	cassava4.1_021817m	Ribosomal protein L23/L15e family protein	No
cassava4.1_021818m|PACid:17986624	cassava4.1_021818m	K-box region and MADS-box transcription factor family protein	No
cassava4.1_021819m|PACid:17971888	cassava4.1_021819m	O-fucosyltransferase family protein	No
cassava4.1_021838m|PACid:17978451	cassava4.1_021838m	helicases;ATP-dependent helicases;nucleic acid binding;ATP binding;DNA-directed DNA polymerases	No
cassava4.1_021841m|PACid:17984499	cassava4.1_021841m	zinc finger WD40 repeat protein 1	GP
cassava4.1_021843m|PACid:17987129	cassava4.1_021843m	Acyl-CoA N-acyltransferase with RING/FYVE/PHD-type zinc finger protein	No
cassava4.1_021848m|PACid:17977688	cassava4.1_021848m		No
cassava4.1_021878m|PACid:17980932	cassava4.1_021878m	Ankyrin repeat family protein	No
cassava4.1_021890m|PACid:17967860	cassava4.1_021890m	Pentatricopeptide repeat (PPR) superfamily protein	No
cassava4.1_021892m|PACid:17972821	cassava4.1_021892m	N-acetylglucosaminylphosphatidylinositol de-N-acetylase family protein	No
cassava4.1_021904m|PACid:17979413	cassava4.1_021904m	Pentatricopeptide repeat (PPR) superfamily protein	No
cassava4.1_021905m|PACid:17983890	cassava4.1_021905m	zinc-finger protein 10	No
cassava4.1_021907m|PACid:17966367	cassava4.1_021907m	Aquaporin-like superfamily protein	No
cassava4.1_021920m|PACid:17963162	cassava4.1_021920m	Early-responsive to dehydration stress protein (ERD4)	No

cassava4.1_021922m|PACid:17984956	cassava4.1_021922m	Ran BP2/NZF zinc finger-like superfamily protein	No
cassava4.1_021935m|PACid:17971681	cassava4.1_021935m	RmlC-like cupins superfamily protein	No
cassava4.1_021953m|PACid:17972383	cassava4.1_021953m	P-loop containing nucleoside triphosphate hydrolases superfamily protein	No
cassava4.1_021987m|PACid:17961753	cassava4.1_021987m	Peroxidase superfamily protein	No
cassava4.1_021997m|PACid:17987198	cassava4.1_021997m	Core-2/I-branching beta-1,6-N-acetylglucosaminyltransferase family protein	No
cassava4.1_022008m|PACid:17991435	cassava4.1_022008m	basal transcription factor complex subunit-related	No
cassava4.1_022023m|PACid:17973722	cassava4.1_022023m		P
cassava4.1_022040m|PACid:17988186	cassava4.1_022040m	carboxypeptidase D, putative	G
cassava4.1_022041m|PACid:17970401	cassava4.1_022041m	homeobox-leucine zipper protein 17	No
cassava4.1_022058m|PACid:17978357	cassava4.1_022058m	Nucleotidylyl transferase superfamily protein	GP
cassava4.1_022068m|PACid:17982363	cassava4.1_022068m	Eukaryotic aspartyl protease family protein	No
cassava4.1_022073m|PACid:17976948	cassava4.1_022073m	beta-D-xylosidase 4	P
cassava4.1_022077m|PACid:17960852	cassava4.1_022077m	VASCULAR-RELATED NAC-DOMAIN 6	G
cassava4.1_022085m|PACid:17979279	cassava4.1_022085m	folate transporter 1	No
cassava4.1_022089m|PACid:17973518	cassava4.1_022089m	NAD+ ADP-ribosyltransferases;NAD+ ADP-ribosyltransferases	No
cassava4.1_022093m|PACid:17967968	cassava4.1_022093m	AGD2-like defense response protein 1	No
cassava4.1_022110m|PACid:17976205	cassava4.1_022110m	UDP-galactose transporter 2	No
cassava4.1_022125m|PACid:17974190	cassava4.1_022125m	RAB GTPase homolog E1B	P
cassava4.1_022132m|PACid:17980591	cassava4.1_022132m	phosphate deficiency response 2	No
cassava4.1_022139m|PACid:17982646	cassava4.1_022139m	poly(A) polymerase 3	G
cassava4.1_022150m|PACid:17973962	cassava4.1_022150m	Cysteine proteinases superfamily protein	No
cassava4.1_022176m|PACid:17975678	cassava4.1_022176m	cytochrome P450, family 703, subfamily A, polypeptide 2	No
cassava4.1_022185m|PACid:17963281	cassava4.1_022185m	calcineurin B subunit-related	No
cassava4.1_022282m|PACid:17967309	cassava4.1_022282m	RNase L inhibitor protein-related	G
cassava4.1_022283m|PACid:17982819	cassava4.1_022283m	Peroxidase superfamily protein	P
cassava4.1_022288m|PACid:17974416	cassava4.1_022288m	response regulator 9	No
cassava4.1_022294m|PACid:17992143	cassava4.1_022294m	ELMO/CED-12 family protein	No
cassava4.1_022298m|PACid:17973345	cassava4.1_022298m	Protein prenylyltransferase superfamily protein	GP
cassava4.1_022299m|PACid:17979859	cassava4.1_022299m	Leucine-rich repeat (LRR) family protein	No
cassava4.1_022305m|PACid:17993567	cassava4.1_022305m	Pentatricopeptide repeat (PPR) superfamily protein	No
cassava4.1_022307m|PACid:17978154	cassava4.1_022307m	Leucine-rich repeat protein kinase family protein	No
cassava4.1_022330m|PACid:17964257	cassava4.1_022330m	xyloglucan endotransglucosylase/hydrolase 26	No
cassava4.1_022350m|PACid:17982249	cassava4.1_022350m	xyloglucan endotransglucosylase/hydrolase 10	P
cassava4.1_022357m|PACid:17962237	cassava4.1_022357m	Tetratricopeptide repeat (TPR)-like superfamily protein	No
cassava4.1_022371m|PACid:17982545	cassava4.1_022371m	RNA-binding (RRM/RBD/RNP motifs) family protein	No
cassava4.1_022383m|PACid:17966179	cassava4.1_022383m	histone-lysine N-methyltransferase ATXR2	G
cassava4.1_022384m|PACid:17973432	cassava4.1_022384m	CAP (Cysteine-rich secretory proteins, Antigen 5, and Pathogenesis-related 1 protein) superfamily protein	No
cassava4.1_022396m|PACid:17979588	cassava4.1_022396m	root hair specific 14	No
cassava4.1_022398m|PACid:17975648	cassava4.1_022398m	WRKY DNA-binding protein 24	No

cassava4.1_022400m|PACid:17990180	cassava4.1_022400m	DnaJ/Hsp40 cysteine-rich domain superfamily protein	No
cassava4.1_022406m|PACid:17986023	cassava4.1_022406m	3-methylcrotonyl-CoA carboxylase	GP
cassava4.1_022413m|PACid:17981121	cassava4.1_022413m	G-box binding factor 1	No
cassava4.1_022422m|PACid:17990223	cassava4.1_022422m	Single hybrid motif superfamily protein	G
cassava4.1_022431m|PACid:17980201	cassava4.1_022431m	elongation factor family protein	No
cassava4.1_022447m|PACid:17990423	cassava4.1_022447m	Major facilitator superfamily protein	G
cassava4.1_022453m|PACid:17977635	cassava4.1_022453m	Pathogenesis-related thaumatin superfamily protein	No
cassava4.1_022460m|PACid:17970554	cassava4.1_022460m	Protein kinase protein with adenine nucleotide alpha hydrolases-like domain	No
cassava4.1_022469m|PACid:17981733	cassava4.1_022469m	sequence-specific DNA binding transcription factors	No
cassava4.1_022477m|PACid:17962341	cassava4.1_022477m	Glycosyl hydrolases family 31 protein	No
cassava4.1_022495m|PACid:17986842	cassava4.1_022495m	molybdopterin biosynthesis CNX1 protein / molybdenum cofactor biosynthesis enzyme CNX1 (CNX1)	GP
cassava4.1_022502m|PACid:17972581	cassava4.1_022502m	CDPK-related kinase 3	No
cassava4.1_022525m|PACid:17993538	cassava4.1_022525m	translocase of outer membrane 22-V	No
cassava4.1_022528m|PACid:17982777	cassava4.1_022528m	NAD(P)-linked oxidoreductase superfamily protein	No
cassava4.1_022536m|PACid:17973658	cassava4.1_022536m	O-fucosyltransferase family protein	No
cassava4.1_022550m|PACid:17965360	cassava4.1_022550m	cyclic nucleotide-gated channel 16	No
cassava4.1_022570m|PACid:17973917	cassava4.1_022570m	AGAMOUS-like 80	No
cassava4.1_022572m|PACid:17966447	cassava4.1_022572m	HOPZ-ACTIVATED RESISTANCE 1	No
cassava4.1_022575m|PACid:17976548	cassava4.1_022575m	Protein kinase superfamily protein	No
cassava4.1_022578m|PACid:17977514	cassava4.1_022578m	Protein of unknown function (DUF679)	No
cassava4.1_022585m|PACid:17969220	cassava4.1_022585m	Homeodomain-like superfamily protein	G
cassava4.1_022592m|PACid:17978975	cassava4.1_022592m	inositol polyphosphate 5-phosphatase 11	No
cassava4.1_022599m|PACid:17967873	cassava4.1_022599m	TRICHOME BIREFRINGENCE-LIKE 39	No
cassava4.1_022621m|PACid:17990121	cassava4.1_022621m	Transducin/WD40 repeat-like superfamily protein	G
cassava4.1_022626m|PACid:17965756	cassava4.1_022626m	GDSL-like Lipase/Acylhydrolase superfamily protein	No
cassava4.1_022634m|PACid:17983333	cassava4.1_022634m	Molecular chaperone Hsp40/DnaJ family protein	GP
cassava4.1_022665m|PACid:17982681	cassava4.1_022665m	ascorbate peroxidase 4	No
cassava4.1_022693m|PACid:17976249	cassava4.1_022693m	AGAMOUS-like 6	No
cassava4.1_022697m|PACid:17968357	cassava4.1_022697m	Subtilase family protein	No
cassava4.1_022708m|PACid:17959909	cassava4.1_022708m		No
cassava4.1_022710m|PACid:17984304	cassava4.1_022710m	basic helix-loop-helix (bHLH) DNA-binding family protein	No
cassava4.1_022750m|PACid:17980258	cassava4.1_022750m	K-box region and MADS-box transcription factor family protein	No
cassava4.1_022757m|PACid:17977246	cassava4.1_022757m	cytochrome P450, family 87, subfamily A, polypeptide 2	No
cassava4.1_022761m|PACid:17966330	cassava4.1_022761m	NAD(P)-binding Rossmann-fold superfamily protein	No
cassava4.1_022780m|PACid:17970606	cassava4.1_022780m	Calcium-dependent lipid-binding (CaLB domain) plant phosphoribosyltransferase family protein	P
cassava4.1_022781m|PACid:17962092	cassava4.1_022781m	ethylene response factor 1	No
cassava4.1_022788m|PACid:17987172	cassava4.1_022788m	UDP-Glycosyltransferase superfamily protein	No
cassava4.1_022793m|PACid:17986935	cassava4.1_022793m	Exostosin family protein	G
cassava4.1_022796m|PACid:17976300	cassava4.1_022796m	Aldolase superfamily protein	GP

cassava4.1_022803m|PACid:17984827	cassava4.1_022803m	ubiquitin family protein	GP
cassava4.1_022810m|PACid:17972615	cassava4.1_022810m	WUSCHEL related homeobox 1	No
cassava4.1_022835m|PACid:17972414	cassava4.1_022835m	methyltransferases	No
cassava4.1_022841m|PACid:17976215	cassava4.1_022841m	xylem serine peptidase 1	No
cassava4.1_022883m|PACid:17975720	cassava4.1_022883m	beta-amylase 2	No
cassava4.1_022895m|PACid:17979408	cassava4.1_022895m	alpha/beta-Hydrolases superfamily protein	No
cassava4.1_022897m|PACid:17972892	cassava4.1_022897m	K-box region and MADS-box transcription factor family protein	No
cassava4.1_022898m|PACid:17961007	cassava4.1_022898m	cytochrome P450, family 76, subfamily G, polypeptide 1	No
cassava4.1_022900m|PACid:17984098	cassava4.1_022900m	Pentatricopeptide repeat (PPR) superfamily protein	No
cassava4.1_022902m|PACid:17961727	cassava4.1_022902m	Pentatricopeptide repeat (PPR) superfamily protein	G
cassava4.1_022913m|PACid:17969344	cassava4.1_022913m	yeast YAK1-related gene 1	G
cassava4.1_022942m|PACid:17994040	cassava4.1_022942m	Protein kinase superfamily protein	No
cassava4.1_022945m|PACid:17988491	cassava4.1_022945m	GroES-like zinc-binding alcohol dehydrogenase family protein	No
cassava4.1_022948m|PACid:17964558	cassava4.1_022948m	oleosin 1	No
cassava4.1_022962m|PACid:17993451	cassava4.1_022962m	Leucine-rich repeat (LRR) family protein	No
cassava4.1_022966m|PACid:17990630	cassava4.1_022966m	methyltransferases	G
cassava4.1_022982m|PACid:17982844	cassava4.1_022982m		GP
cassava4.1_022987m|PACid:17987210	cassava4.1_022987m	ribosomal protein L6 family protein	No
cassava4.1_022999m|PACid:17984604	cassava4.1_022999m	xylem cysteine peptidase 1	No
cassava4.1_023001m|PACid:17964981	cassava4.1_023001m	CBS / octicosapeptide/Phox/Bemp1 (PB1) domains-containing protein	No
cassava4.1_023042m|PACid:17982619	cassava4.1_023042m	Far-red impaired responsive (FAR1) family protein	No
cassava4.1_023047m|PACid:17987154	cassava4.1_023047m	ABC-2 type transporter family protein	No
cassava4.1_023060m|PACid:17976242	cassava4.1_023060m	AT-hook motif nuclear-localized protein 22	No
cassava4.1_023061m|PACid:17980261	cassava4.1_023061m	2-oxoglutarate (2OG) and Fe(II)-dependent oxygenase superfamily protein	No
cassava4.1_023078m|PACid:17973459	cassava4.1_023078m	galacturonic acid kinase	G
cassava4.1_023106m|PACid:17991948	cassava4.1_023106m	RNA helicase, ATP-dependent, SK12/DOB1 protein	No
cassava4.1_023119m|PACid:17963687	cassava4.1_023119m	shaggy-related kinase 11	G
cassava4.1_023135m|PACid:17991879	cassava4.1_023135m	alpha-galactosidase 2	No
cassava4.1_023174m|PACid:17965448	cassava4.1_023174m	NOD26-like intrinsic protein 1;2	No
cassava4.1_023198m|PACid:17982250	cassava4.1_023198m	CAP (Cysteine-rich secretory proteins, Antigen 5, and Pathogenesis-related 1 protein) superfamily protein	No
cassava4.1_023221m|PACid:17993653	cassava4.1_023221m	Chitinase family protein	G
cassava4.1_023224m|PACid:17989600	cassava4.1_023224m	basic leucine-zipper 42	No
cassava4.1_023230m|PACid:17989571	cassava4.1_023230m	Calcium-binding EF-hand family protein	No
cassava4.1_023248m|PACid:17990273	cassava4.1_023248m	cytochrome P450, family 707, subfamily A, polypeptide 4	No
cassava4.1_023253m|PACid:17975136	cassava4.1_023253m	sulfate transporter 3;5	No
cassava4.1_023265m|PACid:17982068	cassava4.1_023265m	electron-transfer flavoprotein:ubiquinone oxidoreductase	GP
cassava4.1_023276m|PACid:17986191	cassava4.1_023276m	2-oxoglutarate (2OG) and Fe(II)-dependent oxygenase superfamily protein	G
cassava4.1_023286m|PACid:17966497	cassava4.1_023286m	pleiotropic drug resistance 3	No
cassava4.1_023292m|PACid:17974604	cassava4.1_023292m	DNA GYRASE A	GP

cassava4.1_023294m|PACid:17959848	cassava4.1_023294m	KNOTTED1-like homeobox gene 3	G
cassava4.1_023309m|PACid:17984104	cassava4.1_023309m	HPT phosphotransmitter 4	No
cassava4.1_023310m|PACid:17987296	cassava4.1_023310m	isopropylmalate dehydrogenase 2	GP
cassava4.1_023323m|PACid:17967725	cassava4.1_023323m	calcium-dependent protein kinase 17	No
cassava4.1_023334m|PACid:17970355	cassava4.1_023334m	glycosyl hydrolase 9A4	No
cassava4.1_023335m|PACid:17978774	cassava4.1_023335m	expansin-like B1	No
cassava4.1_023342m|PACid:17988415	cassava4.1_023342m	Galactosyltransferase family protein	No
cassava4.1_023343m|PACid:17986457	cassava4.1_023343m	Pentatricopeptide repeat (PPR-like) superfamily protein	No
cassava4.1_023355m|PACid:17987314	cassava4.1_023355m	Mitochondrial glycoprotein family protein	No
cassava4.1_023373m|PACid:17980111	cassava4.1_023373m	Flavin-binding monooxygenase family protein	No
cassava4.1_023374m|PACid:17991124	cassava4.1_023374m	MATE efflux family protein	No
cassava4.1_023377m|PACid:17983607	cassava4.1_023377m	GNOM-like 2	No
cassava4.1_023379m|PACid:17988784	cassava4.1_023379m	Eukaryotic aspartyl protease family protein	No
cassava4.1_023380m|PACid:17981022	cassava4.1_023380m	RNAse l inhibitor protein 2	No
cassava4.1_023381m|PACid:17982453	cassava4.1_023381m	HMG (high mobility group) box protein with ARID/BRIGHT DNA-binding domain	No
cassava4.1_023396m|PACid:17963576	cassava4.1_023396m	cycloartenol synthase 1	GP
cassava4.1_023402m|PACid:17964359	cassava4.1_023402m	Peroxidase superfamily protein	P
cassava4.1_023404m|PACid:17982070	cassava4.1_023404m	Calcium-dependent phosphotriesterase superfamily protein	No
cassava4.1_023406m|PACid:17977798	cassava4.1_023406m	Protein of unknown function (DUF569)	No
cassava4.1_023438m|PACid:17978843	cassava4.1_023438m	structural maintenance of chromosome 3	No
cassava4.1_023445m|PACid:17960145	cassava4.1_023445m	kinesin-like protein 1	No
cassava4.1_023446m|PACid:17994164	cassava4.1_023446m	receptor like protein 29	No
cassava4.1_023450m|PACid:17980664	cassava4.1_023450m	Nitrogen regulatory PII-like, alpha/beta	P
cassava4.1_023467m|PACid:17991867	cassava4.1_023467m	Tetratricopeptide repeat (TPR)-like superfamily protein	No
cassava4.1_023471m|PACid:17977978	cassava4.1_023471m	F-box family protein	No
cassava4.1_023472m|PACid:17988446	cassava4.1_023472m	response regulator 3	G
cassava4.1_023479m|PACid:17971515	cassava4.1_023479m	5\'-3\' exonuclease family protein	No
cassava4.1_023483m|PACid:17966103	cassava4.1_023483m	Heavy metal transport/detoxification superfamily protein	No
cassava4.1_023516m|PACid:17971110	cassava4.1_023516m	AGC kinase 1.7	No
cassava4.1_023517m|PACid:17962006	cassava4.1_023517m	MATE efflux family protein	No
cassava4.1_023523m|PACid:17989330	cassava4.1_023523m	Alba DNA/RNA-binding protein	No
cassava4.1_023525m|PACid:17963749	cassava4.1_023525m	pyrophosphorylase 1	No
cassava4.1_023540m|PACid:17966723	cassava4.1_023540m	Nucleotide-sugar transporter family protein	G
cassava4.1_023542m|PACid:17967171	cassava4.1_023542m	Transmembrane amino acid transporter family protein	No
cassava4.1_023544m|PACid:17983353	cassava4.1_023544m	Protein kinase superfamily protein	No
cassava4.1_023548m|PACid:17972658	cassava4.1_023548m	alpha/beta-Hydrolases superfamily protein	No
cassava4.1_023562m|PACid:17961567	cassava4.1_023562m	translocase outer membrane 20-2	P
cassava4.1_023564m|PACid:17978485	cassava4.1_023564m	alpha/beta-Hydrolases superfamily protein	No
cassava4.1_023566m|PACid:17988382	cassava4.1_023566m	PAS/LOV protein B	No

cassava4.1_023574m|PACid:17962265	cassava4.1_023574m	1-cysteine peroxiredoxin 1	No
cassava4.1_023593m|PACid:17972663	cassava4.1_023593m	O-fucosyltransferase family protein	No
cassava4.1_023605m|PACid:17959845	cassava4.1_023605m	Actin-binding FH2 (Formin Homology) protein	No
cassava4.1_023609m|PACid:17972965	cassava4.1_023609m	myb-like HTH transcriptional regulator family protein	No
cassava4.1_023619m|PACid:17980753	cassava4.1_023619m		G
cassava4.1_023622m|PACid:17987749	cassava4.1_023622m	Ras-related small GTP-binding family protein	No
cassava4.1_023656m|PACid:17989035	cassava4.1_023656m	Plant invertase/pectin methylesterase inhibitor superfamily	No
cassava4.1_023663m|PACid:17961002	cassava4.1_023663m	alpha/beta-Hydrolases superfamily protein	No
cassava4.1_023666m|PACid:17983734	cassava4.1_023666m	HD-ZIP IV family of homeobox-leucine zipper protein with lipid-binding START domain	G
cassava4.1_023668m|PACid:17989561	cassava4.1_023668m	receptor serine/threonine kinase, putative	No
cassava4.1_023673m|PACid:17975662	cassava4.1_023673m	3-ketoacyl-CoA synthase 1	G
cassava4.1_023682m|PACid:17969882	cassava4.1_023682m	DNAJ heat shock family protein	No
cassava4.1_023686m|PACid:17974410	cassava4.1_023686m	Calcium-dependent phosphotriesterase superfamily protein	P
cassava4.1_023692m|PACid:17984959	cassava4.1_023692m	Cytochrome P450 superfamily protein	No
cassava4.1_023693m|PACid:17961596	cassava4.1_023693m	Uncharacterized protein family (UPF0016)	G
cassava4.1_023698m|PACid:17974219	cassava4.1_023698m	ABC2 homolog 6	No
cassava4.1_023711m|PACid:17973474	cassava4.1_023711m	Tetratricopeptide repeat (TPR)-like superfamily protein	No
cassava4.1_023720m|PACid:17964080	cassava4.1_023720m	K-box region and MADS-box transcription factor family protein	No
cassava4.1_023743m|PACid:17988029	cassava4.1_023743m	pentatricopeptide (PPR) repeat-containing protein	No
cassava4.1_023749m|PACid:17964016	cassava4.1_023749m		No
cassava4.1_023751m|PACid:17977954	cassava4.1_023751m	EPS15 homology domain 2	No
cassava4.1_023764m|PACid:17977160	cassava4.1_023764m	ATP-dependent RNA helicase, putative	No
cassava4.1_023776m|PACid:17974944	cassava4.1_023776m	Tetratricopeptide repeat (TPR)-like superfamily protein	No
cassava4.1_023783m|PACid:17992292	cassava4.1_023783m	TRICHOME BIREFRINGENCE-LIKE 8	No
cassava4.1_023807m|PACid:17992062	cassava4.1_023807m	Galactose mutarotase-like superfamily protein	No
cassava4.1_023824m|PACid:17981531	cassava4.1_023824m	poly(ADP-ribose) polymerase	No
cassava4.1_023828m|PACid:17993145	cassava4.1_023828m		No
cassava4.1_023836m|PACid:17992029	cassava4.1_023836m	pfkB-like carbohydrate kinase family protein	No
cassava4.1_023848m|PACid:17979918	cassava4.1_023848m	Pentatricopeptide repeat (PPR) superfamily protein	No
cassava4.1_023852m|PACid:17976082	cassava4.1_023852m	DHHC-type zinc finger family protein	No
cassava4.1_023853m|PACid:17990382	cassava4.1_023853m	oligopeptide transporter 2	No
cassava4.1_023861m|PACid:17965147	cassava4.1_023861m	Cytochrome b561/ferric reductase transmembrane protein family	No
cassava4.1_023865m|PACid:17984747	cassava4.1_023865m	response regulator 6	No
cassava4.1_023866m|PACid:17987612	cassava4.1_023866m	HEAT repeat-containing protein	No
cassava4.1_023867m|PACid:17971502	cassava4.1_023867m	TRAM, LAG1 and CLN8 (TLC) lipid-sensing domain containing protein	No
cassava4.1_023869m|PACid:17981395	cassava4.1_023869m	Protein kinase superfamily protein	No
cassava4.1_023874m|PACid:17976560	cassava4.1_023874m	endonuclease 2	No
cassava4.1_023884m|PACid:17983441	cassava4.1_023884m	Protein kinase superfamily protein	No
cassava4.1_023888m|PACid:17981216	cassava4.1_023888m	calcineurin B-like 3	P

cassava4.1_023898m|PACid:17964941	cassava4.1_023898m	Tetrapyrrole (Corrin/Porphyrin) Methylases	No
cassava4.1_023900m|PACid:17970617	cassava4.1_023900m	threonyl-tRNA synthetase, putative / threonine--tRNA ligase, putative	GP
cassava4.1_023907m|PACid:17989005	cassava4.1_023907m	Yippee family putative zinc-binding protein	No
cassava4.1_023925m|PACid:17975789	cassava4.1_023925m	Peptidyl-tRNA hydrolase II (PTH2) family protein	No
cassava4.1_023945m|PACid:17983420	cassava4.1_023945m	FAD-binding Berberine family protein	No
cassava4.1_023953m|PACid:17971796	cassava4.1_023953m		No
cassava4.1_023954m|PACid:17964352	cassava4.1_023954m	RING-H2 group F2A	No
cassava4.1_023955m|PACid:17976197	cassava4.1_023955m	ataurora3	No
cassava4.1_023967m|PACid:17966745	cassava4.1_023967m	Molecular chaperone Hsp40/DnaJ family protein	No
cassava4.1_023971m|PACid:17976133	cassava4.1_023971m	Polynucleotidyl transferase, ribonuclease H-like superfamily protein	G
cassava4.1_023991m|PACid:17991486	cassava4.1_023991m	2-oxoglutarate (2OG) and Fe(II)-dependent oxygenase superfamily protein	G
cassava4.1_023993m|PACid:17972008	cassava4.1_023993m	SKU5 similar 12	No
cassava4.1_023994m|PACid:17986243	cassava4.1_023994m	Signal recognition particle, SRP9/SRP14 subunit	No
cassava4.1_023995m|PACid:17986526	cassava4.1_023995m	LOB domain-containing protein 4	No
cassava4.1_024005m|PACid:17990650	cassava4.1_024005m	FRIGIDA interacting protein 1	No
cassava4.1_024006m|PACid:17980557	cassava4.1_024006m	SKP1/ASK1-interacting protein 2	No
cassava4.1_024017m|PACid:17973418	cassava4.1_024017m		No
cassava4.1_024024m|PACid:17978105	cassava4.1_024024m	DNAse I-like superfamily protein	G
cassava4.1_024031m|PACid:17982378	cassava4.1_024031m	basic helix-loop-helix (bHLH) DNA-binding superfamily protein	No
cassava4.1_024039m|PACid:17985279	cassava4.1_024039m	AGC (cAMP-dependent, cGMP-dependent and protein kinase C) kinase family protein	No
cassava4.1_024042m|PACid:17974451	cassava4.1_024042m	prefoldin 2	GP
cassava4.1_024052m|PACid:17970192	cassava4.1_024052m	myb domain protein 33	No
cassava4.1_024055m|PACid:17986106	cassava4.1_024055m	Dihydropterin pyrophosphokinase / Dihydropteroate synthase	No
cassava4.1_024060m|PACid:17962220	cassava4.1_024060m	sodium hydrogen exchanger 2	No
cassava4.1_024093m|PACid:17994006	cassava4.1_024093m	root hair specific 19	GP
cassava4.1_024105m|PACid:17967540	cassava4.1_024105m	sucrose phosphate synthase 3F	P
cassava4.1_024108m|PACid:17969644	cassava4.1_024108m	Ribosomal protein S13/S18 family	No
cassava4.1_024120m|PACid:17967186	cassava4.1_024120m	Exostosin family protein	No
cassava4.1_024122m|PACid:17986312	cassava4.1_024122m	Protein phosphatase 2C family protein	No
cassava4.1_024137m|PACid:17979328	cassava4.1_024137m	Pentatricopeptide repeat (PPR) superfamily protein	No
cassava4.1_024141m|PACid:17973072	cassava4.1_024141m	DZC (Disease resistance/zinc finger/chromosome condensation-like region) domain containing protein	No
cassava4.1_024143m|PACid:17993213	cassava4.1_024143m	Alpha-1,4-glucan-protein synthase family protein	GP
cassava4.1_024146m|PACid:17982958	cassava4.1_024146m	Pectin lyase-like superfamily protein	GP
cassava4.1_024165m|PACid:17973892	cassava4.1_024165m	5\'-3\' exonuclease family protein	No
cassava4.1_024202m|PACid:17964602	cassava4.1_024202m	Plant invertase/pectin methylesterase inhibitor superfamily protein	No
cassava4.1_024218m|PACid:17989941	cassava4.1_024218m	Protein kinase superfamily protein	No
cassava4.1_024221m|PACid:17974012	cassava4.1_024221m	quinolinate synthase	G
cassava4.1_024232m|PACid:17961985	cassava4.1_024232m	Thioredoxin superfamily protein	No
cassava4.1_024257m|PACid:17980638	cassava4.1_024257m	P-loop containing nucleoside triphosphate hydrolases superfamily protein	No

cassava4.1_024265m|PACid:17982386	cassava4.1_024265m	Protein of unknown function (DUF803)	No
cassava4.1_024267m|PACid:17971098	cassava4.1_024267m	Basic-leucine zipper (bZIP) transcription factor family protein	No
cassava4.1_024275m|PACid:17971164	cassava4.1_024275m		G
cassava4.1_024282m|PACid:17984425	cassava4.1_024282m	Pentatricopeptide repeat (PPR) superfamily protein	No
cassava4.1_024286m|PACid:17978982	cassava4.1_024286m	lysophosphatidyl acyltransferase 4	G
cassava4.1_024295m|PACid:17990099	cassava4.1_024295m	SKU5 similar 18	No
cassava4.1_024300m|PACid:17979040	cassava4.1_024300m	transcription factor jumonji (jmjC) domain-containing protein	G
cassava4.1_024312m|PACid:17972988	cassava4.1_024312m	Ribonuclease III family protein	G
cassava4.1_024313m|PACid:17973672	cassava4.1_024313m	nuclear factor Y, subunit B7	No
cassava4.1_024331m|PACid:17973093	cassava4.1_024331m	Early-responsive to dehydration stress protein (ERD4)	No
cassava4.1_024358m|PACid:17988104	cassava4.1_024358m	MA3 domain-containing protein	No
cassava4.1_024363m|PACid:17991930	cassava4.1_024363m	Glycosyl hydrolase family protein	GP
cassava4.1_024373m|PACid:17972275	cassava4.1_024373m	nine-cis-epoxycarotenoid dioxygenase 5	No
cassava4.1_024415m|PACid:17993878	cassava4.1_024415m	cation exchanger 5	No
cassava4.1_024422m|PACid:17959759	cassava4.1_024422m	HCO3- transporter family	No
cassava4.1_024428m|PACid:17969660	cassava4.1_024428m	Peroxidase superfamily protein	No
cassava4.1_024435m|PACid:17966058	cassava4.1_024435m	NIMA-related serine/threonine kinase 1	No
cassava4.1_024456m|PACid:17985764	cassava4.1_024456m	Leucine-rich repeat (LRR) family protein	No
cassava4.1_024463m|PACid:17991597	cassava4.1_024463m	Nucleotide-sugar transporter family protein	No
cassava4.1_024464m|PACid:17975650	cassava4.1_024464m	RHO guanyl-nucleotide exchange factor 3	No
cassava4.1_024480m|PACid:17974356	cassava4.1_024480m		No
cassava4.1_024504m|PACid:17978991	cassava4.1_024504m	Vacuolar iron transporter (VIT) family protein	No
cassava4.1_024509m|PACid:17990561	cassava4.1_024509m	ascorbate peroxidase 2	GP
cassava4.1_024512m|PACid:17972668	cassava4.1_024512m	DNA ligase IV	No
cassava4.1_024513m|PACid:17976378	cassava4.1_024513m	nitrate transporter 1.1	No
cassava4.1_024523m|PACid:17986667	cassava4.1_024523m	tubby like protein 6	G
cassava4.1_024524m|PACid:17982883	cassava4.1_024524m	RNA methyltransferase family protein	No
cassava4.1_024545m|PACid:17961176	cassava4.1_024545m	vacuolar proton ATPase A3	P
cassava4.1_024562m|PACid:17987555	cassava4.1_024562m	Heavy metal transport/detoxification superfamily protein	No
cassava4.1_024570m|PACid:17985709	cassava4.1_024570m	Eukaryotic rpb5 RNA polymerase subunit family protein	No
cassava4.1_024574m|PACid:17981580	cassava4.1_024574m	ERD (early-responsive to dehydration stress) family protein	No
cassava4.1_024580m|PACid:17989708	cassava4.1_024580m	Voltage-gated chloride channel family protein	No
cassava4.1_024581m|PACid:17969546	cassava4.1_024581m	RAD3-like DNA-binding helicase protein	No
cassava4.1_024597m|PACid:17981471	cassava4.1_024597m	Thioredoxin superfamily protein	No
cassava4.1_024599m|PACid:17978419	cassava4.1_024599m	NAD(P)-binding Rossmann-fold superfamily protein	No
cassava4.1_024608m|PACid:17980550	cassava4.1_024608m	Thioredoxin superfamily protein	No
cassava4.1_024644m|PACid:17960926	cassava4.1_024644m	TRAM, LAG1 and CLN8 (TLC) lipid-sensing domain containing protein	G
cassava4.1_024654m|PACid:17977869	cassava4.1_024654m	NAD(P)-binding Rossmann-fold superfamily protein	No
cassava4.1_024671m|PACid:17983650	cassava4.1_024671m	multiprotein bridging factor 1C	G

cassava4.1_024688m|PACid:17977213	cassava4.1_024688m	expansin A7	No
cassava4.1_024690m|PACid:17980802	cassava4.1_024690m	Sec14p-like phosphatidylinositol transfer family protein	No
cassava4.1_024691m|PACid:17967002	cassava4.1_024691m	Pyridoxamine 5\'-phosphate oxidase family protein	GP
cassava4.1_024698m|PACid:17984227	cassava4.1_024698m	Dihydroxyacetone kinase	GP
cassava4.1_024699m|PACid:17977389	cassava4.1_024699m		No
cassava4.1_024709m|PACid:17978627	cassava4.1_024709m	Calcium-binding EF-hand family protein	No
cassava4.1_024737m|PACid:17965630	cassava4.1_024737m	non-specific phospholipase C4	No
cassava4.1_024740m|PACid:17978904	cassava4.1_024740m	Endosomal targeting BRO1-like domain-containing protein	No
cassava4.1_024742m|PACid:17966312	cassava4.1_024742m	copper transporter 1	No
cassava4.1_024749m|PACid:17992807	cassava4.1_024749m	plant intracellular ras group-related LRR 6	No
cassava4.1_024767m|PACid:17959939	cassava4.1_024767m	ENTH/ANTH/VHS superfamily protein	No
cassava4.1_024776m|PACid:17989725	cassava4.1_024776m	Heavy metal transport/detoxification superfamily protein	No
cassava4.1_024778m|PACid:17971035	cassava4.1_024778m	2-oxoglutarate (2OG) and Fe(II)-dependent oxygenase superfamily protein	No
cassava4.1_024788m|PACid:17987767	cassava4.1_024788m	Transport protein particle (TRAPP) component	No
cassava4.1_024792m|PACid:17988206	cassava4.1_024792m	alpha/beta-Hydrolases superfamily protein	No
cassava4.1_024801m|PACid:17977262	cassava4.1_024801m	KNOTTED-like homeobox of Arabidopsis thaliana 7	G
cassava4.1_024802m|PACid:17984890	cassava4.1_024802m	FAD-binding Berberine family protein	P
cassava4.1_024805m|PACid:17977437	cassava4.1_024805m	inosine-uridine preferring nucleoside hydrolase family protein	No
cassava4.1_024817m|PACid:17961054	cassava4.1_024817m	P-loop containing nucleoside triphosphate hydrolases superfamily protein	No
cassava4.1_024829m|PACid:17988420	cassava4.1_024829m	Vps51/Vps67 family (components of vesicular transport) protein	No
cassava4.1_024841m|PACid:17971348	cassava4.1_024841m	MSF1-like family protein	No
cassava4.1_024900m|PACid:17994085	cassava4.1_024900m	DNAJ heat shock N-terminal domain-containing protein	No
cassava4.1_024910m|PACid:17980223	cassava4.1_024910m	Protein kinase superfamily protein	No
cassava4.1_024927m|PACid:17980026	cassava4.1_024927m	VH1-interacting kinase	No
cassava4.1_024930m|PACid:17976443	cassava4.1_024930m	Lactate/malate dehydrogenase family protein	No
cassava4.1_024955m|PACid:17976027	cassava4.1_024955m	phospholipase D alpha 4	No
cassava4.1_024962m|PACid:17969015	cassava4.1_024962m	Arabidopsis Hop2 homolog	No
cassava4.1_024973m|PACid:17991731	cassava4.1_024973m	O-Glycosyl hydrolases family 17 protein	No
cassava4.1_024979m|PACid:17992905	cassava4.1_024979m	protodermal factor 2	No
cassava4.1_024985m|PACid:17976711	cassava4.1_024985m	SER/ARG-rich protein 34A	GP
cassava4.1_024993m|PACid:17987127	cassava4.1_024993m	Protein of unknown function (DUF616)	No
cassava4.1_025006m|PACid:17975502	cassava4.1_025006m	Cytochrome P450 superfamily protein	No
cassava4.1_025012m|PACid:17968166	cassava4.1_025012m	AAA-type ATPase family protein	No
cassava4.1_025015m|PACid:17971434	cassava4.1_025015m		No
cassava4.1_025019m|PACid:17960268	cassava4.1_025019m	VPS54	No
cassava4.1_025021m|PACid:17985058	cassava4.1_025021m	Leucine-rich receptor-like protein kinase family protein	No
cassava4.1_025023m|PACid:17981187	cassava4.1_025023m	multidrug resistance-associated protein 11	No
cassava4.1_025028m|PACid:17964689	cassava4.1_025028m	Pyridoxal phosphate (PLP)-dependent transferases superfamily protein	No
cassava4.1_025030m|PACid:17973273	cassava4.1_025030m	pleiotropic drug resistance 1	No

cassava4.1_025032m|PACid:17990631	cassava4.1_025032m	armadillo repeat only 1	No
cassava4.1_025043m|PACid:17972857	cassava4.1_025043m	YELLOW STRIPE like 7	No
cassava4.1_025057m|PACid:17968132	cassava4.1_025057m	PEBP (phosphatidylethanolamine-binding protein) family protein	No
cassava4.1_025058m|PACid:17974846	cassava4.1_025058m	RNA helicase family protein	G
cassava4.1_025059m|PACid:17985336	cassava4.1_025059m	ubiquinol-cytochrome C chaperone family protein	GP
cassava4.1_025060m|PACid:17972032	cassava4.1_025060m	Pentatricopeptide repeat (PPR) superfamily protein	No
cassava4.1_025064m|PACid:17979014	cassava4.1_025064m	PEBP (phosphatidylethanolamine-binding protein) family protein	No
cassava4.1_025075m|PACid:17967123	cassava4.1_025075m	PA-domain containing subtilase family protein	No
cassava4.1_025077m|PACid:17970512	cassava4.1_025077m	Ubiquitin carboxyl-terminal hydrolase family protein	G
cassava4.1_025082m|PACid:17985218	cassava4.1_025082m	Chalcone and stilbene synthase family protein	No
cassava4.1_025088m|PACid:17978013	cassava4.1_025088m	alpha/beta-Hydrolases superfamily protein	No
cassava4.1_025096m|PACid:17975686	cassava4.1_025096m	Pentatricopeptide repeat (PPR-like) superfamily protein	No
cassava4.1_025116m|PACid:17966661	cassava4.1_025116m	Domain of unknown function (DUF1995)	GP
cassava4.1_025121m|PACid:17982302	cassava4.1_025121m	D-aminoacid aminotransferase-like PLP-dependent enzymes superfamily protein	No
cassava4.1_025126m|PACid:17981285	cassava4.1_025126m	FGGY family of carbohydrate kinase	GP
cassava4.1_025131m|PACid:17979098	cassava4.1_025131m		No
cassava4.1_025146m|PACid:17974963	cassava4.1_025146m	Amino acid kinase family protein	GP
cassava4.1_025185m|PACid:17959814	cassava4.1_025185m		No
cassava4.1_025197m|PACid:17960471	cassava4.1_025197m	glyoxal oxidase-related protein	No
cassava4.1_025203m|PACid:17993730	cassava4.1_025203m	bZIP transcription factor family protein	No
cassava4.1_025217m|PACid:17986778	cassava4.1_025217m	Plant protein of unknown function (DUF946)	No
cassava4.1_025240m|PACid:17980396	cassava4.1_025240m	cytochrome P450, family 94, subfamily D, polypeptide 2	No
cassava4.1_025250m|PACid:17984339	cassava4.1_025250m	phytochrome interacting factor 3-like 5	No
cassava4.1_025255m|PACid:17989147	cassava4.1_025255m		No
cassava4.1_025264m|PACid:17991117	cassava4.1_025264m	basic helix-loop-helix (bHLH) DNA-binding superfamily protein	No
cassava4.1_025278m|PACid:17963895	cassava4.1_025278m	UDP-Glycosyltransferase superfamily protein	No
cassava4.1_025303m|PACid:17971792	cassava4.1_025303m	general regulatory factor 11	GP
cassava4.1_025314m|PACid:17984061	cassava4.1_025314m	Peroxidase superfamily protein	P
cassava4.1_025320m|PACid:17983360	cassava4.1_025320m	Eukaryotic release factor 1 (eRF1) family protein	G
cassava4.1_025322m|PACid:17986309	cassava4.1_025322m	KOW domain-containing protein	G
cassava4.1_025327m|PACid:17960130	cassava4.1_025327m	photolyase 1	No
cassava4.1_025352m|PACid:17978340	cassava4.1_025352m	Nucleotide-diphospho-sugar transferases superfamily protein	No
cassava4.1_025355m|PACid:17983624	cassava4.1_025355m	1-deoxy-D-xylulose 5-phosphate synthase 3	No
cassava4.1_025367m|PACid:17988316	cassava4.1_025367m	Ras-related small GTP-binding family protein	P
cassava4.1_025372m|PACid:17960695	cassava4.1_025372m	P-loop containing nucleoside triphosphate hydrolases superfamily protein	G
cassava4.1_025386m|PACid:17968095	cassava4.1_025386m	RAB GTPase homolog A6A	No
cassava4.1_025390m|PACid:17975056	cassava4.1_025390m	MUTS-homologue 5	No
cassava4.1_025413m|PACid:17974130	cassava4.1_025413m	GDSL-like Lipase/Acylhydrolase superfamily protein	No
cassava4.1_025431m|PACid:17990091	cassava4.1_025431m	Seven transmembrane MLO family protein	No

cassava4.1_025435m|PACid:17980488	cassava4.1_025435m	Leucine-rich receptor-like protein kinase family protein	No
cassava4.1_025440m|PACid:17973609	cassava4.1_025440m	myb domain protein 69	No
cassava4.1_025447m|PACid:17964461	cassava4.1_025447m	STELAR K+ outward rectifier	No
cassava4.1_025466m|PACid:17971688	cassava4.1_025466m	Galactose oxidase/kelch repeat superfamily protein	G
cassava4.1_025469m|PACid:17994163	cassava4.1_025469m	Pentatricopeptide repeat (PPR-like) superfamily protein	No
cassava4.1_025482m|PACid:17960773	cassava4.1_025482m	pyruvate kinase family protein	No
cassava4.1_025512m|PACid:17972861	cassava4.1_025512m	cytochrome P450, family 718	No
cassava4.1_025539m|PACid:17968497	cassava4.1_025539m	1-amino-cyclopropane-1-carboxylate synthase 8	No
cassava4.1_025555m|PACid:17972296	cassava4.1_025555m	Glycosyl hydrolase superfamily protein	No
cassava4.1_025556m|PACid:17969091	cassava4.1_025556m	Flavin-binding monooxygenase family protein	No
cassava4.1_025576m|PACid:17983917	cassava4.1_025576m	CBL-interacting protein kinase 20	No
cassava4.1_025579m|PACid:17982199	cassava4.1_025579m	RING/U-box superfamily protein	G
cassava4.1_025593m|PACid:17972574	cassava4.1_025593m	ABC-2 type transporter family protein	No
cassava4.1_025611m|PACid:17974184	cassava4.1_025611m	Galactose mutarotase-like superfamily protein	No
cassava4.1_025620m|PACid:17968227	cassava4.1_025620m	GPI transamidase subunit PIG-U	G
cassava4.1_025628m|PACid:17984467	cassava4.1_025628m	ROOT HAIR DEFECTIVE 6-LIKE 2	No
cassava4.1_025662m|PACid:17989479	cassava4.1_025662m	sporulation 11-2	No
cassava4.1_025670m|PACid:17979966	cassava4.1_025670m	lipid phosphate phosphatase 2	No
cassava4.1_025671m|PACid:17993269	cassava4.1_025671m	RECQ helicase l1	No
cassava4.1_025701m|PACid:17989058	cassava4.1_025701m	Matrixin family protein	No
cassava4.1_025703m|PACid:17973403	cassava4.1_025703m	pfkB-like carbohydrate kinase family protein	P
cassava4.1_025710m|PACid:17969643	cassava4.1_025710m	2Fe-2S ferredoxin-like superfamily protein	No
cassava4.1_025723m|PACid:17970507	cassava4.1_025723m	RNA recognition motif and CCHC-type zinc finger domains containing protein	No
cassava4.1_025726m|PACid:17987201	cassava4.1_025726m	phosphoesterase	No
cassava4.1_025727m|PACid:17963968	cassava4.1_025727m	inositol transporter 4	No
cassava4.1_025731m|PACid:17963071	cassava4.1_025731m	alpha/beta-Hydrolases superfamily protein	No
cassava4.1_025734m|PACid:17985954	cassava4.1_025734m	SPFH/Band 7/PHB domain-containing membrane-associated protein family	No
cassava4.1_025742m|PACid:17988388	cassava4.1_025742m	nitrate transporter 1.7	No
cassava4.1_025758m|PACid:17986508	cassava4.1_025758m	oxidoreductase, 2OG-Fe(II) oxygenase family protein	No
cassava4.1_025763m|PACid:17962602	cassava4.1_025763m	phosphatidylserine decarboxylase 2	GP
cassava4.1_025766m|PACid:17961424	cassava4.1_025766m	DNAse I-like superfamily protein	No
cassava4.1_025791m|PACid:17981877	cassava4.1_025791m	expansin A9	No
cassava4.1_025794m|PACid:17963810	cassava4.1_025794m	MUTL-homologue 1	No
cassava4.1_025804m|PACid:17970906	cassava4.1_025804m	Leucine-rich repeat (LRR) family protein	No
cassava4.1_025812m|PACid:17963658	cassava4.1_025812m	FMN-linked oxidoreductases superfamily protein	G
cassava4.1_025822m|PACid:17966083	cassava4.1_025822m	Tetratricopeptide repeat (TPR)-like superfamily protein	No
cassava4.1_025830m|PACid:17984764	cassava4.1_025830m	ATP-dependent caseinolytic (Clp) protease/crotonase family protein	GP
cassava4.1_025834m|PACid:17978215	cassava4.1_025834m	RAN binding protein 1	GP
cassava4.1_025843m|PACid:17986044	cassava4.1_025843m	SEC14-like 3	No

cassava4.1_025856m|PACid:17980879	cassava4.1_025856m	isovaleryl-CoA-dehydrogenase	GP
cassava4.1_025860m|PACid:17980360	cassava4.1_025860m	MATE efflux family protein	No
cassava4.1_025873m|PACid:17993819	cassava4.1_025873m	Nodulin MtN21 /EamA-like transporter family protein	No
cassava4.1_025875m|PACid:17967994	cassava4.1_025875m		GP
cassava4.1_025880m|PACid:17965247	cassava4.1_025880m	Haloacid dehalogenase-like hydrolase (HAD) superfamily protein	No
cassava4.1_025886m|PACid:17963084	cassava4.1_025886m	like SEX4 1	GP
cassava4.1_025932m|PACid:17980417	cassava4.1_025932m	Pre-mRNA cleavage complex II protein family	G
cassava4.1_025938m|PACid:17993366	cassava4.1_025938m	Acyl-CoA N-acyltransferases (NAT) superfamily protein	No
cassava4.1_025952m|PACid:17972745	cassava4.1_025952m	alpha/beta-Hydrolases superfamily protein	No
cassava4.1_025962m|PACid:17988955	cassava4.1_025962m	Plant protein 1589 of unknown function	No
cassava4.1_025965m|PACid:17989507	cassava4.1_025965m	actin 4	No
cassava4.1_025971m|PACid:17983629	cassava4.1_025971m	HCO3- transporter family	No
cassava4.1_025973m|PACid:17980259	cassava4.1_025973m	amino acid permease 3	No
cassava4.1_025980m|PACid:17993407	cassava4.1_025980m	phosphate 1	No
cassava4.1_025987m|PACid:17983320	cassava4.1_025987m	MUTM homolog-1	No
cassava4.1_026021m|PACid:17976368	cassava4.1_026021m	phosphatidylserine decarboxylase 1	No
cassava4.1_026034m|PACid:17983726	cassava4.1_026034m	PLAC8 family protein	No
cassava4.1_026035m|PACid:17968666	cassava4.1_026035m	alcohol dehydrogenase 1	GP
cassava4.1_026051m|PACid:17977268	cassava4.1_026051m	AGAMOUS-like 19	G
cassava4.1_026058m|PACid:17963309	cassava4.1_026058m	nuclear factor Y, subunit C13	No
cassava4.1_026066m|PACid:17990440	cassava4.1_026066m	transferases, transferring glycosyl groups	No
cassava4.1_026071m|PACid:17989370	cassava4.1_026071m	glyoxal oxidase-related protein	No
cassava4.1_026079m|PACid:17991436	cassava4.1_026079m	Cyclin A1;1	No
cassava4.1_026103m|PACid:17983931	cassava4.1_026103m	Pentatricopeptide repeat (PPR) superfamily protein	No
cassava4.1_026109m|PACid:17982728	cassava4.1_026109m	Glutathione S-transferase family protein	GP
cassava4.1_026119m|PACid:17975519	cassava4.1_026119m	NAD(P)-binding Rossmann-fold superfamily protein	No
cassava4.1_026138m|PACid:17963774	cassava4.1_026138m	Pectin lyase-like superfamily protein	No
cassava4.1_026141m|PACid:17974707	cassava4.1_026141m	Pentatricopeptide repeat (PPR) superfamily protein	No
cassava4.1_026170m|PACid:17961260	cassava4.1_026170m	topoisomerase 3alpha	No
cassava4.1_026191m|PACid:17982265	cassava4.1_026191m	autophagy 9 (APG9)	No
cassava4.1_026203m|PACid:17978136	cassava4.1_026203m	alpha-mannosidase 2	G
cassava4.1_026233m|PACid:17975873	cassava4.1_026233m	Protein kinase superfamily protein	G
cassava4.1_026236m|PACid:17983070	cassava4.1_026236m	Transducin/WD40 repeat-like superfamily protein	GP
cassava4.1_026278m|PACid:17984483	cassava4.1_026278m	Ribosomal protein S14p/S29e family protein	G
cassava4.1_026283m|PACid:17973441	cassava4.1_026283m	nine-cis-epoxycarotenoid dioxygenase 3	G
cassava4.1_026292m|PACid:17968977	cassava4.1_026292m	C2H2-like zinc finger protein	No
cassava4.1_026314m|PACid:17980162	cassava4.1_026314m	Seryl-tRNA synthetase	No
cassava4.1_026339m|PACid:17982161	cassava4.1_026339m	basic leucine-zipper 44	No
cassava4.1_026346m|PACid:17968792	cassava4.1_026346m	GATA transcription factor 11	No

cassava4.1_026369m|PACid:17971237	cassava4.1_026369m	RAB GTPase homolog G3A	No
cassava4.1_026378m|PACid:17985583	cassava4.1_026378m	Peroxidase superfamily protein	No
cassava4.1_026381m|PACid:17973624	cassava4.1_026381m	P-loop containing nucleoside triphosphate hydrolases superfamily protein	No
cassava4.1_026393m|PACid:17962484	cassava4.1_026393m	Lactoylglutathione lyase / glyoxalase I family protein	No
cassava4.1_026405m|PACid:17966667	cassava4.1_026405m	Formin Homology 14	No
cassava4.1_026419m|PACid:17990181	cassava4.1_026419m	glutamate receptor 3.3	No
cassava4.1_026425m|PACid:17966633	cassava4.1_026425m	nuclear factor Y, subunit B4	No
cassava4.1_026433m|PACid:17974103	cassava4.1_026433m	transcription regulators;zinc ion binding	G
cassava4.1_026496m|PACid:17962240	cassava4.1_026496m	Glutaredoxin family protein	No
cassava4.1_026511m|PACid:17964022	cassava4.1_026511m	Putative lysine decarboxylase family protein	No
cassava4.1_026517m|PACid:17968907	cassava4.1_026517m	Putative lysine decarboxylase family protein	No
cassava4.1_026535m|PACid:17978773	cassava4.1_026535m	tRNA/rRNA methyltransferase (SpoU) family protein	No
cassava4.1_026548m|PACid:17969090	cassava4.1_026548m	isochorismate synthase 2	No
cassava4.1_026562m|PACid:17972202	cassava4.1_026562m	Xanthine/uracil permease family protein	No
cassava4.1_026567m|PACid:17960243	cassava4.1_026567m	ATP binding microtubule motor family protein	No
cassava4.1_026586m|PACid:17970899	cassava4.1_026586m	Protein kinase superfamily protein	G
cassava4.1_026604m|PACid:17970992	cassava4.1_026604m	Translation initiation factor 2, small GTP-binding protein	No
cassava4.1_026649m|PACid:17961945	cassava4.1_026649m	Protein phosphatase 2C family protein	No
cassava4.1_026670m|PACid:17980608	cassava4.1_026670m	SECY homolog 1	GP
cassava4.1_026680m|PACid:17991246	cassava4.1_026680m	Tetratricopeptide repeat (TPR)-like superfamily protein	G
cassava4.1_026686m|PACid:17988849	cassava4.1_026686m	RP non-ATPase subunit 8A	No
cassava4.1_026687m|PACid:17968152	cassava4.1_026687m	Pentatricopeptide repeat (PPR) superfamily protein	No
cassava4.1_026695m|PACid:17982677	cassava4.1_026695m	myb domain protein 108	No
cassava4.1_026709m|PACid:17985959	cassava4.1_026709m	phospholipase D alpha 1	P
cassava4.1_026712m|PACid:17993701	cassava4.1_026712m	RAB GTPase homolog A4D	No
cassava4.1_026724m|PACid:17980395	cassava4.1_026724m	glutamate-ammonia ligases;catalytics;glutamate-ammonia ligases	No
cassava4.1_026726m|PACid:17978130	cassava4.1_026726m	DHHC-type zinc finger family protein	GP
cassava4.1_026741m|PACid:17989948	cassava4.1_026741m		No
cassava4.1_026742m|PACid:17962780	cassava4.1_026742m	Leucine-rich repeat (LRR) family protein	No
cassava4.1_026744m|PACid:17960935	cassava4.1_026744m		P
cassava4.1_026759m|PACid:17975724	cassava4.1_026759m	NAC (No Apical Meristem) domain transcriptional regulator superfamily protein	No
cassava4.1_026773m|PACid:17991245	cassava4.1_026773m	plant intracellular ras group-related LRR 6	No
cassava4.1_026790m|PACid:17987333	cassava4.1_026790m	Peptidyl-tRNA hydrolase family protein	No
cassava4.1_026802m|PACid:17979499	cassava4.1_026802m	ribonuclease II family protein	P
cassava4.1_026805m|PACid:17973650	cassava4.1_026805m	plant glycogenin-like starch initiation protein 1	No
cassava4.1_026807m|PACid:17985818	cassava4.1_026807m	RAN GTPase 3	No
cassava4.1_026808m|PACid:17968839	cassava4.1_026808m	transportin 1	P
cassava4.1_026812m|PACid:17985203	cassava4.1_026812m	cytidinediphosphate diacylglycerol synthase 4	No
cassava4.1_026813m|PACid:17992989	cassava4.1_026813m	PLC-like phosphodiesterases superfamily protein	No

cassava4.1_026824m|PACid:17975323	cassava4.1_026824m	prephenate dehydrogenase family protein	No
cassava4.1_026829m|PACid:17980088	cassava4.1_026829m	cycloartenol synthase 1	No
cassava4.1_026844m|PACid:17993864	cassava4.1_026844m	NAD(P)-binding Rossmann-fold superfamily protein	No
cassava4.1_026847m|PACid:17979021	cassava4.1_026847m	F-box family protein	No
cassava4.1_026874m|PACid:17991082	cassava4.1_026874m	Galactose oxidase/kelch repeat superfamily protein	No
cassava4.1_026921m|PACid:17976805	cassava4.1_026921m	Inositol monophosphatase family protein	No
cassava4.1_026933m|PACid:17986542	cassava4.1_026933m	Exostosin family protein	No
cassava4.1_026937m|PACid:17975022	cassava4.1_026937m	homeobox protein 21	No
cassava4.1_026940m|PACid:17979846	cassava4.1_026940m	nucleotide binding;protein binding	No
cassava4.1_026942m|PACid:17968390	cassava4.1_026942m	AGC (cAMP-dependent, cGMP-dependent and protein kinase C) kinase family protein	No
cassava4.1_026945m|PACid:17980413	cassava4.1_026945m	Thioredoxin superfamily protein	No
cassava4.1_026950m|PACid:17968398	cassava4.1_026950m	basic helix-loop-helix (bHLH) DNA-binding superfamily protein	No
cassava4.1_026953m|PACid:17974238	cassava4.1_026953m	Transducin/WD40 repeat-like superfamily protein	No
cassava4.1_026959m|PACid:17985323	cassava4.1_026959m	tonoplast intrinsic protein 5;1	No
cassava4.1_026961m|PACid:17968990	cassava4.1_026961m	allene oxide cyclase 3	No
cassava4.1_026968m|PACid:17982191	cassava4.1_026968m	Protein kinase superfamily protein	G
cassava4.1_026988m|PACid:17974050	cassava4.1_026988m	S-adenosyl-L-methionine-dependent methyltransferases superfamily protein	No
cassava4.1_027000m|PACid:17972099	cassava4.1_027000m	Alkaline phytoceramidase (aPHC)	No
cassava4.1_027003m|PACid:17982025	cassava4.1_027003m	UDP-Glycosyltransferase superfamily protein	No
cassava4.1_027031m|PACid:17989918	cassava4.1_027031m	Homeodomain-like superfamily protein	No
cassava4.1_027039m|PACid:17993642	cassava4.1_027039m	glucosyl transferase family 8	No
cassava4.1_027051m|PACid:17968057	cassava4.1_027051m	metacaspase 9	No
cassava4.1_027056m|PACid:17980970	cassava4.1_027056m	hydrolase family protein / HAD-superfamily protein	No
cassava4.1_027058m|PACid:17981586	cassava4.1_027058m	Thioredoxin superfamily protein	No
cassava4.1_027065m|PACid:17984876	cassava4.1_027065m	toprim domain-containing protein	No
cassava4.1_027080m|PACid:17962637	cassava4.1_027080m	binding to TOMV RNA 1L (long form)	GP
cassava4.1_027085m|PACid:17976919	cassava4.1_027085m		No
cassava4.1_027086m|PACid:17978416	cassava4.1_027086m	Plant invertase/pectin methylesterase inhibitor superfamily	No
cassava4.1_027088m|PACid:17988086	cassava4.1_027088m	ASYMMETRIC LEAVES 2-like 1	No
cassava4.1_027092m|PACid:17960574	cassava4.1_027092m	Duplicated homeodomain-like superfamily protein	No
cassava4.1_027095m|PACid:17990021	cassava4.1_027095m	Metal-dependent protein hydrolase	No
cassava4.1_027096m|PACid:17993558	cassava4.1_027096m	triglyceride lipases;triglyceride lipases	No
cassava4.1_027105m|PACid:17961883	cassava4.1_027105m	Major facilitator superfamily protein	No
cassava4.1_027108m|PACid:17993461	cassava4.1_027108m	peptide transporter 3	No
cassava4.1_027110m|PACid:17963250	cassava4.1_027110m	GATA type zinc finger transcription factor family protein	No
cassava4.1_027112m|PACid:17987981	cassava4.1_027112m	Tetratricopeptide repeat (TPR)-like superfamily protein	No
cassava4.1_027127m|PACid:17964261	cassava4.1_027127m	gamma-glutamyl transpeptidase 4	No
cassava4.1_027134m|PACid:17962975	cassava4.1_027134m	2-oxoglutarate (2OG) and Fe(II)-dependent oxygenase superfamily protein	No
cassava4.1_027150m|PACid:17976818	cassava4.1_027150m	decapping 1	No

cassava4.1_027155m|PACid:17992977	cassava4.1_027155m	Transducin/WD40 repeat-like superfamily protein	G
cassava4.1_027170m|PACid:17960580	cassava4.1_027170m	ubiquitin fusion degradation 1	P
cassava4.1_027198m|PACid:17966331	cassava4.1_027198m	phosphoserine aminotransferase	P
cassava4.1_027209m|PACid:17974669	cassava4.1_027209m	FUMARASE 2	P
cassava4.1_027220m|PACid:17969028	cassava4.1_027220m	myb domain protein 28	No
cassava4.1_027245m|PACid:17962744	cassava4.1_027245m	Presenilin-2	No
cassava4.1_027252m|PACid:17985220	cassava4.1_027252m	Preprotein translocase Sec, Sec61-beta subunit protein	No
cassava4.1_027261m|PACid:17977673	cassava4.1_027261m	RmlC-like cupins superfamily protein	No
cassava4.1_027276m|PACid:17975264	cassava4.1_027276m	NOD26-like intrinsic protein 6;1	No
cassava4.1_027288m|PACid:17959884	cassava4.1_027288m	glutamine dumper 3	No
cassava4.1_027299m|PACid:17971905	cassava4.1_027299m	Tetratricopeptide repeat (TPR)-like superfamily protein	No
cassava4.1_027303m|PACid:17984692	cassava4.1_027303m	Uncharacterised protein family (UPF0497)	No
cassava4.1_027307m|PACid:17987329	cassava4.1_027307m	Actin-like ATPase superfamily protein	No
cassava4.1_027321m|PACid:17992179	cassava4.1_027321m	ATP binding;protein kinases;protein serine/threonine kinases	No
cassava4.1_027328m|PACid:17985566	cassava4.1_027328m	Arabidopsis thaliana protein of unknown function (DUF821)	No
cassava4.1_027337m|PACid:17966072	cassava4.1_027337m		No
cassava4.1_027353m|PACid:17990599	cassava4.1_027353m	Calcium-dependent lipid-binding (CaLB domain) family protein	No
cassava4.1_027359m|PACid:17991191	cassava4.1_027359m	uridine-ribohydrolase 1	GP
cassava4.1_027374m|PACid:17970016	cassava4.1_027374m	Pentatricopeptide repeat (PPR) superfamily protein	No
cassava4.1_027380m|PACid:17976542	cassava4.1_027380m	MATE efflux family protein	No
cassava4.1_027395m|PACid:17973281	cassava4.1_027395m	GINS complex protein	No
cassava4.1_027401m|PACid:17979356	cassava4.1_027401m	Predicted AT-hook DNA-binding family protein	No
cassava4.1_027411m|PACid:17988394	cassava4.1_027411m	Cystathionine beta-synthase (CBS) protein	No
cassava4.1_027417m|PACid:17990971	cassava4.1_027417m	AAA-type ATPase family protein	P
cassava4.1_027429m|PACid:17974409	cassava4.1_027429m	Tetratricopeptide repeat (TPR)-like superfamily protein	G
cassava4.1_027469m|PACid:17982720	cassava4.1_027469m	cationic amino acid transporter 5	No
cassava4.1_027483m|PACid:17993909	cassava4.1_027483m	Mob1/phocein family protein	No
cassava4.1_027499m|PACid:17972101	cassava4.1_027499m	Protein of unknown function (DUF778)	No
cassava4.1_027510m|PACid:17967472	cassava4.1_027510m	Spo11/DNA topoisomerase VI, subunit A protein	No
cassava4.1_027519m|PACid:17981339	cassava4.1_027519m	Peroxidase superfamily protein	No
cassava4.1_027527m|PACid:17983777	cassava4.1_027527m	DNA helicase (RECQl4A)	No
cassava4.1_027529m|PACid:17989909	cassava4.1_027529m	Protein of unknown function (DUF1264)	No
cassava4.1_027548m|PACid:17967244	cassava4.1_027548m	Insulinase (Peptidase family M16) family protein	No
cassava4.1_027560m|PACid:17972995	cassava4.1_027560m	Cox19-like CHCH family protein	No
cassava4.1_027567m|PACid:17964818	cassava4.1_027567m	2-oxoglutarate (2OG) and Fe(II)-dependent oxygenase superfamily protein	No
cassava4.1_027603m|PACid:17967337	cassava4.1_027603m	phosphate transporter 3;1	P
cassava4.1_027606m|PACid:17989329	cassava4.1_027606m	cysteine synthase D1	No
cassava4.1_027612m|PACid:17966982	cassava4.1_027612m	myb domain protein 93	No
cassava4.1_027613m|PACid:17983909	cassava4.1_027613m	Zinc finger (CCCH-type/C3HC4-type RING finger) family protein	No

cassava4.1_027626m|PACid:17963010	cassava4.1_027626m	Transducin/WD40 repeat-like superfamily protein	No
cassava4.1_027640m|PACid:17975107	cassava4.1_027640m	GDSL-like Lipase/Acylhydrolase superfamily protein	No
cassava4.1_027647m|PACid:17967548	cassava4.1_027647m	cellulose synthase-like D1	No
cassava4.1_027659m|PACid:17979155	cassava4.1_027659m	Protein kinase superfamily protein	No
cassava4.1_027663m|PACid:17989113	cassava4.1_027663m	H(+)-ATPase 7	No
cassava4.1_027686m|PACid:17988188	cassava4.1_027686m	vascular related NAC-domain protein 7	No
cassava4.1_027687m|PACid:17990174	cassava4.1_027687m	SAUR-like auxin-responsive protein family	No
cassava4.1_027690m|PACid:17961691	cassava4.1_027690m	Calcium-dependent phospholipid-binding Copine family protein	No
cassava4.1_027692m|PACid:17962415	cassava4.1_027692m	indole-3-acetic acid inducible 33	No
cassava4.1_027713m|PACid:17986792	cassava4.1_027713m	beta glucosidase 17	No
cassava4.1_027725m|PACid:17983553	cassava4.1_027725m	F-box family protein	No
cassava4.1_027734m|PACid:17968164	cassava4.1_027734m	Disease resistance-responsive (dirigent-like protein) family protein	No
cassava4.1_027746m|PACid:17967161	cassava4.1_027746m	hydroxysteroid dehydrogenase 5	No
cassava4.1_027747m|PACid:17980628	cassava4.1_027747m	Auxin-responsive family protein	G
cassava4.1_027753m|PACid:17981614	cassava4.1_027753m	cytidine deaminase 1	GP
cassava4.1_027770m|PACid:17960090	cassava4.1_027770m	Vacuolar iron transporter (VIT) family protein	No
cassava4.1_027820m|PACid:17970636	cassava4.1_027820m	alpha carbonic anhydrase 4	No
cassava4.1_027845m|PACid:17989940	cassava4.1_027845m	chitinase A	No
cassava4.1_027878m|PACid:17964619	cassava4.1_027878m	expansin A17	No
cassava4.1_027894m|PACid:17990888	cassava4.1_027894m	Pectin lyase-like superfamily protein	No
cassava4.1_027898m|PACid:17976844	cassava4.1_027898m		GP
cassava4.1_027902m|PACid:17973874	cassava4.1_027902m		No
cassava4.1_027914m|PACid:17982782	cassava4.1_027914m	phytosulfokin receptor 1	No
cassava4.1_027916m|PACid:17970326	cassava4.1_027916m	thylakoid lumen 15.0 kDa protein	G
cassava4.1_027928m|PACid:17987582	cassava4.1_027928m	Calcium-dependent lipid-binding (CaLB domain) family protein	G
cassava4.1_027931m|PACid:17994115	cassava4.1_027931m	Protein of unknown function DUF106, transmembrane	No
cassava4.1_027944m|PACid:17971253	cassava4.1_027944m	transmembrane protein G1P-related 1	No
cassava4.1_027945m|PACid:17969642	cassava4.1_027945m	Pentatricopeptide repeat (PPR) superfamily protein	No
cassava4.1_027951m|PACid:17966498	cassava4.1_027951m	golgi nucleotide sugar transporter 2	No
cassava4.1_027960m|PACid:17974465	cassava4.1_027960m	3-oxo-5-alpha-steroid 4-dehydrogenase family protein	No
cassava4.1_027967m|PACid:17985734	cassava4.1_027967m	Calcium-dependent lipid-binding (CaLB domain) family protein	No
cassava4.1_027977m|PACid:17987332	cassava4.1_027977m	alpha/beta-Hydrolases superfamily protein	No
cassava4.1_027983m|PACid:17984516	cassava4.1_027983m	TRICHOME BIREFRINGENCE-LIKE 7	No
cassava4.1_028003m|PACid:17974208	cassava4.1_028003m	dolichol-phosphate mannosyltransferase-related	No
cassava4.1_028010m|PACid:17971165	cassava4.1_028010m	uridine kinase-like 5	No
cassava4.1_028027m|PACid:17991557	cassava4.1_028027m	Calcium-binding tetratricopeptide family protein	P
cassava4.1_028029m|PACid:17968948	cassava4.1_028029m	Pentatricopeptide repeat (PPR) superfamily protein	No
cassava4.1_028041m|PACid:17988530	cassava4.1_028041m	Haloacid dehalogenase-like hydrolase (HAD) superfamily protein	No
cassava4.1_028043m|PACid:17991778	cassava4.1_028043m	protein tyrosine phosphatases;protein tyrosine phosphatases	No

cassava4.1_028047m|PACid:17984359	cassava4.1_028047m	Protein of unknown function, DUF617	No
cassava4.1_028056m|PACid:17962722	cassava4.1_028056m	Tetratricopeptide repeat (TPR)-like superfamily protein	No
cassava4.1_028062m|PACid:17980110	cassava4.1_028062m	LisH dimerisation motif;WD40/YVTN repeat-like-containing domain	GP
cassava4.1_028075m|PACid:17961430	cassava4.1_028075m	TAPETUM 1	No
cassava4.1_028078m|PACid:17992628	cassava4.1_028078m	GDSL-like Lipase/Acylhydrolase superfamily protein	No
cassava4.1_028090m|PACid:17960502	cassava4.1_028090m	GDSL-like Lipase/Acylhydrolase superfamily protein	No
cassava4.1_028108m|PACid:17989131	cassava4.1_028108m	Protein of unknown function, DUF617	No
cassava4.1_028118m|PACid:17990656	cassava4.1_028118m	MITOCHONDRIAL FERREDOXIN 2	No
cassava4.1_028121m|PACid:17977808	cassava4.1_028121m	alpha/beta-Hydrolases superfamily protein	No
cassava4.1_028154m|PACid:17980999	cassava4.1_028154m	Chalcone and stilbene synthase family protein	No
cassava4.1_028155m|PACid:17986059	cassava4.1_028155m	GRAS family transcription factor	No
cassava4.1_028161m|PACid:17978427	cassava4.1_028161m	FKBP-like peptidyl-prolyl cis-trans isomerase family protein	No
cassava4.1_028175m|PACid:17960307	cassava4.1_028175m	copper transporter 5	No
cassava4.1_028181m|PACid:17976467	cassava4.1_028181m	Eukaryotic aspartyl protease family protein	No
cassava4.1_028184m|PACid:17968720	cassava4.1_028184m	Acyl-CoA N-acyltransferases (NAT) superfamily protein	No
cassava4.1_028233m|PACid:17966202	cassava4.1_028233m	RNA-binding (RRM/RBD/RNP motifs) family protein	G
cassava4.1_028235m|PACid:17979011	cassava4.1_028235m	Protein of unknown function (DUF177)	G
cassava4.1_028238m|PACid:17992155	cassava4.1_028238m	Pentatricopeptide repeat (PPR-like) superfamily protein	No
cassava4.1_028243m|PACid:17971872	cassava4.1_028243m	RNA-binding S4 domain-containing protein	No
cassava4.1_028246m|PACid:17961216	cassava4.1_028246m	HAD-superfamily hydrolase, subfamily IG, 5\'-nucleotidase	No
cassava4.1_028254m|PACid:17991079	cassava4.1_028254m	NAC domain containing protein 50	G
cassava4.1_028257m|PACid:17974239	cassava4.1_028257m	Pentatricopeptide repeat (PPR) superfamily protein	G
cassava4.1_028261m|PACid:17976128	cassava4.1_028261m	5\'-nucleotidases;magnesium ion binding	G
cassava4.1_028263m|PACid:17963833	cassava4.1_028263m	C2H2 zinc-finger protein SERRATE (SE)	GP
cassava4.1_028282m|PACid:17978462	cassava4.1_028282m	Phosphoglycerate mutase-like family protein	No
cassava4.1_028287m|PACid:17974147	cassava4.1_028287m	Xanthine/uracil permease family protein	No
cassava4.1_028289m|PACid:17968261	cassava4.1_028289m	myb domain protein 63	No
cassava4.1_028292m|PACid:17971823	cassava4.1_028292m	tRNA modification GTPase, putative	G
cassava4.1_028315m|PACid:17962979	cassava4.1_028315m	Tetratricopeptide repeat (TPR)-like superfamily protein	No
cassava4.1_028340m|PACid:17978565	cassava4.1_028340m	ACC synthase 1	No
cassava4.1_028377m|PACid:17966442	cassava4.1_028377m	Cytochrome P450 superfamily protein	No
cassava4.1_028382m|PACid:17992999	cassava4.1_028382m	O-Glycosyl hydrolases family 17 protein	No
cassava4.1_028396m|PACid:17962224	cassava4.1_028396m	ADP glucose pyrophosphorylase large subunit 1	No
cassava4.1_028404m|PACid:17968927	cassava4.1_028404m	pentatricopeptide (PPR) repeat-containing protein	No
cassava4.1_028409m|PACid:17966835	cassava4.1_028409m	Saccharopine dehydrogenase	No
cassava4.1_028433m|PACid:17978403	cassava4.1_028433m	beta glucosidase 40	No
cassava4.1_028444m|PACid:17975754	cassava4.1_028444m	receptor-like protein kinase 4	G
cassava4.1_028463m|PACid:17986152	cassava4.1_028463m	Tetratricopeptide repeat (TPR)-like superfamily protein	No
cassava4.1_028477m|PACid:17963167	cassava4.1_028477m	Mitochondrial substrate carrier family protein	No

cassava4.1_028496m|PACid:17980954	cassava4.1_028496m	Histone superfamily protein	P
cassava4.1_028517m|PACid:17971308	cassava4.1_028517m	DNA-binding HORMA family protein	G
cassava4.1_028523m|PACid:17964507	cassava4.1_028523m	P-loop containing nucleoside triphosphate hydrolases superfamily protein	No
cassava4.1_028568m|PACid:17976749	cassava4.1_028568m	Pentatricopeptide repeat (PPR) superfamily protein	No
cassava4.1_028573m|PACid:17971169	cassava4.1_028573m	myb domain protein 118	No
cassava4.1_028575m|PACid:17966813	cassava4.1_028575m	lipoyltransferase 2	No
cassava4.1_028581m|PACid:17973308	cassava4.1_028581m	nuclear RNA polymerase C2	No
cassava4.1_028586m|PACid:17978049	cassava4.1_028586m	Tetratricopeptide repeat (TPR)-like superfamily protein	No
cassava4.1_028588m|PACid:17963271	cassava4.1_028588m	actin depolymerizing factor 11	No
cassava4.1_028608m|PACid:17975076	cassava4.1_028608m	Formyl transferase	G
cassava4.1_028617m|PACid:17978250	cassava4.1_028617m	Tetratricopeptide repeat (TPR)-like superfamily protein	No
cassava4.1_028648m|PACid:17961757	cassava4.1_028648m	Serine protease inhibitor, potato inhibitor I-type family protein	No
cassava4.1_028650m|PACid:17987867	cassava4.1_028650m	cellulose synthase A9	P
cassava4.1_028664m|PACid:17964950	cassava4.1_028664m	Major facilitator superfamily protein	G
cassava4.1_028675m|PACid:17962469	cassava4.1_028675m	Seven transmembrane MLO family protein	No
cassava4.1_028680m|PACid:17966456	cassava4.1_028680m	JOSEPHIN-like protein	No
cassava4.1_028682m|PACid:17964172	cassava4.1_028682m	RmlC-like cupins superfamily protein	No
cassava4.1_028698m|PACid:17965571	cassava4.1_028698m	Pentatricopeptide repeat (PPR) superfamily protein	No
cassava4.1_028711m|PACid:17991499	cassava4.1_028711m	root hair specific 14	No
cassava4.1_028723m|PACid:17964084	cassava4.1_028723m	Pentatricopeptide repeat (PPR) superfamily protein	No
cassava4.1_028731m|PACid:17985015	cassava4.1_028731m	Protein kinase superfamily protein	No
cassava4.1_028733m|PACid:17981529	cassava4.1_028733m	Peptide chain release factor 1	GP
cassava4.1_028758m|PACid:17981277	cassava4.1_028758m	HXXXD-type acyl-transferase family protein	No
cassava4.1_028772m|PACid:17993870	cassava4.1_028772m	Polynucleotidyl transferase, ribonuclease H fold protein with HRDC domain	No
cassava4.1_028788m|PACid:17989084	cassava4.1_028788m	tonoplast intrinsic protein 1;3	No
cassava4.1_028791m|PACid:17967928	cassava4.1_028791m	serine carboxypeptidase-like 40	No
cassava4.1_028802m|PACid:17987953	cassava4.1_028802m	Class I peptide chain release factor	No
cassava4.1_028803m|PACid:17985722	cassava4.1_028803m	ubiquitin-conjugating enzyme 37	No
cassava4.1_028806m|PACid:17960299	cassava4.1_028806m	SAUR-like auxin-responsive protein family	No
cassava4.1_028827m|PACid:17963445	cassava4.1_028827m	glutamate receptor 3.4	No
cassava4.1_028848m|PACid:17989389	cassava4.1_028848m	ankyrin repeat family protein	GP
cassava4.1_028853m|PACid:17973329	cassava4.1_028853m	Plant protein of unknown function (DUF639)	No
cassava4.1_028857m|PACid:17984208	cassava4.1_028857m	Protein kinase superfamily protein	No
cassava4.1_028860m|PACid:17979728	cassava4.1_028860m	Arabidopsis thaliana protein of unknown function (DUF821)	No
cassava4.1_028861m|PACid:17967531	cassava4.1_028861m	oligopeptide transporter 1	No
cassava4.1_028863m|PACid:17989774	cassava4.1_028863m	phosphatidylinositolglycan synthase family protein	No
cassava4.1_028887m|PACid:17993117	cassava4.1_028887m	cation/hydrogen exchanger 15	No
cassava4.1_028896m|PACid:17974878	cassava4.1_028896m	Pentatricopeptide repeat (PPR-like) superfamily protein	No
cassava4.1_028915m|PACid:17960151	cassava4.1_028915m	AZA-guanine resistant1	No

cassava4.1_028927m|PACid:17961177	cassava4.1_028927m	xylulose kinase-1	No
cassava4.1_028928m|PACid:17982497	cassava4.1_028928m	Leucine-rich repeat receptor-like protein kinase family protein	No
cassava4.1_028947m|PACid:17964736	cassava4.1_028947m	Pectin lyase-like superfamily protein	No
cassava4.1_028958m|PACid:17977188	cassava4.1_028958m	MATE efflux family protein	No
cassava4.1_028963m|PACid:17986038	cassava4.1_028963m	Malectin/receptor-like protein kinase family protein	No
cassava4.1_028974m|PACid:17974610	cassava4.1_028974m	Tetratricopeptide repeat (TPR)-like superfamily protein	No
cassava4.1_028994m|PACid:17961522	cassava4.1_028994m	WRKY DNA-binding protein 75	No
cassava4.1_029004m|PACid:17965587	cassava4.1_029004m	NAD(P)-binding Rossmann-fold superfamily protein	No
cassava4.1_029009m|PACid:17973580	cassava4.1_029009m	Tetratricopeptide repeat (TPR)-like superfamily protein	No
cassava4.1_029014m|PACid:17975038	cassava4.1_029014m	fimbrin 1	No
cassava4.1_029022m|PACid:17963074	cassava4.1_029022m	embryo defective 3012	No
cassava4.1_029023m|PACid:17971418	cassava4.1_029023m	Leucine-rich repeat (LRR) family protein	P
cassava4.1_029047m|PACid:17966347	cassava4.1_029047m	sugar transporter 6	No
cassava4.1_029048m|PACid:17961401	cassava4.1_029048m	Homeodomain-like superfamily protein	No
cassava4.1_029050m|PACid:17986228	cassava4.1_029050m	Protein kinase family protein	No
cassava4.1_029051m|PACid:17964388	cassava4.1_029051m	Tetratricopeptide repeat (TPR)-like superfamily protein	No
cassava4.1_029053m|PACid:17961112	cassava4.1_029053m	RmlC-like cupins superfamily protein	GP
cassava4.1_029071m|PACid:17988962	cassava4.1_029071m	nuclear factor Y, subunit B5	No
cassava4.1_029076m|PACid:17980046	cassava4.1_029076m	UDP-glucosyl transferase 73B3	No
cassava4.1_029078m|PACid:17968713	cassava4.1_029078m	Auxin efflux carrier family protein	No
cassava4.1_029088m|PACid:17981390	cassava4.1_029088m	Subtilase family protein	No
cassava4.1_029090m|PACid:17971023	cassava4.1_029090m	HMG (high mobility group) box protein	No
cassava4.1_029117m|PACid:17977376	cassava4.1_029117m	DZC (Disease resistance/zinc finger/chromosome condensation-like region) domain containing protein	No
cassava4.1_029124m|PACid:17972806	cassava4.1_029124m	Homeodomain-like superfamily protein	G
cassava4.1_029133m|PACid:17967734	cassava4.1_029133m	Tetratricopeptide repeat (TPR)-like superfamily protein	No
cassava4.1_029134m|PACid:17984871	cassava4.1_029134m	Protein phosphatase 2C family protein	No
cassava4.1_029140m|PACid:17961950	cassava4.1_029140m	Protein kinase superfamily protein	No
cassava4.1_029148m|PACid:17982095	cassava4.1_029148m	ATP-binding cassette A1	P
cassava4.1_029149m|PACid:17986213	cassava4.1_029149m	Leucine-rich repeat (LRR) family protein	No
cassava4.1_029153m|PACid:17972678	cassava4.1_029153m	Trimeric LpxA-like enzymes superfamily protein	No
cassava4.1_029182m|PACid:17979534	cassava4.1_029182m	Pathogenesis-related thaumatin superfamily protein	No
cassava4.1_029188m|PACid:17965293	cassava4.1_029188m	DNA repair (Rad51) family protein	No
cassava4.1_029194m|PACid:17973719	cassava4.1_029194m	Cleavage and polyadenylation specificity factor (CPSF) A subunit protein	P
cassava4.1_029197m|PACid:17965488	cassava4.1_029197m	phosphorylcholine cytidylyltransferase	GP
cassava4.1_029232m|PACid:17979277	cassava4.1_029232m	ferulic acid 5-hydroxylase 1	No
cassava4.1_029236m|PACid:17993402	cassava4.1_029236m	ammonium transporter 1;1	No
cassava4.1_029240m|PACid:17992887	cassava4.1_029240m	Oligosaccharyltransferase complex/magnesium transporter family protein	GP
cassava4.1_029251m|PACid:17971683	cassava4.1_029251m	Flavin-binding monooxygenase family protein	No
cassava4.1_029256m|PACid:17979148	cassava4.1_029256m	Maf-like protein	No

cassava4.1_029262m|PACid:17962862	cassava4.1_029262m		No
cassava4.1_029266m|PACid:17971126	cassava4.1_029266m	Protein of unknown function (DUF962)	No
cassava4.1_029277m|PACid:17988821	cassava4.1_029277m	alpha/beta-Hydrolases superfamily protein	No
cassava4.1_029282m|PACid:17969665	cassava4.1_029282m	Tetrapyrrole (Corrin/Porphyrin) Methylases	No
cassava4.1_029314m|PACid:17985056	cassava4.1_029314m	basic pentacysteine 6	G
cassava4.1_029321m|PACid:17971202	cassava4.1_029321m	bacterial transferase hexapeptide repeat-containing protein	No
cassava4.1_029322m|PACid:17967065	cassava4.1_029322m	prefoldin 5	GP
cassava4.1_029328m|PACid:17984264	cassava4.1_029328m	cooperatively regulated by ethylene and jasmonate 1	No
cassava4.1_029344m|PACid:17986839	cassava4.1_029344m	ubiquitin-specific protease 8	No
cassava4.1_029365m|PACid:17990132	cassava4.1_029365m	Glutathione S-transferase family protein	GP
cassava4.1_029369m|PACid:17967449	cassava4.1_029369m	malate synthase	No
cassava4.1_029399m|PACid:17981317	cassava4.1_029399m	Galactose oxidase/kelch repeat superfamily protein	No
cassava4.1_029406m|PACid:17969025	cassava4.1_029406m	Pentatricopeptide repeat (PPR) superfamily protein	No
cassava4.1_029410m|PACid:17980623	cassava4.1_029410m	Tetratricopeptide repeat (TPR)-like superfamily protein	No
cassava4.1_029423m|PACid:17969389	cassava4.1_029423m	Tetratricopeptide repeat (TPR)-like superfamily protein	No
cassava4.1_029426m|PACid:17992897	cassava4.1_029426m	cytochrome P450, family 77, subfamily B, polypeptide 1	No
cassava4.1_029429m|PACid:17983292	cassava4.1_029429m	cytochrome B5 isoform A	G
cassava4.1_029430m|PACid:17962591	cassava4.1_029430m	Protein of unknown function (DUF679)	No
cassava4.1_029431m|PACid:17984735	cassava4.1_029431m	NagB/RpiA/CoA transferase-like superfamily protein	No
cassava4.1_029432m|PACid:17962787	cassava4.1_029432m	ARM repeat superfamily protein	No
cassava4.1_029434m|PACid:17984368	cassava4.1_029434m	SKP1/ASK-interacting protein 5	No
cassava4.1_029443m|PACid:17988156	cassava4.1_029443m	DNAse I-like superfamily protein	G
cassava4.1_029446m|PACid:17986436	cassava4.1_029446m	cytochrome P450, family 86, subfamily C, polypeptide 1	No
cassava4.1_029457m|PACid:17970319	cassava4.1_029457m	Tetratricopeptide repeat (TPR)-like superfamily protein	No
cassava4.1_029468m|PACid:17978865	cassava4.1_029468m	Tryptophan RNA-binding attenuator protein-like	No
cassava4.1_029470m|PACid:17970893	cassava4.1_029470m	NAD(P)-binding Rossmann-fold superfamily protein	No
cassava4.1_029490m|PACid:17961718	cassava4.1_029490m	ATP binding microtubule motor family protein	No
cassava4.1_029509m|PACid:17993966	cassava4.1_029509m	crinkly4	No
cassava4.1_029519m|PACid:17977833	cassava4.1_029519m	P-loop containing nucleoside triphosphate hydrolases superfamily protein	No
cassava4.1_029521m|PACid:17985610	cassava4.1_029521m	HEAT SHOCK PROTEIN 81.4	GP
cassava4.1_029528m|PACid:17985688	cassava4.1_029528m	Eukaryotic aspartyl protease family protein	GP
cassava4.1_029531m|PACid:17964253	cassava4.1_029531m	Mnd1 family protein	No
cassava4.1_029533m|PACid:17977767	cassava4.1_029533m	chloroplast-localized ISCA-like protein	No
cassava4.1_029536m|PACid:17990438	cassava4.1_029536m	cyclic nucleotide gated channel 8	No
cassava4.1_029547m|PACid:17983711	cassava4.1_029547m	mitochondrial F0-ATPase subunit 9	No
cassava4.1_029565m|PACid:17973468	cassava4.1_029565m	cytochrome P450, family 94, subfamily C, polypeptide 1	No
cassava4.1_029583m|PACid:17986465	cassava4.1_029583m		No
cassava4.1_029591m|PACid:17968614	cassava4.1_029591m	P-loop containing nucleoside triphosphate hydrolases superfamily protein	No
cassava4.1_029597m|PACid:17960747	cassava4.1_029597m	Glucose-methanol-choline (GMC) oxidoreductase family protein	No

cassava4.1_029600m|PACid:17979667	cassava4.1_029600m	phospholipase D P1	No
cassava4.1_029613m|PACid:17982634	cassava4.1_029613m	Pectin lyase-like superfamily protein	No
cassava4.1_029618m|PACid:17967216	cassava4.1_029618m	arabinogalactan protein 14	G
cassava4.1_029624m|PACid:17979572	cassava4.1_029624m	RNA-binding (RRM/RBD/RNP motifs) family protein	G
cassava4.1_029627m|PACid:17967080	cassava4.1_029627m	Transmembrane amino acid transporter family protein	No
cassava4.1_029630m|PACid:17974973	cassava4.1_029630m	chorismate mutase 3	No
cassava4.1_029660m|PACid:17980888	cassava4.1_029660m	Late embryogenesis abundant (LEA) protein-related	No
cassava4.1_029664m|PACid:17976782	cassava4.1_029664m	tyrosyl-DNA phosphodiesterase-related	No
cassava4.1_029666m|PACid:17966961	cassava4.1_029666m	Plant U-Box 15	No
cassava4.1_029678m|PACid:17988972	cassava4.1_029678m	Microsomal signal peptidase 25 kDa subunit (SPC25)	No
cassava4.1_029680m|PACid:17969599	cassava4.1_029680m	plant intracellular ras group-related LRR 9	No
cassava4.1_029718m|PACid:17970743	cassava4.1_029718m	AMP-dependent synthetase and ligase family protein	P
cassava4.1_029739m|PACid:17992507	cassava4.1_029739m	DnaJ/Hsp40 cysteine-rich domain superfamily protein	No
cassava4.1_029766m|PACid:17980348	cassava4.1_029766m	Yippee family putative zinc-binding protein	No
cassava4.1_029770m|PACid:17960599	cassava4.1_029770m	exocyst subunit exo70 family protein B1	No
cassava4.1_029790m|PACid:17963523	cassava4.1_029790m	SH2 domain protein B	No
cassava4.1_029801m|PACid:17964621	cassava4.1_029801m	Peptide chain release factor 1	No
cassava4.1_029811m|PACid:17959812	cassava4.1_029811m	CBL-interacting protein kinase 25	No
cassava4.1_029812m|PACid:17969783	cassava4.1_029812m	origin recognition complex subunit 4	No
cassava4.1_029817m|PACid:17970264	cassava4.1_029817m	ABC transporter family protein	G
cassava4.1_029823m|PACid:17994103	cassava4.1_029823m	calmodulin-domain protein kinase cdpk isoform 2	No
cassava4.1_029834m|PACid:17959986	cassava4.1_029834m	2-oxoglutarate (2OG) and Fe(II)-dependent oxygenase superfamily protein	No
cassava4.1_029862m|PACid:17984690	cassava4.1_029862m	Peroxidase superfamily protein	No
cassava4.1_029868m|PACid:17965402	cassava4.1_029868m	SHK1 binding protein 1	GP
cassava4.1_029876m|PACid:17988385	cassava4.1_029876m	basic helix-loop-helix (bHLH) DNA-binding family protein	No
cassava4.1_029883m|PACid:17993157	cassava4.1_029883m	GDSL-like Lipase/Acylhydrolase superfamily protein	No
cassava4.1_029907m|PACid:17971365	cassava4.1_029907m	SLOW GROWTH 1	No
cassava4.1_029922m|PACid:17991226	cassava4.1_029922m	allantoate amidohydrolase	G
cassava4.1_029923m|PACid:17983644	cassava4.1_029923m	MUTS homolog 7	No
cassava4.1_029935m|PACid:17983304	cassava4.1_029935m	K-box region and MADS-box transcription factor family protein	No
cassava4.1_029952m|PACid:17963099	cassava4.1_029952m	Cytochrome c oxidase subunit Vc family protein	GP
cassava4.1_029979m|PACid:17977487	cassava4.1_029979m	minichromosome maintenance 10	No
cassava4.1_029992m|PACid:17984637	cassava4.1_029992m	Tetratricopeptide repeat (TPR)-like superfamily protein	No
cassava4.1_029993m|PACid:17990060	cassava4.1_029993m	Rhamnogalacturonate lyase family protein	No
cassava4.1_029999m|PACid:17967278	cassava4.1_029999m	Phototropic-responsive NPH3 family protein	No
cassava4.1_030005m|PACid:17984842	cassava4.1_030005m	hercules receptor kinase 2	No
cassava4.1_030007m|PACid:17987041	cassava4.1_030007m	Flavin-binding monooxygenase family protein	No
cassava4.1_030009m|PACid:17990190	cassava4.1_030009m	BTB/POZ domain-containing protein	No
cassava4.1_030015m|PACid:17970269	cassava4.1_030015m	SAUR-like auxin-responsive protein family	No

cassava4.1_030019m|PACid:17979301	cassava4.1_030019m	glutamate receptor 3.6	No
cassava4.1_030020m|PACid:17988367	cassava4.1_030020m	Tetratricopeptide repeat (TPR)-like superfamily protein	No
cassava4.1_030023m|PACid:17981703	cassava4.1_030023m	DNAse I-like superfamily protein	No
cassava4.1_030043m|PACid:17989420	cassava4.1_030043m	with no lysine (K) kinase 1	G
cassava4.1_030053m|PACid:17983722	cassava4.1_030053m	cytochrome c oxidase 19-1	G
cassava4.1_030055m|PACid:17981169	cassava4.1_030055m	S-adenosyl-L-methionine-dependent methyltransferases superfamily protein	No
cassava4.1_030056m|PACid:17963622	cassava4.1_030056m	alpha/beta-Hydrolases superfamily protein	No
cassava4.1_030084m|PACid:17991428	cassava4.1_030084m		No
cassava4.1_030087m|PACid:17959723	cassava4.1_030087m	O-fucosyltransferase family protein	No
cassava4.1_030098m|PACid:17988930	cassava4.1_030098m	Tetratricopeptide repeat (TPR)-like superfamily protein	No
cassava4.1_030101m|PACid:17962674	cassava4.1_030101m	Galactose oxidase/kelch repeat superfamily protein	G
cassava4.1_030113m|PACid:17969723	cassava4.1_030113m	Calcium-dependent lipid-binding (CaLB domain) family protein	No
cassava4.1_030116m|PACid:17960528	cassava4.1_030116m	Peroxidase superfamily protein	No
cassava4.1_030120m|PACid:17992243	cassava4.1_030120m	Amino acid permease family protein	No
cassava4.1_030148m|PACid:17986297	cassava4.1_030148m	Pentatricopeptide repeat (PPR) superfamily protein	No
cassava4.1_030160m|PACid:17964355	cassava4.1_030160m	peroxidase 2	No
cassava4.1_030165m|PACid:17985678	cassava4.1_030165m	Exostosin family protein	No
cassava4.1_030169m|PACid:17960780	cassava4.1_030169m	RNA binding Plectin/S10 domain-containing protein	P
cassava4.1_030171m|PACid:17959733	cassava4.1_030171m	TIP41-like family protein	GP
cassava4.1_030174m|PACid:17987700	cassava4.1_030174m	DGCR14-related	No
cassava4.1_030190m|PACid:17961970	cassava4.1_030190m	galacturonosyltransferase-like 4	No
cassava4.1_030197m|PACid:17970235	cassava4.1_030197m	basic helix-loop-helix (bHLH) DNA-binding superfamily protein	No
cassava4.1_030220m|PACid:17964273	cassava4.1_030220m	1-amino-cyclopropane-1-carboxylate synthase 7	No
cassava4.1_030228m|PACid:17987348	cassava4.1_030228m	Tetratricopeptide repeat (TPR)-like superfamily protein	No
cassava4.1_030230m|PACid:17985867	cassava4.1_030230m	ACT-like superfamily protein	No
cassava4.1_030233m|PACid:17977712	cassava4.1_030233m	Peroxidase superfamily protein	No
cassava4.1_030240m|PACid:17961104	cassava4.1_030240m	protein binding	No
cassava4.1_030256m|PACid:17993853	cassava4.1_030256m	dehydroquinate dehydratase, putative / shikimate dehydrogenase, putative	GP
cassava4.1_030298m|PACid:17992522	cassava4.1_030298m	kinesin like protein for actin based chloroplast movement 2	GP
cassava4.1_030304m|PACid:17991705	cassava4.1_030304m		GP
cassava4.1_030308m|PACid:17971924	cassava4.1_030308m	Protein of unknown function (DUF1264)	No
cassava4.1_030315m|PACid:17974715	cassava4.1_030315m	purple acid phosphatase 16	P
cassava4.1_030320m|PACid:17976171	cassava4.1_030320m	Ribosomal S17 family protein	No
cassava4.1_030323m|PACid:17964938	cassava4.1_030323m	NRAMP metal ion transporter 6	No
cassava4.1_030325m|PACid:17978432	cassava4.1_030325m	Peroxidase superfamily protein	No
cassava4.1_030342m|PACid:17967894	cassava4.1_030342m	Peroxidase superfamily protein	No
cassava4.1_030348m|PACid:17982170	cassava4.1_030348m	photosystem I subunit l	GP
cassava4.1_030356m|PACid:17978368	cassava4.1_030356m	Pentatricopeptide repeat (PPR) superfamily protein	No
cassava4.1_030366m|PACid:17986361	cassava4.1_030366m	defective in exine formation protein (DEX1)	No

cassava4.1_030408m|PACid:17964122	cassava4.1_030408m		No
cassava4.1_030411m|PACid:17975121	cassava4.1_030411m	CBS domain-containing protein / transporter associated domain-containing protein	G
cassava4.1_030434m|PACid:17971148	cassava4.1_030434m	Pectin lyase-like superfamily protein	No
cassava4.1_030463m|PACid:17963885	cassava4.1_030463m	myb domain protein 36	No
cassava4.1_030465m|PACid:17984988	cassava4.1_030465m	2-oxoglutarate (2OG) and Fe(II)-dependent oxygenase superfamily protein	No
cassava4.1_030479m|PACid:17992156	cassava4.1_030479m	NAD(P)H dehydrogenase B2	No
cassava4.1_030492m|PACid:17978301	cassava4.1_030492m	cyclic nucleotide-gated channel 18	No
cassava4.1_030494m|PACid:17984835	cassava4.1_030494m	Subtilase family protein	No
cassava4.1_030496m|PACid:17984372	cassava4.1_030496m	Phosphatidylinositol-4-phosphate 5-kinase family protein	No
cassava4.1_030505m|PACid:17978266	cassava4.1_030505m	FAD/NAD(P)-binding oxidoreductase family protein	No
cassava4.1_030510m|PACid:17984506	cassava4.1_030510m	RmlC-like cupins superfamily protein	No
cassava4.1_030511m|PACid:17985208	cassava4.1_030511m	expansin B2	No
cassava4.1_030512m|PACid:17971049	cassava4.1_030512m	crooked neck protein, putative / cell cycle protein, putative	GP
cassava4.1_030516m|PACid:17973276	cassava4.1_030516m	nuclear RNA polymerase D1B	No
cassava4.1_030535m|PACid:17964761	cassava4.1_030535m	ENTH/ANTH/VHS superfamily protein	No
cassava4.1_030536m|PACid:17982292	cassava4.1_030536m	alpha/beta-Hydrolases superfamily protein	No
cassava4.1_030539m|PACid:17991293	cassava4.1_030539m	Transcription initiation factor IIF, beta subunit	No
cassava4.1_030552m|PACid:17961537	cassava4.1_030552m	acyl-CoA synthetase 5	No
cassava4.1_030574m|PACid:17961012	cassava4.1_030574m	glucose-6-phosphate acetyltransferase 1	No
cassava4.1_030578m|PACid:17981378	cassava4.1_030578m	EXS (ERD1/XPR1/SYG1) family protein	No
cassava4.1_030598m|PACid:17973159	cassava4.1_030598m	Nucleoside diphosphate kinase family protein	No
cassava4.1_030602m|PACid:17977623	cassava4.1_030602m	light-mediated development protein 1 / deetiolated1 (DET1)	No
cassava4.1_030619m|PACid:17960664	cassava4.1_030619m	NAD(P)-binding Rossmann-fold superfamily protein	No
cassava4.1_030620m|PACid:17973731	cassava4.1_030620m	Pectin lyase-like superfamily protein	G
cassava4.1_030625m|PACid:17971422	cassava4.1_030625m	SNARE associated Golgi protein family	G
cassava4.1_030662m|PACid:17963240	cassava4.1_030662m	phloem protein 2-A13	No
cassava4.1_030664m|PACid:17962686	cassava4.1_030664m	U5 small nuclear ribonucleoprotein helicase	No
cassava4.1_030668m|PACid:17986671	cassava4.1_030668m	RAD-like 6	No
cassava4.1_030696m|PACid:17993568	cassava4.1_030696m	myb domain protein 84	No
cassava4.1_030700m|PACid:17962334	cassava4.1_030700m	JUMONJI 14	No
cassava4.1_030728m|PACid:17967854	cassava4.1_030728m	Transducin/WD40 repeat-like superfamily protein	No
cassava4.1_030749m|PACid:17965519	cassava4.1_030749m	serine carboxypeptidase-like 34	No
cassava4.1_030753m|PACid:17971692	cassava4.1_030753m	Leucine-rich repeat transmembrane protein kinase family protein	No
cassava4.1_030813m|PACid:17983361	cassava4.1_030813m	Leucine-rich repeat (LRR) family protein	No
cassava4.1_030820m|PACid:17963202	cassava4.1_030820m	Ku80 family protein	No
cassava4.1_030822m|PACid:17967343	cassava4.1_030822m	Mitochondrial substrate carrier family protein	No
cassava4.1_030827m|PACid:17980880	cassava4.1_030827m	Homeodomain-like superfamily protein	No
cassava4.1_030828m|PACid:17973517	cassava4.1_030828m	F-box/RNI-like superfamily protein	No
cassava4.1_030830m|PACid:17987957	cassava4.1_030830m	homolog of RAD54	No

cassava4.1_030832m|PACid:17985765	cassava4.1_030832m		No
cassava4.1_030833m|PACid:17965341	cassava4.1_030833m	ribonuclease 3	G
cassava4.1_030843m|PACid:17974403	cassava4.1_030843m	phosphatidyl inositol monophosphate 5 kinase 4	No
cassava4.1_030879m|PACid:17988644	cassava4.1_030879m	DNAse I-like superfamily protein	No
cassava4.1_030886m|PACid:17986349	cassava4.1_030886m	EID1-like 3	No
cassava4.1_030889m|PACid:17981220	cassava4.1_030889m	Papain family cysteine protease	No
cassava4.1_030904m|PACid:17991417	cassava4.1_030904m	UDP-sugar pyrophosphorylase	No
cassava4.1_030920m|PACid:17991051	cassava4.1_030920m	P-type ATP-ase 1	No
cassava4.1_030922m|PACid:17992381	cassava4.1_030922m	transducin family protein / WD-40 repeat family protein	No
cassava4.1_030924m|PACid:17971602	cassava4.1_030924m	Cyclin family protein	No
cassava4.1_030937m|PACid:17973950	cassava4.1_030937m	small and basic intrinsic protein 2;1	No
cassava4.1_030943m|PACid:17980527	cassava4.1_030943m	Pectin lyase-like superfamily protein	No
cassava4.1_030962m|PACid:17963847	cassava4.1_030962m	Cyclophilin-like peptidyl-prolyl cis-trans isomerase family protein	GP
cassava4.1_030964m|PACid:17975484	cassava4.1_030964m	actin-related protein C3	No
cassava4.1_030988m|PACid:17964040	cassava4.1_030988m	P-glycoprotein 9	No
cassava4.1_030998m|PACid:17981072	cassava4.1_030998m	Pectin lyase-like superfamily protein	No
cassava4.1_031000m|PACid:17974717	cassava4.1_031000m	UDP-glcnac-adolichol phosphate glcnac-1-p-transferase	No
cassava4.1_031008m|PACid:17969417	cassava4.1_031008m	DNAJ heat shock N-terminal domain-containing protein	No
cassava4.1_031010m|PACid:17968450	cassava4.1_031010m	laccase 11	No
cassava4.1_031023m|PACid:17974681	cassava4.1_031023m	homolog of yeast autophagy 18 (ATG18) B	No
cassava4.1_031026m|PACid:17981716	cassava4.1_031026m	F-box family protein	G
cassava4.1_031046m|PACid:17983878	cassava4.1_031046m	Leucine-rich repeat (LRR) family protein	No
cassava4.1_031056m|PACid:17962134	cassava4.1_031056m	Regulator of chromosome condensation (RCC1) family protein	No
cassava4.1_031073m|PACid:17973718	cassava4.1_031073m	serine carboxypeptidase-like 28	No
cassava4.1_031095m|PACid:17968798	cassava4.1_031095m	nitrate transporter 2.4	No
cassava4.1_031109m|PACid:17964569	cassava4.1_031109m	NAD(P)-linked oxidoreductase superfamily protein	No
cassava4.1_031124m|PACid:17975641	cassava4.1_031124m	Erythronate-4-phosphate dehydrogenase family protein	G
cassava4.1_031141m|PACid:17979438	cassava4.1_031141m	thioredoxin H-type 1	No
cassava4.1_031143m|PACid:17976417	cassava4.1_031143m	Pectin lyase-like superfamily protein	No
cassava4.1_031145m|PACid:17971037	cassava4.1_031145m	Peroxidase superfamily protein	No
cassava4.1_031152m|PACid:17959875	cassava4.1_031152m	Actin-binding FH2 (Formin Homology) protein	No
cassava4.1_031160m|PACid:17979465	cassava4.1_031160m		No
cassava4.1_031170m|PACid:17984293	cassava4.1_031170m	Nucleotide-diphospho-sugar transferases superfamily protein	No
cassava4.1_031173m|PACid:17979059	cassava4.1_031173m	tRNA/rRNA methyltransferase (SpoU) family protein	No
cassava4.1_031182m|PACid:17962976	cassava4.1_031182m	Lactoylglutathione lyase / glyoxalase I family protein	No
cassava4.1_031197m|PACid:17977492	cassava4.1_031197m	glycosyl hydrolase family 35 protein	No
cassava4.1_031219m|PACid:17989745	cassava4.1_031219m	Protein phosphatase 2C family protein	No
cassava4.1_031223m|PACid:17993871	cassava4.1_031223m	ARABIDOPSIS SERIN PROTEASE	GP
cassava4.1_031245m|PACid:17982645	cassava4.1_031245m	calcium-dependent lipid-binding family protein	No

cassava4.1_031246m|PACid:17981861	cassava4.1_031246m	phosphoglucose isomerase 1	P
cassava4.1_031256m|PACid:17985381	cassava4.1_031256m	FMN-linked oxidoreductases superfamily protein	G
cassava4.1_031257m|PACid:17985665	cassava4.1_031257m	eukaryotic translation initiation factor 3E	No
cassava4.1_031258m|PACid:17969750	cassava4.1_031258m		G
cassava4.1_031259m|PACid:17969788	cassava4.1_031259m	methyltransferases;nucleic acid binding	G
cassava4.1_031262m|PACid:17975441	cassava4.1_031262m	Uncharacterised protein family (UPF0497)	No
cassava4.1_031276m|PACid:17982008	cassava4.1_031276m	phytochromobilin:ferredoxin oxidoreductase, chloroplast / phytochromobilin synthase (HY2)	No
cassava4.1_031277m|PACid:17993156	cassava4.1_031277m	Pectin lyase-like superfamily protein	No
cassava4.1_031288m|PACid:17986649	cassava4.1_031288m	3-ketoacyl-CoA synthase 4	No
cassava4.1_031295m|PACid:17967353	cassava4.1_031295m	tapetum determinant 1	No
cassava4.1_031299m|PACid:17980938	cassava4.1_031299m	homolog of RAD51 D	No
cassava4.1_031309m|PACid:17977178	cassava4.1_031309m	myb domain protein 42	No
cassava4.1_031316m|PACid:17961622	cassava4.1_031316m	Translation protein SH3-like family protein	G
cassava4.1_031321m|PACid:17973845	cassava4.1_031321m	phospholipase D P1	No
cassava4.1_031323m|PACid:17964978	cassava4.1_031323m	floral meristem identity control protein LEAFY (LFY)	No
cassava4.1_031331m|PACid:17983807	cassava4.1_031331m	Protein kinase superfamily protein	No
cassava4.1_031346m|PACid:17987958	cassava4.1_031346m	Calcium-binding EF-hand family protein	No
cassava4.1_031356m|PACid:17993573	cassava4.1_031356m	1-amino-cyclopropane-1-carboxylate synthase 8	No
cassava4.1_031371m|PACid:17987624	cassava4.1_031371m	RHD SIX-LIKE 1	No
cassava4.1_031373m|PACid:17970110	cassava4.1_031373m	F-box/RNI-like superfamily protein	No
cassava4.1_031382m|PACid:17986387	cassava4.1_031382m	Protein kinase protein with tetratricopeptide repeat domain	P
cassava4.1_031388m|PACid:17994188	cassava4.1_031388m	Disease resistance-responsive (dirigent-like protein) family protein	GP
cassava4.1_031396m|PACid:17962742	cassava4.1_031396m	Peroxidase superfamily protein	No
cassava4.1_031400m|PACid:17975144	cassava4.1_031400m	nitrate reductase 1	GP
cassava4.1_031411m|PACid:17987559	cassava4.1_031411m	Cytidine/deoxycytidylate deaminase family protein	No
cassava4.1_031414m|PACid:17970406	cassava4.1_031414m	Tetratricopeptide repeat (TPR)-like superfamily protein	No
cassava4.1_031441m|PACid:17991307	cassava4.1_031441m	Pentatricopeptide repeat (PPR) superfamily protein	No
cassava4.1_031445m|PACid:17992839	cassava4.1_031445m	Pectin lyase-like superfamily protein	No
cassava4.1_031461m|PACid:17973641	cassava4.1_031461m	histone deacetylase 14	GP
cassava4.1_031477m|PACid:17963916	cassava4.1_031477m	ABC transporter family protein	No
cassava4.1_031518m|PACid:17982410	cassava4.1_031518m	DNA repair-recombination protein (RAD50)	No
cassava4.1_031545m|PACid:17982772	cassava4.1_031545m	GHMP kinase family protein	No
cassava4.1_031549m|PACid:17980962	cassava4.1_031549m	Ubiquitin-like superfamily protein	No
cassava4.1_031552m|PACid:17975625	cassava4.1_031552m	DYNAMIN-like 1B	No
cassava4.1_031570m|PACid:17984083	cassava4.1_031570m	AMP-dependent synthetase and ligase family protein	GP
cassava4.1_031584m|PACid:17963174	cassava4.1_031584m	EXS (ERD1/XPR1/SYG1) family protein	No
cassava4.1_031602m|PACid:17978501	cassava4.1_031602m	MATE efflux family protein	No
cassava4.1_031606m|PACid:17987497	cassava4.1_031606m	Protein kinase superfamily protein	No
cassava4.1_031611m|PACid:17967013	cassava4.1_031611m	Auxin-responsive GH3 family protein	No

cassava4.1_031631m|PACid:17976236	cassava4.1_031631m	FASCICLIN-like arabinogalactan-protein 10	GP
cassava4.1_031640m|PACid:17967361	cassava4.1_031640m	Protein Transporter, Pam16	No
cassava4.1_031644m|PACid:17977005	cassava4.1_031644m		G
cassava4.1_031669m|PACid:17979235	cassava4.1_031669m	SAUR-like auxin-responsive protein family	No
cassava4.1_031676m|PACid:17967243	cassava4.1_031676m	AMP-dependent synthetase and ligase family protein	No
cassava4.1_031721m|PACid:17965889	cassava4.1_031721m	Protein of unknown function (DUF1685)	No
cassava4.1_031732m|PACid:17991717	cassava4.1_031732m	DNA-directed RNA polymerase family protein	G
cassava4.1_031755m|PACid:17980789	cassava4.1_031755m	organic cation/carnitine transporter4	No
cassava4.1_031766m|PACid:17984145	cassava4.1_031766m	pleiotropic drug resistance 1	No
cassava4.1_031782m|PACid:17967003	cassava4.1_031782m	RNA-binding (RRM/RBD/RNP motifs) family protein	No
cassava4.1_031788m|PACid:17993002	cassava4.1_031788m	ARABIDOPSIS TRITHORAX-RELATED PROTEIN 6	No
cassava4.1_031791m|PACid:17973682	cassava4.1_031791m	basic helix-loop-helix (bHLH) DNA-binding superfamily protein	No
cassava4.1_031792m|PACid:17991080	cassava4.1_031792m	2-oxoglutarate (2OG) and Fe(II)-dependent oxygenase superfamily protein	No
cassava4.1_031809m|PACid:17989865	cassava4.1_031809m	calmodulin-binding receptor-like cytoplasmic kinase 1	No
cassava4.1_031813m|PACid:17960184	cassava4.1_031813m	LOB domain-containing protein 15	No
cassava4.1_031815m|PACid:17992074	cassava4.1_031815m	GDP dissociation inhibitor family protein / Rab GTPase activator family protein	No
cassava4.1_031827m|PACid:17993034	cassava4.1_031827m	flavonol synthase 1	No
cassava4.1_031834m|PACid:17967001	cassava4.1_031834m	Glucose-methanol-choline (GMC) oxidoreductase family protein	No
cassava4.1_031837m|PACid:17983045	cassava4.1_031837m	DegP protease 10	No
cassava4.1_031845m|PACid:17964695	cassava4.1_031845m	Pectin lyase-like superfamily protein	No
cassava4.1_031855m|PACid:17987169	cassava4.1_031855m	anaphase promoting complex 6	No
cassava4.1_031863m|PACid:17989037	cassava4.1_031863m	PLAC8 family protein	No
cassava4.1_031870m|PACid:17971776	cassava4.1_031870m	lysine histidine transporter 1	No
cassava4.1_031883m|PACid:17990078	cassava4.1_031883m	HMG (high mobility group) box protein with ARID/BRIGHT DNA-binding domain	No
cassava4.1_031909m|PACid:17990727	cassava4.1_031909m	Alkaline-phosphatase-like family protein	GP
cassava4.1_031946m|PACid:17980835	cassava4.1_031946m	Peroxidase family protein	P
cassava4.1_031948m|PACid:17988885	cassava4.1_031948m	SET domain group 26	No
cassava4.1_031984m|PACid:17964443	cassava4.1_031984m	uroporphyrinogen-III synthase family protein	No
cassava4.1_032007m|PACid:17975603	cassava4.1_032007m	ACC synthase 1	No
cassava4.1_032015m|PACid:17985332	cassava4.1_032015m	P-loop containing nucleoside triphosphate hydrolases superfamily protein	No
cassava4.1_032024m|PACid:17974350	cassava4.1_032024m	cyclophilin 59	No
cassava4.1_032035m|PACid:17960238	cassava4.1_032035m	organic cation/carnitine transporter 3	No
cassava4.1_032037m|PACid:17961166	cassava4.1_032037m	SAUR-like auxin-responsive protein family	No
cassava4.1_032041m|PACid:17978365	cassava4.1_032041m	isocitrate lyase	No
cassava4.1_032043m|PACid:17988717	cassava4.1_032043m	alpha/beta-Hydrolases superfamily protein	No
cassava4.1_032045m|PACid:17991291	cassava4.1_032045m	PEBP (phosphatidylethanolamine-binding protein) family protein	No
cassava4.1_032046m|PACid:17991707	cassava4.1_032046m	Pseudouridine synthase family protein	No
cassava4.1_032050m|PACid:17960594	cassava4.1_032050m	Major facilitator superfamily protein	No
cassava4.1_032063m|PACid:17990935	cassava4.1_032063m	Tetratricopeptide repeat (TPR)-like superfamily protein	No

cassava4.1_032075m|PACid:17985448	cassava4.1_032075m	ribosomal protein L29 family protein	G
cassava4.1_032098m|PACid:17984953	cassava4.1_032098m	AGC (cAMP-dependent, cGMP-dependent and protein kinase C) kinase family protein	No
cassava4.1_032126m|PACid:17960579	cassava4.1_032126m	vesicle-associated membrane protein 727	G
cassava4.1_032132m|PACid:17963602	cassava4.1_032132m	FRAGILE HISTIDINE TRIAD	G
cassava4.1_032135m|PACid:17971574	cassava4.1_032135m		No
cassava4.1_032151m|PACid:17966437	cassava4.1_032151m	senescence-associated gene 12	No
cassava4.1_032158m|PACid:17980353	cassava4.1_032158m	SGNH hydrolase-type esterase superfamily protein	GP
cassava4.1_032205m|PACid:17989230	cassava4.1_032205m	emp24/gp25L/p24 family/GOLD family protein	No
cassava4.1_032206m|PACid:17980528	cassava4.1_032206m	Endonuclease/exonuclease/phosphatase family protein	No
cassava4.1_032217m|PACid:17982374	cassava4.1_032217m	protein kinase family protein	No
cassava4.1_032219m|PACid:17973656	cassava4.1_032219m	Pectin lyase-like superfamily protein	No
cassava4.1_032232m|PACid:17993811	cassava4.1_032232m	Aldolase-type TIM barrel family protein	P
cassava4.1_032246m|PACid:17964220	cassava4.1_032246m	MAP kinase kinase 6	G
cassava4.1_032252m|PACid:17990268	cassava4.1_032252m	sulfotransferase 2A	No
cassava4.1_032253m|PACid:17967876	cassava4.1_032253m	myb domain protein 105	No
cassava4.1_032268m|PACid:17992232	cassava4.1_032268m		No
cassava4.1_032273m|PACid:17975775	cassava4.1_032273m	RNA-binding (RRM/RBD/RNP motifs) family protein	No
cassava4.1_032283m|PACid:17962322	cassava4.1_032283m	methyltransferases	No
cassava4.1_032291m|PACid:17961341	cassava4.1_032291m	Glycosyl hydrolase family protein	No
cassava4.1_032293m|PACid:17985326	cassava4.1_032293m	structural maintenance of chromosomes 2	No
cassava4.1_032297m|PACid:17968049	cassava4.1_032297m	zinc transporter 10 precursor	No
cassava4.1_032301m|PACid:17982501	cassava4.1_032301m	nine-cis-epoxycarotenoid dioxygenase 6	No
cassava4.1_032323m|PACid:17969092	cassava4.1_032323m	galacturonosyltransferase 11	No
cassava4.1_032325m|PACid:17977115	cassava4.1_032325m	Subtilase family protein	GP
cassava4.1_032327m|PACid:17973427	cassava4.1_032327m	RING/U-box superfamily protein	No
cassava4.1_032346m|PACid:17990098	cassava4.1_032346m	U5 small nuclear ribonucleoprotein helicase, putative	P
cassava4.1_032348m|PACid:17990169	cassava4.1_032348m		GP
cassava4.1_032376m|PACid:17974150	cassava4.1_032376m	BANQUO 3	No
cassava4.1_032386m|PACid:17977990	cassava4.1_032386m	Cation efflux family protein	No
cassava4.1_032388m|PACid:17986984	cassava4.1_032388m		No
cassava4.1_032390m|PACid:17960348	cassava4.1_032390m	Tetratricopeptide repeat (TPR)-like superfamily protein	No
cassava4.1_032400m|PACid:17963190	cassava4.1_032400m	cytochrome P450, family 704, subfamily B, polypeptide 1	No
cassava4.1_032410m|PACid:17984742	cassava4.1_032410m	Leucine-rich repeat (LRR) family protein	P
cassava4.1_032424m|PACid:17979862	cassava4.1_032424m	erf domain protein 9	G
cassava4.1_032435m|PACid:17973448	cassava4.1_032435m	Peroxidase superfamily protein	No
cassava4.1_032447m|PACid:17982172	cassava4.1_032447m	bacterial hemolysin-related	No
cassava4.1_032456m|PACid:17988581	cassava4.1_032456m	nitrate transporter 1.5	No
cassava4.1_032463m|PACid:17969523	cassava4.1_032463m	RAD3-like DNA-binding helicase protein	No
cassava4.1_032464m|PACid:17972702	cassava4.1_032464m	glucuronidase 3	P

cassava4.1_032467m|PACid:17984605	cassava4.1_032467m	Metal-dependent phosphohydrolase	No
cassava4.1_032477m|PACid:17967686	cassava4.1_032477m	Peroxidase superfamily protein	No
cassava4.1_032482m|PACid:17986409	cassava4.1_032482m	BTB/POZ domain-containing protein	No
cassava4.1_032503m|PACid:17987368	cassava4.1_032503m	Pectin lyase-like superfamily protein	No
cassava4.1_032506m|PACid:17990722	cassava4.1_032506m	Protein kinase superfamily protein	No
cassava4.1_032514m|PACid:17984770	cassava4.1_032514m	xyloglucan endotransglucosylase/hydrolase 30	G
cassava4.1_032518m|PACid:17973939	cassava4.1_032518m	beta glucosidase 41	No
cassava4.1_032526m|PACid:17989221	cassava4.1_032526m	RHO guanyl-nucleotide exchange factor 7	No
cassava4.1_032535m|PACid:17970814	cassava4.1_032535m	tRNA synthetase class I (I, L, M and V) family protein	P
cassava4.1_032581m|PACid:17960208	cassava4.1_032581m	NADH dehydrogenase (ubiquinone)s	G
cassava4.1_032592m|PACid:17970873	cassava4.1_032592m	oligopeptide transporter 7	No
cassava4.1_032600m|PACid:17967101	cassava4.1_032600m	LAG1 homologue 2	No
cassava4.1_032607m|PACid:17982916	cassava4.1_032607m	bZIP transcription factor family protein	No
cassava4.1_032628m|PACid:17988853	cassava4.1_032628m	glycerol-3-phosphate acyltransferase 5	No
cassava4.1_032634m|PACid:17964214	cassava4.1_032634m	translocase inner membrane subunit 17-2	No
cassava4.1_032640m|PACid:17993928	cassava4.1_032640m	polyamine oxidase 1	No
cassava4.1_032641m|PACid:17965094	cassava4.1_032641m	SWAP (Suppressor-of-White-APricot)/surp domain-containing protein / ubiquitin family protein	GP
cassava4.1_032646m|PACid:17962100	cassava4.1_032646m	YUCCA 3	No
cassava4.1_032666m|PACid:17971666	cassava4.1_032666m	GDSL-like Lipase/Acylhydrolase superfamily protein	No
cassava4.1_032668m|PACid:17970977	cassava4.1_032668m	RING/U-box superfamily protein	No
cassava4.1_032702m|PACid:17979783	cassava4.1_032702m	ABC-2 type transporter family protein	No
cassava4.1_032708m|PACid:17991833	cassava4.1_032708m	Pentatricopeptide repeat (PPR) superfamily protein	No
cassava4.1_032709m|PACid:17967316	cassava4.1_032709m	Transducin/WD40 repeat-like superfamily protein	No
cassava4.1_032721m|PACid:17970092	cassava4.1_032721m	pectinesterase 11	No
cassava4.1_032740m|PACid:17960842	cassava4.1_032740m	Remorin family protein	No
cassava4.1_032741m|PACid:17989827	cassava4.1_032741m		No
cassava4.1_032742m|PACid:17985375	cassava4.1_032742m	Protein of unknown function (DUF579)	No
cassava4.1_032747m|PACid:17974007	cassava4.1_032747m	Protein kinase superfamily protein	No
cassava4.1_032749m|PACid:17988956	cassava4.1_032749m	carotenoid cleavage dioxygenase 7	No
cassava4.1_032767m|PACid:17989599	cassava4.1_032767m	O-fucosyltransferase family protein	No
cassava4.1_032768m|PACid:17971497	cassava4.1_032768m	RECQ helicase L2	No
cassava4.1_032773m|PACid:17962389	cassava4.1_032773m	Protein of unknown function (DUF620)	No
cassava4.1_032779m|PACid:17979385	cassava4.1_032779m		No
cassava4.1_032788m|PACid:17972201	cassava4.1_032788m	Pentatricopeptide repeat (PPR-like) superfamily protein	No
cassava4.1_032792m|PACid:17991299	cassava4.1_032792m	SU(VAR)3-9 homolog 4	G
cassava4.1_032798m|PACid:17992082	cassava4.1_032798m	expansin 12	No
cassava4.1_032817m|PACid:17960751	cassava4.1_032817m	PEBP (phosphatidylethanolamine-binding protein) family protein	No
cassava4.1_032824m|PACid:17961598	cassava4.1_032824m	Tyrosine phosphatase family protein	No
cassava4.1_032831m|PACid:17960356	cassava4.1_032831m	myb domain protein 6	No

cassava4.1_032835m|PACid:17978362	cassava4.1_032835m	pinoresinol reductase 1	No
cassava4.1_032838m|PACid:17979532	cassava4.1_032838m	Pentatricopeptide repeat (PPR) superfamily protein	G
cassava4.1_032851m|PACid:17993140	cassava4.1_032851m	CYCLIN D4;1	No
cassava4.1_032855m|PACid:17962930	cassava4.1_032855m	histidine phosphotransfer protein 6	No
cassava4.1_032856m|PACid:17975455	cassava4.1_032856m	arginine-rich cyclin 1	No
cassava4.1_032857m|PACid:17966646	cassava4.1_032857m		G
cassava4.1_032874m|PACid:17967781	cassava4.1_032874m	Haloacid dehalogenase-like hydrolase (HAD) superfamily protein	No
cassava4.1_032878m|PACid:17962050	cassava4.1_032878m	Splicing factor 3B subunit 5/RDS3 complex subunit 10	G
cassava4.1_032885m|PACid:17987507	cassava4.1_032885m	SGNH hydrolase-type esterase superfamily protein	No
cassava4.1_032889m|PACid:17977315	cassava4.1_032889m	nuclear factor Y, subunit B6	No
cassava4.1_032917m|PACid:17965672	cassava4.1_032917m	exostosin family protein	No
cassava4.1_032925m|PACid:17990300	cassava4.1_032925m	actin-related protein 8	G
cassava4.1_032951m|PACid:17988572	cassava4.1_032951m	NAD(P)-binding Rossmann-fold superfamily protein	P
cassava4.1_032961m|PACid:17964585	cassava4.1_032961m	cyclin p4;1	No
cassava4.1_032962m|PACid:17968079	cassava4.1_032962m	ketose-bisphosphate aldolase class-II family protein	GP
cassava4.1_032993m|PACid:17967238	cassava4.1_032993m	chloroplast sulfur E	No
cassava4.1_032996m|PACid:17991980	cassava4.1_032996m	non-intrinsic ABC protein 7	GP
cassava4.1_033017m|PACid:17981191	cassava4.1_033017m	GDSL-like Lipase/Acylhydrolase superfamily protein	No
cassava4.1_033019m|PACid:17966164	cassava4.1_033019m	mannosyltransferase family protein	G
cassava4.1_033029m|PACid:17983595	cassava4.1_033029m	EID1-like 2	No
cassava4.1_033034m|PACid:17985108	cassava4.1_033034m	Tetratricopeptide repeat (TPR)-like superfamily protein	No
cassava4.1_033036m|PACid:17968321	cassava4.1_033036m	heptahelical protein 4	No
cassava4.1_033038m|PACid:17973947	cassava4.1_033038m	RNA-binding (RRM/RBD/RNP motifs) family protein	No
cassava4.1_033041m|PACid:17975494	cassava4.1_033041m	Rhodanese/Cell cycle control phosphatase superfamily protein	No
cassava4.1_033058m|PACid:17972603	cassava4.1_033058m	Leucine-rich repeat (LRR) family protein	No
cassava4.1_033064m|PACid:17983047	cassava4.1_033064m	beta-hexosaminidase 3	P
cassava4.1_033085m|PACid:17991764	cassava4.1_033085m	DNA polymerase epsilon catalytic subunit	No
cassava4.1_033096m|PACid:17963878	cassava4.1_033096m	Serine carboxypeptidase S28 family protein	No
cassava4.1_033102m|PACid:17988074	cassava4.1_033102m	Protein phosphatase 2C family protein	No
cassava4.1_033115m|PACid:17975373	cassava4.1_033115m	basic helix-loop-helix (bHLH) DNA-binding family protein	No
cassava4.1_033117m|PACid:17977542	cassava4.1_033117m	Integrin-linked protein kinase family	No
cassava4.1_033148m|PACid:17983279	cassava4.1_033148m	Nucleoporin autopeptidase	GP
cassava4.1_033167m|PACid:17969182	cassava4.1_033167m	actin binding	No
cassava4.1_033177m|PACid:17960703	cassava4.1_033177m	Leucine-rich repeat transmembrane protein kinase	No
cassava4.1_033196m|PACid:17993614	cassava4.1_033196m	NAD(P)-linked oxidoreductase superfamily protein	No
cassava4.1_033228m|PACid:17988531	cassava4.1_033228m	pumilio 7	No
cassava4.1_033277m|PACid:17980252	cassava4.1_033277m	Zinc finger C-x8-C-x5-C-x3-H type family protein	No
cassava4.1_033279m|PACid:17960336	cassava4.1_033279m		G
cassava4.1_033296m|PACid:17973477	cassava4.1_033296m	Family of unknown function (DUF662)	No

cassava4.1_033300m|PACid:17986606	cassava4.1_033300m	Tetratricopeptide repeat (TPR)-like superfamily protein	No
cassava4.1_033304m|PACid:17983729	cassava4.1_033304m	cytochrome c biogenesis protein family	No
cassava4.1_033319m|PACid:17969627	cassava4.1_033319m	Major facilitator superfamily protein	No
cassava4.1_033332m|PACid:17963684	cassava4.1_033332m	pseudo-response regulator 9	G
cassava4.1_033334m|PACid:17989426	cassava4.1_033334m	SET-domain containing protein lysine methyltransferase family protein	No
cassava4.1_033337m|PACid:17965654	cassava4.1_033337m	ARM repeat superfamily protein	No
cassava4.1_033341m|PACid:17966144	cassava4.1_033341m	Beta-ketoacyl synthase	No
cassava4.1_033356m|PACid:17977695	cassava4.1_033356m	cationic amino acid transporter 7	No
cassava4.1_033358m|PACid:17974290	cassava4.1_033358m	C2 calcium/lipid-binding plant phosphoribosyltransferase family protein	No
cassava4.1_033375m|PACid:17990985	cassava4.1_033375m	UDP-D-glucose/UDP-D-galactose 4-epimerase 5	No
cassava4.1_033378m|PACid:17970207	cassava4.1_033378m	Ubiquitin carboxyl-terminal hydrolase family protein	G
cassava4.1_033379m|PACid:17967592	cassava4.1_033379m	Pectin lyase-like superfamily protein	No
cassava4.1_033381m|PACid:17979423	cassava4.1_033381m	Putative methyltransferase family protein	No
cassava4.1_033385m|PACid:17990610	cassava4.1_033385m	Insulinase (Peptidase family M16) family protein	GP
cassava4.1_033395m|PACid:17963243	cassava4.1_033395m	NAD(P)-binding Rossmann-fold superfamily protein	No
cassava4.1_033411m|PACid:17988301	cassava4.1_033411m	phosphoenolpyruvate carboxykinase 1	No
cassava4.1_033414m|PACid:17975909	cassava4.1_033414m	Protein of unknown function (DUF167)	No
cassava4.1_033418m|PACid:17980555	cassava4.1_033418m	ADP-ribosylation factor A1F	P
cassava4.1_033427m|PACid:17990444	cassava4.1_033427m	lipid-binding serum glycoprotein family protein	No
cassava4.1_033434m|PACid:17973562	cassava4.1_033434m	Protein kinase protein with adenine nucleotide alpha hydrolases-like domain	No
cassava4.1_033437m|PACid:17978785	cassava4.1_033437m	pectin methylesterase 31	GP
cassava4.1_033469m|PACid:17981271	cassava4.1_033469m	Protein kinase superfamily protein	No
cassava4.1_033488m|PACid:17963431	cassava4.1_033488m	Acid phosphatase/vanadium-dependent haloperoxidase-related protein	No
cassava4.1_033493m|PACid:17970307	cassava4.1_033493m		No
cassava4.1_033497m|PACid:17975044	cassava4.1_033497m	DEA(D/H)-box RNA helicase family protein	G
cassava4.1_033508m|PACid:17979420	cassava4.1_033508m	alpha/beta-Hydrolases superfamily protein	No
cassava4.1_033525m|PACid:17983780	cassava4.1_033525m	HSP20-like chaperones superfamily protein	No
cassava4.1_033528m|PACid:17969917	cassava4.1_033528m	eukaryotic initiation factor 3 gamma subunit family protein	No
cassava4.1_033529m|PACid:17984928	cassava4.1_033529m	K+ transporter 1	No
cassava4.1_033544m|PACid:17982887	cassava4.1_033544m		No
cassava4.1_033556m|PACid:17985557	cassava4.1_033556m	Leucine-rich repeat transmembrane protein kinase	No
cassava4.1_033564m|PACid:17965392	cassava4.1_033564m	P-loop containing nucleoside triphosphate hydrolases superfamily protein	No
cassava4.1_033575m|PACid:17980024	cassava4.1_033575m	SET domain protein 35	No
cassava4.1_033597m|PACid:17977937	cassava4.1_033597m	Ribosome associated membrane protein RAMP4	No
cassava4.1_033602m|PACid:17964620	cassava4.1_033602m		No
cassava4.1_033612m|PACid:17962453	cassava4.1_033612m	COBRA-like protein 6 precursor	No
cassava4.1_033630m|PACid:17972128	cassava4.1_033630m		G
cassava4.1_033631m|PACid:17977884	cassava4.1_033631m	dephospho-CoA kinase family	No
cassava4.1_033635m|PACid:17977264	cassava4.1_033635m	Leucine-rich repeat protein kinase family protein	No

cassava4.1_033636m|PACid:17976901	cassava4.1_033636m	Myzus persicae-induced lipase 1	No
cassava4.1_033646m|PACid:17967813	cassava4.1_033646m	Haloacid dehalogenase-like hydrolase (HAD) superfamily protein	No
cassava4.1_033647m|PACid:17983299	cassava4.1_033647m	PLC-like phosphodiesterases superfamily protein	No
cassava4.1_033699m|PACid:17976412	cassava4.1_033699m	MUTS-like protein 4	No
cassava4.1_033703m|PACid:17965621	cassava4.1_033703m	Myosin family protein with Dil domain	No
cassava4.1_033708m|PACid:17966056	cassava4.1_033708m	Ribosomal L18p/L5e family protein	No
cassava4.1_033721m|PACid:17978783	cassava4.1_033721m	Calcium/lipid-binding (CaLB) phosphatase	No
cassava4.1_033737m|PACid:17971057	cassava4.1_033737m		G
cassava4.1_033741m|PACid:17980518	cassava4.1_033741m	PDI-like 5-4	GP
cassava4.1_033749m|PACid:17963147	cassava4.1_033749m	Protein of unknown function (DUF707)	No
cassava4.1_033750m|PACid:17988806	cassava4.1_033750m	RAB GTPase homolog H1E	P
cassava4.1_033765m|PACid:17988733	cassava4.1_033765m	tetratricopeptide repeat (TPR)-containing protein	No
cassava4.1_033774m|PACid:17963262	cassava4.1_033774m	RNA 2\'-phosphotransferase, Tpt1 / KptA family	G
cassava4.1_033783m|PACid:17983227	cassava4.1_033783m	Phosphoinositide-specific phospholipase C family protein	No
cassava4.1_033794m|PACid:17993012	cassava4.1_033794m	Pentatricopeptide repeat (PPR) superfamily protein	G
cassava4.1_033799m|PACid:17975632	cassava4.1_033799m	D-arabinono-1,4-lactone oxidase family protein	No
cassava4.1_033804m|PACid:17992612	cassava4.1_033804m	DNAse I-like superfamily protein	No
cassava4.1_033809m|PACid:17979271	cassava4.1_033809m	alpha/beta-Hydrolases superfamily protein	No
cassava4.1_033814m|PACid:17994173	cassava4.1_033814m	laccase 3	No
cassava4.1_033827m|PACid:17970286	cassava4.1_033827m	beta-hexosaminidase 1	GP
cassava4.1_033850m|PACid:17970077	cassava4.1_033850m	AMMECR1 family	No
cassava4.1_033858m|PACid:17961795	cassava4.1_033858m	ATPase E1-E2 type family protein / haloacid dehalogenase-like hydrolase family protein	No
cassava4.1_033859m|PACid:17971250	cassava4.1_033859m	alpha/beta-Hydrolases superfamily protein	No
cassava4.1_033860m|PACid:17960904	cassava4.1_033860m	FAD/NAD(P)-binding oxidoreductase family protein	GP
cassava4.1_033865m|PACid:17960498	cassava4.1_033865m	Protein phosphatase 2C family protein	No
cassava4.1_033875m|PACid:17970735	cassava4.1_033875m	SGNH hydrolase-type esterase superfamily protein	No
cassava4.1_033877m|PACid:17976813	cassava4.1_033877m	Tetratricopeptide repeat (TPR)-like superfamily protein	G
cassava4.1_033878m|PACid:17969787	cassava4.1_033878m	K+ uptake permease 7	GP
cassava4.1_033879m|PACid:17988308	cassava4.1_033879m	Aldolase-type TIM barrel family protein	No
cassava4.1_033888m|PACid:17969068	cassava4.1_033888m	K-box region and MADS-box transcription factor family protein	No
cassava4.1_033890m|PACid:17971863	cassava4.1_033890m	RNA-binding (RRM/RBD/RNP motifs) family protein	G
cassava4.1_033893m|PACid:17972382	cassava4.1_033893m	amine oxidase 1	No
cassava4.1_033905m|PACid:17964754	cassava4.1_033905m	pantothenate kinase 2	G
cassava4.1_033908m|PACid:17969441	cassava4.1_033908m	HIS HF	P
cassava4.1_033937m|PACid:17987838	cassava4.1_033937m	Protein kinase superfamily protein	No
cassava4.1_033958m|PACid:17964956	cassava4.1_033958m	glycosyl hydrolase 9B8	No
cassava4.1_033965m|PACid:17967964	cassava4.1_033965m	Nucleotide-diphospho-sugar transferases superfamily protein	P
cassava4.1_033968m|PACid:17969699	cassava4.1_033968m	GRAS family transcription factor family protein	G
cassava4.1_033978m|PACid:17961035	cassava4.1_033978m	Integral membrane Yip1 family protein	No

cassava4.1_033999m|PACid:17979478	cassava4.1_033999m	Eukaryotic aspartyl protease family protein	G
cassava4.1_034020m|PACid:17976026	cassava4.1_034020m	beta-galactosidase 17	GP
cassava4.1_034033m|PACid:17993753	cassava4.1_034033m	Tetratricopeptide repeat (TPR)-like superfamily protein	No
cassava4.1_034057m|PACid:17989136	cassava4.1_034057m	GRAS family transcription factor	G
cassava4.1_034063m|PACid:17962776	cassava4.1_034063m	Calcium-binding EF-hand family protein	No
cassava4.1_034082m|PACid:17972601	cassava4.1_034082m	Core-2/I-branching beta-1,6-N-acetylglucosaminyltransferase family protein	G
cassava4.1_034097m|PACid:17962215	cassava4.1_034097m	sugar transporter 14	No
cassava4.1_034101m|PACid:17972979	cassava4.1_034101m	Plant protein of unknown function (DUF828)	No
cassava4.1_034111m|PACid:17987774	cassava4.1_034111m	ATPase, F1 complex, gamma subunit protein	No
cassava4.1_034113m|PACid:17969978	cassava4.1_034113m	Concanavalin A-like lectin protein kinase family protein	No
cassava4.1_034125m|PACid:17973527	cassava4.1_034125m	Rho GTPase activating protein with PAK-box/P21-Rho-binding domain	No
cassava4.1_034126m|PACid:17966588	cassava4.1_034126m	Protein of unknown function (DUF579)	G
cassava4.1_034129m|PACid:17978492	cassava4.1_034129m	Fatty acid/sphingolipid desaturase	G
cassava4.1_034139m|PACid:17992188	cassava4.1_034139m	lectin protein kinase family protein	GP
cassava4.1_034143m|PACid:17991762	cassava4.1_034143m	arogenate dehydratase 6	GP
cassava4.1_034144m|PACid:17990203	cassava4.1_034144m	S-adenosyl-L-methionine-dependent methyltransferases superfamily protein	No
cassava4.1_034145m|PACid:17980619	cassava4.1_034145m	Leucine-rich repeat protein kinase family protein	No
cassava4.1_034147m|PACid:17986357	cassava4.1_034147m	Pentatricopeptide repeat (PPR) superfamily protein	No
cassava4.1_034153m|PACid:17984581	cassava4.1_034153m	Tetratricopeptide repeat (TPR)-like superfamily protein	No
cassava4.1_034154m|PACid:17976648	cassava4.1_034154m	Leucine-rich repeat (LRR) family protein	No
cassava4.1_034158m|PACid:17977352	cassava4.1_034158m	plant glycogenin-like starch initiation protein 3	No
cassava4.1_034167m|PACid:17982079	cassava4.1_034167m	Phosphoenolpyruvate carboxylase family protein	No
cassava4.1_034173m|PACid:17965028	cassava4.1_034173m	ARM repeat superfamily protein	No
cassava4.1_034181m|PACid:17991536	cassava4.1_034181m	AAA-type ATPase family protein	G
cassava4.1_034183m|PACid:17985537	cassava4.1_034183m	Transducin/WD40 repeat-like superfamily protein	No
cassava4.1_034184m|PACid:17975719	cassava4.1_034184m	P-loop containing nucleoside triphosphate hydrolases superfamily protein	G
cassava4.1_034193m|PACid:17983821	cassava4.1_034193m	Phototropic-responsive NPH3 family protein	No
cassava4.1_034194m|PACid:17982055	cassava4.1_034194m	Tetratricopeptide repeat (TPR)-like superfamily protein	G
cassava4.1_034206m|PACid:17993458	cassava4.1_034206m	Acyl-CoA N-acyltransferases (NAT) superfamily protein	No
cassava4.1_034211m|PACid:17978907	cassava4.1_034211m	RING/U-box superfamily protein	GP
cassava4.1_034217m|PACid:17987620	cassava4.1_034217m	Sec1/munc18-like (SM) proteins superfamily	No
cassava4.1_034218m|PACid:17960342	cassava4.1_034218m	C2 calcium/lipid-binding plant phosphoribosyltransferase family protein	No
cassava4.1_034224m|PACid:17987625	cassava4.1_034224m	RING/U-box superfamily protein	No
cassava4.1_034229m|PACid:17988391	cassava4.1_034229m	Protein kinase superfamily protein	No
cassava4.1_034231m|PACid:17983414	cassava4.1_034231m	FAD-binding Berberine family protein	GP
cassava4.1_034243m|PACid:17961914	cassava4.1_034243m	heat-shock protein 70T-2	P
cassava4.1_034261m|PACid:17979406	cassava4.1_034261m	cytochrome BC1 synthesis	No
cassava4.1_034262m|PACid:17971338	cassava4.1_034262m	Calcium-dependent lipid-binding (CaLB domain) plant phosphoribosyltransferase family protein	GP
cassava4.1_034278m|PACid:17979929	cassava4.1_034278m	C2 calcium/lipid-binding plant phosphoribosyltransferase family protein	GP

cassava4.1_034283m|PACid:17985679	cassava4.1_034283m	serine-rich protein-related	No
cassava4.1_034286m|PACid:17990533	cassava4.1_034286m	chromatin remodeling 4	No
cassava4.1_034298m|PACid:17963821	cassava4.1_034298m	P-loop containing nucleoside triphosphate hydrolases superfamily protein	GP
cassava4.1_034300m|PACid:17993547	cassava4.1_034300m	AT-hook motif nuclear-localized protein 20	G
cassava4.1_034305m|PACid:17964116	cassava4.1_034305m	PYR1-like 6	G
cassava4.1_034306m|PACid:17978441	cassava4.1_034306m	Cation efflux family protein	No
cassava4.1_034308m|PACid:17971311	cassava4.1_034308m	NIMA-related kinase 5	No
cassava4.1_034310m|PACid:17964219	cassava4.1_034310m	Leucine-rich repeat (LRR) family protein	G
cassava4.1_034315m|PACid:17983269	cassava4.1_034315m	cytochrome P450, family 77, subfamily A, polypeptide 4	No
cassava4.1_034329m|PACid:17973503	cassava4.1_034329m	arogenate dehydratase 6	No
cassava4.1_034339m|PACid:17967612	cassava4.1_034339m	serine acetyltransferase 2;2	No
cassava4.1_034340m|PACid:17985078	cassava4.1_034340m	sacI homology domain-containing protein / WW domain-containing protein	No
cassava4.1_034345m|PACid:17991112	cassava4.1_034345m	GDSL-motif lipase/hydrolase 6	No
cassava4.1_034348m|PACid:17993635	cassava4.1_034348m	DEAD/DEAH box helicase, putative	No
cassava4.1_034351m|PACid:17980339	cassava4.1_034351m	golgi nucleotide sugar transporter 4	G
cassava4.1_034357m|PACid:17992495	cassava4.1_034357m	exocyst subunit exo70 family protein D1	GP
cassava4.1_034372m|PACid:17982049	cassava4.1_034372m	RmlC-like cupins superfamily protein	No
cassava4.1_034374m|PACid:17982513	cassava4.1_034374m	Tetratricopeptide repeat (TPR)-like superfamily protein	No
cassava4.1_034376m|PACid:17971199	cassava4.1_034376m	polyamine oxidase 5	GP
cassava4.1_034378m|PACid:17977639	cassava4.1_034378m	Thiamine pyrophosphate dependent pyruvate decarboxylase family protein	GP
cassava4.1_034379m|PACid:17985098	cassava4.1_034379m	nine-cis-epoxycarotenoid dioxygenase 3	G
cassava4.1_034382m|PACid:17962467	cassava4.1_034382m	Protein kinase superfamily protein	No
cassava4.1_034390m|PACid:17986627	cassava4.1_034390m	Octicosapeptide/Phox/Bem1p (PB1) domain-containing protein / tetratricopeptide repeat (TPR)-containing protein	GP
cassava4.1_034392m|PACid:17990841	cassava4.1_034392m	Transducin/WD40 repeat-like superfamily protein	No
cassava4.1_034405m|PACid:17985960	cassava4.1_034405m	DOMON domain-containing protein / dopamine beta-monooxygenase N-terminal domain-containing protein	G
cassava4.1_034406m|PACid:17967791	cassava4.1_034406m	cytochrome P450, family 710, subfamily A, polypeptide 1	No
cassava4.1_034413m|PACid:17971931	cassava4.1_034413m	Pentatricopeptide repeat (PPR) superfamily protein	No
cassava4.1_034420m|PACid:17976307	cassava4.1_034420m	Major facilitator superfamily protein	No
cassava4.1_034440m|PACid:17971685	cassava4.1_034440m	ammonium transporter 1;1	No
cassava4.1_034445m|PACid:17959730	cassava4.1_034445m	Homeodomain-like protein with RING/FYVE/PHD-type zinc finger domain	No
cassava4.1_034446m|PACid:17970185	cassava4.1_034446m	Subtilase family protein	G
cassava4.1_034450m|PACid:17989375	cassava4.1_034450m	Rubredoxin-like superfamily protein	No
cassava4.1_034454m|PACid:17983016	cassava4.1_034454m	pyridoxine biosynthesis 1.2	No
cassava4.1_034464m|PACid:17964039	cassava4.1_034464m	S-adenosyl-L-methionine-dependent methyltransferases superfamily protein	No
cassava4.1_034465m|PACid:17973456	cassava4.1_034465m	Calcium-dependent lipid-binding (CaLB domain) family protein	GP
cassava4.1_034468m|PACid:17991902	cassava4.1_034468m	Tetratricopeptide repeat (TPR)-like superfamily protein	No
sp|B1NWD0|PSBA_MANES	cassava4.1_031236m	Photosystem Q(B) protein (EC 1.10.3.9) (32 kDa thylakoid membrane protein) (Photosystem II protein D1)	P
sp|B1NWD5|ATPA_MANES	cassava4.1_028060m	ATP synthase subunit alpha, chloroplastic (EC 3.6.3.14) (ATP synthase F1 sector subunit alpha)	P
sp|B1NWD7|ATPH_MANES	No_in_Phytozome	ATP synthase subunit c, chloroplastic (ATP synthase F(0) sector subunit c) (F-ATPase subunit c)	No

sp|B1NWD9|RR2_MANES	cassava4.1_032466m	30S ribosomal protein S2, chloroplastic	No
sp|B1NWE1|RPOC1_MANES	cassava4.1_032799m	DNA-directed RNA polymerase subunit beta' (EC 2.7.7.6) (PEP) (Plastid-encoded RNA polymerase subunit beta')	No
sp|B1NWE5|PSBD_MANES	cassava4.1_031110m	Photosystem II D2 protein (PSII D2 protein) (EC 1.10.3.9) (Photosystem Q(A) protein)	P
sp|B1NWE6|PSBC_MANES	cassava4.1_022561m	Photosystem II CP43 chlorophyll apoprotein (PSII 43 kDa protein) (Photosystem II 44 kDa reaction center protein) (Protein	P
sp|B1NWE8|RR14_MANES	cassava4.1_030985m	30S ribosomal protein S14, chloroplastic	G
sp|B1NWF7|ATPB_MANES	cassava4.1_004552m	ATP synthase subunit beta, chloroplastic (EC 3.6.3.14) (ATP synthase F1 sector subunit beta)	P
sp|B1NWF8|RBL_MANES	cassava4.1_017330m	Ribulose bisphosphate carboxylase large chain (RuBisCO large subunit) (EC 4.1.1.39)	P
sp|B1NWG3|CYF_MANES	cassava4.1_022160m	Apocytochrome f	GP
sp|B1NWI3|RR11_MANES	cassava4.1_025357m	30S ribosomal protein S11, chloroplastic	No
sp|B1NWI6|RK14_MANES	No_in_Phytozome	50S ribosomal protein L14, chloroplastic	No
sp|B1NWI7|RK16_MANES	No_in_Phytozome	50S ribosomal protein L16, chloroplastic	No
sp|B1NWI9|RK22_MANES	No_in_Phytozome	50S ribosomal protein L22, chloroplastic	No
sp|B1NWJ0|RR19_MANES	No_in_Phytozome	30S ribosomal protein S19, chloroplastic	No
sp|B1NWJ1|RK2_MANES	No_in_Phytozome	50S ribosomal protein L2, chloroplastic	No
sp|B1NWJ5|RR7_MANES	cassava4.1_030690m	30S ribosomal protein S7, chloroplastic	No
sp|B1NWK1|NU4LC_MANES	No_in_Phytozome	NAD(P)H-quinone oxidoreductase subunit 4L, chloroplastic (EC 1.6.5.-)	No
sp|B1NWK5|NDHH_MANES	cassava4.1_033831m	NAD(P)H-quinone oxidoreductase subunit H, chloroplastic (EC 1.6.5.-)	No
sp|O49169|EF1A_MANES	cassava4.1_007545m	Elongation factor 1-alpha (EF-1-alpha)	GP
sp|Q42915|RBS_MANES	cassava4.1_017243m	Ribulose bisphosphate carboxylase small chain, chloroplastic (RuBisCO small subunit) (EC 4.1.1.39)	G
tr|A0SVL9|A0SVL9_MANES	cassava4.1_010983m	Beta-1,3-glucanase (Fragment)	GP
tr|A0SWU5|A0SWU5_MANES	cassava4.1_012282m	1-amino-cyclopropane-1-carboxylic acid oxidase 3	No
tr|A0ZQS3|A0ZQS3_MANES	cassava4.1_017330m	Ribulose-1,5-bisphosphate carboxylase/oxygenase large subunit (Fragment)	No
tr|B0FTX3|B0FTX3_MANES	cassava4.1_009789m	Cysteine synthase (EC 2.5.1.47)	No
tr|B1NWC9|B1NWC9_MANES	No_in_Phytozome	30S ribosomal protein S12, chloroplastic	No
tr|B1NWE2|B1NWE2_MANES	cassava4.1_025998m	DNA-directed RNA polymerase subunit beta (EC 2.7.7.6) (PEP) (Plastid-encoded RNA polymerase subunit beta)	No
tr|B1NWE9|B1NWE9_MANES	cassava4.1_029388m	Photosystem I P700 chlorophyll a apoprotein A2 (EC 1.97.1.12) (PSI-B) (PsaB)	P
tr|B1NWF0|B1NWF0_MANES	cassava4.1_027296m	Photosystem I P700 chlorophyll a apoprotein A1 (EC 1.97.1.12) (PSI-A) (PsaA)	G
tr|B1NWF2|B1NWF2_MANES	No_in_Phytozome	30S ribosomal protein S4, chloroplastic	No
tr|B1NWF6|B1NWF6_MANES	cassava4.1_004552m	ATP synthase epsilon chain, chloroplastic (ATP synthase F1 sector epsilon subunit) (F-ATPase epsilon subunit)	P
tr|B1NWF9|B1NWF9_MANES	cassava4.1_033680m	Acetyl-coenzyme A carboxylase carboxyl transferase (ACCase) subunit beta, chloroplastic (EC 6.4.1.2)	No
tr|B1NWH5|B1NWH5_MANES	cassava4.1_025353m	ATP-dependent Clp protease proteolytic subunit (EC 3.4.21.92) (Endopeptidase Clp)	No
tr|B1NWI0|B1NWI0_MANES	cassava4.1_024794m	Cytochrome b6	No
tr|B1NWI2|B1NWI2_MANES	cassava4.1_025357m	DNA-directed RNA polymerase subunit alpha (PEP) (EC 2.7.7.6) (	No
tr|B1NWI5|B1NWI5_MANES	No_in_Phytozome	30S ribosomal protein S8, chloroplastic	No
tr|B1NWI8|B1NWI8_MANES	cassava4.1_020914m	30S ribosomal protein S3, chloroplastic	P
tr|B1NWK3|B1NWK3_MANES	cassava4.1_021875m	NAD(P)H-quinone oxidoreductase subunit I, chloroplastic (EC 1.6.5.-)	No
tr|B1NWK4|B1NWK4_MANES	cassava4.1_021730m	NAD(P)H-quinone oxidoreductase subunit 1, chloroplastic (EC 1.6.5.-)	No
tr|B2M0U5|B2M0U5_MANES	cassava4.1_013498m	Aquaporin	G
tr|B3SRP2|B3SRP2_MANES	cassava4.1_004619m	Starch synthase isoform I (EC 2.4.1.21)	G

tr|B3SRP3|B3SRP3_MANES	cassava4.1_002278m	Starch synthase isoform II (EC 2.4.1.21)	No
tr|C5H5H1|C5H5H1_MANES	cassava4.1_003897m	Carotenoid isomerase (EC 5.-.-.-) (Fragment)	G
tr|D2XSJ1|D2XSJ1_MANES	cassava4.1_031179m	PT1	No
tr|D2XSJ3|D2XSJ3_MANES	cassava4.1_006465m	AMT2	G
tr|D3VW90|D3VW90_MANES	cassava4.1_008945m	Isoamylase isoform 3 (Fragment)	G
tr|D6BCR4|D6BCR4_MANES	cassava4.1_004359m	Phytoene desaturase (Fragment)	G
tr|E0XGY1|E0XGY1_MANES	cassava4.1_008121m	Phytoene synthase 2 (EC 2.5.1.-)	No
tr|E2DQG2|E2DQG2_MANES	cassava4.1_004265m	Zeta-carotene desaturase (Fragment)	G
tr|E2DQG5|E2DQG5_MANES	cassava4.1_012536m	Beta-carotene hydroxylase (Fragment)	G
tr|E2DQG6|E2DQG6_MANES	cassava4.1_022943m	Carotenoid cleavage dioxygenase 1	G
tr|G5DC09|G5DC09_MANES	cassava4.1_002913m	Neutral/alkaline invertase	G
tr|G5DCB5|G5DCB5_MANES	cassava4.1_002180m	ARC5 protein	G
tr|H6V7I5|H6V7I5_MANES	cassava4.1_004485m	Cell wall invertase	No
tr|H9AY26|H9AY26_MANES	cassava4.1_015317m	MinE protein	No
tr|H9AY27|H9AY27_MANES	cassava4.1_011379m	MinD protein	G
tr|H9AY28|H9AY28_MANES	cassava4.1_008560m	FtsZ1 protein	G
tr|H9AY29|H9AY29_MANES	cassava4.1_006511m	FtsZ2 protein	No
tr|I0CL53|I0CL53_MANES	cassava4.1_004474m	Neutral/alkaline invertase	No
tr|I0CL57|I0CL57_MANES	cassava4.1_003183m	Vacuolar invertase	No
tr|J9UMU3|J9UMU3_MANES	cassava4.1_025414m	Cell wall invertase	No
tr|K0E5T2|K0E5T2_MANES	cassava4.1_009099m	Acyl-[acyl-carrier-protein] desaturase (EC 1.14.19.2)	G
tr|Q1AP39|Q1AP39_MANES	cassava4.1_014065m	14-3-3 protein	GP
tr|Q1WLP2|Q1WLP2_MANES	cassava4.1_011031m	Sucrose transporter type 2	No
tr|Q1WLP4|Q1WLP4_MANES	cassava4.1_006194m	Sucrose transporter type 4	G
tr|Q1WLP5|Q1WLP5_MANES	cassava4.1_004783m	Neutral/alkaline invertase (EC 3.2.1.26)	G
tr|Q52QX6|Q52QX6_MANES	cassava4.1_012494m	ACC oxidase ACCO2	GP
tr|Q52QX8|Q52QX8_MANES	cassava4.1_007020m	Cysteine protease CP1	No
tr|Q52QX9|Q52QX9_MANES	No_in_Phytozome	Aldo/keto reductase AKR	P
tr|Q58ZE5|Q58ZE5_MANES	cassava4.1_018289m	Superoxide dismutase [Cu-Zn] (EC 1.15.1.1)	P
tr|Q5PYQ5|Q5PYQ5_MANES	cassava4.1_015521m	Chloroplast oxygen-evolving enhancer protein	G
tr|Q94C45|Q94C45_MANES	cassava4.1_034377m	Phenylalanine ammonia-lyase (EC 4.3.1.24)	G
tr|Q94F89|Q94F89_MANES	cassava4.1_002628m	Phenylalanine ammonia-lyase (EC 4.3.1.24)	GP
tr|Q9T4H0|Q9T4H0_MANES	cassava4.1_017170m	Ribulose bisphosphate carboxylase small chain (EC 4.1.1.39)	GP
tr|Q9TLH3|Q9TLH3_MANES	cassava4.1_017170m	Ribulose bisphosphate carboxylase small chain (EC 4.1.1.39)	G

[a] Protein names corresponded to the MePPI-In in Table S1.

[b] Expression support information; GP: both gene and protein expression support; G: gene expression support; P: protein expression support and ; No: no expression support
