# Supplementary material for: Prediction of cassava protein interactome based on interolog method
Source: Sci Rep. 2017 Dec 8;7:17206. doi: 10.1038/s41598-017-17633-2 (PMC5722940; doi:10.1038/s41598-017-17633-2)
Supplement: Supplementary file 3 — Supplement Table S2 [file 41598_2017_17633_MOESM3_ESM.pdf]

Prediction of cassava protein interactome based on interolog method

Ratana Thanasomboon,

Saowalak Kalapanulak,

Supatcharee Netrphand,

Treenut Saithong*

Protein_A	Protein_B	Protein Symbol A	Protein Symbol B	References*
cassava4.1_003447m	cassava4.1_004026m	MF_1	MF_2	[66]
cassava4.1_017409m	cassava4.1_018531m	RPL18ae/LX	SH3L	[65]
cassava4.1_009356m	cassava4.1_019995m	M3	SUMO1	[65]
cassava4.1_016348m	cassava4.1_019402m	BBC	NFU4	[65]
cassava4.1_000475m	cassava4.1_002589m	EMB1989	GCN3	[65]
cassava4.1_008521m	cassava4.1_010685m	HSR8	DFR	[45]
cassava4.1_006175m	cassava4.1_006449m	GH9B1	CEL3	[45]
cassava4.1_003144m	cassava4.1_002955m	BIP	HSC70-5	[52]
cassava4.1_002955m	cassava4.1_008510m	HSC70-5	J2	[52]
cassava4.1_003274m	cassava4.1_003946m	ECT5	TPDPD	[65]
cassava4.1_003946m	cassava4.1_019747m	TPDPD	SNR	[65]
cassava4.1_011958m	cassava4.1_012375m	URH2	TSC13	[45]
cassava4.1_009245m	cassava4.1_012375m	SAM2	TSC13	[45]
cassava4.1_003901m	cassava4.1_012149m	PES	RRB	[66]
cassava4.1_002025m	cassava4.1_003901m	NOC2P	PES	[66]
cassava4.1_002025m	cassava4.1_012149m	NOC2P	RRB	[66]

*Co-expression was derived from the time-series expression datasets reported by Yang et al [45], cassava fibrous, intermediate and storage roots at 4 months old; Li et al [65], cassava leaves, stems and roots harvested at 2nd, 4th, 7th and 10th month; An et al [66], cassava apical shoots subjected to cold at 7°C for 0, 4 and 9 h; Naconsie et al [52], cassava storage roots harvested after 3, 6, 9, and 12 months of planting.
